# Compound 331 selectively induces glioma cell death by upregulating miR-494 and downregulating CDC20

**DOI:** 10.1038/srep12003

**Published:** 2015-07-08

**Authors:** Lei Zhang, Tianhui Niu, Yafei Huang, Haichuan Zhu, Wu Zhong, Jian Lin, Yan Zhang

**Affiliations:** 1 State Key Laboratory of Biomembrane and Membrane Biotechnology, College of Life Sciences, PKU-IDG/McGovern Institute for Brain Research, Peking University, Beijing, 100871, China; 2 Synthetic and Functional Biomolecules Center, College of Chemistry and Molecular Engineering, Peking University, Beijing, China; 3 Laboratory of Computer-Aided Drug Design & Discovery, Beijing Institute of Pharmacology and Toxicology, Beijing, China; 4 Aviation Medicine Research Laboratory, The General Hospital of the Air Force, Beijing, China

## Abstract

Malignant gliomas are the most common malignant tumors in the central nervous system (CNS). Up to date, the prognosis of glioma is still very poor, effective therapy with less side-effect is very necessary. Herein, we identify a compound named as “331” selectively induced cell death in glioma cells but not in astrocytes. Compound 331 upregulated miR-494 and downregulated CDC20 in glioma cells but not in astrocytes. These results suggest that compound 331 could be a potential drug selectively targeting glioma cells through upregulating miR-494 and downregulating CDC20.

Approximately 80% of malignant tumors that develop in the central nervous system (CNS) are malignant gliomas^1^. Despite some advances in the treatment of glioma, the prognosis of this disease remains very poor^2^. Glioblastomas are the most aggressive gliomas accounting for more than 50% of gliomas^3^. The median survival time for patients with glioblastoma is only about 15 months^4^.

The current standard treatments for glioblastoma include surgery, radiation, and cytotoxic chemotherapy^5^. Surgery is the recommended first step in the treatment of glioblastoma^6^. However, the infiltrative nature of glioblastoma always makes it difficult to completely remove the tumor tissue. Radiation therapy, in addition to chemotherapy, is the standard for the treatment of glioblastoma^5^. Temozolomide is a frequently used cytotoxic agent. The combination of temozolomide and radiation therapy has shown a markedly increased survival rate^7^. Nonetheless, the median survival time for patients with glioblastoma is still only about 15 months despite the advancement of the current standard treatment methods. New strategies of treatment are in the process of implementation, including targeted therapy, antiangiogenic therapy and immunotherapy. A representative example of targeted therapy is directed towards the EGFR (epidermal growth factor receptor) kinase by erlotinib and gefitinib, but the clinical trials have yet to produce successful results^8^,^9^. Antiangiogenic therapy is also a hopeful therapy, but is suffering from the lack of sustainable response with the use of antiangiogenic agents^5^. The responses to immunotherapy shown in other kinds of cancers indicated great potential for treatment in glioblastoma, although the application of immunotherapy to glioblastoma is in its preliminary stage^5^. The new strategies for the treatment of glioblastoma are exciting and promising, but almost all of the new strategies have several problems, thus, further study regarding these new therapies are necessary and more novel trials are needed to advance the level of glioblastoma therapy.

MicroRNAs (miRNAs) are a class of endogenous non-coding RNAs that play important roles in cells through targeting mRNAs to control the expression of specific genes^7^,^10^,^11^. The alterations of miRNA expression levels in various cancers have been observed^12^. For glioma, using high throughput sequencing and microarray-based technology, several studies have observed multiple alterations of miRNA expression levels in human glioma tissue samples^12^,^13^,^14^,^15^,^16^,^17^,^18^. Several lines of evidence have shown that microRNA-494 (miR-494) plays an important role in many kinds of cancers^19^,^20^,^21^,^22^. One potential target for miR-494 is the cell division cycle protein 20 (CDC20)^23^, a key regulator in cell cycle^24^,^25^. Overexpression of miR-494 significantly downregulates the level of CDC20^23^. CDC20 is one of the cofactors of the anaphase-promoting complex/cyclosome (APC/C)^26^. APC/C-CDC20 complex plays a key role during mitotic exit^26^. CDC20 is related to mitotic catastrophe^27^,^28^, which refers to a type of cell death triggered by aberrant mitosis^29^. Due to the crucial role in the cell cycle, CDC20 is considered to be a potential target for cancer therapy^24^,^30^.

In the present study, we found that an iron chelator named as “331” could selectively decrease cell viability of human glioma (U251 and SF767) cells and rat glioma (C6) cells. On the other hand, compound 331 did not affect the viability of normal rat astrocytes. MiR-494 was upregulated in glioma cells treated with compound 331 but not in rat astrocytes. Meanwhile, CDC20 was downregulated in glioma cells treated with compound 331. The decrease of cell viability in glioma cells induced by compound 331 was followed by apoptosis. Our data suggests that compound 331 has the potential to selectively induce glioma cell death by selectively upregulating miR-494 and downregulating CDC20 in glioma cells.

## Results

### Compound 331 selectively induced cell death in glioma but not in astrocytes

**Figure 1:**
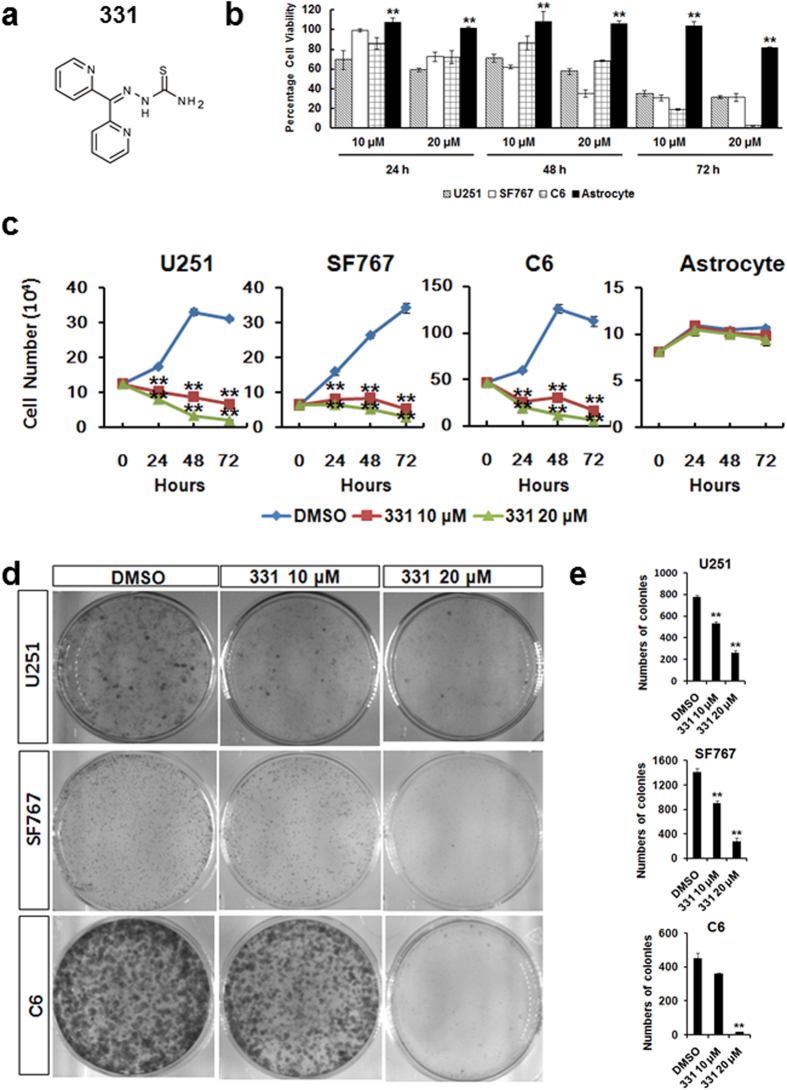
Compound 331 selectively induced cell death in glioma cells. **(a)** The structure of compound 331. **(b)** Compound 331 treatment induced cell death in human glioma cells (U251, SF767) and rat glioma cells (C6) but not in normal rat astrocytes. All cells were treated with compound 331 at 10 μM or 20 μM for 24 h, 48 h and 72 h. Data represents the mean ± S.E.M. of three independent experiments. **p < 0.01 compared with glioma cells. **(c)** Compound 331 treatment induced significant proliferation inhibition in human glioma cells (U251, SF767) and rat glioma cells (C6) but not in normal rat astrocytes. All cells were treated with compound 331 at 10 μM or 20 μM for 24 h, 48 h and 72 h. Data represents the mean ± S.E.M. of three independent experiments. **p < 0.01 compared with control. **(d)** Glioma cells were treated with DMSO as control or compound 331 (10 μM, 24 h or 20 μM, 24 h). The cells were then collected and 3000 cells were incubated in medium for an additional 10 days. Cells were then stained with crystal violet (0.4 g/L). **(e)** The numbers of colonies per dish. Data represents the mean ± S.E.M. of three independent experiments. **p < 0.01 compared with the controls (DMSO).

Compound 331 has been reported as an iron chelator^31^ (Fig. 1a). Compound 331 treatment significantly induced cell death in human and rat glioma cells including U251, SF767 and C6 cells at 10 μM and 20 μM at 24 h, 48 h and 72 h (Fig. 1b). In contrast, it did not affect cell viability in rat astrocytes (Fig. 1b). The proliferation of glioma cells was markedly inhibited by compound 331 while it did not significantly affect the total cell numbers of rat astrocytes after treatment of 10 μM or 20 μM for 24 h, 48 h and 72 h (Fig. 1c). Incubated with 20 μM for 24 h, compound 331 also inhibited the formation of colonies of glioma cells (Figs 1d,e). These results indicate that compound 331 has the potential to selectively induce cell death in glioma, but does not affect astrocytes.

**Figure 2:**
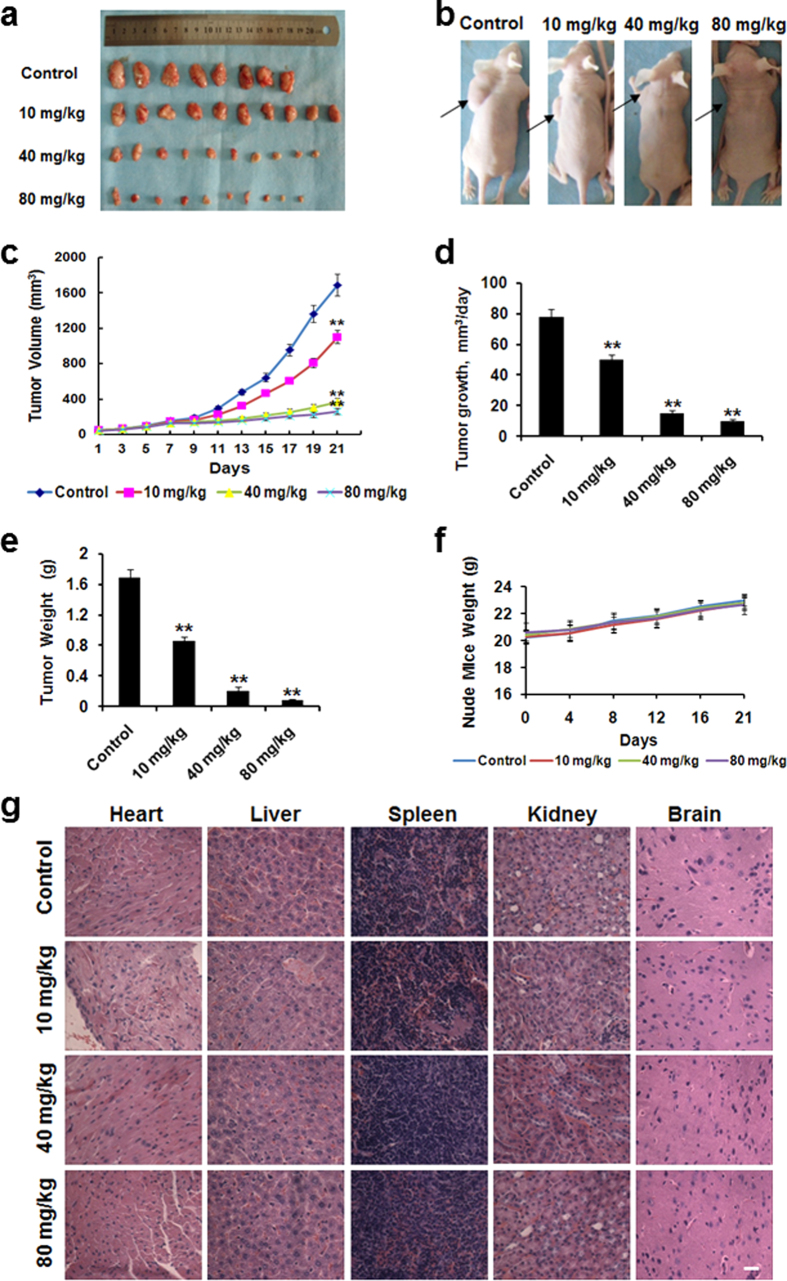
*In vivo* anti-tumor effects of compound 331. **(a, b)** Inhibition of mammary tumor growth by compound 331 treatment. Experiments were carried out using 6 week old female nude mice (BALB/c-nu), when tumor sizes had grown to about 50 mm^3^, compound 331 (10 mg/kg, 40 mg/kg, 80 mg/kg, 0.9% saline as control) was intragastricly administrated daily for three weeks. **(c)** Effects of compound 331 on the volume of human tumor xenografts. **(d)** Effects of compound 331 on the tumor growth rate. **(e)** Effects of compound 331 on the weights of human tumor xenografts. **(f)** Effects of compound 331 on the weights of the nude mice. **(g)** The H&E staining of sections from hearts, livers, spleens, kidneys and brains in control group and nude mice treated with compound 331 (10 mg/kg, 40 mg/kg and 80 mg/kg) for 21 days. Scale bar: 10 μm. **(c–f)** Data represents the mean ± S.E.M. (n = 8). **p < 0.01 compared with control.

We then tested the anti-tumor efficacy of compound 331 in tumor xenografts in immuno-deficient mice. Human U251 glioma cells were subcutaneously implanted into flanks of female nude mice. When tumor sizes had grown to approximately 50 mm^3^, the mice were intragastrically administrated with compound 331 daily for three weeks. Tumor growth was significantly inhibited by compound 331 administration compared with the controls (Figs 2a–e). Compound 331 treatments particularly reduced the daily tumor growth rate significantly (Fig. 2d), yet, the body weight of the mice did not change significantly (Fig. 2f). Furthermore, histological results (H&E staining) of hearts, livers, spleens, kidneys and brains of the mice showed no significant damage in these organs with compound 331 treatment (Fig. 2g). Our data indicated the safety and significant anti-tumor efficacy *in vivo* of compound 331 at the dosage of 10 mg/kg, 40 mg/kg and 80 mg/kg.

### Compound 331 treatment induced global changes of gene expression in glioma cells

**Figure 3:**
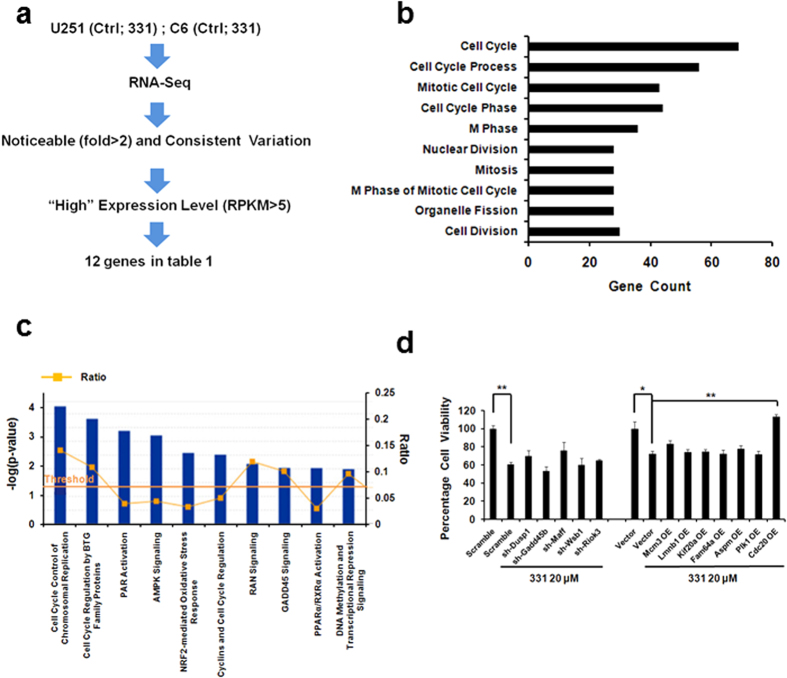
Global changes of gene expression in glioma cells treated with compound 331. **(a)** Schematic representation of the protocol used to choose genes which were highly expressed (RPKM >5) and whose RNA levels changed noticeable (fold >2 or fold <−2) and consistent in compound 331 treated U251 and C6 compared with the controls. **(b)** The function of genes (fold >1 or fold <−1, p value < 0.01) analyzed by DAVID. **(c)** Ingenuity Software Analysis (IPA) report. The RNA-seq data were arranged by signaling pathways in order of statistical significance. The top ten ranked pathways are listed (The full report is shown in Fig. S1). Ratio = number of genes in specific pathway/total number of genes uploaded to IPA. **(d)** Overexpression of CDC20 could rescue the decreased cell viability induced by compound 331. Data represents the mean ± S.E.M. of three independent experiments. *p < 0.05, **p < 0.01.

To identify genes differentially regulated in compound 331 induced cell death, DMSO treated control cells and compound 331 treated cells were collected and processed for RNA-Seq. Human glioma cell U251 and rat glioma cell C6 were prepared for RNA-Seq respectively. The sequencing data were analyzed to identify genes that were involved in compound 331 induced cell death. Compared with the control, there were 767 genes that were upregulated (fold >1, p value < 0.05), while 1780 genes that were downregulated (fold <−1, p value < 0.05) in compound 331 treated U251 cells and 4846 genes that were upregulated (fold >1, p value < 0.05), while 2517 genes that were downregulated (fold < −1, p value < 0.05) in compound 331 treated C6 cells (Supplementary Table S1 and Table S2). Furthermore, there were 357 genes that changed significantly (fold >1 or fold < −1, p value < 0.01) compared with the controls and consistent in compound 331 treated U251 and C6 cells. We uploaded the 357 genes to the DAVID knowledge database, a functional annotation tool for gene functional classification (http://david.abcc.ncifcrf.gov/). Classification of these genes showed that the top one category are cell cycle-related genes (Fig. 3b), indicating that most of the changed genes in compound 331 treated U251 and C6 cells were related to the cell cycle. In addition, we analyzed the 357 genes with IPA software (Ingenuity Systems Inc.), which can provide a global functional analysis of the RNA-Seq data and statistically rank various pathways depending on significance. Our data showed that the top two ranked pathways were also cell cycle-related pathways (Fig. 3c and Supplementary Fig. S1).

To identify the genes that played key roles in compound 331 induced glioma cell death, we chose genes with remarkable RNA level changes (fold >2 or fold < −2) that are consistent in both compound 331 treated U251 and C6 cells compared with the controls. Among these genes, we chose those that were highly expressed (RPKM >5) (Fig. 3a), resulting in 12 candidate genes (Table 1). We then did a rescue experiment with these 12 candidate genes to identify the genes involved in compound 331 induced cell death. U251 cells were transfected with shRNAs against the genes that were upregulated and the plasmids of the genes that were downregulated were overexpressed, before treatment with compound 331. After treatment with compound 331 at 20 μM for 24 h, U251 cells transfected with CDC20 overexpression plasmid showed the most significant increase in cell viability compared with other compound 331 treated groups (Fig. 3d). CDC20 is a key regulator in the cell cycle, and the global analysis results of DAVID and IPA, as well as the rescue experiments data suggested that CDC20 may indeed play a key role in compound 331 induced cell death.

### Compound 331 downregulated CDC20 expression significantly in glioma cells

**Figure 4:**
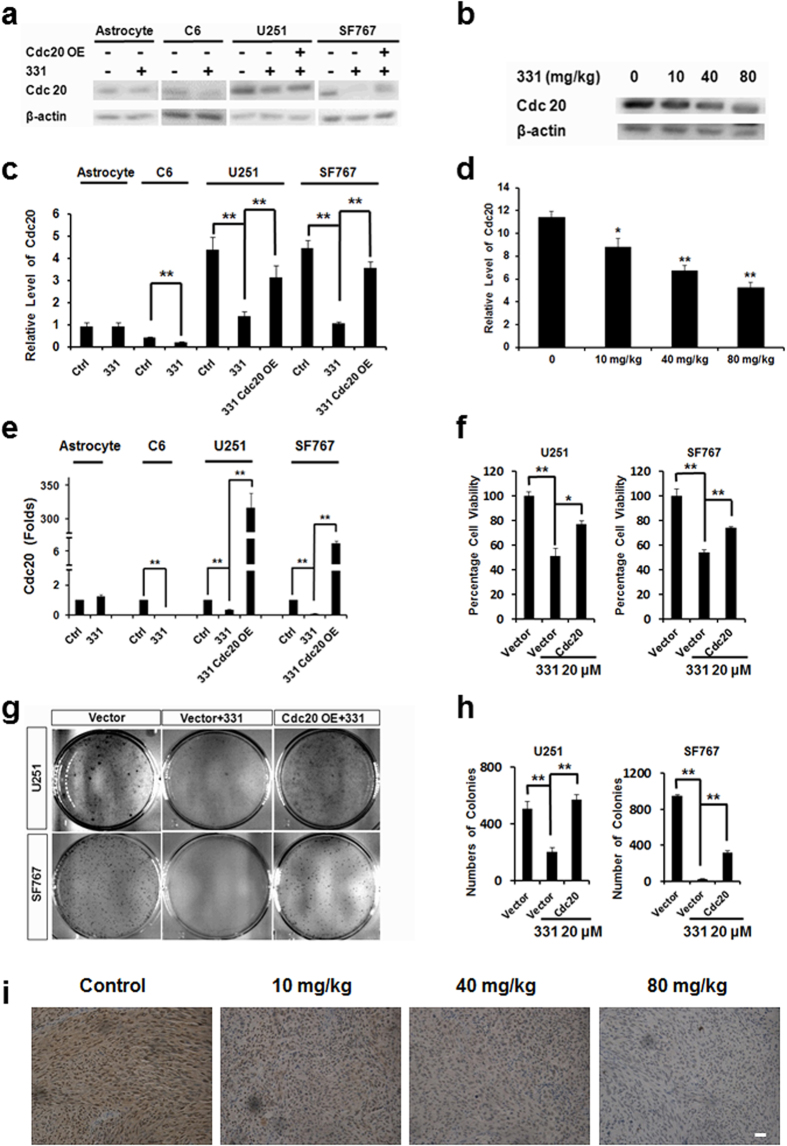
Compound 331 downregulated CDC20 in glioma cells. **(a)** Western blot analysis of Cdc20 in glioma cells and astrocytes after treatment with compound 331 (20 μM, 48 h) or transfection with CDC20 overexpressing plasmid (24 h before treated with compound 331). **(b)** Western blot analysis of CDC20 in tumor excised from the control group and nude mice administrated by 10 mg/kg, 40 mg/kg, 80 mg/kg 331. **(c)** Quantification of western blotting data in **a**. Data represents the mean ± S.E.M. of three independent experiments. *p < 0.05, **p < 0.01. **(d)** Quantification of western blotting data in **b**. Data represents the mean ± S.E.M. of three independent experiments. *p < 0.05, **p < 0.01. **(e)** Quantitative RT-PCR analysis of CDC20 in glioma cells and astrocytes after treatment with compound 331 (20 μM, 48 h) or transfection with CDC20 overexpressing plasmid (24 h before treated with compound 331). Data represents the mean ± S.E.M of three independent experiments. **p < 0.01. **(f)** Cell viability of glioma cells after treatment with compound 331 (20 μM, 48 h) or transfection with CDC20 overexpressing plasmid (24 h before treatment with compound 331). Data represents the mean ± S.E.M. of three independent experiments. *p < 0.05, **p < 0.01. **(g)** Glioma cells were treated with compound 331 (20 μM, 24 h) or transfected wtih CDC20 overexpressing plasmid (24 h before treated with compound 331). The cells were stained with crystal violet (0.4 g/L). **(h)** Statistics of the numbers of colonies per dish. Data represents the mean ± S.E.M. of three independent experiments. **p < 0.01. **(i)** Representative images of CDC20 staining in the tumors excised from nude mice treated with compound 331 (10 mg/kg, 40 mg/kg and 80 mg/kg) for 21 days. Scale bar: 50 μm.

Western blot and RT-PCR data confirmed that CDC20 was downregulated by compound 331 treatment both *in vitro* in cultured glioma cells (20 μM, 48 h, Figs 4a,c,e) and *in vivo* in nude mice (Figs 4b,d). In astrocytes, CDC20 levels did not significantly alter at the mRNA level nor protein level after treated with compound 331 (20 μM, 48 h) (Figs 4a,c,e). Overexpression of CDC20 could significantly increase cell viability of glioma cells treated with compound 331 (20 μM, 48 h) (Fig. 4f). In addition, overexpression of CDC20 could also significantly rescue the glioma cells ability to form colonies (Figs 4g,h). At the same time, CDC20 was also significantly downregulated in the tumor xenografts from the nude mice treated with 331 (10 mg/kg, 40 mg/kg, 80 mg/kg) (Fig. 4i).

### Compound 331 induced mitotic catastrophe and apoptosis in glioma cells

**Figure 5:**
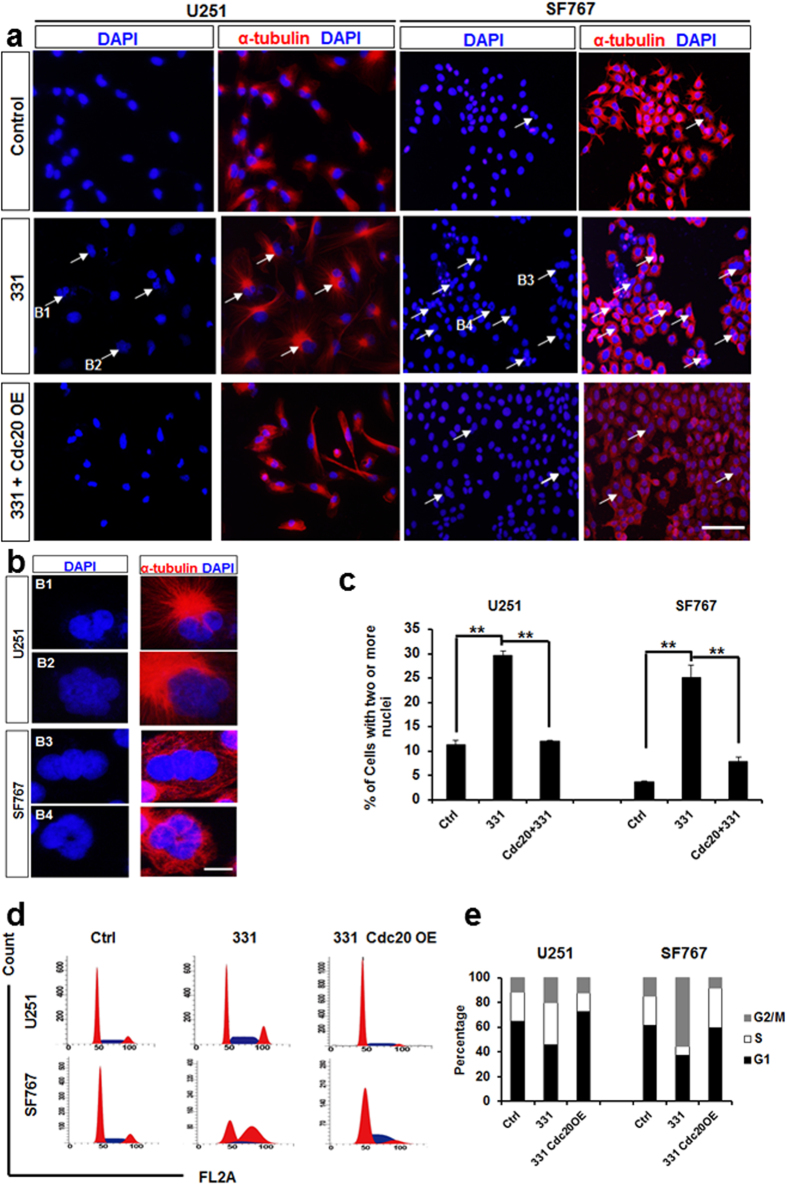
Compound 331 induced mitotic catastrophe in glioma cells. **(a)** Representative micrograph images obtained from glioma cells treated with compound 331 (20 μM, 48 h) or transfection wtih CDC20 overexpression plasmid (24 h before treated with compound 331). The arrows indicate cells with two or more nuclei. The scale bar represents 200 μm. **(b)** High-magnification images of cells in **a**. The scale bar represents 20 μm. **(c)** Quantification of cells with two or more nuclei. The cells with two or more nuclei were counted in 5 random fields, and 100 cells were counted every field. Data represents the mean ± S.E.M. of three independent experiments. **p < 0.01. **(d)** Cell cycle analysis. Treatment with compound 331 (20 μM, 48 h) induced accumulation of cells in G2/M phase. **(e)** Quantification of cell cycle distribution (G1, S and G2/M) in glioma cells treated with compound 331 (20 μM, 48 h) or meanwhile transfected wtih CDC20 overexpressing plasmid (24 h before treated with compound 331).

**Figure 6:**
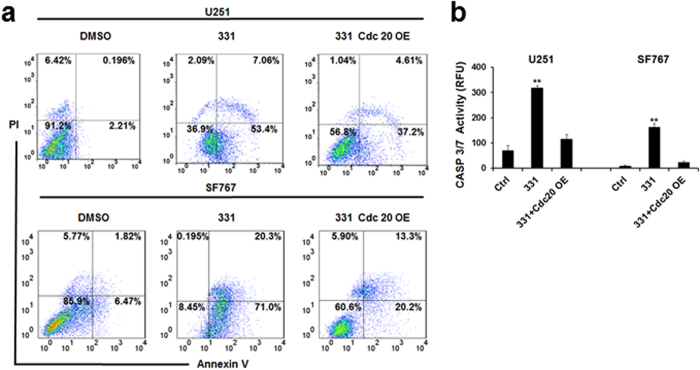
Compound 331 induced apoptosis in glioma cells. **(a)** Flow cytometric analysis for annexin V and PI in glioma cells treated with compound 331 (20 μM, 48 h) or transfection wtih CDC20 overexpressing plasmid (24 h before treated with compound 331). Percentage of annexin V-positive and PI-negative cells (bottom right) were indicated. **(b)** Proteolytic activities of caspase-3/7 induced by compound 331 (20 μM, 48 h) and rescued by overexpression of CDC20 (transfected 24 h before treatment with compound 331). Data represents the mean ± S.E.M. of three independent experiments. **p < 0.01 compared with the control groups.

There has been increasing evidence suggesting that CDC20 is related to mitotic catastrophe^27^,^28^,^32^. Our data demonstrated that treatment with compound 331 (20 μM, 48 h) increased the percentage of cells with multi-nuclei (Figs 5a,b,c), a character of mitotic catastrophe^33^,^34^, and induced significant G2/M arrest in glioma cells. CDC20 overexpression could further rescue the increase of multi-nuclei cells and G2/M arrest in compound 331 treated glioma cells (Figs 5d,e). Furthermore, flow cytometric analysis data indicated that compound 331 (20 μM, 48 h) significantly increased the percentage of annexin V-positive and PI-negative cells (Fig. 6a). In addition, compound 331 (20 μM, 48 h) could significantly increase the enzymatic activities of caspase-3/7 in glioma cells (Fig. 6b), indicating an increase of apoptosis in treated glioma cells. Similarly, CDC20 overexpression decreased the percentage of annexin V-positive and PI-negative cells (Fig. 6a) and the enzymatic activities of caspase-3/7 (Fig. 6b) in glioma cells treated with compound 331 (20 μM, 48 h). In conclusion, compound 331 downregulated CDC20 in glioma cells and ensued in the accumulation of multi-nucleus cells, G2/M arrest, and cell apoptosis. Our data suggests that compound 331 induces mitotic catastrophe, which results in apoptosis by downregulating CDC20 in glioma cells.

### Compound 331 selectively downregulated miR-494 in glioma cells

**Figure 7:**
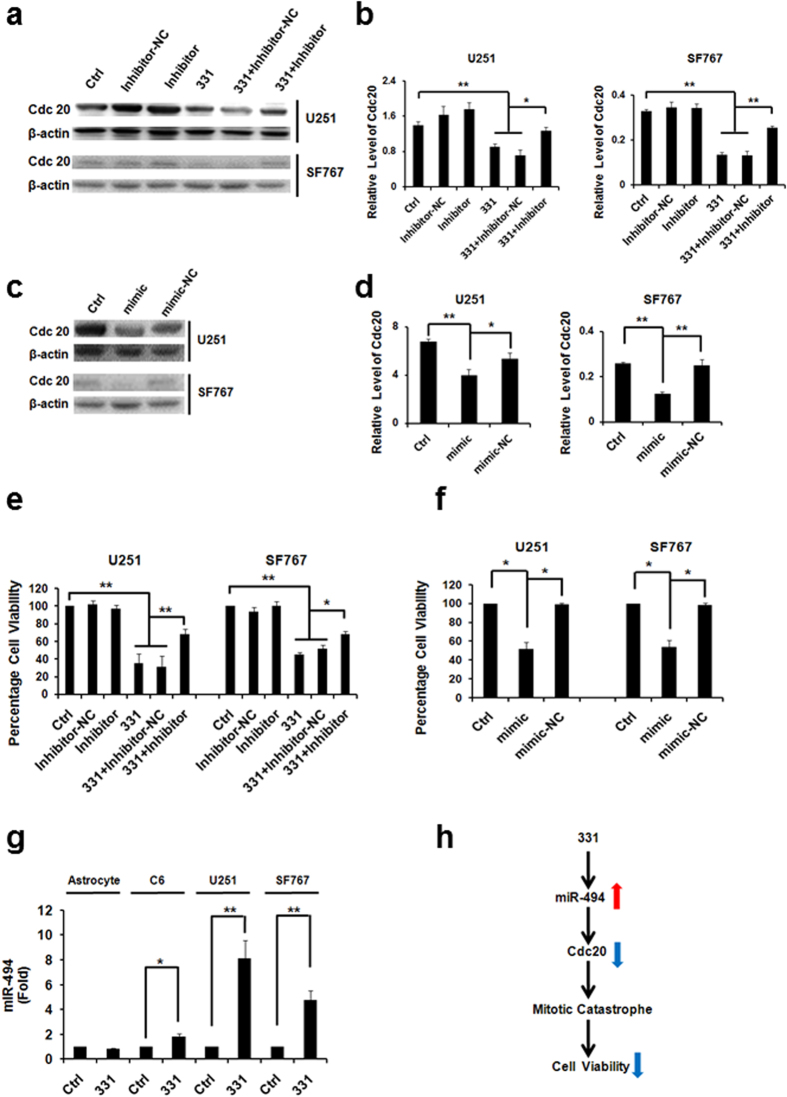
Compound 331 selectively downregulated miR-494 in glioma cells. **(a)** Western blot analysis of CDC20 in glioma cells treated with compound 331 (20 μM, 48 h) or transfection with miR-494 inhibitor (24 h before treated with 331). “Inhibitor-NC” refers to “Inhibitor negative control”. **(b)** Quantification of western blotting data in **(a)**. Data represents the mean ± S.E.M. of three independent experiments. **p < 0.01. **(c)** Western blot analysis of CDC20 in glioma cells transfected with miR-494 mimic for 72 h. “mimic-NC” refers to “mimic negative control”. **(d)** Quantification of western blotting data in **(c)**. Data represents the mean ± S.E.M. of three independent experiments. **p < 0.01. **(e)** MiR-494 inhibitor (24 h before treated with compound 331) could significantly rescue the cell death induced by compound 331 (20 μM, 48 h). Data represents the mean ± S.E.M. of three independent experiments. *p < 0.05, **p < 0.01. **(f)** Mimic-miR-494 (72 h) could induce cell death in glioma cells. Data represents the mean ± S.E.M. of three independent experiments. *p < 0.05. **(g)** Quantitative RT-PCR analysis of miR-494 in glioma cells treated with compound 331 (20 μM, 48 h). Data represents the mean ± S.E.M. of three independent experiments. *p < 0.05, **p < 0.01. **(h)** Schematic drawing for the pathway of compound 331 induced cell death.

It has been demonstrated that the overexpression of miR-494 significantly downregulates the expression of CDC20^23^, and miR-494 overexpression could suppress cancer cell proliferation^19^,^20^,^21^. Based on the results from the Western Blots, overexpression of miR-494 could significantly downregulate CDC20 while inhibiton of miR-494 could upregulate the expression of CDC20 in glioma cells treated with compound 331 (Figs 7a–d). Overexpression of miR-494 for 72 h could significantly decrease the cell viability of glioma cells and inhibiton of miR-494 could rescue the decreased cell viability of glioma cells induced by compound 331 (Figs 7e,f). The level of miR-494 was also upregulated in glioma cells treated with compound 331 compared with the controls. It is important to note that there were no significant changes of miR-494 expression level in astrocytes treated with compound 331 compared with the controls (Fig. 7g). These results suggest that miR-494 might play a significant role in compound 331 induced cell death.

## Discussion

We have identified a new compound that could selectively and significantly reduce viability in human and rat glioma cells but not in astrocytes. It is well accepted that selectivity is critical in cancer therapy. The *in vivo* data presented here, shows that compound 331 does not cause significant damage in major organs of mice, suggesting that compound 331 might become a potential highly selective glioma treatment. Our data also demonstrated that such selectivity depends on the selective upregulation of miR-494 in glioma cells but not in astrocytes. Compound 331 selectively upregulated miR-494 and downregulated CDC20 in glioma cells but not in astrocytes. The downregulation of CDC20 induced mitotic catastrophe in glioma cells further leads to cell death of glioma cells (Fig. 7h). We also found that a high dosage of compound 331 also induces cell death in astrocytes (Fig. S2), which indicates that only a specific range of dosage of compound 331 can induce selective killing of glioma cells. Further investigative efforts are necessary to elaborate on this topic, thus the rationale explaining why the high dosage cause astrocytes die is still unclear. Therefore, it is evident that the application of compound 331 to selectively kill glioma cells should be based on an appropriate dosage range, although this appropriate range should be identified.

Based on the high throughput RNA sequencing data, there were large numbers of mRNA in glioma cells treated with compound 331. Among these altered genes, our data has shown that CDC20 overexpression could adequately rescue the decreased cell viability or other alterations caused by compound 331 treatment, such as mitotic catastrophe and apoptosis. Therefore, our data suggests that CDC20 downregulation could be one of the mechanisms for the decreased cell viability caused by compound 331 in glioma cells.

There have been several studies suggesting that miRNAs can act as targets for glioma therapy^13^,^35^,^36^,^37^,^38^,^39^,^40^,^41^. Nonetheless, therapeutic methods based on miRNAs are still limited and one of the obstacles is to effectively deliver the miRNAs to the tumors^10^. Numerous studies have tried to optimize the delivery of miRNA to the target^42^,^43^. In this study, we attempt to alter the miRNA level by a compound instead of focusing on the delivery of miRNAs to the target. There are currently very few studies reporting that a compound can indeed alter the miRNA level selectively in different types of cells. Our research is the first, to our knowledge, to report that a compound could selectively regulate a certain miRNA in glioma cells but not in astrocytes. Our results indicate that compound 331 can be a potent and selective treatment for glioma.

## Methods

### Compound 331

Compound 331 was dissolved in DMSO as 100 mM stock solution. 0.2‰ DMSO in medium as a control for the experiments conducted.

### Cell culture and cell lines

Rat astrocytes were cultured from newborn Sprague Dawley rat brains. All protocols in this study had been approved by Peking University Institutional. Animal Care and Use Committee (IACUC) and are in accordance with the NIH Guidelines regarding the care and use of animals for experimental procedures. In brief, the fresh rat brains were dissociated with 0.25% trypsin (Invitrogen), and then inactivated by fetal bovine serum (FBS, HyClone). The mixture was triturated through a pipette, and filtered through the 70 μm sterilizedfilters. The flowthrough was centrifuged. Then pellet was then washed once with phosphate buffered saline (PBS, 0.003 M KCl, 0.14 M NaCl, 0.01 M Na_2_HPO_4_, 0.002 M KH_2_PO_4_, pH 7.2). The cells were then plated on plates at a density of 5 × 10^4^ cells/mL. The cells were rocked in a covered culture flask at 250 g for 18 h until the cells were confluent on the surface of the culture flask to obtain the astrocyte culture. Rat C6 glioma cells, human U251 glioma cells and SF767 glioma cells were obtained from Cell Resource Center of Peking Union Medical College (Beijing, China). C6 glioma cells were incubated at 37 °C in DMEM (Dulbecco’s Minimum Essential Medium, Gibco) with 10% FBS without phenol red and with 5% circulating CO_2_. The medium was changed every 48 hours. U251 glioma cells and SF767 glioma cells were incubated at 37 °C in MEM-EBSS (Minimum Essential Medium Eagles with Earle’s Balanced Salts Solution, Hyclone) medium with 10% FBS without phenol red and with 5% circulating CO_2_. The medium was changed every 48 hours.

### Cell viability and clonogenetic assay

Cell viability was measured by CellTiter 96^®^ AQueous One Solution Cell Proliferation Assay (Promega). Approximately 1000 cells were plated in each well on a 96-well plate at 37° in a humidified 5% CO_2_ for 24 h prior to treatment with compounds or transfection reagent. After treatment, 20 μL of CellTiter 96^®^ AQueous One Solution reagent was added to each well, and the cultures were incubated for 1–2 h at 37 °C and humidified with 5% CO_2_. The OD value was then measured with a Varioskan Flash reader (Thermo) at 490 nm. The percentage of cell viability = [(OD_treated_ – OD_blank_)/(OD_control_ – OD_blank_)] × 100%. Approximately 5000 cells were plated in each well on a 24-well plate at 37 °C in a humidified 5% CO_2_ for 24 h before treatment with compounds or transfection reagents. Cell numbers were counted with a hematocytometer after the cells were stained with trypan blue (Invitrogen). For the clonogenetic assay, glioma cells were treated with compound 331 (20 μM, 24 h) or transfected wtih CDC20 overexpression plasmid (24 h before treated with compound 331). The cells were then collected and 3000 cells were incubated in medium for an additional 10 days. The cells were then stained with crystal violet (0.4 g/L) and imaged.

### Tumor xenograft experiments

The protocol was approved by Peking University Institutional Animal Care and Use Committee (IACUC) and followed the NIH Guidelines regarding the care and use of animals for experimental procedures. Experiments were carried out using 6 week old female nude mice (BALB/c-nu). U251 cells were harvested and suspended in PBS and then injected subcutaneously, into the flanks of the mice (8 × 10^6^ cells/flank). After injection, when tumor sizes had grown to about 50 mm^3^, compound 331 (10 mg/kg, 40 mg/kg, 80 mg/kg, 0.9% saline as control) was intragastricly administrated daily for three weeks. The tumor sizes in every group were measured using calipers and the tumor volume was estimated according to the formula: tumor volume (mm^3^) = L (Length) × W (Width)^2^/2. For histological study, tumor tissues were fixed in 10% formalin.

### Histopathology

Tissues from nude mice were harvested and fixed in 10% formalin. The histological examination used standard techniques. The heart, liver, spleen, kidney and brain were embedded in paraffin, sectioned, and stained by hematoxylin and eosin (H&E). After staining, the slides were imaged using an optical microscope.

### RNA-seq

The total RNA was extracted from compound 331 treated cells and control cells using the RNeasy mini Kit (Qiagen). The sample was then run using a fragmented RNA on a RNAPico 6000 chip in an Agilent 2100 Bioanalyzer (Agilent). In brief, mRNAs with poly(A) tails were isolated from the total RNA and converted to cDNA, then sequencing adapters were ligated to short fragments after purification using a QiaQuick PCR extraction kit. 200–700 bp fragments were separated using agarose gel electrophoresis and then subjected to 15 cycles of PCR. The prepared libraries were then sequenced using Illumina HiSeq™ 2000 (Illumina) and checked by q-PCR and Agilent 2100 Bioanalyzer. The results obtained from each group were matched to the human and rat genomes. Results that correctly aligned were subsequently analyzed. To generate gene counts, unambiguously mapped results were used first. Feature counts were normalized by the RPKM (read per kilobases per million aligned reads) method, which is able to eliminate the influence of different gene length and sequencing discrepancy on the calculation of gene expression, and thus the calculated gene expression can be directly used for comparing the differences of gene expression among groups. To detect different expression levels, the p value (one-tailed) was utilized, which corresponds to differential gene expression test (two-sample t test with equal variances). Differentially expressed gene analysis generates large multiplicity problems in which thousands of hypotheses are tested simultaneously. Corrections for false positives (type I errors) and false negatives (type II errors) were performed by the false discovery rate (FDR) method.

### Real-time PCR

RNA was isolated from cells harvested from every experiment group. cDNA was synthesized using 2 μg of total RNA by Scientific Revert Aid First Strand cDNA Synthesis Kit (Thermo) following the protocol provided by the manufacturer. 100 ng of cDNA was used for qRT-PCR. SYBR Green PCR Master Mix (Invitrogen) was used to detect miR-494 and CDC20 expression. Real time PCR reactions were performed on iCycler Real Time PCRSystem (BioRad) according to the manufacturer’s recommendations. MiRNA and mRNA quantities were calculated by the ΔCt method. The U6 shRNA qRT-PCR Primer Set (Ruibo) was used to detect the reference gene for miR-494. Expression levels for miR-494 were normalized to the U6 shRNA of each sample. Expression levels for CDC20 were normalized to the GAPDH of each sample. The sequences for primers of the human CDC20 used were as following:

forward: (5’-GAGGGTGGCTGGGTTCCT-3’); and reverse: (5’-GCCTTGACAGCCCCTTGAT-3’), and rat *Cdc20* were used as following: forward: (5’-CCCAATGCACCGATTGCT-3’); and reverse: (5’-GCTGGGCCTGTGGCTTCT-3’). Real-time PCR quantifications were run in triplicate for each sample and then averaged. The amplification efficiency of the target and housekeeping genes was approximately equivalent for utilization of the comparative Ct method for relative quantification. Amplification was done for 40 cycles at 95 °C for 10 s, 60 °C for 30 s.

### Western blots

Proteins were obtained from the cultured cells by using the cell lysis buffer (50 mM Tris, pH8.0, 150 mM NaCl, 1% NP-40, 0.1% SDS) and then the protein concentrations were measured by bicinchoninic acid (BCA) assay kit (Pierce). All protein extracts were denatured at 100 °C for 5 minutes and separated on 10% sodium dodecyl sulphate-polyacrylamide gel electrophoresis (SDS-PAGE) at 80 volts for approximately 1.5 hours. Proteins were blotting onto a Immobilon-PTM polyvynilidene fluoride (PVDF) membrane (Millipore) at 100 volts for 2 hours. The membranes were blocked with 5% bovine serum albumin in Tris buffered saline with 0.1% tween-20 (TBST) at room temperature for half an hour. Anti-CDC20 (Bioworld, 1:1000) and β-actin (Kangchen, 1:4000) were used as primary antibodies. After washing 3 times with TBST, goat anti-rabbit IgG conjugated with horseradish peroxidase (HRP) (Kangchen, 1:5000) was added at a dilution of 1:4000 as the secondary antibody and detected by enhanced chemiluminescence. The optical density was then analyzed by BioRadChemiDox (BioRad). The aggregate absolute density of CDC20/β-actin determined the relative density.

### Immunostaining

Cells were permeabilized in PBS-Triton at 4 °C and blocked by 10% bovine serum albumin at room temperature, and followed by an incubation with anti-α-tubulin antibody (Sigma, 1:200) at 4 °C overnight. Cy3-conjugated anti-rabbit antibody was applied as secondary antibody. The nuclei were stained by DAPI (10 μg/ml) for 15 minutes in the dark. The coverslips were mounted with an ImmunonTM mounting medium (Shandon) onto glass slides and the results were analyzed with a fluorescence microscope (Olympus BH2-RFCA, Olympus) and a digital camera (Olympus DP70 Digital Microscope Camera, Olympus). The cells with two or more nuclei were counted in 5 random fields, and 100 cells were counted every field.

### Cell cycle analysis

Cells were collected and washed with PBS twice, then fixed in 70% cold ethanol overnight. After incubation, cells were washed with PBS three times, then resuspended in 300 μL of PBS containing 0.1 mg/ml Rnase (Sigma), 50 μg/ml propidium iodide (Roche) and 0.2% Triton X-100 (Sigma), and incubated in the dark for 30 min at room temperature. The samples were then placed in Falcon tubes and read on a Becton Dickinson FACStarPLUS (Becton Dickinson). Data analysis was performed using the ModFit software.

### Annexin V-FITC/PI FACS (Fluorescence-activated cell sorting) apoptotic assay

According to the instruction of annexin V-FITC/PI Apoptosis Detection Kit (Solarbio), cells were tripsinized and washed twice with PBS. The cells were then resuspended in 200 μL binding buffer with 10 μL annexin V-FITC and 10 μL PI, gently mixed and incubated for 15 min in the dark at room temperature. Binding buffer (300 μL) was added to each tube and the samples were analyzed with flow cytometry Becton Dickinson FACStarPLUS (Becton Dickinson) within 1 h.

### Caspase activity assays

The caspase-3/7 activation assay was performed using Apo-One homogenous caspase-3/7 assay (Promega). Briefly, 10^4^ cells were collected from different groups and lysed in the homogeneous caspase-3/7 reagent. The lysates were then incubated at 37 °C for 4 h before reading in a fluorometer at 485/530 nm. The relative caspase-3/7 activity was given to evaluate the fold-changes of samples treated with compound 331 or combined with CDC20 overexpression compared with the controls.

### Statistical evaluation

Statistical significance was assessed by one-way analysis of variances (ANOVA). The Sheffé’s test was applied as a post hoc for the significant difference demonstrated by ANOVAs. The data were presented as the mean ± S.E.M. (standard error of the mean). Statistical significance was indicatived by p < 0.05.

## Additional Information

**How to cite this article**: Zhang, L. *et al.* Compound 331 selectively induces glioma cell death by upregulating miR-494 and downregulating CDC20. *Sci. Rep.* **5**, 12003; doi: 10.1038/srep12003 (2015).

## Supplementary Material

Supplementary Information

## Figures and Tables

**Table 1:** Genes highly expressed (RPKM > 5) with RNA levels changed noticeable (fold >2 or fold <−2) and consistent in compound 331 treated U251 and C6 compared with the controls.

Gene	Description	U251		C6	
		Fold changes	p value	Fold changes	p value
*DUSP1*	dual specificity phosphatase 1	2.77	<1 × 10^−13^	2.74	6.61 × 10^−13^
*GADD45B*	growth arrest and DNA-damage-inducible, beta	2.507	<1 × 10^−13^	5.18	<1 × 10^−13^
*MAFF*	v-maf avian musculoaponeurotic fibrosarcoma oncogene homolog F	2.467	1.31 × 10^−12^	2.95	5.14 × 10^−13^
*WSB1*	WD repeat and SOCS box containing 1	2.18	1.55 × 10^−12^	3.76	<1 × 10^−13^
*RIOK3*	RIO kinase 3	2.04	<1 × 10^−13^	2.06	<1 × 10^−13^
*MCM3*	minichromosome maintenance complex component 3	−2.32	<1 × 10^−13^	−2.23	<1 × 10^−13^
*LMNB1*	lamin B1	−2.68	<1 × 10^−13^	−2.50	<1 × 10^−13^
*KIF20a*	kinesin family member 20A	−2.72	<1 × 10^−13^	−2.13	<1 × 10^−13^
*FAM64A*	family with sequence similarity 64, member A	−2.74	<1 × 10^−13^	−3.14	<1 × 10^−13^
*ASPM*	asp (abnormal spindle) homolog, microcephaly associated (Drosophila)	−2.80	<1 × 10^−13^	−2.07	<1 × 10^−13^
*PLK1*	polo-like kinase 1	−2.81	<1 × 10^−13^	−3.94	<1 × 10^−13^
*CDC20*	cell division cycle 20	−3.45	<1 × 10^−13^	−3.41	<1 × 10^−13^
