# Supplementary material for: Compound 331 selectively induces glioma cell death by upregulating miR-494 and downregulating CDC20
Source: Sci Rep. 2015 Jul 8;5:12003. doi: 10.1038/srep12003 (PMC4495416; doi:10.1038/srep12003)
Supplement: Supplementary Information [file srep12003-s1.pdf]

Compound 331 selectively induces glioma cell death by upregulating miR-494 and downregulating CDC20

Lei Zhang[1], Tianhui Niu[2,4], Yafei Huang[3], Haichuan Zhu[2],

Wu Zhong[3*], Jian Lin[2*], Yan Zhang[1*]

[1]State Key Laboratory of Biomembrane and Membrane Biotechnology, College of Life Sciences, PKU-IDG/McGovern Institute for Brain Research, Peking University, Beijing, 100871, China

[2]Synthetic and Functional Biomolecules Center, College of Chemistry and Molecular Engineering, Peking University, Beijing, China

[3]Laboratory of Computer-Aided Drug Design & Discovery, Beijing Institute of Pharmacology and Toxicology, Beijing, China

[4]Aviation Medicine Research Laboratory, The General Hospital of the Air Force, Beijing, China

[*]Corresponding author: Yan Zhang, Ph.D. (yanzhang@pku.edu.cn), Wu Zhong, Ph.D. (zhongwu@bmi.ac.cn) and Jian Lin, Ph.D. (linjian@pku.edu.cn)

Figure Legends

Fig. S1. List of results from Ingenuity Software Analysis (IPA). The RNA-seq data were arranged by signaling pathways in order of statistical significance.

Fig. S2. High dosage of 331 could also induce cell death in astrocytes. The astrocytes and C6 glioma cells were treated with 5 μM, 10 μM, 20 μM, 30 μM compound 331 for 24 h. 30 μM of compound 331 induced significant cell death in astrocytes and C6 glioma cells. Data represents the mean ± S.E.M. of three independent experiments. *p<0.05, **p<0.01 compared with control.

Table S1. The RNA-seq report of U251 cells.

Table S2. The RNA-seq report of C6 cells.

Supplementary Figure 1

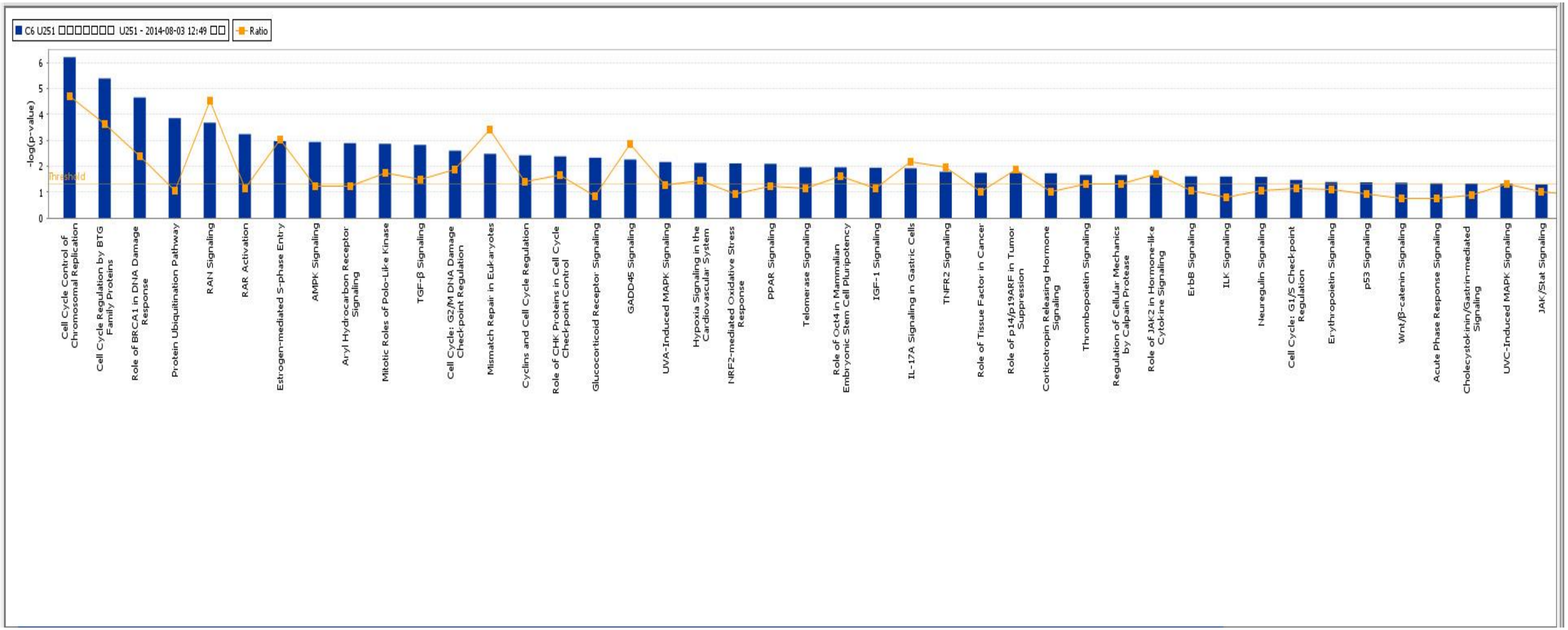

Supplementary Figure 2

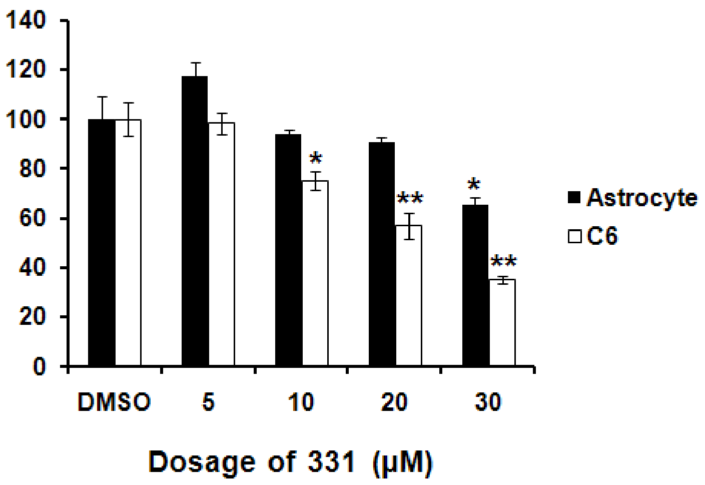

Supplementary Table1

GeneID	Gene_length	U251-DMSO-Expression	U251-331-Expression	U251-DMSO-RPKM	U251-331-RPKM	log2 Ratio(U251-3-3/U251-DMSO)	Up-Down-Regulation (331/DMSO)	P-value	FDR
HBA1	576	0	65	0.001	3.316064	11.69526	Up	0	0
HBA2	622	0	49	0.001	2.345238	11.19552	Up	1.78E-15	6.79E-15
PRTN3	1001	0	54	0.001	1.601751	10.64543	Up	0	0
BARX1	1498	0	41	0.001	0.807861	9.657964	Up	4.55E-13	1.58E-12
LHX5	2084	0	43	0.001	0.607769	9.247379	Up	1.14E-13	4.11E-13
TNNT3	1217	0	18.5	0.001	0.451429	8.818356	Up	3.81E-06	9.16E-06
KISS1	731	0	8	0.001	0.32828	8.358783	Up	0.003906	0.007649
CBFA2T3	4038	0	40	0.001	0.293598	8.197697	Up	9.09E-13	3.09E-12
CA9	1561	15	4318	0.280077	81.84002	8.190841	Up	7.51E-08	1.95E-07
C2orf66	1386	0	11.5	0.001	0.243596	7.928347	Up	0.000488	0.001031
S1PR4	1612	0	12.5	0.001	0.227386	7.829003	Up	0.000244	0.000526
MYCL1	3260	1	211	0.009269	1.917455	7.692531	Up	0.000575	0.001207
FAM110C	3963	0	25	0.001	0.185346	7.534076	Up	2.98E-08	7.89E-08
ADRA2C	1958	0	12	0.001	0.182378	7.510787	Up	0.000244	0.000526
HAND1	1749	0	8.5	0.001	0.145065	7.180554	Up	0.003906	0.007651
ADRB3	2660	0	12	0.001	0.134247	7.068741	Up	0.000244	0.000526
MT3	599	15	1798	0.731798	88.79589	6.922903	Up	7.51E-08	1.96E-07
OLIG1	2293	0	8	0.001	0.10403	6.700858	Up	0.003906	0.00765
RIMS4	5207	0	18	0.001	0.101358	6.663314	Up	3.81E-06	9.16E-06
ATG9B	4577	2.5	241.5	0.016505	1.56346	6.565683	Up	0.000115	0.000255
CHRNA4	5487	6	491	0.032625	2.645282	6.341321	Up	1.62E-05	3.75E-05
SNCB	1467	1	76	0.020598	1.533523	6.218185	Up	0.000575	0.001207

PNCK	1522	1	64	0.019854	1.241602	5.966639	Up	0.000575	0.001207
C7orf51	3581	3	180	0.024995	1.482002	5.889792	Up	7.36E-05	0.000164
KISS1R	1650	0.5	29.5	0.009157	0.528386	5.850596	Up	1.86E-09	5.16E-09
CLEC3B	886	4	233.5	0.136423	7.751723	5.828363	Up	4.59E-05	0.000104
NDUFA4L2	1275	92	4945	2.11381	114.7108	5.762012	Up	6.66E-15	2.51E-14
C2orf72	3595	15	770.5	0.120337	6.343467	5.720125	Up	7.51E-08	1.96E-07
KCNC3	3176	1	54	0.009514	0.500327	5.71662	Up	0.000575	0.001207
CREG2	3488	4	163	0.034653	1.384039	5.319752	Up	4.59E-05	0.000104
C20orf46	1720	5	187	0.085173	3.22193	5.241379	Up	2.76E-05	6.33E-05
L1CAM	5034	24.5	798.5	0.144103	4.688747	5.02403	Up	2.47E-10	7.08E-10
EGR2	2892	8	244.5	0.080415	2.505637	4.961563	Up	5.24E-06	1.25E-05
AK4	6998	555	16182	2.300095	68.57935	4.898009	Up	0	0
JAM2	1721	5	147	0.085124	2.532435	4.894817	Up	2.76E-05	6.33E-05
PPFIA4	5055	101	2836.5	0.583779	16.60567	4.83011	Up	0	0
ISM2	3006	25	693	0.250547	6.827634	4.768231	Up	1.30E-10	3.79E-10
CSF2	781	13	334.5	0.476536	12.68913	4.734863	Up	2.60E-07	6.61E-07
PTPRN	3801	5	136	0.03975	1.056708	4.732495	Up	2.76E-05	6.33E-05
HRH3	2680	0.5	13.5	0.005638	0.146495	4.699624	Up	0.000122	0.000269
GPR146	1776	43	1094.5	0.721283	18.21453	4.658382	Up	1.18E-13	4.25E-13
C5orf46	587	2	46	0.095137	2.342133	4.621673	Up	0.000115	0.000255
LOC100507405	1397	23	546	0.479428	11.56035	4.591727	Up	4.68E-10	1.33E-09
NEUROD2	3048	0.5	11	0.00458	0.104381	4.510207	Up	0.000488	0.001031
NDRG1	3123	817.5	18022.5	7.599885	170.9985	4.491863	Up	1.28E-12	4.29E-12
ENO2	2423	1206	25923.5	14.43452	316.8643	4.45627	Up	4.33E-12	1.39E-11
CDK18	3122	19	407.5	0.178754	3.868027	4.43555	Up	6.02E-09	1.63E-08

PRRT4	2411	1	22	0.012533	0.271042	4.434688	Up	0.000578	0.001213
C4orf47	974	7	144	0.210101	4.39072	4.385302	Up	9.28E-06	2.18E-05
SLC38A5	2006	9	184.5	0.130996	2.720461	4.376252	Up	2.92E-06	7.05E-06
EGLN3	2722	360.5	7206	3.849822	78.40573	4.348095	Up	4.37E-13	1.52E-12
BEND5	1342	8	162.5	0.180135	3.585955	4.315209	Up	5.24E-06	1.25E-05
DKFZp451A211	5341	86	1667.5	0.466365	9.228026	4.306492	Up	0	0
CERS1	2137	8	158	0.110974	2.18867	4.301763	Up	5.24E-06	1.25E-05
NOTUM	2281	0.5	10	0.006624	0.12931	4.287036	Up	0.000977	0.002013
LOC100507855	672	27	509	1.169705	22.41653	4.260347	Up	3.64E-11	1.08E-10
RGS16	2432	4	74.5	0.047813	0.907193	4.245942	Up	4.59E-05	0.000104
SH3D21	2333	119	2206.5	1.481314	28.02073	4.241545	Up	0	0
PDZD4	3745	13	227.5	0.10183	1.795799	4.140388	Up	2.60E-07	6.61E-07
C7orf52	2953	1	18	0.010233	0.178239	4.122531	Up	0.000615	0.001288
PMP2	3579	1	17	0.008443	0.144182	4.093982	Up	0.000651	0.001361
GPR160	2021	10	161	0.146111	2.360769	4.014115	Up	1.61E-06	3.94E-06
ATF3	1662	313.5	4916.5	5.473438	87.7148	4.002301	Up	0	0
C10orf10	2060	255	4006.5	3.599604	57.60043	4.00017	Up	0	0
C7orf68	1432	293	4449.5	5.960833	91.99592	3.947984	Up	5.51E-13	1.90E-12
PDGFB	3393	14	208.5	0.119271	1.822881	3.933903	Up	1.40E-07	3.60E-07
FAM115C	2983	230.5	3370	2.253787	33.44117	3.891202	Up	3.86E-13	1.35E-12
C12orf68	2380	4	58	0.049822	0.722288	3.857729	Up	4.59E-05	0.000104
LANCL3	3259	4	56.5	0.03568	0.512407	3.844109	Up	4.59E-05	0.000104
ZP1	1972	46	659.5	0.696727	9.878193	3.825582	Up	7.84E-14	2.85E-13
GFAP	2193	13.5	182	0.179216	2.515241	3.810925	Up	2.60E-07	6.61E-07
AKAP12	8246	750.5	10061	2.651978	36.18043	3.770069	Up	0	0

FAM153B	2096	1	14	0.014417	0.195919	3.764438	Up	0.001094	0.002244
SERPINE1	3153	29647.5	382313.5	273.1052	3589.234	3.716148	Up	1.90E-10	5.47E-10
GDF1	2558	3	38.5	0.034542	0.443149	3.681376	Up	7.36E-05	0.000164
VLDLR	3562	458.5	5754.5	3.754951	47.8275	3.670974	Up	1.74E-12	5.78E-12
BNIP3L	3505	2394.5	29248	19.82032	247.4938	3.642341	Up	0	0
LINGO1	2932	10	123	0.103061	1.214319	3.558572	Up	1.61E-06	3.94E-06
CAMK2B	3935	10	114.5	0.073876	0.861058	3.542935	Up	1.61E-06	3.94E-06
EFCAB3	1694	30	352.5	0.529044	6.151593	3.5395	Up	5.60E-12	1.78E-11
LINGO3	2130	2	21.5	0.026218	0.300346	3.517969	Up	0.000151	0.000331
ANO9	2879	1	11.5	0.010496	0.119757	3.512222	Up	0.003992	0.007806
SH2D2A	1630	5	58	0.092692	1.056385	3.510546	Up	2.76E-05	6.33E-05
EPHA3	5837	141.5	1581.5	0.713464	8.024479	3.491496	Up	0	0
WFDC3	1000	14	150.5	0.409277	4.458834	3.445517	Up	1.40E-07	3.60E-07
PTGES	1787	6	65	0.09889	1.074067	3.44112	Up	1.62E-05	3.75E-05
INSIG2	2592	443	4687.5	4.960425	53.63997	3.434773	Up	0	0
SPINK1	454	4	43	0.26118	2.805613	3.425202	Up	4.59E-05	0.000104
NPAS1	2066	42.5	447.5	0.598284	6.421017	3.423899	Up	1.10E-13	3.98E-13
ESM1	1939	1	11	0.015584	0.167034	3.421991	Up	0.003992	0.007807
LRRC4B	2958	1	11	0.010216	0.107557	3.396256	Up	0.003992	0.007806
RASSF2	5426	28	284	0.151915	1.549196	3.350183	Up	1.93E-11	5.89E-11
RTN4RL1	3183	2	20	0.018266	0.185781	3.346383	Up	0.000181	0.000395
ESPN	3531	2	20	0.016466	0.167472	3.346383	Up	0.000181	0.000395
BAI1	5535	4	40	0.021008	0.213415	3.344636	Up	4.59E-05	0.000104
SPAG4	1452	67	666	1.348504	13.57193	3.331195	Up	0	0
MXI1	3272	234.5	2278.5	2.087102	20.64487	3.30621	Up	4.49E-13	1.56E-12

DOK3	2354	108	1046.5	1.32982	13.14934	3.305687	Up	0	0
CAMK2N1	2371	32.5	308	0.396295	3.844118	3.278004	Up	1.74E-12	5.78E-12
BNIP3	1535	3013	28298	56.97037	546.0767	3.26082	Up	0	0
GDF6	3716	2	18.5	0.015646	0.14823	3.243973	Up	0.000337	0.000719
SIGLEC14	2113	6	56	0.083633	0.784148	3.228987	Up	1.62E-05	3.75E-05
HHIP	3555	91.5	841.5	0.750317	7.017655	3.225417	Up	0	0
ARC	2985	11	100.5	0.109048	0.994409	3.188873	Up	8.84E-07	2.19E-06
IGFBP3	2620	1305	11463.5	14.48266	129.5305	3.160893	Up	7.23E-12	2.27E-11
OTP	2702	2	17.5	0.021518	0.192358	3.16021	Up	0.000518	0.001092
GRIN1	4100	3	27	0.021551	0.191166	3.149022	Up	7.82E-05	0.000174
CYP1A1	2608	7	60	0.076706	0.679127	3.146275	Up	9.28E-06	2.18E-05
LOX	5177	9774	83803	54.72542	479.6815	3.131794	Up	0	0
FAM162A	838	691.5	5919.5	23.93947	209.5574	3.129882	Up	1.59E-12	5.30E-12
ERO1L	3334	2179	18147	18.94464	161.4672	3.09138	Up	0	0
ITGAM	4745	17	146	0.107778	0.909546	3.077091	Up	2.14E-08	5.69E-08
ALOXE3	3415	9	75	0.078292	0.651552	3.056935	Up	2.92E-06	7.05E-06
SIK1	4706	1045	8168.5	6.44572	51.39031	2.995083	Up	0	0
TGFA	4261	43	336.5	0.295247	2.345081	2.989642	Up	1.18E-13	4.25E-13
AFAP1L1	4223	230	1789.5	1.583267	12.5573	2.987551	Up	3.86E-13	1.35E-12
HTR2A	3564	1.5	12	0.012396	0.097384	2.973817	Up	0.002405	0.0048
SLC6A8	3129	1470.5	11242	13.66305	106.367	2.9607	Up	2.06E-12	6.83E-12
NRN1	2072	1537	11663	21.50097	166.9261	2.956735	Up	4.01E-12	1.29E-11
PVRL4	3520	71.5	548.5	0.596192	4.624008	2.955295	Up	0	0
ANKRD1	1994	148	1118.5	2.159966	16.63375	2.945033	Up	0	0
IL8	1718	2538	18973	42.74962	327.7224	2.938491	Up	0	0

NDNF	2911	78.5	588.5	0.784122	5.982909	2.931696	Up	0	0
KIAA1244	14895	285	2143.5	0.562004	4.259676	2.922091	Up	6.22E-13	2.14E-12
NMU	818	9	66.5	0.321245	2.419565	2.913	Up	2.92E-06	7.05E-06
DUOXA2	1451	3	23	0.062476	0.468327	2.906141	Up	0.000123	0.00027
ZSWIM5	5860	187	1360	0.927861	6.873353	2.889033	Up	3.37E-13	1.18E-12
ANO7	916	18.5	135	0.5915	4.361116	2.882248	Up	1.14E-08	3.05E-08
MT1X	468	862.5	6209.5	53.42891	393.0851	2.879149	Up	0	0
LIMS2	2173	2	15	0.027812	0.202946	2.867323	Up	0.001428	0.002907
DPYSL4	2732	1373	9813	14.58772	106.3424	2.865891	Up	5.26E-12	1.68E-11
STC1	3897	3733	26429	27.78465	200.9926	2.854783	Up	0	0
PFKFB4	3503	1167.5	8257	9.672453	69.73147	2.849856	Up	3.45E-12	1.12E-11
HOXD1	1991	6	41.5	0.086452	0.618682	2.839226	Up	1.63E-05	3.77E-05
LOC646626	748	3	22	0.121194	0.865985	2.837028	Up	0.000162	0.000353
BOK	2617	2	14	0.022216	0.158556	2.835298	Up	0.002465	0.004915
EFNA3	1782	92.5	638.5	1.506075	10.59661	2.814737	Up	6.66E-15	2.50E-14
GPR155	7324	376.5	2596.5	1.496348	10.50568	2.811652	Up	4.39E-13	1.53E-12
LCN15	762	2	13.5	0.074794	0.523686	2.807712	Up	0.004296	0.008375
TTC29	1798	2	14	0.032336	0.226003	2.805122	Up	0.002465	0.004915
C2orf82	480	2	13	0.116345	0.810171	2.799822	Up	0.004296	0.008374
ANG	1222	68.5	464	1.647857	11.24753	2.770946	Up	0	0
KIF1A	8843	8	55	0.027077	0.184584	2.769113	Up	5.24E-06	1.25E-05
DUSP1	2040	2609	17454	37.19363	253.5255	2.769004	Up	0	0
TFF2	717	2	14	0.084289	0.570733	2.7594	Up	0.002465	0.004914
ZMAT4	2482	30	196	0.349522	2.339084	2.742486	Up	5.60E-12	1.78E-11
INHBA	2175	139	940	1.910045	12.77843	2.742032	Up	1.77E-13	6.32E-13

PRODH	2423	3	20	0.036466	0.243463	2.739066	Up	0.000351	0.000748
SLC16A3	2031	2289	14999.5	32.77822	218.3256	2.735672	Up	0	0
ISG20	974	18.5	121	0.559811	3.693038	2.721795	Up	1.14E-08	3.05E-08
HSPC159	3842	8	52	0.061129	0.401148	2.714216	Up	5.24E-06	1.25E-05
NXPH4	1821	997.5	6435.5	15.95512	104.6056	2.712869	Up	0	0
FAM183A	530	2	13.5	0.114029	0.74752	2.712716	Up	0.004296	0.008373
GALNTL4	2531	18	118	0.211275	1.377287	2.704635	Up	1.14E-08	3.05E-08
B3GNT4	1554	7.5	48	0.140669	0.91456	2.700771	Up	9.28E-06	2.18E-05
ALDOC	1665	1045.5	6674	18.28118	118.6974	2.698857	Up	0	0
OSCAR	1385	8.5	54.5	0.179652	1.161895	2.693206	Up	5.24E-06	1.25E-05
PPP2R2C	4161	9.5	62.5	0.068714	0.443552	2.690422	Up	2.92E-06	7.05E-06
C21orf7	1997	100.5	634.5	1.460958	9.416823	2.688325	Up	1.75E-14	6.52E-14
SNTA1	2345	290.5	1836.5	3.615173	23.16791	2.679991	Up	2.80E-13	9.85E-13
3-Mar	4158	33	209.5	0.234303	1.498246	2.676827	Up	1.01E-12	3.43E-12
PDK1	4576	697	4353.5	4.416327	28.22126	2.675864	Up	0	0
APLN	3238	39	242	0.348363	2.217553	2.670306	Up	1.32E-13	4.75E-13
ARHGDIG	942	2	13	0.064156	0.408267	2.66985	Up	0.004296	0.008375
FAM43B	2640	10	61.5	0.109245	0.69248	2.664207	Up	1.61E-06	3.94E-06
PPP1R3C	2646	347	2085	3.797988	23.36503	2.621044	Up	1.58E-13	5.65E-13
ANGPTL4	1967	8308.5	50132	122.8324	754.1229	2.618108	Up	0	0
HOXA13	2514	128.5	770	1.48018	9.075035	2.616131	Up	3.69E-14	1.36E-13
IL2RG	1560	66	406	1.257842	7.70999	2.615779	Up	0	0
KCNT1	4782	4	25	0.025276	0.1545	2.611762	Up	0.000105	0.000233
CAMK2A	4885	4	25	0.024743	0.15095	2.608964	Up	0.000105	0.000233
PRR5L	3378	10	61	0.088095	0.535004	2.602412	Up	1.61E-06	3.94E-06

GPR35	2293	3	18	0.038534	0.233912	2.601769	Up	0.000929	0.001919
C15orf48	929	20	118	0.623367	3.763116	2.593774	Up	3.18E-09	8.74E-09
VEGFA	3494	3466	20116	28.74524	170.5386	2.568704	Up	0	0
MAP6D1	2154	60.5	356.5	0.82316	4.879962	2.567625	Up	0	0
TMEM145	1779	23.5	136.5	0.38433	2.276748	2.566559	Up	4.68E-10	1.33E-09
ZNF395	4807	2045	11882	12.36611	73.23642	2.56617	Up	0	0
SYTL2	4060	354	2073.5	2.558428	15.13699	2.564749	Up	0	0
CELF4	4067	3.5	21	0.025441	0.149696	2.556833	Up	0.00023	0.000497
SORBS2	5529	120	695.5	0.635497	3.719033	2.548971	Up	0	0
FAM83E	2011	5	29	0.072848	0.425631	2.546632	Up	5.00E-05	0.000113
LBH	2956	33	188	0.323367	1.885005	2.543324	Up	1.01E-12	3.43E-12
MUC1	1161	132.5	749.5	3.291462	19.15865	2.541195	Up	1.14E-13	4.12E-13
ANKRD37	934	113.5	645.5	3.533229	20.50478	2.536901	Up	2.08E-13	7.38E-13
RGMA	3058	3	17.5	0.028894	0.167624	2.536383	Up	0.001563	0.003169
POU3F1	2928	5	28	0.04925	0.284164	2.528533	Up	6.62E-05	0.000149
RNF122	1868	114	645	1.77286	10.21821	2.526992	Up	7.79E-14	2.83E-13
TFR2	2471	9	52	0.109131	0.625458	2.518848	Up	2.93E-06	7.07E-06
KCTD11	3081	601	3353	5.644544	32.2137	2.512746	Up	1.08E-13	3.92E-13
DHRS13	1939	107	593	1.589386	9.044771	2.508614	Up	0	0
TMEM158	1876	887	4910	13.70259	77.60263	2.501658	Up	1.58E-12	5.27E-12
ARVCF	4056	80.5	446.5	0.576252	3.262653	2.501273	Up	0	0
GADD45B	1393	567.5	3143	11.81541	66.86604	2.500604	Up	0	0
SEMA7A	3393	385	2103	3.297206	18.32782	2.47472	Up	6.91E-13	2.37E-12
RNASE4	1805	143.5	779.5	2.315885	12.79509	2.465954	Up	0	0
MAFF	2234	435.5	2351	5.653377	31.17941	2.463409	Up	1.31E-12	4.39E-12

GOLGA7B	2894	4	22	0.040973	0.225806	2.462343	Up	0.000357	0.00076
PLOD2	4072	16023.5	86139.5	114.0873	627.467	2.459401	Up	0	0
PPP1R3B	5574	437	2336.5	2.272298	12.41587	2.449962	Up	8.15E-13	2.78E-12
LOC100130503	3038	3	16.5	0.02984	0.16227	2.443091	Up	0.00265	0.005267
C3orf58	3416	518.5	2747.5	4.395135	23.84363	2.439625	Up	0	0
GYS1	3635	3388	17923.5	27.05126	145.9496	2.431702	Up	0	0
HK2	7109	261.5	1378.5	1.072636	5.744243	2.420957	Up	2.23E-13	7.90E-13
LOC554223	1301	10	53	0.225209	1.204667	2.419301	Up	1.63E-06	3.99E-06
POU3F2	4172	10	52.5	0.069679	0.372027	2.416606	Up	1.65E-06	4.02E-06
PRSS53	2028	130	677	1.8663	9.861709	2.401657	Up	0	0
SLAMF9	1170	40	214.5	1.03308	5.413267	2.389547	Up	1.16E-13	4.18E-13
FOSB	3776	62	314.5	0.472759	2.467351	2.383786	Up	0	0
4-Mar	4466	52	266.5	0.343618	1.765667	2.361337	Up	5.13E-14	1.88E-13
RAP1GAP	3301	13	65	0.113441	0.580797	2.356088	Up	2.61E-07	6.63E-07
RORA	10974	32	167	0.088114	0.447839	2.345538	Up	1.74E-12	5.78E-12
CA12	3959	459.5	2272.5	3.361989	16.99325	2.337575	Up	1.20E-12	4.03E-12
ICAM5	3005	176.5	877.5	1.709548	8.634767	2.336542	Up	0	0
IGFBP1	1660	16	81	0.287107	1.447519	2.333924	Up	4.02E-08	1.06E-07
GOLGA6D	3449	5	24.5	0.04181	0.208982	2.321454	Up	0.000353	0.000751
KCNK13	2517	15	74.5	0.175522	0.875988	2.319257	Up	7.52E-08	1.96E-07
RCOR2	2621	26	127.5	0.290124	1.442747	2.314079	Up	6.87E-11	2.02E-10
FABP6	672	8	39	0.346073	1.719038	2.312452	Up	8.54E-06	2.01E-05
HOMER1	4228	365	1764.5	2.512046	12.37279	2.300236	Up	6.76E-13	2.32E-12
IRS2	7014	1111.5	5309.5	4.600249	22.44552	2.286643	Up	5.50E-12	1.75E-11
FST	2098	2421	11553.5	33.46364	163.2574	2.286482	Up	0	0

PTGIR	2103	4	19	0.055293	0.269479	2.28501	Up	0.00159	0.003221
SUSD2	3201	43.5	208.5	0.397379	1.931996	2.281505	Up	1.18E-13	4.25E-13
PFKFB3	4553	2497.5	11827	15.91122	76.951	2.273896	Up	0	0
PEAR1	4866	32.5	156.5	0.1964	0.949634	2.273579	Up	1.74E-12	5.79E-12
SCN3B	6081	6.5	31	0.031168	0.150505	2.271696	Up	4.05E-05	9.21E-05
CLDN14	1768	58.5	275	0.958959	4.60078	2.262337	Up	1.35E-14	5.05E-14
SLC2A3	3938	745.5	3490.5	5.489701	26.27493	2.258888	Up	3.42E-13	1.20E-12
PGK1	2439	21130.5	98791	251.2641	1200.088	2.255864	Up	0	0
CACNB4	7979	106.5	499.5	0.389381	1.856199	2.253097	Up	0	0
NEBL	6956	381.5	1769.5	1.596902	7.552442	2.241668	Up	3.02E-13	1.06E-12
POU2F2	3669	9	43.5	0.074123	0.349546	2.237482	Up	4.14E-06	9.93E-06
PLA2G4D	3584	10	48	0.084313	0.396947	2.235127	Up	1.88E-06	4.58E-06
PTGS1	4982	84	392	0.494289	2.325748	2.234268	Up	0	0
PTPRF	7733	6000	27479.5	22.56786	105.2114	2.220949	Up	1.43E-11	4.40E-11
STC2	5361	6492.5	29586.5	35.12708	163.4478	2.218174	Up	2.56E-11	7.70E-11
PRKAA2	9369	63	290	0.197069	0.916188	2.216946	Up	0	0
AANAT	1048	9	40.5	0.246364	1.140949	2.211374	Up	8.54E-06	2.01E-05
SLC2A1	3687	10740	48717.5	84.56376	391.1447	2.209591	Up	0	0
ANXA8L2	2053	71	323	1.010379	4.663078	2.206386	Up	0	0
WSB1	2849	1087	4794	11.04342	49.89426	2.175687	Up	1.55E-12	5.16E-12
MAP7D2	4158	96	427.5	0.675864	3.045461	2.171856	Up	0	0
COL13A1	2816	1133.5	4969.5	11.68282	52.27286	2.161674	Up	2.19E-12	7.24E-12
EGLN1	7102	2629	11497.5	10.75126	48.00531	2.158689	Up	0	0
ANKZF1	2594	466.5	2003.5	5.216198	22.87586	2.132755	Up	4.74E-13	1.65E-12
ARHGEF37	4865	184.5	795.5	1.104931	4.83938	2.130865	Up	3.15E-13	1.11E-12

CHSY1	4567	2056	8812.5	13.09276	57.18663	2.126909	Up	0	0
FAM83H	5604	9	38	0.046482	0.202253	2.121427	Up	1.81E-05	4.20E-05
WDR69	1669	19	82	0.335749	1.457043	2.117588	Up	6.12E-09	1.66E-08
PLAT	3173	362.5	1531.5	3.313712	14.27029	2.106495	Up	1.14E-12	3.85E-12
CTGF	2358	2413.5	10145	29.70011	127.4345	2.101216	Up	0	0
CRB2	5641	4	17	0.020613	0.088432	2.100988	Up	0.004389	0.008551
SLC1A1	3757	666.5	2792.5	5.148709	22.02383	2.096782	Up	1.73E-14	6.44E-14
PPP1R13L	3120	344	1438	3.205171	13.64572	2.089975	Up	5.51E-13	1.90E-12
LRRC4	3723	5	22	0.040582	0.172065	2.084032	Up	0.00094	0.001941
PPP2R5B	2766	722.5	2982.5	7.562022	31.92247	2.077729	Up	8.86E-14	3.21E-13
ITPR1	10197	340.5	1406.5	0.967846	4.082355	2.076552	Up	1.85E-13	6.59E-13
GREM2	4199	13	54	0.090274	0.380478	2.075434	Up	5.35E-07	1.34E-06
ADM2	4246	6	24	0.039998	0.168203	2.072211	Up	0.000894	0.00185
GLUL	4083	5	21	0.036442	0.152966	2.069532	Up	0.001541	0.003129
RIMKLA	10583	38	162	0.107851	0.451262	2.064928	Up	1.45E-13	5.21E-13
WNT5B	2184	293	1202.5	3.898403	16.31024	2.064823	Up	5.51E-13	1.90E-12
NOS3	2040	13.5	56	0.194345	0.811506	2.061984	Up	3.63E-07	9.15E-07
LOC653125	2000	14	56.5	0.202917	0.839619	2.048843	Up	4.07E-07	1.02E-06
S1PR1	3050	21	86	0.202789	0.837122	2.045461	Up	1.78E-09	4.94E-09
RIOK3	4035	2309.5	9282	16.56101	68.21851	2.042372	Up	0	0
CSPG5	2334	35	142	0.436419	1.794011	2.039402	Up	3.77E-13	1.32E-12
ALS2CL	4938	13	53	0.077693	0.31797	2.033029	Up	7.07E-07	1.76E-06
RNF144B	5036	18.5	75	0.107816	0.440692	2.031199	Up	1.25E-08	3.36E-08
GPT2	3963	1782	7115	13.07659	53.16057	2.02337	Up	0	0
METTL21B	2717	253	1008.5	2.704826	10.97855	2.02108	Up	3.97E-13	1.39E-12

BRSK2	3506	40	159	0.331662	1.341485	2.016047	Up	1.16E-13	4.18E-13
C4orf3	2975	3020.5	11897	29.44205	118.6543	2.010815	Up	0	0
CSTA	838	6	24	0.213617	0.855674	2.002037	Up	0.000894	0.00185
SLC29A4	2910	338	1323.5	3.383236	13.42823	1.988793	Up	1.46E-13	5.22E-13
FAM78B	1481	5.5	22.5	0.112219	0.441826	1.977158	Up	0.00094	0.001941
MME	5710	1357	5221	6.892941	27.11199	1.97574	Up	4.05E-12	1.31E-11
ACHE	2978	25	97.5	0.245197	0.964245	1.975459	Up	1.51E-10	4.37E-10
BLNK	1760	5	20	0.085845	0.33599	1.968604	Up	0.002522	0.005021
TMEM45A	1564	1644.5	6286	30.50148	119.2405	1.966923	Up	5.77E-12	1.83E-11
LOC100288336	2001	6	23	0.088314	0.341032	1.949199	Up	0.001447	0.002943
HSPA6	2664	18	69	0.199004	0.764176	1.941106	Up	3.21E-08	8.49E-08
KIAA1199	7080	2192	8214.5	8.99474	34.355	1.933366	Up	0	0
MYO1E	4729	1268.5	4733	7.767526	29.66316	1.933145	Up	2.55E-12	8.39E-12
KRT17	1574	14	51.5	0.256378	0.970436	1.920358	Up	2.96E-06	7.14E-06
PDK3	12795	1071.5	3963	2.436458	9.187164	1.914834	Up	1.09E-12	3.70E-12
CTAGE6P	2675	7	27	0.079074	0.296749	1.907965	Up	0.000518	0.001091
C7orf63	3855	39	145	0.296774	1.112772	1.906723	Up	1.33E-13	4.78E-13
CHST5	3265	8.5	31.5	0.076207	0.284742	1.90165	Up	0.000187	0.000407
SYTL3	2284	42	154	0.53356	1.988064	1.897642	Up	1.10E-13	3.98E-13
P4HA2	2588	5963.5	21717	66.85989	248.6624	1.894975	Up	3.03E-11	9.07E-11
FOXC2	1683	47	168.5	0.797505	2.962076	1.893043	Up	0	0
LOC100652961	981	6	22	0.180138	0.668328	1.891451	Up	0.002332	0.004657
SYNGR3	2076	199.5	726	2.789998	10.33843	1.889681	Up	3.57E-14	1.32E-13
RSPO2	3149	18	68	0.172727	0.638659	1.886553	Up	4.46E-08	1.17E-07
FAM116B	2029	38.5	139.5	0.550188	2.032989	1.885605	Up	1.52E-13	5.43E-13

TMEM191C	1325	24.5	88.5	0.536225	1.97721	1.882557	Up	8.15E-10	2.30E-09
P4HA1	2860	10116.5	36410	102.4575	377.5509	1.881645	Up	1.48E-11	4.55E-11
IZUMO4	966	26	94	0.784802	2.876687	1.874007	Up	2.68E-10	7.68E-10
MBP	2189	36.5	136	0.492325	1.802833	1.872584	Up	2.61E-13	9.21E-13
TIMP3	5496	4698.5	16762	24.79439	90.38589	1.866084	Up	7.09E-12	2.23E-11
RGS7BP	3821	5	19	0.039541	0.14382	1.862824	Up	0.004105	0.008016
SHANK3	7145	52.5	187.5	0.213361	0.775829	1.862445	Up	5.13E-14	1.88E-13
NIM1	2326	74	272	0.951485	3.457917	1.861651	Up	9.99E-15	3.74E-14
ADAM8	3149	57	201.5	0.521461	1.892787	1.85988	Up	5.37E-14	1.96E-13
PKIA	4215	349	1230	2.387666	8.656826	1.858237	Up	2.05E-13	7.29E-13
WDR54	1223	270	952.5	6.384939	23.08592	1.854268	Up	4.53E-13	1.58E-12
GBE1	3118	3063.5	10792	28.45328	102.6302	1.850789	Up	0	0
UNC5A	3749	6	22	0.047749	0.172018	1.849015	Up	0.002332	0.004658
FAM84A	6366	112.5	395	0.513097	1.845217	1.846487	Up	0	0
FBXL16	3543	18	63.5	0.147689	0.530901	1.84588	Up	3.43E-07	8.64E-07
KDM3A	4907	1015	3560.5	5.993441	21.48887	1.842133	Up	1.25E-12	4.22E-12
EPB49	2561	26	93.5	0.300505	1.074398	1.838068	Up	3.83E-10	1.09E-09
EGR3	3912	19	68	0.143243	0.511167	1.835334	Up	8.44E-08	2.19E-07
KCNMB4	1631	7	25	0.126875	0.451231	1.830455	Up	0.001328	0.002711
CKB	1431	24	84	0.490757	1.744799	1.82998	Up	3.75E-09	1.03E-08
AQP4	5136	7	25	0.041184	0.145524	1.821082	Up	0.001328	0.002711
ROPN1L	974	26	90.5	0.776	2.739573	1.819823	Up	1.28E-09	3.58E-09
MEX3B	3398	57	197	0.486626	1.716837	1.818867	Up	5.37E-14	1.96E-13
PPP1R15A	2399	2559.5	8835.5	30.99948	109.2046	1.816718	Up	0	0
FN1	8815	198474.5	679284.5	651.1983	2281.398	1.80875	Up	7.48E-10	2.11E-09

ITGB3	4894	686.5	2358	4.080477	14.27582	1.806764	Up	0	0
GJA3	5219	117.5	400.5	0.651734	2.277936	1.805372	Up	1.22E-13	4.41E-13
ANXA8L1	1954	57	194	0.845067	2.944466	1.800868	Up	5.37E-14	1.96E-13
PCOLCE2	2047	11	39	0.16126	0.560139	1.796398	Up	5.79E-05	0.00013
FLNB	9434	5883	19949.5	18.03667	62.62274	1.795753	Up	1.29E-11	3.98E-11
BTG2	2718	727	2458.5	7.752351	26.82963	1.791121	Up	1.35E-12	4.52E-12
CHD5	9663	17	60	0.052449	0.181293	1.78934	Up	5.88E-07	1.47E-06
GADD45A	1296	2594.5	8770	58.10563	200.8427	1.789316	Up	0	0
SERTAD1	1213	404.5	1371	9.685058	33.43439	1.7875	Up	2.91E-13	1.02E-12
CSRP2	901	182.5	618	5.896516	20.35077	1.787148	Up	0	0
PSTPIP2	3054	48.5	162.5	0.457713	1.576457	1.784172	Up	1.06E-13	3.85E-13
VSIG1	3236	31	107	0.283094	0.974931	1.78402	Up	3.83E-11	1.14E-10
GNRH1	2166	18	60	0.238402	0.820356	1.782855	Up	1.28E-06	3.14E-06
TMEM47	4068	736	2476.5	5.250752	18.06769	1.782816	Up	1.22E-12	4.12E-12
PTPRR	2650	47	159	0.518615	1.775351	1.775369	Up	0	0
FUT11	2069	772.5	2583.5	10.82976	36.99912	1.772489	Up	2.51E-12	8.27E-12
ERRFI1	3144	6672.5	22222	61.5234	209.5265	1.767926	Up	1.54E-11	4.72E-11
CPA4	2817	1740.5	5808	18.00332	61.04586	1.76163	Up	4.74E-12	1.52E-11
HMGA2	4150	2625	8668.5	18.30812	61.90356	1.757539	Up	0	0
TLE6	1955	22	75	0.335349	1.132275	1.755488	Up	3.99E-08	1.05E-07
CDC42BPG	4945	8.5	29	0.051477	0.173672	1.754357	Up	0.000477	0.001009
WDR52	3281	227.5	759.5	2.032646	6.855766	1.753959	Up	3.55E-15	1.35E-14
MFAP2	1121	8	26	0.203364	0.684873	1.751771	Up	0.001883	0.003792
SYT8	1423	19.5	64	0.395539	1.332006	1.751709	Up	4.81E-07	1.20E-06
RRAD	1476	577.5	1900	11.34095	38.07666	1.747365	Up	5.81E-13	2.00E-12

HYAL3	1855	213	701	3.342304	11.17261	1.741051	Up	2.80E-13	9.85E-13
FOXH1	2193	57	187.5	0.755062	2.521522	1.739628	Up	5.37E-14	1.96E-13
NR1D1	2788	421.5	1381	4.394726	14.67447	1.739464	Up	4.13E-13	1.44E-12
ADAMTS6	7289	349	1138.5	1.394408	4.630425	1.731492	Up	2.05E-13	7.28E-13
DDX41	2118	3111.5	10085	42.69132	140.9543	1.723213	Up	0	0
TPD52	4250	138	440.5	0.933665	3.078448	1.721226	Up	1.83E-13	6.53E-13
MEGF6	7450	59	190.5	0.230066	0.757731	1.719639	Up	2.53E-14	9.36E-14
LOC401097	2606	40	129.5	0.448845	1.474072	1.715517	Up	2.75E-12	9.00E-12
SRD5A3	4089	419.5	1352	2.984198	9.793531	1.714486	Up	0	0
ACPL2	3024	178.5	581	1.732832	5.68453	1.713909	Up	1.14E-13	4.11E-13
ATP2B2	8962	26	88	0.087153	0.28464	1.707511	Up	3.02E-09	8.30E-09
TNFAIP3	4446	558	1785	3.646674	11.89368	1.705542	Up	1.47E-13	5.27E-13
RUNX1	7274	1432.5	4574	5.720887	18.63617	1.703795	Up	2.95E-12	9.65E-12
GCNT1	5606	429	1366	2.220709	7.223136	1.701605	Up	0	0
NEK11	2793	92	297	0.969881	3.152723	1.700719	Up	6.66E-15	2.50E-14
KIAA1715	5765	2372.5	7507.5	11.91552	38.62849	1.696823	Up	0	0
ZNF470	7149	276	878.5	1.122626	3.635974	1.695464	Up	7.04E-14	2.56E-13
MLANA	1524	9.5	30.5	0.180835	0.584474	1.692464	Up	0.000682	0.001425
SLC25A4	4420	244	774.5	1.606076	5.186271	1.691158	Up	2.85E-13	1.00E-12
EDN1	2112	192	612	2.654694	8.572218	1.691123	Up	0	0
PRPH	1833	8	25	0.125623	0.404628	1.6875	Up	0.00294	0.005823
VKORC1	1042	3305.5	10424	92.26282	295.945	1.681508	Up	0	0
ARID3A	2823	235	742	2.427255	7.783417	1.681078	Up	3.29E-13	1.16E-12
KLF5	3350	114	360.5	0.994048	3.186379	1.68053	Up	7.79E-14	2.83E-13
FANK1	1395	63	199.5	1.325184	4.232239	1.675229	Up	0	0

PRR5	1878	19	60.5	0.299606	0.956529	1.674743	Up	2.74E-06	6.62E-06
C17orf103	4787	457	1428	2.774997	8.836523	1.670993	Up	1.73E-12	5.76E-12
DUOX2	6428	24.5	79	0.113566	0.361475	1.670362	Up	3.25E-08	8.57E-08
ABTB2	4919	603	1876	3.562657	11.29701	1.664915	Up	7.03E-13	2.41E-12
SGK1	2638	303.5	943	3.341238	10.58728	1.663878	Up	2.56E-13	9.05E-13
LDHA	2102	42700	131974.5	589.0682	1861.316	1.659816	Up	1.02E-10	2.98E-10
AMPD3	4371	465	1437.5	3.085219	9.748241	1.659769	Up	0	0
GLTP	2371	1489	4604.5	18.23781	57.58438	1.658745	Up	5.61E-12	1.78E-11
FOXL1	3207	159	491	1.445206	4.539547	1.651273	Up	8.53E-14	3.09E-13
C17orf76	2669	38.5	119	0.420408	1.319593	1.650231	Up	4.21E-11	1.25E-10
BCL11A	5946	9	29	0.044966	0.141064	1.649436	Up	0.001068	0.002195
IL21R	4865	56.5	175	0.339613	1.064816	1.648641	Up	1.69E-14	6.27E-14
PLGLB1	3011	8.5	26.5	0.083398	0.261209	1.647112	Up	0.001883	0.003792
VGLL3	10396	265.5	813.5	0.742799	2.321985	1.644314	Up	6.59E-13	2.26E-12
UNC5B	4998	639	1961	3.716882	11.61047	1.643262	Up	7.17E-13	2.46E-12
ACER2	2852	125.5	384	1.27418	3.980083	1.64323	Up	3.11E-15	1.18E-14
CACNA1B	9790	71	228.5	0.219147	0.683985	1.642065	Up	0	0
TMEM236	5464	9	28	0.048513	0.151358	1.641529	Up	0.001661	0.00336
EFNB2	4404	1862	5681	12.26343	38.25576	1.641314	Up	1.92E-13	6.84E-13
CYFIP2	6791	416	1275	1.785835	5.563145	1.639302	Up	0	0
ZNF175	3788	778.5	2377	5.972454	18.60286	1.639129	Up	0	0
ZDHHC9	4619	2358	7200	14.8269	46.15818	1.638369	Up	0	0
TEX9	1470	14	42.5	0.276859	0.86104	1.636931	Up	0.000154	0.000337
ADARB1	7044	389.5	1187.5	1.610456	4.99115	1.631903	Up	0	0
ABL2	12244	2997	9068.5	7.097593	21.97334	1.630352	Up	0	0

DUSP5	2545	446.5	1349	5.092687	15.68719	1.623088	Up	0	0
SNX33	3250	986	2975	8.809904	27.09689	1.620929	Up	0	0
JHDM1D	9178	362	1087	1.144214	3.517523	1.620203	Up	1.14E-12	3.85E-12
VGF	2586	9	27	0.100729	0.308069	1.612781	Up	0.002566	0.005104
MRAP	656	12	36.5	0.538768	1.645285	1.610603	Up	0.00047	0.000995
PPM1D	4790	766.5	2287	4.658179	14.17395	1.605404	Up	1.15E-12	3.89E-12
CPM	6683	54	163	0.237468	0.720432	1.601127	Up	8.02E-14	2.91E-13
ETS2	3672	925.5	2741.5	7.31707	22.09782	1.594566	Up	8.96E-13	3.05E-12
PPP1R16A	2326	1063	3149	13.26506	40.03087	1.593482	Up	2.25E-12	7.45E-12
PTPN14	13443	8842	26070	19.07007	57.49401	1.592101	Up	0	0
RGS4	3055	796	2345.5	7.553758	22.71469	1.588359	Up	3.24E-13	1.14E-12
ZBTB16	2262	7.5	23	0.099176	0.297885	1.586688	Up	0.003336	0.006581
MAP2K1	2603	2729.5	8030	30.44036	91.40635	1.586309	Up	0	0
GCKR	2197	58	171	0.770575	2.307207	1.58214	Up	4.00E-14	1.47E-13
SPOCK1	4841	189	565	1.151772	3.447346	1.581631	Up	2.87E-13	1.01E-12
BIRC3	5243	1910.5	5583.5	10.56827	31.60266	1.580307	Up	4.45E-12	1.43E-11
IL11	2354	48	140	0.586915	1.753891	1.579336	Up	8.37E-12	2.62E-11
DDB2	1870	1436.5	4190.5	22.23943	66.37265	1.577469	Up	2.22E-12	7.35E-12
STBD1	2468	500.5	1462.5	5.888563	17.56678	1.576863	Up	5.05E-13	1.75E-12
SLC35E1	5109	3894	11349.5	22.10673	65.76174	1.572763	Up	1.33E-11	4.09E-11
MN1	7569	625	1823.5	2.399822	7.136727	1.572335	Up	0	0
JAG2	4963	83	241.5	0.485469	1.438255	1.56687	Up	1.43E-13	5.12E-13
OSMR	5556	8073	23395.5	42.15474	124.7666	1.565466	Up	0	0
VSTM2L	2018	287.5	829.5	4.120806	12.17538	1.562968	Up	4.35E-13	1.51E-12
SYT7	4609	11	32.5	0.070376	0.20752	1.560099	Up	0.001261	0.002579

SLC2A5	2454	27	79	0.322181	0.949181	1.558813	Up	2.88E-07	7.28E-07
ECSCR	1027	8.5	24.5	0.240041	0.705315	1.554984	Up	0.004557	0.008857
ZCCHC5	2648	48	142	0.539085	1.578033	1.549544	Up	3.77E-12	1.22E-11
MAMDC4	3669	281	806	2.223599	6.499666	1.547469	Up	0	0
NR1I2	4446	9	27	0.061169	0.17806	1.541487	Up	0.002566	0.005104
SLC30A3	2108	29	83.5	0.40482	1.173509	1.535475	Up	2.20E-07	5.60E-07
EFCAB4A	1838	176	497	2.772412	8.00114	1.529064	Up	0	0
CAMK1D	2242	167	474	2.174051	6.270634	1.528226	Up	1.94E-13	6.91E-13
LDLRAD3	2969	40	114	0.393968	1.134173	1.52549	Up	1.33E-09	3.71E-09
IGFBP2	1439	12.5	36.5	0.259298	0.745066	1.522755	Up	0.00047	0.000995
PLXNA2	11457	838.5	2380.5	2.138816	6.145336	1.52268	Up	0	0
ANXA8	2070	118	333.5	1.658248	4.763194	1.52227	Up	0	0
PTPRU	5614	616.5	1741	3.199178	9.181663	1.521054	Up	5.19E-13	1.80E-12
KCP	2774	16	46	0.171809	0.492001	1.51786	Up	0.000117	0.000258
GPER	2981	30.5	86	0.298393	0.851216	1.512314	Up	1.28E-07	3.30E-07
HOXB7	1377	9.5	26.5	0.20014	0.570649	1.511594	Up	0.003936	0.007702
CSRNP1	3188	555.5	1553	5.07313	14.41213	1.506335	Up	0	0
MPI	1771	473	1311	7.756951	21.93791	1.499864	Up	5.88E-13	2.03E-12
PTX3	1955	702	1936	10.4109	29.39238	1.497347	Up	0	0
KIAA1467	4725	361.5	1002	2.224219	6.274075	1.496104	Up	0	0
GDNF	3830	16	46	0.126235	0.355974	1.495655	Up	0.000117	0.000258
SH2D4A	3266	572.5	1581.5	5.088179	14.33632	1.494453	Up	0	0
JAKMIP1	3011	11	32	0.110393	0.310712	1.492929	Up	0.001261	0.002579
AP3B2	3746	10	28	0.078216	0.22001	1.492037	Up	0.003379	0.006663
TNFAIP8L3	2292	27	73	0.33494	0.941179	1.490567	Up	3.18E-06	7.66E-06

KCTD16	5183	106	296.5	0.603826	1.691721	1.486287	Up	0	0
F3	2233	16417	45003.5	213.1773	597.066	1.485837	Up	0	0
FAM57A	2096	885	2433.5	12.28149	34.39629	1.485768	Up	0	0
FAM13A	5858	1375	3755.5	6.790881	18.99671	1.484079	Up	6.17E-12	1.95E-11
BEX2	1097	15	41.5	0.400633	1.120266	1.483488	Up	0.00046	0.000974
MRO	5006	10	28	0.058529	0.163633	1.483239	Up	0.003379	0.006664
ADAMTS1	4670	1434.5	3930.5	8.926016	24.91694	1.481039	Up	0	0
C1QL4	2065	17	46	0.23654	0.658846	1.477856	Up	0.000227	0.00049
OLFML2A	6556	240.5	659	1.068946	2.974327	1.476375	Up	0	0
SPRY3	9038	129.5	352.5	0.41583	1.155216	1.474096	Up	0	0
RAB20	1530	720.5	1958	13.66895	37.8816	1.470595	Up	0	0
IGF1R	11242	1274.5	3468	3.300109	9.145097	1.470485	Up	3.25E-12	1.06E-11
UAP1	2344	2823.5	7637	34.87076	96.59776	1.469972	Up	0	0
PIAS2	2360	458.5	1243.5	5.640203	15.60697	1.468372	Up	1.74E-12	5.78E-12
ITGB4	5925	38.5	106	0.190153	0.526017	1.467945	Up	8.60E-09	2.32E-08
IKBIP	1246	3624	9815	84.45589	233.5667	1.467564	Up	0	0
P2RY1	3122	52	141	0.484927	1.337437	1.46363	Up	7.60E-11	2.23E-10
DTNA	1763	86	237	1.441487	3.970931	1.461919	Up	0	0
PAM	5035	6740	18178	38.8458	106.9767	1.461466	Up	2.70E-11	8.12E-11
HBEGF	2381	684	1847	8.363636	23.03235	1.461459	Up	0	0
CPEB1	3202	16	44.5	0.148844	0.409559	1.460273	Up	0.000264	0.000568
ANKRD13A	3917	1374	3701	10.1802	28.00137	1.459732	Up	4.59E-12	1.47E-11
PODXL	5911	383	1034	1.896785	5.175559	1.448159	Up	1.49E-13	5.35E-13
KLF4	2949	495.5	1327	4.886596	13.31021	1.445632	Up	4.63E-13	1.61E-12
NAV2	7859	441.5	1197.5	1.654336	4.503434	1.444773	Up	3.59E-13	1.26E-12

PDZD7	2072	49.5	132.5	0.695869	1.892741	1.443589	Up	3.73E-10	1.06E-09
DDIT3	924	317	843	9.960783	27.04127	1.440832	Up	2.57E-13	9.08E-13
DNAJB2	3129	1287.5	3426	11.95187	32.44601	1.440804	Up	7.41E-12	2.33E-11
BOC	4293	314.5	844.5	2.143409	5.810469	1.438747	Up	1.32E-13	4.74E-13
GLT25D2	5182	21.5	59	0.123822	0.335105	1.436345	Up	1.69E-05	3.92E-05
GPRC5A	2856	1334	3547	13.59998	36.79089	1.435744	Up	1.66E-12	5.53E-12
INPP5D	4925	104	276	0.613867	1.658209	1.433628	Up	0	0
ZNF404	1868	58	154.5	0.905063	2.442759	1.432421	Up	2.15E-11	6.52E-11
CEACAM1	3333	69	184	0.607665	1.634072	1.427124	Up	1.57E-13	5.63E-13
CRYBG3	10748	1385.5	3647.5	3.741228	10.05845	1.426824	Up	7.00E-12	2.20E-11
C1orf88	2346	33	86	0.407448	1.090161	1.419854	Up	8.68E-07	2.15E-06
PRELID2	2339	206	537.5	2.549469	6.820584	1.419699	Up	2.46E-13	8.70E-13
LOC728047	1997	121	314	1.752193	4.663133	1.412138	Up	0	0
HOXA4	1728	278.5	728.5	4.701476	12.47723	1.408112	Up	1.88E-13	6.69E-13
CCDC80	4578	4049	10529	25.69197	68.15781	1.407562	Up	2.10E-11	6.38E-11
LOC100652910	1785	33.5	87	0.543324	1.439763	1.405946	Up	5.93E-07	1.48E-06
PLAC8	1403	332	857	6.854502	18.1482	1.404702	Up	0	0
FOS	2158	101.5	262.5	1.359584	3.59581	1.403152	Up	0	0
SERPING1	1832	34	92	0.560807	1.482155	1.40212	Up	1.61E-07	4.11E-07
THSD1	3366	64.5	167	0.55756	1.468999	1.397636	Up	5.90E-12	1.87E-11
MAGED4B	2588	168	432.5	1.879108	4.944708	1.395838	Up	1.69E-13	6.03E-13
SERTAD2	5565	1542.5	3956	8.042268	21.08654	1.390648	Up	3.47E-12	1.13E-11
TGFBR1	6244	853.5	2186.5	3.96803	10.38971	1.388661	Up	9.86E-13	3.35E-12
PRDM6	3027	68	175.5	0.656837	1.718043	1.38716	Up	3.22E-12	1.05E-11
SHROOM2	7445	28	74	0.112413	0.29325	1.383323	Up	4.03E-06	9.66E-06

PLXNB3	6377	2562	6569.5	11.6838	30.46217	1.38251	Up	0	0
YEATS2	6524	1914.5	4885.5	8.513631	22.17624	1.381169	Up	4.86E-12	1.56E-11
MYADM	3141	1906.5	4866	17.66322	45.84003	1.375859	Up	2.27E-12	7.51E-12
PLXNA3	6763	4227	10770.5	18.16327	47.07707	1.374	Up	1.88E-11	5.72E-11
ADAP1	2338	55	139	0.680422	1.757503	1.369026	Up	9.81E-10	2.75E-09
LOC653720	725	30.5	77	1.223743	3.160668	1.36893	Up	4.31E-06	1.03E-05
ZNF667	3877	37.5	95	0.281327	0.725328	1.366386	Up	3.19E-07	8.06E-07
B4GALT7	1747	1662	4199.5	27.6032	71.10785	1.365173	Up	6.43E-12	2.03E-11
MAGED4	2495	40	101.5	0.467894	1.205025	1.364809	Up	1.99E-07	5.08E-07
CCNG2	5489	878	2210.5	4.64619	11.94526	1.362318	Up	8.95E-13	3.05E-12
ATP6V0A4	3152	16.5	42	0.151994	0.39039	1.360906	Up	0.000584	0.001226
C19orf71	671	129.5	327	5.623235	14.42896	1.359495	Up	0	0
HOXB9	2711	115	290.5	1.239075	3.178209	1.358951	Up	0	0
CTHRC1	1236	115.5	289	2.710473	6.938191	1.356015	Up	0	0
ZNF432	2593	199	500.5	2.239399	5.721541	1.353292	Up	3.57E-14	1.32E-13
TNFRSF19	1625	531.5	1332	9.507116	24.28052	1.352719	Up	4.93E-14	1.81E-13
FOXN3	7907	162.5	407	0.59765	1.524065	1.350551	Up	0	0
C8orf58	1955	513	1283.5	7.623431	19.40078	1.347603	Up	0	0
FAM70B	1047	174	431	4.798264	12.20494	1.346881	Up	1.43E-13	5.11E-13
SMAD9	5481	789	1963.5	4.175909	10.61018	1.345288	Up	0	0
IPMK	6133	435.5	1082	2.062474	5.234072	1.343558	Up	1.31E-12	4.39E-12
CXCR7	2114	356	890	4.905759	12.44141	1.342601	Up	6.28E-13	2.16E-12
NRXN2	6413	25	66	0.117798	0.298556	1.341684	Up	1.38E-05	3.22E-05
HES2	4273	52	131	0.359138	0.906139	1.335194	Up	3.28E-09	8.98E-09
FZD3	13758	94.5	235	0.200801	0.50657	1.334995	Up	0	0

TNIK	5816	165	417	0.841096	2.119889	1.333647	Up	4.11E-14	1.51E-13
CDC42EP3	3981	4202.5	10343.5	30.63297	77.04434	1.330604	Up	1.88E-11	5.73E-11
EGFR	5616	3907.5	9624.5	20.20344	50.77545	1.32953	Up	8.73E-12	2.73E-11
TSC22D3	2312	1102	2710	13.82186	34.7099	1.328396	Up	2.55E-12	8.39E-12
CHIC2	1178	379	928	9.326491	23.38146	1.325959	Up	4.40E-14	1.61E-13
TNFSF4	3493	1645	4016	13.65816	34.09529	1.31981	Up	5.18E-12	1.65E-11
IGF2	4856	23	57	0.13887	0.346574	1.319433	Up	0.000127	0.000278
GJB2	2347	16	39	0.198177	0.49403	1.317805	Up	0.001842	0.00371
SPEF1	1597	22	54	0.397591	0.990532	1.316917	Up	0.000217	0.000469
NOL3	1515	1038.5	2534.5	19.90462	49.48565	1.313907	Up	2.62E-14	9.68E-14
PDXP	2072	767	1879	10.78648	26.81231	1.313671	Up	0	0
ERN1	4005	205.5	501.5	1.492617	3.705147	1.311686	Up	4.23E-13	1.48E-12
NFIL3	2104	995.5	2420	13.74926	34.11667	1.311123	Up	0	0
KRT81	1926	26	63	0.39124	0.970605	1.310829	Up	7.66E-05	0.000171
TBC1D3F	2122	102	246	1.387066	3.439001	1.309953	Up	0	0
MPP4	2475	287	697	3.365863	8.344422	1.309836	Up	4.35E-13	1.51E-12
EHD2	3601	9627	23440.5	77.78025	192.7259	1.309075	Up	5.51E-12	1.75E-11
TBC1D3B	2114	20.5	49.5	0.279458	0.692138	1.308426	Up	0.00044	0.000934
PORCN	1849	1227	2987.5	19.2997	47.7856	1.307998	Up	2.32E-12	7.67E-12
LYZ	1516	51	122.5	0.970385	2.401793	1.307483	Up	4.78E-08	1.25E-07
PFKP	2781	8405.5	20384.5	87.82189	217.099	1.305701	Up	0	0
C18orf19	4392	913.5	2208	6.031069	14.90799	1.3056	Up	0	0
SLC25A36	4677	1731	4172	10.70957	26.44687	1.304197	Up	0	0
LMO7	7253	4324	10448	17.30551	42.70739	1.303254	Up	5.89E-12	1.87E-11
C14orf45	3123	95	233	0.895689	2.21013	1.303062	Up	7.55E-15	2.83E-14

SLC25A45	2231	55.5	134	0.718284	1.771198	1.302099	Up	6.00E-09	1.63E-08
S100A10	1069	10117	24376	274.3273	676.1767	1.301502	Up	2.20E-12	7.26E-12
LAMB3	4093	10446	25258.5	74.13231	182.7205	1.301464	Up	0	0
CECR6	3979	17	41	0.123912	0.30522	1.300536	Up	0.001547	0.003139
PMEPA1	4590	5309.5	12770.5	33.53989	82.37889	1.296397	Up	0	0
LPO	2744	17	43	0.187208	0.459499	1.295417	Up	0.00073	0.001519
SLC7A5	4543	10149	24415	64.87059	158.9488	1.292925	Up	0	0
THBS1	5820	190951.5	456767.5	951.301	2326.339	1.290087	Up	2.37E-10	6.80E-10
SLC8A1	6014	644.5	1545	3.118307	7.612293	1.287568	Up	5.13E-13	1.78E-12
PAQR7	3023	571	1364.5	5.478775	13.36022	1.286019	Up	0	0
BAIAP3	4583	153.5	367.5	0.973531	2.370332	1.28379	Up	0	0
CDK5R2	2495	37	88.5	0.427881	1.040841	1.282468	Up	4.10E-06	9.82E-06
CPNE5	3339	37	89.5	0.325224	0.790697	1.281692	Up	2.87E-06	6.93E-06
FAM167A	4093	1408.5	3361.5	10.00949	24.30906	1.280126	Up	5.18E-12	1.65E-11
LOC100507421	10558	27	68	0.077276	0.187502	1.27882	Up	2.15E-05	4.95E-05
ADRB2	2058	55	133.5	0.788608	1.91341	1.278766	Up	8.56E-09	2.31E-08
PAG1	10759	679	1623	1.843255	4.463759	1.276004	Up	1.60E-13	5.71E-13
B3GALT4	1704	31	74.5	0.532225	1.288052	1.275082	Up	2.30E-05	5.28E-05
FAM107B	3785	1902.5	4504	14.58505	35.28722	1.274655	Up	5.11E-12	1.63E-11
ESAM	1869	30.5	71.5	0.46856	1.133276	1.274193	Up	3.89E-05	8.84E-05
PANX1	2782	948.5	2249	9.901536	23.93864	1.273617	Up	0	0
TNFRSF9	6001	72	171	0.349931	0.845158	1.272152	Up	1.29E-10	3.76E-10
DACT1	3877	187	445	1.408361	3.398775	1.270998	Up	3.37E-13	1.18E-12
VWCE	3536	45	106	0.368331	0.888085	1.269694	Up	5.29E-07	1.32E-06
HHAT	3507	177.5	419.5	1.473129	3.549503	1.268733	Up	0	0

ZNF385A	2353	288	682	3.568826	8.574439	1.264593	Up	4.49E-14	1.64E-13
ZNF664-FAM101A	2413	42.5	100	0.510822	1.227106	1.264368	Up	8.74E-07	2.16E-06
ZNF654	4957	760	1785.5	4.446345	10.67543	1.263602	Up	2.39E-12	7.86E-12
TUBB6	1843	3805	8952.5	60.00487	143.8557	1.261471	Up	2.85E-12	9.32E-12
LOC388849	627	25	58.5	1.157267	2.764762	1.256434	Up	0.000266	0.000573
LOXL2	3810	43509.5	101958.5	331.8341	792.3523	1.25568	Up	2.53E-10	7.27E-10
EIF4EBP1	877	2231.5	5226	73.87326	176.356	1.255366	Up	0	0
FEM1C	5813	1200	2800	5.98468	14.28619	1.255275	Up	1.80E-12	5.99E-12
ASL	1937	2504	5868.5	37.56952	89.66603	1.254999	Up	0	0
C11orf45	3648	92	217	0.736903	1.758535	1.254826	Up	6.09E-13	2.10E-12
FBN1	11695	22087.5	51391.5	54.57916	130.1591	1.253854	Up	0	0
FRMD6	4842	2698.5	6291.5	16.17588	38.5522	1.252969	Up	0	0
TSGA10	3664	23	56	0.188432	0.449086	1.252948	Up	0.000183	0.000398
PGAM2	888	88	207.5	2.898916	6.895482	1.250138	Up	2.34E-12	7.72E-12
TNIP1	3268	5033	11756.5	44.76631	106.4778	1.250068	Up	1.54E-11	4.74E-11
BHLHE40	3061	3962.5	9223.5	37.55799	89.30414	1.249607	Up	9.16E-12	2.86E-11
AGPAT5	5535	2050.5	4766	10.73877	25.52178	1.2489	Up	0	0
LRP1	14905	11695	27144.5	22.67731	53.86842	1.248191	Up	4.59E-13	1.60E-12
HDAC9	4238	434.5	1009	2.978109	7.057513	1.244763	Up	0	0
LRRC7	6313	46	109	0.215093	0.509586	1.244367	Up	3.15E-07	7.96E-07
SLAMF8	3316	105	248	0.937452	2.214487	1.240156	Up	0	0
PTGS2	4507	1288	2980.5	8.299963	19.60394	1.239967	Up	6.74E-12	2.13E-11
F11R	4830	138.5	323.5	0.837743	1.978671	1.239953	Up	1.83E-13	6.53E-13
ALOX5AP	921	46.5	108	1.474564	3.48021	1.238887	Up	4.47E-07	1.12E-06
KDELR3	1705	1837.5	4235.5	31.2374	73.59863	1.236404	Up	3.07E-12	1.00E-11

ARL6	1408	162	372	3.32679	7.834237	1.235662	Up	0	0
RHPN1	3795	35.5	82.5	0.273597	0.643282	1.233398	Up	1.16E-05	2.72E-05
EPS8L2	3156	555.5	1288.5	5.146747	12.09208	1.23233	Up	0	0
ETS1	5143	3624	8302.5	20.43065	47.88888	1.228955	Up	0	0
SLC19A2	3655	389.5	895	3.09429	7.25195	1.228761	Up	0	0
FHL2	1727	1309.5	3005.5	22.01956	51.5701	1.227749	Up	5.27E-12	1.68E-11
TCP11L2	2243	137.5	313.5	1.76952	4.143982	1.22766	Up	2.87E-13	1.01E-12
BTG1	4704	1551	3552	9.567185	22.3877	1.22654	Up	2.30E-12	7.59E-12
DUSP14	1508	1080	2471	20.77077	48.54586	1.224793	Up	5.71E-12	1.81E-11
NCKIPSD	2968	906	2075	8.859149	20.68953	1.223661	Up	0	0
ZFP36	1745	457	1047	7.613872	17.76968	1.222716	Up	1.73E-12	5.76E-12
TLN2	11650	400	920	1.002254	2.338355	1.222246	Up	0	0
NPR3	7447	178	415	0.705936	1.644471	1.220015	Up	1.14E-13	4.11E-13
UGCG	1637	1197	2726.5	21.23202	49.4453	1.219592	Up	2.02E-12	6.69E-12
FSTL3	2525	6275.5	14302	72.18555	167.8182	1.217117	Up	1.75E-11	5.35E-11
ATP2B3	6420	16	38	0.075309	0.17454	1.212667	Up	0.002667	0.005299
C7	4034	124	287	0.905526	2.098539	1.212557	Up	1.28E-13	4.59E-13
OCEL1	1124	194	439	4.997029	11.57597	1.21199	Up	5.28E-13	1.83E-12
AHNAK	1108	18638	42072	485.8184	1124.905	1.211315	Up	0	0
NEDD4L	8300	777	1761.5	2.719174	6.295837	1.211229	Up	0	0
DUSP6	2404	1238	2800	14.93985	34.50099	1.207472	Up	2.93E-12	9.56E-12
PLCB4	5640	1046	2360	5.38141	12.42257	1.206907	Up	1.19E-12	4.02E-12
APAF1	7204	1165.5	2628	4.708932	10.81808	1.199973	Up	2.00E-12	6.62E-12
B4GALNT4	3463	269.5	606.5	2.260827	5.1866	1.197939	Up	5.77E-14	2.11E-13
C1orf9	5480	2435.5	5461	12.90522	29.56676	1.196021	Up	0	0

TMEM67	4744	260.5	584.5	1.591326	3.644415	1.195457	Up	4.56E-13	1.59E-12
COL17A1	5610	81	185	0.424843	0.971138	1.192747	Up	1.14E-10	3.31E-10
FAM63B	5013	674	1514	3.919023	8.95732	1.192573	Up	2.58E-14	9.52E-14
KDM4C	3850	1208.5	2711.5	9.128733	20.86268	1.192438	Up	3.18E-12	1.04E-11
BACH1	5770	1376	3071	6.915398	15.78035	1.190245	Up	3.69E-12	1.19E-11
RELT	3520	735.5	1645.5	6.064559	13.83522	1.189871	Up	9.46E-13	3.22E-12
EPB41L4B	3783	95	217	0.743062	1.695023	1.18975	Up	2.82E-12	9.23E-12
EBI3	1149	23.5	53	0.600052	1.367146	1.188008	Up	0.000539	0.001134
MAPK7	2972	964.5	2156	9.44202	21.48464	1.186138	Up	0	0
EPPK1	15534	25	55.5	0.046563	0.105824	1.184411	Up	0.000752	0.001564
CEP250	8341	2283	5084.5	7.937616	18.035	1.184021	Up	0	0
PNPLA7	4731	96	213.5	0.589398	1.336094	1.180709	Up	1.72E-11	5.27E-11
SNTB1	4975	501	1112	2.921931	6.623475	1.180666	Up	7.99E-14	2.90E-13
DNAJB4	2250	2581	5702.5	33.26969	75.23549	1.177205	Up	0	0
CTXN1	1290	250.5	556.5	5.640125	12.74688	1.176345	Up	2.66E-13	9.36E-13
IBA57	7921	315	700.5	1.157212	2.615113	1.17622	Up	4.99E-13	1.73E-12
CPEB4	7769	1449.5	3209.5	5.422356	12.25238	1.17607	Up	6.65E-12	2.10E-11
HS3ST3A1	2546	19	42.5	0.219195	0.495176	1.175727	Up	0.003156	0.006239
CCR10	1244	24	53.5	0.562684	1.269188	1.17351	Up	0.000901	0.001864
ATP2B4	8733	4692	10363	15.60322	35.17145	1.172561	Up	2.82E-11	8.46E-11
GXYLT2	1547	289.5	640	5.447871	12.2709	1.171476	Up	0	0
ACTA2	1415	684.5	1509	14.03539	31.5707	1.169517	Up	0	0
SLAMF7	2672	27	62	0.304484	0.68451	1.168706	Up	0.000188	0.000408
OSR1	1911	320.5	704.5	4.857736	10.88395	1.163846	Up	0	0
ADAMTS9	7335	1169	2572.5	4.636435	10.38719	1.163718	Up	2.25E-12	7.42E-12

SCD	5473	15139	33214.5	80.29153	179.7796	1.16291	Up	0	0
METRNL	1348	3450.5	7584.5	74.41522	166.5745	1.162498	Up	0	0
TSPAN5	3406	1280	2799.5	10.89041	24.37433	1.162304	Up	3.82E-12	1.23E-11
RYR1	15376	123	282	0.241725	0.540696	1.161449	Up	0	0
CHST3	6978	285.5	626	1.188809	2.655928	1.159699	Up	6.22E-13	2.14E-12
KDM4B	5675	1924	4218.5	9.847388	21.97378	1.15797	Up	5.28E-12	1.68E-11
SEC61G	480	6985.5	15190.5	421.5591	940.2842	1.157362	Up	2.60E-11	7.82E-11
CECR5	1734	599	1309.5	10.03086	22.34509	1.155512	Up	7.53E-13	2.58E-12
PCDH1	3851	104.5	236	0.808359	1.7998	1.154769	Up	3.44E-13	1.21E-12
C1orf96	5121	880	1914	4.98627	11.07887	1.151778	Up	1.52E-12	5.08E-12
MAFK	3350	3932.5	8562	34.05724	75.62193	1.150843	Up	8.08E-12	2.53E-11
GPM6B	1799	19	43	0.317865	0.704848	1.148897	Up	0.002228	0.004459
HIST1H2BD	829	314	681	10.99982	24.33785	1.145722	Up	1.32E-13	4.73E-13
TMEM184A	6293	628.5	1363	2.895388	6.405565	1.145569	Up	5.00E-13	1.74E-12
JUN	3338	2624	5676	22.81869	50.37334	1.142445	Up	0	0
COL5A1	8439	89888	194707	309.4913	682.6218	1.141188	Up	0	0
SLC2A13	7018	926.5	2000	3.835239	8.450763	1.139765	Up	0	0
ZFPM1	3241	325	702.5	2.913317	6.413377	1.138421	Up	9.53E-14	3.45E-13
IER3	1254	4974.5	10744	115.2751	253.6074	1.137516	Up	2.92E-11	8.74E-11
GPX3	1779	23	51	0.386802	0.850078	1.136002	Up	0.00108	0.002218
BMPER	3399	392	850.5	3.372183	7.400315	1.133904	Up	0	0
MT2A	466	35276.5	75871	2200.167	4820.74	1.131641	Up	1.56E-10	4.51E-10
ADCY5	6098	28	63	0.137244	0.300688	1.131526	Up	0.000223	0.000481
RLTPR	4512	55	118	0.354103	0.775443	1.130853	Up	1.38E-06	3.37E-06
CGB8	904	28	61	0.910557	1.99283	1.129997	Up	0.000438	0.000929

NAT8L	2327	19	41	0.237851	0.520059	1.128613	Up	0.004444	0.00865
UBR5	10900	5430	11578.5	14.42457	31.47599	1.125723	Up	1.05E-11	3.26E-11
MLXIP	5400	1139	2441.5	6.133136	13.37621	1.124973	Up	3.20E-12	1.04E-11
GCHFR	713	38.5	83.5	1.588214	3.463482	1.124818	Up	3.79E-05	8.63E-05
EFTUD1	3685	527.5	1129.5	4.168026	9.085516	1.124204	Up	6.15E-13	2.12E-12
TANC2	11747	1466	3148.5	3.642544	7.935986	1.123463	Up	1.14E-12	3.86E-12
PARM1	5069	238	511.5	1.370789	2.98533	1.122884	Up	6.71E-13	2.31E-12
AGAP1	4371	750.5	1605.5	4.992784	10.87267	1.12279	Up	0	0
BDKRB1	1307	43	93.5	0.973082	2.11673	1.121204	Up	1.57E-05	3.64E-05
PHLDA3	1516	3162.5	6755	60.54242	131.6879	1.121105	Up	0	0
CHAC1	1443	302.5	648	6.109561	13.28042	1.12016	Up	5.71E-13	1.97E-12
SAMD4A	6268	488.5	1041.5	2.264957	4.920869	1.119429	Up	1.35E-12	4.55E-12
TUBGCP2	3198	3039.5	6475.5	27.61914	59.94469	1.117963	Up	0	0
BHLHE41	3796	1570.5	3330	11.98518	25.98552	1.116456	Up	6.35E-12	2.01E-11
KLF7	1639	211.5	448	3.736921	8.093503	1.114914	Up	2.52E-13	8.92E-13
MKNK2	3805	3744	7957.5	28.61467	61.90567	1.113317	Up	6.16E-12	1.95E-11
AEN	3134	2203	4679	20.45971	44.24884	1.112854	Up	0	0
ADRA1D	2666	125.5	266	1.363076	2.940285	1.10909	Up	6.02E-13	2.08E-12
HIVEP2	9732	1442.5	3067	4.328831	9.323598	1.106909	Up	4.51E-12	1.45E-11
IL32	892	1139.5	2404	37.08919	79.84137	1.106138	Up	3.20E-12	1.04E-11
TMEM191B	1334	41.5	88	0.907367	1.951957	1.105163	Up	3.09E-05	7.07E-05
COL4A1	6549	19700.5	41523	87.32642	187.7001	1.103939	Up	0	0
TBC1D3	2079	166	347.5	2.299061	4.93803	1.102891	Up	2.17E-13	7.69E-13
NR4A3	5707	43	93	0.223657	0.480352	1.102806	Up	1.57E-05	3.64E-05
IMMP2L	1539	143	299.5	2.689941	5.776033	1.102504	Up	0	0

GAL3ST4	2561	30.5	65	0.349569	0.750295	1.101881	Up	0.000306	0.000654
LOC643699	1788	27.5	56.5	0.437803	0.93757	1.098646	Up	0.001404	0.002861
RNF207	3987	21	44.5	0.154267	0.330177	1.09781	Up	0.004272	0.00833
PFKL	2924	6945	14604	69.10858	147.7596	1.096315	Up	4.10E-11	1.22E-10
PITPNC1	2005	141.5	301	2.075335	4.432007	1.094616	Up	1.44E-14	5.37E-14
NEDD9	4745	531	1124	3.286775	7.016769	1.094134	Up	4.93E-14	1.80E-13
DPCD	858	563.5	1176.5	19.04196	40.62968	1.093352	Up	1.35E-13	4.85E-13
RNF182	3284	21	46	0.192532	0.409927	1.090271	Up	0.002186	0.00438
ADRA1B	2272	103	215.5	1.321416	2.812658	1.089849	Up	2.35E-10	6.76E-10
FOSL2	4015	3015	6294.5	21.82805	46.44767	1.089423	Up	0	0
PYGL	2757	1481.5	3088.5	15.60843	33.18517	1.088214	Up	5.28E-12	1.68E-11
RALGAPA2	9453	456.5	957.5	1.408516	2.993603	1.087707	Up	1.95E-12	6.46E-12
ARHGAP22	2775	720	1504.5	7.549969	16.0397	1.087105	Up	0	0
CCRN4L	1971	427	890.5	6.305363	13.39025	1.086532	Up	1.02E-12	3.46E-12
PLAUR	1455	2955	6161	59.01099	125.259	1.085859	Up	0	0
DUSP8	4491	211	437	1.362222	2.882262	1.08124	Up	2.52E-13	8.92E-13
CRIM1	5628	12224	25326.5	63.07851	133.3712	1.080227	Up	3.57E-11	1.06E-10
PIP5KL1	1131	29	60	0.745388	1.574876	1.079174	Up	0.000973	0.002006
KIAA1984	1727	42	86.5	0.700331	1.479328	1.078833	Up	9.21E-05	0.000204
HECW2	6926	812	1680.5	3.412143	7.190963	1.075507	Up	1.63E-12	5.45E-12
ADAM12	6093	4790.5	9898	22.83426	48.12031	1.075446	Up	2.40E-11	7.24E-11
PTPRH	3935	62	132	0.469111	0.987685	1.074121	Up	4.04E-07	1.02E-06
SLC35A3	2149	607.5	1246	8.196205	17.2123	1.070412	Up	0	0
PARP8	7051	1161	2384	4.773111	10.01753	1.069525	Up	3.11E-12	1.01E-11
FAM117B	5795	340.5	701	1.712942	3.583062	1.064717	Up	1.85E-13	6.59E-13

PLOD1	3047	2694	5524.5	25.6786	53.69443	1.064206	Up	0	0
TBC1D3H	2305	25	52	0.317784	0.664291	1.063772	Up	0.002031	0.004083
HDAC11	2918	179	366.5	1.782868	3.716602	1.059784	Up	0	0
NRG4	2195	41	84.5	0.544564	1.134956	1.059462	Up	0.00011	0.000243
F2RL1	2883	6439.5	13132	64.7919	134.9745	1.058802	Up	3.22E-11	9.62E-11
C9orf25	3692	1709.5	3485.5	13.43963	27.95759	1.056746	Up	5.11E-15	1.93E-14
RAB11FIP5	4362	2099.5	4288	13.98916	29.07927	1.055681	Up	0	0
TPI1	1366	24731	50408.5	525.5079	1092.25	1.055519	Up	3.18E-11	9.49E-11
EDEM1	6162	5514.5	11213	25.96007	53.94891	1.055299	Up	2.22E-11	6.71E-11
LRRC17	2303	5634.5	11428	70.94167	147.0166	1.051274	Up	2.50E-11	7.54E-11
FAM101A	2160	37	75.5	0.499555	1.032171	1.046968	Up	0.00031	0.000662
PAPPA	11025	5490.5	11102	14.44059	29.82395	1.046342	Up	1.66E-11	5.09E-11
IRF7	2051	170.5	346	2.414089	4.983243	1.045606	Up	3.83E-13	1.34E-12
FOXD1	2272	937	1897	11.9914	24.7206	1.043713	Up	0	0
RRAGD	4926	43	90	0.262378	0.539477	1.039916	Up	4.11E-05	9.34E-05
NKX3-1	3281	694	1396.5	6.144397	12.61901	1.038255	Up	7.61E-13	2.60E-12
TUFT1	3083	145	291	1.369092	2.807538	1.036086	Up	1.79E-12	5.94E-12
ANKRD36B	5986	120	240.5	0.580654	1.190497	1.035813	Up	1.83E-10	5.28E-10
TRIO	10244	6889.5	13823	19.51382	39.98625	1.035008	Up	3.41E-11	1.02E-10
FNIP2	5801	600	1204	3.001002	6.146712	1.03437	Up	0	0
ZXDA	5204	81	162	0.45005	0.921274	1.033545	Up	1.67E-07	4.26E-07
CTH	2008	150.5	301	2.169956	4.440357	1.03301	Up	7.57E-13	2.59E-12
GRIK2	4724	195	389	1.195359	2.441976	1.030605	Up	1.93E-13	6.84E-13
SLC15A4	2789	677.5	1356.5	7.067235	14.43156	1.03001	Up	5.28E-13	1.83E-12
GGT1	2393	260	519.5	3.151286	6.43087	1.029073	Up	4.56E-13	1.59E-12

KIF17	3810	53	109.5	0.414326	0.8444	1.027159	Up	9.47E-06	2.22E-05
PGAM1	1762	19683	39305	324.388	660.5438	1.025934	Up	0	0
CSPG4	8305	137	273.5	0.477748	0.972656	1.025681	Up	1.31E-11	4.04E-11
HRH1	4427	651	1294	4.258759	8.664603	1.024701	Up	2.50E-13	8.82E-13
LOC100287177	960	29	58.5	0.886528	1.803498	1.024561	Up	0.001824	0.003677
FLVCR2	2787	37	76	0.392932	0.798986	1.023889	Up	0.000227	0.00049
BCKDK	2097	2745	5474	38.05758	77.16871	1.019832	Up	0	0
C5orf13	2099	1131.5	2228	15.61088	31.46705	1.01129	Up	5.50E-12	1.75E-11
FAM120AOS	2779	659	1301	6.882008	13.87007	1.011073	Up	0	0
IMPAD1	7239	9755	19230	39.07634	78.74768	1.010942	Up	0	0
PGAM4	1678	547.5	1080.5	9.459403	19.05748	1.010536	Up	0	0
LOC390940	938	58	116.5	1.817087	3.657102	1.009073	Up	9.69E-06	2.27E-05
LOC100652762	1905	106	208.5	1.610324	3.239599	1.008465	Up	6.72E-09	1.82E-08
FLRT1	3252	26.5	53	0.239887	0.482161	1.007161	Up	0.002325	0.004644
ACTBL2	2814	94	191.5	0.995537	2.00033	1.006691	Up	6.98E-09	1.89E-08
RELB	2297	741.5	1464.5	9.393446	18.86981	1.006354	Up	0	0
VEGFC	2076	2534.5	4980	35.38743	71.08654	1.00634	Up	0	0
SLC38A4	3807	436.5	858.5	3.332349	6.6883	1.0051	Up	4.26E-13	1.49E-12
SLC45A1	2401	177	351.5	2.160713	4.326953	1.001844	Up	0	0
HERPUD1	2198	2394	4701.5	31.60393	63.27753	1.001589	Up	0	0
LOC100653202	1435	11.5	0	0.231767	0.001	-7.85653	Down	0.000488	0.001031
LOC100130589	1356	8	0	0.171505	0.001	-7.42211	Down	0.003906	0.007651
CCDC169-SOHLH2	2650	11.5	0	0.125504	0.001	-6.97159	Down	0.000488	0.001031
FOXD4L5	3109	8	0	0.077017	0.001	-6.26711	Down	0.003906	0.007648
GRASP	1950	24	1	0.362494	0.014832	-4.61115	Down	8.04E-07	2.00E-06

HIST1H2BF	430	12	0.5	0.808592	0.033631	-4.58753	Down	0.000244	0.000526
HIST1H3I	477	10	0.5	0.628682	0.030318	-4.37411	Down	0.000977	0.002013
LOC100653286	694	10	0.5	0.412265	0.021869	-4.2366	Down	0.000977	0.002013
CCDC48	2691	318.5	20	3.440457	0.219748	-3.96868	Down	1.78E-70	1.69E-69
RAB7B	3032	390.5	27.5	3.757842	0.267994	-3.80963	Down	7.30E-84	7.86E-83
PRB3	1091	13	1	0.360063	0.02651	-3.76362	Down	0.000976	0.002013
NKX6-1	1116	14	1	0.368792	0.027199	-3.76116	Down	0.000519	0.001093
AKR1C1	1384	3599	272.5	75.35871	5.828155	-3.69266	Down	0	0
HSPA12B	3175	12	1	0.111317	0.00911	-3.61115	Down	0.001831	0.003689
ITGB1BP2	1235	12	1	0.286179	0.023419	-3.61115	Down	0.001831	0.003689
VN1R4	906	23	2	0.767114	0.063848	-3.58674	Down	1.05E-05	2.46E-05
PRRX2	1327	12	1	0.261151	0.021796	-3.58276	Down	0.001831	0.003688
CDC20	1697	6132.5	551.5	105.0662	9.618432	-3.44935	Down	0	0
AKR1C2	1663	480	43	8.378933	0.768516	-3.44662	Down	9.60E-95	1.13E-93
KLHL14	4261	155	14	1.053429	0.097381	-3.4353	Down	1.63E-31	8.94E-31
S1PR5	2467	33	3	0.388394	0.036332	-3.41819	Down	1.23E-07	3.17E-07
IMPA2	1537	93	9	1.759708	0.173086	-3.34578	Down	5.46E-19	2.28E-18
ZNF323	3039	582.5	60	5.558614	0.580927	-3.2583	Down	########	########
GUCY1B3	3266	1898.5	199	16.89105	1.802184	-3.22844	Down	0	0
SUCNR1	1650	268.5	29	4.719032	0.514416	-3.19748	Down	6.88E-50	5.08E-49
HIST1H2BL	453	13	1.5	0.844375	0.097351	-3.11661	Down	0.000976	0.002013
MEX3A	6124	319	37	1.512769	0.179539	-3.07483	Down	2.27E-57	1.85E-56
HIST1H2AJ	439	16.5	2	1.104376	0.131767	-3.06717	Down	0.000729	0.001517
IFI44L	5889	1698	199	8.355224	1.000451	-3.06203	Down	########	########
GBP4	6160	1314.5	155	6.184642	0.745314	-3.05277	Down	########	########

HIST1H2BM	446	16	2	1.073749	0.129699	-3.04941	Down	0.000729	0.001516
CCNB1	2101	7233	856.5	99.81883	12.06846	-3.04807	Down	0	0
CORT	1238	12	1.5	0.285486	0.035044	-3.02618	Down	0.001831	0.003688
GUCY1A3	1658	1600	203	28.00962	3.617207	-2.95297	Down	########	########
LOC727983	2051	26	3.5	0.371872	0.049357	-2.91349	Down	8.43E-06	1.99E-05
CYS1	2719	15	2	0.158262	0.021801	-2.85984	Down	0.001312	0.00268
MFI2	1696	22	3	0.378442	0.053693	-2.81727	Down	8.80E-05	0.000196
PLK1	2204	5605.5	785	73.8707	10.52298	-2.81146	Down	0	0
SLC14A1	4226	494.5	70	3.401199	0.486197	-2.80643	Down	8.17E-80	8.50E-79
ASPM	10906	5354	754.5	14.22774	2.04013	-2.80197	Down	0	0
SLC43A2	3024	1045	148	10.06243	1.447257	-2.79759	Down	########	########
SOX18	1718	49	7	0.829793	0.120346	-2.78556	Down	4.24E-09	1.16E-08
ARL9	836	34.5	5	1.196234	0.174696	-2.77558	Down	1.38E-06	3.39E-06
NR0B1	1591	13	2	0.246907	0.036358	-2.76362	Down	0.004181	0.008156
FOXD3	2078	14	2	0.193644	0.028526	-2.76305	Down	0.00235	0.004692
CDK15	1534	492.5	71.5	9.322302	1.377032	-2.75912	Down	1.00E-78	1.03E-77
C10orf55	2516	70	10	0.804227	0.118939	-2.75738	Down	1.80E-12	5.98E-12
HIST1H4D	367	20	3	1.602964	0.240328	-2.73767	Down	0.000277	0.000595
FAM64A	1517	651	96	12.49851	1.87562	-2.73632	Down	########	########
KIF20A	3471	3413.5	510	28.53585	4.343729	-2.71577	Down	0	0
NRG2	3945	159	24	1.166704	0.178497	-2.70847	Down	7.29E-26	3.56E-25
HIST1H2BE	435	32	5	2.170148	0.332447	-2.70659	Down	4.26E-06	1.02E-05
KIAA1257	1730	29.5	4.5	0.495373	0.076061	-2.7033	Down	6.16E-06	1.46E-05
MCOLN2	3060	849.5	129	8.055855	1.246434	-2.69223	Down	########	########
LMNB1	2267	4254.5	649	54.4436	8.49006	-2.68092	Down	0	0

HIST2H3C	507	39	6	2.220323	0.34793	-2.6739	Down	3.10E-07	7.84E-07
EXOC3L2	1719	19.5	3	0.331435	0.052142	-2.66821	Down	0.000488	0.001032
LOC440243	2400	18	3	0.226632	0.036154	-2.64814	Down	0.000855	0.001773
RSPO3	2165	51.5	8	0.688062	0.110181	-2.64267	Down	5.21E-09	1.42E-08
DDO	1556	70.5	11	1.316018	0.210908	-2.64149	Down	6.81E-12	2.15E-11
HIST2H3D	457	167.5	27	10.68118	1.74795	-2.61133	Down	4.46E-26	2.18E-25
BATF2	2142	107	17.5	1.454829	0.238972	-2.60594	Down	2.22E-17	8.95E-17
C19orf76	2112	49	8	0.674993	0.110912	-2.60545	Down	1.57E-08	4.20E-08
DEPDC1	4504	4154	670	26.72453	4.409748	-2.5994	Down	0	0
E2F2	5201	424.5	69	2.370578	0.393482	-2.59087	Down	1.37E-63	1.20E-62
C14orf176	1526	15.5	2.5	0.296401	0.04926	-2.58907	Down	0.001312	0.00268
METTL7A	3390	991.5	163	8.478175	1.418982	-2.5789	Down	########	########
MAP2K6	1879	160.5	26.5	2.466925	0.416669	-2.56574	Down	5.68E-25	2.72E-24
IDO1	1944	558.5	93	8.341358	1.412378	-2.56216	Down	5.96E-82	6.32E-81
POLE2	1783	217	36	3.537339	0.600031	-2.55955	Down	6.15E-33	3.47E-32
ALDH1A3	3510	1962	326.5	16.21449	2.763209	-2.55287	Down	########	########
ACY3	1317	111	19	2.448361	0.420524	-2.54156	Down	3.11E-17	1.25E-16
FAM72A	1881	443.5	74.5	6.81112	1.172936	-2.53777	Down	2.55E-65	2.29E-64
PLA2G4A	2940	1771.5	299.5	17.48132	3.01676	-2.53474	Down	########	########
IL7	1957	217.5	37	3.214132	0.55634	-2.53039	Down	2.13E-32	1.19E-31
AURKA	2554	3357.5	582	38.14589	6.745594	-2.49951	Down	0	0
ZNF843	2013	17	3	0.246071	0.043815	-2.48956	Down	0.00149	0.003027
PLEKHG4	4493	2599	459.5	16.80659	3.02327	-2.47485	Down	0	0
HOXA7	2018	164	29	2.367607	0.426992	-2.47115	Down	2.67E-24	1.26E-23
HIST1H2AB	477	16.5	3	1.016396	0.183406	-2.47035	Down	0.002577	0.005124

LYPD6B	1600	56	10	1.020324	0.184347	-2.46853	Down	4.04E-09	1.10E-08
TDO2	1713	220	39	3.722752	0.676876	-2.45941	Down	4.87E-32	2.69E-31
PDK4	3710	1228.5	220.5	9.618482	1.76222	-2.44841	Down	########	########
ALDH3A2	3823	5085.5	914	38.55241	7.076074	-2.4458	Down	0	0
PCDH18	5906	8248.5	1492	40.50943	7.464675	-2.44011	Down	0	0
PTPRC	4847	142.5	26	0.850982	0.157509	-2.43369	Down	8.94E-21	3.89E-20
TNFRSF11B	2354	5440.5	997	67.02747	12.54571	-2.41756	Down	0	0
C10orf91	738	21	4	0.834973	0.156764	-2.41314	Down	0.000534	0.001123
KRT12	1992	18	3.5	0.271899	0.051537	-2.39939	Down	0.000855	0.001773
C8orf80	3912	347.5	65.5	2.577733	0.489756	-2.39597	Down	7.49E-48	5.37E-47
PTPRCAP	917	35	6.5	1.108296	0.21126	-2.39125	Down	2.83E-06	6.83E-06
ASF1A	2525	1277	239	14.64021	2.799452	-2.38672	Down	########	########
CROT	3342	3213.5	602.5	27.81421	5.343837	-2.37987	Down	0	0
VMO1	765	15.5	3	0.588253	0.113423	-2.37472	Down	0.004425	0.008617
KRT39	1696	20	4	0.353633	0.068214	-2.37411	Down	0.000911	0.001882
LCP2	2472	36	7	0.428922	0.083349	-2.36347	Down	5.30E-06	1.26E-05
LHX8	2393	151	29.5	1.844417	0.359542	-2.35893	Down	2.52E-21	1.11E-20
PIK3R3	5194	2561	489	14.28985	2.790948	-2.35616	Down	0	0
C9orf140	3865	1520.5	292	11.44057	2.235309	-2.35561	Down	########	########
RHBG	1805	15	3	0.24603	0.048071	-2.35559	Down	0.004425	0.008616
MCM10	4562	929.5	177.5	5.893693	1.153743	-2.35285	Down	########	########
GBP2	2595	1160	223	12.97485	2.542849	-2.3512	Down	########	########
SLC7A11	9648	22635	4386.5	67.93289	13.48777	-2.33246	Down	0	0
SOCS1	1216	153	30	3.65107	0.727686	-2.32693	Down	2.74E-21	1.20E-20
CDCA7	2602	776.5	152.5	8.650323	1.727047	-2.32445	Down	########	########

MCM3	3143	3474.5	677.5	32.00829	6.392399	-2.32402	Down	0	0
SP110	2519	1283	252.5	14.763	2.95999	-2.31832	Down	########	########
VIPR1	2808	10842.5	2135	112.103	22.4793	-2.31816	Down	0	0
COX7B2	566	61	12	3.12692	0.628382	-2.31503	Down	2.86E-09	7.86E-09
CYP1B1	5160	22052.5	4343	123.9291	24.95672	-2.31201	Down	0	0
SLC22A11	2545	19	4	0.225593	0.045458	-2.3111	Down	0.001544	0.003133
LOC100127983	1053	212	42	5.850501	1.179451	-2.31044	Down	1.01E-28	5.26E-28
KLRK1	1606	50	10	0.910764	0.18455	-2.30307	Down	9.62E-08	2.49E-07
SLC16A14	4411	472	95	3.102333	0.6333	-2.29239	Down	2.76E-61	2.35E-60
HIST1H1D	777	30	6	1.135691	0.232555	-2.28793	Down	4.13E-05	9.37E-05
FZD2	3834	1786	360.5	13.5451	2.780214	-2.2845	Down	########	########
SPTSSB	2306	30	6	0.375204	0.077117	-2.28255	Down	4.13E-05	9.37E-05
CLDN11	2431	607	122	7.237801	1.491838	-2.27846	Down	2.09E-78	2.14E-77
TRPV2	2829	68	14	0.697941	0.143891	-2.27813	Down	6.80E-10	1.92E-09
DLGAP5	2993	1949.5	394.5	18.86305	3.910311	-2.27021	Down	########	########
CCNB2	1566	2109	428	39.01008	8.104143	-2.26712	Down	########	########
CDC25B	3578	13201.5	2689	107.089	22.26138	-2.26619	Down	0	0
SLC37A2	4196	3379	690.5	23.39734	4.872356	-2.26365	Down	0	0
HNF4G	4101	196	40	1.38376	0.288389	-2.2625	Down	4.13E-26	2.03E-25
CRISPLD1	4334	24	5	0.163097	0.034028	-2.26093	Down	0.000325	0.000694
HECW1	6839	560.5	116.5	2.388274	0.500123	-2.25561	Down	7.52E-71	7.18E-70
H2AFX	1651	5776	1191.5	101.778	21.35487	-2.25279	Down	0	0
CLDN10	2658	19	4	0.209095	0.044064	-2.24647	Down	0.001544	0.003134
HJURP	3065	3874.5	803	36.67246	7.746599	-2.24306	Down	0	0
IFIH1	3434	743	154	6.262051	1.325415	-2.24019	Down	2.18E-93	2.54E-92

CCNA2	2811	4344.5	903.5	44.832	9.52571	-2.23463	Down	0	0
FAM72D	2393	707	147	8.570411	1.823672	-2.23252	Down	9.79E-89	1.10E-87
MCM5	2568	2110	441.5	23.87703	5.082912	-2.2319	Down	########	########
MPZ	1980	171.5	36	2.510701	0.536716	-2.22586	Down	1.86E-22	8.43E-22
NDUFAF4	2411	486.5	102	5.850885	1.252116	-2.22429	Down	4.24E-61	3.61E-60
SLC22A18AS	1565	23.5	5	0.439816	0.094235	-2.22257	Down	0.000546	0.001149
LPAR6	2561	317	67	3.583511	0.773441	-2.21201	Down	3.30E-40	2.11E-39
ETV1	6237	9963	2106.5	46.28735	10.00721	-2.20958	Down	0	0
CCRL1	2224	169.5	36	2.224582	0.481694	-2.20735	Down	5.10E-22	2.28E-21
SLC1A7	2677	368	79	4.000911	0.868507	-2.20372	Down	7.32E-46	5.10E-45
C8orf31	1875	102	22	1.585088	0.34547	-2.19793	Down	1.00E-13	3.64E-13
FAM72B	2374	555.5	118.5	6.797144	1.482604	-2.1968	Down	7.75E-69	7.23E-68
HMGB3	3556	1672	357	13.624	2.981577	-2.192	Down	########	########
ANXA10	1447	3710.5	797	74.311	16.30555	-2.18821	Down	0	0
AGXT2L1	2133	23	5	0.315075	0.069141	-2.18808	Down	0.000546	0.001149
GDPD3	1115	37.5	8	0.968948	0.212654	-2.18791	Down	9.25E-06	2.18E-05
C4orf36	922	23	5	0.721444	0.158402	-2.1873	Down	0.000546	0.001149
CDCA8	2319	2543.5	547.5	31.83814	6.999499	-2.18543	Down	########	########
FAM50B	1618	45.5	10	0.824223	0.181412	-2.18377	Down	1.25E-06	3.06E-06
OSGIN1	1958	1274	275	18.8815	4.160179	-2.18226	Down	########	########
NCAPH	4495	1751.5	382	11.29147	2.510833	-2.169	Down	########	########
OLR1	2417	107.5	24	1.305048	0.290749	-2.16626	Down	6.38E-14	2.33E-13
DCTPP1	1143	1438	318.5	36.54383	8.252953	-2.14665	Down	########	########
CCNF	4287	1812	402.5	12.26094	2.78148	-2.14015	Down	########	########
MCM7	2821	11169	2481.5	114.8674	26.08863	-2.13848	Down	0	0

FDXACB1	3081	123.5	28	1.166935	0.265174	-2.13772	Down	1.28E-15	4.94E-15
CDC42EP4	3121	2613	584.5	24.31198	5.542814	-2.13298	Down	########	########
OSR2	1825	257.5	58.5	4.115197	0.939669	-2.13074	Down	2.96E-31	1.62E-30
TMPO	3690	6976.5	1560.5	54.79093	12.53914	-2.1275	Down	0	0
HIST1H2AG	498	33	7.5	1.914815	0.438461	-2.12668	Down	2.53E-05	5.82E-05
CPT1A	5260	6139	1379.5	33.86023	7.756173	-2.12617	Down	0	0
PRC1	3011	5446.5	1224	52.44743	12.04936	-2.12192	Down	0	0
GPRC5C	2389	7282.5	1650	88.67473	20.40241	-2.11978	Down	0	0
C9orf150	2718	1493.5	339	15.96918	3.686125	-2.11511	Down	########	########
OPN1SW	1114	15	3.5	0.396579	0.092156	-2.10546	Down	0.004425	0.008615
FOXF1	2579	6532.5	1490.5	73.56119	17.11196	-2.10394	Down	0	0
IRAK1BP1	1157	35	8	0.880383	0.204935	-2.10297	Down	2.54E-05	5.85E-05
ALX4	5466	259.5	60.5	1.384628	0.32275	-2.10101	Down	8.06E-31	4.35E-30
TROAP	2618	992	229	11.01994	2.573672	-2.09822	Down	########	########
CHRFAM7A	2858	26	6	0.268474	0.062723	-2.0977	Down	0.000324	0.000692
DBP	1658	170	39	2.991728	0.699329	-2.09694	Down	6.55E-21	2.86E-20
GNG2	3875	2558.5	589	19.13206	4.499725	-2.08808	Down	########	########
BANK1	3224	80.5	18.5	0.724251	0.171072	-2.08188	Down	1.01E-10	2.96E-10
NECAB2	1636	42.5	10	0.757639	0.179416	-2.07821	Down	5.55E-06	1.32E-05
RFX5	3618	2294.5	536	18.44282	4.373706	-2.07613	Down	########	########
HIST2H2AC	437	51.5	12	3.442955	0.817153	-2.07497	Down	4.57E-07	1.15E-06
FGF1	3669	648.5	151	5.121138	1.216483	-2.07375	Down	2.80E-74	2.77E-73
PDE5A	6836	17320.5	4027.5	73.42705	17.45173	-2.07294	Down	0	0
UNG	2166	1042.5	244	13.99073	3.327565	-2.07193	Down	########	########
LBX2	1463	34	8	0.677157	0.161092	-2.0716	Down	4.19E-05	9.52E-05

DEPDC1B	2330	900.5	209.5	11.19984	2.671543	-2.06773	Down	########	########
CPXCR1	1630	17	4	0.301074	0.071855	-2.06696	Down	0.004343	0.008462
DSCC1	2291	611	144	7.776927	1.857931	-2.0655	Down	1.87E-69	1.76E-68
HIST1H4C	390	158	37	11.71829	2.80637	-2.06199	Down	3.24E-19	1.36E-18
HMGN5	2126	379.5	89	5.195892	1.245805	-2.06029	Down	7.43E-44	5.05E-43
OIP5	1249	451.5	106	10.47226	2.521104	-2.05444	Down	7.80E-52	5.90E-51
UHRF1	3922	3644	861.5	26.99233	6.507923	-2.05228	Down	0	0
GALNT14	2762	20	5	0.217148	0.052359	-2.05218	Down	0.002494	0.004968
USP13	7933	742.5	176.5	2.715437	0.656314	-2.04873	Down	1.69E-83	1.81E-82
AURKB	1253	1394	331.5	32.31852	7.826796	-2.04587	Down	########	########
TRIM47	2268	865.5	205	11.08775	2.685926	-2.04548	Down	4.08E-97	4.87E-96
HOXD3	2319	25.5	6	0.318917	0.077302	-2.0446	Down	0.000535	0.001126
C5orf39	1280	57.5	14	1.316195	0.320818	-2.03655	Down	1.65E-07	4.21E-07
FOXM1	3527	5417.5	1294	44.5552	10.87741	-2.03426	Down	0	0
BCL2	6492	127.5	30.5	0.567832	0.138639	-2.03413	Down	1.29E-15	4.95E-15
KIF15	4857	1211	289.5	7.22706	1.766974	-2.03213	Down	########	########
UBE2T	935	1628.5	389.5	50.47534	12.36557	-2.02925	Down	########	########
PRTFDC1	1926	230.5	55	3.460293	0.848238	-2.02835	Down	8.05E-27	4.02E-26
HIST1H3H	473	20	5	1.258293	0.308766	-2.02688	Down	0.002494	0.004968
SLC40A1	3381	39	9.5	0.334986	0.082538	-2.02096	Down	9.26E-06	2.18E-05
CORO1A	1943	155.5	38	2.344522	0.578182	-2.0197	Down	3.56E-18	1.46E-17
SLCO3A1	2853	645.5	157	6.576608	1.622732	-2.01892	Down	3.67E-71	3.52E-70
ORAI1	1496	779.5	189.5	15.15217	3.739774	-2.0185	Down	7.86E-86	8.64E-85
MCM6	3769	2959.5	714.5	22.74994	5.615938	-2.01826	Down	0	0
C1orf106	4175	111	27	0.774257	0.191504	-2.01544	Down	1.68E-13	5.99E-13

CENPF	10316	8512.5	2057.5	23.8919	5.910148	-2.01526	Down	0	0
PSMB8	1135	3667	887.5	93.67829	23.1873	-2.01438	Down	0	0
COQ3	1265	287	69	6.543659	1.620617	-2.01355	Down	6.25E-33	3.52E-32
MPV17L2	1360	799	195	17.08715	4.235456	-2.01232	Down	1.60E-87	1.79E-86
PARP9	3253	1620	394.5	14.43819	3.588533	-2.00842	Down	########	########
KLRC3	1042	106	26	2.950625	0.73405	-2.00707	Down	7.24E-13	2.48E-12
ATF7IP	4661	1554	381	9.685729	2.410137	-2.00675	Down	########	########
CDKN3	906	645.5	158	20.69712	5.162459	-2.0033	Down	9.35E-71	8.91E-70
CASP1	1301	1226.5	300.5	27.35918	6.836755	-2.00064	Down	########	########
CBX6	3284	2758	677.5	24.43389	6.108567	-1.99998	Down	########	########
IGFN1	11810	533	132.5	1.313429	0.328435	-1.99966	Down	3.44E-58	2.84E-57
SELENBP1	1721	1084.5	266	18.28509	4.573925	-1.99916	Down	########	########
CENPE	8630	3261.5	799.5	10.94481	2.752795	-1.99128	Down	0	0
IFI44	1742	1888	463	31.34453	7.884549	-1.99111	Down	########	########
MRVI1	6165	158	39.5	0.74819	0.188564	-1.98835	Down	2.16E-18	8.92E-18
HMMR	3144	2508.5	616.5	23.08705	5.824649	-1.98684	Down	########	########
CENPA	1431	606	149.5	12.28016	3.100177	-1.98591	Down	2.33E-66	2.11E-65
NECAB1	5140	154	38	0.866509	0.21884	-1.98534	Down	5.74E-18	2.34E-17
TNFRSF8	3686	19	5	0.155138	0.039233	-1.9834	Down	0.004077	0.007965
CDCA3	1170	810	200.5	20.04899	5.078192	-1.98114	Down	9.16E-88	1.02E-86
BDH2	2936	751.5	185.5	7.394498	1.876631	-1.97831	Down	1.12E-81	1.19E-80
SHISA3	1971	55	14	0.823417	0.209071	-1.97763	Down	4.30E-07	1.08E-06
TACC3	2788	4575	1139	47.61245	12.09925	-1.97642	Down	0	0
GMPR	1515	598	149.5	11.50182	2.927814	-1.97397	Down	1.03E-64	9.12E-64
CMPK2	3009	94.5	24	0.918882	0.234022	-1.97324	Down	3.47E-11	1.03E-10

FBXW10	3449	95	23.5	0.792065	0.201842	-1.97239	Down	8.54E-12	2.67E-11
GIMAP2	1523	219.5	55	4.171985	1.063761	-1.97156	Down	1.52E-24	7.25E-24
TMC6	2789	31	8	0.328466	0.083989	-1.96746	Down	0.000182	0.000396
RDM1	812	38.5	10	1.403056	0.35972	-1.96363	Down	3.85E-05	8.75E-05
KRT78	1785	30	8	0.505288	0.129627	-1.96274	Down	0.000294	0.000631
FAM86B1	2458	214	54.5	2.526256	0.649156	-1.96036	Down	6.46E-24	3.03E-23
ZNF771	1460	301	76	5.986156	1.538914	-1.95972	Down	5.41E-33	3.05E-32
ZNRF2	3485	357.5	90	2.976656	0.765418	-1.95937	Down	6.87E-39	4.29E-38
HOXD4	1298	30	7.5	0.660391	0.170429	-1.95415	Down	0.000116	0.000256
HSPH1	3680	10944.5	2775	86.17215	22.36598	-1.94592	Down	0	0
SOBP	6248	1393	355	6.469476	1.68058	-1.94469	Down	########	########
CCDC102B	2722	508.5	128.5	5.403018	1.405361	-1.94282	Down	1.15E-54	9.02E-54
ZNF467	2295	67.5	17	0.853754	0.222977	-1.93693	Down	2.18E-08	5.78E-08
MTHFD1	3466	3024	773	25.28155	6.604761	-1.93651	Down	0	0
C16orf59	1650	471	121	8.276649	2.164395	-1.93508	Down	6.77E-50	5.00E-49
DBF4	3923	2047.5	523.5	15.11488	3.963219	-1.93122	Down	########	########
VASH2	4337	89.5	23	0.599241	0.157345	-1.9292	Down	1.49E-10	4.32E-10
GBP1	3050	2082	534	19.7672	5.190844	-1.92907	Down	########	########
RPS6KA5	2343	89	23	1.101795	0.28942	-1.92862	Down	1.49E-10	4.32E-10
CENPV	1181	349	90	8.576013	2.25321	-1.92833	Down	2.90E-37	1.77E-36
LNP1	2233	113	29	1.467999	0.386841	-1.92404	Down	4.09E-13	1.43E-12
MCTP2	7637	921	239	3.504574	0.926794	-1.91892	Down	5.60E-95	6.62E-94
HSPA1A	2445	1262.5	328.5	14.98884	3.972617	-1.91573	Down	########	########
TESK2	3093	478	126	4.510382	1.196288	-1.91468	Down	2.21E-49	1.63E-48
ACVRL1	4126	228.5	60	1.618124	0.430657	-1.90971	Down	2.01E-24	9.53E-24

TONSL	4519	1468.5	383.5	9.424963	2.508841	-1.90947	Down	########	########
PRKACB	4317	9964	2594.5	66.88478	17.82144	-1.90806	Down	0	0
OAS1	1673	240.5	64	4.197118	1.119271	-1.90684	Down	2.61E-25	1.26E-24
KIF18B	2669	1079.5	283	11.74671	3.139439	-1.90368	Down	########	########
C15orf23	1763	1093	286	17.99126	4.808904	-1.90352	Down	########	########
LZTS1	5459	1682	442.5	8.96683	2.397691	-1.90295	Down	########	########
GULP1	3464	586.5	154	4.924106	1.317656	-1.90189	Down	2.47E-60	2.09E-59
LAP3	2100	3706.5	972.5	51.28396	13.73014	-1.90116	Down	0	0
HOXA2	1791	76	20	1.22973	0.329375	-1.90054	Down	4.59E-09	1.25E-08
SOX11	8737	245	64	0.808346	0.216617	-1.89983	Down	2.60E-26	1.28E-25
HEY2	2672	91	24	0.992186	0.266216	-1.89801	Down	1.42E-10	4.12E-10
WDR76	4037	917	239.5	6.567154	1.762696	-1.89748	Down	3.56E-94	4.18E-93
B3GALT2	3548	41	11	0.337546	0.090881	-1.89303	Down	2.25E-05	5.18E-05
MAB21L2	2785	1431.5	378	14.923	4.024831	-1.89054	Down	########	########
VARS	4308	9721.5	2571.5	65.51106	17.66887	-1.89053	Down	0	0
FOXD2	4675	118.5	31.5	0.737227	0.198863	-1.89034	Down	2.34E-13	8.29E-13
EXOSC4	948	1108.5	294	33.98276	9.177402	-1.88865	Down	########	########
TMEM220	3092	42	11	0.391162	0.105673	-1.88816	Down	1.40E-05	3.26E-05
C14orf93	2380	180.5	48	2.206377	0.596552	-1.88696	Down	2.35E-19	9.87E-19
AKR1C3	1224	2100	554.5	49.65683	13.4273	-1.88682	Down	########	########
GNAL	4027	39	10.5	0.281819	0.076302	-1.88497	Down	2.39E-05	5.49E-05
SDPR	3265	412.5	110.5	3.676061	0.9964	-1.88336	Down	2.90E-42	1.92E-41
NDP	2058	472	126	6.65271	1.804876	-1.88204	Down	3.48E-48	2.52E-47
KPNA2	2011	11602	3085.5	167.2125	45.46449	-1.87887	Down	0	0
HIST1H4A	372	21.5	6	1.721771	0.468423	-1.87801	Down	0.003719	0.007307

HSD17B11	1892	513	137	7.869393	2.141981	-1.87731	Down	3.34E-52	2.53E-51
STAT1	2798	7643	2035	79.16384	21.5487	-1.87724	Down	0	0
PBK	1899	3307.5	877	50.37093	13.71117	-1.87724	Down	0	0
GSR	3015	9502	2532.5	91.43143	24.89611	-1.87677	Down	0	0
PRMT3	2607	762	203	8.455541	2.306512	-1.87418	Down	6.89E-77	6.96E-76
APEX1	1574	2758.5	737.5	50.80193	13.87748	-1.87214	Down	########	########
ZNF618	9133	2735	735	8.704821	2.379681	-1.87105	Down	########	########
CEP55	2656	1993.5	535	21.7674	5.975803	-1.86496	Down	########	########
TRIL	5056	44	12	0.253892	0.069779	-1.86335	Down	1.31E-05	3.06E-05
DNAJC15	2792	2530.5	680	26.29379	7.230899	-1.86248	Down	########	########
TRIM6-TRIM34	3487	158.5	42.5	1.320548	0.36319	-1.86234	Down	3.22E-17	1.29E-16
KIF22	2097	2294.5	619.5	31.78916	8.74995	-1.86119	Down	########	########
HIST2H2BF	999	715.5	194.5	20.8142	5.750077	-1.85591	Down	6.10E-71	5.83E-70
OAS2	3539	744	204	6.119167	1.691891	-1.8547	Down	5.82E-73	5.69E-72
TOE1	2408	735	201	8.865056	2.451404	-1.85452	Down	2.70E-72	2.62E-71
POP1	4701	1645.5	445	10.13997	2.804859	-1.85405	Down	########	########
HES1	1475	947.5	258	18.6626	5.171158	-1.85159	Down	5.45E-93	6.32E-92
TMEM229B	4068	303	83	2.166101	0.601732	-1.84791	Down	1.00E-30	5.39E-30
INCENP	4146	2822.5	769.5	19.75859	5.499492	-1.84511	Down	########	########
R3HCC1	1644	1378	376	24.33099	6.777809	-1.8439	Down	########	########
MEPE	2007	274	75	3.960144	1.103652	-1.84327	Down	5.89E-28	3.01E-27
TKT	2179	32870.5	8996	438.3272	122.1718	-1.8431	Down	0	0
BIRC5	2537	3834.5	1048	43.8993	12.25011	-1.8414	Down	0	0
SNN	3307	625	171.5	5.494403	1.537811	-1.83708	Down	1.04E-61	8.96E-61
SAMD13	1574	28.5	8	0.531104	0.148822	-1.8354	Down	0.000753	0.001565

ALDH3A1	1789	804.5	221.5	13.08835	3.673433	-1.83308	Down	1.68E-78	1.72E-77
DIO2	6284	12650.5	3474.5	58.39678	16.39369	-1.83275	Down	0	0
HAND2	2368	56.5	15.5	0.694335	0.195061	-1.8317	Down	6.54E-07	1.63E-06
FBXO43	3305	55.5	15.5	0.490424	0.13781	-1.83135	Down	1.04E-06	2.57E-06
HOXD13	2341	35.5	10	0.441568	0.124161	-1.83043	Down	0.000156	0.000342
RNF157	4915	1113	307.5	6.585727	1.8519	-1.83034	Down	########	########
CHCHD10	749	20	5.5	0.768579	0.216207	-1.82978	Down	0.002494	0.004969
SLC25A35	2785	97.5	27	1.01751	0.28657	-1.82808	Down	1.18E-10	3.44E-10
C20orf27	1376	2152.5	598	45.58625	12.85372	-1.82641	Down	########	########
ABCC2	5051	504.5	140.5	2.889818	0.815156	-1.82583	Down	2.53E-49	1.86E-48
PREX1	6633	2854	792	12.51225	3.534201	-1.82389	Down	########	########
NPTX1	5437	411	114	2.194339	0.619868	-1.82375	Down	1.37E-40	8.84E-40
PRKCDBP	1039	147.5	41	4.129659	1.167506	-1.82259	Down	2.09E-15	7.96E-15
FAIM	1538	491	136	9.238743	2.614332	-1.82125	Down	3.10E-48	2.24E-47
TOP2A	5753	11562	3191.5	58.17934	16.46676	-1.82095	Down	0	0
RFC4	1486	985.5	273	19.21841	5.439765	-1.82087	Down	5.99E-95	7.08E-94
ARAP3	5281	1094.5	304.5	6.014321	1.704955	-1.81867	Down	########	########
RBM47	5160	1026.5	286	5.780487	1.639297	-1.81811	Down	3.12E-98	3.77E-97
USP41	9282	43	12	0.133806	0.038009	-1.81572	Down	2.09E-05	4.82E-05
RTN4IP1	2636	245.5	69	2.706318	0.769578	-1.81419	Down	1.86E-24	8.82E-24
SOX5	4333	160.5	45	1.079312	0.306984	-1.81388	Down	1.65E-16	6.52E-16
NRM	1433	660	185	13.41693	3.818358	-1.81303	Down	1.85E-63	1.61E-62
HMBS	1526	1572.5	440	29.95259	8.530879	-1.81191	Down	########	########
E2F5	1594	240.5	67	4.365539	1.246241	-1.80858	Down	3.35E-24	1.58E-23
AP1AR	2812	903.5	252	9.319678	2.666281	-1.80545	Down	1.43E-86	1.59E-85

ARHGEF6	5282	1680	471.5	9.226105	2.641307	-1.80447	Down	########	########
BUB1	3509	2442	685.5	20.18061	5.791795	-1.80089	Down	########	########
NQO1	2499	21409.5	6009.5	248.6054	71.35091	-1.80085	Down	0	0
BAG2	2068	1604.5	452	22.5322	6.473937	-1.79927	Down	########	########
NASP	2284	5961.5	1675	75.70735	21.76975	-1.79811	Down	0	0
TOR3A	2138	1952	549.5	26.44077	7.610753	-1.79665	Down	########	########
TTF2	4860	1439.5	406.5	8.58784	2.476167	-1.79419	Down	########	########
PRRT1	1931	24.5	7	0.368537	0.10633	-1.79326	Down	0.002102	0.004219
KIF23	3697	2741	773	21.46419	6.193624	-1.79308	Down	########	########
FAM111B	3407	887	255	7.59121	2.192915	-1.79148	Down	2.05E-82	2.18E-81
IL23R	2826	53	16	0.566714	0.163753	-1.7911	Down	5.85E-06	1.39E-05
LYPD6	4101	231.5	65.5	1.634145	0.472246	-1.79092	Down	3.63E-23	1.67E-22
KLRC2	1223	394.5	111	9.355036	2.704652	-1.7903	Down	2.33E-38	1.44E-37
STXBP5L	9382	452	129	1.401991	0.406227	-1.78712	Down	3.57E-43	2.40E-42
DRD1	3373	25	7	0.214442	0.062146	-1.78685	Down	0.001319	0.002692
IRF2BPL	4166	4565	1304.5	31.87226	9.269514	-1.78174	Down	0	0
RPP40	1174	455.5	130	11.23934	3.273432	-1.77968	Down	2.13E-43	1.44E-42
HIST1H4K	354	42	12	3.449001	1.004702	-1.77941	Down	3.31E-05	7.54E-05
TCF7	2839	1023	294	10.47306	3.058975	-1.77556	Down	8.29E-95	9.76E-94
C18orf34	3398	111	32	0.951302	0.278274	-1.7734	Down	1.33E-11	4.10E-11
C12orf48	3081	743.5	213	7.00006	2.049256	-1.77227	Down	1.55E-69	1.46E-68
PKN3	3385	327	94.5	2.810627	0.823097	-1.77176	Down	1.91E-31	1.04E-30
PIF1	2659	464.5	134	5.079336	1.488255	-1.77102	Down	1.01E-43	6.84E-43
GINS2	1196	1201	346	29.14755	8.563034	-1.76718	Down	########	########
FBXO24	2292	58	17.5	0.751652	0.221146	-1.76507	Down	1.40E-06	3.42E-06

LBR	3837	3004	862.5	22.69931	6.67847	-1.76506	Down	########	########
LYPD1	2683	3072	886.5	33.2734	9.794499	-1.76433	Down	########	########
FBXO30	4420	1580	455.5	10.37264	3.0548	-1.76363	Down	########	########
PRR11	6487	5478	1583	24.51527	7.230531	-1.76151	Down	0	0
TYW3	3353	1600	462	13.83966	4.084042	-1.76074	Down	########	########
RGS18	2158	27	8	0.364245	0.107885	-1.75542	Down	0.001193	0.002444
FCHO1	3235	26	8	0.240734	0.071525	-1.75092	Down	0.001878	0.003782
PGD	1937	5547.5	1616.5	83.10631	24.71374	-1.74964	Down	0	0
C6orf168	9773	166	49	0.495887	0.147651	-1.74782	Down	2.92E-16	1.15E-15
MCCC2	3696	3899.5	1140.5	30.60756	9.137585	-1.744	Down	0	0
NUP88	2518	2008.5	588.5	23.13264	6.916699	-1.74177	Down	########	########
RTP4	1027	182	55	5.205302	1.558697	-1.73964	Down	2.97E-17	1.19E-16
CMTM1	786	124.5	37	4.606813	1.383367	-1.73558	Down	2.29E-12	7.55E-12
C11orf71	680	49.5	14.5	2.115292	0.635686	-1.73447	Down	7.07E-06	1.68E-05
FAM46B	2391	49.5	14.5	0.603508	0.181388	-1.7343	Down	7.07E-06	1.68E-05
PAX6	6883	135.5	40	0.568029	0.171203	-1.73026	Down	1.94E-13	6.91E-13
GABRQ	2000	168	50	2.444188	0.737389	-1.72886	Down	2.68E-16	1.05E-15
PLAU	2395	13681	4049	165.7633	50.05115	-1.72765	Down	0	0
STOX2	4654	132	40	0.830918	0.251046	-1.72675	Down	7.21E-13	2.47E-12
BRIX1	1303	2338	689.5	51.95159	15.71146	-1.72535	Down	########	########
TIMELESS	5154	2994	890	16.85614	5.109997	-1.72188	Down	########	########
IFIT1	1876	515	155	8.023772	2.434709	-1.72053	Down	3.38E-46	2.36E-45
ATIC	2094	3161	943.5	43.75027	13.34842	-1.71262	Down	########	########
SPC24	654	546.5	163.5	24.31549	7.423354	-1.71173	Down	2.52E-49	1.85E-48
ARHGAP11A	5616	4473	1335	23.07824	7.05317	-1.71019	Down	0	0

HOXA11	2653	40	12	0.436569	0.133522	-1.70913	Down	8.17E-05	0.000182
RFC3	2396	1724.5	515.5	20.8308	6.377221	-1.70772	Down	########	########
SOX12	4645	1113.5	336	6.974753	2.13639	-1.70697	Down	1.83E-97	2.19E-96
CHAC2	1305	180	54	3.996487	1.224235	-1.70685	Down	3.26E-17	1.31E-16
SLC5A6	3272	1577.5	475	13.97272	4.30334	-1.69908	Down	########	########
BFSP1	2215	656.5	200	8.627707	2.662608	-1.69614	Down	1.59E-57	1.30E-56
GEMIN6	705	516	156.5	21.26548	6.564649	-1.69572	Down	4.75E-46	3.31E-45
TUBG1	1968	2899.5	880	42.80495	13.23234	-1.69371	Down	########	########
FAM78A	3957	54.5	16.5	0.40169	0.124221	-1.69317	Down	3.75E-06	9.02E-06
TSPAN15	1726	1014.5	309	17.11563	5.299067	-1.6915	Down	5.34E-88	5.96E-87
PFAS	5400	1713.5	519.5	9.180935	2.848105	-1.68864	Down	########	########
HS3ST1	1965	69.5	21	1.024969	0.318206	-1.68755	Down	2.51E-07	6.38E-07
SUOX	2564	595.5	180	6.723697	2.087699	-1.68734	Down	8.40E-53	6.43E-52
KYNU	1774	3088	937	50.44779	15.66682	-1.68708	Down	########	########
KLHL23	4089	225.5	68.5	1.599652	0.496953	-1.68658	Down	5.59E-21	2.44E-20
PTGER2	2494	239.5	73.5	2.793691	0.869026	-1.6847	Down	6.20E-22	2.77E-21
SLC20A1	3390	21558	6562	184.3524	57.36572	-1.68421	Down	0	0
KIF11	5101	4404.5	1339.5	25.03799	7.792602	-1.68394	Down	0	0
TBXAS1	2385	896	274	10.91245	3.39693	-1.68367	Down	1.19E-77	1.21E-76
ST6GALNAC4	1717	956	293	16.21055	5.047734	-1.68323	Down	1.62E-82	1.73E-81
DLX1	2203	810.5	247	10.6709	3.323411	-1.68295	Down	1.33E-70	1.27E-69
TIMM10	684	810	248	34.42015	10.73364	-1.68111	Down	2.85E-70	2.70E-69
RUNX3	4340	323.5	99	2.164882	0.675595	-1.68006	Down	5.50E-29	2.87E-28
GABRG2	4077	104	32	0.737046	0.230876	-1.67464	Down	2.88E-10	8.23E-10
TSGA14	3526	1201.5	369.5	9.886744	3.102983	-1.67184	Down	########	########

FAM133A	3292	107	33	0.945217	0.29689	-1.67072	Down	1.69E-10	4.88E-10
CACYBP	2875	5011.5	1542.5	50.522	15.91313	-1.66669	Down	0	0
HAUS1	1145	440	137	11.1891	3.534412	-1.66255	Down	3.34E-38	2.06E-37
FAM65C	4292	62	20	0.426349	0.13511	-1.6579	Down	2.43E-06	5.89E-06
C11orf48	1357	1052.5	326	22.49166	7.127661	-1.65789	Down	1.89E-89	2.14E-88
GLDN	5084	250.5	78	1.430884	0.45388	-1.65653	Down	2.46E-22	1.11E-21
GSG2	2797	337	105	3.502963	1.111364	-1.65624	Down	1.29E-29	6.83E-29
FOPNL	2250	2544.5	789.5	32.78459	10.40959	-1.6551	Down	########	########
CIITA	4654	71	23	0.450882	0.143244	-1.65427	Down	4.76E-07	1.19E-06
LTV1	1835	1157	360	18.27734	5.809605	-1.65354	Down	1.02E-97	1.22E-96
PIR	1542	1418	441.5	26.67741	8.489064	-1.65194	Down	########	########
STMN1	1730	4402.5	1374	73.80355	23.5355	-1.64885	Down	0	0
EBF1	5288	941.5	294	5.162222	1.646351	-1.64872	Down	2.00E-79	2.07E-78
LIPE	3806	168	54	1.296446	0.4145	-1.64512	Down	5.49E-15	2.07E-14
TRAM1L1	2037	111	35	1.586904	0.507499	-1.64474	Down	1.37E-10	3.97E-10
CALHM2	1966	336.5	106.5	4.972429	1.593723	-1.64155	Down	4.14E-29	2.17E-28
HERC5	3525	672	211.5	5.52333	1.772129	-1.64005	Down	6.87E-57	5.55E-56
B3GALNT1	3292	1929	607	17.0031	5.455623	-1.63998	Down	########	########
GSTA4	1352	243.5	77	5.222482	1.675826	-1.63986	Down	2.26E-21	9.96E-21
BUB1B	3749	2190.5	685.5	16.91207	5.428277	-1.63949	Down	########	########
RAB26	1641	222.5	70.5	3.943313	1.266565	-1.63849	Down	9.14E-20	3.89E-19
HOXC8	2290	987	312	12.5469	4.036235	-1.63625	Down	4.68E-82	4.96E-81
POP5	660	635.5	200.5	27.88237	8.972968	-1.6357	Down	1.18E-53	9.14E-53
NEIL3	2402	440.5	139	5.311317	1.711271	-1.634	Down	1.47E-37	8.99E-37
LSM2	907	1187	373.5	37.94192	12.23157	-1.63318	Down	5.05E-99	6.12E-98

SLC2A12	5599	319	102	1.667118	0.537899	-1.63195	Down	2.78E-27	1.40E-26
LIMD2	3232	806	256	7.247064	2.341197	-1.63015	Down	5.97E-67	5.47E-66
CDC25C	1896	403.5	128	6.192961	2.00168	-1.62942	Down	2.56E-34	1.48E-33
UBE2C	520	3305.5	1050	184.768	59.78955	-1.62775	Down	########	########
FAH	1810	1069.5	341	17.18881	5.562906	-1.62756	Down	9.57E-88	1.07E-86
BAHCC1	10342	1561.5	498.5	4.398014	1.423543	-1.62737	Down	########	########
RNF26	2803	2842.5	904.5	29.48984	9.551981	-1.62635	Down	########	########
CDC7	3316	876	278	7.657423	2.485867	-1.62311	Down	1.10E-72	1.07E-71
GOLGA6L3	1361	88.5	28	1.880612	0.610812	-1.6224	Down	1.37E-08	3.66E-08
MARS2	3102	357	114	3.348927	1.088775	-1.62099	Down	2.16E-30	1.16E-29
FAM83D	2445	2187.5	699.5	26.02195	8.4635	-1.6204	Down	########	########
ISOC1	1942	672.5	215	10.06234	3.274315	-1.6197	Down	1.29E-55	1.02E-54
MIIP	1588	158.5	51	2.90333	0.945111	-1.61915	Down	4.11E-14	1.51E-13
CYP4F11	3395	24.5	8	0.209954	0.068365	-1.61874	Down	0.004551	0.008848
PDE1A	2009	172	55	2.491118	0.812482	-1.61639	Down	2.12E-15	8.09E-15
KIFC1	2721	1947.5	623.5	20.77495	6.776395	-1.61626	Down	########	########
ZNF174	2498	334	107	3.889178	1.269265	-1.61547	Down	1.99E-28	1.03E-27
MEIS1	3198	503	161	4.554023	1.486982	-1.61475	Down	4.92E-42	3.25E-41
ADAT3	1597	169	54	3.070555	1.003081	-1.61406	Down	3.60E-15	1.37E-14
FAM26E	3556	703	224.5	5.730539	1.872078	-1.61403	Down	2.16E-58	1.79E-57
RUVBL1	1785	2208.5	708	35.92958	11.74222	-1.61347	Down	########	########
FGF7	3936	2468	787.5	18.14805	5.934272	-1.61267	Down	########	########
RHOJ	1905	275	88.5	4.188648	1.372219	-1.60997	Down	1.03E-23	4.82E-23
RANBP3L	2635	269.5	87	2.979523	0.976138	-1.60992	Down	6.13E-23	2.81E-22
SSX2B	1306	444.5	143	9.890137	3.24199	-1.60911	Down	5.09E-37	3.09E-36

SQRDL	1682	2139.5	688	36.84652	12.108	-1.60557	Down	########	########
APOBEC3B	1536	827.5	265.5	15.61597	5.136842	-1.60407	Down	6.52E-68	6.03E-67
LRR1	1760	417.5	134	6.877321	2.263089	-1.60355	Down	5.79E-35	3.39E-34
MIS18BP1	4792	802.5	260.5	4.871033	1.603062	-1.6034	Down	5.68E-65	5.07E-64
KIF14	7293	2447	787	9.727552	3.201699	-1.60324	Down	########	########
XAF1	3581	1005	324	8.128035	2.675639	-1.60302	Down	1.58E-81	1.67E-80
ALKBH2	953	299	96.5	9.108605	2.999312	-1.6026	Down	1.52E-25	7.36E-25
CRYZ	2317	919	296	11.50296	3.788856	-1.60217	Down	8.60E-75	8.54E-74
LRRCC1	3876	500	162	3.751204	1.237292	-1.60017	Down	3.52E-41	2.29E-40
C5orf56	822	408	132.5	14.44154	4.767513	-1.59892	Down	5.78E-34	3.33E-33
TIGD1	2448	157.5	51	1.863526	0.615426	-1.59838	Down	6.24E-14	2.27E-13
LGALS9	1835	258	83.5	4.080994	1.348097	-1.598	Down	3.34E-22	1.50E-21
SIX1	2687	165.5	53.5	1.792008	0.592124	-1.5976	Down	9.33E-15	3.49E-14
ZWINT	1687	3084	997	52.97778	17.51362	-1.59691	Down	########	########
SOX15	1394	47.5	15.5	0.997551	0.329812	-1.59675	Down	3.76E-05	8.56E-05
CCT5	3403	14258	4608.5	121.441	40.15502	-1.5966	Down	0	0
HMGB1	3428	25443.5	8225	215.1317	71.20029	-1.59526	Down	0	0
CHEK1	2748	1390	451	14.64629	4.853084	-1.59356	Down	########	########
PER3	6203	452.5	149.5	2.133667	0.707002	-1.59355	Down	1.34E-36	8.10E-36
GCLM	3074	4606	1492	43.42618	14.40526	-1.59197	Down	0	0
KIAA0930	6215	8224.5	2676	38.40102	12.73854	-1.59195	Down	0	0
UGT1A4	2374	273	89.5	3.350195	1.112105	-1.59095	Down	4.89E-23	2.25E-22
ELAC2	3694	3151	1025	24.78766	8.230644	-1.59054	Down	########	########
KAT2A	3127	1889.5	615	17.51565	5.820924	-1.58932	Down	########	########
CENPH	1405	1075	349.5	22.21044	7.381163	-1.58932	Down	2.53E-86	2.79E-85

CYP26B1	4534	44	14.5	0.283628	0.094392	-1.58727	Down	6.53E-05	0.000147
C1QBP	1163	5078	1653.5	126.5628	42.14971	-1.58626	Down	0	0
HR	5528	181	59	0.950996	0.31672	-1.58623	Down	8.88E-16	3.43E-15
SPAG5	3824	4064.5	1328.5	30.84638	10.28624	-1.58439	Down	0	0
TMEM177	1759	235.5	77	3.881232	1.294582	-1.58403	Down	6.37E-20	2.73E-19
IFI35	1260	1360	444.5	31.33541	10.45218	-1.58399	Down	########	########
FIGN	4535	415.5	138	2.68658	0.896539	-1.58333	Down	2.27E-33	1.29E-32
UGT2B7	1899	31.5	11	0.50124	0.167537	-1.58102	Down	0.001914	0.003851
SSX1	1271	36.5	12	0.83708	0.27983	-1.58081	Down	0.00047	0.000994
RRS1	1724	2004	657	33.74626	11.29046	-1.57962	Down	########	########
ANGPTL1	3555	1026	336.5	8.359526	2.797107	-1.57949	Down	1.42E-81	1.50E-80
PLEKHA4	2808	1435	471.5	14.86908	4.977108	-1.57894	Down	########	########
CRTAM	2422	24	8	0.287113	0.096125	-1.57864	Down	0.004551	0.008848
MON1A	2244	563	185.5	7.284243	2.439075	-1.57844	Down	2.34E-45	1.62E-44
LONRF2	13920	108.5	35.5	0.22663	0.076076	-1.57483	Down	4.86E-10	1.38E-09
PPM1L	3056	234.5	78	2.233869	0.749927	-1.57472	Down	1.94E-19	8.20E-19
METTL12	1382	57.5	19	1.201616	0.403853	-1.57307	Down	9.78E-06	2.29E-05
CKS1B	834	2447.5	803.5	85.07208	28.59555	-1.57289	Down	########	########
IDH1	2339	5015.5	1651	62.20858	20.91732	-1.57242	Down	0	0
SUV39H1	2745	1043.5	343.5	11.01515	3.706409	-1.5714	Down	1.86E-82	1.99E-81
GLMN	2042	560	184	7.918281	2.667872	-1.5695	Down	3.95E-45	2.73E-44
GINS1	3289	1720	569.5	15.18564	5.122778	-1.56771	Down	########	########
SLC4A8	4010	82	28	0.600747	0.20267	-1.56763	Down	1.68E-07	4.28E-07
RFC5	2486	844.5	278.5	9.852351	3.324519	-1.56732	Down	6.24E-67	5.71E-66
FMO4	2148	106	36	1.450586	0.490073	-1.56557	Down	2.25E-09	6.21E-09

GYG2	3411	356.5	119	3.045494	1.029602	-1.56459	Down	1.14E-28	5.93E-28
CEP97	4439	460.5	153.5	3.015596	1.020145	-1.56367	Down	8.40E-37	5.08E-36
KIAA0101	1345	1855	615	40.05635	13.55652	-1.56304	Down	########	########
PHOSPHO2-KLHL23	4206	189	62.5	1.302744	0.441018	-1.56264	Down	2.73E-16	1.07E-15
H1F0	2336	496	165	6.171938	2.090116	-1.56214	Down	1.52E-39	9.58E-39
CBR3	1128	592	196.5	15.21293	5.155192	-1.5612	Down	3.70E-47	2.62E-46
MBNL3	11272	552.5	183	1.421573	0.481753	-1.56112	Down	5.18E-44	3.53E-43
TMEM53	1614	152.5	51	2.751336	0.932547	-1.56088	Down	4.93E-13	1.71E-12
PHF19	1001	2713	902.5	78.73466	26.69038	-1.56068	Down	########	########
CKAP2L	3273	1782	591.5	15.79442	5.363225	-1.55824	Down	########	########
RTEL1	5042	2370.5	791	13.64539	4.635587	-1.55759	Down	########	########
SUN3	1452	26	9	0.533184	0.181246	-1.55668	Down	0.003933	0.007698
CARD9	2147	25.5	8.5	0.346603	0.11784	-1.55646	Down	0.002935	0.005814
ECT2	3916	2971	989.5	21.99641	7.479914	-1.55617	Down	########	########
PLK4	3870	987	329.5	7.405713	2.5186	-1.55602	Down	8.74E-77	8.83E-76
MSX1	1940	601	200.5	9.007527	3.065942	-1.5548	Down	1.54E-47	1.10E-46
MUM1	4119	1327.5	443	9.351798	3.184004	-1.5544	Down	########	########
XRCC6BP1	1182	267	89	6.538455	2.22744	-1.55356	Down	5.77E-22	2.58E-21
SSX2	1322	836	277.5	18.27822	6.228204	-1.55324	Down	8.15E-66	7.34E-65
DUSP23	718	103	35	4.218174	1.437805	-1.55275	Down	3.86E-09	1.05E-08
CKAP2	3733	3716	1237.5	28.84186	9.831543	-1.55267	Down	########	########
GRK6	2952	2064.5	692	20.34264	6.939095	-1.55169	Down	########	########
C4orf46	3525	952	319	7.840911	2.674684	-1.55165	Down	1.26E-73	1.24E-72
FANCD2	5204	1537.5	513	8.570243	2.924612	-1.55109	Down	########	########
TTC35	1268	1454.5	484	33.16169	11.31771	-1.55094	Down	########	########

EEF1E1	609	1000	334	47.59293	16.26915	-1.54861	Down	1.43E-77	1.45E-76
POLR3K	824	915	306.5	32.24993	11.02937	-1.54795	Down	5.16E-71	4.94E-70
C9orf40	2335	539.5	180	6.681009	2.286008	-1.54724	Down	1.28E-42	8.53E-42
MRPS12	991	1314.5	443	38.53833	13.18851	-1.54701	Down	########	4.95E-99
PA2G4	2643	6441.5	2156.5	70.65484	24.18158	-1.54688	Down	0	0
C9orf84	4630	73	25	0.461563	0.158026	-1.54636	Down	8.51E-07	2.11E-06
CHN1	2446	765.5	258	9.082546	3.110146	-1.54611	Down	5.15E-59	4.30E-58
MRPL50	1050	570.5	190.5	15.75817	5.398097	-1.54558	Down	4.43E-45	3.06E-44
SLC12A8	3510	2297	772	19.02136	6.520252	-1.54462	Down	########	########
HSPA8	2276	68192	22879.5	868.366	297.6967	-1.54446	Down	0	0
VAV3	3115	192.5	65	1.799969	0.617315	-1.54389	Down	6.50E-16	2.53E-15
NSMCE4A	1420	435.5	147	8.918357	3.059662	-1.54341	Down	3.39E-34	1.96E-33
UBE2S	1207	6685.5	2254.5	160.9917	55.25669	-1.54277	Down	0	0
ZIC1	5231	313.5	106	1.741002	0.597856	-1.54205	Down	5.53E-25	2.65E-24
FAM125A	1216	1147	388	27.43288	9.420589	-1.54202	Down	3.05E-87	3.39E-86
NPB	634	54	18.5	2.470577	0.848482	-1.54189	Down	1.69E-05	3.92E-05
C13orf15	1126	92	31	2.364991	0.812807	-1.54085	Down	2.20E-08	5.85E-08
BOP1	2422	4127.5	1395	49.55493	17.03436	-1.54058	Down	0	0
HSPD1	2319	21935	7372	274.0603	94.22383	-1.54033	Down	0	0
MEGF9	6227	1607.5	542	7.490547	2.577807	-1.53893	Down	########	########
CKS2	627	2709	912.5	125.2575	43.21495	-1.53529	Down	########	########
LOC100130921	582	26.5	9	1.324627	0.457101	-1.535	Down	0.003933	0.007697
PSMB9	1048	4023.5	1361.5	111.501	38.49085	-1.53447	Down	########	########
C7orf31	3609	439.5	149.5	3.534249	1.221512	-1.53273	Down	2.65E-34	1.53E-33
STEAP1	1330	998.5	337.5	21.7809	7.533206	-1.53173	Down	2.53E-76	2.54E-75

SLC7A8	3145	165	57	1.537913	0.531937	-1.53165	Down	1.53E-13	5.49E-13
GINS3	2297	440	149.5	5.559018	1.923267	-1.53127	Down	1.77E-34	1.03E-33
IL22RA1	2988	77	26.5	0.751051	0.259866	-1.53114	Down	3.28E-07	8.27E-07
C14orf159	3164	761.5	258.5	6.982173	2.417986	-1.52987	Down	2.58E-58	2.13E-57
THYN1	790	631.5	215	23.20649	8.046295	-1.52813	Down	2.49E-48	1.80E-47
SAMD9L	7133	7938.5	2693.5	32.22801	11.1767	-1.52782	Down	0	0
CEP78	2719	679	231	7.250657	2.514613	-1.52778	Down	5.56E-52	4.21E-51
EXOC6	3637	640.5	216.5	5.097205	1.76834	-1.52731	Down	1.31E-49	9.68E-49
C1QTNF6	1670	1058.5	360	18.41907	6.393895	-1.52643	Down	5.43E-80	5.65E-79
ARL6IP1	2280	7933.5	2692	100.8384	35.04124	-1.52492	Down	0	0
SKP2	1600	1189	407	21.56977	7.508923	-1.52233	Down	5.88E-89	6.62E-88
PPP2R5D	2871	2604.5	890	26.33955	9.176175	-1.52127	Down	########	########
AIM1	7553	1727.5	591.5	6.641927	2.316601	-1.51959	Down	########	########
HNMT	3373	409.5	140	3.520255	1.229337	-1.5178	Down	1.01E-31	5.57E-31
HOXB13	3047	72	25	0.692947	0.242005	-1.51771	Down	1.28E-06	3.14E-06
ACAT2	1567	1437	492	26.60208	9.304005	-1.51562	Down	########	########
HNRNPA3	5808	13288.5	4548	66.32844	23.21584	-1.51452	Down	0	0
HNRNPR	2751	8145	2795	85.94366	30.11508	-1.5129	Down	0	0
TSEN54	1970	497.5	172	7.36025	2.582657	-1.5109	Down	1.20E-37	7.37E-37
NUP37	1228	863.5	295.5	20.31762	7.12949	-1.51086	Down	3.51E-65	3.14E-64
ABHD2	9159	4261.5	1464	13.49751	4.738	-1.51034	Down	0	0
FERMT3	2558	1180	407.5	13.39968	4.711634	-1.5079	Down	2.12E-87	2.36E-86
C17orf59	1932	363.5	125	5.453723	1.918729	-1.50709	Down	4.09E-28	2.09E-27
GCA	3241	305	105	2.728971	0.960438	-1.50659	Down	7.00E-24	3.28E-23
TEAD2	2220	1096.5	378.5	14.37406	5.059874	-1.50629	Down	2.57E-81	2.72E-80

WBSCR17	3298	38	14	0.348171	0.122778	-1.50375	Down	0.000802	0.001666
UNC5C	9879	547.5	190	1.609633	0.568004	-1.50276	Down	4.58E-41	2.97E-40
STOML2	1303	3030	1051	67.51087	23.85	-1.50113	Down	########	########
FHOD1	3865	6701.5	2323.5	50.36492	17.79485	-1.50096	Down	0	0
PHOSPHO1	2173	40	14	0.534059	0.188977	-1.49879	Down	0.000355	0.000757
ZNF804A	4700	751.5	262	4.654113	1.64824	-1.49758	Down	2.04E-55	1.62E-54
ANKRD35	3363	724.5	251	6.247818	2.213383	-1.4971	Down	6.09E-54	4.73E-53
UBR7	3709	1217	421	9.500468	3.3675	-1.49632	Down	1.01E-89	1.14E-88
CASP6	1394	521	181	10.82119	3.83603	-1.49617	Down	3.49E-39	2.19E-38
PRIM1	1471	450.5	156	8.867365	3.146112	-1.49494	Down	3.65E-34	2.10E-33
NUP35	1649	513	178	9.026259	3.203666	-1.4944	Down	1.14E-38	7.07E-38
WDR62	4731	1102	383.5	6.767722	2.402167	-1.49433	Down	6.65E-81	6.99E-80
FOXA2	2428	1139.5	396	13.58379	4.825144	-1.49324	Down	1.62E-83	1.73E-82
MIS18A	1587	845	294.5	15.45458	5.496227	-1.49152	Down	2.32E-62	2.00E-61
HSPE1	965	5121	1775.5	153.6802	54.66527	-1.49123	Down	0	0
PLEKHA6	7434	2795	978.5	10.92672	3.888339	-1.49064	Down	########	########
C16orf54	2686	472.5	165.5	5.115288	1.821015	-1.49007	Down	2.33E-35	1.38E-34
PLSCR1	2228	938.5	328	12.22951	4.355617	-1.48942	Down	1.47E-68	1.37E-67
CABLES1	4307	791	276	5.329274	1.898302	-1.48923	Down	2.91E-58	2.40E-57
SHCBP1	3249	4534.5	1581	40.47264	14.42979	-1.4879	Down	0	0
NOC4L	1682	913	319.5	15.73784	5.616963	-1.48638	Down	7.48E-67	6.84E-66
CACNA2D4	5343	410	143.5	2.224497	0.793947	-1.48636	Down	5.07E-31	2.74E-30
HIST1H3B	472	24.5	8.5	1.505291	0.537539	-1.4856	Down	0.004551	0.008849
HSPA1B	2551	1981.5	692	22.52976	8.047271	-1.48526	Down	########	########
AASDHPPT	2880	1532	535.5	15.39956	5.505105	-1.48405	Down	########	########

TCF12	4796	4053	1418.5	24.49313	8.757725	-1.48375	Down	########	########
NUF2	1843	1239	433.5	19.469	6.963096	-1.48338	Down	4.76E-90	5.42E-89
PTGR1	1249	5212.5	1827.5	121.1345	43.32492	-1.48334	Down	0	0
SYNM	6419	1008.5	355	4.573249	1.63659	-1.48253	Down	8.02E-73	7.83E-72
COL24A1	6500	161	58	0.727283	0.260284	-1.48243	Down	1.45E-12	4.84E-12
PRR3	3404	348.5	123	2.987145	1.069071	-1.48241	Down	4.16E-26	2.04E-25
GALM	2483	349.5	122.5	4.08051	1.460654	-1.48213	Down	1.45E-26	7.21E-26
HCFC1	8436	8094	2850	27.8832	10.00153	-1.47917	Down	0	0
ZDHHC14	2774	173.5	61	1.821297	0.653558	-1.47858	Down	9.03E-14	3.27E-13
GRWD1	2456	1687	596.5	19.99647	7.177649	-1.47816	Down	########	########
GTF2H4	1736	433	153	7.256741	2.605157	-1.47795	Down	4.17E-32	2.31E-31
CENPM	558	418.5	147.5	21.85505	7.846787	-1.47779	Down	3.00E-31	1.63E-30
MNS1	2023	318	112	4.56223	1.639481	-1.4765	Down	4.19E-24	1.97E-23
C5orf35	1398	253.5	88.5	5.242166	1.884205	-1.47621	Down	8.29E-20	3.53E-19
MED30	987	355	125	10.42095	3.747107	-1.47564	Down	9.79E-27	4.88E-26
PRDM8	3150	224	79	2.059197	0.741729	-1.47312	Down	2.14E-17	8.61E-17
USP46	8057	3521	1238.5	12.64417	4.554513	-1.4731	Down	########	########
PITX1	2383	1612.5	573.5	19.72887	7.107558	-1.47288	Down	########	########
DHX30	4133	4703	1664	33.06879	11.91596	-1.47258	Down	0	0
TMEM140	2072	1724	612	24.22039	8.734942	-1.47135	Down	########	########
PHF14	8370	2509.5	887.5	8.715493	3.143591	-1.47117	Down	########	########
TAF6L	2176	611	216	8.165257	2.947668	-1.46992	Down	1.35E-44	9.26E-44
BCL2L12	1893	821	290.5	12.56963	4.539472	-1.46935	Down	2.13E-59	1.78E-58
NCAPD3	5661	4956.5	1755.5	25.4053	9.179389	-1.46866	Down	0	0
TOX2	2433	2769.5	984	33.11786	11.98087	-1.46688	Down	########	########

MAPK10	4186	90.5	32	0.623142	0.225548	-1.46613	Down	9.74E-08	2.52E-07
SP140	854	235.5	83.5	7.976781	2.887452	-1.46601	Down	3.82E-18	1.57E-17
PSRC1	1759	1648.5	585.5	27.19081	9.843754	-1.46584	Down	########	########
CCDC28B	895	545.5	194.5	17.73933	6.423041	-1.46562	Down	1.39E-39	8.75E-39
C14orf169	2463	365.5	130	4.31134	1.561459	-1.46524	Down	5.02E-27	2.51E-26
HIBCH	1924	680.5	242	10.28153	3.724224	-1.46504	Down	5.49E-49	4.00E-48
SPR	1466	1559.5	554.5	30.89017	11.20542	-1.46295	Down	########	########
SELRC1	2114	451.5	161.5	6.236645	2.263077	-1.46248	Down	6.52E-33	3.67E-32
RAD54L	2567	820.5	292.5	9.271018	3.365925	-1.46172	Down	1.15E-58	9.55E-58
KCNN3	13080	809.5	289	1.797394	0.652782	-1.46123	Down	1.20E-57	9.83E-57
NDST3	5961	92.5	33	0.449653	0.163359	-1.46077	Down	8.46E-08	2.19E-07
ZNF367	3714	450	159	3.497824	1.270789	-1.46073	Down	2.64E-33	1.50E-32
NUP107	3158	1486	528	13.64738	4.959731	-1.46029	Down	########	########
HIST2H3A	507	31	11	1.772938	0.64446	-1.45998	Down	0.001914	0.003852
DDX39A	1558	3637.5	1297.5	67.85568	24.66767	-1.45985	Down	########	########
SLC25A28	1584	552	197	10.14065	3.686578	-1.4598	Down	6.27E-40	3.99E-39
LANCL1	4526	2837	1007.5	18.15744	6.60232	-1.45952	Down	########	########
SOCS2	2210	572.5	203.5	7.505954	2.730308	-1.45897	Down	1.23E-41	8.10E-41
LYN	3043	1086	387	10.3581	3.767953	-1.45891	Down	4.85E-77	4.91E-76
SCLY	2544	523	186	5.961408	2.168668	-1.45884	Down	4.22E-38	2.60E-37
CLSPN	8484	2029.5	722	6.943534	2.528199	-1.45756	Down	########	########
CMTM8	1185	27	9.5	0.659453	0.240328	-1.45627	Down	0.002563	0.005099
SLC4A4	7693	581	209	2.193532	0.800001	-1.45518	Down	1.77E-41	1.16E-40
CC2D2A	1682	451	162	7.785803	2.84015	-1.45488	Down	1.24E-32	6.95E-32
RAD9A	2128	442	159	6.054783	2.211182	-1.45326	Down	5.98E-32	3.30E-31

HSD17B10	936	2014	721.5	62.43896	22.81552	-1.45243	Down	########	########
CCBL2	2065	439.5	158	6.196254	2.264289	-1.45234	Down	1.01E-31	5.56E-31
ORC2	3140	859.5	308	7.94721	2.904956	-1.45193	Down	8.78E-61	7.44E-60
MND1	941	255	91	7.842309	2.866989	-1.45174	Down	2.70E-19	1.13E-18
NEK3	2345	164.5	59	2.033133	0.74357	-1.45116	Down	8.51E-13	2.90E-12
DNA2	4287	519.5	185	3.496098	1.279187	-1.45052	Down	1.05E-37	6.47E-37
EFHD1	2000	72	26	1.047673	0.383514	-1.44984	Down	2.48E-06	6.02E-06
ALDH16A1	2966	1159.5	418.5	11.3619	4.16305	-1.44849	Down	1.04E-80	1.09E-79
TMEM97	2585	1559.5	559	17.48197	6.405697	-1.44844	Down	########	########
ADAMTS17	6331	49	18.5	0.230613	0.084517	-1.44817	Down	0.000131	0.000288
QRSL1	4118	526.5	190	3.717412	1.363325	-1.44717	Down	1.71E-37	1.05E-36
LOC100509263	3032	87.5	31	0.830416	0.304923	-1.44539	Down	1.67E-07	4.27E-07
CHD4	6511	13040.5	4685	58.00552	21.30645	-1.4449	Down	0	0
FAM184A	3519	79.5	29	0.658862	0.242014	-1.44488	Down	1.08E-06	2.67E-06
ENPP2	3120	1583	571.5	14.73908	5.416738	-1.44415	Down	########	########
NAPEPLD	5363	1908.5	687	10.32395	3.796315	-1.44332	Down	########	########
DHX58	2631	193.5	70	2.131675	0.78421	-1.44268	Down	1.20E-14	4.46E-14
FRK	2864	122.5	45	1.254016	0.461443	-1.44233	Down	1.45E-09	4.02E-09
DHRS11	1608	205.5	74	3.688362	1.357738	-1.44178	Down	1.44E-15	5.54E-15
LMO4	5415	1470	529	7.860046	2.893865	-1.44154	Down	########	########
TBPL1	1340	543	196	11.74909	4.327734	-1.44087	Down	1.09E-38	6.77E-38
PAQR8	4758	299.5	109	1.83457	0.676429	-1.43943	Down	1.00E-21	4.45E-21
NCAPD2	4806	6628.5	2395	39.93195	14.75869	-1.43598	Down	0	0
CBX2	4265	1380	501	9.392868	3.473032	-1.43537	Down	7.65E-95	9.02E-94
FAM55C	8610	3516.5	1273	11.84133	4.378546	-1.43531	Down	########	########

GALT	1347	210	76.5	4.540613	1.679281	-1.43504	Down	7.40E-16	2.87E-15
BCL3	1864	2117.5	770	33.01963	12.21235	-1.43498	Down	########	########
GLCCI1	4762	642.5	233	3.910284	1.446585	-1.43462	Down	4.34E-45	3.00E-44
FAM120C	1176	713.5	257.5	17.56468	6.498591	-1.43448	Down	2.25E-50	1.67E-49
SMC3	4131	4141.5	1498	29.03196	10.7555	-1.43257	Down	########	########
KIF21B	9895	486.5	178	1.42898	0.529406	-1.43254	Down	4.27E-34	2.46E-33
HERC6	3781	722	262.5	5.548353	2.057414	-1.43123	Down	1.67E-50	1.24E-49
IGFLR1	1217	84.5	31	2.031157	0.753206	-1.43118	Down	5.44E-07	1.36E-06
AGPAT9	2534	1317	477.5	15.07607	5.591672	-1.43091	Down	6.60E-91	7.55E-90
ZFPM2	4507	187	69	1.216079	0.451054	-1.43086	Down	6.46E-14	2.35E-13
MAPRE2	3209	5456.5	1981	49.3216	18.30143	-1.43026	Down	0	0
E2F1	2722	1880.5	685	20.07032	7.450521	-1.42965	Down	########	########
FAM86C1	2106	236	86	3.246727	1.205899	-1.42888	Down	1.78E-17	7.21E-17
CSRNP3	11687	322.5	119	0.802429	0.298175	-1.42821	Down	7.73E-23	3.53E-22
LSM10	869	1497	545.5	49.95811	18.59242	-1.426	Down	########	########
FLOT1	1839	3701	1351	58.42651	21.7457	-1.42589	Down	########	########
FANCF	3309	483	177	4.248095	1.583658	-1.42356	Down	7.20E-34	4.13E-33
CDK1	1742	3449.5	1256	57.35412	21.38624	-1.42321	Down	########	########
IFIT3	2552	1555	567	17.6579	6.584525	-1.42316	Down	########	########
TIGD4	2472	27	10	0.319835	0.119319	-1.42251	Down	0.005098	0.009878
UBQLN4	3545	1945	711	15.92859	5.946076	-1.42161	Down	########	########
SARS2	2077	601	220	8.411176	3.140079	-1.42151	Down	8.36E-42	5.50E-41
CABLES2	3785	1118	410	8.588761	3.209209	-1.42023	Down	4.60E-76	4.62E-75
RCCD1	2690	647.5	237	6.98052	2.60996	-1.41931	Down	7.87E-45	5.41E-44
GTSE1	3128	1445.5	530	13.41233	5.015266	-1.41916	Down	9.45E-98	1.14E-96

SAMD11	2575	2393	880.5	27.03598	10.11289	-1.41869	Down	########	########
SRXN1	2580	6170	2261.5	69.37104	25.94971	-1.41862	Down	0	0
IPCEF1	6815	36	13.5	0.156593	0.05866	-1.41658	Down	0.000936	0.001934
EXOSC8	1427	1385.5	507	28.10854	10.53086	-1.41639	Down	4.87E-94	5.72E-93
KLHDC8B	1999	369	135.5	5.366126	2.010637	-1.41623	Down	2.58E-26	1.27E-25
LMNB2	4653	11834	4358	73.96732	27.72545	-1.41568	Down	0	0
DNAJA1	1538	12041.5	4411.5	226.9726	85.08516	-1.41554	Down	0	0
CDC45	1938	685	251	10.23361	3.839024	-1.4145	Down	2.43E-47	1.73E-46
NR2C2AP	1316	340	126	7.498318	2.822518	-1.40958	Down	6.19E-24	2.90E-23
NMI	1501	898.5	331	17.35288	6.532577	-1.40945	Down	5.39E-61	4.58E-60
ETFDH	2349	1029.5	378.5	12.68909	4.779563	-1.40864	Down	5.58E-70	5.27E-69
C14orf126	2696	499.5	185.5	5.395959	2.032805	-1.40841	Down	2.32E-34	1.35E-33
CCT4	2349	7273.5	2681	89.76219	33.83047	-1.40778	Down	0	0
RANGRF	854	771	284.5	26.18571	9.869171	-1.40778	Down	1.15E-52	8.77E-52
LOC100509860	1002	34	12.5	0.981832	0.370101	-1.40756	Down	0.001089	0.002235
FAM109A	3256	812.5	300.5	7.268771	2.740336	-1.40736	Down	3.92E-55	3.09E-54
MRPL4	1511	2867.5	1063	55.16218	20.81268	-1.40622	Down	########	########
ORC1	3177	516.5	192.5	4.727698	1.784483	-1.40563	Down	2.74E-35	1.62E-34
CDC6	3053	1422	524.5	13.48442	5.092227	-1.40492	Down	1.44E-95	1.71E-94
C9orf64	2485	151.5	56	1.75774	0.663882	-1.40472	Down	1.97E-11	5.99E-11
EPHA7	6644	82.5	31	0.360021	0.136028	-1.40418	Down	1.18E-06	2.90E-06
ZNF77	2057	111	41	1.568128	0.592497	-1.40416	Down	8.27E-09	2.23E-08
NAPRT1	1715	207	77	3.507421	1.325291	-1.4041	Down	4.40E-15	1.67E-14
GATSL1	990	51	19	1.506826	0.569546	-1.40363	Down	0.000112	0.000248
SCIN	2586	49	18.5	0.555707	0.210233	-1.40233	Down	0.000131	0.000287

RRP9	1588	1364.5	507	24.96175	9.447407	-1.40173	Down	1.42E-90	1.62E-89
PARP2	1904	769	287	11.74285	4.446922	-1.4009	Down	1.56E-51	1.17E-50
KCNAB2	4172	1614.5	600.5	11.25507	4.26238	-1.40084	Down	########	########
BEND3	6660	371.5	139.5	1.6275	0.61635	-1.40083	Down	1.43E-25	6.95E-25
ASRGL1	2347	69.5	26	0.868411	0.328947	-1.40052	Down	8.05E-06	1.90E-05
TTC5	1881	356	132.5	5.520758	2.091402	-1.4004	Down	5.73E-25	2.75E-24
TTK	3019	909	336	8.717094	3.303384	-1.3999	Down	1.82E-61	1.56E-60
ZNF454	2172	43	17	0.598231	0.227036	-1.39778	Down	0.00073	0.001519
HMGN2	1989	16176	6000.5	235.7066	89.46584	-1.39758	Down	0	0
TIPIN	1295	400	147.5	8.941097	3.394905	-1.39708	Down	3.26E-28	1.67E-27
MAMSTR	1579	40	15	0.739326	0.281105	-1.3951	Down	0.000686	0.00143
ATP5G1	663	2875	1068	125.6412	47.77725	-1.39491	Down	########	########
ASF1B	1746	1462.5	546	24.30761	9.244271	-1.39478	Down	2.90E-96	3.45E-95
H1FX	1524	2542.5	948.5	48.48326	18.44146	-1.39453	Down	########	########
WNT3	1506	69	26	1.337233	0.508839	-1.39397	Down	8.05E-06	1.90E-05
QTRT1	1352	710	265.5	15.27631	5.815291	-1.39337	Down	1.04E-47	7.47E-47
TMEM218	2104	659	246	9.10242	3.466041	-1.39296	Down	2.15E-44	1.47E-43
XRCC3	2620	712.5	267	7.917572	3.015241	-1.39278	Down	1.66E-47	1.19E-46
DDX54	4404	4039	1511.5	26.65902	10.15533	-1.39239	Down	########	########
CDT1	2742	1603.5	601.5	17.02657	6.486173	-1.39235	Down	########	########
RNASEH2B	1691	1061.5	396.5	18.23037	6.948947	-1.39148	Down	1.99E-70	1.89E-69
AGPHD1	1235	46.5	17.5	1.097796	0.418533	-1.3912	Down	0.000227	0.00049
HAUS8	1468	400.5	150	7.921934	3.020676	-1.39098	Down	2.07E-27	1.05E-26
NUP188	5689	3748	1401.5	19.11434	7.291945	-1.39028	Down	########	########
ALPK1	5389	567	215	3.07414	1.175163	-1.38732	Down	1.50E-37	9.16E-37

ABCF2	2262	6870.5	2578	88.17644	33.71813	-1.38687	Down	0	0
C10orf81	3459	283.5	107.5	2.393706	0.915638	-1.3864	Down	1.29E-19	5.47E-19
RBMXL1	5128	823	309	4.665734	1.787207	-1.3844	Down	1.53E-54	1.19E-53
NOP16	537	900	338	48.68887	18.65394	-1.38411	Down	1.85E-59	1.55E-58
PARP1	4001	9362	3509	67.82695	25.99494	-1.38363	Down	0	0
ESRRA	2221	1450.5	546.5	19.00101	7.284367	-1.3832	Down	2.62E-94	3.08E-93
TRIM34	2330	278	104.5	3.454667	1.324528	-1.38307	Down	1.37E-19	5.80E-19
SUMO3	1831	5932	2231	94.04745	36.06079	-1.38296	Down	0	0
SEMA6C	3904	50	19	0.371725	0.142596	-1.3823	Down	0.000166	0.000362
PIK3CG	5379	263	99	1.416872	0.543768	-1.38165	Down	1.88E-18	7.78E-18
RCC1	2633	2505	941.5	27.58721	10.592	-1.38103	Down	########	########
TXNRD1	3859	41326.5	15534	310.5309	119.3253	-1.37984	Down	0	0
APEH	2775	3501	1320.5	36.66519	14.09356	-1.37938	Down	########	########
SRSF2	2008	6928	2608.5	99.98238	38.43792	-1.37914	Down	0	0
FGD6	9288	892.5	336.5	2.78605	1.071138	-1.37908	Down	1.11E-58	9.22E-58
CHAF1B	2297	1436.5	541	18.13221	6.97254	-1.3788	Down	2.41E-93	2.81E-92
RBPMS2	2019	94.5	36	1.364901	0.524931	-1.3786	Down	2.56E-07	6.50E-07
MX1	2683	315.5	120	3.420349	1.315484	-1.37855	Down	2.00E-21	8.81E-21
TRMT11	1967	598.5	226.5	8.853064	3.409074	-1.3768	Down	1.04E-39	6.57E-39
TRIM14	1789	1700	642	27.60279	10.62933	-1.37676	Down	########	########
RPAIN	1679	978.5	369	16.86415	6.495474	-1.37645	Down	5.21E-64	4.59E-63
FMO3	2090	1034.5	390.5	14.38107	5.53964	-1.37631	Down	1.32E-67	1.22E-66
OSGEPL1	2339	291	110	3.60834	1.390502	-1.37573	Down	3.91E-20	1.68E-19
NUP160	5383	2529	955	13.62959	5.257015	-1.37443	Down	########	########
CIT	8708	1846.5	697.5	6.143862	2.372338	-1.37284	Down	########	########

KCND1	4720	44.5	17	0.273708	0.105688	-1.37282	Down	0.000497	0.001049
MKI67	12507	12479	4723.5	28.90782	11.16616	-1.37233	Down	0	0
WDR17	7529	179.5	68	0.691923	0.267309	-1.3721	Down	7.33E-13	2.51E-12
HRSP12	1011	294	112	8.451365	3.265009	-1.3721	Down	4.24E-20	1.82E-19
CYB5B	4286	6900.5	2611	46.70322	18.04916	-1.37159	Down	0	0
LSR	2228	72.5	28	0.958057	0.370552	-1.37044	Down	8.64E-06	2.04E-05
ZNF385D	1812	771	292	12.35469	4.780164	-1.36993	Down	1.52E-50	1.13E-49
AKR1B10	1610	1145.5	433.5	20.64929	7.989915	-1.36984	Down	2.25E-74	2.23E-73
METTL16	5758	1485	564	7.477921	2.894802	-1.36917	Down	2.79E-95	3.31E-94
PDK2	2674	608	231	6.609817	2.558805	-1.36914	Down	5.00E-40	3.18E-39
PSMA3	993	3106.5	1174.5	90.58423	35.06793	-1.36911	Down	########	########
OLFML2B	3240	346.5	132	3.112256	1.205072	-1.36884	Down	2.48E-23	1.15E-22
TOX	4131	120.5	46	0.851439	0.32969	-1.36879	Down	5.74E-09	1.56E-08
CEND1	1646	340.5	129	6.001401	2.324578	-1.36833	Down	3.86E-23	1.78E-22
FOXG1	2600	4470	1697	49.89577	19.32953	-1.36811	Down	########	########
CNTLN	5522	730.5	277	3.834129	1.48534	-1.36811	Down	8.23E-48	5.90E-47
CUTC	1378	674	255.5	14.17201	5.496717	-1.3664	Down	1.80E-44	1.23E-43
ALKBH4	2138	470	180	6.407176	2.485263	-1.36629	Down	6.03E-31	3.26E-30
MTHFD2L	2372	433	165	5.296976	2.055377	-1.36577	Down	7.50E-29	3.90E-28
MRPL9	1314	1958.5	745	43.22957	16.77487	-1.36572	Down	########	########
ANP32B	1617	5277	2000	94.53762	36.69251	-1.3654	Down	0	0
EML3	3265	1977	753.5	17.59427	6.83095	-1.36495	Down	########	########
SLC35G1	2428	260.5	99	3.1135	1.209971	-1.36356	Down	5.82E-18	2.37E-17
MELK	2501	1817.5	688.5	21.03998	8.176834	-1.36352	Down	########	########
NOP2	2661	2510.5	956	27.36284	10.63815	-1.36297	Down	########	########

CPS1	5738	906.5	345	4.580259	1.782166	-1.3618	Down	1.33E-58	1.10E-57
ZNF784	2006	116	44	1.673018	0.651528	-1.36055	Down	7.77E-09	2.10E-08
TMCO4	2989	1033.5	395.5	10.04058	3.910613	-1.36038	Down	3.81E-66	3.44E-65
ITPKB	6162	1048.5	400.5	4.943941	1.925616	-1.36034	Down	2.86E-67	2.63E-66
SFXN4	1400	460	176	9.53195	3.712715	-1.3603	Down	2.29E-30	1.23E-29
CALCRL	5006	70	27	0.408328	0.159142	-1.35941	Down	1.01E-05	2.37E-05
SLMO2	2581	3716	1415	41.72355	16.26373	-1.3592	Down	########	########
IRF2	2302	1081.5	412.5	13.61131	5.305887	-1.35914	Down	1.74E-69	1.64E-68
UBA7	3330	2724	1042.5	23.72133	9.250516	-1.35858	Down	########	########
C8orf4	1833	2194.5	837.5	34.72536	13.54215	-1.35853	Down	########	########
NPM3	904	1061	405.5	34.07061	13.30152	-1.35694	Down	4.51E-68	4.18E-67
ZNF689	2724	544	208	5.788222	2.260007	-1.35679	Down	1.20E-35	7.11E-35
NRF1	3523	738	281.5	6.069112	2.369962	-1.35662	Down	4.58E-48	3.30E-47
HYLS1	2073	77	30	1.089198	0.425472	-1.35613	Down	4.31E-06	1.03E-05
WDR12	2299	1325	504	16.67486	6.518736	-1.35501	Down	5.13E-85	5.59E-84
LRRC45	2639	854.5	328	9.397389	3.6751	-1.35448	Down	1.28E-54	1.01E-53
CDH15	2875	391.5	151	3.971168	1.55319	-1.35433	Down	1.08E-25	5.27E-25
IL15RA	1657	1029.5	393.5	18.05135	7.061198	-1.35412	Down	5.12E-66	4.62E-65
LOC100287313	1228	55.5	21	1.303093	0.509765	-1.35404	Down	8.22E-05	0.000183
DTX3L	5768	3312	1269.5	16.67206	6.522357	-1.35397	Down	########	########
MESP1	1184	39	15	0.964331	0.377304	-1.3538	Down	0.001016	0.002091
NEK2	1938	1276	488.5	19.09096	7.475095	-1.35273	Down	2.94E-81	3.10E-80
RARG	2992	2583	993.5	25.09594	9.829272	-1.3523	Down	########	########
ARHGAP19	5408	1122.5	430.5	6.009165	2.355595	-1.35107	Down	1.93E-71	1.86E-70
SLC25A5	1351	12129	4657.5	260.4788	102.1209	-1.35089	Down	0	0

MYPOP	1909	728.5	281.5	11.11069	4.356819	-1.3506	Down	1.85E-46	1.30E-45
GPATCH4	2172	2894	1117	38.79841	15.21502	-1.3505	Down	########	########
SSBP4	1751	1981	766.5	32.93646	12.92181	-1.34988	Down	########	########
NOL11	2454	1908.5	734	22.56957	8.860371	-1.34894	Down	########	########
METAP1D	1550	223.5	86	4.169149	1.637081	-1.34863	Down	2.46E-15	9.38E-15
TOMM22	1396	1700.5	653.5	35.28897	13.87328	-1.34691	Down	########	########
COMMD8	1438	841.5	324	16.96242	6.670018	-1.34658	Down	1.47E-53	1.14E-52
ECHDC1	2132	1835	705	24.91933	9.802131	-1.3461	Down	########	########
H2AFZ	951	10641	4096	324.4052	127.7004	-1.34503	Down	0	0
MPP6	2201	770	297.5	10.15742	3.998822	-1.34489	Down	4.29E-49	3.13E-48
C14orf49	3277	215	85	1.931418	0.760479	-1.34468	Down	2.62E-14	9.67E-14
MGST1	987	5693.5	2190.5	167.0428	65.77687	-1.34457	Down	0	0
NRGN	1238	997.5	386	23.47799	9.246926	-1.34426	Down	1.10E-62	9.53E-62
CHORDC1	3343	1433.5	551.5	12.4145	4.893504	-1.34309	Down	2.86E-90	3.26E-89
CEP70	2678	594	229	6.429119	2.534719	-1.3428	Down	2.74E-38	1.69E-37
E2F8	3736	379.5	146.5	2.950006	1.163086	-1.34276	Down	4.81E-25	2.31E-24
GPD2	6046	3663	1414	17.57928	6.934196	-1.34208	Down	########	########
FAM81A	3468	125	49	1.0478	0.413611	-1.34102	Down	5.24E-09	1.43E-08
NMRAL1	1401	672	261.5	13.956	5.512975	-1.33998	Down	1.32E-42	8.80E-42
RAVER2	4376	832	322.5	5.519182	2.180943	-1.3395	Down	1.25E-52	9.52E-52
TRMT1L	4421	1199	463.5	7.851804	3.10418	-1.33881	Down	2.51E-75	2.51E-74
FAM122B	2717	3503.5	1355.5	37.41826	14.79571	-1.33856	Down	########	########
RRM2	3284	8954.5	3461	79.05661	31.27473	-1.33789	Down	0	0
HOXC13	2435	290.5	112.5	3.466002	1.371549	-1.33747	Down	1.88E-19	7.92E-19
RHBDL3	4688	253.5	98	1.563502	0.61897	-1.33684	Down	4.34E-17	1.74E-16

DIMT1	1557	1393.5	541	25.98176	10.29697	-1.33528	Down	1.96E-86	2.16E-85
PSMD5	3411	2107.5	816.5	17.91855	7.102561	-1.33504	Down	########	########
ID3	1288	8553	3335	193.14	76.58856	-1.33445	Down	0	0
SPC25	1375	1136	440	23.93294	9.490629	-1.33442	Down	3.98E-71	3.82E-70
C18orf54	5023	548.5	212.5	3.160801	1.25352	-1.3343	Down	2.89E-35	1.71E-34
HMGA1	1998	12508.5	4871	181.9466	72.16953	-1.33405	Down	0	0
SLC4A11	3110	243	94	2.26365	0.897903	-1.33402	Down	1.67E-16	6.58E-16
TIMM8A	1459	427	165.5	8.491335	3.369636	-1.3334	Down	6.94E-28	3.53E-27
OR10H1	1120	41	17	1.10618	0.439009	-1.33326	Down	0.001547	0.00314
XRCC1	2102	1611	625.5	22.20441	8.812717	-1.33319	Down	########	9.42E-99
EZH2	2681	2355.5	912.5	25.46135	10.10793	-1.33282	Down	########	########
RFTN2	3131	507.5	198.5	4.713961	1.87207	-1.33231	Down	2.81E-32	1.56E-31
FGD1	4291	1631	637	11.05913	4.394033	-1.33162	Down	########	6.72E-99
PHB2	1457	5585	2177	111.344	44.24632	-1.33139	Down	0	0
DDX60	6099	1380.5	536.5	6.557129	2.607703	-1.33028	Down	1.24E-85	1.36E-84
PKIB	1909	736.5	284.5	11.11951	4.426643	-1.32881	Down	5.67E-47	4.01E-46
CHAF1A	3344	1982	773	17.19909	6.847437	-1.3287	Down	########	########
DBR1	2690	663	259.5	7.18151	2.859597	-1.32848	Down	1.11E-41	7.31E-41
PGAM5	1194	1947.5	761	47.34199	18.85556	-1.32813	Down	########	########
FECH	7295	1356.5	530	5.407697	2.154405	-1.32773	Down	2.44E-83	2.61E-82
GGCT	1217	1896	736.5	45.09878	17.96988	-1.32751	Down	########	########
ALKBH6	980	526.5	205	15.53524	6.196279	-1.32607	Down	1.56E-33	8.92E-33
SMARCD2	2770	2628	1029.5	27.5882	11.00662	-1.32568	Down	########	########
PKNOX2	3730	101	41	0.798536	0.318687	-1.32522	Down	3.52E-07	8.88E-07
APOL3	2145	1029.5	406	13.97559	5.580237	-1.32451	Down	9.94E-63	8.59E-62

PBX1	4162	2013	789.5	14.03448	5.605985	-1.32394	Down	########	########
EIF2B3	1994	2079.5	812	30.23003	12.07706	-1.32371	Down	########	########
MCM4	4800	7716.5	3017.5	46.60815	18.62948	-1.32299	Down	0	0
ZNF792	3898	264.5	104	1.966235	0.785997	-1.32284	Down	2.51E-17	1.01E-16
SEMA3A	5672	8638.5	3379.5	44.16432	17.65855	-1.32251	Down	0	0
ADAMTS3	5836	410.5	161.5	2.042316	0.816821	-1.32211	Down	3.45E-26	1.69E-25
AFP	2032	165.5	65	2.366825	0.947031	-1.32147	Down	2.27E-11	6.87E-11
SCFD2	3204	1276.5	502.5	11.57946	4.636886	-1.32034	Down	1.03E-77	1.05E-76
RSRC1	1647	1029.5	403	18.10243	7.25004	-1.32012	Down	1.78E-63	1.55E-62
HNRNPA1	1941	24284.5	9510.5	362.856	145.3336	-1.32003	Down	0	0
HSP90AB1	2567	62658.5	24550	707.5013	283.5231	-1.31927	Down	0	0
FGB	3451	53	22	0.462083	0.185212	-1.31897	Down	0.000313	0.000669
ABHD11	1446	790.5	311.5	15.88693	6.369725	-1.31854	Down	9.69E-49	7.03E-48
FARSB	2233	2505	981	32.50017	13.03624	-1.31792	Down	########	########
IRF1	3567	5103.5	2015	41.6117	16.70318	-1.31687	Down	########	########
POLD1	3470	1884.5	743.5	15.81315	6.347754	-1.31681	Down	########	########
C14orf102	3480	340	135	2.857003	1.146897	-1.31677	Down	1.29E-21	5.72E-21
NR1I3	1285	45	18	1.017128	0.408488	-1.31613	Down	0.000617	0.001293
SRSF1	5468	10677	4190.5	56.58834	22.73817	-1.31539	Down	0	0
SOX4	4912	3719.5	1464	21.9939	8.839366	-1.31509	Down	########	########
DLAT	4352	2071.5	815.5	13.80659	5.549656	-1.31489	Down	########	########
MXD3	2662	1216	481.5	13.2719	5.335341	-1.31472	Down	1.68E-73	1.65E-72
NUP85	2311	1499	591	18.82618	7.569075	-1.31455	Down	1.17E-90	1.33E-89
HNRPDL	4157	5530	2174.5	38.57418	15.51423	-1.31404	Down	0	0
CREB3L4	1747	150	59.5	2.501917	1.006373	-1.31387	Down	1.74E-10	5.03E-10

NCOA7	6457	2597	1022.5	11.66516	4.692723	-1.31371	Down	########	########
SLC12A9	3308	2288	906	20.10309	8.087414	-1.31367	Down	########	########
LPAR1	3182	1449.5	573	13.23605	5.330677	-1.31208	Down	2.84E-87	3.15E-86
RNFT2	2914	119.5	47.5	1.196664	0.482024	-1.31184	Down	1.50E-08	4.02E-08
SMAD1	3056	408.5	162	3.888666	1.567414	-1.31089	Down	1.26E-25	6.13E-25
ST8SIA1	9737	182	72.5	0.543839	0.219325	-1.31011	Down	2.54E-12	8.37E-12
TRIM16L	1982	3556.5	1406	52.09313	21.01873	-1.30942	Down	########	########
TMCO7	4834	1166.5	461.5	6.999178	2.824338	-1.30927	Down	1.31E-70	1.25E-69
RANBP1	884	2134	841.5	69.88927	28.22874	-1.30791	Down	########	########
WDR4	1524	1110.5	441	21.19284	8.560114	-1.30788	Down	8.93E-67	8.14E-66
NOP56	1992	4987	1969.5	72.64677	29.35261	-1.30741	Down	########	########
HSP90AA1	3366	42864	16938	368.8752	149.2822	-1.30509	Down	0	0
C2orf43	2876	651.5	257.5	6.561519	2.655789	-1.30489	Down	2.76E-40	1.77E-39
COASY	1840	3581	1424.5	56.57184	22.90012	-1.30473	Down	########	########
C1orf135	2061	209	83.5	2.950703	1.195408	-1.30356	Down	7.40E-14	2.69E-13
SLC25A18	2087	433	172	6.025827	2.441643	-1.30331	Down	4.43E-27	2.22E-26
RPF1	1975	1790	710	26.30646	10.66471	-1.30257	Down	########	########
RBM24	2399	311	124	3.766664	1.527197	-1.3024	Down	8.94E-20	3.81E-19
C16orf53	3843	1081	431	8.173878	3.319382	-1.30011	Down	1.05E-64	9.33E-64
ACYP1	621	158	63	7.388879	3.001061	-1.29988	Down	9.31E-11	2.73E-10
C3orf37	1809	1103.5	439	17.72376	7.202082	-1.2992	Down	3.57E-66	3.22E-65
ADCY3	4410	995.5	397.5	6.556613	2.664617	-1.29902	Down	1.25E-59	1.05E-58
MRPS27	2797	2533.5	1009.5	26.28207	10.68179	-1.29893	Down	########	########
CLIC2	2702	187	74	1.997023	0.811719	-1.2988	Down	1.29E-12	4.35E-12
FLAD1	1911	1308.5	524	19.89861	8.091033	-1.29827	Down	2.64E-77	2.68E-76

HIST1H3D	864	65.5	27	2.229711	0.907155	-1.29744	Down	6.47E-05	0.000145
THEM4	5172	484.5	193	2.727767	1.11002	-1.29713	Down	6.73E-30	3.57E-29
PSMC6	1599	2335.5	929	42.32891	17.23405	-1.29638	Down	########	########
NUFIP1	3487	677	269.5	5.630798	2.292843	-1.2962	Down	2.13E-41	1.39E-40
WDR67	3232	697	278.5	6.263825	2.551185	-1.29588	Down	2.59E-42	1.72E-41
AHSA1	1429	2253	898.5	45.73803	18.63391	-1.29546	Down	########	########
LOH12CR1	2085	222	89.5	3.106246	1.265566	-1.29539	Down	2.01E-14	7.47E-14
HEATR7B1	5110	51	21	0.293052	0.119422	-1.29509	Down	0.000371	0.00079
OSBPL11	4651	1207.5	482	7.536013	3.071104	-1.29504	Down	7.51E-72	7.27E-71
CAT	2300	2878.5	1150.5	36.30435	14.80447	-1.29411	Down	########	########
CYP4X1	2382	89	37	1.103024	0.449866	-1.2939	Down	2.87E-06	6.93E-06
CAND2	4248	65	27	0.453185	0.184843	-1.2938	Down	6.47E-05	0.000145
IFIT5	4034	1656	660	11.8989	4.855377	-1.29317	Down	3.28E-98	3.96E-97
HNRNPM	2436	11523	4610.5	137.3512	56.07326	-1.29248	Down	0	0
ABHD8	2114	429	172	5.895488	2.409104	-1.29111	Down	1.87E-26	9.24E-26
MRPL47	1222	923	368.5	21.87837	8.942671	-1.29073	Down	1.65E-55	1.31E-54
TBL3	2618	1352	544	15.00641	6.140919	-1.28905	Down	2.87E-79	2.96E-78
LOC731282	1038	108.5	43.5	3.034774	1.242429	-1.28843	Down	8.54E-08	2.21E-07
DCAF4	2468	245	98	2.878839	1.178641	-1.28836	Down	8.08E-16	3.13E-15
SMC6	5188	954	381.5	5.32104	2.178725	-1.28822	Down	3.72E-57	3.01E-56
LACTB2	1553	496	197.5	9.23793	3.784688	-1.2874	Down	8.59E-31	4.63E-30
FASTKD3	2451	513	205.5	6.056829	2.481649	-1.28726	Down	1.74E-31	9.52E-31
DTYMK	1227	2044	822.5	48.43591	19.84887	-1.28702	Down	########	########
SLC25A12	3995	1621	651.5	11.77417	4.833713	-1.28442	Down	5.70E-95	6.74E-94
TFAP4	2147	281	113.5	3.820209	1.569001	-1.28381	Down	8.87E-18	3.60E-17

BLM	4528	485.5	198	3.125695	1.284341	-1.28315	Down	7.62E-29	3.96E-28
ACTR5	2479	295	120	3.473222	1.427779	-1.2825	Down	2.94E-18	1.21E-17
DDAH2	1351	608.5	247	13.11931	5.393339	-1.28244	Down	4.91E-36	2.94E-35
MMAA	5943	362	146.5	1.76879	0.727427	-1.28189	Down	2.33E-22	1.05E-21
RIPK4	3890	492	199	3.680869	1.514197	-1.28149	Down	1.11E-29	5.85E-29
NAE1	1716	3116	1252.5	52.5684	21.62709	-1.28136	Down	########	########
QPRT	1575	140	57	2.582556	1.063095	-1.28053	Down	2.12E-09	5.86E-09
PHTF2	5025	4452	1790	25.66212	10.56926	-1.27977	Down	########	########
FAM54A	1523	126.5	50.5	2.388567	0.985822	-1.27675	Down	6.48E-09	1.76E-08
POLR1E	1854	904	365	14.12307	5.829999	-1.27649	Down	2.73E-53	2.10E-52
CENPW	567	741	299.5	37.95068	15.66644	-1.27645	Down	4.88E-44	3.33E-43
HYAL2	2413	1725.5	702	20.82163	8.599772	-1.27571	Down	1.00E-98	1.21E-97
FEN1	2308	2625.5	1062.5	33.01027	13.63425	-1.27568	Down	########	########
PDCL3	1086	973.5	391.5	25.98531	10.73311	-1.27563	Down	1.13E-57	9.24E-57
PRMT1	1473	5695.5	2310	112.4225	46.44379	-1.27537	Down	0	0
HIST1H1B	790	64	27	2.401529	0.992129	-1.27535	Down	9.26E-05	0.000206
AGFG2	4821	785.5	319	4.731846	1.956259	-1.27431	Down	5.18E-46	3.62E-45
CCDC116	2252	34	14.5	0.454178	0.187816	-1.27394	Down	0.003802	0.007461
SS18L2	822	477.5	193	16.83311	6.966801	-1.27273	Down	8.24E-29	4.28E-28
RACGAP1	3326	3938.5	1593	34.33784	14.2117	-1.27272	Down	########	########
MIA2	2351	40.5	17	0.507372	0.210054	-1.27228	Down	0.002233	0.004468
FBXO5	2269	721.5	292.5	9.215197	3.816508	-1.27176	Down	1.23E-42	8.19E-42
KCNE4	2976	3003	1218.5	29.31693	12.14387	-1.27151	Down	########	########
ALG6	3371	478	193.5	4.107103	1.701408	-1.27139	Down	5.77E-29	3.01E-28
NQO2	1139	1007	409.5	25.72233	10.66311	-1.27039	Down	1.39E-58	1.15E-57

PDE6D	1140	488	198.5	12.39575	5.143507	-1.26902	Down	2.63E-29	1.38E-28
BRD7	2330	2869	1163.5	35.69844	14.81424	-1.26888	Down	########	########
MAT2B	2090	3472	1408.5	48.14218	19.98226	-1.26858	Down	########	########
EPHX1	1847	10227	4164	160.7751	66.73652	-1.2685	Down	0	0
TMEM160	696	192	79	8.053973	3.344623	-1.26786	Down	3.53E-12	1.15E-11
AQP3	1835	91	37.5	1.451006	0.60316	-1.26644	Down	1.40E-06	3.42E-06
SCML1	2693	805.5	327.5	8.660412	3.601618	-1.26579	Down	3.59E-47	2.55E-46
KIAA1383	5455	86.5	36	0.464436	0.193237	-1.26511	Down	4.85E-06	1.16E-05
KIAA1524	4284	1402	568	9.457909	3.936871	-1.26447	Down	3.38E-81	3.56E-80
STAT5A	4314	298.5	121	1.99989	0.832474	-1.26444	Down	1.76E-18	7.28E-18
NEURL1B	6417	327	134	1.482441	0.617132	-1.26432	Down	7.72E-20	3.30E-19
B4GALT6	4814	580.5	237.5	3.503898	1.458888	-1.26409	Down	4.00E-34	2.31E-33
ELOVL4	3085	43.5	18	0.414181	0.172468	-1.26393	Down	0.001299	0.002654
CENPI	2571	621	252	6.985015	2.910088	-1.2632	Down	7.75E-37	4.69E-36
ZBTB45	2393	735	301.5	8.935969	3.723934	-1.2628	Down	1.22E-42	8.17E-42
FAR2	2235	272.5	110.5	3.523555	1.468401	-1.26279	Down	4.17E-17	1.67E-16
DENND1B	8388	958.5	390.5	3.319327	1.383444	-1.26263	Down	1.33E-55	1.06E-54
CXorf57	3628	122	51	0.985775	0.41092	-1.2624	Down	4.78E-08	1.25E-07
FAM63A	2250	628	258	8.123492	3.387118	-1.26204	Down	1.75E-36	1.05E-35
RABL3	3883	1112.5	454.5	8.315602	3.467603	-1.26188	Down	6.04E-64	5.31E-63
DLX2	2308	606.5	249.5	7.642333	3.189899	-1.2605	Down	2.97E-35	1.75E-34
TRIM16	2920	4205	1722	41.80917	17.45469	-1.2602	Down	########	########
ZMYM1	4172	612.5	250.5	4.255335	1.777113	-1.25974	Down	6.21E-36	3.70E-35
CITED1	830	61	25	2.146158	0.897044	-1.25851	Down	9.06E-05	0.000201
LYSMD2	1118	332	135	8.581322	3.587235	-1.25833	Down	2.28E-20	9.84E-20

DDX56	2499	2742	1122	31.79726	13.29829	-1.25766	Down	########	########
LOC100287837	1695	238.5	97.5	4.085325	1.709315	-1.25703	Down	5.68E-15	2.14E-14
ANLN	4786	9640.5	3943	58.35846	24.42266	-1.25672	Down	0	0
C1orf190	1863	95	39.5	1.492845	0.62476	-1.25669	Down	1.01E-06	2.48E-06
CTPS2	4334	582.5	239	3.900612	1.63262	-1.25651	Down	5.90E-34	3.39E-33
FDX1	3236	692.5	284	6.222572	2.606037	-1.25565	Down	4.24E-40	2.70E-39
NUDT12	3502	883	361	7.312102	3.063245	-1.25523	Down	5.06E-51	3.79E-50
AAMP	1859	2621	1080	40.99644	17.17685	-1.25503	Down	########	########
ALKBH8	4085	258	106	1.830392	0.76698	-1.25489	Down	6.64E-16	2.58E-15
POU6F1	4715	154	64.5	0.960432	0.40249	-1.25473	Down	6.81E-10	1.92E-09
GCSH	1538	1165	476.5	21.90881	9.187486	-1.25377	Down	8.02E-67	7.32E-66
HVCN1	1736	65	27	1.095727	0.459734	-1.25302	Down	6.47E-05	0.000145
TUBGCP4	3087	852	349	7.988308	3.352418	-1.25269	Down	3.92E-49	2.87E-48
GPR18	1484	39	17	0.789489	0.331327	-1.25266	Down	0.003202	0.006323
PDCD2L	1174	248	103	6.178024	2.593622	-1.25218	Down	4.39E-15	1.66E-14
DLX6	1892	34	14	0.522402	0.219314	-1.25216	Down	0.003802	0.007462
XRCC5	3448	12532.5	5146.5	105.2895	44.23978	-1.25095	Down	0	0
MYBPH	1806	41	18	0.686003	0.288268	-1.2508	Down	0.00267	0.005305
WDR74	1385	851.5	351	17.85207	7.503571	-1.25044	Down	1.65E-48	1.19E-47
LOC653375	805	748	308	26.95475	11.33647	-1.24957	Down	5.74E-43	3.85E-42
WSCD2	4650	37	16	0.237974	0.100135	-1.24885	Down	0.003838	0.007525
OASL	1584	60	25.5	1.121425	0.471942	-1.24865	Down	0.00013	0.000287
PCDH20	4786	167.5	71	1.029502	0.433556	-1.24765	Down	3.05E-10	8.72E-10
DHX37	4568	2140	885	13.61717	5.736375	-1.24722	Down	########	########
CPA1	1445	51	22	1.056975	0.445303	-1.24708	Down	0.000643	0.001344

C9orf46	1020	577.5	236.5	16.3919	6.906143	-1.24703	Down	6.67E-34	3.83E-33
ABCA6	5296	305.5	125.5	1.670088	0.703636	-1.24702	Down	1.33E-18	5.52E-18
NLRC5	6822	1037	429.5	4.423611	1.863844	-1.24694	Down	1.98E-58	1.64E-57
CCDC61	1825	165.5	69	2.644713	1.114703	-1.24645	Down	2.11E-10	6.09E-10
P2RX6	2754	483.5	200.5	5.086347	2.144147	-1.24623	Down	4.55E-28	2.33E-27
ABHD15	3365	617	255	5.318654	2.243045	-1.2456	Down	1.62E-35	9.63E-35
HIST2H2BE	2223	489	202	6.379163	2.691927	-1.24473	Down	1.65E-28	8.49E-28
MRPL24	894	1789	738.5	57.97773	24.47101	-1.24443	Down	########	6.97E-99
MCM2	3453	3590	1486	30.1677	12.73803	-1.24386	Down	########	########
CDH24	3453	329.5	136.5	2.763863	1.167393	-1.2434	Down	1.13E-19	4.81E-19
FANCG	2649	1055.5	438	11.57116	4.887923	-1.24324	Down	4.72E-59	3.95E-58
TNFAIP8L1	3817	675	280	5.137472	2.171365	-1.24246	Down	1.86E-38	1.15E-37
DOCK10	7431	2155	893.5	8.407993	3.55493	-1.24194	Down	########	########
HMGB2	1454	3755	1552.5	74.96487	31.70242	-1.24162	Down	########	########
CSE1L	3579	11579	4785.5	93.75714	39.65116	-1.24157	Down	0	0
IRF3	1245	1661.5	690	38.79016	16.40499	-1.24156	Down	7.83E-92	9.01E-91
GBGT1	1949	59	25	0.884131	0.373935	-1.24147	Down	0.000187	0.000406
EML5	7171	62.5	27	0.259366	0.109698	-1.24145	Down	0.000188	0.000408
TOMM40L	2713	783	328	8.430551	3.565887	-1.24137	Down	1.34E-43	9.05E-43
RBMX	2097	7511	3110	103.8631	43.97199	-1.24003	Down	0	0
MID1IP1	2454	1811	755	21.46531	9.089502	-1.23973	Down	2.46E-99	2.99E-98
HMG20B	1584	1892	786.5	34.69008	14.69362	-1.23933	Down	########	########
C18orf45	2994	401	166.5	3.888132	1.647168	-1.23909	Down	1.39E-23	6.47E-23
MRPS28	724	607	252.5	24.35016	10.31742	-1.23885	Down	1.06E-34	6.15E-34
TMEM121	1538	82	34	1.548412	0.656143	-1.23871	Down	6.78E-06	1.61E-05

ANKRD44	1638	304.5	126.5	5.404417	2.290476	-1.23849	Down	3.25E-18	1.33E-17
MRPL52	1120	1278	532	33.17932	14.06624	-1.23805	Down	7.94E-71	7.58E-70
ETAA1	3298	810.5	337	7.148136	3.030528	-1.238	Down	1.41E-45	9.79E-45
CCNE2	2739	674	280	7.135011	3.027787	-1.23665	Down	2.63E-38	1.63E-37
CYP2S1	2657	810.5	337.5	8.85621	3.758734	-1.23644	Down	1.41E-45	9.79E-45
PSME2	829	4754	1975	166.3616	70.61089	-1.23636	Down	########	########
APOL6	10156	2981	1237	8.509089	3.611682	-1.23633	Down	########	########
TLR1	2867	183.5	77	1.866415	0.792771	-1.23529	Down	2.83E-11	8.50E-11
SNRPD1	1614	2756.5	1144.5	49.48839	21.02169	-1.23521	Down	########	########
ZNF642	2176	143	60	1.921474	0.817243	-1.23338	Down	3.77E-09	1.03E-08
COPS4	1765	1550	644.5	25.41981	10.81524	-1.23289	Down	8.55E-86	9.39E-85
MRPL34	968	1526	638.5	45.83583	19.517	-1.23174	Down	1.41E-83	1.51E-82
PRICKLE3	1990	471.5	198	6.90416	2.940703	-1.2313	Down	1.01E-26	5.01E-26
ALG14	1057	528	220.5	14.50721	6.179859	-1.23112	Down	3.25E-30	1.73E-29
BEND6	2727	148.5	62	1.582818	0.674904	-1.22974	Down	1.91E-09	5.29E-09
HADH	2037	1140.5	476	16.25446	6.9308	-1.22974	Down	4.86E-63	4.21E-62
PFKM	3088	3117.5	1301.5	29.28302	12.48963	-1.22933	Down	########	########
RPP25	2333	62	25.5	0.764676	0.326256	-1.22885	Down	6.27E-05	0.000141
GK	4590	527.5	221	3.336726	1.424085	-1.2284	Down	7.82E-30	4.14E-29
PFDN4	1383	1015	423	21.25528	9.072443	-1.22826	Down	1.65E-56	1.33E-55
ATP5G3	3305	6834	2857.5	60.01856	25.62137	-1.22806	Down	0	0
TIGD2	2101	587.5	246.5	8.143323	3.476511	-1.22798	Down	4.42E-33	2.50E-32
TAF5	3283	357.5	150	3.156661	1.348303	-1.22725	Down	1.19E-20	5.15E-20
L3MBTL2	3205	1163	488.5	10.5376	4.504409	-1.22614	Down	1.00E-63	8.79E-63
WDR46	2201	1991.5	834	26.25909	11.22578	-1.226	Down	########	########

TTC27	2953	803.5	336	7.886368	3.371641	-1.22591	Down	9.32E-45	6.40E-44
MRPL18	994	1317	550.5	38.38951	16.42071	-1.2252	Down	1.59E-72	1.55E-71
BTN3A3	2927	1312.5	552	13.03441	5.577305	-1.22468	Down	2.55E-71	2.45E-70
PKMYT1	2187	1249.5	526.5	16.62572	7.114459	-1.22459	Down	7.23E-68	6.68E-67
NDRG4	3581	548.5	230.5	4.445771	1.902472	-1.22456	Down	6.51E-31	3.52E-30
ECSIT	1725	1040	436.5	17.51305	7.495522	-1.22433	Down	2.91E-57	2.37E-56
ABCC4	3077	6429.5	2696	60.61538	25.95854	-1.22347	Down	0	0
ST20-MTHFS	2282	94.5	39.5	1.198546	0.513498	-1.22285	Down	1.44E-06	3.53E-06
HAT1	1682	1904	796	32.75077	14.03406	-1.2226	Down	########	########
SMC4	5297	7833.5	3272.5	42.7955	18.34971	-1.2217	Down	0	0
TTC23	3440	705	297	5.950686	2.552056	-1.2214	Down	4.83E-39	3.03E-38
NUP205	6286	7337.5	3075.5	33.80211	14.49898	-1.22116	Down	0	0
HSPA14	1810	692	290.5	11.0691	4.748822	-1.2209	Down	1.06E-38	6.59E-38
KIAA0889	3728	2374	1003	18.56396	7.966465	-1.22049	Down	########	########
RBM42	1669	3032.5	1279.5	52.84841	22.68644	-1.22003	Down	########	########
DNAJC9	1450	1879.5	790.5	37.56421	16.12982	-1.21963	Down	########	########
HTATIP2	782	1161	486.5	43.01534	18.4714	-1.21956	Down	6.95E-64	6.11E-63
NDC80	2209	1642.5	688	21.54262	9.256663	-1.21863	Down	1.91E-89	2.16E-88
SETDB2	5636	808.5	339.5	4.15747	1.788734	-1.21677	Down	8.13E-45	5.59E-44
UTP20	9029	1755	738	5.621624	2.419079	-1.21653	Down	7.25E-95	8.55E-94
SNAI1	1722	769	325	12.94065	5.569307	-1.21634	Down	3.39E-42	2.24E-41
NOL7	896	1897	798	61.45604	26.45688	-1.21591	Down	########	########
GSDMD	1772	1682.5	712	27.60738	11.88719	-1.21565	Down	6.24E-90	7.09E-89
CCT6A	2682	21921	9228.5	236.9279	102.0251	-1.21552	Down	0	0
ATE1	2224	436.5	185	5.699607	2.454828	-1.21524	Down	1.82E-24	8.66E-24

TRABD	2315	1187	503	14.91097	6.42254	-1.21516	Down	6.99E-64	6.14E-63
HDGF	2397	14608	6160.5	176.8664	76.18345	-1.21511	Down	0	0
HINT3	3396	868.5	366.5	7.419086	3.196216	-1.21488	Down	1.34E-47	9.58E-47
PARP12	3805	1913	808	14.59177	6.287822	-1.21452	Down	########	########
IDH3A	2701	2410.5	1018	25.9217	11.17154	-1.21433	Down	########	########
AHCYL1	4044	8687	3660.5	62.2419	26.838	-1.21361	Down	0	0
PCGF6	2023	633.5	267	9.079176	3.91638	-1.21304	Down	3.77E-35	2.22E-34
FAM164A	3352	446.5	189	3.860452	1.666244	-1.21217	Down	4.80E-25	2.31E-24
DGKI	4780	148	63.5	0.905361	0.390816	-1.212	Down	3.25E-09	8.91E-09
CWC27	2093	1229	519	17.05889	7.364537	-1.21186	Down	1.74E-66	1.58E-65
ERCC6L	4224	1021	441.5	7.069855	3.052222	-1.21182	Down	2.46E-53	1.90E-52
AIFM1	2289	2522	1067.5	31.99866	13.81654	-1.21161	Down	########	########
CAP2	3080	1811	766	17.04705	7.360725	-1.2116	Down	8.03E-97	9.58E-96
RNFT1	2031	581	246	8.310896	3.589916	-1.21105	Down	3.47E-32	1.93E-31
DHX57	4845	966	411.5	5.80053	2.505999	-1.2108	Down	6.04E-52	4.57E-51
DNAJC12	1218	392	166	9.333301	4.032376	-1.21076	Down	3.14E-22	1.41E-21
NCAPG	4682	2459	1037.5	15.22092	6.576528	-1.21066	Down	########	########
KIF18A	3463	1303	550	10.89595	4.708305	-1.21051	Down	1.95E-70	1.85E-69
CISH	2128	88	37.5	1.197835	0.517758	-1.21008	Down	4.10E-06	9.82E-06
KIF4A	4504	2831	1196.5	18.22957	7.879878	-1.21004	Down	########	########
TRIM59	3886	388	165.5	2.901936	1.255185	-1.20912	Down	7.33E-22	3.27E-21
PTCD2	2103	404	171	5.55401	2.402846	-1.20879	Down	7.01E-23	3.21E-22
BIN1	2333	2268.5	963.5	28.25686	12.22523	-1.20874	Down	########	########
HNRNPA2B1	3666	43020	18193.5	340.1526	147.2915	-1.20751	Down	0	0
NDRG2	2075	99.5	43	1.405303	0.609025	-1.20631	Down	2.10E-06	5.10E-06

UBE2L6	1283	3665	1557	82.95643	35.95166	-1.2063	Down	########	########
PRR16	1798	45.5	20	0.757025	0.328093	-1.20624	Down	0.001858	0.003742
TTC38	2616	516	220	5.7213	2.479691	-1.20618	Down	2.01E-28	1.03E-27
NOXO1	1587	67	28.5	1.224392	0.530685	-1.20614	Down	5.46E-05	0.000123
DOLPP1	2225	964	409.5	12.55673	5.446327	-1.2051	Down	4.23E-52	3.20E-51
CCDC8	3336	386.5	164.5	3.365066	1.459677	-1.20499	Down	8.63E-22	3.84E-21
TXNRD3NB	1165	38	17	0.975789	0.42328	-1.20496	Down	0.004562	0.008865
KRT4	2147	92.5	40.5	1.268206	0.550256	-1.20461	Down	4.95E-06	1.18E-05
MRPS7	1432	1612	686.5	32.67855	14.18203	-1.20428	Down	1.90E-85	2.08E-84
CCT3	2107	11890	5055.5	163.6611	71.06176	-1.20357	Down	0	0
METTL4	3691	597.5	254.5	4.694225	2.039657	-1.20256	Down	9.47E-33	5.32E-32
LIPH	2478	51	22	0.601537	0.261403	-1.20238	Down	0.000643	0.001344
COX10	3016	713.5	304.5	6.885352	2.993199	-1.20184	Down	1.21E-38	7.50E-38
CDAN1	4757	932	397.5	5.6962	2.477017	-1.2014	Down	4.49E-50	3.33E-49
PDE4B	4273	587.5	253	4.013943	1.745664	-1.20124	Down	1.68E-31	9.16E-31
DCAF15	2274	821.5	352	10.50459	4.569616	-1.20088	Down	8.33E-44	5.66E-43
PDCD2	1961	1152	491	17.02944	7.409694	-1.20054	Down	2.05E-61	1.75E-60
ZNF816	2760	311	132.5	3.268591	1.422483	-1.20026	Down	6.95E-18	2.83E-17
KIAA0146	3253	1940	824.5	17.27526	7.518226	-1.20024	Down	########	########
BLMH	2543	1947.5	830	22.21422	9.668594	-1.20011	Down	########	########
TMEM126A	818	915.5	390	32.46287	14.13354	-1.19967	Down	3.87E-49	2.83E-48
PPM1B	1776	1451	617.5	23.68519	10.31419	-1.19935	Down	3.30E-77	3.35E-76
ACSF2	2257	475	203.5	6.101221	2.657278	-1.19915	Down	3.48E-26	1.71E-25
MRPL15	1749	1757.5	749.5	29.16387	12.70375	-1.19893	Down	1.12E-92	1.30E-91
TDRKH	2343	355.5	152	4.415424	1.923394	-1.1989	Down	6.70E-20	2.86E-19

NAA15	5554	4150.5	1765	21.63619	9.429032	-1.19827	Down	########	########
TPMT	3258	984	420	8.756338	3.816432	-1.1981	Down	1.40E-52	1.07E-51
TFDP2	9185	1594	681	5.039948	2.196945	-1.19791	Down	6.41E-84	6.91E-83
LOC100652810	605	59.5	25.5	2.869397	1.251009	-1.19766	Down	0.000187	0.000406
SAAL1	1545	570	243	10.69446	4.66301	-1.19753	Down	3.11E-31	1.70E-30
RNASEH2A	1148	1214.5	520	30.7376	13.40275	-1.19748	Down	4.77E-64	4.20E-63
ATPBD4	2139	402	171.5	5.461785	2.381548	-1.19747	Down	1.39E-22	6.32E-22
TFEB	2198	240	103.5	3.175219	1.385051	-1.19692	Down	6.97E-14	2.54E-13
MRPS16	2651	3855.5	1650	42.22622	18.42005	-1.19686	Down	########	########
TYSND1	3802	692.5	295.5	5.293508	2.310081	-1.19628	Down	1.44E-37	8.85E-37
JRK	9116	1536.5	660	4.902223	2.140036	-1.1958	Down	4.30E-80	4.49E-79
C2CD2	5848	1930	823.5	9.565887	4.176764	-1.19551	Down	########	########
POC1A	2069	584.5	249.5	8.196063	3.57879	-1.19546	Down	5.93E-32	3.28E-31
NTHL1	1067	439	187.5	11.91636	5.203939	-1.19527	Down	1.85E-24	8.79E-24
TRMT5	5302	574.5	246	3.141574	1.372194	-1.195	Down	3.76E-31	2.04E-30
CNDP2	5089	2774.5	1188.5	15.83279	6.91579	-1.19495	Down	########	########
EFR3B	7500	61.5	26.5	0.238758	0.104294	-1.19489	Down	0.000157	0.000344
NIP7	2187	2023.5	864	26.81108	11.7137	-1.19463	Down	########	########
HIST1H1A	781	46	21	1.779782	0.777697	-1.19442	Down	0.002186	0.004381
DAB1	2580	57	26	0.666709	0.291471	-1.1937	Down	0.000628	0.001314
PNPT1	4579	1627	697.5	10.30458	4.507158	-1.193	Down	3.40E-85	3.72E-84
WDR83	1219	210	90.5	5.01269	2.193072	-1.19263	Down	2.35E-12	7.75E-12
ZC3HC1	1945	1435.5	615	21.43417	9.379346	-1.19235	Down	2.65E-75	2.65E-74
CCNO	1448	373	160.5	7.499469	3.282015	-1.19221	Down	9.13E-21	3.97E-20
MRPS15	965	2800.5	1201	84.13004	36.81963	-1.19215	Down	########	########

CXorf38	4260	1556.5	667.5	10.58987	4.635438	-1.19191	Down	1.82E-81	1.93E-80
UBL7	1461	1394	601	27.7543	12.15941	-1.19064	Down	2.06E-72	2.01E-71
CHTF18	3096	1059	455.5	9.928374	4.350084	-1.19051	Down	8.67E-56	6.91E-55
C2orf18	3912	2874.5	1237	21.33627	9.348713	-1.19047	Down	########	########
CDC25A	3717	664	285.5	5.184719	2.272386	-1.19006	Down	1.11E-35	6.60E-35
PRKD1	3679	413	178	3.25683	1.428164	-1.18931	Down	1.22E-22	5.56E-22
SNRNP35	935	324	139.5	10.09314	4.428537	-1.18847	Down	3.10E-18	1.27E-17
SYTL5	4821	86.5	37.5	0.519325	0.227945	-1.18795	Down	8.27E-06	1.95E-05
SLC15A3	2134	911	392	12.37557	5.433676	-1.18749	Down	4.18E-48	3.01E-47
NUP62	3241	3072	1324.5	27.55643	12.10196	-1.18715	Down	########	########
CCHCR1	2974	1030.5	448	10.10781	4.43971	-1.18693	Down	4.15E-53	3.19E-52
SULT1C2	2832	42	19	0.440849	0.194045	-1.18389	Down	0.003156	0.006238
TPP2	4716	3355.5	1442.5	20.5781	9.060611	-1.18343	Down	########	########
MED24	3735	2480.5	1073.5	19.29095	8.495361	-1.18318	Down	########	########
HACL1	2037	424	183	6.043607	2.661627	-1.1831	Down	3.83E-23	1.77E-22
POLA1	5455	1249.5	538.5	6.636919	2.924196	-1.18247	Down	3.55E-65	3.18E-64
ZNF512	3460	1429	616	11.95839	5.270717	-1.18195	Down	3.33E-74	3.28E-73
PNO1	1994	1640.5	708	23.87885	10.52582	-1.1818	Down	1.17E-84	1.27E-83
RPUSD1	2067	1173.5	509.5	16.51541	7.28165	-1.18148	Down	1.69E-60	1.43E-59
TMEM126B	1205	1058	454	25.37148	11.18638	-1.18146	Down	7.29E-56	5.82E-55
WDR3	3877	2480	1069.5	18.54055	8.181322	-1.18028	Down	########	########
TMEM180	2834	211	92	2.165172	0.955593	-1.18001	Down	4.66E-12	1.50E-11
PAFAH1B3	1086	797	345.5	21.34474	9.421015	-1.17993	Down	7.57E-42	4.98E-41
KIAA0391	2684	802.5	346.5	8.673205	3.829107	-1.17956	Down	2.35E-42	1.56E-41
TUSC1	2477	448.5	194.5	5.261062	2.322823	-1.17948	Down	3.19E-24	1.51E-23

TCF7L2	4022	586	252	4.213514	1.860939	-1.17899	Down	1.41E-31	7.73E-31
ISOC2	1170	2003	870.5	49.82595	22.00734	-1.17891	Down	########	########
C14orf142	1191	240.5	104	5.883175	2.598915	-1.17869	Down	1.16E-13	4.20E-13
RPUSD4	2527	908	393.5	10.42186	4.606647	-1.17782	Down	1.90E-47	1.36E-46
CLDN22	2708	54	23.5	0.579685	0.256279	-1.17755	Down	0.000378	0.000804
ESPL1	6641	1385	601.5	6.056963	2.678397	-1.17722	Down	4.19E-71	4.01E-70
SF3A2	1678	3435	1491.5	59.5561	26.33834	-1.17708	Down	########	########
SALL1	5253	1550.5	674	8.582557	3.796058	-1.17691	Down	4.73E-79	4.87E-78
GTPBP6	1877	2244	977	34.73941	15.37733	-1.17577	Down	########	########
TIAF1	2110	219.5	96	3.030376	1.341706	-1.17543	Down	2.38E-12	7.86E-12
CYC1	1235	5812.5	2526	136.6819	60.51875	-1.17537	Down	########	########
TREX1	1515	674	294	12.94346	5.73277	-1.17492	Down	3.70E-35	2.18E-34
RRM1	3234	5443.5	2357	48.76564	21.60412	-1.17456	Down	########	########
CEP57L1	2915	299	129	2.966458	1.314328	-1.17442	Down	8.80E-17	3.49E-16
DNMT1	5425	7570.5	3283.5	40.44076	17.92461	-1.17387	Down	0	0
C1orf216	2934	1676.5	730	16.58793	7.354313	-1.17347	Down	4.60E-85	5.01E-84
DUS3L	2127	1273	556	17.41184	7.720665	-1.17327	Down	1.02E-64	9.08E-64
SETD1B	8195	1538.5	669.5	5.457456	2.420014	-1.17321	Down	2.09E-78	2.14E-77
PCCA	2577	531	230	5.961066	2.643619	-1.17306	Down	2.11E-28	1.09E-27
KLHL38	1834	115	52	1.868501	0.828647	-1.17305	Down	8.74E-07	2.16E-06
FAM203B	2404	654.5	285	7.907561	3.507486	-1.1728	Down	3.18E-34	1.83E-33
USP1	3519	3762.5	1632.5	31.02511	13.76363	-1.17258	Down	########	########
EXOSC9	1593	1431.5	618.5	26.01314	11.54552	-1.17191	Down	4.69E-74	4.62E-73
TSFM	2102	972.5	423	13.41532	5.954184	-1.17191	Down	3.63E-50	2.69E-49
CCDC89	2428	119	54	1.455964	0.646209	-1.1719	Down	6.18E-07	1.54E-06

WDR18	1551	1186	518.5	22.25935	9.880771	-1.17172	Down	2.04E-60	1.72E-59
KLRC4-KLRK1	1947	43.5	19	0.647424	0.287394	-1.17168	Down	0.002228	0.004459
CCL2	760	3105	1346.5	118.2143	52.48058	-1.17155	Down	########	########
ZNF300	3282	549.5	242.5	4.884125	2.170203	-1.17027	Down	2.14E-28	1.10E-27
CCDC73	4015	100	44	0.724039	0.321777	-1.17001	Down	2.49E-06	6.04E-06
SCARA5	4170	49.5	22	0.351819	0.156367	-1.1699	Down	0.001294	0.002644
PSMG2	1141	1700	740.5	43.23983	19.2243	-1.16943	Down	2.36E-86	2.60E-85
SMARCB1	1690	1699.5	741.5	29.20199	12.98577	-1.16913	Down	5.42E-86	5.97E-85
DLG3	5087	487.5	212	2.77673	1.234907	-1.16898	Down	5.99E-26	2.93E-25
FANCL	1753	355.5	154	5.866814	2.609458	-1.16883	Down	1.86E-19	7.86E-19
SNRPA1	1068	1710	745.5	46.54061	20.72399	-1.16719	Down	1.04E-86	1.15E-85
CORO1B	1920	3643	1596	55.20121	24.58162	-1.16712	Down	########	########
ANKDD1A	3126	200.5	88	1.864361	0.830237	-1.16709	Down	2.50E-11	7.53E-11
NTNG2	3113	111.5	49.5	1.050252	0.467723	-1.16701	Down	7.52E-07	1.87E-06
UTP15	3655	1057.5	460.5	8.380876	3.732372	-1.16701	Down	2.13E-54	1.66E-53
EMG1	1072	1453	632.5	39.27568	17.49306	-1.16685	Down	3.52E-74	3.46E-73
FAM189B	3209	2629	1152	23.84385	10.62127	-1.16666	Down	########	########
DNAAF2	2832	233	102	2.391718	1.065979	-1.16587	Down	4.46E-13	1.55E-12
C14orf80	1620	333.5	146.5	5.996899	2.673883	-1.16528	Down	5.23E-18	2.14E-17
CHRNA5	3646	305.5	134	2.438476	1.087729	-1.16466	Down	1.47E-16	5.79E-16
MRPS14	2160	946.5	412.5	12.6988	5.666298	-1.16421	Down	8.39E-49	6.10E-48
UGT1A3	2345	298.5	130.5	3.694284	1.64925	-1.16348	Down	2.05E-16	8.06E-16
FAM203A	2441	744.5	327.5	8.878323	3.964932	-1.16299	Down	4.21E-38	2.59E-37
ZNF234	4482	525.5	230	3.402877	1.519992	-1.16269	Down	1.57E-27	7.93E-27
RASL11A	1543	166.5	72.5	3.123106	1.395167	-1.16254	Down	7.22E-10	2.04E-09

SFMBT1	4558	609.5	267.5	3.881372	1.734019	-1.16245	Down	1.24E-31	6.80E-31
HOXB5	1830	39	17	0.618901	0.276506	-1.1624	Down	0.003202	0.006324
GNAI1	3318	8409	3680.5	73.55624	32.87065	-1.16205	Down	0	0
EHMT2	3982	3149	1383	22.96876	10.26766	-1.16157	Down	########	########
HTATSF1	3037	2548.5	1114	24.33472	10.87906	-1.16146	Down	########	########
ARMC4	3572	160.5	70.5	1.31054	0.586076	-1.161	Down	1.99E-09	5.50E-09
ACAA2	1952	1407	617.5	20.93434	9.36222	-1.16095	Down	8.35E-71	7.96E-70
TDRD3	2592	526	230	5.886871	2.632738	-1.16094	Down	1.12E-27	5.69E-27
TNPO2	5092	3608	1583	20.57571	9.202967	-1.16077	Down	########	########
DZIP1	7457	1499.5	657	5.836144	2.611513	-1.16013	Down	2.17E-75	2.17E-74
GALNT9	1744	304	134	5.073853	2.270718	-1.15993	Down	2.05E-16	8.05E-16
GMNN	1215	1011.5	442.5	24.12423	10.7964	-1.15993	Down	1.14E-51	8.62E-51
FUT8	4749	1120.5	491	6.839972	3.06209	-1.15947	Down	9.28E-57	7.49E-56
ICT1	888	792.5	347.5	25.87725	11.59324	-1.1584	Down	1.09E-40	7.04E-40
HEATR1	8497	3701.5	1624.5	12.62835	5.658265	-1.15823	Down	########	########
SPA17	955	251	110	7.639226	3.422875	-1.15822	Down	5.99E-14	2.19E-13
PPIL1	1750	1598.5	703.5	26.54203	11.89655	-1.15773	Down	1.10E-79	1.14E-78
USP18	2037	336.5	148	4.802494	2.153074	-1.15739	Down	5.23E-18	2.14E-17
FANCE	2565	255	112	2.885094	1.293607	-1.15722	Down	4.29E-14	1.57E-13
MRPL12	1032	3130	1379.5	88.20155	39.56572	-1.15655	Down	########	########
ALDH1B1	3044	1374.5	603.5	13.09648	5.875076	-1.1565	Down	4.38E-69	4.10E-68
MUTYH	1945	342	152.5	5.138702	2.305272	-1.15647	Down	5.19E-18	2.12E-17
KBTBD6	5228	1217	536	6.766217	3.036102	-1.15613	Down	5.47E-61	4.65E-60
MMP16	6347	344	151	1.569422	0.704338	-1.15589	Down	1.63E-18	6.75E-18
FH	1877	4359	1913.5	67.34094	30.23316	-1.15535	Down	########	########

GYG1	2051	2145	940.5	30.28667	13.59748	-1.15534	Down	########	########
ZFHX4	13975	2594	1154.5	5.418745	2.433218	-1.15509	Down	########	########
CS	2997	7437	3272.5	71.94944	32.31273	-1.15488	Down	0	0
APEX2	1991	1153	509	16.83873	7.563484	-1.15466	Down	1.28E-57	1.04E-56
TBX2	3396	1855.5	819	15.87975	7.133937	-1.15442	Down	1.42E-91	1.63E-90
ATAD2	5580	3779	1660	19.62537	8.821223	-1.15367	Down	########	########
PTTG1	728	2651	1165.5	105.6488	47.49613	-1.1534	Down	########	########
JAKMIP2	3357	842	372	7.282123	3.274125	-1.15325	Down	1.74E-42	1.16E-41
HSPA2	2802	161	72.5	1.688769	0.759347	-1.15314	Down	3.86E-09	1.06E-08
CEP85	3961	1218	538.5	8.938437	4.020451	-1.15267	Down	1.05E-60	8.92E-60
NDE1	3222	943	417	8.522718	3.835476	-1.15191	Down	2.70E-47	1.92E-46
LRRC8B	7766	265	119	1.000681	0.450381	-1.15176	Down	4.95E-14	1.81E-13
ATF1	2505	866.5	381	10.02743	4.515922	-1.15086	Down	5.44E-44	3.70E-43
HSPA4	3385	10671	4701.5	91.35505	41.14627	-1.15072	Down	0	0
TCP1	2377	7298	3213	88.97863	40.0787	-1.15062	Down	0	0
SMARCE1	2425	4618	2035	55.17178	24.85494	-1.1504	Down	########	########
EMILIN2	4009	108	48	0.787712	0.354866	-1.15039	Down	1.25E-06	3.07E-06
GEMIN5	5404	1871.5	827.5	10.05551	4.530613	-1.15021	Down	3.79E-92	4.37E-91
PCYT2	3265	1566	694	13.9451	6.286552	-1.14942	Down	4.81E-77	4.87E-76
NOC2L	2817	6950.5	3076	71.73392	32.35636	-1.14861	Down	0	0
C7orf26	1913	1430.5	631.5	21.69634	9.790571	-1.14799	Down	4.30E-71	4.12E-70
SLC25A10	1960	935.5	413.5	13.86599	6.257787	-1.14783	Down	5.25E-47	3.70E-46
HABP4	2708	732	323	7.831687	3.534927	-1.14764	Down	3.05E-37	1.86E-36
CCT2	1962	6459.5	2848.5	95.36593	43.06095	-1.14709	Down	0	0
BCS1L	1454	577	256.5	11.53215	5.209115	-1.14655	Down	2.08E-29	1.10E-28

PSMA4	1159	6559.5	2891	163.8741	74.04044	-1.1462	Down	0	0
LRRC8D	3782	1217.5	539.5	9.349909	4.225008	-1.146	Down	2.39E-60	2.01E-59
CDK4	1474	5124.5	2274.5	100.9051	45.59673	-1.146	Down	########	########
C1QTNF2	2411	202	89.5	2.423677	1.095632	-1.14543	Down	2.11E-11	6.40E-11
TEF	4349	368.5	163	2.459878	1.112007	-1.14542	Down	2.20E-19	9.27E-19
PHF15	6463	6414	2848.5	28.86236	13.04982	-1.14516	Down	0.00E-01	########
SRSF4	2535	3600	1593	41.23145	18.64507	-1.14495	Down	########	########
PSMD6	1308	3431.5	1518	76.06863	34.40158	-1.14483	Down	########	########
PPIH	813	1170	515	41.54735	18.79243	-1.14461	Down	9.12E-59	7.60E-58
INCA1	1404	80.5	35.5	1.662276	0.752215	-1.14394	Down	2.33E-05	5.37E-05
PFKFB2	3529	480	213.5	3.958879	1.791582	-1.14386	Down	1.00E-24	4.79E-24
ANKRD23	2591	87	38.5	0.969911	0.439162	-1.1431	Down	9.85E-06	2.31E-05
LYRM2	5371	1119.5	494	6.035179	2.733361	-1.14272	Down	5.68E-56	4.55E-55
DHX35	3336	669	297	5.821117	2.637839	-1.14194	Down	8.52E-34	4.88E-33
CCDC74B	1471	42	19	0.834691	0.378446	-1.14116	Down	0.003156	0.006237
ACSL5	3399	888	393.5	7.555851	3.426093	-1.14103	Down	1.44E-44	9.86E-44
C8orf73	3251	181.5	80.5	1.618545	0.734012	-1.14082	Down	2.62E-10	7.53E-10
LARS2	4203	1289	575	8.902295	4.039115	-1.14014	Down	6.06E-63	5.25E-62
CKLF	792	1470.5	652.5	53.82436	24.42864	-1.13969	Down	2.56E-72	2.49E-71
CENPN	4659	2692	1196	16.74594	7.60325	-1.13912	Down	########	########
FAM136A	1824	1892	840.5	30.04382	13.64359	-1.13884	Down	2.26E-92	2.61E-91
UBB	971	25051	11130	748.3244	339.9572	-1.13831	Down	0	0
ADAT1	3545	1404.5	626	11.50069	5.225724	-1.13802	Down	1.84E-68	1.71E-67
SNRPE	1593	3553	1574.5	64.59194	29.35043	-1.13797	Down	########	########
HMGN3	894	981.5	435.5	31.81848	14.46011	-1.13779	Down	7.09E-49	5.15E-48

DBF4B	1821	333	149	5.305876	2.411375	-1.13774	Down	2.30E-17	9.25E-17
SH3TC1	4273	650.5	291	4.436369	2.016278	-1.13768	Down	2.28E-32	1.27E-31
HOXC6	1681	343.5	153	5.925596	2.694226	-1.13709	Down	6.09E-18	2.48E-17
HDGFRP3	1973	1995.5	889	29.35834	13.35541	-1.13635	Down	1.31E-96	1.56E-95
IPO4	3606	1930.5	865	15.57423	7.085851	-1.13615	Down	1.76E-92	2.03E-91
C15orf41	2595	378.5	168.5	4.225284	1.922448	-1.1361	Down	9.54E-20	4.06E-19
MRPL22	3190	1283.5	570.5	11.65954	5.309675	-1.13482	Down	3.78E-63	3.28E-62
MRPL1	1377	654	290	13.72508	6.250308	-1.13481	Down	3.79E-33	2.15E-32
AXIN2	4241	91	41	0.627281	0.285689	-1.13467	Down	1.15E-05	2.69E-05
ELOVL6	3392	879.5	393	7.518725	3.424469	-1.13461	Down	2.72E-43	1.84E-42
POR	2509	4933.5	2211	57.1624	26.0474	-1.13393	Down	########	########
RFXAP	2825	336	149.5	3.438051	1.566843	-1.13373	Down	8.57E-18	3.48E-17
ZRANB3	3717	178.5	80	1.396487	0.637135	-1.13213	Down	7.09E-10	2.00E-09
NDUFS2	2082	3878.5	1733	54.06111	24.66838	-1.13193	Down	########	########
PRMT7	2193	1148	514.5	15.22665	6.948805	-1.13176	Down	7.44E-56	5.93E-55
OTUD6B	3306	939.5	418.5	8.231835	3.758291	-1.13114	Down	1.63E-46	1.15E-45
CETN3	997	743.5	331.5	21.62633	9.875971	-1.13079	Down	4.16E-37	2.53E-36
ABHD3	2085	549.5	245	7.643528	3.491307	-1.13047	Down	9.31E-28	4.72E-27
STMN3	2255	2751	1237	35.45564	16.19508	-1.13046	Down	########	########
C15orf63	1295	1936	865.5	43.36894	19.81342	-1.13018	Down	2.51E-93	2.93E-92
ABHD16A	2146	2733.5	1227.5	37.03737	16.92432	-1.12988	Down	########	########
G2E3	5581	961	429.5	4.984863	2.277907	-1.12985	Down	2.62E-47	1.86E-46
PXMP4	5727	937	419.5	4.745977	2.169208	-1.12954	Down	5.08E-46	3.54E-45
C2orf47	1525	494.5	221	9.421463	4.307854	-1.12898	Down	5.06E-25	2.43E-24
ATG4C	2690	516.5	231	5.571661	2.548109	-1.12868	Down	4.94E-26	2.42E-25

TIMM50	2572	2165.5	971.5	24.45492	11.18418	-1.12866	Down	########	########
SEC16B	4024	225.5	101.5	1.62435	0.742883	-1.12866	Down	3.86E-12	1.25E-11
LRRC20	2925	353	158	3.498488	1.600017	-1.12864	Down	2.63E-18	1.08E-17
AAAS	1854	1651	741	25.84385	11.82118	-1.12845	Down	3.65E-79	3.76E-78
EIF2AK2	2696	1947.5	871	20.94461	9.582047	-1.12817	Down	1.28E-93	1.50E-92
NUP93	2876	3722	1671	37.54837	17.18594	-1.12752	Down	########	########
C3orf26	1230	849	379	19.98153	9.145958	-1.12746	Down	5.38E-42	3.55E-41
BCCIP	1261	2511.5	1122	57.72035	26.42044	-1.12743	Down	########	########
SNRNP70	1940	6526.5	2929	97.7471	44.7498	-1.12717	Down	########	########
THOC4	1113	3220	1445	84.06418	38.49579	-1.12679	Down	########	########
PAXIP1	3723	1103.5	495.5	8.589138	3.933607	-1.12666	Down	1.70E-53	1.32E-52
TTLL12	3421	2811.5	1267	23.89542	10.94538	-1.12641	Down	########	########
LRRC34	1892	89.5	40.5	1.367566	0.626688	-1.12579	Down	1.37E-05	3.19E-05
MYBBP1A	4122	3559.5	1599	25.04428	11.48142	-1.12518	Down	########	########
ANP32E	3347	6110	2737.5	52.95042	24.27999	-1.12487	Down	########	########
DIS3L	3714	1240.5	559.5	9.714098	4.456574	-1.12415	Down	2.14E-59	1.79E-58
SUMO2	994	7370.5	3299	214.5869	98.4482	-1.12413	Down	0	0
CCDC138	2196	286	129	3.789138	1.739115	-1.12352	Down	6.58E-15	2.47E-14
CTU1	2107	182.5	82	2.510045	1.152117	-1.12343	Down	5.04E-10	1.43E-09
MECOM	5195	1182	534	6.614008	3.038053	-1.12238	Down	1.83E-56	1.47E-55
FAM22A	3308	95	43	0.834844	0.383536	-1.12214	Down	8.12E-06	1.92E-05
MAD2L1	1453	2157.5	968.5	43.02411	19.77125	-1.12174	Down	########	########
SLC16A9	4001	780	352	5.658085	2.600576	-1.12148	Down	6.31E-38	3.88E-37
CTCF	3946	3120.5	1401	22.91644	10.53426	-1.12129	Down	########	########
SCRN1	5286	13816.5	6224.5	75.86793	34.88145	-1.12103	Down	0	0

DEPDC4	1155	47	21.5	1.193869	0.548927	-1.12096	Down	0.00155	0.003145
RPUSD3	1244	611	275.5	14.24205	6.549778	-1.12064	Down	3.21E-30	1.71E-29
VRK2	1944	59.5	27	0.894177	0.41128	-1.12044	Down	0.000524	0.001105
LPA	6489	50	24	0.232483	0.106973	-1.11988	Down	0.002444	0.004875
C6orf136	1441	459	207	9.234199	4.249162	-1.11981	Down	5.15E-23	2.36E-22
DUSP19	5226	167	76	0.933565	0.429656	-1.11957	Down	3.75E-09	1.03E-08
TMEM201	3857	317	144	2.388629	1.0995	-1.11933	Down	3.75E-16	1.47E-15
HNRNPA0	2983	6496.5	2933.5	63.22189	29.111	-1.11886	Down	########	########
HNRNPU	6846	24424.5	10998	103.437	47.64257	-1.11843	Down	0	0
CNKSR3	3277	397	180	3.531577	1.627129	-1.11798	Down	6.45E-20	2.76E-19
TACO1	1479	888.5	401.5	17.48194	8.054887	-1.11793	Down	6.88E-43	4.61E-42
IFIT2	3505	536.5	242.5	4.446245	2.048873	-1.11776	Down	1.49E-26	7.40E-26
NOD1	4506	330	150	2.136103	0.984417	-1.11764	Down	9.83E-17	3.89E-16
TPK1	2449	46	21	0.546967	0.252104	-1.11744	Down	0.002186	0.00438
SMAD6	1293	1409.5	635.5	31.6002	14.56969	-1.11696	Down	2.76E-67	2.54E-66
RTTN	7109	503	227	2.053803	0.947108	-1.1167	Down	4.89E-25	2.35E-24
SF3B3	9736	10389.5	4688.5	30.89806	14.25162	-1.11639	Down	0	0
TAF4	4647	1340	606	8.375452	3.864161	-1.11601	Down	1.21E-63	1.06E-62
MRPL38	1614	1682	763	30.31838	13.98829	-1.11597	Down	6.06E-79	6.24E-78
GIN1	3549	310	139.5	2.529536	1.167323	-1.11567	Down	3.30E-16	1.29E-15
LPCAT4	1908	614.5	279	9.38082	4.330584	-1.11515	Down	8.20E-30	4.34E-29
MRM1	1851	304.5	138	4.775696	2.204665	-1.11515	Down	1.43E-15	5.50E-15
UTS2D	2408	123.5	56	1.49117	0.688678	-1.11454	Down	4.37E-07	1.10E-06
CCDC111	2295	310	140.5	3.921687	1.811208	-1.11452	Down	5.34E-16	2.08E-15
FUNDC1	1170	812.5	367	20.17338	9.317112	-1.1145	Down	2.68E-39	1.69E-38

C14orf21	2047	580.5	263	8.234631	3.804154	-1.11413	Down	2.30E-28	1.18E-27
RPIA	1834	686.5	311.5	10.88931	5.030736	-1.11407	Down	2.90E-33	1.65E-32
DHX9	4543	9426.5	4255.5	60.05379	27.74988	-1.11377	Down	0	0
WBSCR16	2386	3594	1628	43.76345	20.22258	-1.11376	Down	########	########
EXOSC5	1006	340	155.5	9.848855	4.551802	-1.11352	Down	4.21E-17	1.69E-16
TUBGCP3	3908	1883.5	854	13.98114	6.462726	-1.11327	Down	3.42E-88	3.83E-87
ATAD5	6245	466	211.5	2.168655	1.002458	-1.11326	Down	3.63E-23	1.67E-22
PSMB2	4235	14117.5	6394	96.7129	44.71231	-1.11304	Down	0	0
NUDT16L1	1437	1004	456	20.30987	9.38974	-1.11302	Down	9.98E-48	7.15E-47
EXOG	3146	535	242	4.922434	2.276714	-1.11242	Down	2.06E-26	1.02E-25
BIVM	3210	1169	528.5	10.55122	4.881662	-1.11197	Down	6.89E-56	5.50E-55
SLC38A6	1766	739	333	12.08087	5.589534	-1.11192	Down	4.00E-36	2.39E-35
STRA13	863	1691	769	56.99058	26.37306	-1.11166	Down	5.79E-79	5.96E-78
ANKRD30B	4617	227	102.5	1.426036	0.659934	-1.11162	Down	3.26E-12	1.06E-11
HEXIM1	4785	2080.5	943.5	12.60782	5.835967	-1.11128	Down	2.71E-97	3.25E-96
SLC9A3R1	2013	1312.5	597	18.94981	8.771881	-1.11123	Down	1.34E-61	1.14E-60
NUSAP1	2434	3225.5	1459	38.42692	17.78822	-1.11119	Down	########	########
CCBL1	1925	159	72	2.399331	1.110795	-1.11104	Down	7.46E-09	2.02E-08
DNAJB1	2233	4004.5	1815.5	52.01385	24.08101	-1.111	Down	########	########
HOXA1	2561	277	124.5	3.121402	1.445182	-1.11094	Down	1.10E-14	4.13E-14
EMR1	3150	464	211.5	4.293008	1.988777	-1.11011	Down	6.91E-23	3.16E-22
PRR22	1407	76	34.5	1.563718	0.724459	-1.11	Down	5.49E-05	0.000124
CHCHD1	844	1185	537.5	40.72831	18.87069	-1.10988	Down	2.90E-56	2.33E-55
PPA1	1316	5084	2298	111.838	51.83343	-1.10946	Down	########	########
ATN1	4367	10331.5	4706.5	68.78488	31.88297	-1.10931	Down	0	0

KNTC1	6965	1460.5	660.5	6.056302	2.809905	-1.10792	Down	3.08E-69	2.88E-68
RIPK2	2588	1095.5	499.5	12.2799	5.698464	-1.10765	Down	1.49E-51	1.12E-50
PPP1R3D	3481	593	268.5	4.943957	2.294655	-1.10739	Down	3.78E-29	1.98E-28
ELMO1	3590	436.5	199.5	3.543044	1.64476	-1.10711	Down	1.89E-21	8.33E-21
DOLK	2267	1072.5	488.5	13.7354	6.376374	-1.10709	Down	1.30E-50	9.66E-50
LYPD5	2518	158	72.5	1.826834	0.848119	-1.10701	Down	1.03E-08	2.78E-08
PPARGC1B	10642	376	171.5	1.027853	0.477404	-1.10635	Down	7.76E-19	3.24E-18
C4orf27	1233	634	287.5	14.88997	6.918144	-1.10588	Down	5.91E-31	3.20E-30
SATB2	5326	981.5	449	5.359445	2.49394	-1.10366	Down	5.70E-46	3.97E-45
TWISTNB	3892	2169.5	987.5	16.14533	7.513567	-1.10355	Down	########	1.53E-99
CENPK	1781	892.5	405.5	14.52165	6.759624	-1.10319	Down	1.27E-42	8.47E-42
HNRNPL	2129	9986.5	4547	136.0395	63.32522	-1.10317	Down	0	0
NDUFV2	937	5722	2601.5	176.8817	82.35079	-1.10293	Down	########	########
PDLIM3	2853	351.5	161.5	3.597033	1.674875	-1.10275	Down	2.10E-17	8.46E-17
KATNAL2	2850	129	59	1.317015	0.613322	-1.10256	Down	2.60E-07	6.59E-07
PAIP1	2559	3722.5	1694	42.16169	19.63441	-1.10255	Down	########	########
HMGN1	1313	7166.5	3262	158.139	73.64434	-1.10255	Down	0	0
PIPOX	2412	86.5	42	1.081296	0.503633	-1.10232	Down	9.21E-05	0.000204
SRGAP2	6303	3946.5	1802.5	18.16156	8.460991	-1.10199	Down	########	########
SMARCD1	3308	1914.5	875.5	16.81546	7.834374	-1.1019	Down	3.38E-88	3.79E-87
TAF1B	2306	557.5	253.5	6.993933	3.259286	-1.10155	Down	3.24E-27	1.63E-26
GPC2	2666	105.5	47.5	1.137248	0.530085	-1.10125	Down	2.08E-06	5.05E-06
HNRNPD	2053	10541.5	4803	148.7705	69.36039	-1.1009	Down	0	0
LDHB	1317	16302.5	7429.5	358.8376	167.318	-1.10074	Down	0	0
GPR39	2821	1282	588	13.21578	6.163838	-1.10036	Down	2.75E-59	2.29E-58

ABCD4	3157	292.5	135	2.701927	1.260385	-1.10012	Down	1.63E-14	6.06E-14
NARS2	2513	595.5	273	6.879785	3.210973	-1.09935	Down	2.11E-28	1.08E-27
TSHZ1	4982	1012	463.5	5.905057	2.756069	-1.09934	Down	2.09E-47	1.49E-46
ZNF593	653	829	379	36.92462	17.23403	-1.09932	Down	3.36E-39	2.11E-38
PLEKHF1	1774	317.5	146	5.214769	2.43442	-1.09903	Down	9.62E-16	3.72E-15
NOL12	2840	596	272.5	6.081244	2.839191	-1.09889	Down	9.59E-29	4.97E-28
KIAA1683	4421	79	38	0.533737	0.24925	-1.09854	Down	0.000138	0.000303
MKL2	8680	1751.5	801	5.851995	2.73336	-1.09825	Down	9.69E-81	1.02E-79
MYO18A	7591	2653.5	1217.5	10.15273	4.742214	-1.09824	Down	########	########
IRX1	1858	125.5	57.5	1.961404	0.916273	-1.09804	Down	3.67E-07	9.25E-07
CASP9	2165	172	78.5	2.299959	1.074494	-1.09795	Down	1.92E-09	5.32E-09
AKAP1	4048	1017.5	466.5	7.311145	3.416598	-1.09754	Down	1.73E-47	1.24E-46
TMSB15B	683	135	61.5	5.70729	2.667213	-1.09747	Down	9.54E-08	2.47E-07
FAM109B	2369	224	102.5	2.748234	1.284347	-1.09747	Down	8.65E-12	2.70E-11
TPX2	3685	8435	3855	66.37184	31.03515	-1.09667	Down	0	0
TACC2	3916	600	278.5	4.471053	2.090952	-1.09645	Down	4.39E-28	2.25E-27
CXCL12	524	56.5	27	3.192502	1.493036	-1.09644	Down	0.001404	0.002861
FHAD1	5090	150	70	0.861193	0.402824	-1.09619	Down	5.30E-08	1.39E-07
KPNB1	4205	15630	7146	107.7259	50.40323	-1.09578	Down	0	0
CENPO	4102	1880	863	13.3154	6.231005	-1.09556	Down	6.01E-86	6.61E-85
GPR125	4576	1310	600.5	8.292003	3.880594	-1.09544	Down	1.03E-60	8.71E-60
STEAP2	6708	1407.5	643.5	6.075927	2.844261	-1.09505	Down	2.29E-65	2.06E-64
IKBKG	1982	2454.5	1128.5	35.99137	16.85294	-1.09465	Down	########	########
BAZ1A	5935	2375	1092	11.63567	5.448979	-1.0945	Down	########	########
TTLL2	2877	47	23	0.493649	0.231223	-1.0942	Down	0.004065	0.007944

GATSL2	990	89	41	2.615695	1.225292	-1.09407	Down	2.23E-05	5.14E-05
SDSL	1414	206.5	94.5	4.221454	1.977514	-1.09405	Down	6.48E-11	1.91E-10
CHEK2	1991	229.5	105	3.341946	1.565582	-1.09399	Down	7.15E-12	2.25E-11
SIPA1L2	6501	3037.5	1408	13.6376	6.389813	-1.09374	Down	########	########
RDH14	1602	196	90	3.553783	1.665989	-1.09298	Down	2.45E-10	7.04E-10
POLR3GL	1191	1030.5	472.5	25.09531	11.76473	-1.09295	Down	4.59E-48	3.31E-47
FANCM	7144	372	171	1.511964	0.708936	-1.0927	Down	2.79E-18	1.15E-17
C20orf96	1615	282	130.5	5.081	2.383209	-1.09221	Down	3.83E-14	1.41E-13
FPGT	3682	776.5	356.5	6.110527	2.86687	-1.09182	Down	1.49E-36	9.00E-36
WDYHV1	1348	406	187	8.76236	4.11213	-1.09143	Down	9.77E-20	4.16E-19
URB2	5613	1538	711	7.97196	3.741461	-1.09133	Down	1.20E-69	1.13E-68
ETHE1	978	771	354	22.86682	10.73251	-1.09127	Down	2.88E-36	1.73E-35
PION	3265	396	182	3.521947	1.654334	-1.09012	Down	2.28E-19	9.61E-19
KIAA1644	6741	2562	1180	11.03111	5.182361	-1.0899	Down	########	########
ARHGAP26	8876	705	327.5	2.315309	1.087901	-1.08966	Down	1.21E-32	6.80E-32
INPP1	2179	675	311	8.978353	4.22003	-1.0892	Down	9.67E-32	5.32E-31
MOCOS	2747	347.5	161	3.678884	1.730071	-1.08844	Down	7.47E-17	2.96E-16
DECR1	1251	2383.5	1096.5	55.20092	25.96312	-1.08823	Down	########	########
LIN9	3340	479.5	221.5	4.167382	1.960096	-1.08822	Down	6.26E-23	2.87E-22
CCDC106	1993	151	70.5	2.222656	1.04574	-1.08776	Down	3.85E-08	1.01E-07
GLI3	8228	631.5	292	2.232602	1.050965	-1.08701	Down	1.60E-29	8.42E-29
GTF2H2C	2106	1162	534.5	15.98408	7.52685	-1.08652	Down	1.08E-53	8.34E-53
AGK	2925	2323	1073	23.06677	10.86378	-1.08629	Down	########	########
C17orf97	1839	245	114	3.874731	1.825633	-1.0857	Down	2.90E-12	9.47E-12
VARS2	3629	1158.5	536	9.275911	4.371889	-1.08523	Down	9.55E-53	7.31E-52

MAD2L2	1196	270.5	125	6.564739	3.095894	-1.08438	Down	1.70E-13	6.06E-13
RTKN2	6659	203.5	94	0.882615	0.416237	-1.08438	Down	1.70E-10	4.91E-10
NGDN	1142	483	222.5	12.23471	5.769914	-1.08436	Down	2.80E-23	1.30E-22
MAP9	7333	2488	1147.5	9.843442	4.643215	-1.08404	Down	########	########
GATSL3	1644	220	102	3.891562	1.836285	-1.08356	Down	3.12E-11	9.34E-11
HELLS	3165	462	212.5	4.220575	1.992334	-1.08298	Down	2.09E-22	9.48E-22
C9orf37	1468	608	281	12.0126	5.673347	-1.08228	Down	1.41E-28	7.28E-28
LRWD1	2220	1809	841	23.71512	11.20059	-1.08223	Down	1.17E-80	1.23E-79
LIFR	10107	4462	2061	12.797	6.046061	-1.08174	Down	########	########
HSPE1-PHOCN	4279	1116	513.5	7.540181	3.563724	-1.08121	Down	1.31E-51	9.85E-51
BYSL	2005	1185.5	550.5	17.18121	8.120393	-1.08121	Down	1.23E-53	9.52E-53
PTMA	1223	24296.5	11229	576.214	272.3519	-1.08113	Down	0	0
ZNF680	3050	517.5	240.5	4.935592	2.333212	-1.08091	Down	2.49E-24	1.18E-23
EWSR1	2676	7061	3272	76.59631	36.22085	-1.08045	Down	0	0
EIF2B5	2898	1447	670.5	14.49244	6.853819	-1.08032	Down	2.03E-65	1.82E-64
SAE1	2393	5505	2547.5	66.77401	31.5919	-1.07973	Down	########	########
MSH2	3145	1910.5	885	17.63796	8.345535	-1.07961	Down	1.21E-85	1.33E-84
ILF3	3757	13801	6405	106.5398	50.44189	-1.0787	Down	0	0
PSMD11	1598	5063	2345	91.92017	43.53031	-1.07836	Down	########	########
LYRM5	1149	552	256	13.9019	6.584885	-1.07805	Down	6.43E-26	3.14E-25
SRRT	3020	6212.5	2883	59.66075	28.26169	-1.07793	Down	########	########
ZHX1	5139	3250	1503.5	18.30703	8.675403	-1.0774	Down	########	########
SLC26A11	2930	334	156	3.323198	1.575101	-1.07713	Down	4.51E-16	1.76E-15
EIF2B4	1763	944.5	439.5	15.59569	7.392516	-1.07701	Down	6.53E-43	4.38E-42
RPUSD2	1839	421	196	6.661233	3.158104	-1.07673	Down	5.45E-20	2.34E-19

TARBP2	1488	1388	646.5	27.0795	12.84098	-1.07645	Down	3.63E-62	3.12E-61
TSPAN12	2579	479	223.5	5.385878	2.554796	-1.07597	Down	1.57E-22	7.13E-22
MRPL20	749	2457.5	1141.5	95.19117	45.15838	-1.07583	Down	########	########
BCL9	6278	1238	579	5.740028	2.723564	-1.07556	Down	4.36E-55	3.44E-54
TPRKB	752	695.5	323	26.82728	12.73235	-1.0752	Down	4.47E-32	2.48E-31
CRISPLD2	4607	206	96	1.307082	0.620714	-1.07435	Down	1.65E-10	4.79E-10
RPP30	2371	1113.5	516	13.5935	6.455694	-1.07427	Down	1.34E-50	9.96E-50
SAP30L	6120	744	345.5	3.520894	1.672587	-1.07386	Down	2.25E-34	1.30E-33
PPP5C	2166	2624	1223.5	35.21279	16.73281	-1.07342	Down	########	########
LRIG3	3662	898	419	7.11859	3.382806	-1.07337	Down	1.25E-40	8.07E-40
HSF2	2643	353.5	165	3.880521	1.844358	-1.07313	Down	7.08E-17	2.81E-16
CENPB	2856	8310.5	3881	84.58162	40.20516	-1.07296	Down	0	0
PHF11	1471	893.5	414	17.583	8.36004	-1.0726	Down	6.11E-41	3.96E-40
NUCKS1	6471	27090	12581	121.3677	57.70689	-1.07257	Down	0	0
ERCC3	2751	1610	751.5	17.00848	8.088567	-1.0723	Down	1.72E-71	1.66E-70
MDFIC	5312	3719	1731.5	20.30789	9.659348	-1.07204	Down	########	########
NICN1	3227	521.5	244.5	4.700199	2.235986	-1.07181	Down	4.42E-24	2.08E-23
BRAT1	3013	3607.5	1691.5	34.86258	16.58632	-1.07169	Down	########	########
AZI1	3630	823	384	6.589255	3.13553	-1.07141	Down	2.23E-37	1.36E-36
RAD54B	3074	826.5	385	7.791208	3.708343	-1.07107	Down	1.38E-37	8.46E-37
PDS5A	2745	5370.5	2498	56.70443	26.9934	-1.07085	Down	########	########
RSAD1	2499	1282	599	14.89339	7.097972	-1.06919	Down	4.27E-57	3.46E-56
AGBL3	3542	95	46	0.790381	0.376835	-1.06862	Down	3.29E-05	7.52E-05
COPG2	3154	2165	1009	19.89714	9.488106	-1.06837	Down	1.14E-95	1.36E-94
SCRN2	1582	1298	606	23.80652	11.35293	-1.06829	Down	7.03E-58	5.76E-57

C19orf57	2420	49	23	0.590033	0.281394	-1.0682	Down	0.002118	0.004249
GABPB1	1622	1181	551	21.09916	10.06353	-1.06805	Down	6.77E-53	5.19E-52
SFPQ	3073	10520	4906	99.26382	47.35199	-1.06784	Down	0	0
ATG3	1372	1717.5	799.5	36.26061	17.30176	-1.06748	Down	1.70E-76	1.71E-75
KLF9	5208	4281.5	2003	23.86741	11.39068	-1.06719	Down	########	########
IQSEC2	5248	1916	898	10.5973	5.058058	-1.06704	Down	6.85E-84	7.38E-83
PPARG	1892	354	167	5.441556	2.597563	-1.06686	Down	1.28E-16	5.07E-16
SNRPF	806	2010.5	938	72.27604	34.52465	-1.06589	Down	1.04E-88	1.17E-87
WDR36	6611	5421	2531	23.76922	11.35888	-1.06527	Down	########	########
UCK2	4940	1430.5	670.5	8.415083	4.022895	-1.06474	Down	4.06E-63	3.52E-62
THAP7	1097	685.5	323.5	18.20999	8.706706	-1.06453	Down	1.00E-30	5.40E-30
RNF43	4573	330	155	2.098533	1.003496	-1.06435	Down	1.00E-15	3.86E-15
GFM1	3468	2642.5	1234.5	22.07753	10.55861	-1.06416	Down	########	########
MRPS36	1329	798.5	371.5	17.37161	8.309513	-1.0639	Down	1.48E-36	8.89E-36
TAP2	5679	7669	3606	39.2337	18.77661	-1.06316	Down	0	0
DCPS	1508	960	451	18.475	8.844632	-1.0627	Down	1.03E-42	6.89E-42
PAK1IP1	1511	1004	470	19.27952	9.2315	-1.06243	Down	6.42E-45	4.42E-44
RNF31	3571	1021.5	482	8.332833	3.990897	-1.06209	Down	7.31E-45	5.03E-44
MB21D1	1802	345	161	5.561138	2.663565	-1.06202	Down	1.40E-16	5.54E-16
SAMHD1	3189	2973	1395.5	27.08819	12.97803	-1.06159	Down	########	########
TARS2	2390	934	440	11.35999	5.444217	-1.06116	Down	2.29E-41	1.50E-40
C4orf33	1873	316	148	4.892881	2.345418	-1.06084	Down	3.32E-15	1.26E-14
SESTD1	10448	659	308.5	1.829954	0.877442	-1.06043	Down	3.71E-30	1.98E-29
CAB39L	3691	497.5	233	3.911293	1.875448	-1.06041	Down	5.36E-23	2.46E-22
SLC9A5	3745	516.5	242	3.992576	1.914855	-1.06008	Down	8.48E-24	3.96E-23

TRMT12	2232	404.5	192.5	5.294789	2.53969	-1.05992	Down	1.77E-18	7.33E-18
CNTRL	7431	697	333	2.745356	1.317004	-1.05973	Down	2.13E-30	1.14E-29
UROD	1408	2564	1208.5	52.89992	25.38351	-1.05937	Down	########	########
SNRPA	1646	2608	1225	45.90884	22.03491	-1.05898	Down	########	########
C2orf44	3779	433	203.5	3.315996	1.591789	-1.05879	Down	3.09E-20	1.33E-19
OBFC1	6483	956	450	4.280034	2.054751	-1.05866	Down	2.27E-42	1.51E-41
ZNF326	2729	1240.5	582	13.18917	6.33191	-1.05864	Down	9.06E-55	7.13E-54
TENC1	4690	3503	1652	21.71078	10.4254	-1.05831	Down	########	########
LOC100131091	1240	58	27	1.346778	0.64709	-1.05747	Down	0.000732	0.001523
UGT1A10	2399	329	155.5	3.975215	1.910549	-1.05705	Down	1.37E-15	5.25E-15
TLE4	4749	3385	1592	20.6651	9.937694	-1.05621	Down	########	########
ANKRD9	1626	271	128	4.837968	2.327019	-1.05592	Down	4.89E-13	1.70E-12
PPP2R1B	5470	1480	698	7.848214	3.775899	-1.05554	Down	2.26E-64	2.00E-63
C6orf26	917	56.5	27	1.795507	0.864091	-1.05514	Down	0.001404	0.00286
HGF	2805	29413	13835	303.8201	146.256	-1.05472	Down	0	0
BEX1	862	73	35	2.479162	1.194293	-1.0537	Down	0.000237	0.000512
KIAA0586	5600	841.5	395	4.343619	2.092504	-1.05367	Down	1.20E-37	7.36E-37
FANCI	4749	2702	1271	16.46926	7.93628	-1.05324	Down	########	########
HDDC2	1615	924	435.5	16.60082	8.000553	-1.05308	Down	5.36E-41	3.47E-40
METTL5	956	791	372	23.95919	11.54802	-1.05293	Down	2.05E-35	1.22E-34
UBTF	4603	4324	2040.5	27.25678	13.13854	-1.05281	Down	########	########
METAP2	3506	3176.5	1498.5	26.27934	12.66757	-1.05279	Down	########	########
NDUFB10	741	2640	1248	103.4242	49.86854	-1.05237	Down	########	########
GNB1L	1537	183	87	3.470141	1.673937	-1.05175	Down	3.78E-09	1.03E-08
CDH26	3193	50	25	0.47031	0.226904	-1.05153	Down	0.003836	0.007523

C6orf1	1244	946.5	447.5	22.08534	10.65579	-1.05145	Down	1.29E-41	8.49E-41
NR2F1	3210	13461.5	6377	121.8577	58.80277	-1.05124	Down	0	0
DZIP3	5568	790.5	373	4.114679	1.98588	-1.051	Down	4.37E-35	2.56E-34
F12	2060	160	76	2.266788	1.094161	-1.05082	Down	3.50E-08	9.24E-08
KDM5D	5331	2104	994	11.42778	5.516528	-1.05071	Down	2.09E-90	2.39E-89
C8orf41	2198	789.5	372.5	10.4237	5.031892	-1.0507	Down	3.80E-35	2.24E-34
AATF	2172	2632.5	1244.5	35.17143	16.9804	-1.05053	Down	########	########
OTX1	2872	57	27	0.577349	0.278886	-1.04977	Down	0.001016	0.002091
PRDM10	6318	679.5	323	3.122949	1.508671	-1.04963	Down	6.35E-30	3.37E-29
CKAP5	6534	8905.5	4204.5	39.48052	19.0758	-1.0494	Down	0	0
BARD1	2610	342.5	163	3.817619	1.845788	-1.04844	Down	8.76E-16	3.39E-15
BZW2	1883	2568	1214.5	39.55148	19.12344	-1.04839	Down	########	########
MTMR2	4674	4586.5	2170	28.44186	13.75761	-1.04779	Down	########	########
FBXL19	3814	2143	1020.5	16.34529	7.906593	-1.04775	Down	1.36E-90	1.55E-89
SPSB1	3130	340.5	162	3.158575	1.52807	-1.04756	Down	1.04E-15	4.01E-15
DNTTIP2	2725	3140	1483.5	33.37818	16.16785	-1.04578	Down	########	########
DCLRE1B	3795	743.5	351.5	5.675796	2.749812	-1.04549	Down	4.63E-33	2.62E-32
NFYC	2102	1672.5	793.5	23.08523	11.18598	-1.04528	Down	1.25E-71	1.21E-70
EID2	1409	470.5	222.5	9.662046	4.682126	-1.04517	Down	1.65E-21	7.28E-21
CENPP	3428	376	178.5	3.178516	1.540294	-1.04515	Down	1.95E-17	7.86E-17
ZW10	2900	554.5	262	5.527353	2.67869	-1.04506	Down	5.22E-25	2.51E-24
CAD	7108	3510.5	1665	14.288	6.92534	-1.04485	Down	########	########
RFC2	1613	1925	916	34.66398	16.80211	-1.04479	Down	1.30E-81	1.37E-80
ENAH	13172	9646	4574.5	21.23804	10.2971	-1.04441	Down	0	0
FZD9	2342	116	55.5	1.446223	0.701299	-1.04419	Down	2.60E-06	6.30E-06

GPAM	6390	1018	483	4.619176	2.241528	-1.04315	Down	2.86E-44	1.96E-43
FUBP1	2884	4135.5	1964.5	41.59411	20.18595	-1.04303	Down	########	########
TAF6	2458	4365.5	2080.5	51.63318	25.06363	-1.0427	Down	########	########
MTERFD1	1424	802	381	16.33692	7.933535	-1.0421	Down	3.83E-35	2.25E-34
SLIRP	436	1015	481	67.32738	32.70594	-1.04164	Down	2.96E-44	2.02E-43
MRPS30	1686	1132.5	537.5	19.45554	9.451207	-1.04161	Down	4.75E-49	3.46E-48
DNAJC17	1031	280	133.5	7.88711	3.831893	-1.04144	Down	2.82E-13	9.93E-13
STXBP4	6046	386.5	183.5	1.853895	0.900797	-1.04129	Down	8.28E-18	3.36E-17
PEX14	1945	336.5	161.5	5.046764	2.453458	-1.04054	Down	2.28E-15	8.69E-15
CMKLR1	5283	167.5	82.5	0.93352	0.453832	-1.04052	Down	5.70E-08	1.49E-07
VPS36	4427	1531	727	10.02541	4.876631	-1.0397	Down	1.42E-65	1.28E-64
DHFRL1	4047	344.5	164	2.469345	1.201958	-1.03874	Down	7.38E-16	2.86E-15
SET	2936	15538.5	7379	153.2857	74.62136	-1.03856	Down	0	0
ARHGAP28	5542	161	77.5	0.847619	0.412728	-1.03823	Down	4.02E-08	1.06E-07
GCNT3	2240	162	80.5	2.141839	1.042933	-1.0382	Down	1.09E-07	2.82E-07
NDOR1	4726	1578	754.5	9.691157	4.718983	-1.03819	Down	1.22E-66	1.11E-65
DCK	2618	1277.5	607.5	14.14257	6.889748	-1.03752	Down	7.11E-55	5.60E-54
CCNE1	2021	594	283.5	8.527656	4.15462	-1.03743	Down	2.69E-26	1.32E-25
PPM1G	2265	4952	2360	63.43944	30.91437	-1.0371	Down	########	########
TSPYL1	5259	2423	1160.5	13.39819	6.529395	-1.03702	Down	########	########
GPRIN1	4208	531	254.5	3.659585	1.783789	-1.03673	Down	1.75E-23	8.11E-23
NCL	2732	25577.5	12195	271.5939	132.4041	-1.0365	Down	0	0
PACSIN3	1913	483.5	230.5	7.318826	3.568027	-1.03649	Down	1.06E-21	4.69E-21
GFER	2447	930	445.5	11.05535	5.390015	-1.03638	Down	7.12E-40	4.52E-39
FKBP3	1353	1352.5	644.5	28.94958	14.11598	-1.03621	Down	9.80E-58	8.01E-57

KLHL8	4576	1208.5	576.5	7.65911	3.734844	-1.03613	Down	1.16E-51	8.76E-51
L3MBTL3	4217	435	207.5	2.989448	1.457965	-1.03592	Down	1.00E-19	4.25E-19
LAS1L	2448	1810	864	21.45027	10.46335	-1.03565	Down	2.49E-76	2.51E-75
ARAP2	7375	374.5	179.5	1.474228	0.719483	-1.03493	Down	5.61E-17	2.24E-16
PGPEP1	7084	1320	632.5	5.421658	2.64606	-1.03489	Down	8.55E-56	6.82E-55
TYRP1	2876	4542	2165.5	45.70714	22.31351	-1.0345	Down	########	########
EBAG9	1655	483	230.5	8.468664	4.135063	-1.03423	Down	1.06E-21	4.69E-21
PSMB6	849	3137	1505.5	107.3542	52.42272	-1.03411	Down	########	########
NSMAF	3522	2978	1420.5	24.49696	11.96237	-1.0341	Down	########	########
PRIC285	10002	4002	1922.5	11.62822	5.67883	-1.03396	Down	########	########
HOXA3	3396	1227.5	586.5	10.48372	5.120067	-1.03392	Down	2.88E-52	2.19E-51
UBLCP1	2410	2610.5	1245	31.36937	15.32286	-1.03367	Down	########	########
ADSL	1388	1255	601	26.22426	12.81542	-1.03302	Down	4.12E-53	3.17E-52
APOBEC3A	1103	137	66	3.604466	1.762453	-1.0322	Down	5.13E-07	1.28E-06
C17orf80	3545	1098	526	8.995865	4.399951	-1.03177	Down	1.19E-46	8.38E-46
KIAA0664	5252	4041	1943.5	22.38511	10.94959	-1.03166	Down	########	########
DTX3	2029	297.5	143	4.256442	2.083587	-1.03058	Down	1.26E-13	4.55E-13
KIAA1826	4095	850.5	409	6.030782	2.953434	-1.02995	Down	3.70E-36	2.21E-35
SQSTM1	2931	40787	19585	403.933	197.8227	-1.02991	Down	0	0
SLC6A15	4362	107	51.5	0.71125	0.348371	-1.02973	Down	7.23E-06	1.71E-05
GEMIN2	1368	206.5	99.5	4.403665	2.15757	-1.0293	Down	6.40E-10	1.81E-09
MMP28	2480	140	67	1.633654	0.800434	-1.02925	Down	3.16E-07	7.96E-07
EXOSC2	2194	945.5	453.5	12.50968	6.131378	-1.02876	Down	2.47E-40	1.58E-39
SPTBN2	7884	453	219	1.678176	0.822571	-1.02868	Down	7.94E-20	3.39E-19
PTCH2	3773	71	34.5	0.549169	0.269212	-1.02851	Down	0.000285	0.000611

FRAT1	2651	52	25	0.568487	0.278695	-1.02844	Down	0.002031	0.004083
MORC2	4467	1455	700.5	9.470585	4.646319	-1.02737	Down	1.10E-60	9.33E-60
LSM5	2212	2464	1179	32.24914	15.82175	-1.02735	Down	########	########
AR	4314	874.5	421	5.887419	2.888602	-1.02726	Down	4.78E-37	2.90E-36
MAGEA10	1610	60.5	29.5	1.096308	0.537957	-1.02709	Down	0.000973	0.002006
NARFL	2109	735	355	10.11536	4.964534	-1.02682	Down	3.06E-31	1.67E-30
TALDO1	1319	9425	4529.5	207.1676	101.7281	-1.02608	Down	0	0
AMIGO1	2501	116	56	1.34648	0.661352	-1.0257	Down	4.08E-06	9.78E-06
TCF3	4462	5213.5	2515	33.97483	16.68752	-1.0257	Down	########	########
TXN2	1342	1538	741.5	33.29962	16.36064	-1.02528	Down	7.66E-64	6.71E-63
PECAM1	6831	947	456	4.029906	1.980088	-1.02518	Down	4.96E-40	3.16E-39
NDUFA9	1621	2104.5	1012.5	37.59056	18.48648	-1.0239	Down	5.59E-87	6.21E-86
NETO2	3692	3794.5	1827	29.80869	14.66294	-1.02356	Down	########	########
KRBA2	1964	56	27	0.826547	0.406728	-1.02303	Down	0.001404	0.002861
USP49	3194	81	38.5	0.728958	0.358717	-1.02299	Down	7.29E-05	0.000163
DNAJC19	1488	601.5	288.5	11.71605	5.766443	-1.02273	Down	2.85E-26	1.41E-25
CSMD1	14340	88	44	0.181915	0.089544	-1.0226	Down	0.000119	0.000261
DUT	1874	2172.5	1044.5	33.5968	16.53768	-1.02257	Down	7.31E-90	8.30E-89
PTPLB	1187	728	351.5	17.84354	8.78489	-1.02231	Down	4.48E-31	2.43E-30
MRPS9	1473	616.5	296.5	12.11192	5.965727	-1.02166	Down	9.86E-27	4.91E-26
CCDC56	772	754	364	28.376	13.98121	-1.02118	Down	4.87E-32	2.70E-31
SLC2A8	2145	278.5	134.5	3.76341	1.854293	-1.02117	Down	8.10E-13	2.77E-12
NFIB	8507	2108	1016	7.178916	3.537425	-1.02107	Down	9.43E-87	1.05E-85
ALAS1	2258	2730	1318	35.09244	17.29257	-1.02101	Down	########	########
GTF3C6	957	1974.5	952.5	59.92711	29.53934	-1.02057	Down	3.02E-81	3.18E-80

KIF20B	6319	2381.5	1145.5	10.91281	5.379717	-1.02042	Down	3.17E-98	3.82E-97
FANCA	1673	1243	601	21.56895	10.63399	-1.02027	Down	1.54E-51	1.16E-50
FAM86A	2374	821.5	397	10.02199	4.941354	-1.02019	Down	1.29E-34	7.52E-34
CTPS	3217	4417.5	2129.5	39.78515	19.61662	-1.02015	Down	########	########
PSMB10	1019	3685.5	1787	105.0981	51.84895	-1.01935	Down	########	########
PLEKHH3	3076	1186	575.5	11.21293	5.531831	-1.01933	Down	5.75E-49	4.19E-48
SKA3	2808	1230.5	593.5	12.71417	6.272986	-1.01921	Down	2.43E-51	1.83E-50
RCC2	4109	4792.5	2314.5	33.84361	16.70321	-1.01876	Down	########	########
TRDMT1	7687	345	166	1.2938	0.638741	-1.01831	Down	1.31E-15	5.03E-15
FAM155A	3859	52	25.5	0.393504	0.194274	-1.01829	Down	0.002031	0.004082
MICB	2497	982	477	11.4435	5.651251	-1.01789	Down	1.13E-40	7.28E-40
POLR1B	5427	1198.5	582	6.416159	3.169532	-1.01744	Down	3.17E-49	2.32E-48
ZFP14	7502	193	95.5	0.75552	0.373435	-1.01661	Down	5.86E-09	1.59E-08
C16orf87	1410	660	319.5	13.58289	6.714226	-1.0165	Down	3.54E-28	1.82E-27
ZNRF3	6696	412	201	1.792949	0.886485	-1.01617	Down	7.86E-18	3.20E-17
ADNP	4713	4343.5	2111	26.77437	13.23978	-1.01597	Down	########	########
DPF1	2329	258	125.5	3.228192	1.596336	-1.01596	Down	7.05E-12	2.22E-11
DGAT2	2485	368.5	179	4.301801	2.127739	-1.01562	Down	3.46E-16	1.35E-15
ZNF260	5341	970.5	473.5	5.289248	2.616527	-1.01541	Down	7.37E-40	4.68E-39
USF1	1800	1718.5	834.5	27.74404	13.72552	-1.01532	Down	6.22E-70	5.88E-69
IMP4	1074	1567	760.5	42.44632	21.00151	-1.01515	Down	4.49E-64	3.95E-63
CHERP	4105	2722	1320	19.25974	9.531463	-1.01482	Down	########	########
EXOSC10	2759	2610	1264.5	27.44864	13.58434	-1.01479	Down	########	########
FEZ1	1781	100.5	49	1.651674	0.817448	-1.01473	Down	2.63E-05	6.03E-05
OXA1L	1737	2031	986.5	33.97662	16.81941	-1.01442	Down	2.51E-82	2.67E-81

DUS4L	2252	524	254.5	6.754931	3.344244	-1.01426	Down	1.46E-22	6.61E-22
ZNF319	4220	813.5	395.5	5.605693	2.77529	-1.01425	Down	6.02E-34	3.47E-33
SGOL1	2355	733	356	9.033057	4.472208	-1.01423	Down	8.57E-31	4.62E-30
FZD6	3806	2301	1114.5	17.52886	8.682869	-1.01349	Down	1.36E-93	1.59E-92
SLC25A29	2370	768	374	9.405192	4.659041	-1.01342	Down	5.33E-32	2.95E-31
MMS22L	8643	773.5	376	2.595973	1.286403	-1.01293	Down	2.81E-32	1.56E-31
RBBP9	3871	1397	678.5	10.46382	5.185295	-1.01291	Down	3.11E-57	2.52E-56
PDZRN3	4161	189.5	93	1.32984	0.658996	-1.01291	Down	8.31E-09	2.25E-08
DEM1	2217	69.5	34	0.911569	0.451957	-1.01217	Down	0.000534	0.001125
FASTKD1	3020	765.5	370	7.321686	3.630757	-1.01191	Down	2.36E-32	1.31E-31
ETFA	1369	4284	2076	90.70411	44.98595	-1.01169	Down	########	########
SRI	2030	3836	1861.5	54.80877	27.19141	-1.01126	Down	########	########
C16orf88	1974	947.5	462	13.95367	6.927651	-1.01021	Down	6.49E-39	4.05E-38
KIAA0922	5034	719	351	4.153421	2.06246	-1.00993	Down	6.65E-30	3.53E-29
LSM6	789	623	301	22.84347	11.34695	-1.00948	Down	1.04E-26	5.17E-26
DARS2	3348	1810.5	879	15.66596	7.783196	-1.0092	Down	1.62E-73	1.59E-72
FAM92A1	1135	1003	488	25.63775	12.73892	-1.00903	Down	2.33E-41	1.52E-40
GTPBP3	2549	353.5	172.5	4.029476	2.002528	-1.00877	Down	1.56E-15	5.98E-15
SNX18	3259	2201	1070	19.59622	9.740247	-1.00854	Down	8.63E-89	9.71E-88
CABYR	2279	108	53.5	1.38466	0.688394	-1.00823	Down	1.29E-05	3.00E-05
SUCLG1	1475	1981	965	38.94312	19.36259	-1.0081	Down	9.65E-80	1.00E-78
MPHOSPH9	3624	624	303	4.982026	2.47728	-1.00798	Down	1.82E-26	8.99E-26
THAP10	2119	168	81.5	2.290139	1.139105	-1.00753	Down	2.72E-08	7.21E-08
PPIF	2213	3325	1621.5	43.61214	21.69329	-1.00748	Down	########	########
MPP1	2016	782	381	11.24833	5.59568	-1.00733	Down	1.61E-32	9.01E-32

JAKMIP3	6488	122	59.5	0.544865	0.271203	-1.00653	Down	2.40E-06	5.82E-06
SSRP1	2825	8212.5	4000	84.30189	41.97516	-1.00603	Down	0	0
DPH5	1828	933	455	14.79956	7.369807	-1.00586	Down	2.14E-38	1.33E-37
SHKBP1	2379	2377.5	1163	29.0757	14.47897	-1.00585	Down	2.26E-94	2.65E-93
PHC1	5206	1007	490.5	5.606528	2.792161	-1.00573	Down	1.66E-41	1.09E-40
EXOSC3	1070	955	465	25.86322	12.88371	-1.00535	Down	2.14E-39	1.35E-38
RIOK1	1796	890	433.5	14.36406	7.156474	-1.00514	Down	6.80E-37	4.12E-36
DDX11	3797	1622	792	12.38644	6.171275	-1.00512	Down	2.81E-65	2.52E-64
BAI2	5434	1245.5	609.5	6.650671	3.315376	-1.00433	Down	2.55E-50	1.89E-49
PRKD2	2939	1526.5	749	15.11625	7.536575	-1.00412	Down	8.20E-61	6.96E-60
ATR	8258	1867	911	6.549641	3.2655	-1.00411	Down	5.61E-75	5.58E-74
DUS2L	1999	489	240	7.111782	3.546249	-1.00392	Down	1.30E-20	5.65E-20
C7orf49	1589	1803	881	32.92887	16.42107	-1.00381	Down	3.03E-72	2.94E-71
KANK2	4966	4311.5	2111	25.23685	12.5889	-1.00338	Down	########	########
AIP	1250	1866	912.5	43.3592	21.6331	-1.0031	Down	1.15E-74	1.14E-73
DDX20	3513	1105.5	540.5	9.133828	4.557169	-1.00308	Down	5.94E-45	4.09E-44
PFDN2	668	2370	1155.5	102.786	51.29713	-1.00269	Down	6.12E-95	7.23E-94
ZNHIT2	1321	318.5	157	7.019823	3.503576	-1.00261	Down	9.23E-14	3.34E-13
NDUFS3	971	1575.5	770.5	47.1224	23.52271	-1.00236	Down	2.90E-63	2.52E-62
RARRES3	779	554.5	272	20.63571	10.30287	-1.0021	Down	3.99E-23	1.84E-22
TRIM45	3527	228.5	114.5	1.903996	0.95072	-1.00194	Down	5.20E-10	1.48E-09
TGIF2	3358	567	277.5	4.905776	2.450256	-1.00155	Down	6.94E-24	3.25E-23
RALGPS1	2362	101.5	50	1.252362	0.625589	-1.00137	Down	2.98E-05	6.82E-05
C19orf20	1134	415	204	10.62438	5.307844	-1.00118	Down	1.15E-17	4.64E-17
LYAR	1584	957.5	468.5	17.56189	8.774151	-1.00112	Down	4.22E-39	2.64E-38

Supplementary Table 2

GeneID	Gene_length	C6-DMSO-Expression	C6-331-Expression	C6-DMSO-RPKM	C6-331-RPKM	log2 Ratio(C6-331/C6-DMSO)	Up-Down-Regulation (331/DMSO)	P-value	FDR
Cxcl2	1101	0	1803	0.001	54.67023	15.73847	Up	0	0
Otor	919	0	499	0.001	18.11275	14.14472	Up	0	0
LOC100909799	819	0	443	0.001	17.57221	14.10101	Up	0	0
Il22ra2	2506	0	950.5	0.001	12.6595	13.62793	Up	0	0
Arl9	645	0	220	0.001	11.39473	13.47608	Up	0	0
LOC100359479	768	0	259.5	0.001	11.29105	13.46289	Up	0	0
LOC367195	457	0	138	0.001	10.10346	13.30256	Up	0	0
Olr1646	945	0	236	0.001	8.339775	13.02579	Up	0	0
LOC100912293	528	0	110	0.001	6.955586	12.76396	Up	0	0
LOC100912893	1965	0	406.5	0.001	6.904676	12.75336	Up	0	0
Nox4	2176	0	428.5	0.001	6.562775	12.68009	Up	0	0
RGD1561410	618	0	112	0.001	6.041946	12.5608	Up	0	0
Ccl20	816	0	125.5	0.001	5.13541	12.32626	Up	0	0
LOC100910820	1527	0	221.5	0.001	4.712391	12.20224	Up	0	0
Uts2d	747	0	98.5	0.001	4.398194	12.1027	Up	0	0
Lyz2	1223	0	156	0.001	4.243943	12.05119	Up	0	0
S100a7a	581	0	65	0.001	3.730538	11.86517	Up	0	0
C1qtnf4	1348	0	144.5	0.001	3.563905	11.79924	Up	0	0
Ptcra	621	0	59.5	0.001	3.180781	11.63517	Up	0	0
LOC100359989	649	0	60	0.001	3.07274	11.58531	Up	0	0

LOC679753	302	0	26.5	0.001	2.931128	11.51724	Up	1.49E-08	3.43E-08
Wfikkn2	1608	0	140.5	0.001	2.905719	11.50468	Up	0	0
Asb11	1317	0	103	0.001	2.613851	11.35196	Up	0	0
Alox15	2216	0	172	0.001	2.588146	11.3377	Up	0	0
LOC100912017	2709	0	207.5	0.001	2.550835	11.31675	Up	0	0
LOC100910784	588	0	42.5	0.001	2.348109	11.19728	Up	2.27E-13	8.19E-13
LOC100911329	570	0	38	0.001	2.165785	11.08067	Up	3.64E-12	1.03E-11
Cyp2b21	1885	0	121.5	0.001	2.149362	11.06969	Up	0	0
LOC100362620	240	0	15.5	0.001	2.131856	11.05789	Up	3.05E-05	5.93E-05
Lcn11	669	0	42	0.001	2.08527	11.02602	Up	2.27E-13	8.19E-13
RGD1559679	403	0	25	0.001	2.073376	11.01777	Up	2.98E-08	6.78E-08
LOC363746	619	0	38	0.001	2.052502	11.00317	Up	3.64E-12	1.03E-11
RGD1562229	921	0	56	0.001	2.014398	10.97613	Up	0	0
RGD1559980	364	0	22	0.001	2.007994	10.97154	Up	2.38E-07	5.24E-07
LOC100909551	1518	0	91	0.001	2.000856	10.9664	Up	0	0
LOC100909765	582	0	35	0.001	1.953672	10.93197	Up	2.91E-11	7.39E-11
LOC500007	657	0	37.5	0.001	1.893996	10.88722	Up	7.28E-12	1.94E-11
Sh2d5	1386	0	78.5	0.001	1.880241	10.8767	Up	0	0
RGD1562344	850	0	47	0.001	1.855624	10.85769	Up	7.11E-15	2.97E-14
Dand5	555	0	30	0.001	1.801449	10.81494	Up	9.31E-10	2.24E-09
Krtap3-3l1	713	0	38.5	0.001	1.795851	10.81045	Up	3.64E-12	1.03E-11
Cuzd1	2282	0	121	0.001	1.76753	10.78752	Up	0	0
Tll2	3045	0	161	0.001	1.763802	10.78447	Up	0	0
Gpr151	1395	0	73.5	0.001	1.761993	10.78299	Up	0	0
LOC100911244	722	0	39	0.001	1.754826	10.77711	Up	1.82E-12	5.48E-12

LOC688802	1167	0	61	0.001	1.736671	10.76211	Up		0	0
Lypd6b	2326	0	120	0.001	1.712389	10.74179	Up		0	0
Gzmm	952	0	48.5	0.001	1.692868	10.72525	Up		3.55E-15	1.49E-14
RGD1564897	570	0	29	0.001	1.6781	10.71261	Up		1.86E-09	4.43E-09
LOC300024	848	0	40.5	0.001	1.591882	10.63652	Up		9.09E-13	2.89E-12
LOC683581	694	0	33	0.001	1.587557	10.63259	Up		1.16E-10	2.89E-10
LOC100911585	1484	0	67.5	0.001	1.51163	10.56189	Up		0	0
LOC365062	1356	0	61	0.001	1.502578	10.55322	Up		0	0
LOC100362780	1008	0	44.5	0.001	1.475261	10.52675	Up		5.68E-14	2.25E-13
L3mbtl1	2481	0	108.5	0.001	1.458815	10.51058	Up		0	0
LOC100911132	2442	0	108	0.001	1.436762	10.48861	Up		0	0
Il23r	1920	0	81	0.001	1.411787	10.46331	Up		0	0
Crygs	696	0	29	0.001	1.39241	10.44337	Up		1.86E-09	4.43E-09
RGD1306626	1295	0	53.5	0.001	1.381733	10.43226	Up		0	0
Serpina7	1748	0	72	0.001	1.37623	10.42651	Up		0	0
Slc8a2	3004	0	123	0.001	1.361943	10.41145	Up		0	0
Spint3	441	0	18	0.001	1.356604	10.40578	Up		3.81E-06	7.86E-06
LOC683684	615	0	24	0.001	1.3029	10.34751	Up		5.96E-08	1.34E-07
Hic1	3411	0	128	0.001	1.249432	10.28706	Up		0	0
Spic	1133	0	42	0.001	1.240818	10.27708	Up		2.27E-13	8.19E-13
RGD1559917	327	0	12	0.001	1.219699	10.25231	Up		0.000244	0.00044
Glyatl3	873	0	32	0.001	1.215553	10.2474	Up		2.33E-10	5.71E-10
Glt8d4	1832	0	66	0.001	1.19887	10.22746	Up		0	0
Tac2	727	0	26	0.001	1.184122	10.2096	Up		1.49E-08	3.43E-08
LOC684709	909	0	32	0.001	1.179294	10.20371	Up		2.33E-10	5.71E-10

Tmprss4	2235	0	79	0.001	1.177297	10.20126	Up	0	0
Ak5	955	0	33	0.001	1.154623	10.17321	Up	1.16E-10	2.89E-10
LOC100909507	495	0	17.5	0.001	1.148522	10.16556	Up	7.63E-06	1.54E-05
LOC100911106	611	0	21	0.001	1.13719	10.15126	Up	4.77E-07	1.03E-06
LOC100912067	1038	0	34.5	0.001	1.110978	10.11761	Up	5.82E-11	1.46E-10
Ccl4	279	0	9	0.001	1.060864	10.05102	Up	0.001953	0.003246
Tnni1	821	0	26	0.001	1.055124	10.0432	Up	1.49E-08	3.43E-08
Angptl4	1871	1	1108.5	0.018819	19.79563	10.0388	Up	0.000575	0.001004
Krtap1-5	369	0	11.5	0.001	1.029534	10.00778	Up	0.000488	0.000864
Olr837	948	0	28.5	0.001	1.017484	9.99079	Up	3.73E-09	8.78E-09
C4bpb	1144	0	34.5	0.001	1.005677	9.973952	Up	5.82E-11	1.46E-10
Klk9	1032	0	31	0.001	1.003771	9.971214	Up	4.66E-10	1.13E-09
Fam24a	571	0	17	0.001	1.001884	9.9685	Up	7.63E-06	1.54E-05
LOC100910954	749	0	23	0.001	0.997591	9.962305	Up	1.19E-07	2.65E-07
RGD1563606	609	0	18	0.001	0.99567	9.959523	Up	3.81E-06	7.85E-06
LOC100361147	435	0	13	0.001	0.99156	9.953556	Up	0.000122	0.000224
LOC100910525	1401	0	41	0.001	0.982841	9.940814	Up	4.55E-13	1.54E-12
Fgf17	651	0	19	0.001	0.975806	9.93045	Up	1.91E-06	3.99E-06
Tprg1	1103	0	32	0.001	0.968611	9.919774	Up	2.33E-10	5.71E-10
Atp13a4	3901	0	112	0.001	0.957632	9.903328	Up	0	0
LOC100912135	692	0	20	0.001	0.938924	9.874865	Up	9.54E-07	2.03E-06
Ccdc116	1991	0	55.5	0.001	0.925473	9.854048	Up	0	0
Amy1a	1639	0	45	0.001	0.918308	9.842834	Up	2.84E-14	1.16E-13
LOC688823	1410	0	38	0.001	0.904893	9.821603	Up	3.64E-12	1.03E-11
LOC100911739	777	0	21	0.001	0.904663	9.821237	Up	4.77E-07	1.03E-06

Ppp1r42	1426	0	38.5	0.001	0.899188	9.812479	Up	3.64E-12	1.03E-11
LOC100911047	332	0	9	0.001	0.896931	9.808854	Up	0.001953	0.003244
Tmem190	537	0	14	0.001	0.87042	9.765567	Up	6.10E-05	0.000116
LOC100362253	789	0	20.5	0.001	0.870317	9.765396	Up	9.54E-07	2.03E-06
LOC681827	927	0	24	0.001	0.866325	9.758765	Up	5.96E-08	1.34E-07
LOC100909803	783	0	20	0.001	0.848194	9.728251	Up	9.54E-07	2.03E-06
Fam217a	1876	0	46	0.001	0.818653	9.677109	Up	1.42E-14	5.86E-14
LOC687707	702	0	17	0.001	0.812359	9.665973	Up	7.63E-06	1.54E-05
Lyc2	1601	0	38.5	0.001	0.803149	9.649525	Up	3.64E-12	1.03E-11
RGD1304622	4254	0	102	0.001	0.801799	9.647098	Up	0	0
Tnfrsf18	1029	0	24.5	0.001	0.790988	9.627512	Up	5.96E-08	1.34E-07
LOC683536	459	0	10.5	0.001	0.780418	9.608103	Up	0.000977	0.001662
Fam186b	3140	0	72.5	0.001	0.77417	9.596507	Up	0	0
LOC681354	741	0	17	0.001	0.767174	9.58341	Up	7.63E-06	1.54E-05
Vom1r73	918	0	21	0.001	0.76277	9.575105	Up	4.77E-07	1.03E-06
LOC691189	471	0	10.5	0.001	0.75098	9.552631	Up	0.000977	0.001658
Tmem182	2708	0	60	0.001	0.741067	9.53346	Up	0	0
LOC100911540	1079	1	780.5	0.032632	23.50031	9.492182	Up	0.000575	0.001009
Cplx4	613	0	13	0.001	0.706572	9.464692	Up	0.000122	0.000224
Ctage5	2027	0	42.5	0.001	0.702906	9.457187	Up	2.27E-13	8.19E-13
Ptger3	1253	0	26.5	0.001	0.702873	9.457121	Up	1.49E-08	3.43E-08
LOC498446	1335	0	28	0.001	0.700922	9.45311	Up	3.73E-09	8.78E-09
LOC100363341	1816	0	38	0.001	0.691684	9.43397	Up	3.64E-12	1.03E-11
Klk8	1302	0	27	0.001	0.687514	9.425246	Up	7.45E-09	1.74E-08
LOC100363124	1041	0	21	0.001	0.667457	9.382531	Up	4.77E-07	1.03E-06

Serpina3k	1700	0	33.5	0.001	0.658711	9.363501	Up		1.16E-10	2.89E-10
Lbx2	816	0	16	0.001	0.656849	9.359419	Up		1.53E-05	3.03E-05
LOC683129	1419	0	27.5	0.001	0.654325	9.353864	Up		7.45E-09	1.74E-08
Adcy1	3219	0	63	0.001	0.653703	9.352491	Up		0	0
Pvalb	563	0	11	0.001	0.650719	9.345891	Up		0.000488	0.000863
Olr836	960	0	18	0.001	0.635378	9.311472	Up		3.81E-06	7.85E-06
LOC100909834	2728	0	52	0.001	0.633766	9.307807	Up		2.22E-16	9.52E-16
RGD1561147	927	0	17	0.001	0.617126	9.269422	Up		7.63E-06	1.54E-05
Sdr42e2	1251	0	23	0.001	0.615985	9.266751	Up		1.19E-07	2.65E-07
LOC100910896	1114	0	21	0.001	0.612408	9.258349	Up		4.77E-07	1.03E-06
Asb9	963	0	17.5	0.001	0.609989	9.252639	Up		7.63E-06	1.54E-05
Oxtr	1167	0	21	0.001	0.598477	9.225153	Up		4.77E-07	1.03E-06
LOC689621	453	0	8	0.001	0.585639	9.193868	Up		0.003906	0.006347
Mael	1559	0	27.5	0.001	0.585175	9.192723	Up		7.45E-09	1.74E-08
LOC288930	1197	0	21	0.001	0.584982	9.192248	Up		4.77E-07	1.03E-06
Wee2	1674	0	29	0.001	0.583225	9.187908	Up		1.86E-09	4.43E-09
B4galnt4	3546	0	62	0.001	0.581213	9.182924	Up		0	0
LOC690352	976	0	17	0.001	0.58061	9.181427	Up		7.63E-06	1.54E-05
LOC100362817	748	0	13	0.001	0.579049	9.177541	Up		0.000122	0.000225
Tdrd5	3393	0	58.5	0.001	0.576828	9.171998	Up		0	0
Oaz3	878	0	15	0.001	0.573465	9.163562	Up		3.05E-05	5.93E-05
Tmed6	867	0	14.5	0.001	0.556815	9.121055	Up		6.10E-05	0.000116
Zyg11a	2283	0	37.5	0.001	0.547418	9.096499	Up		7.28E-12	1.94E-11
Ppp1r27	789	0	13	0.001	0.542114	9.082454	Up		0.000122	0.000224
Cyp2c11	1856	0	30	0.001	0.541597	9.081077	Up		9.31E-10	2.24E-09

RGD1564744	483	0	8	0.001	0.539947	9.076673	Up	0.003906	0.006326
Slc13a3	3206	0	52	0.001	0.539836	9.076377	Up	2.22E-16	9.52E-16
Gpbar1	990	0	16	0.001	0.537767	9.070836	Up	1.53E-05	3.03E-05
Slc9a10	3902	0	63	0.001	0.536973	9.068706	Up	0	0
Olr1587	939	0	15	0.001	0.532377	9.056305	Up	3.05E-05	5.93E-05
Olr1387	936	0	15	0.001	0.530237	9.050494	Up	3.05E-05	5.93E-05
Adam2	2431	0	38.5	0.001	0.529676	9.048966	Up	3.64E-12	1.03E-11
Tal2	1170	0	18	0.001	0.519797	9.021805	Up	3.81E-06	7.85E-06
Stk32b	1245	0	19	0.001	0.508795	8.99094	Up	1.91E-06	3.99E-06
LOC100912184	864	0	13	0.001	0.507556	8.987424	Up	0.000122	0.000225
LOC100911068	3264	0	49.5	0.001	0.505636	8.981956	Up	1.78E-15	7.51E-15
Pou2f3	2407	0	37	0.001	0.505364	8.981179	Up	7.28E-12	1.94E-11
Tmem40	609	0	9	0.001	0.500791	8.968064	Up	0.001953	0.003247
Mrgprb2	939	0	14	0.001	0.49778	8.959364	Up	6.10E-05	0.000116
RGD1564854	1146	0	17	0.001	0.492911	8.945183	Up	7.63E-06	1.54E-05
Tnfrsf13b	817	0	12	0.001	0.492585	8.944228	Up	0.000244	0.00044
RGD1561282	691	0	10	0.001	0.488377	8.93185	Up	0.000977	0.00166
Clnk	1317	0	19	0.001	0.487813	8.930184	Up	1.91E-06	3.99E-06
LOC367365	717	0	10.5	0.001	0.480769	8.9092	Up	0.000977	0.001661
LOC100910205	1305	0	19	0.001	0.479884	8.906543	Up	1.91E-06	3.99E-06
LOC100909622	1893	0	27	0.001	0.475723	8.893978	Up	7.45E-09	1.74E-08
Grk1	2157	0	30.5	0.001	0.473968	8.888644	Up	9.31E-10	2.24E-09
LOC100911567	813	0	11.5	0.001	0.470601	8.878359	Up	0.000488	0.000864
Pbp2	1295	1	509.5	0.028075	13.14288	8.870798	Up	0.000575	0.001011
Olr1585	939	0	13	0.001	0.467017	8.86733	Up	0.000122	0.000224

LOC686932	2643	0	37	0.001	0.466368	8.865327	Up	7.28E-12	1.94E-11
LOC366782	780	0	11	0.001	0.466224	8.86488	Up	0.000488	0.000864
Acrv1	827	0	11.5	0.001	0.462634	8.853727	Up	0.000488	0.000865
Dgat2l6	1386	0	19	0.001	0.46093	8.848405	Up	1.91E-06	3.99E-06
Far2	1783	0	24	0.001	0.448392	8.808618	Up	5.96E-08	1.34E-07
Apol2	1298	0	17.5	0.001	0.447703	8.8064	Up	7.63E-06	1.54E-05
Olr816	933	0	12.5	0.001	0.444893	8.797314	Up	0.000244	0.00044
LOC100910615	981	0	13	0.001	0.443352	8.79231	Up	0.000122	0.000224
Mrc1	5091	0	67	0.001	0.442215	8.788604	Up	0	0
Pde6a	4104	0	54	0.001	0.440835	8.784096	Up	0	0
LOC100911359	960	0	12.5	0.001	0.438943	8.77789	Up	0.000244	0.00044
Ccl22	1770	0	23	0.001	0.438416	8.776158	Up	1.19E-07	2.65E-07
Prss47	1373	0	18	0.001	0.438356	8.775959	Up	3.81E-06	7.86E-06
LOC686143	987	0	13	0.001	0.43701	8.771521	Up	0.000122	0.000224
Il1b	1339	0	17	0.001	0.424553	8.729799	Up	7.63E-06	1.54E-05
Kb23	1668	0	21	0.001	0.423036	8.724635	Up	4.77E-07	1.03E-06
Gpr55	960	0	12	0.001	0.42296	8.724379	Up	0.000244	0.000441
LOC681107	825	0	10.5	0.001	0.422196	8.721768	Up	0.000977	0.001657
Mc5r	1034	0	13	0.001	0.418886	8.710415	Up	0.000122	0.000224
Fam209a	640	0	8	0.001	0.417335	8.705063	Up	0.003906	0.006324
Ptprh	3407	0	41.5	0.001	0.406282	8.666337	Up	4.55E-13	1.54E-12
LOC100910452	731	0	9	0.001	0.399974	8.643762	Up	0.001953	0.003238
RGD1308742	1003	0	12	0.001	0.397649	8.635351	Up	0.000244	0.00044
Ngp	1099	0	13	0.001	0.397387	8.634402	Up	0.000122	0.000224
Efcab8	3435	0	41	0.001	0.396145	8.629886	Up	4.55E-13	1.54E-12

Muc3a	1772	0	21	0.001	0.393128	8.618855	Up	4.77E-07	1.03E-06
LOC100360320	861	0	10	0.001	0.391949	8.614523	Up	0.000977	0.001659
Tmem156	1534	0	18	0.001	0.391762	8.613833	Up	3.81E-06	7.86E-06
RGD1560880	761	0	9	0.001	0.391303	8.612141	Up	0.001953	0.003245
Gpr115	2214	0	26	0.001	0.387198	8.596928	Up	1.49E-08	3.43E-08
Gzma	786	0	9	0.001	0.385727	8.591436	Up	0.001953	0.003237
Nckap5	5590	0	64.5	0.001	0.384508	8.586869	Up	0	0
LOC100911005	680	0	8	0.001	0.383521	8.583162	Up	0.003906	0.006332
Serpinb3a	1673	0	19	0.001	0.380783	8.572824	Up	1.91E-06	3.99E-06
Nts	798	0	9	0.001	0.379927	8.569577	Up	0.001953	0.003241
Robo4	2886	0	32	0.001	0.370193	8.532135	Up	2.33E-10	5.71E-10
Grik4	3312	0	37	0.001	0.368904	8.527103	Up	7.28E-12	1.94E-11
Tigit	726	0	8	0.001	0.367899	8.523165	Up	0.003906	0.006336
Cd40	1272	0	14	0.001	0.367465	8.521463	Up	6.10E-05	0.000116
LOC299282	1882	0	20.5	0.001	0.36678	8.518771	Up	9.54E-07	2.03E-06
Vom1r77	918	0	10	0.001	0.365652	8.514326	Up	0.000977	0.00166
Hsd17b3	1111	0	12	0.001	0.360614	8.49431	Up	0.000244	0.000441
Fam132b	1026	0	11	0.001	0.36058	8.494175	Up	0.000488	0.000865
Prss39	1595	0	17	0.001	0.359797	8.491039	Up	7.63E-06	1.54E-05
Capn11	2650	0	28.5	0.001	0.359575	8.49015	Up	3.73E-09	8.78E-09
Dcdc2b	1255	0	13.5	0.001	0.3595	8.489846	Up	0.000122	0.000224
Tspan12	2563	0	27	0.001	0.352768	8.462575	Up	7.45E-09	1.74E-08
Fam20a	1626	0	17	0.001	0.349616	8.449628	Up	7.63E-06	1.54E-05
Rnase9	1279	0	13	0.001	0.340054	8.409618	Up	0.000122	0.000224
LOC679368	2001	0	20	0.001	0.3355	8.390171	Up	9.54E-07	2.03E-06

Nkg7	813	0	8	0.001	0.335172	8.388757	Up	0.003906	0.006334
Slc24a4	2295	0	23	0.001	0.334988	8.387964	Up	1.19E-07	2.65E-07
LOC100912239	1008	0	10	0.001	0.333004	8.379396	Up	0.000977	0.00166
LOC309220	939	0	9.5	0.001	0.328673	8.360511	Up	0.001953	0.003246
LOC685680	1008	0	10	0.001	0.327647	8.355998	Up	0.000977	0.001657
Nlrp6	2906	1	341.5	0.012116	3.918047	8.337049	Up	0.000575	0.001004
Snap91	3419	0	33	0.001	0.321458	8.328486	Up	1.16E-10	2.89E-10
Ereg	834	0	8	0.001	0.320257	8.323087	Up	0.003906	0.006335
RGD1311676	861	0	8	0.001	0.314396	8.296437	Up	0.003906	0.006334
Sox30	2013	0	19	0.001	0.312444	8.287453	Up	1.91E-06	3.99E-06
LOC100910040	1956	1	328	0.018001	5.601827	8.281683	Up	0.000575	0.001006
LOC100360712	921	0	8.5	0.001	0.309596	8.274243	Up	0.003906	0.006347
Stmn4	1333	2	661	0.053688	16.54012	8.267147	Up	0.000115	0.000214
Fam50b	1316	0	12	0.001	0.305807	8.256477	Up	0.000244	0.00044
LOC680549	3228	0	30	0.001	0.305268	8.253931	Up	9.31E-10	2.24E-09
Arrdc4	949	5	1621.5	0.189136	57.10344	8.238014	Up	2.76E-05	5.41E-05
Actl6b	1562	0	14	0.001	0.299818	8.227942	Up	6.10E-05	0.000116
LOC100911650	1234	0	11	0.001	0.299801	8.227864	Up	0.000488	0.000865
RGD1309410	901	0	8	0.001	0.296442	8.211607	Up	0.003906	0.00634
LOC100912860	1224	0	11	0.001	0.296368	8.211247	Up	0.000488	0.000865
Rbm11	2501	0	22	0.001	0.295846	8.208701	Up	2.38E-07	5.23E-07
P2ry12	1254	0	11	0.001	0.293585	8.197632	Up	0.000488	0.000865
LOC690984	924	0	8	0.001	0.289063	8.175242	Up	0.003906	0.006331
Hs6st2	2394	0	21	0.001	0.288731	8.173584	Up	4.77E-07	1.03E-06
Gprin2	1601	0	14	0.001	0.288579	8.172823	Up	6.10E-05	0.000116

LOC690309	2232	0	19	0.001	0.286223	8.160995	Up		1.91E-06	3.99E-06
Plek	1671	0	14	0.001	0.281876	8.138919	Up		6.10E-05	0.000116
Cx3cl1	3044	1	297	0.011567	3.257222	8.13749	Up		0.000575	0.00101
RGD1305725	1370	0	11.5	0.001	0.28124	8.135656	Up		0.000488	0.000864
Naip2	5990	0	50	0.001	0.277937	8.118612	Up		8.88E-16	3.77E-15
LOC100360779	3994	0	33	0.001	0.276531	8.111298	Up		1.16E-10	2.89E-10
LOC100362038	1104	0	9	0.001	0.274621	8.101297	Up		0.001953	0.003247
RGD1565143	1093	0	9	0.001	0.272444	8.089815	Up		0.001953	0.003236
LOC100910397	969	0	8	0.001	0.270066	8.07717	Up		0.003906	0.006327
RGD1562655	3977	0	32	0.001	0.269544	8.074379	Up		2.33E-10	5.71E-10
Echdc2	1390	0	11	0.001	0.266155	8.056121	Up		0.000488	0.000865
Ust5r	2079	0	16.5	0.001	0.265624	8.053242	Up		1.53E-05	3.03E-05
Fam124b	1371	0	11	0.001	0.263278	8.040445	Up		0.000488	0.000864
Avpr1b	1516	0	12	0.001	0.263088	8.039403	Up		0.000244	0.00044
Capza3	1222	0	9.5	0.001	0.262868	8.038195	Up		0.001953	0.003239
Gabrg2	1401	0	11	0.001	0.261495	8.030641	Up		0.000488	0.000865
Cd96	2079	0	16	0.001	0.257811	8.01017	Up		1.53E-05	3.03E-05
Lbx1	1164	0	9	0.001	0.257372	8.007713	Up		0.001953	0.003242
Etv2	1040	0	8	0.001	0.256822	8.004623	Up		0.003906	0.006346
Trim63	1861	1	271	0.01892	4.857456	8.004158	Up		0.000575	0.001004
RGD1562063	1443	0	11	0.001	0.252637	7.980921	Up		0.000488	0.000865
LOC100911572	1216	0	9	0.001	0.250807	7.970435	Up		0.001953	0.003236
12-Sep	1320	0	10	0.001	0.250203	7.966955	Up		0.000977	0.001661
C5ar1	1772	0	13	0.001	0.249508	7.962944	Up		0.000122	0.000224
LOC689946	1362	0	10	0.001	0.247774	7.952881	Up		0.000977	0.001657

RGD1564409	1482	0	11	0.001	0.244774	7.935305	Up	0.000488	0.000865
RGD1559459	2704	0	20	0.001	0.244281	7.932398	Up	9.54E-07	2.03E-06
LOC100909648	1266	0	9	0.001	0.240902	7.9123	Up	0.001953	0.003244
Nfe2	1684	0	12	0.001	0.240049	7.907183	Up	0.000244	0.00044
Ankk1	2412	0	17	0.001	0.237925	7.894365	Up	7.63E-06	1.55E-05
Apoa4	1423	0	10	0.001	0.235888	7.881956	Up	0.000977	0.001658
MGC116197	1431	0	10	0.001	0.232053	7.858311	Up	0.000977	0.001661
Aoah	1747	0	12	0.001	0.231392	7.854195	Up	0.000244	0.00044
Matn3	2003	1	252	0.018151	4.196394	7.852949	Up	0.000575	0.001008
Trpv3	3028	0	21	0.001	0.230655	7.849592	Up	4.77E-07	1.03E-06
LOC100912561	1158	0	8	0.001	0.229097	7.839815	Up	0.003906	0.006342
Mboat4	1308	0	9	0.001	0.229038	7.839441	Up	0.001953	0.003238
LOC686581	1167	0	8	0.001	0.228873	7.838402	Up	0.003906	0.006344
Trim50	1452	0	10	0.001	0.228077	7.833378	Up	0.000977	0.001661
RGD1560110	1299	0	9	0.001	0.227853	7.83196	Up	0.001953	0.003245
Fam163b	1313	0	9	0.001	0.226795	7.825242	Up	0.001953	0.003247
Lipk	2070	0	14	0.001	0.226674	7.824476	Up	6.10E-05	0.000116
Kiss1r	1188	0	8	0.001	0.226342	7.822362	Up	0.003906	0.006331
Rac2	1325	0	9	0.001	0.226099	7.820811	Up	0.001953	0.00324
Ank1	5124	0	34.5	0.001	0.225936	7.819769	Up	5.82E-11	1.46E-10
Mmp20	3284	0	22	0.001	0.224759	7.812238	Up	2.38E-07	5.23E-07
St8sia6	1197	0	8	0.001	0.223137	7.801783	Up	0.003906	0.006345
Arg1	1507	0	10	0.001	0.222739	7.799213	Up	0.000977	0.001659
RGD1561442	4371	0	29	0.001	0.220892	7.787195	Up	1.86E-09	4.43E-09
Slc17a1	1848	0	12	0.001	0.220694	7.785902	Up	0.000244	0.00044

Ces2c	1902	2	476	0.03823	8.375827	7.775385	Up	0.000115	0.000214
LOC367746	1385	0	9	0.001	0.218904	7.774152	Up	0.001953	0.003235
LOC687071	1518	0	10	0.001	0.217568	7.765322	Up	0.000977	0.001657
Hoxb2	1657	0	11	0.001	0.215664	7.752639	Up	0.000488	0.000864
Btn3a2	1770	0	11.5	0.001	0.212598	7.731983	Up	0.000488	0.000865
Prps1l1	1691	0	10.5	0.001	0.207576	7.697498	Up	0.000977	0.001658
Ccdc154	2391	0	15	0.001	0.207571	7.69746	Up	3.05E-05	5.93E-05
Lca5l	2086	0	13	0.001	0.20591	7.68587	Up	0.000122	0.000225
Asic3	1602	0	10	0.001	0.205036	7.679734	Up	0.000977	0.001662
Esr1	2090	0	13	0.001	0.203793	7.670964	Up	0.000122	0.000224
Kirrel2	3364	0	20.5	0.001	0.203591	7.669529	Up	9.54E-07	2.03E-06
LOC500827	1981	0	12	0.001	0.202242	7.65994	Up	0.000244	0.00044
Klhdc7a	2322	0	14	0.001	0.202074	7.658739	Up	6.10E-05	0.000116
LOC683506	2338	0	14	0.001	0.199921	7.643286	Up	6.10E-05	0.000116
Btnl8	1933	0	11.5	0.001	0.199792	7.642357	Up	0.000488	0.000865
LOC100911250	2694	0	16	0.001	0.19762	7.626587	Up	1.53E-05	3.03E-05
Gys2	2386	0	14	0.001	0.197408	7.625037	Up	6.10E-05	0.000116
Col8a2	4063	0	24	0.001	0.196329	7.617127	Up	5.96E-08	1.34E-07
Hck	2081	0	12	0.001	0.195119	7.608208	Up	0.000244	0.000441
Hpca	1561	0	9	0.001	0.193069	7.592976	Up	0.001953	0.003237
Stac3	1546	0	9	0.001	0.192614	7.589569	Up	0.001953	0.003237
Ankrd35	3207	0	18	0.001	0.189075	7.562813	Up	3.81E-06	7.85E-06
Lep	3290	1	197	0.010702	1.996687	7.543575	Up	0.000575	0.001004
Bcl2a1	699	0.5	99	0.025186	4.696414	7.542803	Up	0	0
Asb2	2512	0	14	0.001	0.186073	7.539725	Up	6.10E-05	0.000116

Gstt3	1785	0	10	0.001	0.186033	7.539411	Up	0.000977	0.00166
Fam71a	2181	0	12	0.001	0.183696	7.521179	Up	0.000244	0.00044
Slfn14	2718	0	15	0.001	0.183261	7.517752	Up	3.05E-05	5.93E-05
Gpr39	1575	0.5	96.5	0.011178	2.04589	7.51596	Up	0	0
LOC100911827	1452	0	8	0.001	0.18271	7.513409	Up	0.003906	0.006338
Ly6b	905	1	192	0.038906	7.087147	7.509074	Up	0.000575	0.001005
Bend5	1834	0	10	0.001	0.180081	7.4925	Up	0.000977	0.001659
Il1rapl1	2165	0	12	0.001	0.180065	7.492376	Up	0.000244	0.00044
Gata1	1863	0	10	0.001	0.178244	7.477707	Up	0.000977	0.001662
B3gat1	2090	0	11	0.001	0.175289	7.453595	Up	0.000488	0.000863
Htr7	1493	0	8	0.001	0.174075	7.443567	Up	0.003906	0.006343
Pabpc3	3150	0	16.5	0.001	0.173026	7.434846	Up	1.53E-05	3.03E-05
Slc9b2	2011	0.5	91	0.008754	1.50363	7.424242	Up	0	0
Efcab6	4724	0	24	0.001	0.168477	7.396404	Up	5.96E-08	1.34E-07
Kif4b	2982	0	15	0.001	0.16764	7.389222	Up	3.05E-05	5.93E-05
Il10ra	1814	0	9	0.001	0.16515	7.367629	Up	0.001953	0.003242
Chrnb3	2644	0	13	0.001	0.164496	7.361913	Up	0.000122	0.000225
Kremen2	1936	0	9.5	0.001	0.162668	7.345784	Up	0.001953	0.00324
Fbll1	1644	0	8	0.001	0.162466	7.343996	Up	0.003906	0.006345
Mmp21	1854	0	9	0.001	0.161586	7.336162	Up	0.001953	0.003235
Tekt3	1761	0	8.5	0.001	0.161407	7.33456	Up	0.003906	0.006326
LOC689458	3573	0	17	0.001	0.158599	7.309244	Up	7.63E-06	1.54E-05
Lrrc6	1698	0	8	0.001	0.15836	7.30706	Up	0.003906	0.006342
Vom2r66	1671	0	8	0.001	0.157687	7.300917	Up	0.003906	0.006328
Fli1	1871	0	9	0.001	0.157232	7.296751	Up	0.001953	0.003239

LOC689599	1722	0	8	0.001	0.157198	7.296437	Up	0.003906	0.006329
RGD1564259	5736	0	27	0.001	0.156999	7.294608	Up	7.45E-09	1.74E-08
Cytip	1694	0	8	0.001	0.155546	7.281194	Up	0.003906	0.006333
LOC685081	1736	0	8	0.001	0.154893	7.27513	Up	0.003906	0.006341
LOC317471	2158	0	10	0.001	0.153878	7.26564	Up	0.000977	0.001662
LOC499971	2583	0	12	0.001	0.153713	7.264099	Up	0.000244	0.00044
Tmie	2599	0	12	0.001	0.15346	7.261716	Up	0.000244	0.00044
Rhbdl3	3750	0	17	0.001	0.152074	7.248626	Up	7.63E-06	1.54E-05
LOC294165	2559	0	11.5	0.001	0.149863	7.227496	Up	0.000488	0.000864
Zfp286a	2961	0	13	0.001	0.147494	7.204509	Up	0.000122	0.000224
Ttc39d	2309	0	10	0.001	0.144594	7.175866	Up	0.000977	0.001657
RGD1559958	1939	0	8.5	0.001	0.144269	7.172618	Up	0.003906	0.006345
LOC100359988	2121	0	9	0.001	0.143791	7.167833	Up	0.001953	0.00324
Cldn2	3052	0	13	0.001	0.141326	7.142888	Up	0.000122	0.000224
Ankar	4398	0	18.5	0.001	0.141156	7.141151	Up	3.81E-06	7.86E-06
Havcr1	2538	0	10.5	0.001	0.138302	7.111683	Up	0.000977	0.001659
Neil3	1975	0	8	0.001	0.137972	7.108232	Up	0.003906	0.006328
Aldh8a1	1499	1.5	220	0.035616	4.898198	7.103589	Up	0.000575	0.00101
Prnd	537	0.5	73	0.032784	4.501744	7.101357	Up	0	0
Abcg5	2470	0	10	0.001	0.135898	7.086381	Up	0.000977	0.001657
RGD1564285	1980	0	8	0.001	0.135805	7.085396	Up	0.003906	0.00633
LOC100910423	2219	0	9	0.001	0.135007	7.076894	Up	0.001953	0.003243
Best2	2006	0.5	70.5	0.008776	1.178525	7.069183	Up	0	0
Ptchd1	2676	0	11	0.001	0.134213	7.068383	Up	0.000488	0.000864
Paqr9	2297	0	9	0.001	0.133557	7.061317	Up	0.001953	0.003246

LOC680874	1253	1	140	0.0281	3.728933	7.052029	Up	0.000575	0.001004
Sypl2	3403	0	13.5	0.001	0.132052	7.044959	Up	0.000122	0.000224
Alpk2	6534	0	26	0.001	0.132026	7.044677	Up	1.49E-08	3.43E-08
Vom2r45	3001	0	12	0.001	0.131703	7.041147	Up	0.000244	0.00044
Trpc7	3055	0	12	0.001	0.131143	7.034997	Up	0.000244	0.00044
Tnfrsf11a	2718	0	10.5	0.001	0.13113	7.034856	Up	0.000977	0.001658
Phlda2	642	2	284.5	0.113261	14.77772	7.027633	Up	0.000115	0.000214
Nodal	2034	0	8	0.001	0.129545	7.017309	Up	0.003906	0.006341
Cd83	2119	4	556.5	0.067547	8.746724	7.016699	Up	4.59E-05	8.81E-05
Vom2r58	2574	0	10	0.001	0.129358	7.015228	Up	0.000977	0.001659
Ly75	5974	0	23	0.001	0.128992	7.011135	Up	1.19E-07	2.65E-07
RGD1565356	2107	0	8	0.001	0.128474	7.005332	Up	0.003906	0.006339
Fap	2293	0	9	0.001	0.128295	7.003324	Up	0.001953	0.003246
Pitx1	2287	0	9	0.001	0.127845	6.998249	Up	0.001953	0.003243
Kcns3	2370	0	9	0.001	0.127165	6.990559	Up	0.001953	0.003244
Vipr1	4945	0	19	0.001	0.126643	6.984622	Up	1.91E-06	3.99E-06
Trim72	2363	1.5	200.5	0.022593	2.833054	6.970315	Up	0.000575	0.001009
Itih3	2783	0	10.5	0.001	0.125157	6.967593	Up	0.000977	0.001662
Fam163a	3908	0	15	0.001	0.125154	6.96756	Up	3.05E-05	5.93E-05
Hsh2d	2176	2	266.5	0.032889	4.089171	6.958059	Up	0.000115	0.000214
Amn	1759	0.5	65.5	0.010008	1.239901	6.95286	Up	0	0
Gria1	2992	0	11	0.001	0.123648	6.950096	Up	0.000488	0.000864
Myom1	5551	10	1312.5	0.063843	7.883163	6.948106	Up	1.61E-06	3.40E-06
Zfp458	3170	26.5	3451	0.297235	36.32618	6.933262	Up	6.87E-11	1.72E-10
Iqub	3058	11.5	1501.5	0.135036	16.37654	6.92214	Up	8.84E-07	1.89E-06

Scn5a	8468	3	386	0.012609	1.522627	6.915921	Up	7.36E-05	0.000139
Fstl5	1160	3	383	0.092049	11.02418	6.904061	Up	7.36E-05	0.000139
Kprp	2815	0	10	0.001	0.119243	6.897757	Up	0.000977	0.001657
Dscaml1	6929	0	25	0.001	0.119032	6.895201	Up	2.98E-08	6.78E-08
Apba1	3681	0	13	0.001	0.118644	6.890496	Up	0.000122	0.000224
Bnc1	4454	0	16	0.001	0.118318	6.886527	Up	1.53E-05	3.03E-05
Kcnj11	3051	0	11	0.001	0.117127	6.87193	Up	0.000488	0.000863
Ace3	2583	0	9	0.001	0.116679	6.866399	Up	0.001953	0.003237
LOC500797	2571	0	9	0.001	0.116523	6.864474	Up	0.001953	0.003238
Cers3	2825	0	10	0.001	0.116272	6.861358	Up	0.000977	0.001658
Gpr61	3368	0	12	0.001	0.115749	6.854851	Up	0.000244	0.00044
LOC302999	2126	0.5	63	0.00855	0.982158	6.843805	Up	0	0
Pmfbp1	3312	0	11	0.001	0.112245	6.810506	Up	0.000488	0.000864
Ccdc125	2438	1	117.5	0.014442	1.610747	6.801307	Up	0.000575	0.00101
RGD1624205	8935	0	30	0.001	0.11089	6.792988	Up	9.31E-10	2.24E-09
Ampd1	2275	2	233.5	0.030954	3.42258	6.78883	Up	0.000115	0.000214
Csf2	441	116.5	13800	9.457491	1044.9	6.787692	Up	9.68E-14	3.75E-13
LOC100912082	2718	0	9	0.001	0.110221	6.784258	Up	0.001953	0.003244
Vom2r47	2475	0	8	0.001	0.107917	6.753778	Up	0.003906	0.00634
Trim43a	1356	1	113.5	0.025966	2.799523	6.752418	Up	0.000575	0.001003
Ccdc135	2813	0	9	0.001	0.107139	6.743337	Up	0.001953	0.003243
Slc5a1	2522	0	8	0.001	0.10662	6.736328	Up	0.003906	0.006343
Vom2r9	2601	0	8	0.001	0.104765	6.711018	Up	0.003906	0.006335
Cdh8	3123	0	10	0.001	0.104024	6.700776	Up	0.000977	0.00166
Colq	2645	6.5	717	0.088261	9.059941	6.681577	Up	1.62E-05	3.20E-05

Aoc3	3961	4	436	0.036136	3.673403	6.667554	Up	4.59E-05	8.83E-05
Trpc5	2925	0	9	0.001	0.100575	6.652124	Up	0.001953	0.003243
Lax1	2241	2	214	0.031935	3.1858	6.640371	Up	0.000115	0.000214
LOC100360041	2683	0	8	0.001	0.099551	6.637359	Up	0.003906	0.006325
Slc18a1	2357	1	104	0.014938	1.469337	6.619997	Up	0.000575	0.00101
Trpc3	3420	0	10	0.001	0.098149	6.616896	Up	0.000977	0.001657
Zscan10	2748	0	8	0.001	0.097851	6.612515	Up	0.003906	0.006327
LOC689074	4141	0	12	0.001	0.09675	6.59619	Up	0.000244	0.00044
Pram1	2279	1	101.5	0.01545	1.491493	6.593037	Up	0.000575	0.001006
Adra2b	4047	5	510.5	0.043643	4.213175	6.59302	Up	2.76E-05	5.40E-05
Grm3	3384	0	10	0.001	0.096533	6.592951	Up	0.000977	0.001661
Serpina9	1680	1	104.5	0.021641	2.084507	6.589804	Up	0.000575	0.001009
Ccdc153	1392	0.5	53	0.013059	1.256969	6.588744	Up	0	0
Mttp	3997	0	11.5	0.001	0.095721	6.580769	Up	0.000488	0.000865
LOC690507	1396	1	103.5	0.026043	2.4737	6.569606	Up	0.000575	0.001005
Wdr93	2082	1	100.5	0.016912	1.60102	6.564841	Up	0.000575	0.001006
Gpr179	6930	0	19.5	0.001	0.0944	6.560717	Up	1.91E-06	3.99E-06
Aldh1l2	3502	0	10	0.001	0.094308	6.559314	Up	0.000977	0.00166
Il6	1046	23	2294	0.78408	73.2401	6.54549	Up	4.68E-10	1.13E-09
RGD1566124	2905	3	295.5	0.036756	3.399709	6.531286	Up	7.36E-05	0.000139
Tdh	1546	0.5	49	0.011387	1.052945	6.53085	Up	1.78E-15	7.51E-15
Lix1	1522	1.5	145	0.035454	3.177193	6.485645	Up	0.000575	0.001011
Amhr2	1964	2	192	0.036439	3.248761	6.478257	Up	0.000115	0.000214
Itgam	3456	0	9	0.001	0.087205	6.446343	Up	0.001953	0.003245
LOC100912250	976	1	91.5	0.036076	3.13693	6.442186	Up	0.000575	0.001004

Tmem30b	3076	0	8	0.001	0.086247	6.430395	Up	0.003906	0.00633
Kif27	4468	1.5	136.5	0.011821	1.01908	6.429813	Up	0.000575	0.001003
Cntnap5c	3918	0	10	0.001	0.086133	6.428491	Up	0.000977	0.001661
Txnip	2771	477.5	43899	6.15594	528.9393	6.424979	Up	0	0
Muc19	12067	0	31	0.001	0.085547	6.418639	Up	4.66E-10	1.13E-09
Lhfpl3	3106	0	8	0.001	0.085414	6.416393	Up	0.003906	0.006324
Mpp4	2201	1	89.5	0.015997	1.35537	6.404725	Up	0.000575	0.001005
Mapk4	3596	0	9	0.001	0.08381	6.389053	Up	0.001953	0.003245
Kcnc1	3977	0	10	0.001	0.083271	6.379739	Up	0.000977	0.00166
Col6a6	7165	0	17.5	0.001	0.081482	6.348411	Up	7.63E-06	1.54E-05
B3gnt6	1594	1	85.5	0.022089	1.787718	6.33865	Up	0.000575	0.001008
Ankrd34b	3292	0	8	0.001	0.080588	6.332486	Up	0.003906	0.006344
Cldn6	1544	1	87.5	0.023547	1.883028	6.321364	Up	0.000575	0.001007
Efnb3	3143	1	86	0.011568	0.911825	6.300607	Up	0.000575	0.001006
Dll4	3409	0	8	0.001	0.07835	6.291858	Up	0.003906	0.006329
Snai3	1594	1	81.5	0.022089	1.710148	6.274652	Up	0.000575	0.00101
Cdcp1	5232	0	12	0.001	0.076231	6.25231	Up	0.000244	0.00044
RGD1565058	566	0.5	40	0.031104	2.364263	6.248143	Up	9.09E-13	2.89E-12
LOC367140	1059	3	243.5	0.100827	7.659337	6.247259	Up	7.36E-05	0.000139
Cacna1f	5975	0.5	40	0.002946	0.222606	6.239384	Up	9.09E-13	2.89E-12
Tsks	1820	2	164.5	0.039952	3.014934	6.237701	Up	0.000115	0.000214
LOC294887	3924	0	9	0.001	0.075428	6.237036	Up	0.001953	0.003241
Gpr97	1962	2	157.5	0.035892	2.686327	6.225839	Up	0.000115	0.000214
LOC100910903	797	4	316.5	0.178151	13.27588	6.219566	Up	4.59E-05	8.83E-05
Col4a5	6615	0	15	0.001	0.073666	6.20293	Up	3.05E-05	5.93E-05

Tp73	4749	5	398	0.038037	2.789334	6.196384	Up		2.76E-05	5.40E-05
LOC100362022	3479	0.5	38.5	0.00506	0.370119	6.192611	Up		3.64E-12	1.03E-11
LOC100360457	4605	0	10	0.001	0.072501	6.179933	Up		0.000977	0.00166
Actbl2	2748	0.5	38	0.006406	0.462663	6.174296	Up		3.64E-12	1.03E-11
LOC100910799	633	1	76	0.055624	4.005683	6.170205	Up		0.000575	0.001009
Iqsec3	7410	0	16	0.001	0.071847	6.166863	Up		1.53E-05	3.03E-05
Impg2	4370	0.5	38	0.00416	0.290732	6.127031	Up		3.64E-12	1.03E-11
Ms4a14	3801	0	8	0.001	0.069796	6.125072	Up		0.003906	0.006337
Gprc5d	1402	4	299.5	0.102092	7.113266	6.122571	Up		4.59E-05	8.82E-05
Cgnl1	6480	1	74.5	0.005611	0.38322	6.093876	Up		0.000575	0.001004
Lrrc37a	9414	6	440	0.022928	1.561993	6.090119	Up		1.62E-05	3.21E-05
RGD1564308	1216	2	148	0.059797	4.056125	6.083881	Up		0.000115	0.000214
LOC687465	5496	0	11	0.001	0.067314	6.072824	Up		0.000488	0.000865
Il12a	793	1	73	0.045847	3.056415	6.058869	Up		0.000575	0.001007
LOC100361323	4073	0	8	0.001	0.065577	6.035115	Up		0.003906	0.006325
Cd69	1630	11	777	0.243241	15.89408	6.02996	Up		8.84E-07	1.88E-06
Bai1	5543	0	11	0.001	0.065119	6.025007	Up		0.000488	0.000864
Mybpc3	4165	0	8	0.001	0.064344	6.007743	Up		0.003906	0.006346
Tchh	4722	0	9	0.001	0.063444	5.987406	Up		0.001953	0.003242
Baat	1734	2	133	0.040611	2.56133	5.978876	Up		0.000115	0.000214
Nell1	2915	1	69	0.012472	0.786274	5.978237	Up		0.000575	0.00101
Agbl3	3123	18	1210	0.205876	12.93075	5.972886	Up		1.14E-08	2.63E-08
Scn11a	5905	0	11	0.001	0.062651	5.969269	Up		0.000488	0.000863
Nhlh1	2452	1	68.5	0.014827	0.92665	5.965692	Up		0.000575	0.001005
Anxa3	3392	2	133	0.021268	1.308032	5.942593	Up		0.000115	0.000214

Il17f	1308	1	67	0.027796	1.707428	5.940824	Up	0.000575	0.001008
Il25	510	2	134	0.142575	8.740455	5.937914	Up	0.000115	0.000214
LOC100912110	1372	9	590.5	0.235984	14.37964	5.929197	Up	2.92E-06	6.06E-06
RGD1563398	5019	0	9	0.001	0.060407	5.916638	Up	0.001953	0.00324
RGD1563124	372	2	129.5	0.1893	11.31166	5.900993	Up	0.000115	0.000214
Trhde	6678	0	12	0.001	0.059725	5.900256	Up	0.000244	0.00044
Gpr81	1056	1	63	0.033343	1.985861	5.896254	Up	0.000575	0.001006
Abcc12	4541	0	8	0.001	0.059215	5.887887	Up	0.003906	0.006333
RGD1564171	636	15	944.5	0.83583	49.38835	5.884818	Up	7.51E-08	1.68E-07
Fsd2	2927	2	125	0.024059	1.420892	5.884099	Up	0.000115	0.000213
Tnfsf4	1804	1	61.5	0.019518	1.137438	5.864867	Up	0.000575	0.001005
Fam71e2	3063	1	63.5	0.01187	0.690242	5.861756	Up	0.000575	0.001011
Moxd1	2990	0.5	30.5	0.005888	0.342223	5.861035	Up	9.31E-10	2.24E-09
Plcz1	1938	1	63	0.01876	1.085794	5.854955	Up	0.000575	0.001003
LOC100912131	743	15	919.5	0.713917	41.2785	5.853489	Up	7.51E-08	1.68E-07
Pi16	1900	2	123.5	0.037667	2.175117	5.851665	Up	0.000115	0.000214
Slc6a20	1950	1	62.5	0.01835	1.05832	5.849823	Up	0.000575	0.001008
Tmem132b	4605	1	62	0.007895	0.449508	5.831255	Up	0.000575	0.001004
Gsdmc	1529	1	60	0.023028	1.308966	5.828895	Up	0.000575	0.001008
LOC299567	1615	1	60	0.021802	1.232575	5.821089	Up	0.000575	0.001009
LOC499240	1255	4	238.5	0.113136	6.353072	5.81132	Up	4.59E-05	8.81E-05
Foxd4	1451	0.5	30.5	0.012528	0.70148	5.807158	Up	9.31E-10	2.24E-09
Jakmip3	5964	0	10	0.001	0.055377	5.791213	Up	0.000977	0.001658
Cidec	1654	1	60	0.021981	1.21113	5.783949	Up	0.000575	0.00101
Muc2	6134	0	10	0.001	0.054136	5.758507	Up	0.000977	0.001658

Mcf2	3204	1	57	0.010989	0.590308	5.747294	Up	0.000575	0.001007
LOC100912291	709	1	58.5	0.051279	2.736358	5.737751	Up	0.000575	0.001008
Rasal3	3966	2	114	0.018045	0.961043	5.73493	Up	0.000115	0.000214
Lypd1	2038	9	512.5	0.158022	8.404461	5.732957	Up	2.92E-06	6.07E-06
LOC684158	2151	1.5	86	0.025087	1.333178	5.731801	Up	0.000575	0.001007
Dpf3	1240	1	58	0.02932	1.555835	5.729665	Up	0.000575	0.001005
Fgg	1554	3.5	199	0.081146	4.278878	5.720562	Up	7.36E-05	0.000139
Mab21l3	3926	7	393	0.063655	3.337727	5.71245	Up	9.28E-06	1.87E-05
Cux2	5092	1	54.5	0.006915	0.357076	5.690415	Up	0.000575	0.001005
Tfr2	2964	3	165	0.036024	1.851594	5.68365	Up	7.36E-05	0.000139
Ltk	2252	1	54	0.015635	0.803369	5.683222	Up	0.000575	0.001009
RGD1565989	1061	1	54	0.033185	1.699235	5.678189	Up	0.000575	0.001009
LOC100125386	1326	1	55	0.027418	1.382786	5.656295	Up	0.000575	0.001007
Ceacam19	2024	6	325.5	0.106643	5.351707	5.649132	Up	1.62E-05	3.21E-05
LOC498470	1405	1	53.5	0.025877	1.278039	5.626138	Up	0.000575	0.001007
LOC100360380	2193	50	2627	0.81219	39.96377	5.620731	Up	3.62E-14	1.46E-13
LOC100363061	1137	1	53.5	0.031976	1.57295	5.620342	Up	0.000575	0.00101
Gpr83	3673	1	52	0.009586	0.471199	5.619247	Up	0.000575	0.001005
C9	2083	1	51.5	0.016903	0.823078	5.605642	Up	0.000575	0.001008
LOC288019	1536	7.5	394	0.172296	8.336713	5.596516	Up	9.28E-06	1.87E-05
LOC688591	996	1	52.5	0.036503	1.759395	5.590933	Up	0.000575	0.001011
Ly6g6c	846	3	155	0.127569	6.127608	5.585977	Up	7.36E-05	0.000139
Itgb3	2378	2	101	0.029613	1.414621	5.578043	Up	0.000115	0.000214
Lppr4	5554	0	8	0.001	0.047766	5.577923	Up	0.003906	0.006338
LOC688839	2076	2.5	127	0.042953	2.045484	5.573525	Up	0.000115	0.000214

Gpr111	1953	1	50	0.018029	0.858444	5.573367	Up		0.000575	0.001011
Spdya	1896	6.5	329	0.122523	5.781044	5.560201	Up		1.62E-05	3.21E-05
LOC100912633	1111	1	49.5	0.031692	1.489556	5.554621	Up		0.000575	0.001011
Acsbg2	2644	0.5	24.5	0.006658	0.311924	5.549868	Up		5.96E-08	1.34E-07
Grin2b	5259	2	99	0.01339	0.623882	5.542012	Up		0.000115	0.000214
Atp2b2	7019	0	10	0.001	0.046541	5.540415	Up		0.000977	0.001659
Zc2hc1c	2004	1	49	0.01757	0.815895	5.537217	Up		0.000575	0.001007
Otogl-ps1	6798	1	49	0.005179	0.239461	5.53085	Up		0.000575	0.001007
RGD1560099	588	1	49	0.059881	2.766928	5.530052	Up		0.000575	0.001006
Plg	2752	3	148.5	0.039216	1.80403	5.523628	Up		7.36E-05	0.000139
Lman1l	1588	0.5	24.5	0.011086	0.509714	5.522851	Up		5.96E-08	1.34E-07
LOC689991	11066	1	49	0.003182	0.146128	5.521246	Up		0.000575	0.001006
Olr809	936	2	100	0.077685	3.559274	5.5178	Up		0.000115	0.000214
LOC100912068	2148	1	48	0.016392	0.749426	5.514731	Up		0.000575	0.001006
Neurod4	3314	2	98	0.021595	0.986755	5.513911	Up		0.000115	0.000214
Phgr1	443	2	98.5	0.164138	7.434653	5.50128	Up		0.000115	0.000214
Abca13	15126	0.5	24	0.001164	0.052498	5.495241	Up		5.96E-08	1.34E-07
Timp1	740	6	290	0.290134	13.05605	5.491853	Up		1.62E-05	3.21E-05
Gzmk	884	2	96	0.080958	3.635899	5.489003	Up		0.000115	0.000214
Slc23a3	2052	9	429.5	0.156665	7.006768	5.482998	Up		2.92E-06	6.06E-06
Bmp6	1521	1	48	0.023903	1.052444	5.460399	Up		0.000575	0.001005
Xlr4a	648	1	48	0.056106	2.461986	5.455524	Up		0.000575	0.001004
Pdlim3	1568	1	48	0.023187	1.016305	5.453895	Up		0.000575	0.001005
Cel	2062	1	46	0.017076	0.747427	5.451927	Up		0.000575	0.001008
Btn1a1	1575	1	46	0.022355	0.977393	5.450241	Up		0.000575	0.001009

LOC100365119	1805	1	47.5	0.020142	0.879348	5.448142	Up		0.000575	0.001008
Iqch	3618	4.5	209	0.044269	1.928398	5.44497	Up		4.59E-05	8.84E-05
Vom2r44	2670	7	324.5	0.093599	4.052798	5.436282	Up		9.28E-06	1.86E-05
RGD1560527	2331	1	47	0.015597	0.674337	5.434127	Up		0.000575	0.001009
Col20a1	4188	14	647.5	0.119345	5.152963	5.432188	Up		1.40E-07	3.11E-07
LOC687758	984	10	458.5	0.360154	15.53161	5.430449	Up		1.61E-06	3.40E-06
LOC100912842	614	0.5	23.5	0.029606	1.275635	5.429163	Up		1.19E-07	2.65E-07
Cxcl11	1054	6	272.5	0.202067	8.623677	5.415394	Up		1.62E-05	3.20E-05
LOC100911495	1243	3	138	0.086825	3.705213	5.415307	Up		7.36E-05	0.000139
LOC100365779	2316	2	91	0.030901	1.312997	5.409071	Up		0.000115	0.000213
LOC498465	1403	2	89.5	0.050192	2.125638	5.40429	Up		0.000115	0.000214
Hmgb1-ps2	1904	0.5	22.5	0.009246	0.389103	5.395138	Up		2.38E-07	5.24E-07
Tmco5b	1235	3	132	0.08553	3.558266	5.378602	Up		7.36E-05	0.000139
LOC300308	3991	3	135	0.027042	1.121453	5.374043	Up		7.36E-05	0.000139
LOC679119	1929	1	45.5	0.018847	0.780741	5.372405	Up		0.000575	0.001006
Klhl14	4068	3	132.5	0.026248	1.086456	5.371286	Up		7.36E-05	0.000139
St6galnac3	2956	1.5	67	0.018255	0.754911	5.369948	Up		0.000575	0.001003
RGD1565804	8082	0	10	0.001	0.04131	5.368422	Up		0.000977	0.001661
Cmya5	11694	1	43	0.003011	0.122844	5.35047	Up		0.000575	0.001008
Rab44	2169	4	173	0.065462	2.665015	5.347353	Up		4.59E-05	8.82E-05
Nfe2l3	1944	3	130	0.054926	2.233583	5.345725	Up		7.36E-05	0.000139
Tspan1	1256	1	44	0.028946	1.172468	5.340021	Up		0.000575	0.001005
P2ry4	1177	4	170	0.119659	4.805401	5.32765	Up		4.59E-05	8.81E-05
4-Mar	1230	1	44	0.029558	1.183349	5.32317	Up		0.000575	0.001011
Tspy1	1245	2	83.5	0.056562	2.244618	5.310495	Up		0.000115	0.000213

LOC100913005	558	15.5	657.5	0.99141	39.27487	5.307982	Up	7.51E-08	1.68E-07
Fam71f1	1600	45.5	1865.5	1.012031	38.94353	5.266059	Up	2.49E-14	1.02E-13
LOC690486	966	3	123	0.110534	4.249252	5.264641	Up	7.36E-05	0.000139
Bcmo1	1932	2	81	0.037043	1.401621	5.241764	Up	0.000115	0.000214
Dnah12	11853	1	41	0.003067	0.116018	5.241235	Up	0.000575	0.00101
Slco5a1	3246	2.5	102	0.027824	1.050787	5.238972	Up	0.000115	0.000214
RGD1563285	931	1	41	0.039051	1.471277	5.235559	Up	0.000575	0.001006
LOC100911553	695	170	6834	8.711479	328.0185	5.234716	Up	3.78E-13	1.30E-12
Kcnk2	3187	4	160.5	0.044552	1.677011	5.23427	Up	4.59E-05	8.81E-05
Efna2	1177	1	41	0.030889	1.160713	5.231762	Up	0.000575	0.001009
Ch25h	1341	0.5	20	0.013128	0.491899	5.227621	Up	9.54E-07	2.03E-06
LOC501529	1005	1	41.5	0.036176	1.346867	5.218439	Up	0.000575	0.001004
Bai2	5001	4	161	0.02885	1.073221	5.217222	Up	4.59E-05	8.81E-05
Ablim3	2833	1	40	0.012428	0.459962	5.209795	Up	0.000575	0.001009
Isg20	817	155.5	6169	6.821519	251.8491	5.206323	Up	3.73E-13	1.29E-12
Spata17	1197	2.5	97	0.074496	2.701772	5.180605	Up	0.000115	0.000214
Ttc23l	1455	2	79	0.049975	1.811518	5.179854	Up	0.000115	0.000214
Fam23a	1139	1	38.5	0.030913	1.12023	5.179442	Up	0.000575	0.001004
Gadd45b	1300	740.5	28691	20.35026	736.8497	5.178251	Up	0	0
Adssl1	1792	6	230	0.11853	4.284635	5.175849	Up	1.62E-05	3.20E-05
RGD1561678	8136	0	9	0.001	0.035937	5.167386	Up	0.001953	0.003247
LOC100365773	2618	1	38	0.013449	0.483231	5.167129	Up	0.000575	0.001006
Tg	8461	34	1316	0.144606	5.184168	5.163913	Up	5.99E-13	1.97E-12
Pla2g12b	1114	1.5	57	0.04741	1.699415	5.163704	Up	0.000575	0.001008
Timm8a2	856	0.5	19	0.020566	0.733702	5.156829	Up	1.91E-06	3.99E-06

Plk2	2781	3294.5	125658	42.3155	1508.456	5.155742	Up	0	0
Edn1	1390	142	5395.5	3.653903	129.4339	5.146633	Up	2.91E-13	1.03E-12
Gadd45g	1064	460	17518.5	15.52732	549.7645	5.145933	Up	1.49E-13	5.55E-13
RGD1560289	3034	6	223	0.06963	2.453348	5.13889	Up	1.62E-05	3.20E-05
Tp53inp1	1013	120	4511.5	4.218504	148.6245	5.138797	Up	0	0
Gbp4	2560	23	863.5	0.319922	11.25327	5.13648	Up	4.68E-10	1.13E-09
St8sia5	1131	4	149.5	0.12554	4.408027	5.133911	Up	4.59E-05	8.83E-05
Robo3	5682	0.5	18.5	0.003098	0.108625	5.131703	Up	3.81E-06	7.85E-06
Ankrd60	938	1	37	0.037537	1.316004	5.131703	Up	0.000575	0.001009
LOC499994	4044	0.5	18.5	0.004353	0.151955	5.125378	Up	3.81E-06	7.85E-06
RGD1562099	2609	1	38	0.013935	0.485588	5.122937	Up	0.000575	0.00101
Armcx6	1955	0.5	18.5	0.009005	0.312944	5.119025	Up	3.81E-06	7.85E-06
RGD1308775	2518	10	371.5	0.141654	4.919917	5.118188	Up	1.61E-06	3.40E-06
LOC100911416	1430	2	73	0.049244	1.701844	5.110993	Up	0.000115	0.000214
Ppp2r2c	1344	5	184.5	0.133549	4.58156	5.1004	Up	2.76E-05	5.40E-05
Dnase1	1313	1	36.5	0.026816	0.917493	5.096515	Up	0.000575	0.001011
Slc6a5	7075	1	36	0.004977	0.169883	5.093226	Up	0.000575	0.001004
Tat	2037	0.5	18	0.008643	0.295023	5.093226	Up	3.81E-06	7.85E-06
RGD1560775	746	1	36	0.047198	1.606334	5.088899	Up	0.000575	0.001011
Rsad2	3628	8	288	0.07843	2.645122	5.075777	Up	5.24E-06	1.07E-05
Ldhc	1193	2.5	91.5	0.076187	2.555023	5.067639	Up	0.000115	0.000214
Niacr1	1083	12.5	449.5	0.413805	13.86761	5.066625	Up	4.81E-07	1.04E-06
Tcte1	2697	1	35	0.013055	0.434942	5.05813	Up	0.000575	0.001007
Lgals4	1053	2	70.5	0.066875	2.226325	5.057049	Up	0.000115	0.000214
Rinl	2998	1	35	0.011744	0.390673	5.055914	Up	0.000575	0.00101

Slitrk1	4039	1	37	0.009001	0.299384	5.055702	Up	0.000575	0.001005
LOC100912995	1182	8	281.5	0.241703	7.937933	5.037459	Up	5.24E-06	1.07E-05
Vwce	2625	1	34.5	0.013413	0.439998	5.035766	Up	0.000575	0.001003
Ly49i6	1207	1	35.5	0.030122	0.981592	5.026258	Up	0.000575	0.001006
Lpar2	2836	5	173	0.062885	2.038547	5.018674	Up	2.76E-05	5.40E-05
Ceacam18-ps1	1113	4	140	0.129631	4.200408	5.018042	Up	4.59E-05	8.84E-05
Prdm1	5159	19	653	0.131007	4.23989	5.016306	Up	6.02E-09	1.41E-08
Evpl	6090	4	140.5	0.023691	0.764712	5.012489	Up	4.59E-05	8.83E-05
LOC683206	2779	744	25729	9.576664	308.9613	5.011759	Up	0	0
Sgk2	2708	2	70	0.026851	0.859703	5.000775	Up	0.000115	0.000214
LOC100911050	569	1	34	0.06188	1.977594	4.998126	Up	0.000575	0.001003
Klk7	1369	0.5	17	0.01286	0.409989	4.99466	Up	7.63E-06	1.54E-05
LOC685297	4674	1	33.5	0.007533	0.238234	4.982991	Up	0.000575	0.001003
LOC679528	600	1	34	0.058683	1.845418	4.974861	Up	0.000575	0.001003
Ngb	1773	1	33	0.019859	0.623951	4.973576	Up	0.000575	0.001002
Ptk6	2142	1	34	0.016973	0.53163	4.969089	Up	0.000575	0.001003
Myt1l	4491	1	33	0.00784	0.245528	4.968874	Up	0.000575	0.001002
Pld6	685	4	134	0.210627	6.502237	4.948171	Up	4.59E-05	8.83E-05
Afaf	1051	7	228.5	0.237237	7.277105	4.938966	Up	9.28E-06	1.87E-05
Cd209d	915	1	32	0.038481	1.165659	4.920869	Up	0.000575	0.001002
Adam32	2399	1	32	0.014677	0.443842	4.918432	Up	0.000575	0.001002
Ccdc81	2378	1	33	0.015289	0.46218	4.917912	Up	0.000575	0.001003
LOC100909810	1325	1	33	0.027439	0.826766	4.913179	Up	0.000575	0.001003
Ptpro	4871	14	450.5	0.102611	3.086432	4.910682	Up	1.40E-07	3.11E-07
RGD1559747	3298	1	31.5	0.010676	0.319022	4.901199	Up	0.000575	0.001002

Kcnh5	3505	1	32.5	0.010373	0.309193	4.89763	Up	0.000575	0.001002
Lrrtm4	2732	1	32	0.012888	0.382495	4.891351	Up	0.000575	0.001002
Sspo	15561	4	127.5	0.009272	0.272545	4.877486	Up	4.59E-05	8.80E-05
Lrp2bp	1540	4.5	142.5	0.10512	3.087903	4.876519	Up	4.59E-05	8.80E-05
RGD1305939	3237	4	124.5	0.043863	1.287306	4.875191	Up	4.59E-05	8.84E-05
Trpv1	2847	2	63	0.025138	0.736589	4.872947	Up	0.000115	0.000214
Cd80	1000	60	1880.5	2.145847	62.72495	4.86942	Up	0	0
Clk4	1416	231	7191	5.824965	169.4292	4.862289	Up	0	0
Crtam	1717	1	31.5	0.021175	0.612775	4.854955	Up	0.000575	0.001002
LOC100362909	927	1	30.5	0.037983	1.098973	4.854676	Up	0.000575	0.001001
RGD1308818	1761	11	340	0.223193	6.450662	4.853086	Up	8.84E-07	1.89E-06
LOC360998	1780	1	30.5	0.019781	0.570308	4.849569	Up	0.000575	0.001001
Gjb5	816	1	30	0.043149	1.23628	4.840526	Up	0.000575	0.001002
Bbc3	1823	244	7524.5	4.80955	137.7231	4.839725	Up	2.85E-13	1.01E-12
Lpar3	2374	4.5	136.5	0.067466	1.926306	4.835531	Up	4.59E-05	8.85E-05
Neu3	2307	12	368.5	0.186626	5.322579	4.833905	Up	4.81E-07	1.04E-06
Birc3	2636	209.5	6395	2.839683	80.96068	4.833419	Up	1.08E-13	4.16E-13
N4bp2l1	2156	24	732	0.398329	11.33165	4.830254	Up	2.47E-10	6.04E-10
Htatip2	1263	13	392.5	0.366045	10.37662	4.825172	Up	2.60E-07	5.70E-07
Zmat4	4123	1	30	0.00854	0.241185	4.819783	Up	0.000575	0.001001
RGD1566144	6187	146	4395.5	0.840144	23.70038	4.818129	Up	3.33E-14	1.35E-13
Otop1	1827	4	120.5	0.078029	2.196366	4.814959	Up	4.59E-05	8.84E-05
Fhl1	2266	1	30.5	0.015538	0.437267	4.814613	Up	0.000575	0.001001
Foxred2	1998	43	1294	0.769823	21.61839	4.811589	Up	1.18E-13	4.49E-13
Svop	2264	3	91	0.047669	1.338383	4.811289	Up	7.36E-05	0.000139

RGD1562143	636	1	30.5	0.057165	1.593314	4.800765	Up	0.000575	0.001002
Ankrd1	1749	19.5	579.5	0.398464	11.06444	4.795339	Up	6.02E-09	1.41E-08
Grap	1560	6	179	0.138363	3.837269	4.79355	Up	1.62E-05	3.21E-05
Fam71d	1551	1	30	0.023441	0.648101	4.789125	Up	0.000575	0.001001
Fam189a2	2457	2	59	0.029128	0.80355	4.78593	Up	0.000115	0.000214
Stag3	4181	1	30	0.008696	0.23913	4.781354	Up	0.000575	0.001001
Itih1	2906	1	29	0.012116	0.33225	4.777253	Up	0.000575	0.001001
Sag	1482	0.5	14.5	0.011879	0.325748	4.777253	Up	6.10E-05	0.000116
Hapln3	1363	43	1254.5	1.123422	30.73603	4.773959	Up	1.18E-13	4.49E-13
Cpne2	3767	4	116.5	0.037692	1.031222	4.77395	Up	4.59E-05	8.85E-05
LOC100361931	936	1	30	0.038843	1.060474	4.770926	Up	0.000575	0.001001
Lcn3	786	3	89	0.138766	3.777005	4.766518	Up	7.36E-05	0.000139
Myo7a	6807	5	144.5	0.0262	0.709864	4.759909	Up	2.76E-05	5.40E-05
Cplx1	2102	4	115.5	0.068094	1.832172	4.74989	Up	4.59E-05	8.84E-05
Arrdc3	1245	225	6476.5	6.469152	173.6405	4.746383	Up	0	0
Pdzd3	2121	2	57.5	0.033742	0.905324	4.745823	Up	0.000115	0.000214
Ccr5	2495	112.5	3228	1.611519	43.15865	4.743157	Up	0	0
Wdr66	3852	6	170.5	0.055142	1.474403	4.740845	Up	1.62E-05	3.21E-05
LOC100910626	1056	1	29	0.034429	0.920282	4.740394	Up	0.000575	0.001001
Fbp1	1474	1	29	0.024665	0.658696	4.739057	Up	0.000575	0.001001
Ubap1l	1383	3	87.5	0.078865	2.102238	4.736401	Up	7.36E-05	0.000139
RGD1307660	10021	0	8	0.001	0.026653	4.736252	Up	0.003906	0.006323
Muc13	2672	2	56	0.026355	0.701071	4.733432	Up	0.000115	0.000214
Eps8l3	2717	0.5	14	0.00648	0.172034	4.730656	Up	6.10E-05	0.000116
Htr2b	2021	0.5	14.5	0.008995	0.238426	4.728318	Up	6.10E-05	0.000116

Gal3st4	2207	0.5	14.5	0.008237	0.218332	4.728318	Up	6.10E-05	0.000116
Prl7a3	859	2.5	71	0.103808	2.750132	4.727507	Up	0.000115	0.000214
Clec2l	1430	1	28	0.024622	0.648693	4.719501	Up	0.000575	0.001
RGD1563667	1920	2	56	0.037274	0.981282	4.718419	Up	0.000115	0.000213
Ccdc166	1727	0.5	14	0.010194	0.268046	4.716699	Up	6.10E-05	0.000116
LOC100911162	528	3	85	0.2044	5.373066	4.716279	Up	7.36E-05	0.000139
Sptbn4	8736	23.5	663	0.096815	2.528669	4.706997	Up	4.68E-10	1.13E-09
LOC100360795	717	1	27.5	0.049107	1.282409	4.706781	Up	0.000575	0.001
Snrpel	279	1	27.5	0.1262	3.292428	4.705368	Up	0.000575	0.001
LOC501718	627	0.5	14	0.028078	0.728254	4.696929	Up	6.10E-05	0.000116
Morc1	3036	11	301.5	0.128705	3.315439	4.68706	Up	8.84E-07	1.89E-06
Dao	1646	1.5	41.5	0.032783	0.84259	4.683791	Up	0.000575	0.001007
Pipox	1716	1	27	0.020519	0.525841	4.679629	Up	0.000575	0.001
Flrt3	2846	354	9678	4.440222	113.5491	4.676541	Up	0	0
Artn	1357	3	81.5	0.078686	2.008825	4.67411	Up	7.36E-05	0.000139
Fcgr1a	1265	1	28	0.02874	0.733305	4.673258	Up	0.000575	0.001
Mrap2	1313	2.5	67	0.067041	1.707781	4.670941	Up	0.000115	0.000214
Gabrr2	1757	1	26.5	0.02004	0.508424	4.665097	Up	0.000575	0.000999
Pacrg	1360	1	28	0.026733	0.67811	4.664835	Up	0.000575	0.001001
Nod2	3625	1	27.5	0.010029	0.254397	4.664768	Up	0.000575	0.001
Tcap	997	3	82	0.108248	2.74054	4.66205	Up	7.36E-05	0.000139
LOC688981	438	1	27.5	0.083006	2.099286	4.660538	Up	0.000575	0.001
LOC685440	1942	3	79.5	0.054392	1.368384	4.652933	Up	7.36E-05	0.000139
RGD1564447	411	1.5	40	0.129898	3.266844	4.652445	Up	0.000575	0.001007
Tmem220	1411	1	26.5	0.024954	0.626081	4.649019	Up	0.000575	0.000999

LOC100910117	1566	19	507	0.431588	10.81891	4.647755	Up	6.02E-09	1.41E-08
Tm6sf2	1412	3	80.5	0.075621	1.895459	4.647624	Up	7.36E-05	0.000139
Tmem116	1357	4	106.5	0.105477	2.639165	4.645075	Up	4.59E-05	8.82E-05
Agbl2	3295	1	27	0.011034	0.275491	4.641994	Up	0.000575	0.001
Grhl3	2480	1	26	0.014197	0.354378	4.64158	Up	0.000575	0.000999
Elavl4	1444	1	27	0.024384	0.60744	4.638763	Up	0.000575	0.001
Pln	2254	7	189.5	0.1124	2.797538	4.637442	Up	9.28E-06	1.87E-05
Liph	2785	2	53	0.025697	0.637954	4.633774	Up	0.000115	0.000214
LOC100912377	3096	1	26	0.011373	0.282124	4.632688	Up	0.000575	0.001
Rps6ka6	2583	1	27	0.014075	0.348643	4.630505	Up	0.000575	0.001
Clca1	3608	1	26	0.009759	0.241091	4.62673	Up	0.000575	0.000999
Tmem130	2774	0.5	13.5	0.006553	0.161345	4.621827	Up	0.000122	0.000224
LOC100912317	1086	1	27	0.033478	0.824258	4.621827	Up	0.000575	0.001
Psors1c2	405	0.5	13.5	0.044885	1.105116	4.621827	Up	0.000122	0.000225
Tiparp	4169	1004.5	26329.5	8.57936	210.7167	4.618291	Up	0	0
RGD1562811	4805	10	264.5	0.074948	1.835871	4.614427	Up	1.61E-06	3.40E-06
Atp2a1	3457	1	26.5	0.010517	0.256581	4.608643	Up	0.000575	0.000999
Gnrh1	467	4	103.5	0.305267	7.413887	4.602089	Up	4.59E-05	8.83E-05
Il18r1	2436	112.5	2904.5	1.649373	39.80326	4.592897	Up	0	0
Gpr160	1630	1	26	0.022305	0.538072	4.592379	Up	0.000575	0.001
LOC501437	351	1	26	0.10358	2.493611	4.589415	Up	0.000575	0.001
LOC363301	842	2.5	65	0.106585	2.565609	4.589221	Up	0.000115	0.000214
Zfp867	2800	36	926	0.459251	11.02253	4.585029	Up	2.57E-13	9.17E-13
Hormad2	1668	1	25	0.021109	0.505258	4.581091	Up	0.000575	0.000999
Dnah2	13538	1	25	0.002601	0.061853	4.571817	Up	0.000575	0.000999

Ankrd2	1116	1	25	0.03155	0.750332	4.571817	Up	0.000575	0.000999
Slc17a3	2292	1.5	38	0.023293	0.552748	4.568638	Up	0.000575	0.001003
Fgd5	6131	1	26	0.00593	0.140704	4.568495	Up	0.000575	0.001
Col11a1	7673	7	179	0.032719	0.776168	4.56815	Up	9.28E-06	1.86E-05
LOC680415	5925	10	254	0.0602	1.427924	4.56801	Up	1.61E-06	3.40E-06
LOC100911400	1356	0.5	13	0.013406	0.317425	4.565481	Up	0.000122	0.000224
Pvrl4	3794	20	505	0.188782	4.44466	4.557282	Up	3.18E-09	7.52E-09
Cga	667	0.5	12.5	0.026394	0.620967	4.556227	Up	0.000244	0.00044
Lrrd1	2687	4	100.5	0.053269	1.246902	4.548916	Up	4.59E-05	8.82E-05
LOC100909712	938	283	7087.5	10.78684	251.9219	4.545633	Up	0	0
Fam169a	3206	11	271.5	0.12188	2.828063	4.536277	Up	8.84E-07	1.89E-06
Npm2	624	2	50	0.115609	2.665129	4.526882	Up	0.000115	0.000214
Myadml2	1666	17	416.5	0.363414	8.343732	4.521007	Up	2.14E-08	4.89E-08
Myct1	1560	1.5	37	0.034591	0.791867	4.516799	Up	0.000575	0.001008
Papln	4296	1	25	0.008463	0.193662	4.51624	Up	0.000575	0.000999
Dydc1	691	0.5	12	0.025477	0.582405	4.514731	Up	0.000244	0.00044
Grb7	2445	3.5	86	0.051341	1.173606	4.514701	Up	7.36E-05	0.000139
Gnal	1523	1	24	0.023119	0.526122	4.508264	Up	0.000576	0.000999
Grtp1	1111	3	72	0.095076	2.155581	4.502852	Up	7.36E-05	0.000139
Lpxn	1730	46	1120	0.953782	21.60262	4.501403	Up	7.84E-14	3.07E-13
LOC311134	5784	48	1160.5	0.297155	6.697241	4.494281	Up	1.06E-13	4.08E-13
RGD1563020	3924	0.5	12.5	0.004633	0.103487	4.481489	Up	0.000244	0.000441
Pygm	2862	12	288.5	0.150836	3.365669	4.479841	Up	4.81E-07	1.04E-06
Ano3	5764	10.5	251	0.065234	1.4509	4.475173	Up	1.61E-06	3.40E-06
Oxct2a	1779	1	23.5	0.019792	0.439258	4.472086	Up	0.000576	0.001001

Btg2	2519	479.5	11392.5	6.812937	150.8945	4.469119	Up	0	0
Calhm2	1855	1	23.5	0.018981	0.419806	4.467093	Up	0.000576	0.001001
Rbpms	1060	1	23.5	0.033217	0.73466	4.467093	Up	0.000576	0.001
LOC100360476	681	4	93.5	0.208496	4.597823	4.462856	Up	4.59E-05	8.82E-05
Chst1	2395	0.5	12	0.00759	0.166907	4.458776	Up	0.000244	0.00044
RGD1566325	3972	13	305	0.116393	2.555533	4.456542	Up	2.60E-07	5.70E-07
LOC687424	4014	6	142.5	0.054059	1.186266	4.455747	Up	1.62E-05	3.21E-05
Ebi3	1198	9	214	0.272173	5.958662	4.452393	Up	2.92E-06	6.06E-06
Guca2b	526	0.5	11.5	0.033469	0.729084	4.445171	Up	0.000488	0.000864
Dsg2	4210	1	23	0.008363	0.181757	4.441782	Up	0.000576	0.001001
LOC100362565	6522	7.5	176.5	0.041545	0.901797	4.440066	Up	9.28E-06	1.87E-05
LOC680456	2512	4	92	0.056523	1.224915	4.437697	Up	4.59E-05	8.85E-05
LOC501441	696	0.5	11.5	0.025294	0.547124	4.434978	Up	0.000488	0.000864
LOC100910163	1289	27	623.5	0.748198	16.15262	4.432205	Up	3.64E-11	9.17E-11
Ebf2	5439	11	253.5	0.072053	1.555177	4.43188	Up	8.84E-07	1.89E-06
Ankrd7	840	1	22.5	0.041916	0.902326	4.428061	Up	0.000578	0.001002
Neurl3	1394	77	1775.5	1.97696	42.44285	4.424166	Up	0	0
Glt8d2	1420	3	69	0.076002	1.621683	4.415307	Up	7.36E-05	0.000139
Cage1	2890	7	161	0.087267	1.856532	4.411022	Up	9.28E-06	1.87E-05
Olr1076	957	1	23	0.03799	0.808043	4.41073	Up	0.000576	0.001
Cryba4	800	12	273.5	0.536748	11.4158	4.410642	Up	4.81E-07	1.04E-06
Chrne	1624	6	134	0.130085	2.752606	4.403267	Up	1.62E-05	3.20E-05
Chrm1	1383	1	23	0.026288	0.551986	4.392141	Up	0.000576	0.001
LOC100910596	424	13	288.5	1.08766	22.75648	4.386977	Up	2.60E-07	5.69E-07
Myh3	6035	6	134.5	0.035576	0.744155	4.386639	Up	1.62E-05	3.21E-05

LOC100911312	735	1	22	0.047904	0.999333	4.382733	Up		0.000578	0.001002
Fam19a2	1677	2	44	0.041991	0.875981	4.382733	Up		0.000115	0.000214
LOC100909904	599	3	67.5	0.180172	3.75853	4.382721	Up		7.36E-05	0.000139
Pkhd1l1	13006	2	44	0.005414	0.112257	4.373867	Up		0.000115	0.000214
RGD1561145	1356	1	22	0.025966	0.537691	4.372087	Up		0.000578	0.001002
LOC680899	2260	1	22	0.01558	0.322615	4.372087	Up		0.000578	0.001002
RGD1564472	1815	3	66	0.05883	1.212082	4.364794	Up		7.36E-05	0.000139
Lppr3	2597	2	43	0.027116	0.555923	4.357683	Up		0.000115	0.000214
Gjc2	2081	6	131.5	0.103171	2.111685	4.355281	Up		1.62E-05	3.20E-05
LOC100912207	574	2.5	55	0.155351	3.173997	4.352699	Up		0.000115	0.000214
Atp10b	6756	3	66.5	0.016144	0.328563	4.347086	Up		7.36E-05	0.000139
Hecw2	5296	29	636	0.196917	4.006044	4.346516	Up		1.04E-11	2.73E-11
Stac	2184	2	43.5	0.032769	0.666427	4.346064	Up		0.000115	0.000214
Ros1	7902	6	131.5	0.027388	0.555203	4.341402	Up		1.62E-05	3.21E-05
LOC681177	1524	11.5	247.5	0.267196	5.415279	4.341066	Up		8.84E-07	1.89E-06
RGD1308454	1394	0.5	10.5	0.012629	0.255676	4.339496	Up		0.000977	0.001659
Zfp541	4318	7	152	0.057876	1.171513	4.339261	Up		9.28E-06	1.87E-05
LOC100910986	1401	4	86.5	0.101346	2.047544	4.33653	Up		4.59E-05	8.81E-05
Col4a3	5020	1	22	0.007242	0.145958	4.33295	Up		0.000578	0.001002
Tas2r108	915	2	43	0.078215	1.568014	4.325355	Up		0.000115	0.000214
Hmcn2	15680	21	450.5	0.047814	0.955587	4.320875	Up		1.68E-09	4.01E-09
Txndc2	1807	1.5	32.5	0.03018	0.602724	4.319839	Up		0.000575	0.001002
Nkapl	1519	1	21	0.02318	0.460976	4.313766	Up		0.000581	0.001006
Megf6	5523	4	84.5	0.025916	0.51431	4.310736	Up		4.59E-05	8.83E-05
RGD1562511	3475	7	148.5	0.072246	1.429206	4.306146	Up		9.28E-06	1.87E-05

Mmp25	1503	4	84	0.094468	1.86593	4.303918	Up	4.59E-05	8.81E-05
Nalcn	6779	6	126.5	0.031502	0.620959	4.30098	Up	1.62E-05	3.21E-05
Ccno	2410	7	147	0.103221	2.034216	4.300666	Up	9.28E-06	1.87E-05
LOC680585	210	1	21	0.167666	3.300108	4.298854	Up	0.000581	0.001006
LOC316124	2611	2	41	0.02697	0.5253	4.283696	Up	0.000115	0.000214
Tex14	4748	5	105.5	0.038045	0.74024	4.282223	Up	2.76E-05	5.40E-05
Tmem82	1110	1	20.5	0.031721	0.615387	4.278005	Up	0.000586	0.001015
LOC100359836	3364	1	20.5	0.010467	0.203056	4.278005	Up	0.000586	0.001015
Rdh8	1158	2	41.5	0.061802	1.197671	4.276439	Up	0.000115	0.000214
RGD1559884	2530	4.5	92.5	0.063079	1.221909	4.275824	Up	4.59E-05	8.82E-05
LOC100362279	835	3	61	0.126502	2.445504	4.272897	Up	7.36E-05	0.000139
Krt40	1296	1.5	31	0.041195	0.795826	4.271929	Up	0.000575	0.001002
LOC498350	2686	4	82.5	0.053289	1.029323	4.271728	Up	4.59E-05	8.83E-05
LOC100360307	4793	1	21	0.007585	0.146468	4.271227	Up	0.000581	0.001006
Lig4	3192	380	7839	4.254879	81.95332	4.267613	Up	0	0
LOC171573	619	1	20.5	0.056882	1.094797	4.266554	Up	0.000586	0.001015
Svep1	11265	2	42	0.006455	0.124159	4.265667	Up	0.000115	0.000214
Macc1	2792	2	40.5	0.025222	0.483817	4.26171	Up	0.000115	0.000213
RGD1307937	2101	3	62	0.051368	0.985237	4.261542	Up	7.36E-05	0.000139
LOC100910424	1008	20.5	420	0.72745	13.90849	4.256974	Up	3.18E-09	7.52E-09
Rps6kl1	1724	1	20	0.020423	0.389406	4.252987	Up	0.000586	0.001015
Adcy8	4601	1	20	0.007653	0.14552	4.249113	Up	0.000586	0.001015
Wt1	2645	23	466	0.311375	5.873631	4.237526	Up	4.68E-10	1.13E-09
RGD1562865	3764	13	262	0.12313	2.316776	4.233863	Up	2.60E-07	5.70E-07
Gatsl3	1546	3	59.5	0.068324	1.282319	4.230212	Up	7.36E-05	0.000139

RGD1311447	934	11	219	0.417746	7.838985	4.229968	Up		8.84E-07	1.89E-06
Zfp354a	2561	81	1626.5	1.13512	21.17895	4.221714	Up		5.82E-14	2.30E-13
LOC100363294	947	3	59	0.111541	2.081016	4.221641	Up		7.36E-05	0.000139
Mamdc2	3295	29	576.5	0.314762	5.844567	4.214763	Up		1.04E-11	2.73E-11
Ccnl1	2128	1765.5	34997	29.59265	548.4395	4.212022	Up		3.87E-12	1.09E-11
LOC100322897	2013	1	20	0.018061	0.3335	4.206744	Up		0.000586	0.001015
RGD1559690	3368	766	15025.5	8.113831	148.9549	4.198349	Up		1.15E-12	3.59E-12
Tas2r137	951	1.5	29	0.055536	1.019051	4.19766	Up		0.000575	0.001001
LOC100912247	525	91	1783	6.17949	113.2577	4.195977	Up		0	0
Cckar	1502	2	39	0.047647	0.868699	4.188385	Up		0.000115	0.000213
Sdcbp2	1444	14	270	0.342958	6.247673	4.187216	Up		1.40E-07	3.11E-07
Tmem30c	1291	2	39.5	0.056323	1.021866	4.181331	Up		0.000115	0.000213
RGD1565374	1151	2	39.5	0.063174	1.146159	4.181331	Up		0.000115	0.000213
Cyp3a9	1976	164	3166	2.95071	53.48823	4.180087	Up		1.50E-13	5.56E-13
RGD1308195	548	1.5	28.5	0.096377	1.747034	4.180072	Up		0.000575	0.001001
Nppb	628	72.5	1408.5	4.12783	74.80298	4.17964	Up		0	0
LOC679582	1011	1	19	0.034827	0.630118	4.177357	Up		0.000596	0.001032
Fam154b	2808	10	192.5	0.127025	2.287682	4.170705	Up		1.61E-06	3.40E-06
RGD1561343	935	1	19	0.037658	0.677486	4.169181	Up		0.000596	0.001031
LOC100913030	1090	1	19	0.032303	0.579495	4.165075	Up		0.000596	0.001031
Trpm6	6441	7	132	0.038622	0.685058	4.148743	Up		9.28E-06	1.87E-05
LOC365238	2462	176	3321	2.540786	45.03997	4.147859	Up		0	0
Lekr1	2542	26	494	0.365997	6.484691	4.147135	Up		6.87E-11	1.72E-10
Myo1a	3332	8	151.5	0.08557	1.513311	4.144461	Up		5.24E-06	1.07E-05
LOC100910531	571	3	57	0.189007	3.337563	4.142282	Up		7.36E-05	0.000139

RGD1560166	1666	2	38	0.042957	0.758282	4.141768	Up		0.000115	0.000213
Akr1c19	1355	48	901	1.258711	22.18975	4.139875	Up		1.06E-13	4.07E-13
LOC100363223	867	1	19	0.041934	0.738927	4.139244	Up		0.000596	0.001032
Reep6	2152	1	18.5	0.016361	0.28806	4.138001	Up		0.000615	0.001063
Fos	1589	171	3233.5	3.861995	67.82278	4.134352	Up		3.66E-13	1.27E-12
LOC100909860	478	12	225	0.898324	15.7099	4.128295	Up		4.81E-07	1.04E-06
Sh3bgr	548	0.5	9.5	0.033172	0.579607	4.127031	Up		0.001953	0.003239
LOC100911777	1095	9	168	0.293585	5.119894	4.124263	Up		2.92E-06	6.06E-06
G6pc	2293	11.5	214.5	0.179587	3.129272	4.123069	Up		8.84E-07	1.88E-06
RGD1309621	5238	1599.5	29769.5	10.9005	189.7277	4.121465	Up		5.57E-12	1.51E-11
Upk1a	1204	2	37	0.058964	1.026007	4.121054	Up		0.000115	0.000213
LOC100361741	642	8	148.5	0.444111	7.712136	4.11814	Up		5.24E-06	1.07E-05
RGD1566386	2940	47.5	883.5	0.578423	10.03048	4.116121	Up		0	0
Pla2g4f	2583	11	204	0.151721	2.629156	4.115107	Up		8.84E-07	1.89E-06
Slc7a14	2812	0.5	9.5	0.006465	0.111993	4.114715	Up		0.001953	0.003242
Gal3st1	1858	2	37	0.038518	0.667283	4.114694	Up		0.000115	0.000213
Nrg3	3601	1	19	0.010096	0.17441	4.110586	Up		0.000596	0.001031
Gpr35	921	0.5	9	0.019115	0.330164	4.110409	Up		0.001953	0.003238
LOC100911522	496	1	18	0.070987	1.22432	4.108272	Up		0.000615	0.001063
Zmynd15	2879	36.5	676	0.45376	7.825593	4.108199	Up		2.57E-13	9.18E-13
LOC100910729	4281	5	93	0.041927	0.722559	4.107162	Up		2.76E-05	5.40E-05
RGD1559875	1704	1	18	0.020663	0.355847	4.106132	Up		0.000615	0.001063
Camk4	1803	4	75	0.080658	1.388303	4.105358	Up		4.59E-05	8.83E-05
Arpp21	3204	15.5	284.5	0.17284	2.970068	4.102985	Up		7.51E-08	1.68E-07
Zfp385b	3803	3	56	0.02868	0.491155	4.098061	Up		7.36E-05	0.000139

Has1	2095	4	73.5	0.068321	1.168535	4.096223	Up	4.59E-05	8.84E-05
Ccdc114	2618	2	36.5	0.027336	0.467024	4.094606	Up	0.000115	0.000213
LOC100910982	968	5.5	100	0.201833	3.446261	4.093799	Up	2.76E-05	5.39E-05
Eda2r	1082	90.5	1653.5	2.990575	50.99514	4.091865	Up	0	0
Adipoq	1487	0.5	9	0.011839	0.201467	4.088899	Up	0.001953	0.003236
Ostalpha	1603	0.5	9	0.010982	0.186888	4.088899	Up	0.001953	0.003236
Emilin3	2440	2	37	0.029801	0.506644	4.087562	Up	0.000115	0.000213
Kcna7	3592	1	18	0.009802	0.166304	4.084558	Up	0.000615	0.001063
Dll3	1998	7.5	138.5	0.136474	2.305567	4.078424	Up	9.28E-06	1.86E-05
Lrrc66	2598	12.5	223.5	0.170733	2.883797	4.07816	Up	4.81E-07	1.04E-06
RGD1564786	682	4.5	82	0.237368	4.008971	4.078037	Up	4.59E-05	8.82E-05
Gdf15	912	22	400	0.866965	14.58215	4.072087	Up	8.87E-10	2.13E-09
Rnd1	1639	132	2387	2.891318	48.60233	4.071226	Up	1.14E-13	4.37E-13
LOC100360291	846	9.5	170.5	0.399449	6.711112	4.070469	Up	2.92E-06	6.06E-06
Kbtbd8	3510	53	950	0.538193	9.03324	4.069049	Up	4.04E-14	1.62E-13
Adcy10	5177	3	53.5	0.020404	0.34233	4.068494	Up	7.36E-05	0.000139
Casc1	2433	35	626	0.51264	8.57733	4.064512	Up	3.77E-13	1.30E-12
Fgf12	732	5.5	98.5	0.268471	4.485862	4.062545	Up	2.76E-05	5.41E-05
LOC365659	519	0.5	9	0.035026	0.58243	4.0556	Up	0.001953	0.003241
Fbxo17	1639	1.5	26	0.032224	0.534568	4.052179	Up	0.000575	0.000999
Dnah5	14532	8.5	150.5	0.020871	0.345738	4.050108	Up	5.24E-06	1.07E-05
RGD1560958	649	4.5	79.5	0.24767	4.09461	4.047235	Up	4.59E-05	8.84E-05
LOC100910746	1161	1	18	0.031315	0.517625	4.046983	Up	0.000615	0.001063
Naip5	4209	6	106.5	0.05101	0.842966	4.046634	Up	1.62E-05	3.21E-05
Rassf9	1790	57	1002	1.136583	18.72485	4.04218	Up	5.37E-14	2.13E-13

Gpr156	2379	2	35	0.030083	0.49535	4.041449	Up	0.000115	0.000213
Dlgap1	4181	60.5	1068	0.518271	8.525787	4.040053	Up	0	0
Lrrc7	5109	3	52.5	0.0209	0.342113	4.032925	Up	7.36E-05	0.000139
Smr3a	660	0.5	8.5	0.026674	0.436118	4.031209	Up	0.003906	0.006337
Gcgr	1893	9	158	0.171641	2.789496	4.022539	Up	2.92E-06	6.06E-06
LOC100912365	1059	35	608.5	1.181013	19.19039	4.022287	Up	3.77E-13	1.30E-12
Tuba8	1478	0.5	8.5	0.011911	0.19353	4.022158	Up	0.003906	0.006336
Plek2	1553	3	51	0.068016	1.101628	4.017611	Up	7.36E-05	0.000139
Abca17	5322	1.5	26	0.010139	0.164122	4.016733	Up	0.000575	0.000999
RGD1564013	837	49	853.5	2.102373	33.98207	4.014683	Up	3.91E-14	1.57E-13
RGD1563155	555	1	17	0.063441	1.024281	4.01305	Up	0.000651	0.001123
Hils1	797	5	86	0.223768	3.604851	4.009867	Up	2.76E-05	5.40E-05
Tnfsf13b	1254	1	17.5	0.028993	0.467001	4.009671	Up	0.000651	0.001124
Hnf1a	3538	1	17	0.009952	0.160168	4.008475	Up	0.000651	0.001124
Ldhal6b	1442	1	17	0.024417	0.392355	4.006181	Up	0.000651	0.001123
Tmprss9	3341	6	103	0.064262	1.031171	4.004173	Up	1.62E-05	3.20E-05
LOC100912988	605	13	221.5	0.763209	12.23757	4.003095	Up	2.60E-07	5.70E-07
Cdh16	2894	3	52	0.037292	0.596169	3.998783	Up	7.36E-05	0.000139
Apoa1	889	10	170	0.403156	6.394554	3.987433	Up	1.61E-06	3.40E-06
LOC501046	1543	26	440	0.601471	9.521126	3.984564	Up	6.87E-11	1.72E-10
Sim1	7666	8	133.5	0.036893	0.581945	3.979446	Up	5.24E-06	1.07E-05
Ppp1r1c	2848	2	33.5	0.024726	0.389715	3.978319	Up	0.000115	0.000213
Ttll3	2906	37.5	629	0.45949	7.223126	3.974519	Up	1.86E-13	6.81E-13
LOC100912479	1385	3	50	0.077095	1.211799	3.974373	Up	7.36E-05	0.000139
Selplg	1263	3	51.5	0.086358	1.356746	3.973679	Up	7.36E-05	0.000139

RGD1564937	1639	1	16.5	0.021482	0.335834	3.966517	Up		0.00072	0.001238
LOC100910797	621	142	2372.5	8.160156	127.4114	3.964753	Up		2.91E-13	1.03E-12
RGD1564149	1532	1	17	0.023732	0.369893	3.962232	Up		0.000651	0.001123
RGD1564894	1950	3	49.5	0.054757	0.853281	3.961904	Up		7.36E-05	0.000139
Lhx4	1790	1	16.5	0.01967	0.305996	3.959422	Up		0.00072	0.001238
Creb5	4148	153	2548.5	1.321118	20.49579	3.955497	Up		0	0
Myoc	2068	2	33	0.034607	0.534944	3.950269	Up		0.000115	0.000213
LOC100362676	3178	1	17	0.01144	0.176046	3.943783	Up		0.000651	0.001124
RGD1561909	2286	244	4001.5	3.806088	58.41062	3.93985	Up		2.85E-13	1.01E-12
Dscam	6656	1	17	0.005462	0.083515	3.934469	Up		0.000651	0.001123
Isyna1	1881	19.5	320	0.372635	5.688932	3.932322	Up		6.02E-09	1.41E-08
Galnt6	3387	1	16	0.010396	0.158249	3.928155	Up		0.00072	0.001237
Mslnl	2307	7	113.5	0.108326	1.646274	3.925746	Up		9.28E-06	1.87E-05
Gpr45	3470	1	16.5	0.010477	0.158626	3.920273	Up		0.00072	0.001238
LOC687483	743	4	65	0.192642	2.914728	3.919368	Up		4.59E-05	8.84E-05
Evc2	4067	1	16	0.008657	0.130905	3.918432	Up		0.00072	0.001238
Asz1	1725	1	16.5	0.021076	0.318048	3.915548	Up		0.00072	0.001238
Doc2g	1487	1	16	0.023678	0.356818	3.913546	Up		0.00072	0.001238
Cdo1	1458	11	177	0.269576	4.058687	3.912248	Up		8.84E-07	1.88E-06
Crb1	4603	18	290	0.140179	2.105404	3.908753	Up		1.14E-08	2.63E-08
Lin7b	624	2	31.5	0.112852	1.694767	3.908584	Up		0.000115	0.000213
Gpr22	4537	2	32.5	0.015774	0.236482	3.906116	Up		0.000115	0.000213
LOC100365683	931	0.5	8	0.019526	0.29269	3.905936	Up		0.003906	0.006339
RGD1562977	870	1	16	0.040471	0.605734	3.903723	Up		0.00072	0.001238
Zfp455	2185	129.5	2066	2.109375	31.56066	3.90324	Up		0	0

Ephx1	1732	2	32	0.04132	0.617886	3.902423	Up		0.000115	0.000213
Exd1	3435	11.5	184	0.120216	1.793384	3.898988	Up		8.84E-07	1.89E-06
Ccdc30	2136	4	63.5	0.066473	0.990221	3.896913	Up		4.59E-05	8.85E-05
RGD1564265	2764	19.5	309.5	0.251517	3.740944	3.894675	Up		6.02E-09	1.41E-08
Pbld1	2010	2	32.5	0.036176	0.537822	3.894031	Up		0.000115	0.000213
Cldn11	1873	16	252	0.303839	4.495343	3.88705	Up		4.01E-08	9.10E-08
Sele	3351	1	16	0.010849	0.160486	3.886749	Up		0.00072	0.001238
Slc24a1	3550	1	16	0.010241	0.15149	3.886749	Up		0.00072	0.001238
Cox6b2	483	1	15.5	0.072898	1.077941	3.886253	Up		0.000849	0.001453
LOC100362405	255	2.5	40	0.345194	5.103024	3.885873	Up		0.000115	0.000213
LOC366632	756	1	15.5	0.046574	0.686304	3.881257	Up		0.000849	0.001451
RGD1304624	1111	46.5	729	1.488129	21.91376	3.880265	Up		7.84E-14	3.07E-13
Fam169b	1026	9	141	0.311093	4.580351	3.880038	Up		2.92E-06	6.06E-06
LOC501223	1432	1	15.5	0.024588	0.361694	3.878752	Up		0.000849	0.001453
Nup210l	5646	3	47	0.018912	0.278087	3.878175	Up		7.36E-05	0.000139
LOC100912412	1194	3	47.5	0.090388	1.328579	3.877611	Up		7.36E-05	0.000139
LOC100361584	3101	61	957.5	0.701491	10.30178	3.876326	Up		3.49E-14	1.41E-13
LOC685796	1379	1	15.5	0.025533	0.374942	3.876243	Up		0.000849	0.001452
Mdm2	2855	3202	50279	40.04198	587.6306	3.875324	Up		0	0
Csrnp1	2872	465.5	7302.5	5.78455	84.81791	3.874092	Up		0	0
RGD1562532	2433	1	15.5	0.014472	0.212144	3.873729	Up		0.000849	0.001453
Runx1t1	3364	7.5	117.5	0.079523	1.160939	3.867783	Up		9.28E-06	1.87E-05
Atp6v1c2	1595	16	248	0.356078	5.19005	3.865484	Up		4.01E-08	9.10E-08
Ctsk	1446	9	138.5	0.219941	3.190057	3.858392	Up		2.92E-06	6.06E-06
Mbp	1495	18	280	0.430451	6.239202	3.857439	Up		1.14E-08	2.63E-08

LOC100910316	2327	6	93	0.092264	1.335098	3.855027	Up	1.62E-05	3.21E-05
Dok3	1527	9	140.5	0.212405	3.07341	3.854949	Up	2.92E-06	6.06E-06
LOC100360880	3251	187.5	2902	2.062459	29.80878	3.853299	Up	3.37E-13	1.18E-12
LOC100360619	309	2	31	0.231607	3.346573	3.852936	Up	0.000115	0.000213
RGD1565866	4989	1	16	0.007287	0.105269	3.852544	Up	0.00072	0.001237
LOC680810	1019	3.5	54	0.122625	1.768388	3.850113	Up	7.36E-05	0.000139
LOC100360238	2454	5	76.5	0.072674	1.043171	3.843386	Up	2.76E-05	5.41E-05
Arid3a	2387	41	631.5	0.615107	8.810314	3.840285	Up	1.47E-13	5.48E-13
Hmcn1	18310	0	8	0.001	0.014292	3.837179	Up	0.003906	0.006332
Grm1	6820	5	76.5	0.02615	0.37351	3.836268	Up	2.76E-05	5.40E-05
Oscar	2029	18	275.5	0.316881	4.525538	3.836077	Up	1.14E-08	2.63E-08
Sema4d	4187	2	30	0.016819	0.240077	3.835368	Up	0.000116	0.000213
Aox3	4728	40	608.5	0.301158	4.295881	3.834362	Up	1.16E-13	4.44E-13
Clic2	1219	2	30	0.057768	0.823137	3.832782	Up	0.000116	0.000213
Mab21l1	2390	1	15	0.014732	0.209164	3.827597	Up	0.000849	0.001452
Krt17	1536	13.5	205.5	0.313941	4.450676	3.825459	Up	2.60E-07	5.70E-07
Ccdc63	2122	1	15	0.016593	0.235157	3.824997	Up	0.000849	0.001451
Il31ra	2444	2	30	0.028813	0.408349	3.824997	Up	0.000116	0.000213
LOC688328	2718	1	15.5	0.013376	0.189568	3.824968	Up	0.000849	0.001451
Btk	1983	1	15	0.017756	0.251186	3.822393	Up	0.000849	0.001452
Cdh7	2361	4.5	68.5	0.068566	0.968465	3.820132	Up	4.59E-05	8.82E-05
Ddit3	813	418	6313.5	18.38861	259.2675	3.817557	Up	5.87E-13	1.94E-12
Rsph4a	2215	1	15	0.015896	0.224064	3.817169	Up	0.000849	0.001452
LOC690930	651	6	90	0.328038	4.615688	3.814612	Up	1.62E-05	3.20E-05
LOC680643	1072	3	44.5	0.098535	1.386346	3.814509	Up	7.36E-05	0.000139

Tas1r1	2679	3	45	0.040285	0.565176	3.810391	Up	7.36E-05	0.000139
Ces1d	1938	4.5	67.5	0.082644	1.156119	3.806234	Up	4.59E-05	8.81E-05
Got1l1	1450	1.5	22.5	0.037215	0.520244	3.805236	Up	0.000578	0.001002
Ncf1	2800	9	135.5	0.115632	1.61488	3.803815	Up	2.92E-06	6.07E-06
Mok	1648	66.5	1000	1.448968	20.22294	3.802895	Up	0	0
Vwde	5079	14	209	0.098635	1.375811	3.802044	Up	1.40E-07	3.11E-07
LOC100911864	1115	68	1014	2.17921	30.3585	3.800223	Up	0	0
Olr813	942	4.5	66	0.1682	2.336337	3.796002	Up	4.59E-05	8.81E-05
Notch3	7319	2	30	0.009778	0.13562	3.793863	Up	0.000116	0.000213
LOC691138	1545	1	15	0.023532	0.325891	3.791706	Up	0.000849	0.001453
Teddm1	1258	1	15	0.0289	0.40024	3.791706	Up	0.000849	0.001453
LOC100909587	2795	32	475	0.408862	5.659154	3.7909	Up	1.74E-12	5.25E-12
Hao1	1527	4.5	67	0.10639	1.468446	3.78685	Up	4.59E-05	8.81E-05
LOC100912425	573	1	15	0.06345	0.875571	3.786539	Up	0.000849	0.001452
LOC299727	1248	1	15	0.029132	0.401284	3.783949	Up	0.000849	0.001452
Lrcol1	806	1	15	0.045108	0.620226	3.781354	Up	0.000849	0.001451
Naaladl1	2570	9	132.5	0.125088	1.719378	3.780874	Up	2.92E-06	6.06E-06
LOC689561	1761	5	73	0.100622	1.382985	3.780764	Up	2.76E-05	5.40E-05
Slc16a6	2034	41	601.5	0.719885	9.884522	3.779334	Up	1.47E-13	5.48E-13
RGD1308065	1605	1	15	0.022652	0.310344	3.776149	Up	0.000849	0.001453
Rhcg	1958	2	29	0.035965	0.492195	3.774561	Up	0.000116	0.000213
Tmem22	1593	1	14.5	0.022103	0.301355	3.769162	Up	0.001094	0.00185
LOC257650	1948	35	510	0.640862	8.728888	3.767712	Up	3.77E-13	1.30E-12
Ctrc	898	4	57.5	0.158114	2.150325	3.765521	Up	4.59E-05	8.81E-05
Abcb1b	3888	3902	56893.5	35.93288	488.3333	3.76449	Up	4.26E-12	1.19E-11

Cdkl1	1861	10	144	0.189815	2.579527	3.764444	Up	1.61E-06	3.40E-06
Actc1	1489	1	15	0.024417	0.330894	3.760423	Up	0.000849	0.001452
LOC687615	1008	2	29	0.070998	0.961426	3.759316	Up	0.000116	0.000213
Degs2	1317	1	14.5	0.026735	0.361776	3.758302	Up	0.001094	0.001849
Wsb1	1890	2523	36355	47.54963	642.227	3.755577	Up	0	0
Kin	1396	210	3043	5.388615	72.76559	3.75527	Up	0	0
Lefty2	1384	83.5	1208.5	2.161166	29.15738	3.753979	Up	1.43E-13	5.34E-13
Enthd1	2693	14.5	209	0.192136	2.591438	3.75355	Up	1.40E-07	3.11E-07
Pspn	471	10.5	153.5	0.806847	10.87988	3.753225	Up	1.61E-06	3.40E-06
Nlgn3	3166	19.5	283	0.220849	2.974121	3.751334	Up	6.02E-09	1.41E-08
RGD1559708	492	2.5	37	0.182408	2.454087	3.749945	Up	0.000115	0.000213
Fam71f2	1260	1	14	0.027944	0.37525	3.747229	Up	0.001094	0.001848
Ush2a	15658	1.5	22	0.003483	0.046737	3.746214	Up	0.000578	0.001003
Ccl7	807	1563.5	22326	68.97515	923.8311	3.74348	Up	3.52E-12	1.00E-11
LOC498675	1023	12.5	178	0.435833	5.830355	3.741738	Up	4.81E-07	1.04E-06
Psmb11	2616	45.5	646.5	0.618979	8.254252	3.737175	Up	2.49E-14	1.02E-13
Egr4	2145	5	72	0.083678	1.115641	3.736878	Up	2.76E-05	5.41E-05
Tnfsf18	603	142.5	2024	8.410115	112.0615	3.736022	Up	2.91E-13	1.03E-12
Lrrc2	2528	2	28.5	0.02831	0.376216	3.732202	Up	0.000116	0.000214
Pnpla3	3308	55	782.5	0.594078	7.893631	3.731964	Up	0	0
LOC500350	1230	33	466.5	0.953977	12.66292	3.730512	Up	1.01E-12	3.19E-12
Hhatl	1773	1.5	21	0.029788	0.394937	3.728803	Up	0.000581	0.001006
Mterf	1506	192.5	2725.5	4.563796	60.41826	3.726679	Up	0	0
Hal	2136	1	14	0.016484	0.217985	3.72509	Up	0.001094	0.001848
Galr3	1163	1	14	0.030275	0.400357	3.72509	Up	0.001094	0.001849

Nr1d1	2344	1114.5	15824.5	17.06704	225.3012	3.72257	Up	3.05E-12	8.83E-12
Zfp2	2106	18	253	0.304206	4.01513	3.722327	Up	1.14E-08	2.63E-08
Matn4	2292	1	14	0.015362	0.202363	3.719501	Up	0.001094	0.00185
Rbp1	695	1	14	0.050662	0.66736	3.719501	Up	0.001094	0.001849
Gadd45a	1237	1170	16485.5	33.75702	444.6699	3.719476	Up	2.96E-12	8.57E-12
LOC680627	3717	23	322.5	0.220031	2.894215	3.717396	Up	4.68E-10	1.13E-09
Prodh	2182	1	14	0.016136	0.212152	3.716699	Up	0.001094	0.00185
Tcte3	423	3	41.5	0.249715	3.276602	3.713847	Up	7.36E-05	0.000139
LOC100366216	1055	9.5	133.5	0.320316	4.200463	3.712979	Up	2.92E-06	6.07E-06
Tssk3	1003	2	28	0.071352	0.93024	3.704569	Up	0.000116	0.000214
Kcnb2	3230	1	14.5	0.011256	0.146674	3.70386	Up	0.001094	0.001848
Cd28	1284	453	6310	12.5923	164.0822	3.703805	Up	1.56E-12	4.76E-12
Cercam	2750	20.5	286.5	0.267061	3.47388	3.701309	Up	3.18E-09	7.53E-09
Usp51	2310	1	14	0.015739	0.204682	3.700986	Up	0.001094	0.001848
Dapk2	1302	6	83	0.164019	2.131801	3.70014	Up	1.62E-05	3.20E-05
LOC498601	463	8	111	0.615808	7.99835	3.69915	Up	5.24E-06	1.07E-05
Bmpr1b	1529	38.5	535	0.900079	11.66916	3.696505	Up	1.45E-13	5.43E-13
Qrich2	5940	4	56	0.02429	0.314758	3.695836	Up	4.59E-05	8.82E-05
Flt3	3668	1	14	0.009912	0.128412	3.695483	Up	0.001094	0.00185
Zfp51	2095	16	221.5	0.273285	3.520682	3.687377	Up	4.01E-08	9.10E-08
Zfp36	1811	205.5	2843	4.073896	52.42424	3.685753	Up	4.23E-13	1.44E-12
LOC292722	2296	1	14	0.015835	0.203578	3.684413	Up	0.001094	0.00185
Frem3	6595	1	14	0.005513	0.070874	3.684413	Up	0.001094	0.001849
Prkcg	3113	38	524.5	0.438459	5.634929	3.683883	Up	1.45E-13	5.43E-13
Iqcg	1643	3	41.5	0.065687	0.844128	3.683791	Up	7.36E-05	0.000139

LOC100911521	811	20	274	0.879619	11.30097	3.683424	Up	3.18E-09	7.52E-09
Calr4	1660	10	136	0.21418	2.746691	3.6808	Up	1.61E-06	3.40E-06
Clk1	1791	1149	15750.5	22.88757	293.5026	3.680737	Up	3.45E-12	9.87E-12
Dhh	2479	232	3178	3.337248	42.7718	3.679929	Up	3.80E-13	1.31E-12
RGD1560556	1518	1	14	0.02395	0.306729	3.678847	Up	0.001094	0.00185
Ddit4	1754	643.5	8810.5	13.11607	167.5313	3.675023	Up	0	0
Ccdc62	2896	105.5	1446	1.30327	16.64611	3.674977	Up	0	0
Shc2	3904	2	27.5	0.018332	0.234141	3.674976	Up	0.000116	0.000214
Pou2af1	2527	4	54.5	0.056188	0.716672	3.67299	Up	4.59E-05	8.84E-05
Itga2b	3317	4	54.5	0.043151	0.549783	3.671387	Up	4.59E-05	8.82E-05
Tuba4a	1477	28	383	0.680685	8.670921	3.671127	Up	1.93E-11	4.97E-11
MGC114499	2472	1	13.5	0.014243	0.181421	3.670969	Up	0.001551	0.002597
Ddx3y	3087	308.5	4197.5	3.562538	45.36381	3.670565	Up	0	0
Tas2r103	939	2.5	34.5	0.096186	1.223317	3.668835	Up	0.000115	0.000213
Rgs9	2424	3	40.5	0.043576	0.55244	3.664197	Up	7.36E-05	0.000139
Myo15	10224	4.5	61.5	0.015722	0.198938	3.661494	Up	4.59E-05	8.82E-05
Pax8	2517	5	68	0.071311	0.901988	3.660913	Up	2.76E-05	5.40E-05
LOC100910200	1329	216	2895.5	5.745022	72.62254	3.660033	Up	2.34E-13	8.42E-13
Zfp819	2366	23	309	0.345185	4.361464	3.659373	Up	4.68E-10	1.13E-09
Gsto2	1415	9	123	0.229623	2.899631	3.658531	Up	2.92E-06	6.06E-06
LOC100910239	673	5	68	0.268405	3.382769	3.655722	Up	2.76E-05	5.41E-05
RGD1563354	3762	3	41	0.028993	0.365061	3.654383	Up	7.36E-05	0.000139
LOC100912866	798	2	27	0.089682	1.126245	3.650554	Up	0.000116	0.000214
Ccdc11	1856	12	161.5	0.231975	2.907823	3.647898	Up	4.81E-07	1.04E-06
Grin2d	5246	1	13.5	0.00693	0.086861	3.647704	Up	0.001551	0.002597

Icam4	845	4	53.5	0.169388	2.120763	3.646179	Up	4.59E-05	8.84E-05
Piwil1	4005	54	726	0.482471	6.039781	3.645983	Up	3.66E-14	1.48E-13
Chka	2540	289	3872.5	4.065981	50.86082	3.644879	Up	0	0
LOC100360982	2154	295.5	3971.5	4.914971	61.4595	3.644381	Up	3.74E-13	1.29E-12
Ccl2	780	10072.5	134255.5	460.5772	5743.974	3.640534	Up	0	0
Itih4	2992	8	105.5	0.094335	1.174986	3.638699	Up	5.24E-06	1.07E-05
Slc12a8	2452	4	53.5	0.058842	0.73085	3.634665	Up	4.59E-05	8.83E-05
RGD1565469	1118	4	53	0.127	1.577103	3.634376	Up	4.59E-05	8.81E-05
Apcdd1l	2285	1	13.5	0.015911	0.197449	3.633386	Up	0.001551	0.002598
Nt5c1b	1869	5	66.5	0.096035	1.191532	3.633112	Up	2.76E-05	5.40E-05
LOC100909469	1274	3	40	0.084712	1.049664	3.631217	Up	7.36E-05	0.000139
LOC100362123	3428	192	2537.5	1.99282	24.68483	3.630741	Up	0	0
Tssk4	1376	2	27	0.052844	0.654465	3.630505	Up	0.000116	0.000214
Grhl1	3427	27	359	0.28209	3.493278	3.630357	Up	3.64E-11	9.17E-11
Zswim2	2079	1	13	0.016936	0.209201	3.62673	Up	0.001551	0.002598
LOC100910996	1020	1	13	0.034519	0.426401	3.62673	Up	0.001551	0.002597
LOC100363289	576	11.5	150	0.704964	8.681982	3.622402	Up	8.84E-07	1.89E-06
RGD1564480	1671	17	225	0.365758	4.49984	3.620911	Up	2.14E-08	4.89E-08
LOC686545	3169	1	13	0.011111	0.136677	3.620746	Up	0.001551	0.002596
Nkpd1	2754	4	53	0.051973	0.638925	3.61982	Up	4.59E-05	8.83E-05
LOC100365826	543	2	26.5	0.131798	1.616943	3.616866	Up	0.000117	0.000216
RGD1562118	609	1	13	0.057816	0.708257	3.614738	Up	0.001551	0.002595
Hydin	15586	1	13	0.002259	0.027674	3.614738	Up	0.001551	0.002596
Tmprss7	2590	1	13	0.013595	0.166536	3.614738	Up	0.001551	0.002596
LOC100909569	2319	42.5	551	0.650725	7.95491	3.611725	Up	1.10E-13	4.23E-13

LOC100912483	729	9	116.5	0.436262	5.332392	3.611516	Up	2.92E-06	6.05E-06
Ccrn4l	2609	116.5	1527	1.597723	19.52137	3.610965	Up	9.68E-14	3.74E-13
Rrad	1471	8	104	0.193047	2.358007	3.610544	Up	5.24E-06	1.07E-05
Rwdd2a	1513	337	4401	7.940662	96.92667	3.609562	Up	0	0
Sorl1	10537	10	131.5	0.03396	0.414484	3.609424	Up	1.61E-06	3.40E-06
Eif2c4	3737	1	13	0.009422	0.114939	3.608705	Up	0.001551	0.002599
LOC100360244	378	4	53	0.384727	4.693125	3.608643	Up	4.59E-05	8.84E-05
Rtp3	1964	4	52.5	0.073462	0.895447	3.607534	Up	4.59E-05	8.82E-05
Ptchd3	2718	2	26	0.026331	0.320698	3.606405	Up	0.000117	0.000216
Olr1	3751	90	1168.5	0.8549	10.41075	3.606176	Up	0	0
Bbs7	2719	540.5	7025.5	7.094131	86.27642	3.604269	Up	7.21E-13	2.32E-12
Gcfc2	2583	290	3769	4.008595	48.71099	3.603079	Up	2.80E-13	9.94E-13
Tas2r139	960	3.5	46	0.130758	1.588534	3.602721	Up	7.36E-05	0.000139
Wwc1	4453	69	895	0.552536	6.705501	3.601205	Up	0	0
Fam212b	3374	205	2664.5	2.174144	26.37502	3.600652	Up	4.23E-13	1.44E-12
LOC291746	1959	1	13	0.017973	0.217421	3.596562	Up	0.001551	0.002598
Yod1	912	58.5	757.5	2.296249	27.72441	3.593805	Up	1.35E-14	5.59E-14
Kif9	2373	5	65	0.076122	0.918307	3.5926	Up	2.76E-05	5.39E-05
Myh7	5925	1	13.5	0.006136	0.074021	3.59252	Up	0.001551	0.002596
Vom2r60	2595	1	13	0.01401	0.16899	3.592379	Up	0.001551	0.002597
Tctex1d1	979	5	64	0.182168	2.191784	3.588762	Up	2.76E-05	5.41E-05
LOC680875	2680	1	13	0.013566	0.162958	3.586445	Up	0.001551	0.002595
Dlgap2	3718	2.5	32	0.023984	0.288079	3.586339	Up	0.000115	0.000213
Ttn	106973	938.5	11966	0.312276	3.739251	3.581856	Up	5.56E-13	1.84E-12
Sfxn2	2110	114	1458	1.927333	23.00913	3.577529	Up	7.79E-14	3.06E-13

Mip	2431	479.5	6080	7.020874	83.57356	3.573324	Up	0	0
LOC100362099	296	3	38.5	0.360731	4.292371	3.572781	Up	7.36E-05	0.000139
RGD1307315	1700	185	2359.5	3.895744	46.34767	3.572526	Up	0	0
LOC303590	2421	14	178.5	0.206925	2.459189	3.571003	Up	1.40E-07	3.11E-07
LOC684871	1318	54.5	691.5	1.474217	17.50678	3.569892	Up	3.66E-14	1.48E-13
Slc37a2	1521	20	255.5	0.470899	5.589138	3.569135	Up	3.18E-09	7.52E-09
LOC498836	372	1	12.5	0.09465	1.123078	3.568712	Up	0.002405	0.003964
Inca1	1287	1	13	0.028249	0.335143	3.568495	Up	0.001551	0.002598
Tmem72	3368	2	26	0.021589	0.256133	3.568495	Up	0.000117	0.000216
Olr1l	975	1.5	19	0.054757	0.648769	3.566587	Up	0.000596	0.001031
Scml4	4333	44	557	0.362307	4.292027	3.566376	Up	8.13E-14	3.18E-13
RGD1311186	2217	1038	13129.5	16.67586	197.4773	3.565853	Up	2.62E-14	1.07E-13
Btnl5	1944	2	25	0.036224	0.428894	3.565601	Up	0.000118	0.000218
RGD1563866	993	28	356	1.011303	11.95136	3.562887	Up	1.93E-11	4.98E-11
RGD1564613	729	21.5	269.5	1.047076	12.32839	3.557546	Up	1.68E-09	4.01E-09
Trmt13	1824	174.5	2190	3.406836	40.07726	3.556279	Up	1.43E-13	5.33E-13
Actrt3	1116	2	25	0.064128	0.753558	3.5547	Up	0.000118	0.000218
Rhoh	1556	4.5	56	0.102196	1.200425	3.55413	Up	4.59E-05	8.84E-05
Dsg4	3123	3	37	0.033823	0.396994	3.553038	Up	7.36E-05	0.000139
Asb16	1402	3	37	0.075342	0.884317	3.553038	Up	7.36E-05	0.000139
Acer2	5156	583.5	7302	4.031039	47.23662	3.550682	Up	5.08E-13	1.70E-12
Wdr78	2669	118.5	1490	1.589909	18.62677	3.550361	Up	0	0
Gabbr2	5459	11	136.5	0.071369	0.834906	3.548251	Up	8.84E-07	1.89E-06
Pklr	2922	34	427	0.416565	4.871515	3.547757	Up	5.99E-13	1.97E-12
Mapk13	1904	13.5	170	0.254769	2.975766	3.545998	Up	2.60E-07	5.69E-07

LOC498222	2866	2	25	0.024971	0.290918	3.542294	Up	0.000118	0.000218
Kbtbd10	2316	17	211	0.261667	3.047158	3.541659	Up	2.14E-08	4.90E-08
Dnahc3l	12261	19	237.5	0.055591	0.647045	3.540942	Up	6.02E-09	1.41E-08
Slc16a8	1829	3	37.5	0.058693	0.682316	3.539176	Up	7.36E-05	0.000139
Naaa	1830	13.5	170	0.266952	3.099048	3.537175	Up	2.60E-07	5.70E-07
RGD1304931	3861	5.5	68	0.05075	0.588708	3.53606	Up	2.76E-05	5.40E-05
Lvrn	2964	9	112	0.109234	1.265831	3.534592	Up	2.92E-06	6.06E-06
Mixl1	2342	32	396	0.487212	5.641024	3.533337	Up	1.74E-12	5.25E-12
RGD1359452	1846	78.5	973.5	1.521504	17.58798	3.531019	Up	0	0
Park2	1564	3	37.5	0.069005	0.79735	3.530449	Up	7.36E-05	0.000139
Trpc4	2875	1.5	18.5	0.018769	0.216872	3.5304	Up	0.000615	0.001063
RGD1310371	1416	1	12	0.024866	0.286753	3.52758	Up	0.002405	0.003968
Kcnj14	2751	106.5	1317.5	1.387264	15.96513	3.524611	Up	0	0
Ptgs2	1815	991	12208	19.53563	224.5495	3.522854	Up	2.91E-12	8.45E-12
Ifrd1	1736	2226	27397.5	45.82818	526.2494	3.52144	Up	0	0
Ebpl	1118	6.5	80.5	0.209324	2.403567	3.521365	Up	1.62E-05	3.21E-05
Dnahc6	12656	1	12	0.002782	0.031941	3.52117	Up	0.002405	0.003964
LOC497899	1127	15	185.5	0.479825	5.500536	3.518993	Up	7.51E-08	1.68E-07
Slc44a4	2254	2	24.5	0.031496	0.360704	3.517553	Up	0.000121	0.000223
Zfp498	1849	298	3655.5	5.766809	65.9865	3.516326	Up	5.55E-13	1.84E-12
Kctd16	1393	1	12.5	0.0261	0.298625	3.51624	Up	0.002405	0.003969
Areg	1154	198.5	2431	6.153846	70.28617	3.513681	Up	0	0
Plcg2	4321	28	343.5	0.232936	2.660037	3.513439	Up	1.93E-11	4.98E-11
Lama1	10570	1472	17885	4.958129	56.4926	3.510194	Up	3.92E-12	1.10E-11
RGD1559859	906	68	829	2.690148	30.51254	3.503645	Up	0	0

Nrn1l	620	2	24	0.11358	1.288038	3.503394	Up	0.000121	0.000223
LOC685289	578	1	12	0.060917	0.690037	3.501767	Up	0.002405	0.003968
Fam184b	2847	6	73	0.075413	0.853859	3.501122	Up	1.62E-05	3.21E-05
Lcp2	3407	29	354.5	0.307276	3.474839	3.49934	Up	1.04E-11	2.73E-11
Pex11b	1456	1575	18951.5	38.45537	434.5337	3.498211	Up	4.40E-12	1.22E-11
Omg	1855	1.5	18.5	0.028781	0.324963	3.497102	Up	0.000615	0.001063
Olr1105	918	1	12	0.038355	0.432507	3.495241	Up	0.002405	0.003967
Ppp1r14d	757	1	12	0.046512	0.524494	3.495241	Up	0.002405	0.003967
Ldlrad1	1776	12.5	149.5	0.249108	2.807139	3.494256	Up	4.81E-07	1.04E-06
Aldob	1589	21.5	259	0.482903	5.440204	3.493856	Up	1.68E-09	4.01E-09
Nlgn1	3322	1	12.5	0.010944	0.123054	3.49105	Up	0.002405	0.003967
Slc39a8	3097	10.5	127	0.121596	1.366492	3.490304	Up	1.61E-06	3.40E-06
LOC691995	1491	8.5	101.5	0.20188	2.26768	3.489645	Up	5.24E-06	1.07E-05
Zfp347	4884	507.5	6081.5	3.69977	41.55601	3.48955	Up	9.98E-13	3.15E-12
LOC100360692	867	2	23.5	0.081222	0.90962	3.485318	Up	0.000126	0.000231
Cd27	1602	1	12	0.022337	0.250088	3.484957	Up	0.002405	0.003964
Serpinb7	1143	5	60	0.157034	1.757313	3.484225	Up	2.76E-05	5.40E-05
Rab30	1336	295.5	3533.5	7.888662	88.26388	3.483971	Up	3.74E-13	1.29E-12
Nags	2111	2.5	30.5	0.043056	0.481737	3.483954	Up	0.000116	0.000213
C4a	5377	13	157	0.087047	0.973671	3.483576	Up	2.60E-07	5.70E-07
Serpinc1	1561	8	96	0.183386	2.050951	3.483336	Up	5.24E-06	1.07E-05
Ankrd61	1472	5	60	0.121936	1.363321	3.482931	Up	2.76E-05	5.39E-05
Trim71	2568	7	84	0.097763	1.091391	3.480732	Up	9.28E-06	1.87E-05
Dqx1	2606	66	786.5	0.901851	10.05985	3.479576	Up	0	0
Tigd3	1586	17.5	207.5	0.391399	4.365519	3.479441	Up	2.14E-08	4.90E-08

Nkx2-1	2297	35.5	421	0.549158	6.117261	3.477592	Up	3.77E-13	1.30E-12
Ppp1r15a	2033	2052.5	24530.5	36.17624	402.756	3.476792	Up	0	0
Dnah1	12872	24	287	0.066807	0.742939	3.475165	Up	2.47E-10	6.04E-10
Xkr4	3727	1	12	0.009755	0.108463	3.474927	Up	0.002405	0.003966
Baiap2l2	2079	1	12	0.017488	0.194441	3.474927	Up	0.002405	0.003966
LOC100911717	786	49.5	589.5	2.256807	25.02694	3.471127	Up	3.91E-14	1.57E-13
Tspan33	1983	4	47	0.071602	0.793586	3.470321	Up	4.59E-05	8.83E-05
Apoc3	536	1	12	0.06783	0.750824	3.468488	Up	0.002405	0.003966
Syt4	3877	1	12	0.009378	0.103802	3.468488	Up	0.002405	0.003965
Krt5	2088	3	36	0.052237	0.577359	3.466336	Up	7.36E-05	0.000139
Tsga13	1134	5.5	65	0.172793	1.909738	3.466255	Up	2.76E-05	5.40E-05
RGD1307443	4098	5	59	0.043799	0.483973	3.465947	Up	2.76E-05	5.41E-05
Lin28a	3520	25.5	300.5	0.257678	2.846758	3.465681	Up	1.30E-10	3.22E-10
Micb	1202	12	141.5	0.356283	3.935178	3.465334	Up	4.81E-07	1.04E-06
Art4	2436	3	35	0.043362	0.478587	3.464284	Up	7.36E-05	0.000139
Acap1	2468	94	1110	1.363359	15.03892	3.463464	Up	0	0
Ret	3928	1	12	0.009256	0.101996	3.462021	Up	0.002405	0.003965
Ttc39a	2647	5	59	0.067809	0.747232	3.462014	Up	2.76E-05	5.39E-05
LOC690286	1376	13.5	159.5	0.350863	3.855993	3.458124	Up	2.60E-07	5.70E-07
LOC100911221	1524	162.5	1904.5	3.795339	41.68978	3.457393	Up	0	0
Cyp4f17	2156	629	7358.5	10.37598	113.9539	3.457131	Up	1.00E-12	3.16E-12
Spata4	1086	1	11.5	0.032422	0.355615	3.455293	Up	0.003992	0.006452
LOC691257	3291	118.5	1393.5	1.286976	14.10312	3.453957	Up	0	0
Cadps2	4949	4	47	0.02869	0.314342	3.453724	Up	4.59E-05	8.85E-05
Shd	1459	11.5	135	0.281065	3.076915	3.45251	Up	8.84E-07	1.89E-06

Cxcl3	674	312.5	3671.5	16.59302	181.6159	3.452242	Up	1.77E-13	6.50E-13
Fam83h	4523	9	106	0.071456	0.780456	3.449188	Up	2.92E-06	6.07E-06
Accsl	2167	1	11.5	0.016248	0.177387	3.448553	Up	0.003992	0.006457
Fibcd1	4695	2	23	0.015243	0.166048	3.445373	Up	0.000126	0.000231
Cd99l2	4174	1	11.5	0.008436	0.091878	3.445171	Up	0.003992	0.006459
Fam59b	2662	10	117	0.134422	1.463019	3.444103	Up	1.61E-06	3.40E-06
Pld5	1720	5	58	0.104354	1.134207	3.44212	Up	2.76E-05	5.40E-05
Igfals	2190	3.5	40.5	0.057057	0.620099	3.442025	Up	7.36E-05	0.000139
LOC100909560	886	4.5	52	0.178831	1.943243	3.4418	Up	4.59E-05	8.83E-05
LOC100912741	551	63	729	4.072635	44.22139	3.44071	Up	0	0
Cygb	2138	2	23	0.033205	0.360429	3.440227	Up	0.000126	0.000231
LOC100909666	4718	9	104	0.068138	0.737861	3.436815	Up	2.92E-06	6.07E-06
Hpx	1484	7.5	86	0.179106	1.939061	3.43647	Up	9.28E-06	1.87E-05
Pde5a	3397	2	24	0.021405	0.231111	3.432553	Up	0.000121	0.000223
LOC501230	300	5	58.5	0.602122	6.493928	3.430965	Up	2.76E-05	5.41E-05
Cplx3	3391	29.5	341	0.310366	3.347302	3.430956	Up	1.04E-11	2.73E-11
LOC687399	1485	77.5	897.5	1.866896	20.1258	3.430333	Up	0	0
Serpinf2	2197	6	69	0.097724	1.053478	3.430305	Up	1.62E-05	3.20E-05
LOC100910689	1068	53	610.5	1.767706	19.0558	3.43028	Up	4.04E-14	1.62E-13
RGD1562299	927	1	12	0.03922	0.422483	3.429241	Up	0.002405	0.003965
Ptgir	1360	4	46	0.105245	1.130583	3.425247	Up	4.59E-05	8.80E-05
LOC100912582	3480	35	400	0.357747	3.838082	3.423375	Up	3.77E-13	1.30E-12
Sarm1	4039	2	23.5	0.018003	0.192582	3.419181	Up	0.000126	0.000231
Zc3h6	5324	106	1213.5	0.712008	7.597942	3.415644	Up	0	0
LOC100909559	798	10	113.5	0.445537	4.749189	3.414064	Up	1.61E-06	3.40E-06

Rp1	6991	2	23	0.010237	0.10894	3.411671	Up	0.000126	0.000231
Micalcl	2352	36.5	418	0.559089	5.937367	3.408675	Up	2.57E-13	9.18E-13
LOC100910258	1764	1	11	0.01996	0.211766	3.407273	Up	0.003992	0.006458
Sec14l3	1595	870	9887	19.46096	206.4254	3.406965	Up	0	0
Tspyl3	2100	1	11.5	0.017313	0.183475	3.405684	Up	0.003992	0.006457
Adam4l1	2464	8.5	96	0.122859	1.297497	3.400659	Up	5.24E-06	1.07E-05
Hspa1l	2632	39	440.5	0.530005	5.590631	3.398934	Up	1.32E-13	4.99E-13
Fas	2554	397	4481	5.550782	58.54201	3.398709	Up	6.21E-13	2.04E-12
Gck	2220	1	11	0.01586	0.167052	3.396807	Up	0.003992	0.006453
Myom3	4752	373.5	4202	2.802673	29.51407	3.396528	Up	3.81E-13	1.31E-12
LOC100910088	3102	3	34	0.034422	0.36246	3.39643	Up	7.36E-05	0.000139
Dnhd1-ps1	14585	94	1055.5	0.229443	2.411198	3.393545	Up	0	0
RGD1564567	1956	2	22	0.036002	0.378277	3.393301	Up	0.000135	0.000247
Tulp2	1605	2	23	0.045304	0.475636	3.392141	Up	0.000126	0.000231
Zc3h8	1561	132.5	1479.5	3.025395	31.63359	3.386262	Up	1.14E-13	4.37E-13
Nat8b	916	10	111.5	0.389395	4.06155	3.382725	Up	1.61E-06	3.40E-06
LOC100362345	1375	220.5	2469	5.743959	59.8249	3.380629	Up	3.61E-13	1.25E-12
LOC688297	862	5	55.5	0.206894	2.153269	3.379564	Up	2.76E-05	5.41E-05
Kcnh3	3715	1	11	0.009478	0.098615	3.379193	Up	0.003992	0.006454
LOC690995	1083	1	11	0.032511	0.338278	3.379193	Up	0.003992	0.006451
LOC100362120	708	1	11	0.049731	0.51745	3.379193	Up	0.003992	0.006455
P2ry6	2530	18	200	0.254358	2.645321	3.378513	Up	1.14E-08	2.63E-08
Cnr2	3286	444.5	4908.5	4.813467	49.93123	3.374794	Up	1.07E-12	3.36E-12
Pramef12	1672	1	11	0.021058	0.218035	3.372087	Up	0.003992	0.006458
RGD1562146	666	3	33.5	0.162047	1.67734	3.371693	Up	7.37E-05	0.000139

Dnai2	2923	29.5	327.5	0.36202	3.744896	3.370783	Up	1.04E-11	2.73E-11
B3gnt4	1414	3	33.5	0.076325	0.789398	3.37053	Up	7.37E-05	0.000139
Rasl11a	810	10	110.5	0.441768	4.567399	3.370011	Up	1.61E-06	3.40E-06
Dusp10	2704	930	10325	12.31622	127.3333	3.369978	Up	0	0
Rel	2087	157	1731.5	2.686666	27.65549	3.363677	Up	0	0
LOC685248	825	2	22	0.086052	0.88486	3.362166	Up	0.000135	0.000247
Ankrd33b	1791	5	55.5	0.100218	1.030328	3.361897	Up	2.76E-05	5.41E-05
Shpk	2794	40	439	0.511466	5.250326	3.359697	Up	1.16E-13	4.43E-13
Anxa9	1575	1	11	0.022355	0.229178	3.357769	Up	0.003992	0.006451
Map3k8	2720	50	548.5	0.657358	6.733756	3.356659	Up	3.62E-14	1.46E-13
Ccdc64b	1966	4	43	0.072221	0.739386	3.355842	Up	4.59E-05	8.80E-05
Il1a	1992	177.5	1947	3.192114	32.64072	3.354089	Up	0	0
Dynlrb2	533	1	11	0.068211	0.697477	3.354061	Up	0.003992	0.006459
Ly6g5c	686	1	11	0.052162	0.532733	3.352338	Up	0.003992	0.006453
Adcy5	4847	3	33	0.022266	0.227123	3.350561	Up	7.37E-05	0.000139
Chrnb1	2283	19	208.5	0.298555	3.043408	3.349618	Up	6.02E-09	1.41E-08
Coq10b	1651	872.5	9484	18.83369	191.6617	3.347174	Up	4.97E-13	1.67E-12
LOC100912991	663	92.5	1009	4.998004	50.79546	3.345276	Up	6.66E-15	2.78E-14
Kcp	4769	2	22	0.015247	0.154772	3.343544	Up	0.000135	0.000247
Flcn	2987	473	5141.5	5.662154	57.42552	3.342269	Up	5.88E-13	1.94E-12
Kcng4	4083	645.5	6973	5.629115	57.05943	3.341485	Up	5.31E-13	1.77E-12
Arid3b	3772	50	540.5	0.474327	4.782296	3.333749	Up	3.62E-14	1.46E-13
RGD1565456	381	2	22	0.190849	1.923123	3.33295	Up	0.000135	0.000247
Bfsp1	2132	15	161.5	0.252027	2.537721	3.331882	Up	7.51E-08	1.68E-07
Lrrc25	3748	38.5	414	0.366423	3.683308	3.32942	Up	1.45E-13	5.43E-13

LOC100366121	979	2.5	27.5	0.092841	0.932775	3.328691	Up	0.000116	0.000214
LOC100362678	3947	1050.5	11303	9.520202	95.57837	3.32762	Up	0	0
LOC100912305	482	3	32	0.221528	2.224021	3.327613	Up	7.38E-05	0.000139
Dnah7	12427	1	11	0.002926	0.029336	3.325844	Up	0.003992	0.006456
Atp1b4	4174	1	11	0.00871	0.087339	3.325844	Up	0.003992	0.006455
Dnd1	1517	13	141	0.30778	3.085985	3.325759	Up	2.60E-07	5.70E-07
Loxhd1	7038	3	32	0.015171	0.152057	3.325188	Up	7.38E-05	0.000139
LOC100359977	854	5	53.5	0.208832	2.09209	3.324528	Up	2.76E-05	5.40E-05
LOC100912930	562	2.5	26.5	0.156627	1.568684	3.324148	Up	0.000117	0.000216
Dab1	1668	1	11	0.021797	0.217479	3.318703	Up	0.003992	0.006456
Rsph10b	2901	3	32	0.037202	0.370761	3.317036	Up	7.38E-05	0.000139
Tmem86a	1754	17	182.5	0.34845	3.472545	3.316968	Up	2.14E-08	4.90E-08
Mb	1015	3	31.5	0.104068	1.035699	3.315001	Up	7.40E-05	0.000139
LOC685385	291	10	105	1.2139	12.04679	3.310927	Up	1.61E-06	3.40E-06
Egf	4801	11.5	122	0.085773	0.850467	3.309668	Up	8.84E-07	1.89E-06
Catsperg1	3829	1.5	16	0.014093	0.139511	3.307346	Up	0.00072	0.001238
RGD1566248	1167	2	21.5	0.061325	0.606226	3.305305	Up	0.000151	0.000276
LOC691228	3312	134	1413	1.441692	14.23459	3.303566	Up	0	0
Nid1	4982	5	52	0.035567	0.351007	3.302878	Up	2.76E-05	5.40E-05
Trim55	2709	15	157	0.196442	1.938586	3.302831	Up	7.51E-08	1.68E-07
Gsg1	1374	8.5	90	0.221992	2.188219	3.301175	Up	5.24E-06	1.07E-05
Gltpd2	1375	274.5	2896.5	7.127578	70.24083	3.300826	Up	1.86E-13	6.82E-13
Scel	2886	7	74	0.086594	0.852953	3.300134	Up	9.28E-06	1.87E-05
Nrxn3	6007	7	75	0.041794	0.411305	3.298844	Up	9.28E-06	1.86E-05
Cyp1a1	2615	28.5	299	0.388564	3.81746	3.296389	Up	1.93E-11	4.98E-11

LOC100362255	19203	17	178.5	0.031529	0.309431	3.294873	Up	2.14E-08	4.90E-08
Cpxm1	2367	7	74.5	0.107034	1.050261	3.294605	Up	9.28E-06	1.87E-05
Nptx1	4949	3	31	0.021344	0.209131	3.292535	Up	7.40E-05	0.000139
LOC691170	4221	471.5	4944	3.995003	39.10104	3.290939	Up	2.91E-13	1.03E-12
Hps5	4641	486	5082.5	3.740629	36.55719	3.288802	Up	1.59E-13	5.89E-13
Cybrd1	3427	98.5	1030.5	1.027908	10.03982	3.28795	Up	0	0
LOC100909701	822	4	41.5	0.172732	1.686135	3.287109	Up	4.59E-05	8.80E-05
Dyx1c1	2012	13	136	0.231489	2.258552	3.286384	Up	2.60E-07	5.69E-07
Dntt	1851	5.5	58	0.10679	1.04178	3.286205	Up	2.76E-05	5.41E-05
RGD1308160	2592	1.5	15.5	0.020597	0.200519	3.283215	Up	0.000849	0.001453
Igflr1	1219	7	73	0.205012	1.994206	3.282037	Up	9.28E-06	1.87E-05
Gstt2	1381	427	4438	11.02958	107.173	3.280493	Up	1.02E-12	3.21E-12
LOC688286	1176	3	31	0.090796	0.877799	3.273187	Up	7.40E-05	0.000139
Ttc18	3604	21	218	0.208981	2.019513	3.272562	Up	1.68E-09	4.01E-09
LOC100910685	393	227.5	2346.5	20.63615	199.3748	3.272238	Up	3.55E-15	1.49E-14
Rapgef4	4189	51	530	0.436884	4.220508	3.272096	Up	8.44E-15	3.52E-14
LOC687813	2050	189	1956	3.297641	31.82517	3.270662	Up	2.87E-13	1.02E-12
LOC684792	737	3	31	0.146436	1.412878	3.270298	Up	7.40E-05	0.000139
Gdap1l1	1219	10	102.5	0.290723	2.795896	3.265592	Up	1.61E-06	3.40E-06
Ankrd13d	2123	3	31	0.050835	0.488785	3.265301	Up	7.40E-05	0.000139
Fbxo48	1016	28	287.5	0.980508	9.422301	3.264478	Up	1.93E-11	4.98E-11
Rsph9	890	2	21	0.0817	0.784745	3.263809	Up	0.000151	0.000276
Kpna5	1664	144	1479.5	3.092491	29.67388	3.262352	Up	1.05E-13	4.06E-13
Baalc	2369	4	41	0.060419	0.579721	3.262282	Up	4.59E-05	8.80E-05
RGD1563049	486	14	144	1.033153	9.892389	3.259266	Up	1.40E-07	3.10E-07

LOC679979	1585	3	31	0.06809	0.651288	3.257774	Up	7.40E-05	0.000139
Tmprss11b	1263	21	214	0.595425	5.682644	3.254571	Up	1.68E-09	4.01E-09
Actn3	2904	2	21	0.025039	0.238954	3.254483	Up	0.000151	0.000276
LOC363337	1654	22	223	0.474569	4.522588	3.252458	Up	8.87E-10	2.13E-09
Zfp563	6581	67	678.5	0.361427	3.437456	3.249564	Up	0	0
Cxcl10	1133	915.5	9321	28.88792	274.5731	3.248652	Up	0	0
Dlg2	3002	11	112	0.130927	1.243812	3.247936	Up	8.84E-07	1.88E-06
LOC690130	1971	36	367.5	0.655321	6.221209	3.246922	Up	2.57E-13	9.17E-13
Hnf1b	2328	5	51	0.0771	0.731799	3.246638	Up	2.76E-05	5.41E-05
Pou5f2	1234	15	152.5	0.434037	4.116887	3.245664	Up	7.51E-08	1.68E-07
Plcxd2	7364	249	2522	1.205505	11.43177	3.245339	Up	1.13E-14	4.70E-14
LOC100362347	6454	1218.5	12337.5	6.728291	63.77213	3.244614	Up	4.65E-12	1.29E-11
LOC100912205	1635	76	764	1.650692	15.6191	3.242168	Up	0	0
Zcwpw2	1371	14	142.5	0.367074	3.470511	3.241004	Up	1.40E-07	3.10E-07
Bcl6	3389	412	4183.5	4.358959	41.1938	3.240372	Up	2.14E-13	7.77E-13
Chd5	6628	5	51	0.027254	0.257035	3.237449	Up	2.76E-05	5.40E-05
Zfp654	4999	511.5	5135.5	3.63939	34.30701	3.236735	Up	0	0
Pcf11	6053	1321.5	13323	7.791644	73.4274	3.236319	Up	2.69E-12	7.87E-12
Rab25	1082	3	30	0.099744	0.939006	3.234831	Up	7.43E-05	0.00014
LOC100910013	1108	1.5	15.5	0.049219	0.462586	3.232425	Up	0.000849	0.001452
Psd2	4397	2	20.5	0.016537	0.155351	3.231762	Up	0.000181	0.00033
Cct6b	1803	4	40	0.07875	0.738698	3.229635	Up	4.59E-05	8.80E-05
Cox11	2506	225.5	2261.5	3.210424	30.10121	3.228986	Up	0	0
Ccnt2	5652	789.5	7905.5	4.979462	46.68607	3.228931	Up	0	0
Sytl3	2227	10	100	0.160679	1.506458	3.228906	Up	1.61E-06	3.40E-06

Cldn20	660	2	20	0.108434	1.015813	3.227745	Up	0.000181	0.00033
LOC688019	867	75	750.5	3.086176	28.89176	3.226766	Up	0	0
LOC100911406	1248	6	60.5	0.172954	1.618151	3.225888	Up	1.62E-05	3.21E-05
LOC100910867	3241	96	959.5	1.056378	9.872939	3.224353	Up	0	0
LOC100912277	2695	53.5	533.5	0.704502	6.583678	3.224218	Up	4.04E-14	1.62E-13
Akap14	1879	2	20	0.037477	0.34962	3.221704	Up	0.000181	0.00033
Smox	2282	84	835	1.311143	12.23067	3.221606	Up	0	0
Atf3	1928	177.5	1771	3.287964	30.6508	3.220658	Up	0	0
Csf3	1305	175.5	1755	4.812448	44.83881	3.219905	Up	3.75E-13	1.30E-12
LOC689291	952	3	30.5	0.114569	1.066332	3.218363	Up	7.43E-05	0.00014
Acrbp	1284	21	209.5	0.5839	5.430986	3.217421	Up	1.68E-09	4.01E-09
LOC500354	1494	55.5	550.5	1.324499	12.31874	3.217336	Up	0	0
Zbtb26	2219	84.5	841.5	1.359403	12.63414	3.216282	Up	0	0
Dner	3619	12.5	124	0.123199	1.144947	3.21622	Up	4.81E-07	1.04E-06
RGD1562839	483	4	40.5	0.293967	2.7315	3.215967	Up	4.59E-05	8.80E-05
Lhx9	1298	6.5	65	0.179413	1.667059	3.215951	Up	1.62E-05	3.20E-05
Lrrc23	1548	7.5	73.5	0.171331	1.589006	3.213266	Up	9.28E-06	1.87E-05
LOC100359562	2163	3	30.5	0.050425	0.46766	3.213238	Up	7.43E-05	0.00014
LOC100910758	739	11	108	0.527201	4.889007	3.213116	Up	8.84E-07	1.89E-06
Ctbs	1616	10.5	103	0.230906	2.135236	3.209021	Up	1.61E-06	3.40E-06
Cacna2d3	3276	24	239	0.263724	2.437377	3.208231	Up	2.47E-10	6.04E-10
LOC100912128	1370	9	89	0.234654	2.166953	3.207063	Up	2.92E-06	6.06E-06
Ier5	2114	2426.5	23985.5	41.06811	377.9811	3.202224	Up	0	0
Mos	1299	2.5	24	0.067763	0.622389	3.199242	Up	0.000121	0.000223
LOC100911303	1182	2	20	0.061517	0.564921	3.198986	Up	0.000181	0.00033

Enpp2	3147	8	78	0.090236	0.828081	3.198	Up	5.24E-06	1.07E-05
LOC691422	2721	152	1490	1.992805	18.26419	3.196145	Up	1.08E-13	4.15E-13
Cfhr1	1166	2	20	0.062361	0.571129	3.195092	Up	0.000181	0.00033
Klhl35	1965	8	78.5	0.145099	1.328048	3.194201	Up	5.24E-06	1.07E-05
Cfb	2573	4	39.5	0.056075	0.51307	3.193738	Up	4.59E-05	8.80E-05
LOC691519	3192	6	58	0.066543	0.608344	3.192527	Up	1.62E-05	3.21E-05
LOC100911332	1803	5	49	0.098914	0.903857	3.191841	Up	2.76E-05	5.39E-05
Rapsn	1430	29	284.5	0.726075	6.62755	3.190285	Up	1.04E-11	2.73E-11
Slc4a1	4508	3.5	33.5	0.027337	0.248205	3.182616	Up	7.37E-05	0.000139
Ccdc151	2201	11	108	0.180138	1.634973	3.182096	Up	8.84E-07	1.89E-06
Cd247	1660	24	232.5	0.514239	4.667219	3.182052	Up	2.47E-10	6.04E-10
Irf1	2078	1403	13620	24.15115	218.6488	3.178451	Up	1.34E-12	4.13E-12
Vwf	8822	4	39	0.016485	0.149126	3.177342	Up	4.59E-05	8.80E-05
Tceanc	1077	30.5	294	1.005107	9.090549	3.177018	Up	5.60E-12	1.52E-11
RGD1565014	2644	5	48	0.067018	0.605434	3.175346	Up	2.76E-05	5.39E-05
LOC100360914	3911	369	3566.5	3.373629	30.45595	3.174351	Up	0	0
Best1	2035	2	19.5	0.035168	0.316606	3.17036	Up	0.000237	0.000428
Cldn15	684	11	105.5	0.572946	5.155495	3.169639	Up	8.84E-07	1.89E-06
Rsrc2	2095	2614	25193.5	44.58222	401.1423	3.169574	Up	0	0
Pkib	1520	2	19	0.046329	0.416743	3.169181	Up	0.000237	0.000427
Spire2	2304	1.5	14.5	0.023421	0.209921	3.163985	Up	0.001094	0.001849
Mir3085	89	5.5	52	2.188773	19.60803	3.163251	Up	2.76E-05	5.40E-05
Lrp2	15438	7.5	71	0.017328	0.154772	3.15894	Up	9.28E-06	1.87E-05
Matk	1670	3	29	0.064625	0.577076	3.158606	Up	7.50E-05	0.000141
Zbtb24	3769	442.5	4228	4.191779	37.41622	3.158029	Up	0	0

Tmem25	2539	23.5	223.5	0.330857	2.949745	3.156311	Up	4.68E-10	1.14E-09
Aen	2402	1493.5	14263	22.23152	198.0314	3.155051	Up	5.68E-12	1.54E-11
Phex	4245	28	266	0.234405	2.087842	3.154938	Up	1.93E-11	4.97E-11
Cep95	3016	272.5	2598.5	3.232972	28.77781	3.154024	Up	7.17E-13	2.31E-12
Rwdd3	1044	37.5	355.5	1.277902	11.37353	3.15383	Up	1.86E-13	6.81E-13
RGD1561560	1575	6	57.5	0.136681	1.216312	3.153627	Up	1.62E-05	3.20E-05
LOC100361645	1755	225	2131	4.555573	40.51823	3.152867	Up	0	0
Unc13d	4018	42	399.5	0.373184	3.318786	3.152695	Up	1.10E-13	4.23E-13
Sncaip	3803	42	401	0.396695	3.514728	3.147312	Up	1.10E-13	4.23E-13
Sstr1	3615	1.5	14.5	0.015086	0.133543	3.146048	Up	0.001094	0.001849
LOC100911486	4891	5	47.5	0.036698	0.324703	3.145351	Up	2.76E-05	5.39E-05
RGD1304731	3430	17	160.5	0.175847	1.55584	3.145304	Up	2.14E-08	4.90E-08
Asah2	2456	23	219	0.335804	2.967553	3.143583	Up	4.68E-10	1.13E-09
Tslp	591	28	263	1.683671	14.87112	3.142832	Up	1.93E-11	4.98E-11
Nrsn2	1402	2	19	0.051455	0.453744	3.140496	Up	0.000237	0.000428
St7l	2538	351.5	3312.5	4.941673	43.55905	3.139901	Up	9.48E-14	3.68E-13
LOC685294	2855	55.5	523.5	0.694305	6.119841	3.139852	Up	0	0
Dchs1	10462	2	19	0.00695	0.061236	3.139244	Up	0.000237	0.000427
Socs2	918	4	37	0.15342	1.350558	3.138001	Up	4.60E-05	8.81E-05
LOC682419	2598	5	47	0.068646	0.602956	3.134802	Up	2.76E-05	5.39E-05
Alppl2	1410	2	18.5	0.049943	0.438373	3.133806	Up	0.000337	0.000602
Parp16	2336	2	19	0.030636	0.268857	3.133522	Up	0.000237	0.000428
Alkbh1	2931	552	5177	6.714268	58.91833	3.133415	Up	0	0
Tas1r3	2577	15.5	145.5	0.215561	1.881036	3.125361	Up	7.51E-08	1.68E-07
Ccdc146	3391	3	28	0.031488	0.274618	3.124547	Up	7.61E-05	0.000143

Mme	3493	17	160.5	0.17563	1.530871	3.123741	Up	2.14E-08	4.89E-08
LOC100912031	772	60	558	2.764739	24.07246	3.122168	Up	0	0
Ppp1r3c	2783	19	175	0.242031	2.103626	3.119612	Up	6.02E-09	1.41E-08
Nfkbib	1491	546	5103	13.15372	114.1015	3.116776	Up	1.34E-13	5.05E-13
Hs3st5	3043	179.5	1669	2.110493	18.29871	3.11609	Up	0	0
Cd72	1495	2	19	0.048638	0.421304	3.114715	Up	0.000237	0.000428
Card9	1879	6	56	0.114873	0.993114	3.11192	Up	1.62E-05	3.20E-05
Vstm5	1894	15.5	144	0.294506	2.545988	3.111858	Up	7.51E-08	1.69E-07
Ccdc15	2601	113	1045.5	1.555699	13.43766	3.110648	Up	2.08E-13	7.57E-13
Fmo3	1916	14.5	134	0.270952	2.337804	3.109046	Up	1.40E-07	3.11E-07
Abhd10	1251	355	3280	10.13781	87.46358	3.108936	Up	5.33E-13	1.78E-12
Ccr1	1870	7.5	69.5	0.143669	1.237237	3.106302	Up	9.28E-06	1.87E-05
LOC100909675	1387	817	7521.5	21.03066	180.9438	3.104975	Up	1.28E-12	3.95E-12
LOC498122	1425	11	101	0.274209	2.358784	3.104694	Up	8.84E-07	1.89E-06
LOC100909946	706	17	156	0.85595	7.361958	3.104491	Up	2.14E-08	4.89E-08
Lyl1	1484	5	46	0.12095	1.039753	3.10376	Up	2.76E-05	5.39E-05
LOC100911932	2519	7	64.5	0.09921	0.85256	3.103249	Up	9.28E-06	1.87E-05
Enpp3	2799	23.5	216	0.298894	2.568435	3.103182	Up	4.68E-10	1.13E-09
Oasl	1980	13.5	124	0.243253	2.088164	3.101708	Up	2.60E-07	5.70E-07
RGD1311659	1839	336.5	3090.5	6.531241	56.05649	3.101452	Up	2.61E-13	9.32E-13
Zfp105	2025	231.5	2128	4.0895	35.07967	3.100639	Up	0	0
LOC100909483	3372	25.5	233	0.269497	2.309113	3.098995	Up	1.30E-10	3.22E-10
Zfp473	2932	9	82.5	0.110035	0.942347	3.098297	Up	2.92E-06	6.07E-06
Rnd3	2004	2255.5	20706	40.27328	344.8658	3.09814	Up	0	0
LOC100362607	691	14	128	0.723326	6.184532	3.095946	Up	1.40E-07	3.11E-07

Il1r2	1380	23	210.5	0.596387	5.098894	3.095863	Up	4.68E-10	1.14E-09
Efcab2	2604	188.5	1717	2.58094	21.99733	3.09136	Up	1.80E-13	6.62E-13
Ccl9	1313	15.5	141	0.422204	3.598356	3.091327	Up	7.51E-08	1.68E-07
Neu2	1693	1.5	14	0.032212	0.274492	3.091093	Up	0.001094	0.001849
Zfp438	3117	55	503	0.632873	5.391776	3.090773	Up	0	0
Cpn1	1780	2	18.5	0.040206	0.342194	3.089337	Up	0.000337	0.000602
Scgb2a2	1118	8	73	0.256052	2.175167	3.086619	Up	5.24E-06	1.07E-05
Pbx4	1384	17.5	159	0.451426	3.832372	3.085678	Up	2.14E-08	4.90E-08
Spata25	1608	4	35.5	0.087587	0.743521	3.085593	Up	4.61E-05	8.83E-05
Phf7	1574	82	742.5	1.858719	15.74521	3.082532	Up	0	0
LOC100912373	897	2	18	0.078506	0.664953	3.082382	Up	0.000337	0.000602
Egr2	2976	354	3207	4.257822	36.0116	3.080274	Up	0	0
Rex2	1251	3.5	32	0.100342	0.847544	3.078361	Up	7.38E-05	0.000139
LOC100912840	1449	14.5	130	0.354715	2.987912	3.074404	Up	1.40E-07	3.11E-07
LOC686785	1240	15	135.5	0.431474	3.634163	3.074277	Up	7.51E-08	1.68E-07
Zfp788	4947	78	699	0.561533	4.718027	3.070741	Up	0	0
LOC684623	1332	1.5	13.5	0.040081	0.336691	3.070427	Up	0.001551	0.002595
LOC100910286	1051	264	2371	8.957813	75.24008	3.070283	Up	6.88E-14	2.71E-13
LOC100910931	1432	284.5	2545	7.07373	59.39402	3.069775	Up	1.18E-13	4.49E-13
Wdr27	2759	20	179.5	0.258977	2.170998	3.067462	Up	3.18E-09	7.52E-09
Stard13	5529	982.5	8829	6.358815	53.30471	3.067433	Up	0	0
Adam30	3441	24.5	218.5	0.252694	2.116498	3.066213	Up	2.47E-10	6.04E-10
Sh2d4b	1296	6	53.5	0.164336	1.373722	3.063372	Up	1.62E-05	3.20E-05
Zbtb43	5108	150	1337.5	1.045748	8.735534	3.062361	Up	7.11E-15	2.97E-14
Asgr1	1214	14	124.5	0.410767	3.421341	3.058169	Up	1.40E-07	3.11E-07

Pdk4	1435	53	472	1.320412	10.99664	3.058003	Up	4.04E-14	1.62E-13
Ddn	3582	8	71.5	0.079918	0.665049	3.056871	Up	5.24E-06	1.07E-05
RGD1565622	2133	27	239.5	0.452683	3.762069	3.054952	Up	3.64E-11	9.17E-11
Gpr37l1	2302	41.5	371	0.646214	5.36903	3.054577	Up	1.47E-13	5.48E-13
Slc17a2	2822	5	44.5	0.063197	0.524721	3.053616	Up	2.76E-05	5.39E-05
Zfp59	2004	45.5	403.5	0.81087	6.730653	3.053204	Up	2.49E-14	1.02E-13
Atg9b	2775	53	471.5	0.684461	5.67648	3.051957	Up	4.04E-14	1.62E-13
LOC499746	1431	2	18	0.050813	0.421218	3.051298	Up	0.000337	0.000602
Pus3	1718	297	2632	6.169353	51.13282	3.051059	Up	3.95E-13	1.35E-12
LOC100912465	758	211.5	1856	9.859169	81.66256	3.050137	Up	2.52E-13	9.04E-13
Alox12	2839	2	17.5	0.024804	0.205326	3.049247	Up	0.000518	0.000915
LOC100910206	739	5	44	0.24133	1.995156	3.047423	Up	2.76E-05	5.39E-05
LOC499136	702	74	653.5	3.755685	31.03209	3.046613	Up	9.99E-15	4.15E-14
Styxl1	1306	34.5	304	0.941537	7.769442	3.044721	Up	5.99E-13	1.97E-12
Ccdc148	2340	2.5	22	0.038107	0.314278	3.043896	Up	0.000135	0.000247
RGD1561662	4502	112	988	0.888171	7.320606	3.043053	Up	0	0
Rbm12b	3427	159	1395	1.651675	13.60493	3.042128	Up	8.53E-14	3.33E-13
Mesp1	1275	1.5	13	0.041423	0.34112	3.041767	Up	0.001551	0.002598
Cwf19l2	6346	552.5	4849	3.099527	25.51239	3.041078	Up	0	0
Giot1	2557	102	894	1.418889	11.67861	3.041035	Up	0	0
Slc19a2	3479	267.5	2349	2.738925	22.53792	3.040673	Up	7.08E-13	2.29E-12
Elavl2	4159	96.5	845.5	0.825235	6.785068	3.039487	Up	0	0
Tifa	1662	135	1186.5	2.900715	23.83887	3.038835	Up	2.15E-13	7.77E-13
Hspb3	741	1.5	13	0.071275	0.585734	3.038779	Up	0.001551	0.002597
Neb	20076	30	264.5	0.053472	0.439398	3.038683	Up	5.60E-12	1.52E-11

Zc3h12d	3691	11	97.5	0.10773	0.883518	3.035846	Up	8.84E-07	1.89E-06
L1td1	2887	1.5	13	0.018294	0.150027	3.035784	Up	0.001551	0.002596
Foxh1	1721	4	36	0.084501	0.692112	3.033961	Up	4.60E-05	8.81E-05
Bucs1	2142	2.5	22	0.041898	0.342488	3.031113	Up	0.000135	0.000247
Slc35f2	2455	2	17.5	0.029151	0.238175	3.030388	Up	0.000518	0.000914
Dnah17	14065	24.5	213.5	0.06227	0.507212	3.025977	Up	2.47E-10	6.04E-10
Mpp7	1892	17	147	0.319398	2.601143	3.025719	Up	2.14E-08	4.90E-08
Synpo2l	3440	4	35	0.041942	0.341523	3.025521	Up	4.61E-05	8.83E-05
Efcab3	1791	2	17	0.039319	0.31992	3.024426	Up	0.000518	0.000914
Sfrp5	1989	32.5	284	0.586567	4.767599	3.022894	Up	1.74E-12	5.25E-12
Trim10	2366	4	34	0.059526	0.481298	3.015333	Up	4.63E-05	8.85E-05
Myl4	795	50	430.5	2.24619	18.11494	3.011628	Up	3.62E-14	1.46E-13
Ptpn22	2747	9.5	82.5	0.124063	1.000241	3.011203	Up	2.92E-06	6.07E-06
RGD1560020_predicted	3287	15	129.5	0.162422	1.306188	3.007543	Up	7.51E-08	1.68E-07
Nrgn	1384	10	85.5	0.255235	2.049871	3.005637	Up	1.61E-06	3.40E-06
Krt34	1176	4.5	38.5	0.137169	1.100291	3.003854	Up	4.60E-05	8.80E-05
Rnf165	6832	4.5	39	0.023779	0.190718	3.003681	Up	4.59E-05	8.80E-05
Ripk4	3586	14	120	0.138741	1.111717	3.002325	Up	1.40E-07	3.11E-07
Tinag	1737	2	17	0.040541	0.324166	2.99928	Up	0.000518	0.000914
Epn3	3522	2	17	0.02032	0.162429	2.998851	Up	0.000518	0.000914
Sema4g	4326	53	454	0.439591	3.505297	2.995302	Up	4.04E-14	1.62E-13
Vsig8	1812	1.5	12.5	0.029147	0.231559	2.989953	Up	0.002405	0.003969
Mdh1b	1655	4	34.5	0.087178	0.6919	2.988526	Up	4.63E-05	8.85E-05
Zfp189	2945	197	1671.5	2.387613	18.93552	2.987454	Up	0	0

Cstf3	2028	738.5	6244	13.00777	102.8318	2.982841	Up	1.08E-12	3.38E-12
Timd2	1371	98	825	2.542334	20.07478	2.981159	Up	0	0
Lrrc24	1930	23	194	0.425244	3.356913	2.980773	Up	4.68E-10	1.13E-09
Klhl26	3820	583.5	4932.5	5.451356	43.01476	2.980145	Up	5.08E-13	1.70E-12
Aire	1891	3	25.5	0.057072	0.449981	2.979009	Up	8.88E-05	0.000166
LOC680200	1602	34.5	291	0.768644	6.059589	2.978833	Up	5.99E-13	1.97E-12
LOC100912972	744	3	25	0.143516	1.129127	2.975919	Up	8.88E-05	0.000166
Cdkn1a	1894	4910.5	41456	92.88415	730.3556	2.975095	Up	3.42E-11	8.63E-11
LOC100363993	4062	24.5	204.5	0.213921	1.681845	2.974893	Up	2.47E-10	6.04E-10
RGD1566314	2946	68	572.5	0.827122	6.490699	2.972202	Up	0	0
LOC100362205	1138	1.5	12.5	0.046914	0.367913	2.971275	Up	0.002405	0.003965
LOC100364265	1899	579	4845.5	10.87247	85.18786	2.969969	Up	9.45E-13	3.00E-12
Irs3	1969	9	75.5	0.163268	1.276767	2.967177	Up	2.92E-06	6.06E-06
Rdh14	1865	158	1317	3.017358	23.59498	2.967122	Up	1.62E-14	6.66E-14
Dusp8	2439	329	2764	4.850125	37.77771	2.961442	Up	1.13E-14	4.70E-14
LOC498132	2193	13	108.5	0.212121	1.650807	2.96021	Up	2.60E-07	5.70E-07
Zfp597	1610	46.5	388.5	1.033313	8.038773	2.959698	Up	7.84E-14	3.07E-13
Rho	1493	2	17	0.048703	0.378349	2.957642	Up	0.000518	0.000915
Gpr113	3145	2	17	0.02312	0.179611	2.957642	Up	0.000518	0.000914
LOC679608	1029	16	133.5	0.558625	4.336336	2.956524	Up	4.01E-08	9.10E-08
Sema4f	4008	8	67.5	0.072139	0.55992	2.95637	Up	5.24E-06	1.07E-05
LOC100911895	980	3.5	29	0.12692	0.984303	2.955189	Up	7.50E-05	0.000141
Tpd52l1	1272	2.5	20.5	0.069652	0.539843	2.954292	Up	0.000181	0.00033
LOC317588	2882	2434	20127.5	30.09069	233.182	2.954067	Up	0	0
Maff	1941	754	6257.5	13.88823	107.5992	2.953733	Up	5.14E-13	1.72E-12

Dmrt1	1415	5	41.5	0.126848	0.980143	2.949894	Up	2.77E-05	5.39E-05
Ppm1h	3132	273	2271.5	3.129839	24.1733	2.949254	Up	2.09E-13	7.61E-13
LOC100911284	501	4	33	0.285694	2.204521	2.94792	Up	4.65E-05	8.90E-05
LOC303140	2938	11.5	94	0.1386	1.068809	2.947006	Up	8.84E-07	1.89E-06
Sh3rf2	2497	2	16	0.028202	0.217176	2.945014	Up	0.000844	0.001445
Dapp1	3062	32	265	0.375458	2.891217	2.944955	Up	1.74E-12	5.25E-12
LOC100362110	1866	800	6570.5	15.27108	117.5116	2.943929	Up	7.63E-13	2.45E-12
Zfp12	4065	223	1828	1.95554	15.02155	2.941395	Up	7.02E-14	2.76E-13
LOC100362224	3369	88.5	725	0.937349	7.199181	2.941175	Up	1.80E-14	7.37E-14
LOC100363776	341	33	268	3.41749	26.23421	2.940441	Up	1.01E-12	3.19E-12
Obsl1	5808	3	25	0.018582	0.142626	2.940271	Up	8.88E-05	0.000166
Snx20	1393	5	40.5	0.126381	0.969071	2.938821	Up	2.77E-05	5.40E-05
Slc9a4	3183	2	16.5	0.022484	0.172363	2.938484	Up	0.000844	0.001445
LOC501456	2052	20	165	0.348206	2.668822	2.938192	Up	3.18E-09	7.52E-09
Snai1	1623	19.5	161	0.432931	3.314712	2.936674	Up	6.02E-09	1.41E-08
Mcmdc2	3200	19	154	0.209417	1.602803	2.936149	Up	6.02E-09	1.41E-08
Zfp84	5897	275.5	2258	1.671113	12.77945	2.934945	Up	5.96E-13	1.97E-12
Zfp52	2957	211.5	1718.5	2.549997	19.38782	2.926583	Up	2.52E-13	9.04E-13
LOC100911534	900	109	889	4.335659	32.94175	2.925594	Up	0	0
Zfp385c	1555	2	16.5	0.046023	0.349345	2.924213	Up	0.000844	0.001445
Mctp1-ps1	2879	8	64.5	0.098636	0.747516	2.921923	Up	5.24E-06	1.07E-05
Mgmt	812	4	32.5	0.176272	1.334632	2.920566	Up	4.70E-05	8.99E-05
LOC100364397	438	2	16	0.160775	1.2155	2.918432	Up	0.000844	0.001445
Lztfl1	1442	141	1132	3.485792	26.22183	2.91121	Up	0	0
Cpeb2	6178	1032.5	8271	5.942738	44.67875	2.910389	Up	1.69E-12	5.13E-12

RGD1310862	7479	216	1727	1.028084	7.708734	2.906536	Up	2.34E-13	8.42E-13
Thap1	1100	206	1652.5	6.701224	50.18269	2.904693	Up	2.46E-13	8.84E-13
Helz	6193	501.5	4022	2.897164	21.6744	2.903279	Up	7.99E-14	3.13E-13
Gsdma	2910	1294	10319.5	15.849	118.4893	2.902293	Up	2.60E-12	7.61E-12
LOC682357	1162	39	311	1.197038	8.948106	2.902113	Up	1.32E-13	4.99E-13
LOC100361114	3215	329	2628	3.646638	27.25264	2.901757	Up	1.13E-14	4.70E-14
Gpbp1	3305	2329.5	18573	25.12303	187.5792	2.900417	Up	0	0
Rora	1684	80.5	642	1.709689	12.72609	2.895983	Up	0	0
Prodh2	1466	53.5	431.5	1.315061	9.784964	2.895437	Up	4.04E-14	1.62E-13
Msi1	1089	2	16	0.065718	0.488879	2.895125	Up	0.000844	0.001445
Csf1r	3698	2	16	0.019353	0.143967	2.895125	Up	0.000844	0.001446
Ihh	2272	2.5	19.5	0.038743	0.287937	2.893739	Up	0.000237	0.000428
Errfi1	3007	1334	10623.5	15.86256	117.8796	2.893616	Up	1.66E-12	5.03E-12
Mesp2	1857	34.5	276	0.666801	4.952967	2.892965	Up	5.99E-13	1.97E-12
Mogat2	1774	2	16	0.040342	0.299599	2.892684	Up	0.000844	0.001446
Myoz3	917	2	16	0.079295	0.588429	2.89157	Up	0.000844	0.001445
Dhdh	1214	35.5	283	1.048505	7.775509	2.890603	Up	3.77E-13	1.30E-12
Pcsk4	2458	3	24	0.043907	0.325258	2.889064	Up	0.000101	0.000188
Scg5	1139	3	23.5	0.094249	0.697138	2.886894	Up	0.000123	0.000225
Vegfb	1812	757	5991	14.91373	110.3051	2.886786	Up	8.99E-13	2.86E-12
Gtf2a1l	1602	1.5	12	0.033684	0.248965	2.885812	Up	0.002405	0.003968
Slco1b3	3218	2	16	0.022239	0.164322	2.885336	Up	0.000844	0.001444
RGD1310212	3498	306	2406.5	3.118954	22.99506	2.88219	Up	0	0
Zswim3	2691	144	1136.5	1.914822	14.11559	2.882008	Up	1.05E-13	4.06E-13
Gsta5	1055	2.5	20	0.086153	0.634632	2.880942	Up	0.000181	0.00033

Cd248	2565	6	48	0.084151	0.61987	2.88092	Up	1.62E-05	3.20E-05
Sycp2	4771	2	16	0.015	0.110457	2.880416	Up	0.000844	0.001445
RGD1559709	1825	6	47.5	0.117644	0.866259	2.880377	Up	1.62E-05	3.20E-05
Zfp292	9966	1220.5	9592	4.364435	32.12224	2.879706	Up	7.20E-12	1.92E-11
Dear	3274	2	15.5	0.021509	0.1582	2.878752	Up	0.001428	0.0024
Lif	609	558.5	4412	32.8673	241.6811	2.87838	Up	1.47E-13	5.48E-13
Inpp5d	4943	63	497.5	0.456184	3.346912	2.875143	Up	0	0
RGD1561413	1567	81.5	639.5	1.862374	13.65374	2.874082	Up	5.82E-14	2.30E-13
Atp1a2	5149	6.5	52	0.045896	0.336476	2.874064	Up	1.62E-05	3.20E-05
RGD1565775	3011	1307.5	10231.5	15.47961	113.3345	2.872146	Up	2.67E-12	7.82E-12
LOC100910319	805	43.5	339	1.924011	14.06873	2.870303	Up	1.18E-13	4.49E-13
Klf6	4206	5867	45815	49.78658	363.7381	2.869071	Up	1.83E-11	4.72E-11
LOC365990	702	2	15.5	0.100313	0.732686	2.868689	Up	0.001428	0.002399
Cml1	2180	3	24	0.050032	0.365084	2.867302	Up	0.000101	0.000188
RatNP-3b	537	3.5	27	0.230554	1.682019	2.867014	Up	7.82E-05	0.000147
Slc4a11	3039	56	436.5	0.657872	4.795292	2.86574	Up	1.58E-14	6.49E-14
Cnot4	3502	1042	8150	10.66413	77.68067	2.86479	Up	1.99E-12	5.95E-12
Cyp11a1	1581	56	436	1.264563	9.207812	2.86422	Up	1.58E-14	6.49E-14
Mylk	7764	17	133	0.077834	0.566711	2.864146	Up	2.14E-08	4.89E-08
LOC100910826	615	3	23	0.171755	1.250076	2.863591	Up	0.000123	0.000225
Ovol3	573	2	16	0.126899	0.922843	2.8624	Up	0.000844	0.001445
Kctd11	2164	238	1850.5	3.929664	28.54942	2.860983	Up	6.71E-13	2.19E-12
Rasgrp3	4293	148.5	1152.5	1.233844	8.960412	2.860405	Up	0	0
Sfn	1284	13	101	0.362738	2.631128	2.858682	Up	2.60E-07	5.69E-07
Fgf20	639	2.5	19.5	0.140446	1.018142	2.857855	Up	0.000237	0.000428

Syt13	1281	18.5	144.5	0.521476	3.773493	2.855228	Up	1.14E-08	2.63E-08
Scn10a	6524	3	23	0.016367	0.118393	2.854751	Up	0.000123	0.000225
Grm4	4488	209	1624.5	1.673659	12.07755	2.85125	Up	1.08E-13	4.16E-13
RGD1562449	1679	2	16	0.043308	0.312262	2.850069	Up	0.000844	0.001446
Icam1	2602	1213.5	9387.5	16.71419	120.4563	2.849364	Up	5.51E-12	1.50E-11
Ovol1	2993	3.5	27	0.041557	0.299079	2.847351	Up	7.82E-05	0.000147
Zfp112	3446	19.5	151	0.20307	1.460623	2.846533	Up	6.02E-09	1.41E-08
Cyp2c6v1	1681	16.5	126.5	0.349357	2.51272	2.846475	Up	4.01E-08	9.10E-08
Akr1c14	2334	16	123.5	0.245792	1.76719	2.845947	Up	4.01E-08	9.10E-08
Tbx4	3254	2.5	19.5	0.02758	0.198277	2.84583	Up	0.000237	0.000428
Yjefn3	785	1.5	11.5	0.068741	0.493119	2.842696	Up	0.003992	0.006452
Prss8	2208	5.5	42	0.088225	0.632629	2.842102	Up	2.76E-05	5.39E-05
Dhrs7	1753	5	39	0.103044	0.738155	2.84066	Up	2.77E-05	5.40E-05
Taf1d	1299	1083	8288	29.75765	212.9165	2.838955	Up	4.28E-12	1.20E-11
Slc13a5	3254	15	113.5	0.163188	1.167165	2.8384	Up	7.51E-08	1.68E-07
Gpr19	1544	315	2409.5	7.289937	52.0755	2.836627	Up	4.99E-13	1.67E-12
RGD1565212	1081	3	22.5	0.097715	0.697829	2.836229	Up	0.000162	0.000294
Bmp5	3708	2	15	0.018991	0.135545	2.835368	Up	0.001428	0.0024
Abcb1a	4927	706	5398	5.125126	36.57461	2.835183	Up	3.70E-13	1.28E-12
Katnal2	1569	10	76	0.225871	1.611471	2.834806	Up	1.61E-06	3.40E-06
Ccdc117	2224	194	1482.5	3.117254	22.23251	2.834323	Up	5.28E-13	1.76E-12
LOC498029	1362	71	542	1.862405	13.28212	2.834247	Up	0	0
Fbxo15	1693	19	144	0.39989	2.850916	2.83375	Up	6.02E-09	1.41E-08
Fscn3	1795	23	176	0.456907	3.256534	2.833366	Up	4.68E-10	1.14E-09
LOC100361431	744	3	22.5	0.141975	1.009077	2.829327	Up	0.000162	0.000294

Cyp2s1	2632	7	53.5	0.095386	0.676423	2.826075	Up	9.28E-06	1.86E-05
Cacna1s	6125	8	61	0.046737	0.331183	2.824982	Up	5.24E-06	1.07E-05
Ppil6	942	13	98	0.49078	3.477184	2.824771	Up	2.60E-07	5.70E-07
LOC499742	906	61.5	463.5	2.410323	17.06891	2.824073	Up	3.49E-14	1.41E-13
LOC100911163	729	2	15.5	0.099744	0.705549	2.822446	Up	0.001428	0.002399
Krcc1	1650	830.5	6267.5	17.92523	126.7819	2.822286	Up	0	0
Slc35b3	1925	217	1632.5	4.001574	28.2878	2.82154	Up	2.24E-13	8.07E-13
Epha10	3782	2	15	0.018771	0.132655	2.821082	Up	0.001428	0.002399
Srfbp1	1540	547.5	4150.5	12.73672	89.96864	2.820429	Up	0	0
Rlf	6213	1255	9476.5	7.209881	50.89006	2.819337	Up	5.54E-12	1.51E-11
Cpg1	4207	3	23	0.025653	0.181031	2.819023	Up	0.000123	0.000225
Klb-ps1	3557	6.5	49	0.065309	0.460431	2.817631	Up	1.62E-05	3.20E-05
Itk	4199	3	23	0.025975	0.18309	2.817348	Up	0.000123	0.000225
Hoxc6	1682	72	545.5	1.534471	10.81103	2.816691	Up	0	0
LOC100362202	2649	121	913.5	1.635575	11.52167	2.81648	Up	0	0
RGD1564664	1390	65.5	498.5	1.693408	11.92084	2.815485	Up	0	0
Ctss	1330	5.5	41	0.146898	1.033955	2.815287	Up	2.77E-05	5.39E-05
Pgf	1586	473	3568.5	10.66854	75.04911	2.814472	Up	5.88E-13	1.94E-12
Siglec10	2373	68	513	1.026361	7.219146	2.814291	Up	0	0
LOC100911882	2714	7	53	0.092927	0.653317	2.813618	Up	9.28E-06	1.86E-05
Efcab11	1497	55	414	1.314298	9.233227	2.812542	Up	0	0
LOC691636	572	1.5	11.5	0.094338	0.662584	2.812187	Up	0.003992	0.006454
LOC100912887	1628	365.5	2729.5	7.984507	56.04008	2.811184	Up	6.76E-13	2.20E-12
RGD1560046	2411	69	515.5	1.018128	7.14577	2.81117	Up	0	0
Trim66	4047	4	30	0.035368	0.247938	2.809476	Up	4.94E-05	9.44E-05

Rag1	6537	9.5	71.5	0.052134	0.36497	2.807476	Up	2.92E-06	6.06E-06
Adap2	1798	2	15	0.039803	0.278032	2.80429	Up	0.001428	0.002399
Naglt1	2173	15.5	115	0.254582	1.775602	2.802107	Up	7.51E-08	1.68E-07
LOC681820	1695	2.5	19	0.053285	0.371592	2.801917	Up	0.000237	0.000428
Ehhadh	3097	50.5	377.5	0.584133	4.072932	2.801699	Up	3.62E-14	1.46E-13
Zfp26	6741	335	2492	1.770453	12.32849	2.799805	Up	1.62E-13	6.00E-13
LOC100911245	1147	109.5	818	3.412848	23.75537	2.799206	Up	0	0
Zfp770	2621	191	1419.5	2.598222	18.08376	2.799098	Up	8.66E-14	3.37E-13
LOC100362957	333	13.5	102	1.4412	10.01577	2.796931	Up	2.60E-07	5.70E-07
Cdh11	5173	14	107	0.096842	0.673011	2.796921	Up	1.40E-07	3.11E-07
LOC100366195	1074	2	15	0.067703	0.470486	2.796855	Up	0.001428	0.0024
LOC683891	1533	3	22	0.069652	0.483829	2.796265	Up	0.000162	0.000294
Tank	2077	696.5	5183	11.9947	83.30609	2.796025	Up	4.30E-13	1.46E-12
Slc5a5	2862	19	140.5	0.237955	1.6464	2.790552	Up	6.02E-09	1.41E-08
Dkk3	5010	44	324	0.313119	2.166333	2.790471	Up	8.13E-14	3.18E-13
LOC100911850	1886	65	483	1.238419	8.565552	2.790047	Up	0	0
Krt80	1359	8.5	63	0.224021	1.548395	2.78907	Up	5.24E-06	1.07E-05
LOC100909763	539	53	389.5	3.494105	24.13899	2.78837	Up	4.04E-14	1.62E-13
Cd101	3053	8	58.5	0.092638	0.638709	2.785475	Up	5.24E-06	1.07E-05
Zfp141	4219	75.5	557	0.638107	4.395841	2.784269	Up	0	0
Myh13	5678	6	44	0.037611	0.259038	2.783958	Up	1.62E-05	3.20E-05
LOC499806	1679	17	125.5	0.361283	2.487787	2.783661	Up	2.14E-08	4.89E-08
RGD1565073	312	2	15	0.22938	1.579173	2.783359	Up	0.001428	0.0024
Xkr7	2718	16	119	0.212543	1.463072	2.783173	Up	4.01E-08	9.10E-08
RGD1562465	942	56.5	416.5	2.143495	14.75367	2.783037	Up	1.58E-14	6.49E-14

Zfp46	4501	489.5	3600.5	3.893403	26.71792	2.778704	Up	5.92E-13	1.95E-12
Nrap	5378	3	21.5	0.019641	0.134728	2.778107	Up	0.00023	0.000417
LOC100912188	1479	21	154	0.507691	3.477599	2.77607	Up	1.68E-09	4.01E-09
Ammecr1l	4267	893	6554	7.482283	51.25089	2.776027	Up	0	0
Rhod	1486	39	287.5	0.941059	6.444588	2.775731	Up	1.32E-13	4.99E-13
Zfp23	4888	192	1403	1.401807	9.578579	2.772524	Up	0	0
LOC100361380	414	35	256	3.023767	20.65803	2.772284	Up	3.77E-13	1.30E-12
Socs4	2037	385	2811	6.742604	46.06221	2.772206	Up	6.91E-13	2.24E-12
LOC100174910	1114	157	1148	5.035338	34.37995	2.771407	Up	0	0
Tcp11	1963	2.5	18	0.044842	0.306145	2.771298	Up	0.000337	0.000602
LOC100125368	1990	143	1041	2.556086	17.44752	2.771014	Up	0	0
LOC687141	1431	149	1091	3.727861	25.44291	2.770844	Up	2.66E-13	9.48E-13
Zfp709l2	2382	114	831.5	1.706289	11.62904	2.768799	Up	7.79E-14	3.06E-13
LOC297970	4512	9832	71519.5	77.72866	529.2402	2.767404	Up	0	0
LOC682870	5492	103.5	750.5	0.672529	4.56545	2.763089	Up	0	0
Mef2b	1346	21	153	0.558708	3.786391	2.760656	Up	1.68E-09	4.01E-09
Ces2g	2480	3	22	0.043517	0.294722	2.759689	Up	0.000162	0.000294
Manba	3688	53	384	0.514083	3.476528	2.757574	Up	4.04E-14	1.62E-13
B3gntl1	1511	53	383.5	1.25248	8.465713	2.756844	Up	4.04E-14	1.62E-13
Ddc8	1809	17	123.5	0.337222	2.276573	2.755092	Up	2.14E-08	4.90E-08
Bpifc	2228	8	58	0.129	0.869944	2.753548	Up	5.24E-06	1.07E-05
Trpm5	3471	3	22	0.031093	0.209539	2.752565	Up	0.000162	0.000294
Enkur	1205	7.5	54.5	0.224859	1.514881	2.752114	Up	9.28E-06	1.86E-05
Frmd4b	5031	53	383.5	0.377763	2.544006	2.751549	Up	4.04E-14	1.62E-13
Magi2	4767	4	29	0.030026	0.201976	2.749906	Up	5.21E-05	9.94E-05

Sbsn	690	9.5	68	0.488928	3.287686	2.749379	Up	2.92E-06	6.06E-06
Snai2	807	741.5	5355	32.96168	221.5612	2.748844	Up	0	0
Lctl	1371	4.5	32.5	0.11766	0.789804	2.746876	Up	4.70E-05	8.99E-05
Txlnb	4044	8	57	0.070788	0.474592	2.745114	Up	5.24E-06	1.07E-05
Dusp1	1940	378.5	2723	6.984228	46.78174	2.743773	Up	6.61E-13	2.16E-12
Reep1	3680	2	14	0.019136	0.127993	2.741726	Up	0.002465	0.004058
Klf10	2962	1451.5	10404	17.53165	117.2271	2.741273	Up	2.10E-12	6.25E-12
Tusc5	3410	3.5	25	0.036812	0.245827	2.739406	Up	8.88E-05	0.000166
Ushbp1	2483	8.5	60.5	0.12215	0.814761	2.737729	Up	5.24E-06	1.07E-05
Cxcr4	1726	8	57	0.165855	1.106227	2.737653	Up	5.24E-06	1.07E-05
LOC100362987	283	4.5	32.5	0.563926	3.759437	2.73694	Up	4.70E-05	8.99E-05
Gnat2	1525	3	21	0.069265	0.461523	2.736202	Up	0.00023	0.000416
LOC100910406	442	424.5	3032.5	34.36064	228.8433	2.735532	Up	0	0
LOC100912058	686	15.5	111	0.811439	5.403551	2.735353	Up	7.51E-08	1.69E-07
Ccdc79	2325	5	35.5	0.076707	0.510745	2.735181	Up	2.84E-05	5.54E-05
Slc12a3	4382	8	57	0.065328	0.434082	2.732202	Up	5.24E-06	1.07E-05
LOC100360852	351	2	14.5	0.203893	1.352303	2.729534	Up	0.002465	0.004059
LOC100364391	1783	2328.5	16495	46.6166	309.0877	2.7291	Up	0	0
RGD1306880	2374	3	21	0.044494	0.294955	2.728803	Up	0.00023	0.000416
LOC100911556	624	29	206.5	1.660246	11.00469	2.728649	Up	1.04E-11	2.73E-11
Tm4sf19	766	9.5	68	0.445658	2.950918	2.727154	Up	2.92E-06	6.06E-06
LOC683963	792	32	229.5	1.457372	9.643336	2.726163	Up	1.74E-12	5.25E-12
Fam65c	2817	15	107	0.19115	1.26432	2.725586	Up	7.51E-08	1.68E-07
RGD1561916	1122	2	14	0.063785	0.421404	2.723922	Up	0.002465	0.00406
RGD1305537	1717	171.5	1203.5	3.544264	23.39952	2.722921	Up	3.66E-13	1.27E-12

Slc38a3	2354	14	99	0.212814	1.404883	2.722782	Up	1.40E-07	3.11E-07
Fam151b	1377	8	57	0.208724	1.37745	2.722332	Up	5.24E-06	1.07E-05
LOC100360638	3569	245.5	1734	2.454263	16.19401	2.722098	Up	3.55E-13	1.23E-12
Itgb2	3161	16.5	116	0.185967	1.222642	2.71688	Up	4.01E-08	9.11E-08
Atp8b3	4100	4	28	0.034631	0.227568	2.716175	Up	5.69E-05	0.000108
Pigr	3923	81.5	570	0.738643	4.853067	2.715948	Up	5.82E-14	2.30E-13
Trmt1l	2642	536	3766	7.249597	47.58767	2.714615	Up	0	0
Dnajb13	2280	24.5	172	0.383381	2.516286	2.714445	Up	2.47E-10	6.04E-10
Etv3l	1783	2.5	17.5	0.04969	0.325922	2.713489	Up	0.000518	0.000914
LOC366449	1184	5	35	0.150627	0.987701	2.713092	Up	2.84E-05	5.54E-05
Esam	1837	2	14	0.038958	0.255425	2.712895	Up	0.002465	0.004059
Sec14l5	2233	4	28.5	0.064613	0.423499	2.712473	Up	5.69E-05	0.000108
LOC100911299	1145	34.5	242.5	1.076933	7.05725	2.712178	Up	5.99E-13	1.97E-12
Fam167a	3483	465.5	3264.5	4.776883	31.27206	2.710733	Up	0	0
Mettl6	1671	577.5	4040.5	12.33431	80.69447	2.709793	Up	5.81E-13	1.92E-12
Mir881	77	3	21	1.386705	9.070428	2.70951	Up	0.00023	0.000416
Slc4a9	3175	3	21	0.03363	0.219976	2.70951	Up	0.00023	0.000416
LOC100362270	1004	4	28	0.142563	0.931106	2.70735	Up	5.69E-05	0.000108
LOC689959	1347	8.5	59.5	0.227294	1.484459	2.707308	Up	5.24E-06	1.07E-05
Adam1a	2917	24	167	0.292052	1.905553	2.705911	Up	2.47E-10	6.04E-10
Pdik1l	4489	195	1363	1.551852	10.11522	2.704465	Up	1.92E-13	7.02E-13
Zfp445	6069	933.5	6470	5.481727	35.58316	2.698492	Up	3.87E-13	1.32E-12
Pih1d2	1304	176	1226	4.828759	31.32142	2.697425	Up	0	0
Ppp1r10	4170	2814	19594	24.18108	156.7729	2.696726	Up	0	0
Zfp939	3144	6	41.5	0.067924	0.440268	2.696392	Up	1.63E-05	3.22E-05

Cilp2	4081	6	42	0.052891	0.342721	2.695955	Up	1.62E-05	3.21E-05
Slc10a5	1305	19.5	135.5	0.533154	3.454531	2.695865	Up	6.02E-09	1.41E-08
Ctrb1	792	127	881.5	5.730004	37.12503	2.695784	Up	9.17E-14	3.56E-13
Meis1	2506	595.5	4138	8.503959	55.08932	2.695566	Up	1.07E-12	3.35E-12
Trim17	1703	3	21	0.063372	0.410113	2.694097	Up	0.00023	0.000416
Cd46	1192	26	179.5	0.778101	5.031781	2.693039	Up	6.87E-11	1.72E-10
RGD1307621	1267	34	235	0.954361	6.169061	2.692444	Up	5.99E-13	1.97E-12
Rnf144b	3160	121.5	837	1.374482	8.870052	2.690055	Up	0	0
Eml2	2490	3	21	0.043803	0.28266	2.689959	Up	0.00023	0.000417
Acta1	1518	55.5	381	1.292981	8.317439	2.685438	Up	0	0
Ing3	2210	566.5	3904	9.171322	58.94616	2.684196	Up	8.82E-14	3.43E-13
Spata1	1897	15	102	0.28083	1.804668	2.683965	Up	7.51E-08	1.68E-07
Slc10a1	1663	4	27.5	0.086759	0.556697	2.681812	Up	6.52E-05	0.000124
Ppm1k	2624	251.5	1730.5	3.426949	21.94502	2.678896	Up	1.37E-13	5.15E-13
Ifi204	1768	1096	7515.5	22.16745	141.8411	2.677761	Up	4.94E-12	1.36E-11
Ccdc58	1216	795	5436	23.33127	149.196	2.676872	Up	0	0
Ceacam9	980	2.5	17	0.091576	0.585588	2.676839	Up	0.000518	0.000914
Pnma2	3327	9	62	0.096626	0.617307	2.675501	Up	2.92E-06	6.05E-06
Stx11	1642	251	1717.5	5.46048	34.87125	2.674938	Up	1.37E-13	5.15E-13
Amn1	1310	249.5	1700.5	6.793973	43.34979	2.673698	Up	1.13E-14	4.69E-14
Gimap9	1700	23.5	160	0.49347	3.144406	2.671753	Up	4.68E-10	1.13E-09
Mis18a	1099	892.5	6091.5	28.97589	184.6348	2.671749	Up	4.11E-13	1.40E-12
Spata18	2052	3	20.5	0.052594	0.335078	2.671523	Up	0.000351	0.000627
Rsph3	1896	44	300	0.831017	5.290319	2.670404	Up	8.13E-14	3.18E-13
Snap25	2135	5.5	37	0.091779	0.583238	2.667852	Up	2.79E-05	5.43E-05

Pou4f3	2272	2	13.5	0.031499	0.200164	2.667788	Up	0.004296	0.006925
LOC498826	771	247.5	1672.5	11.42101	72.50817	2.666453	Up	0	0
LOC501406	12571	303.5	2055.5	0.862062	5.465957	2.66461	Up	2.56E-13	9.16E-13
LOC100361238	1547	9	61.5	0.208918	1.324653	2.664607	Up	2.92E-06	6.05E-06
Dcst1	2205	4	27.5	0.065953	0.417817	2.663359	Up	6.52E-05	0.000124
Slc7a3	2152	2	13.5	0.033256	0.210489	2.662066	Up	0.004296	0.006929
LOC679087	3836	78	525	0.722821	4.57124	2.660875	Up	0	0
Pigl	1410	100	677	2.535378	16.03296	2.660768	Up	1.75E-14	7.19E-14
LOC100361997	1421	247.5	1670	6.201203	39.15417	2.658546	Up	0	0
RGD1562550	726	30	204	1.480226	9.344227	2.658258	Up	5.60E-12	1.52E-11
LOC100360287	3434	105.5	710	1.095248	6.912358	2.65792	Up	0	0
Zswim6	5258	765	5161.5	5.195725	32.77518	2.657207	Up	0	0
LOC501386	549	27.5	185.5	1.792943	11.30474	2.656527	Up	3.64E-11	9.17E-11
Sh2b2	2856	24	161.5	0.300298	1.891569	2.655117	Up	2.47E-10	6.04E-10
Hsd17b13	1323	4	27.5	0.109055	0.686838	2.654914	Up	6.52E-05	0.000124
LOC688903	721	20	134	0.986237	6.200045	2.652272	Up	3.18E-09	7.52E-09
Serpine3	1407	4	27	0.101729	0.638766	2.650554	Up	6.52E-05	0.000124
Letm2	1787	143	962.5	2.863781	17.95862	2.648684	Up	0	0
LOC363324	963	8.5	57.5	0.316141	1.979949	2.646821	Up	5.24E-06	1.07E-05
Sult1b1	1276	17.5	116.5	0.489185	3.062707	2.646357	Up	2.14E-08	4.89E-08
Suv420h1	6096	832	5570	4.875053	30.49093	2.644891	Up	0	0
Tsga10ip	1824	3	20	0.059168	0.370031	2.644748	Up	0.000351	0.000627
RGD1564425	618	3	20	0.172777	1.080479	2.644687	Up	0.000351	0.000627
Trit1	2113	389	2593.5	6.563755	40.96902	2.64194	Up	0	0
Rbm39	2041	1238	8262	21.64699	135.0449	2.641201	Up	2.93E-12	8.49E-12

LOC367042	1085	5.5	36.5	0.178483	1.112782	2.640313	Up	2.81E-05	5.47E-05
Avpr2	1116	6	40.5	0.193411	1.203957	2.638044	Up	1.64E-05	3.23E-05
Slc25a34	1337	9.5	64	0.255757	1.59144	2.637485	Up	2.92E-06	6.05E-06
Inpp4b	4398	58	389	0.473989	2.948538	2.637075	Up	1.35E-14	5.59E-14
Hdgfl1	2068	8	52.5	0.136485	0.846933	2.633501	Up	5.24E-06	1.07E-05
LOC501421	711	72.5	483	3.660477	22.71214	2.63336	Up	0	0
Birc2	2907	759.5	5018.5	9.301498	57.58949	2.630271	Up	1.87E-12	5.61E-12
Stambp	1735	398.5	2639	8.19583	50.73383	2.629986	Up	5.90E-13	1.95E-12
Pnldc1	2027	26	172	0.457854	2.830356	2.628024	Up	6.87E-11	1.72E-10
RGD1566400	3921	7	46	0.063736	0.39398	2.627938	Up	9.30E-06	1.87E-05
RGD1309748	3841	1177.5	7766.5	10.92159	67.5012	2.62773	Up	2.81E-12	8.18E-12
Wisp1	1888	380.5	2516	7.216318	44.47167	2.623552	Up	0	0
Irf2	1368	289.5	1910	7.561859	46.59818	2.623461	Up	0	0
Frmpd4	5123	3	20	0.021066	0.129638	2.621475	Up	0.000351	0.000627
Chic2	961	583.5	3831	21.65078	133.0478	2.619454	Up	5.08E-13	1.70E-12
Cyp27b1	2426	33.5	220	0.495421	3.036562	2.615711	Up	1.01E-12	3.18E-12
Gata3	1335	43	283.5	1.157293	7.082255	2.613454	Up	1.18E-13	4.48E-13
Pglyrp1	630	18.5	121.5	1.052143	6.435314	2.61268	Up	1.14E-08	2.63E-08
Ngf	1162	763.5	5011.5	23.56417	144.0321	2.611723	Up	8.47E-13	2.71E-12
LOC100911217	501	2	13	0.142847	0.871714	2.609382	Up	0.004296	0.006927
Il9r	3692	7	45.5	0.067379	0.411092	2.609094	Up	9.32E-06	1.87E-05
Ccdc112	2220	547	3574	8.817635	53.738	2.607479	Up	0	0
Agbl4	675	14.5	94.5	0.765703	4.661487	2.605932	Up	1.40E-07	3.11E-07
Trim31	2451	12.5	81	0.182142	1.108132	2.604994	Up	4.81E-07	1.04E-06
Gcm2	3365	2	13	0.021097	0.128181	2.603038	Up	0.004296	0.006925

Apom	787	10	65	0.454679	2.760919	2.602228	Up	1.61E-06	3.40E-06
Siae	3460	450.5	2934	4.659805	28.27535	2.601203	Up	1.46E-12	4.47E-12
Ppp1r32	1491	4	26	0.096767	0.587027	2.600838	Up	7.99E-05	0.00015
RGD1307934	2556	317.5	2059.5	4.432453	26.8784	2.60027	Up	2.57E-13	9.19E-13
Rxfp1	2277	27	175	0.423048	2.564775	2.599939	Up	3.64E-11	9.17E-11
Nupl2	2435	388.5	2521.5	5.705502	34.55527	2.59848	Up	6.64E-13	2.16E-12
Peo1	3601	843	5464	8.360352	50.6097	2.597778	Up	2.43E-12	7.16E-12
LOC100909512	2496	11	71	0.157009	0.950426	2.597724	Up	8.84E-07	1.88E-06
LOC100360623	6076	34	220	0.199669	1.208574	2.597624	Up	5.99E-13	1.97E-12
Adam4	2472	26	168.5	0.376825	2.280675	2.597495	Up	6.87E-11	1.72E-10
Syt16	3051	2	13	0.023457	0.141963	2.59744	Up	0.004296	0.006928
LOC100911030	685	4	26	0.210627	1.272492	2.594892	Up	7.99E-05	0.00015
LOC100910868	2216	4.5	29	0.072535	0.43814	2.594639	Up	5.21E-05	9.94E-05
LOC100910297	1118	6	39	0.193065	1.164656	2.592748	Up	1.65E-05	3.26E-05
Cwc15	1376	2551.5	16428	66.13008	398.8319	2.592402	Up	0	0
LOC100912695	796	8	51.5	0.35819	2.15725	2.590397	Up	5.24E-06	1.07E-05
LOC100909641	1941	16.5	107	0.304924	1.835388	2.589565	Up	4.01E-08	9.10E-08
LOC499418	1336	2	13	0.053568	0.321503	2.585398	Up	0.004296	0.006929
Htr1d	1125	8	51	0.25242	1.514336	2.584789	Up	5.24E-06	1.07E-05
LOC100911950	1020	35	224.5	1.223921	7.341754	2.584615	Up	3.77E-13	1.30E-12
LOC100910201	2116	189.5	1212	3.198226	19.12836	2.580369	Up	2.87E-13	1.02E-12
Slc26a5	2235	3	19	0.047261	0.282617	2.580113	Up	0.000562	0.000989
Pxdc1	1815	888	5685	17.48474	104.5003	2.57934	Up	0	0
Mylip	3069	20	127	0.23207	1.385411	2.577679	Up	3.18E-09	7.52E-09
Depdc7	1732	1117.5	7141	23.06163	137.5684	2.576583	Up	4.68E-12	1.29E-11

Rfc5	1644	2909	18575.5	63.20381	376.946	2.576275	Up	0	0
Arhgef15	3641	9	57.5	0.088293	0.526392	2.575761	Up	2.92E-06	6.05E-06
Dnase1l3	1417	8	51	0.201213	1.198467	2.574393	Up	5.24E-06	1.07E-05
LOC100910680	1043	3	19	0.102374	0.60906	2.572732	Up	0.000562	0.000989
Zan	14777	8	50.5	0.019217	0.114312	2.572505	Up	5.25E-06	1.07E-05
LOC691962	435	144	917	11.8323	70.30231	2.570841	Up	1.05E-13	4.06E-13
Fbxo4	1993	165	1048.5	2.954141	17.55164	2.570795	Up	4.11E-14	1.65E-13
LOC100910900	1000	66.5	423	2.379298	14.11902	2.569032	Up	0	0
RGD1308114	1734	11	69.5	0.226006	1.340504	2.568339	Up	8.84E-07	1.88E-06
Atp2a3	4472	30	191.5	0.241074	1.42959	2.568051	Up	5.60E-12	1.52E-11
Popdc2	2243	25	159.5	0.399599	2.369526	2.567972	Up	1.30E-10	3.22E-10
Il23a	1568	44	279	1.00339	5.94236	2.566154	Up	8.13E-14	3.18E-13
LOC100909776	656	8.5	54	0.464092	2.748305	2.56606	Up	5.24E-06	1.07E-05
Rassf6	1948	11.5	72.5	0.209921	1.24281	2.565685	Up	8.84E-07	1.88E-06
Gnl3	1810	2022	12772	39.79098	235.4559	2.564943	Up	0	0
Dcx	8827	11.5	72.5	0.046327	0.274067	2.564612	Up	8.84E-07	1.88E-06
LOC500034	4208	1163.5	7325	9.827256	58.11556	2.564064	Up	4.69E-12	1.29E-11
Agphd1	2504	29	183	0.413277	2.443964	2.564041	Up	1.04E-11	2.73E-11
LOC100910727	1172	355.5	2257.5	10.86701	64.25382	2.563827	Up	5.33E-13	1.78E-12
Asb15	2075	51	323.5	0.878662	5.19363	2.563362	Up	8.44E-15	3.52E-14
LOC100909462	1386	11.5	72	0.292972	1.730484	2.562339	Up	8.84E-07	1.88E-06
Hbegf	1550	2630.5	16642.5	60.68895	358.4318	2.562193	Up	0	0
RGD1303142	1566	923.5	5846.5	21.09638	124.5615	2.561791	Up	0	0
Ccdc13	1986	10	64	0.181332	1.070471	2.561537	Up	1.61E-06	3.40E-06
LOC100912996	943	389.5	2456	14.74871	87.06322	2.561474	Up	0	0

Gjb1	1555	2	13	0.046023	0.271594	2.561009	Up	0.004296	0.00693
Vom2r52	2583	23.5	147.5	0.323001	1.904957	2.560148	Up	4.68E-10	1.14E-09
Als2cr12	1874	20	127	0.38128	2.247238	2.559232	Up	3.18E-09	7.52E-09
Aplf	2572	208.5	1315	2.894648	17.0583	2.559014	Up	3.49E-13	1.21E-12
Zfp608	6070	803.5	5075.5	4.740063	27.91891	2.558265	Up	2.31E-14	9.44E-14
Zfp786	2794	35	222	0.449277	2.645695	2.557969	Up	3.77E-13	1.30E-12
RGD1562626	2289	128	804.5	1.993469	11.73208	2.557106	Up	3.69E-14	1.49E-13
LOC100911353	718	25	156	1.238746	7.290311	2.5571	Up	1.30E-10	3.22E-10
Dusp13	1296	5	32	0.13938	0.820199	2.55695	Up	3.19E-05	6.19E-05
N5	2162	15.5	97	0.254286	1.49543	2.556038	Up	7.51E-08	1.68E-07
Zfp655	4024	1016	6381.5	9.005092	52.92544	2.555149	Up	3.94E-13	1.35E-12
Rasgrp4	2164	5.5	34.5	0.090549	0.532068	2.554843	Up	2.90E-05	5.65E-05
LOC683603	2171	20	126	0.330176	1.937696	2.553036	Up	3.18E-09	7.52E-09
Rnf39	2001	62	389.5	1.105287	6.484664	2.552611	Up	0	0
Nfkbid	2013	81.5	515	1.455159	8.537113	2.552571	Up	5.82E-14	2.30E-13
LOC100912731	1179	478.5	2980.5	14.41838	84.4561	2.550292	Up	1.04E-12	3.26E-12
Cacna1d	7986	32.5	204	0.146163	0.855561	2.549295	Up	1.74E-12	5.25E-12
Phospho2	2131	316.5	1980.5	5.304769	31.04389	2.548948	Up	3.85E-13	1.32E-12
Efemp1	2038	3	19.5	0.053518	0.313048	2.548285	Up	0.000562	0.000989
Etl4	7387	16	99.5	0.07704	0.450377	2.547463	Up	4.01E-08	9.10E-08
Dsc3	2878	5.5	34.5	0.068882	0.401944	2.544803	Up	2.90E-05	5.65E-05
Blk	2290	40.5	253	0.632722	3.689766	2.543885	Up	1.16E-13	4.43E-13
Rccd1	1137	105	656	3.301999	19.24771	2.543276	Up	0	0
Murc	1089	7.5	46.5	0.245125	1.4285	2.542914	Up	9.30E-06	1.87E-05
LOC100360752	1118	40	248	1.276155	7.434195	2.542372	Up	1.16E-13	4.44E-13

RGD1624210	1617	4	25	0.088518	0.515628	2.542294	Up	0.000105	0.000196
Irg1	2143	9	56	0.149477	0.870351	2.541675	Up	2.92E-06	6.05E-06
Sfxn4	1304	61	378.5	1.662915	9.680182	2.54132	Up	3.49E-14	1.41E-13
Lyve1	3083	5	31	0.057475	0.334249	2.539915	Up	3.51E-05	6.79E-05
Znf397	2027	70	434.5	1.22979	7.142683	2.538054	Up	0	0
LOC100909579	2044	38	237	0.665246	3.861044	2.537031	Up	1.45E-13	5.43E-13
Hpgd	1728	5	31	0.103208	0.598432	2.535639	Up	3.51E-05	6.79E-05
Il11	1674	198	1225	4.224204	24.48235	2.53499	Up	0	0
Nudt7	2877	88	547	1.091527	6.323699	2.534421	Up	1.80E-14	7.37E-14
Mafk	2670	1026.5	6367	13.75249	79.59578	2.532999	Up	2.32E-12	6.87E-12
Gucy2g	3442	6	37	0.062043	0.358632	2.531161	Up	1.71E-05	3.38E-05
Dctn3l1	561	4	24.5	0.25105	1.449245	2.529254	Up	0.00015	0.000273
Pkd1l1	7851	23.5	144.5	0.106487	0.614666	2.529122	Up	4.68E-10	1.13E-09
RGD1562963	1609	23	142	0.509725	2.939793	2.527924	Up	4.68E-10	1.14E-09
Egr1	1527	5516	34236	129.9384	748.9387	2.52702	Up	2.72E-11	6.93E-11
LOC100910671	2938	38	234	0.461648	2.65882	2.525921	Up	1.45E-13	5.43E-13
Neu4	2545	5.5	33.5	0.076543	0.440357	2.52434	Up	3.01E-05	5.85E-05
Thrsp	1300	4	24.5	0.10922	0.627482	2.522337	Up	0.00015	0.000273
LOC100909849	3376	102	629	1.081808	6.211938	2.521599	Up	0	0
Mmp12	1831	13	79.5	0.254059	1.457238	2.520001	Up	2.60E-07	5.70E-07
LOC100362429	927	8	48.5	0.305097	1.748231	2.518554	Up	5.27E-06	1.07E-05
Aox4	4879	3	18.5	0.021885	0.125396	2.518483	Up	0.000929	0.001583
RGD1305539	3039	5	30.5	0.058685	0.336113	2.517891	Up	4.05E-05	7.83E-05
LOC100911641	3719	71	433	0.680214	3.890586	2.515928	Up	0	0
LOC691135	2235	81.5	500.5	1.306258	7.469507	2.515573	Up	5.82E-14	2.30E-13

Hmox2-ps1	2211	1337	8168.5	21.61751	123.3069	2.511981	Up	8.66E-12	2.29E-11
Fam35a	3372	354.5	2163.5	3.754511	21.40641	2.511346	Up	0	0
Tmem86b	1123	19.5	119.5	0.623135	3.552337	2.511153	Up	6.02E-09	1.41E-08
Slc16a4	1981	10	60.5	0.179474	1.023045	2.511021	Up	1.61E-06	3.40E-06
RGD1310788	2173	27	164.5	0.444614	2.534276	2.510947	Up	3.64E-11	9.17E-11
Rsbn1	6739	426	2590.5	2.251625	12.8341	2.510944	Up	5.03E-13	1.69E-12
Lrrc17	2071	2.5	15	0.042503	0.242251	2.510854	Up	0.001428	0.0024
Tgm1	2707	63	383.5	0.830453	4.7304	2.509992	Up	0	0
Asmt	1548	10	61.5	0.231899	1.320309	2.509309	Up	1.61E-06	3.40E-06
Ctrl	798	10	61	0.451286	2.569043	2.509118	Up	1.61E-06	3.40E-06
RGD1560436	1083	37	226	1.225159	6.968808	2.507943	Up	1.86E-13	6.81E-13
Ttll13	3394	30.5	185.5	0.319621	1.817738	2.50771	Up	5.60E-12	1.52E-11
Hoxa9l	2216	24.5	149	0.394453	2.243247	2.507662	Up	2.47E-10	6.04E-10
Crisp2	1440	3	18.5	0.074947	0.426115	2.507308	Up	0.000929	0.001583
Klhl28	3036	177	1071.5	2.075788	11.77955	2.504553	Up	0	0
Gem	2093	1056	6419.5	18.03979	102.3666	2.504491	Up	4.40E-13	1.49E-12
LOC100910882	2725	3747.5	22705	49.10062	278.0326	2.501441	Up	0	0
Celsr3	11868	13	79	0.039051	0.221104	2.501277	Up	2.60E-07	5.70E-07
RGD1565712	3551	1737.5	10502.5	17.51039	98.82691	2.496693	Up	4.86E-12	1.34E-11
Fam26d	948	12	73	0.455372	2.569976	2.496638	Up	4.81E-07	1.04E-06
LOC500124	2014	12	72	0.211499	1.19357	2.496563	Up	4.81E-07	1.04E-06
Dyrk3	2146	241	1461	4.025742	22.70169	2.495473	Up	2.12E-13	7.71E-13
Zfp329	8686	260	1564.5	1.067146	6.015256	2.494868	Up	4.56E-13	1.54E-12
LOC360919	2633	53	320.5	0.720068	4.05561	2.493715	Up	4.04E-14	1.62E-13
Tmc5	3044	11	66.5	0.12912	0.725977	2.491207	Up	8.84E-07	1.88E-06

LOC100910345	1528	3	18	0.07063	0.396834	2.490177	Up	0.000929	0.001583
Syt15	4070	6.5	40	0.058063	0.325915	2.488791	Up	1.64E-05	3.23E-05
Rab11fip4	3127	7	42	0.080287	0.450159	2.487203	Up	9.49E-06	1.90E-05
Sim2	4150	5	30	0.042974	0.240917	2.486993	Up	4.05E-05	7.83E-05
Atxn7l4	2565	208.5	1256	2.911267	16.3204	2.486958	Up	3.49E-13	1.21E-12
Atl1	2585	163	979	2.256573	12.64715	2.486607	Up	2.37E-13	8.51E-13
Loxl4	3789	49	294.5	0.463814	2.599147	2.486419	Up	3.91E-14	1.57E-13
Ska2	1214	566.5	3394.5	16.64661	93.23408	2.485629	Up	8.82E-14	3.43E-13
Slc16a7	2178	125	748	2.048936	11.47403	2.485425	Up	3.11E-15	1.31E-14
Zfp418	2715	120	720.5	1.583269	8.862596	2.484823	Up	0	0
RGD1564148	1317	3.5	21	0.094007	0.526213	2.484801	Up	0.00023	0.000417
Dnah9	13766	3	18	0.007757	0.043394	2.484016	Up	0.000929	0.001583
RGD1565796	1185	3	18	0.090107	0.504103	2.484016	Up	0.000929	0.001583
Zfp36l1	2741	2947.5	17705	38.57016	215.6529	2.483154	Up	0	0
Apoe	1191	71	426	2.130766	11.90636	2.482289	Up	0	0
LOC100360358	1249	184.5	1103.5	5.273667	29.46829	2.482285	Up	3.15E-13	1.11E-12
RGD1561551	903	31	185	1.226534	6.853002	2.482149	Up	3.08E-12	8.90E-12
Obfc2a	2321	271.5	1623.5	4.176742	23.33237	2.481883	Up	8.22E-14	3.21E-13
Osgin1	2355	86.5	520.5	1.319079	7.364409	2.481039	Up	0	0
Cbll1	4045	718	4286.5	6.340577	35.38334	2.480384	Up	7.05E-13	2.28E-12
Alx1	1760	40.5	242.5	0.824236	4.598893	2.480158	Up	1.16E-13	4.43E-13
Slc25a47	1550	14.5	86.5	0.334561	1.861167	2.475865	Up	1.40E-07	3.11E-07
Cpeb4	2830	784.5	4677.5	9.924785	55.17896	2.475011	Up	1.17E-13	4.46E-13
LOC100365363	2025	174	1031.5	3.059982	16.99666	2.473657	Up	1.43E-13	5.34E-13
Immp1l	920	260.5	1548	10.10187	56.09965	2.473369	Up	4.56E-13	1.54E-12

Zfp862	3546	153	909.5	1.542006	8.560845	2.472945	Up	0	0
LOC314776	983	6	35.5	0.216079	1.198862	2.472035	Up	1.90E-05	3.75E-05
Prpf39	4674	1390	8222.5	10.58759	58.71896	2.471453	Up	3.51E-12	1.00E-11
Phf11l	1351	4	23.5	0.105097	0.582413	2.470321	Up	0.000226	0.000409
RGD1562552	2040	36	215	0.631469	3.497531	2.469553	Up	2.57E-13	9.17E-13
Lrtm2	3681	33	196.5	0.321107	1.778228	2.469316	Up	1.01E-12	3.18E-12
RGD1563270	876	6	35	0.241163	1.334975	2.468733	Up	1.90E-05	3.75E-05
Usp13	5043	71.5	426	0.509327	2.814769	2.466352	Up	0	0
RGD1564162	1609	4	23.5	0.088601	0.489584	2.466156	Up	0.000226	0.000409
Cyp24a1	1545	3	18	0.070224	0.387807	2.465298	Up	0.000929	0.001583
Zfp758	1757	132.5	781	2.686269	14.83147	2.464986	Up	1.14E-13	4.37E-13
RGD1563533	7745	14.5	86.5	0.067326	0.37166	2.464756	Up	1.40E-07	3.11E-07
RGD1563015	1698	40	236.5	0.84599	4.666332	2.463577	Up	1.16E-13	4.44E-13
LOC100909889	943	405	2389.5	15.34083	84.59951	2.463273	Up	0	0
Tekt2	1555	6	36	0.139545	0.769469	2.463129	Up	1.78E-05	3.51E-05
Fam43a	2436	245.5	1452	3.609646	19.88647	2.461858	Up	3.55E-13	1.23E-12
Gfer	1227	456	2694.5	13.30402	73.27328	2.461425	Up	1.95E-12	5.83E-12
LOC100188933	726	45	264	2.204541	12.14066	2.461296	Up	2.49E-14	1.02E-13
Rhbdl2	973	48.5	286.5	1.78394	9.821039	2.460809	Up	1.06E-13	4.07E-13
LOC100364132	1989	14	82.5	0.250715	1.380071	2.460624	Up	1.40E-07	3.10E-07
Ascl3	689	8	47	0.416312	2.290538	2.459948	Up	5.29E-06	1.08E-05
Klf5	3232	350	2066.5	3.883202	21.34195	2.458374	Up	3.03E-13	1.07E-12
RGD1566319	2723	708	4177.5	9.319487	51.18893	2.457509	Up	1.11E-12	3.47E-12
Agt	1958	15	88.5	0.273252	1.500553	2.457188	Up	7.51E-08	1.68E-07
Slc38a4	1946	7	41	0.128127	0.70296	2.455864	Up	9.64E-06	1.93E-05

LOC100359687	1082	153.5	900	5.060293	27.76177	2.455807	Up	0	0
Aim1l	4629	7.5	44.5	0.058534	0.321055	2.455469	Up	9.35E-06	1.88E-05
Nat2	1385	46	270.5	1.190125	6.525547	2.454986	Up	7.84E-14	3.07E-13
Ets1	5031	4165	24456	29.61328	162.251	2.453911	Up	1.66E-11	4.31E-11
LOC100910119	4720	38	222.5	0.288085	1.578139	2.453656	Up	1.45E-13	5.44E-13
LOC100911152	1586	110.5	646.5	2.48749	13.60802	2.451695	Up	0	0
Bcl3	1788	258.5	1513.5	5.168067	28.24429	2.450262	Up	2.94E-13	1.04E-12
Plekhf1	1504	227.5	1335.5	5.416693	29.59158	2.449702	Up	3.55E-15	1.49E-14
Strn3	4152	2882	16801.5	24.73867	135.0149	2.448278	Up	0	0
Tcte4	924	23	134	0.890088	4.847655	2.445268	Up	4.68E-10	1.13E-09
Slc38a6	2318	135	787.5	2.082774	11.34239	2.445147	Up	2.15E-13	7.77E-13
Esm1	2160	38	219.5	0.623945	3.397572	2.445014	Up	1.45E-13	5.43E-13
RGD1563955	2016	2.5	14.5	0.044232	0.240803	2.444697	Up	0.002465	0.004058
Napb	897	24.5	143.5	0.978955	5.328603	2.444443	Up	2.47E-10	6.04E-10
Blcap	2036	700.5	4082.5	12.30485	66.95619	2.44399	Up	0	0
RGD1308138	1135	4.5	26.5	0.14263	0.775947	2.443685	Up	7.99E-05	0.00015
Rhbdl1	1512	13	76	0.308798	1.678173	2.442157	Up	2.60E-07	5.69E-07
Sobp	2595	2.5	15	0.034805	0.188825	2.439698	Up	0.001428	0.002399
Chchd10	640	8	47	0.450874	2.442	2.437266	Up	5.29E-06	1.08E-05
Acsm3	2585	19	109.5	0.261014	1.413387	2.436959	Up	6.02E-09	1.41E-08
Zfp748	4998	121	701.5	0.86458	4.680912	2.436719	Up	0	0
Prr16	3138	13	76	0.14879	0.805448	2.436517	Up	2.60E-07	5.69E-07
Abcb4	3910	983	5706	8.997609	48.6795	2.435701	Up	3.73E-13	1.29E-12
Olr373	951	2.5	14.5	0.094369	0.510472	2.435449	Up	0.002465	0.004057
Zfp709l1	2600	63.5	365.5	0.869195	4.699473	2.434748	Up	0	0

Osgin2	2666	421	2430	5.634129	30.42493	2.432989	Up		4.13E-13	1.41E-12
Tcp11l2	2293	178.5	1028.5	2.77619	14.97038	2.430933	Up		1.14E-13	4.37E-13
Mettl19	3136	207.5	1197.5	2.365204	12.74795	2.430228	Up		1.16E-13	4.44E-13
Hspb9	646	5	29	0.277848	1.497396	2.430089	Up		5.00E-05	9.55E-05
Dnmt3b	4289	31.5	182.5	0.262738	1.415072	2.429176	Up		3.08E-12	8.91E-12
LOC100909952	635	3	17.5	0.169958	0.915148	2.42883	Up		0.001563	0.002615
Osr1	1726	35	203	0.727609	3.917858	2.428829	Up		3.77E-13	1.30E-12
LOC100361923	3454	3.5	20.5	0.036343	0.19568	2.428756	Up		0.000351	0.000627
Napsa	1434	4.5	25.5	0.111291	0.598406	2.42679	Up		0.000105	0.000196
Prl8a4	914	4.5	26	0.175235	0.941856	2.42622	Up		7.99E-05	0.00015
Aoc2-ps1	1459	29.5	170	0.72253	3.882767	2.425955	Up		1.04E-11	2.73E-11
LOC681383	921	2.5	14.5	0.098688	0.530031	2.425131	Up		0.002465	0.004058
Cadps	4926	6.5	37.5	0.047275	0.253889	2.42504	Up		1.71E-05	3.38E-05
Gng3	1153	13.5	77.5	0.41723	2.239833	2.424475	Up		2.60E-07	5.69E-07
LOC686013	1077	3.5	20	0.117086	0.628353	2.424009	Up		0.000351	0.000627
Cks2	640	1648	9442.5	91.81748	492.4221	2.423055	Up		8.75E-12	2.31E-11
Rhbdf2	3408	9	52	0.095003	0.509423	2.422823	Up		2.93E-06	6.07E-06
Eppk1-ps1	10275	12	69	0.042014	0.225079	2.421496	Up		4.81E-07	1.04E-06
Zfp317	4025	715.5	4089	6.335397	33.91276	2.420321	Up		0	0
Epha2	3911	3822.5	21940	34.99162	187.296	2.420239	Up		2.87E-12	8.35E-12
LOC367516	723	3	17	0.147685	0.790008	2.419344	Up		0.001563	0.002613
Nipal4	3265	9.5	54	0.103853	0.555494	2.419227	Up		2.93E-06	6.06E-06
Spag6l	1736	4	23	0.08245	0.440781	2.418475	Up		0.000226	0.000409
Il1r1	1773	616.5	3536	12.45161	66.56247	2.418377	Up		5.19E-13	1.74E-12
Vgf	2636	68.5	394	0.932377	4.978681	2.416778	Up		0	0

LOC100909649	2013	61	348	1.079498	5.762399	2.41631	Up	3.49E-14	1.41E-13
Map3k13	3451	30	172.5	0.312065	1.665075	2.415667	Up	5.60E-12	1.52E-11
Zfp763	3382	66	377	0.697634	3.718252	2.414081	Up	0	0
Prpf38b	2993	2390	13657.5	28.57762	152.3124	2.414076	Up	0	0
Siglech	2136	21	119.5	0.350996	1.869323	2.412989	Up	1.68E-09	4.01E-09
RGD1561459	876	6	34	0.243781	1.29789	2.412508	Up	2.10E-05	4.14E-05
LOC100911546	1971	16.5	93.5	0.298828	1.59042	2.412021	Up	4.01E-08	9.10E-08
Rev1	4092	1112	6338	9.711473	51.6665	2.411467	Up	4.84E-12	1.33E-11
Zfat	4444	219	1247.5	1.763266	9.372704	2.410215	Up	3.38E-13	1.18E-12
Ptbp2	1842	575.5	3277.5	11.16255	59.33068	2.410111	Up	3.54E-13	1.23E-12
Cenpk	1641	539	3064.5	11.74526	62.34493	2.408194	Up	4.59E-13	1.55E-12
Slc16a12	1876	3	17	0.056917	0.302066	2.407933	Up	0.001563	0.002614
Mgat4a	7181	57	325	0.284832	1.509402	2.405794	Up	5.37E-14	2.13E-13
Adamts15	6064	58.5	332.5	0.345914	1.831772	2.404756	Up	1.35E-14	5.59E-14
Wnt7a	3150	52	295.5	0.590343	3.125572	2.404494	Up	5.13E-14	2.04E-13
Capn13	2659	7	39	0.092908	0.491382	2.402974	Up	1.03E-05	2.07E-05
Cyb5d2	1481	38	215.5	0.920849	4.869351	2.402693	Up	1.45E-13	5.43E-13
Mefv	3231	6.5	37	0.072254	0.382053	2.402631	Up	1.71E-05	3.38E-05
Rgs16	600	91.5	519	5.465067	28.87959	2.401739	Up	0	0
Hcn1	2807	6	34	0.076079	0.401835	2.401042	Up	2.10E-05	4.14E-05
MGC105649	1174	136	766	4.12864	21.8	2.40059	Up	1.95E-13	7.10E-13
Cyb5rl	1031	13.5	76.5	0.470495	2.483842	2.400321	Up	2.60E-07	5.69E-07
RGD1562846	1469	1148	6490.5	27.96988	147.5143	2.398909	Up	3.66E-12	1.04E-11
LOC100909638	951	3	17	0.113484	0.597766	2.397095	Up	0.001563	0.002614
LOC100912411	1813	6336.5	35581.5	124.559	655.966	2.396791	Up	3.71E-12	1.05E-11

Lysmd3	1186	444	2499.5	13.37094	70.37613	2.395985	Up	1.07E-12	3.36E-12
Ptp4a1	2484	2629.5	14777.5	37.7226	198.519	2.395776	Up	0	0
Akr1d1	3189	3	17	0.033842	0.177979	2.394809	Up	0.001563	0.002614
Tmem161b	2651	541.5	3038	7.280297	38.2675	2.39405	Up	0	0
RGD1304580	1169	5	28	0.151579	0.796604	2.393794	Up	6.62E-05	0.000126
Zrsr1	1755	207	1167.5	4.226468	22.18237	2.391889	Up	1.16E-13	4.44E-13
LOC100912096	1643	47	263	1.016293	5.33392	2.391879	Up	0	0
Vom2r34	2370	6	33.5	0.089623	0.470214	2.391383	Up	2.45E-05	4.82E-05
Otud3	1528	492	2763	11.50119	60.33623	2.391242	Up	0	0
LOC100913024	619	3	17	0.174351	0.914016	2.390226	Up	0.001563	0.002615
Nvl	2901	436.5	2444.5	5.369411	28.12113	2.388819	Up	4.26E-13	1.45E-12
Acn9	336	39	218	4.141464	21.66704	2.387289	Up	1.32E-13	4.99E-13
LOC681138	476	17.5	97	1.296887	6.784707	2.387233	Up	2.14E-08	4.89E-08
LOC100125366	837	2.5	14	0.107222	0.560592	2.38635	Up	0.002465	0.004059
LOC100912748	1098	10	56	0.324328	1.694591	2.385412	Up	1.62E-06	3.40E-06
Inhbb	3131	8	44.5	0.091063	0.475236	2.383703	Up	5.48E-06	1.11E-05
LOC100911927	546	4.5	25	0.296492	1.543536	2.380173	Up	0.000105	0.000196
Fgd3	2789	3	17	0.038696	0.201246	2.378705	Up	0.001563	0.002614
Cfp	1505	43.5	242.5	1.032171	5.362559	2.37724	Up	1.18E-13	4.48E-13
LOC100364135	1944	25	138	0.458995	2.382552	2.375956	Up	1.30E-10	3.22E-10
Lrrc51	915	14.5	81.5	0.568623	2.950682	2.375503	Up	1.40E-07	3.10E-07
Pappa	4933	55	306	0.39838	2.067011	2.375327	Up	0	0
Smoc1	1359	10	55.5	0.26415	1.369771	2.374508	Up	1.62E-06	3.41E-06
Ankrd32	3750	654.5	3625.5	6.225564	32.24945	2.372998	Up	1.65E-12	5.01E-12
Ythdc1	2968	2057	11396.5	24.74735	128.1925	2.372966	Up	0	0

Megf11	3664	4	22.5	0.039378	0.203917	2.372535	Up	0.000357	0.000637
Tmprss5	2385	16	88.5	0.238613	1.234919	2.371674	Up	4.01E-08	9.09E-08
LOC100364350	4008	5	28	0.045069	0.233241	2.371616	Up	6.62E-05	0.000126
LOC100360611	483	3	16.5	0.219882	1.137748	2.371382	Up	0.00265	0.004349
LOC100365697	2464	2320.5	12814.5	33.55137	173.5411	2.370834	Up	0	0
Rbm7	1411	783.5	4341.5	19.87073	102.6853	2.369513	Up	0	0
Rnf185	2786	652	3616.5	8.384956	43.32432	2.369302	Up	1.68E-12	5.10E-12
LOC100911168	1167	8	44.5	0.246283	1.271948	2.368648	Up	5.48E-06	1.11E-05
L1cam	5093	49.5	274.5	0.348742	1.799731	2.367548	Up	3.91E-14	1.57E-13
Fbxo36	901	3.5	19	0.138684	0.715039	2.366216	Up	0.000562	0.000989
Nrip3	3859	4	22	0.037091	0.190803	2.362958	Up	0.000357	0.000637
Dnai1	2441	8	44	0.117509	0.603654	2.360952	Up	5.48E-06	1.11E-05
Slc7a13	1928	68	371	1.253736	6.425004	2.357463	Up	0	0
Htr5b	2223	98	539.5	1.581616	8.10285	2.35703	Up	0	0
LOC100912573	3311	5	27	0.053517	0.27416	2.356941	Up	9.38E-05	0.000175
Csf1	3975	9688.5	53266	87.35167	447.2459	2.356161	Up	1.88E-12	5.64E-12
Slc9a8	2160	260	1422	4.293702	21.98382	2.356148	Up	4.56E-13	1.54E-12
Atp1a4	3447	4	22	0.041857	0.214131	2.354966	Up	0.000357	0.000637
Zfp322a	3136	316.5	1723.5	3.593402	18.32236	2.350183	Up	3.85E-13	1.32E-12
LOC690276	971	14.5	78.5	0.532286	2.713507	2.349885	Up	1.40E-07	3.10E-07
RGD1311648	2738	1385.5	7543.5	18.07616	92.05792	2.348454	Up	7.00E-12	1.87E-11
RGD1306474	1265	4	22	0.114055	0.58064	2.347912	Up	0.000357	0.000637
LOC100910223	1942	9	49	0.165538	0.842407	2.347351	Up	2.97E-06	6.16E-06
LOC499602	2350	103	559	1.561539	7.946012	2.347262	Up	0	0
Sntg1	1560	11	59	0.249009	1.266745	2.346854	Up	8.87E-07	1.89E-06

Gas2	2056	17	92	0.293363	1.491336	2.345847	Up	2.14E-08	4.89E-08
Zic4	1985	7.5	41	0.135923	0.690962	2.345813	Up	9.64E-06	1.93E-05
Xkrx	1817	3.5	19	0.069085	0.3511	2.345433	Up	0.000562	0.000989
Golga1	4483	1441.5	7847.5	11.48307	58.35143	2.34526	Up	3.21E-13	1.12E-12
Cd3eap	2040	1821.5	9926.5	31.93778	162.0637	2.343225	Up	3.23E-12	9.30E-12
Greb1l	6307	3	16.5	0.01693	0.085846	2.342189	Up	0.00265	0.004347
Tex15	8416	8	43.5	0.034014	0.172407	2.341596	Up	5.64E-06	1.15E-05
Hp	1239	46.5	251	1.338095	6.780299	2.341169	Up	7.84E-14	3.07E-13
Tmem35	2006	38.5	206.5	0.678048	3.435307	2.340979	Up	1.45E-13	5.43E-13
RGD1562079	986	212.5	1156	7.709868	39.05188	2.340614	Up	0	0
LOC100361383	3155	5	27	0.056527	0.286004	2.339025	Up	9.38E-05	0.000175
Cep44	1589	233	1256.5	5.218497	26.39181	2.338384	Up	0	0
Ing4	1615	606	3288	13.43199	67.92584	2.338287	Up	6.55E-13	2.14E-12
LOC100909998	1266	10.5	57.5	0.296554	1.499677	2.338285	Up	1.62E-06	3.40E-06
Slfn1	1622	4	21.5	0.088245	0.446157	2.33797	Up	0.000579	0.001005
LOC498265	2932	390	2109	4.750319	23.99287	2.336509	Up	1.18E-13	4.48E-13
Gins2	4192	1395	7527.5	11.89032	60.02972	2.33589	Up	5.52E-12	1.50E-11
Mtpap	2680	823	4447.5	10.97667	55.41683	2.335883	Up	4.79E-13	1.61E-12
Suv39h2	4132	276.5	1493.5	2.387073	12.05047	2.335775	Up	7.04E-14	2.77E-13
Rfx8	2001	73.5	398.5	1.316239	6.631681	2.332953	Up	0	0
Slc17a9	2276	13	70.5	0.205142	1.032786	2.331849	Up	2.60E-07	5.69E-07
Ppcdc	2714	153	822.5	2.01282	10.13324	2.331805	Up	0	0
Wdr38	1186	4.5	24	0.136496	0.687002	2.331451	Up	0.00015	0.000273
Lipogenin	1265	6	32.5	0.170629	0.85812	2.330312	Up	3.05E-05	5.93E-05
Zfp157	3225	224	1200.5	2.474563	12.43055	2.328644	Up	7.97E-14	3.12E-13

RGD1306576	797	152	813	6.796346	34.08078	2.326127	Up	1.08E-13	4.15E-13
Arl5b	2827	345.5	1853.5	4.36217	21.86831	2.325724	Up	9.33E-13	2.96E-12
LOC100365935	2893	174.5	938	2.159464	10.82255	2.325295	Up	1.43E-13	5.33E-13
Adcyap1r1	6235	6	32	0.034251	0.17164	2.325188	Up	3.05E-05	5.93E-05
LOC367196	1950	266.5	1429.5	4.880524	24.4481	2.324614	Up	2.19E-13	7.92E-13
Nrcam	7556	18	97	0.085243	0.426578	2.32315	Up	1.14E-08	2.63E-08
Slc10a2	377	351	1876.5	33.25154	166.3568	2.322788	Up	9.48E-14	3.68E-13
Atp6v1e2	992	3	16	0.107637	0.538497	2.322759	Up	0.00265	0.004349
Dus4l	1821	138.5	742	2.723931	13.62335	2.322319	Up	1.83E-13	6.72E-13
LOC100911038	540	14	74.5	0.921342	4.601977	2.320445	Up	1.40E-07	3.10E-07
RGD1564214	1104	49.5	265.5	1.610904	8.043432	2.319941	Up	3.91E-14	1.57E-13
Klf15	2458	22	116	0.31724	1.581477	2.317625	Up	8.87E-10	2.13E-09
RGD1565170	495	4.5	24	0.325881	1.624209	2.317319	Up	0.00015	0.000273
Slc17a4	2461	3	16	0.043853	0.218525	2.317036	Up	0.00265	0.004348
LOC100302465	2943	240	1280.5	2.914985	14.52523	2.317001	Up	0	0
Mrgpre	930	3	16	0.11543	0.574397	2.315032	Up	0.00265	0.004349
Rabl2a	1830	161.5	859	3.150869	15.65452	2.312758	Up	0	0
Jph3	3606	14	75	0.139084	0.690907	2.31253	Up	1.40E-07	3.10E-07
Guca1b	1677	14.5	77	0.308541	1.53243	2.312287	Up	1.40E-07	3.10E-07
Zfp672	2947	293	1563.5	3.564686	17.70371	2.312205	Up	5.51E-13	1.83E-12
LOC690136	4032	181	956	1.597096	7.92036	2.310115	Up	1.41E-13	5.30E-13
Osmr	2889	16	85	0.198375	0.982616	2.308399	Up	4.01E-08	9.09E-08
LOC687056	1308	15.5	81.5	0.418995	2.074446	2.307721	Up	7.51E-08	1.68E-07
Ccnl2	1171	482.5	2542.5	14.63569	72.42905	2.307078	Up	1.31E-14	5.42E-14
Cwc25	3195	448	2371	5.009236	24.78724	2.306935	Up	0	0

Nedd9	4393	2052.5	10900.5	16.73348	82.8005	2.306902	Up	0	0
Gtpbp8	1225	161.5	854.5	4.708416	23.27321	2.305356	Up	0	0
Mtf2	2575	593.5	3138	8.233596	40.69038	2.305093	Up	1.48E-12	4.54E-12
Fbxo28	1523	583.5	3081	13.66748	67.47883	2.303687	Up	5.08E-13	1.70E-12
Serpinb5	2576	8.5	45.5	0.11863	0.585695	2.303679	Up	5.38E-06	1.09E-05
Zbtb10	3849	312	1647.5	2.895226	14.28611	2.302865	Up	1.77E-13	6.50E-13
Ccr7	1215	8	42.5	0.234666	1.157851	2.30277	Up	5.93E-06	1.20E-05
Zscan12	2344	186	977	2.826978	13.92475	2.300319	Up	6.10E-13	2.00E-12
Hoxa5	1971	37.5	198	0.679207	3.34525	2.30019	Up	1.86E-13	6.81E-13
Slc9b1	2254	143	751	2.259757	11.1264	2.299747	Up	0	0
Lat	1188	7	36.5	0.208913	1.028425	2.299461	Up	1.46E-05	2.90E-05
Fam13c	3535	11	58	0.111835	0.549826	2.297607	Up	8.89E-07	1.89E-06
Rasgrp1	3624	3	16	0.02978	0.14641	2.29759	Up	0.00265	0.004347
Gyltl1b	2455	7	37	0.102263	0.502449	2.29669	Up	1.24E-05	2.48E-05
Cmah	2221	10	52.5	0.161113	0.791426	2.296381	Up	1.65E-06	3.46E-06
LOC100909521	1851	52	274.5	1.007734	4.948044	2.295743	Up	5.13E-14	2.04E-13
LOC100363112	670	21.5	112	1.140137	5.589139	2.293419	Up	1.68E-09	4.01E-09
Zbtb6	2418	419.5	2200.5	6.1956	30.36523	2.293104	Up	0	0
Gpr68	3115	18	94	0.206037	1.005188	2.286492	Up	1.14E-08	2.63E-08
LOC100912790	701	17	89	0.8686	4.234987	2.285594	Up	2.14E-08	4.89E-08
Cst6	836	15.5	81.5	0.66859	3.258583	2.285052	Up	7.51E-08	1.68E-07
Slc27a3	2274	13	68	0.205574	1.001541	2.28449	Up	2.61E-07	5.69E-07
Senp8	1007	71	370	2.526935	12.30211	2.283445	Up	0	0
LOC500475	2155	17	87.5	0.278821	1.355822	2.281754	Up	2.14E-08	4.89E-08
LOC100363065	3068	156	812	1.820982	8.836112	2.278695	Up	1.64E-13	6.07E-13

LOC100911686	707	5	26	0.252253	1.223984	2.278643	Up	0.000141	0.000257
RGD1560191	3737	403	2093	3.855967	18.70032	2.277898	Up	0	0
Lats2	4958	2429.5	12611	17.52274	84.89868	2.276514	Up	0	0
Camk2a	1510	18	94.5	0.428074	2.073644	2.276236	Up	1.14E-08	2.63E-08
Irak2	2977	121	628.5	1.453252	7.035727	2.275415	Up	0	0
LOC687866	3108	2.5	13	0.028691	0.13878	2.274134	Up	0.004296	0.006927
Actr6	1707	533.5	2755	11.14208	53.87649	2.273637	Up	0	0
Dennd2c	4980	54.5	281.5	0.390855	1.889305	2.273149	Up	3.66E-14	1.48E-13
LOC100362641	3123	173	892.5	1.978005	9.554781	2.272177	Up	3.79E-13	1.30E-12
LOC100912109	375	7	36.5	0.675599	3.25805	2.269769	Up	1.46E-05	2.90E-05
Tnfsf15	759	29	149.5	1.364945	6.579155	2.269059	Up	1.04E-11	2.73E-11
Slc26a9	3580	17	88	0.170401	0.821184	2.268773	Up	2.14E-08	4.89E-08
LOC691960	2020	8	41	0.140012	0.674534	2.268336	Up	6.40E-06	1.30E-05
RGD1305938	4512	584.5	3009	4.618898	22.23455	2.267183	Up	5.51E-13	1.83E-12
Rad9b	1783	78	400.5	1.560245	7.508227	2.266699	Up	0	0
Traf2	2098	773.5	3989.5	13.19395	63.49196	2.266697	Up	9.31E-13	2.96E-12
Cntf	1101	54	276	1.743578	8.383343	2.265475	Up	3.66E-14	1.48E-13
Txk	2308	19.5	100	0.302452	1.453588	2.264839	Up	6.02E-09	1.41E-08
Ica1l	1308	2.5	13	0.069489	0.33389	2.264517	Up	0.004296	0.006926
Rasl11b	1840	281.5	1451	5.482081	26.33036	2.263932	Up	0	0
Cdc42bpg	5967	6	31	0.035981	0.172698	2.262942	Up	4.05E-05	7.83E-05
LOC688416	465	2.5	13	0.191766	0.91791	2.259003	Up	0.004296	0.006931
LOC691141	1301	6	31	0.165908	0.792768	2.256515	Up	4.05E-05	7.83E-05
Fam133b	2009	1583	8083	28.11734	134.3436	2.256395	Up	4.71E-12	1.30E-11
Mtcp1	538	69	353.5	4.598892	21.97154	2.256278	Up	0	0

RGD1566244	921	2.5	13	0.09682	0.462463	2.255958	Up	0.004296	0.00693
Lce1m	829	2.5	13	0.108257	0.517043	2.255827	Up	0.004296	0.006928
LOC100912922	524	7038.5	36117	480.8097	2294.079	2.254377	Up	3.35E-11	8.47E-11
Tgds	1797	466	2373	9.25669	44.10696	2.252438	Up	4.74E-13	1.60E-12
LOC690857	1092	48.5	249	1.600039	7.623642	2.252374	Up	1.06E-13	4.07E-13
Zfp638	6336	1971.5	10037	11.10643	52.87772	2.251265	Up	9.95E-13	3.14E-12
Vgll3	1176	315.5	1612.5	9.63146	45.83613	2.250659	Up	4.99E-13	1.67E-12
Lrrc39	2875	54.5	277.5	0.680021	3.235858	2.250497	Up	3.66E-14	1.48E-13
Gipr	2698	8	41	0.106528	0.50636	2.24893	Up	6.40E-06	1.30E-05
LOC365791	4770	9.5	48	0.070846	0.336723	2.24881	Up	3.01E-06	6.23E-06
Styk1	1290	30.5	155	0.842259	4.002524	2.248574	Up	5.60E-12	1.52E-11
Exog	4085	269.5	1372	2.360379	11.21626	2.248501	Up	5.77E-14	2.28E-13
Nmb	660	5.5	28	0.29689	1.409592	2.247275	Up	6.62E-05	0.000126
LOC365218	1254	6.5	34	0.187994	0.892305	2.246848	Up	2.10E-05	4.14E-05
Rasgrf1	1395	52	265.5	1.340432	6.357168	2.245686	Up	5.13E-14	2.04E-13
LOC100361503	3102	243	1236	2.808127	13.30799	2.244613	Up	0	0
Map4k1	2699	44.5	226	0.588811	2.7903	2.244544	Up	8.13E-14	3.18E-13
RGD1309170	1580	3	15	0.066854	0.316394	2.242634	Up	0.004499	0.007237
Fam221a	1427	9	45.5	0.224477	1.061703	2.24174	Up	3.36E-06	6.93E-06
Gas5	429	997.5	5021	82.62149	390.6972	2.241462	Up	0	0
Rad18	1737	765	3866.5	15.73437	74.28443	2.239141	Up	0	0
LOC100912030	972	239	1210.5	8.799142	41.53219	2.238795	Up	5.84E-13	1.93E-12
Zfp868	2262	366	1844.5	5.776678	27.22477	2.236608	Up	0	0
Il34	1549	7.5	38	0.174922	0.823692	2.235391	Up	1.11E-05	2.22E-05
Dnajb4	1508	798.5	4025.5	18.93672	89.11271	2.234445	Up	1.74E-12	5.25E-12

Ankrd16	1378	48	242.5	1.248937	5.873768	2.233585	Up	1.06E-13	4.07E-13
LOC100909526	2556	55	275.5	0.763926	3.591399	2.233041	Up	0	0
RGD1565744	1424	308.5	1557	7.756827	36.46528	2.232985	Up	0	0
Ankrd55	2977	31	157	0.373195	1.753184	2.231977	Up	3.08E-12	8.90E-12
LOC685925	1672	121.5	609	2.58673	12.14825	2.231547	Up	0	0
RGD1359529	1515	652	3283	15.39372	72.28376	2.231329	Up	1.68E-12	5.10E-12
Rnpc3	1818	561.5	2824.5	11.05139	51.8918	2.231279	Up	0	0
Mmp1b	1383	5	25	0.129783	0.609379	2.23124	Up	0.00022	0.000398
Alkbh4	1803	87.5	439.5	1.735455	8.147618	2.231064	Up	0	0
Jam2	4225	11	55	0.092756	0.43526	2.230362	Up	9.06E-07	1.93E-06
Ppm1d	2909	412	2069.5	5.062439	23.74608	2.229785	Up	2.14E-13	7.77E-13
Itgb6	2818	75.5	380	0.959011	4.495099	2.228733	Up	0	0
Scn2b	873	5	25.5	0.205601	0.962329	2.226684	Up	0.00022	0.000399
LOC100911466	1816	85.5	427.5	1.681097	7.859753	2.225081	Up	0	0
Ano7	2742	8	39.5	0.103564	0.484073	2.224705	Up	8.54E-06	1.72E-05
RGD1559502	1490	62	311	1.488582	6.955972	2.224313	Up	0	0
LOC499110	4857	263.5	1318	1.938523	9.056911	2.224061	Up	1.80E-13	6.60E-13
Mtif2	2259	249	1242.5	3.926719	18.3354	2.223235	Up	1.13E-14	4.69E-14
Rbm41	2732	229	1141.5	2.992053	13.96091	2.222185	Up	2.54E-13	9.10E-13
LOC100911674	758	61.5	308	2.903635	13.54121	2.221425	Up	3.49E-14	1.41E-13
LOC680222	1672	79	394.5	1.687971	7.866954	2.220515	Up	0	0
Epc2	3686	978	4883.5	9.492903	44.19328	2.218906	Up	2.73E-12	7.98E-12
Ccdc8	3408	9.5	46.5	0.098654	0.459107	2.218379	Up	3.18E-06	6.58E-06
RGD1309708	1918	832	4143	15.50998	72.16453	2.218093	Up	0	0
Lrrtm2	4715	18	90.5	0.136606	0.635579	2.218049	Up	1.14E-08	2.63E-08

Disc1	2475	8	40	0.1152	0.535948	2.217958	Up	7.20E-06	1.46E-05
Zfp280d	4256	1129	5630	9.485977	44.12623	2.217768	Up	3.41E-12	9.77E-12
Cd180	2566	3	15	0.042059	0.195519	2.216827	Up	0.004499	0.007236
Zfp202	3481	140	695.5	1.43387	6.660953	2.215814	Up	1.60E-14	6.57E-14
Pou2f1	2566	118	589	1.649102	7.660106	2.215683	Up	0	0
Il17rb	2056	3.5	17.5	0.060776	0.282208	2.215194	Up	0.001563	0.002614
Kdm6a	6059	929	4614.5	5.473521	25.40975	2.214841	Up	1.35E-12	4.16E-12
Aplnr	3670	42	207.5	0.406383	1.886325	2.214665	Up	1.10E-13	4.23E-13
Samd10	1316	39	195.5	1.063496	4.934856	2.214194	Up	1.32E-13	4.99E-13
Zfp281	3114	1019	5060.5	11.70168	54.28559	2.213854	Up	1.08E-12	3.38E-12
Nf1	9132	629	3119	2.458485	11.39762	2.212891	Up	1.00E-12	3.16E-12
Adm	1395	1540	7659.5	39.54991	183.3425	2.212795	Up	1.19E-12	3.68E-12
Zfp37	3369	361.5	1791.5	3.833906	17.76406	2.212075	Up	0	0
Ybey	4419	150.5	746	1.217324	5.639299	2.211802	Up	7.11E-15	2.97E-14
LOC654482	1344	58.5	290.5	1.556889	7.210728	2.211479	Up	1.35E-14	5.59E-14
Tex12	372	5	25	0.482499	2.23406	2.21107	Up	0.00022	0.000398
Daam1	5722	1827	9032	11.41545	52.70839	2.207045	Up	2.38E-12	7.04E-12
Harbi1	1460	203	1004	4.969448	22.93696	2.206516	Up	1.92E-13	7.01E-13
Vav1	2917	15.5	76	0.18788	0.867089	2.206369	Up	7.52E-08	1.68E-07
LOC100913004	2186	117	578	1.914418	8.831097	2.205687	Up	1.22E-13	4.64E-13
Btbd11	5629	50	248	0.318458	1.468063	2.204737	Up	3.62E-14	1.46E-13
Zfp949	3074	86	422	0.995496	4.58804	2.20439	Up	0	0
Med12l	3985	153.5	758.5	1.380581	6.359661	2.203674	Up	0	0
RGD1565007	3423	4	20	0.04215	0.194022	2.20261	Up	0.000956	0.001628
Gbp5	2810	177.5	875	2.25941	10.38792	2.200889	Up	0	0

Map3k14	4206	443.5	2183.5	3.773217	17.32823	2.199258	Up	0	0
LOC100909537	826	11	54.5	0.480003	2.203428	2.198633	Up	9.20E-07	1.96E-06
Asb14	2008	6.5	31.5	0.113976	0.523075	2.198288	Up	4.05E-05	7.83E-05
Zfp35	2807	371	1818	4.715563	21.61999	2.196864	Up	5.35E-13	1.78E-12
LOC100361928	2487	28	137.5	0.403328	1.848949	2.196679	Up	1.93E-11	4.98E-11
Mxd1	2633	155	763	2.110197	9.67358	2.196672	Up	3.73E-13	1.29E-12
LOC100912751	651	15	73.5	0.821856	3.767405	2.196614	Up	7.53E-08	1.68E-07
Il1rl1	2065	1391	6809.5	24.02694	110.1133	2.196264	Up	7.32E-12	1.95E-11
Gabrb1	1944	4	19.5	0.073038	0.334667	2.196006	Up	0.00159	0.002657
Iah1	1036	74.5	362	2.549691	11.67821	2.195425	Up	9.99E-15	4.15E-14
Lcor	4732	214.5	1048	1.614711	7.391542	2.1946	Up	0	0
Hes1	1436	1559	7638	38.76317	177.308	2.193499	Up	7.56E-12	2.01E-11
LOC100909474	1564	106	522	2.434739	11.13166	2.19283	Up	0	0
Tmem128	1104	629.5	3059.5	20.22827	92.48324	2.192819	Up	1.00E-12	3.16E-12
Nr3c1	6327	3273.5	15994	18.46674	84.32969	2.191111	Up	0	0
Ssu72	1063	909	4452	30.63805	139.7307	2.189252	Up	2.43E-12	7.16E-12
Zxdb	2313	182	890	2.817612	12.83807	2.187884	Up	0	0
Ifngr2	2610	247.5	1211	3.396863	15.47407	2.187578	Up	0	0
LOC691254	1443	13	62.5	0.31959	1.455112	2.186837	Up	2.65E-07	5.80E-07
Pan2	3618	463	2260.5	4.584139	20.86518	2.186375	Up	1.22E-12	3.78E-12
Odf2l	2396	177.5	861.5	2.639997	12.01519	2.186251	Up	0	0
Smndc1	1843	1482.5	7235	28.78743	130.9927	2.185975	Up	3.37E-12	9.65E-12
LOC691849	737	57.5	281.5	2.794495	12.70643	2.184899	Up	5.37E-14	2.13E-13
Gjb3	813	53.5	262	2.364261	10.74827	2.184643	Up	4.04E-14	1.62E-13
Ccdc73	3201	10	49	0.111071	0.504609	2.183682	Up	1.78E-06	3.72E-06

RGD1560065	1472	226.5	1097.5	5.476246	24.87097	2.183204	Up	3.18E-14	1.29E-13
LOC100911496	2915	25	122.5	0.308069	1.398571	2.182627	Up	1.30E-10	3.22E-10
Fam212a	1062	20	98	0.679283	3.083435	2.182453	Up	3.18E-09	7.52E-09
LOC100912181	937	20.5	99	0.776452	3.522726	2.181724	Up	3.18E-09	7.52E-09
Zfp53	2536	72	349.5	1.015928	4.607075	2.181053	Up	0	0
LOC686240	3820	627.5	3038.5	5.858265	26.56015	2.180718	Up	6.79E-13	2.21E-12
Gria3	5052	768	3712.5	5.421003	24.53357	2.178125	Up	0	0
Orc4	1755	809.5	3910	16.43179	74.36161	2.178068	Up	1.03E-12	3.23E-12
Luc7l3	3331	2237	10823.5	23.97293	108.4828	2.177988	Up	0	0
Jmjd1c	8363	2577.5	12472.5	11.01584	49.79617	2.176455	Up	0	0
LOC303448	3174	32.5	157.5	0.365407	1.651756	2.176425	Up	1.74E-12	5.25E-12
Txlng	3067	184.5	892	2.146892	9.701055	2.175892	Up	3.15E-13	1.11E-12
LOC100363155	1248	12	58	0.343151	1.550188	2.17553	Up	4.95E-07	1.07E-06
Itpka	1831	6	29	0.117258	0.5283	2.171671	Up	8.58E-05	0.000161
LOC681908	1923	62.5	301	1.160467	5.226381	2.171107	Up	0	0
Mybl1	3643	2633.5	12696	25.82488	116.2921	2.17092	Up	0	0
Mrpl1	1207	295	1412.5	8.685358	39.08792	2.170066	Up	3.74E-13	1.29E-12
Crnn	1578	12	57	0.269935	1.214543	2.169727	Up	5.04E-07	1.09E-06
Ninj2	907	28	135.5	1.108458	4.98728	2.169699	Up	1.93E-11	4.98E-11
Btbd10	2300	1281.5	6169.5	19.92315	89.57352	2.168627	Up	4.10E-12	1.15E-11
Pkp3	2955	23.5	112.5	0.284279	1.277925	2.168421	Up	4.68E-10	1.13E-09
LOC682431	1846	122	587	2.364872	10.60675	2.165148	Up	4.93E-14	1.96E-13
Vtn	1628	4	19.5	0.088624	0.397416	2.16488	Up	0.00159	0.002657
RGD1308147	2035	285	1369.5	5.008875	22.45051	2.16419	Up	6.22E-13	2.04E-12
LOC100361765	1477	5	24	0.11997	0.537633	2.163945	Up	0.000353	0.00063

Bcdin3d	1254	60.5	288.5	1.718382	7.69581	2.163023	Up	0	0
Nuak2	2900	900.5	4325.5	11.12545	49.77317	2.161505	Up	6.71E-13	2.18E-12
Zmynd10	1754	44.5	213.5	0.908332	4.063646	2.161483	Up	8.13E-14	3.18E-13
Tmem68	2664	681	3239	9.074319	40.58021	2.160915	Up	1.16E-13	4.45E-13
Taf9b	2477	181	871	2.615919	11.69746	2.160805	Up	1.41E-13	5.30E-13
Tmem67	4035	296.5	1418.5	2.625801	11.74033	2.160644	Up	3.73E-13	1.29E-12
Cep112	3450	83	397	0.859708	3.842424	2.160097	Up	1.43E-13	5.34E-13
Prr5l	2466	321	1532.5	4.645823	20.74373	2.158669	Up	0	0
Abca12	8344	6	29	0.025868	0.115498	2.158606	Up	8.58E-05	0.00016
LOC682225	4765	169.5	808.5	1.27041	5.668959	2.15779	Up	8.88E-15	3.70E-14
Prss22	1271	42	199.5	1.176135	5.246514	2.157305	Up	1.10E-13	4.23E-13
LOC100361568	1087	218	1037	7.146856	31.86019	2.156374	Up	0	0
Pex11g	1152	30	143	0.929864	4.145151	2.156333	Up	5.60E-12	1.52E-11
Zfp968	3308	16	76.5	0.172381	0.767334	2.15425	Up	4.02E-08	9.11E-08
Pex12	2347	54.5	259.5	0.829095	3.69051	2.154211	Up	3.66E-14	1.48E-13
LOC684233	4674	577	2755.5	4.419608	19.66902	2.153935	Up	5.81E-13	1.92E-12
Pcbd1	744	17	81	0.816857	3.633645	2.153262	Up	2.14E-08	4.89E-08
LOC100363406	717	115	548	5.748089	25.56259	2.15288	Up	0	0
Ptpn7	2032	16.5	79	0.292397	1.300226	2.152762	Up	4.02E-08	9.10E-08
Cbwd1	1405	196	932	4.978356	22.12072	2.151657	Up	1.28E-13	4.85E-13
LOC684258	625	1171	5569.5	66.93795	297.1126	2.150114	Up	3.90E-12	1.10E-11
Rassf1	1148	914	4353.5	28.49543	126.4601	2.14988	Up	0	0
Rab32	2069	1116.5	5291	19.23622	85.36183	2.149766	Up	3.19E-12	9.19E-12
Fdx1	838	437	2079	18.6267	82.6094	2.148934	Up	8.15E-13	2.61E-12
RGD1310552	4329	154.5	733.5	1.275829	5.655458	2.148209	Up	2.09E-13	7.59E-13

LOC687516	2534	1213.5	5754	17.10931	75.7506	2.146475	Up	5.51E-12	1.50E-11
LOC100912612	826	12	57	0.521936	2.31047	2.146242	Up	5.04E-07	1.09E-06
Adrb2	2065	91.5	432	1.579304	6.986297	2.145239	Up	0	0
LOC500227	1949	7.5	36	0.139022	0.614841	2.144896	Up	1.46E-05	2.90E-05
Wdr20a	4155	525	2492.5	4.523831	20.0063	2.144837	Up	4.15E-14	1.66E-13
Sp6	1995	7	33	0.125843	0.556323	2.144303	Up	3.46E-05	6.71E-05
RGD1560860	1431	4	19	0.100424	0.44392	2.144199	Up	0.00159	0.002657
Tgfb2	2880	1733.5	8201	21.49736	95.00564	2.143854	Up	2.65E-12	7.76E-12
LOC685655	1622	119	561	2.617856	11.56135	2.142852	Up	0	0
LOC100158225	1994	504.5	2387.5	9.050461	39.95038	2.142146	Up	1.66E-13	6.14E-13
Slc9a2	3942	14.5	68	0.12995	0.573415	2.141625	Up	1.41E-07	3.12E-07
LOC100910178	4016	37.5	176	0.332775	1.468098	2.14133	Up	1.86E-13	6.81E-13
Lrif1	3176	452	2128.5	5.075785	22.39042	2.141179	Up	6.60E-13	2.15E-12
Il13ra2	1938	10	47.5	0.18464	0.813892	2.140123	Up	2.07E-06	4.32E-06
Ccdc18	4389	164	772	1.331071	5.865577	2.139685	Up	1.50E-13	5.56E-13
Myc	2355	3202.5	15160.5	48.79809	214.8288	2.138291	Up	0	0
RGD1565883	1807	18	85	0.357081	1.571986	2.138266	Up	1.14E-08	2.63E-08
Kif5a	3485	6	28.5	0.061936	0.272647	2.138188	Up	0.000133	0.000244
Ranbp6	4522	1051.5	4938	8.282056	36.45026	2.137868	Up	1.50E-12	4.60E-12
Ppp1r14a	559	5.5	25.5	0.348481	1.533478	2.137656	Up	0.00022	0.000399
LOC686087	579	22.5	104	1.372218	6.034247	2.136664	Up	8.87E-10	2.13E-09
LOC100912806	1514	58	272	1.364764	5.999942	2.136297	Up	1.35E-14	5.60E-14
Optc	1253	13	60.5	0.368966	1.618877	2.133432	Up	2.74E-07	5.99E-07
Syt2	2681	4	19	0.053602	0.234931	2.131883	Up	0.00159	0.002658
Arl4a	1067	690.5	3222.5	23.00929	100.8091	2.131338	Up	2.44E-15	1.03E-14

Tspan2	3854	184	862.5	1.704814	7.466729	2.130862	Up	3.15E-13	1.11E-12
LOC100361574	486	547.5	2540.5	39.87066	174.6001	2.130655	Up	0	0
LOC100363372	1980	39	182	0.701636	3.072525	2.130631	Up	1.32E-13	4.99E-13
Polh	3179	556.5	2611	6.258354	27.4048	2.130574	Up	8.86E-14	3.44E-13
Unc13b	6599	116	544.5	0.629534	2.756125	2.130285	Up	9.68E-14	3.75E-13
Zfp39	4013	150	703.5	1.336238	5.848734	2.129947	Up	7.11E-15	2.97E-14
Kif24	4077	286.5	1340.5	2.516467	10.98748	2.126389	Up	3.49E-14	1.41E-13
Rbak	2924	76	355.5	0.929678	4.057477	2.12578	Up	0	0
Slamf8	1600	37	173.5	0.828562	3.616163	2.125778	Up	1.86E-13	6.81E-13
Tial1	1680	2040.5	9516.5	43.3711	189.045	2.123924	Up	0	0
Gpcpd1	3229	291	1352	3.209363	13.98788	2.123819	Up	1.98E-13	7.23E-13
LOC689826	510	3.5	16.5	0.247258	1.077514	2.123619	Up	0.00265	0.004347
LOC100909688	2554	53	248	0.743239	3.23666	2.122608	Up	4.04E-14	1.62E-13
Kcnh2	3889	24	111.5	0.220827	0.96127	2.122023	Up	2.47E-10	6.03E-10
Chd7	9304	201.5	937.5	0.773398	3.364304	2.121024	Up	0	0
Chrna5	3007	12	56	0.1428	0.621171	2.120997	Up	5.19E-07	1.12E-06
RGD1565367	1989	24.5	113.5	0.439183	1.910384	2.120968	Up	2.47E-10	6.03E-10
Plac9	410	17	79	1.478101	6.428678	2.120778	Up	2.14E-08	4.90E-08
Btn2a2	1572	105	486	2.378793	10.32359	2.117644	Up	0	0
Higd1a	1443	3067.5	14216	75.81194	328.8388	2.116884	Up	0	0
Zfp18	2192	322.5	1497.5	5.260848	22.81469	2.116596	Up	3.42E-13	1.19E-12
LOC100912964	1029	14.5	67	0.500612	2.170375	2.11618	Up	1.41E-07	3.13E-07
LOC100911441	835	7.5	34.5	0.317629	1.37676	2.115862	Up	2.44E-05	4.78E-05
Spint1	2321	4	18.5	0.061669	0.267086	2.114694	Up	0.002645	0.004342
Plch1	5623	52	242	0.331118	1.434005	2.114633	Up	5.13E-14	2.04E-13

Slc44a3	2302	8	37	0.123857	0.536235	2.114188	Up	1.45E-05	2.88E-05
Zcchc10	1170	232	1076.5	7.083716	30.65756	2.113665	Up	3.80E-13	1.31E-12
LOC100362927	366	5.5	25.5	0.535376	2.315064	2.112428	Up	0.00022	0.000398
Cd274	873	30	139	1.230978	5.318977	2.111344	Up	5.60E-12	1.52E-11
Fam171b	3127	267	1235	3.051146	13.17193	2.110043	Up	7.08E-13	2.29E-12
Cyp2j3	3678	67.5	311.5	0.65429	2.824563	2.110025	Up	0	0
11-Mar	1572	4.5	21	0.103345	0.446007	2.1096	Up	0.000579	0.001005
Dcun1d1	1703	449.5	2075.5	9.440631	40.73951	2.109473	Up	1.57E-12	4.80E-12
Kcnk5	3489	3.5	16.5	0.036143	0.155956	2.109372	Up	0.00265	0.004346
Epc2l1	2427	369.5	1704.5	5.441105	23.44094	2.107059	Up	0	0
Hist2h2ac	506	7	32.5	0.496158	2.136406	2.106313	Up	5.16E-05	9.84E-05
Cpt1b	2826	78	358.5	0.986836	4.247319	2.10567	Up	0	0
Pnrc1	1619	837	3868.5	18.50474	79.60477	2.10496	Up	8.45E-13	2.70E-12
Rbm4	1444	426.5	1970.5	10.57073	45.45752	2.104444	Up	5.03E-13	1.69E-12
Mettl7b	1317	3.5	16	0.094443	0.40561	2.102582	Up	0.00265	0.004348
Toag1	1789	173.5	798	3.470153	14.90004	2.102245	Up	3.79E-13	1.30E-12
Amotl2	4141	5557	25618	48.06266	206.3302	2.101966	Up	1.55E-11	4.03E-11
LOC100911566	1461	52.5	240.5	1.281724	5.501149	2.101647	Up	5.13E-14	2.04E-13
RGD1310794	1910	249.5	1145	4.65884	19.99387	2.101515	Up	1.13E-14	4.70E-14
LOC100911574	338	7	32	0.735983	3.155556	2.100149	Up	5.16E-05	9.84E-05
Kctd19	2965	31	142	0.373931	1.601392	2.098481	Up	3.08E-12	8.90E-12
Timm9	801	415.5	1900.5	18.48046	79.1408	2.098421	Up	0	0
Tmprss6	2999	23	106.5	0.27615	1.181876	2.097552	Up	4.68E-10	1.13E-09
LOC686999	1067	457	2089.5	15.28632	65.37403	2.096477	Up	1.73E-12	5.23E-12
RGD1309313	2064	10	45.5	0.172257	0.736652	2.09642	Up	2.86E-06	5.94E-06

RGD1561241	4941	192.5	878	1.386039	5.91834	2.094225	Up	0	0
Mettl16	2747	520.5	2376.5	6.768392	28.88886	2.093628	Up	1.11E-12	3.45E-12
Hfe2	2150	3.5	16	0.057852	0.246786	2.092826	Up	0.00265	0.004346
Oit3	2289	59.5	272	0.929519	3.964575	2.09261	Up	2.53E-14	1.03E-13
Itga8	5896	85	387.5	0.514898	2.193579	2.090927	Up	0	0
Kdm6b	4679	1524	6982.5	11.69187	49.80632	2.090823	Up	3.96E-12	1.11E-11
Usp49	5363	93	424.5	0.619663	2.639409	2.090659	Up	3.69E-14	1.49E-13
Slc28a2	2881	10	46	0.124204	0.528703	2.089743	Up	2.37E-06	4.93E-06
Ppef1	2497	92	419.5	1.318175	5.610299	2.089535	Up	6.66E-15	2.78E-14
Gpr15	1939	6	27.5	0.111318	0.473743	2.089413	Up	0.000211	0.000383
Slc16a10	2540	4.5	20.5	0.063283	0.269283	2.089244	Up	0.000956	0.001629
Spag8	1592	8	37	0.181256	0.770862	2.088443	Up	1.45E-05	2.88E-05
Ell	3069	810.5	3700.5	9.477832	40.273	2.087184	Up	0	0
LOC100909847	937	11	49.5	0.418245	1.776732	2.086807	Up	1.34E-06	2.84E-06
LOC689963	1247	3.5	16	0.101124	0.429101	2.085194	Up	0.00265	0.004348
Papolg	3725	342.5	1556	3.288828	13.95449	2.085084	Up	2.29E-13	8.24E-13
LOC100361514	1425	5.5	24.5	0.136702	0.578756	2.081918	Up	0.000353	0.00063
Icam5	3117	28	127	0.322544	1.365231	2.081575	Up	1.93E-11	4.98E-11
Col11a2	5951	194.5	882	1.168512	4.94313	2.080752	Up	5.28E-13	1.76E-12
Tbc1d10c	1719	5	23	0.103748	0.438858	2.08067	Up	0.000574	0.001009
Tesc	909	8	36	0.312401	1.32027	2.079364	Up	2.06E-05	4.06E-05
Trim69	1632	9.5	43	0.20777	0.875817	2.075642	Up	4.14E-06	8.51E-06
RGD1561444	2213	70	314.5	1.125132	4.741302	2.075189	Up	0	0
Shank1	7585	4	18.5	0.018871	0.079473	2.074337	Up	0.002645	0.004342
Tubd1	1847	148.5	669	2.87001	12.08667	2.074289	Up	0	0

Dcaf13	1526	1130.5	5094.5	26.47062	111.4628	2.074099	Up	2.28E-12	6.74E-12
Casp4	1350	286.5	1288	7.569148	31.86683	2.073852	Up	3.49E-14	1.41E-13
Myo16	6843	6.5	29.5	0.034283	0.144259	2.073094	Up	8.58E-05	0.000161
Fam18b2	1677	1185.5	5314.5	25.15849	105.7935	2.072134	Up	2.13E-12	6.34E-12
Kctd9	3106	1289	5788	14.79015	62.17716	2.071748	Up	5.50E-12	1.50E-11
LOC100912012	980	8	36	0.293278	1.231965	2.070619	Up	2.06E-05	4.06E-05
Eif4a2	1912	4611	20662.5	85.91464	360.6317	2.06955	Up	2.21E-11	5.66E-11
Ier3	1114	8621	38795.5	277.2989	1161.712	2.066738	Up	0	0
Zfp184	2595	124	555.5	1.710978	7.156503	2.064433	Up	1.27E-13	4.83E-13
Chic1	1561	34	151	0.774983	3.241133	2.064262	Up	5.99E-13	1.97E-12
Riok3	3843	3253	14543	30.23303	126.2836	2.062469	Up	0	0
Magee1	3548	7	31.5	0.071083	0.296289	2.059428	Up	7.95E-05	0.000149
Tf	2323	64	284.5	0.980912	4.088333	2.059317	Up	0	0
Ip6k2	1894	530.5	2361.5	10.00136	41.67361	2.058938	Up	0	0
Fat3	14492	6.5	29.5	0.016149	0.067248	2.058092	Up	8.58E-05	0.000161
Serpinb2	1942	449	1998	8.253772	34.36022	2.057614	Up	1.57E-12	4.80E-12
Tec	2646	89	396	1.201209	4.999387	2.057264	Up	0	0
Tmprss11d	1683	121.5	544.5	2.589244	10.77514	2.057104	Up	0	0
Fzd8	3012	126	563	1.499574	6.234952	2.055826	Up	1.12E-13	4.31E-13
Taf15	1494	3276	14552	78.29901	325.2691	2.054568	Up	0	0
Vwa3a	4100	25.5	113	0.221785	0.921271	2.054463	Up	1.30E-10	3.22E-10
LOC100909523	2463	16	71	0.231987	0.963526	2.054277	Up	4.13E-08	9.35E-08
RGD1565787	734	62.5	277.5	3.049672	12.65489	2.052969	Up	0	0
Nr4a3	4400	63	280.5	0.513002	2.128307	2.052669	Up	0	0
Spock2	5322	53.5	237.5	0.360092	1.49322	2.051989	Up	4.04E-14	1.62E-13

LOC307263	1235	14.5	64.5	0.418502	1.735303	2.051881	Up	1.45E-07	3.21E-07
N4bp3	2018	126.5	562.5	2.244664	9.305169	2.051533	Up	1.12E-13	4.31E-13
Clcn1	3197	4	18	0.045488	0.18854	2.051298	Up	0.002645	0.004342
Gtlf3b	3411	532	2365.5	5.587692	23.14595	2.050436	Up	0	0
Zcchc7	2019	670	2968.5	11.85498	49.08692	2.049846	Up	2.10E-13	7.62E-13
Tbx6	1726	8.5	38	0.176387	0.729837	2.048829	Up	1.08E-05	2.16E-05
Rfc4	1265	1231	5426	34.62111	143.1593	2.047898	Up	3.14E-12	9.07E-12
Zfp94	2476	63	279	0.910246	3.762811	2.047482	Up	0	0
Ccdc96	1773	11.5	50.5	0.232258	0.959833	2.047054	Up	1.16E-06	2.46E-06
Chac1	1569	757.5	3354	17.2731	71.37219	2.046835	Up	8.99E-13	2.86E-12
Nsg1	1640	6	26.5	0.130914	0.540854	2.046617	Up	0.00034	0.000608
F2rl3	1694	9	39.5	0.187742	0.775578	2.04652	Up	1.22E-05	2.43E-05
Ajuba	3261	204	902.5	2.234289	9.229085	2.046372	Up	1.72E-13	6.33E-13
LOC100912156	1099	66	291.5	2.140599	8.837129	2.045563	Up	0	0
LOC306365	1020	13.5	59.5	0.471634	1.94624	2.04495	Up	2.83E-07	6.18E-07
LOC100912294	2086	58	255	0.990258	4.085636	2.044684	Up	1.35E-14	5.59E-14
Lrriq3	2417	11	48	0.161667	0.666763	2.044153	Up	1.64E-06	3.45E-06
Fam126b	2080	107.5	473	1.835727	7.56676	2.043324	Up	0	0
Il1rl2	2044	11	48.5	0.192291	0.792422	2.04298	Up	1.64E-06	3.45E-06
LOC688064	1276	291	1275.5	8.094984	33.35856	2.042957	Up	1.98E-13	7.23E-13
RGD1305014	1739	248.5	1096.5	5.113855	21.05595	2.041745	Up	7.11E-14	2.79E-13
RGD1560394	665	4	18	0.216962	0.892876	2.041016	Up	0.002645	0.004343
LOC100912538	1005	586.5	2575	20.80229	85.53616	2.039792	Up	0	0
Kcnj15	1486	162.5	716.5	3.912846	16.08015	2.038991	Up	0	0
Npy5r	2475	6.5	28.5	0.09386	0.385363	2.037632	Up	0.000133	0.000244

Ccdc36	1725	39	171.5	0.808015	3.316457	2.037189	Up	1.32E-13	4.99E-13
Trmu	1566	133.5	586.5	3.049205	12.51412	2.037052	Up	1.20E-13	4.54E-13
Tmem17	1532	248.5	1089.5	5.784614	23.72433	2.036075	Up	7.11E-14	2.79E-13
Ift74	2133	947	4152	15.8643	64.95	2.033545	Up	9.46E-13	3.00E-12
RGD1305222	953	405.5	1780	15.21638	62.27348	2.032992	Up	0	0
Myo5b	6075	4	18	0.023939	0.097887	2.031779	Up	0.002645	0.004343
Fbxo34	2926	600.5	2639	7.361297	30.09634	2.031556	Up	0	0
Fam188a	2321	1120	4899.5	17.23362	70.44491	2.03127	Up	3.22E-12	9.26E-12
Hsf2bp	2816	86.5	379	1.098656	4.49091	2.031268	Up	0	0
RGD1562657	3551	546.5	2387.5	5.501802	22.47701	2.030474	Up	1.34E-13	5.04E-13
Rnf167	1882	502	2198.5	9.548387	38.99174	2.02984	Up	8.49E-13	2.71E-12
Dido1	8741	1642.5	7185	6.725875	27.41999	2.027434	Up	1.13E-12	3.52E-12
Mei1	4018	4.5	19.5	0.039719	0.16192	2.027376	Up	0.00159	0.002657
PVR	1938	3471	15166.5	64.10719	261.2925	2.027108	Up	0	0
LOC100912041	1099	1287.5	5603.5	41.87667	170.5312	2.025817	Up	7.41E-12	1.97E-11
Usp16	2903	1541.5	6701	18.92918	77.07149	2.025585	Up	1.54E-12	4.71E-12
Fhit	620	8	35	0.465419	1.894901	2.025521	Up	3.07E-05	5.96E-05
LOC100911831	3005	287.5	1253	3.418655	13.91387	2.025023	Up	4.35E-13	1.48E-12
LOC100362783	1054	38	165.5	1.290098	5.243707	2.023107	Up	1.45E-13	5.43E-13
Cpt1c	2747	311	1355.5	4.052848	16.46268	2.022191	Up	0	0
Nsrp1	1974	812.5	3533	14.71287	59.74139	2.021652	Up	1.63E-12	4.97E-12
RGD1562665	1898	74	322.5	1.394527	5.657532	2.020397	Up	9.99E-15	4.15E-14
S100a5	445	12	52	0.964941	3.911493	2.019207	Up	7.60E-07	1.63E-06
LOC502730	1734	3.5	15.5	0.071731	0.290395	2.017353	Up	0.004499	0.007238
LOC685009	2089	168	728.5	2.876085	11.63937	2.016834	Up	1.69E-13	6.23E-13

Mmrn2	3795	18	78	0.169421	0.685262	2.016047	Up	1.16E-08	2.69E-08
Med6	1086	737	3187	24.24582	97.93128	2.014034	Up	0	0
Rab40b	1861	21.5	92	0.409858	1.65534	2.013933	Up	1.69E-09	4.02E-09
Mpzl2	3255	39	168	0.426449	1.722084	2.013711	Up	1.32E-13	4.98E-13
Ccdc65	1863	55	239	1.059788	4.276837	2.012768	Up	0	0
Slfn4	2307	56	241.5	0.867109	3.499075	2.012688	Up	1.58E-14	6.49E-14
Eed	2046	1160	5007	20.23833	81.65673	2.012481	Up	3.48E-12	9.94E-12
Pot1b	2141	65	280.5	1.086634	4.383163	2.012106	Up	0	0
LOC679504	1906	344	1482	6.434787	25.95312	2.011944	Up	5.51E-13	1.83E-12
Tbk1	2987	1028.5	4434	12.29698	49.57222	2.011227	Up	0	0
LOC679863	1209	27.5	118	0.807051	3.251153	2.01022	Up	3.64E-11	9.17E-11
Lsm5	539	616	2635.5	40.48872	162.9972	2.009255	Up	5.19E-13	1.73E-12
Alg12	2058	397.5	1716	6.912181	27.80533	2.008148	Up	6.21E-13	2.04E-12
Xkr6	2835	76.5	331	0.966085	3.884421	2.007478	Up	0	0
LOC681469	912	5.5	24	0.21737	0.873666	2.006929	Up	0.000353	0.00063
Ankrd37	793	76	325.5	3.41206	13.71268	2.006796	Up	0	0
Atp6v1d	1328	3044	13100	81.96896	329.3929	2.00666	Up	0	0
LOC100911886	2310	43	185	0.664854	2.670329	2.005909	Up	1.18E-13	4.48E-13
Rbp2	674	123.5	533.5	6.575012	26.39572	2.005238	Up	0	0
Myo1f	3818	54.5	233	0.50891	2.042438	2.00481	Up	3.66E-14	1.48E-13
Smoc2	2718	22.5	96.5	0.296535	1.188844	2.003284	Up	8.89E-10	2.14E-09
Mamdc4	3906	34	146.5	0.312358	1.251873	2.002816	Up	5.99E-13	1.97E-12
Nxph1	2500	90.5	388	1.295009	5.180553	2.000144	Up	0	0
Tyw5	1492	391.5	1677.5	9.38585	37.51939	1.999077	Up	3.67E-13	1.27E-12
Ccdc34	2530	965.5	4136	13.65573	54.57982	1.998861	Up	0	0

St14	3174	4	17	0.045095	0.180238	1.998851	Up		0.004389	0.007067
Zfp606	2914	69	295.5	0.84632	3.376236	1.996141	Up		0	0
Cdkn2aip	2280	1024	4369	16.00844	63.85437	1.995953	Up		3.91E-14	1.57E-13
RGD1306502	3738	411	1746.5	3.918785	15.62467	1.995347	Up		0	0
LOC500625	2678	93	397.5	1.242017	4.950787	1.994973	Up		3.69E-14	1.48E-13
RGD1566229	771	4.5	19	0.205505	0.818092	1.993093	Up		0.00159	0.002657
Chrna1	1849	11	46.5	0.211019	0.839879	1.992806	Up		2.92E-06	6.06E-06
Iba57	2338	51.5	219	0.785881	3.122332	1.990242	Up		8.44E-15	3.52E-14
LOC501033	4356	4	17	0.032859	0.130504	1.989743	Up		0.004389	0.007068
Med13	7024	3316.5	14083	16.85749	66.94819	1.989655	Up		0	0
LOC690517	902	14.5	61	0.568554	2.256869	1.988953	Up		1.63E-07	3.61E-07
Dus2l	1936	233	989	4.293228	17.03743	1.988573	Up		0	0
Rn5-8s	156	1200	5070.5	271.5026	1077.306	1.98839	Up		1.80E-12	5.43E-12
Prkrip1	1391	301.5	1282	7.75789	30.7803	1.988271	Up		0	0
Mbd1	2491	739.5	3143	10.61452	42.10604	1.987988	Up		7.94E-13	2.54E-12
RGD1561328	1280	4	17	0.110927	0.439903	1.987579	Up		0.004389	0.007067
Klhl32	2456	44	186.5	0.642469	2.547187	1.987207	Up		8.13E-14	3.18E-13
Asb5	1614	69	290	1.5152	6.006119	1.986925	Up		0	0
Plxdc1	2874	5.5	23	0.068379	0.270945	1.986378	Up		0.000574	0.001009
Lime1	1888	91.5	386.5	1.730705	6.855961	1.985999	Up		0	0
Zfp263	3215	280.5	1191.5	3.120652	12.34776	1.984329	Up		0	0
Ubxn7	2733	137.5	581.5	1.796409	7.107494	1.984225	Up		2.87E-13	1.01E-12
Ahsp	557	13.5	57	0.863674	3.414988	1.983321	Up		3.23E-07	7.04E-07
Tmem150c	2807	12.5	52.5	0.157612	0.622999	1.982854	Up		7.60E-07	1.63E-06
Gckr	2296	10.5	44.5	0.163018	0.644148	1.982356	Up		3.67E-06	7.57E-06

Rnf138	2753	489	2062.5	6.327648	24.9865	1.981408	Up	5.92E-13	1.95E-12
Atg14	3409	205.5	867	2.153454	8.501721	1.981102	Up	4.23E-13	1.44E-12
Clp1	1835	343	1448.5	6.677075	26.35392	1.98073	Up	0	0
Pdzd9	936	5	21.5	0.192988	0.761609	1.980542	Up	0.001541	0.002584
Da2-19	1073	77.5	324.5	2.556473	10.07807	1.978993	Up	0	0
Acad11	3449	399	1683.5	4.134953	16.29508	1.978494	Up	0	0
Dennd2d	1926	20	85	0.373069	1.470186	1.978483	Up	3.25E-09	7.66E-09
Atp6v1h	1683	1289.5	5427.5	27.30556	107.588	1.97825	Up	5.50E-12	1.50E-11
LOC680227	1191	58.5	246	1.750152	6.895261	1.978125	Up	1.35E-14	5.59E-14
Tpd52l3	2290	15.5	65.5	0.241575	0.95161	1.977899	Up	8.38E-08	1.87E-07
Ggps1	2620	636	2676.5	8.657859	34.08361	1.976996	Up	1.16E-12	3.60E-12
LOC100910577	2022	123	518	2.166232	8.527283	1.976899	Up	0	0
Fcrlb	1412	23.5	99.5	0.596152	2.346627	1.976837	Up	4.69E-10	1.14E-09
Fnip2	4298	465	1958	3.866446	15.2137	1.976291	Up	0	0
Zkscan3	2588	532.5	2238.5	7.342389	28.8417	1.973835	Up	0	0
Fcgbpl1	7938	5	21	0.022467	0.088212	1.973162	Up	0.001541	0.002584
LOC302022	1388	824.5	3455	21.19668	83.18792	1.972536	Up	7.74E-13	2.48E-12
Nos3	3953	10	42	0.090812	0.356323	1.972235	Up	7.16E-06	1.45E-05
Amt	2177	56.5	236.5	0.928029	3.633409	1.969082	Up	1.58E-14	6.49E-14
Dnah8	14196	17	71	0.04273	0.167044	1.966912	Up	2.46E-08	5.60E-08
Vpreb3	513	10	42	0.702	2.74395	1.966711	Up	7.16E-06	1.45E-05
Fam83f	3062	23	95.5	0.268221	1.046731	1.964395	Up	4.76E-10	1.15E-09
Zfp296	1438	15	63	0.374457	1.460827	1.963914	Up	9.82E-08	2.19E-07
Gch1	1016	267	1119	9.403665	36.68291	1.963813	Up	7.08E-13	2.29E-12
Asb1	2044	97	406.5	1.701214	6.635171	1.963569	Up	5.35E-14	2.12E-13

Tsc22d1	4547	7489	31282.5	58.91149	229.642	1.962766	Up	0	0
Zfp187	4080	853.5	3553.5	7.456654	29.0587	1.96237	Up	9.86E-13	3.11E-12
LOC678893	2664	1181	4927.5	15.85238	61.75348	1.96182	Up	2.07E-12	6.17E-12
Usp11	3423	47.5	198.5	0.496302	1.933343	1.961806	Up	0	0
Arid5b	4037	1621	6780	14.38913	56.03185	1.961268	Up	3.94E-12	1.11E-11
Hsd17b1	1431	25	105	0.627147	2.440961	1.960572	Up	1.31E-10	3.23E-10
LOC684830	1368	130	540.5	3.390812	13.19614	1.960413	Up	0	0
Cir1	1356	619.5	2581.5	16.34851	63.62324	1.960395	Up	4.35E-13	1.48E-12
Cetn3	936	304	1261	11.57657	45.0083	1.958983	Up	0	0
Ifit3	2053	33	137.5	0.574343	2.232361	1.958585	Up	1.01E-12	3.19E-12
Meig1	566	18	75	1.137983	4.422457	1.95837	Up	1.25E-08	2.90E-08
Sco1	1600	380	1576	8.470204	32.89051	1.957203	Up	0	0
Ifitm3	642	185	768.5	10.30512	39.9184	1.953693	Up	0	0
Hnrnph3	1879	443	1837.5	8.419297	32.60751	1.953433	Up	0	0
Vwa3b	4071	5	21	0.04409	0.170676	1.952745	Up	0.001541	0.002584
Rrh	1109	17	70.5	0.54594	2.11147	1.951432	Up	2.65E-08	6.05E-08
Lilrb4	1152	9.5	39.5	0.296332	1.145945	1.951253	Up	1.22E-05	2.43E-05
Fbxl22	4460	18	74.5	0.144417	0.558401	1.951064	Up	1.33E-08	3.06E-08
Atxn7	2760	309.5	1279.5	4.006727	15.48438	1.950317	Up	7.28E-13	2.34E-12
Spin2a	1370	12	49.5	0.313011	1.209268	1.949848	Up	1.70E-06	3.56E-06
LOC100359879	803	16	66	0.714419	2.759813	1.949728	Up	5.40E-08	1.22E-07
Sgca	1693	50	206	1.056122	4.069866	1.946205	Up	3.62E-14	1.46E-13
Hist1h1b	669	39	161	2.086875	8.037484	1.9454	Up	1.32E-13	4.99E-13
Ttf1	2587	281	1156	3.875693	14.92619	1.94532	Up	0	0
Slfn13	3396	4300.5	17716	45.24064	174.1611	1.944731	Up	1.61E-11	4.17E-11

LOC100359937	917	660.5	2718.5	25.69747	98.92366	1.944689	Up	0	0
LOC100910965	537	20	82.5	1.330573	5.121717	1.94458	Up	3.45E-09	8.13E-09
Lamp3	1927	8	33	0.148555	0.571751	1.944389	Up	7.40E-05	0.000139
Gabpb1	2527	702.5	2893	9.93506	38.16899	1.941801	Up	0	0
Mmp13	2602	346	1422	4.750769	18.24391	1.941182	Up	3.04E-13	1.07E-12
Atf4	1173	6524.5	26850.5	199.0193	763.9557	1.940581	Up	4.50E-11	1.13E-10
RGD1560994	660	2424	9911.5	130.6201	501.3096	1.940325	Up	0	0
S1pr3	3606	7.5	31	0.073868	0.283275	1.939188	Up	7.95E-05	0.000149
LOC100909787	2886	226.5	927.5	2.804477	10.7543	1.939111	Up	3.18E-14	1.29E-13
LOC100910438	1457	270.5	1109	6.645517	25.42298	1.93568	Up	4.53E-13	1.53E-12
RGD1308019	3010	40	164	0.474763	1.814298	1.934133	Up	1.16E-13	4.43E-13
Herc6	4591	26	106	0.201151	0.768504	1.933776	Up	6.94E-11	1.73E-10
Unc13c	7264	93.5	383	0.460472	1.758982	1.933554	Up	3.69E-14	1.49E-13
Orai2	4047	856.5	3510	7.576856	28.9429	1.933538	Up	1.12E-12	3.49E-12
Atp6v1b2	2717	2643.5	10786.5	34.70938	132.5774	1.933437	Up	0	0
LOC681371	3469	1585.5	6472.5	16.29722	62.24355	1.933299	Up	4.62E-12	1.28E-11
Slitrk2	2947	11	45.5	0.134927	0.515015	1.932437	Up	4.20E-06	8.62E-06
Gstm4	1583	101	412.5	2.278727	8.697416	1.932359	Up	0	0
LOC100911235	1229	452.5	1847	13.14291	50.1504	1.931977	Up	6.60E-13	2.15E-12
LOC100363500	695	64	260.5	3.285246	12.52894	1.931191	Up	0	0
LOC685572	1310	4.5	18.5	0.121387	0.462905	1.931097	Up	0.002645	0.004343
Tmem19	1842	351	1430	6.793724	25.90746	1.931093	Up	9.48E-14	3.68E-13
Plch2	4470	24	97.5	0.191098	0.728538	1.930688	Up	2.55E-10	6.23E-10
LOC366469	774	9	36.5	0.41238	1.571536	1.93013	Up	4.35E-05	8.39E-05
Ccdc28b	820	28	114.5	1.221866	4.655902	1.929974	Up	1.94E-11	5.00E-11

Slc7a7	2077	87	355.5	1.502454	5.723814	1.929657	Up	0	0
Slc25a32	2348	328	1335.5	4.977188	18.95014	1.928806	Up	2.05E-13	7.47E-13
LOC687539	1254	79	323.5	2.259317	8.598232	1.928153	Up	0	0
Lpar6	1834	261.5	1066	5.099165	19.39948	1.927685	Up	2.23E-13	8.05E-13
Mlycd	2020	144.5	587	2.555909	9.700672	1.924248	Up	1.05E-13	4.06E-13
LOC100912353	647	24	98	1.325581	5.020876	1.921315	Up	2.52E-10	6.16E-10
Pdrg1	1237	579	2348	16.73554	63.35977	1.920652	Up	9.45E-13	3.00E-12
Tmem71	879	20	81	0.808962	3.062265	1.920456	Up	3.61E-09	8.51E-09
Jrkl	2897	357	1438	4.381295	16.57861	1.919894	Up	3.20E-13	1.12E-12
RGD1561238	336	18	73	1.906719	7.213502	1.919608	Up	1.45E-08	3.33E-08
Sertad4	3197	2695.5	10920.5	30.14576	114.0417	1.919534	Up	0	0
Zap70	2277	10.5	42	0.16312	0.617016	1.919379	Up	7.16E-06	1.45E-05
Six4	5970	344	1394.5	2.061017	7.792841	1.918793	Up	5.51E-13	1.83E-12
Ppp1r3f_predicted	1920	113	457.5	2.103902	7.953342	1.918494	Up	2.08E-13	7.57E-13
LOC100361660	1689	167.5	675.5	3.533555	13.34875	1.917513	Up	1.94E-13	7.09E-13
MGC114483	1467	21.5	86	0.518763	1.958463	1.916576	Up	1.78E-09	4.24E-09
RGD1563701	2157	68	275.5	1.128076	4.257819	1.91625	Up	0	0
Nek3	1801	31	125.5	0.615606	2.322262	1.915451	Up	3.09E-12	8.93E-12
RGD1565065	576	16	65	0.99597	3.756671	1.915281	Up	6.27E-08	1.41E-07
Cutc	1302	254	1022	6.944639	26.19095	1.915097	Up	3.19E-13	1.12E-12
Ppp1r3b	4360	62	250.5	0.508187	1.916043	1.9147	Up	0	0
LOC100911230	598	16	65	0.959329	3.616961	1.914681	Up	6.27E-08	1.41E-07
Rpp14	1906	1209.5	4874	22.64351	85.36313	1.914515	Up	3.27E-12	9.39E-12
LOC100909617	1296	17	69	0.470706	1.774066	1.914161	Up	2.97E-08	6.77E-08
Lmod1	3937	11	44.5	0.09925	0.374057	1.914115	Up	6.26E-06	1.27E-05

Slc1a2	2229	5	20	0.079496	0.299568	1.913933	Up	0.002522	0.004147
Phc3	3415	650.5	2628.5	6.820385	25.68506	1.913004	Up	0	0
Syce2	753	18.5	74.5	0.872663	3.285879	1.912782	Up	1.33E-08	3.06E-08
LOC100359855	728	14.5	58	0.706808	2.659938	1.912003	Up	2.42E-07	5.30E-07
LOC680770	1941	15	60.5	0.276237	1.038564	1.910614	Up	1.74E-07	3.83E-07
Rictor	5300	643	2586	4.334757	16.29398	1.910316	Up	0	0
RGD1305089	2363	364.5	1464.5	5.508389	20.69629	1.90967	Up	1.34E-13	5.03E-13
Sh3bgrl2	2685	50	202	0.669558	2.514799	1.909162	Up	3.62E-14	1.46E-13
Spata7	1958	344.5	1386.5	6.2928	23.63063	1.908884	Up	5.51E-13	1.83E-12
LOC100912037	1037	137.5	547.5	4.691834	17.60408	1.907686	Up	2.87E-13	1.01E-12
Olr1220	942	5.5	22	0.207404	0.777824	1.907002	Up	0.00094	0.001601
Rnf146	1889	715.5	2863	13.51315	50.63115	1.905661	Up	0	0
Fam46b	1724	46	185.5	0.954773	3.576452	1.905299	Up	7.84E-14	3.07E-13
Kcnn1	1611	25	100	0.554228	2.07578	1.905103	Up	1.35E-10	3.34E-10
Dusp12	1314	187.5	751	5.099727	19.07633	1.903292	Up	3.37E-13	1.18E-12
LOC686506	1839	153	612	2.972394	11.11861	1.903279	Up	0	0
Grin2a	4717	7	28	0.053224	0.198947	1.902241	Up	0.000322	0.000577
Ces1a	1896	7	28	0.131204	0.490206	1.901579	Up	0.000322	0.000577
Pigv	2429	381.5	1527	5.618599	20.98059	1.900773	Up	3.02E-13	1.06E-12
Mlh1	2274	505.5	2025	7.9513	29.68944	1.900687	Up	0	0
Rnf190	3008	21	83.5	0.248101	0.926346	1.900625	Up	2.10E-09	4.98E-09
Tnfaip6	1401	208	831	5.30152	19.79111	1.900374	Up	3.49E-13	1.21E-12
RGD1311188	1874	190.5	761	3.638281	13.57752	1.899891	Up	2.69E-13	9.58E-13
Noxo1	1965	16.5	66	0.300616	1.121847	1.899883	Up	5.40E-08	1.22E-07
Cgrrf1	1265	519	2072	14.65519	54.66654	1.899247	Up	0	0

Adat1	3057	100	399.5	1.172035	4.370325	1.898725	Up	1.75E-14	7.19E-14
Hmg20a	3638	1949.5	7790	19.16756	71.44817	1.89823	Up	3.04E-12	8.80E-12
Cdk7	1460	759	3018	18.5415	69.03685	1.896609	Up	1.87E-12	5.61E-12
Jun	3107	6127.5	24471	70.62645	262.8175	1.895781	Up	1.55E-11	4.02E-11
Plcb2	3999	6	24	0.053975	0.200821	1.895546	Up	0.000894	0.001526
Prnp	2142	7797.5	31032	130.0679	483.8384	1.89526	Up	0	0
Scube1	4237	6	24	0.051214	0.19039	1.894354	Up	0.000894	0.001526
Tmem174	1378	6	24	0.155805	0.57887	1.893498	Up	0.000894	0.001525
Trim39	2904	223.5	889	2.753287	10.22348	1.892659	Up	7.02E-14	2.76E-13
Nmnat1	2130	222	883	3.727366	13.82627	1.891183	Up	0	0
Adamts6	4770	539	2137.5	4.034775	14.96551	1.891081	Up	4.59E-13	1.55E-12
LOC687565	815	4088	16209.5	179.0577	664.0349	1.890834	Up	1.29E-11	3.36E-11
Chd2	6092	1298.5	5155	7.616926	28.24012	1.890466	Up	2.50E-12	7.36E-12
F11r	1895	14	55.5	0.263151	0.975207	1.889817	Up	5.70E-07	1.23E-06
Taf5	3240	216.5	859.5	2.387443	8.840529	1.888666	Up	2.34E-13	8.42E-13
Tesk2	3045	107	426	1.257031	4.654016	1.888456	Up	0	0
RGD1562462	3136	318	1265	3.641695	13.48221	1.888375	Up	1.88E-13	6.87E-13
Noc3l	2936	1122	4446	13.65083	50.52594	1.888036	Up	2.95E-12	8.57E-12
Stam	3022	2574	10204	30.46693	112.6978	1.887144	Up	0	0
LOC100362059	2568	47	186	0.654688	2.420307	1.886311	Up	0	0
LOC257642	1038	58269.5	231873.5	2012.397	7435.518	1.885518	Up	9.96E-11	2.48E-10
LOC502619	419	7	27.5	0.590968	2.181592	1.88423	Up	0.000518	0.000914
Olr87	939	7.5	29.5	0.284893	1.05129	1.883671	Up	0.0002	0.000364
Prss12	2499	57.5	228.5	0.823917	3.03891	1.882982	Up	5.37E-14	2.13E-13
Prpf4b	4378	2093.5	8256	17.0586	62.91435	1.88289	Up	0	0

Gen1	4285	451	1775	3.750292	13.82476	1.88218	Up	2.05E-12	6.11E-12
LOC100911339	1770	67.5	265.5	1.359917	5.012852	1.882113	Up	0	0
Gabpb1l	1832	337	1329.5	6.567685	24.1886	1.88087	Up	0	0
Ngrn	1349	468	1849	12.40215	45.6734	1.880764	Up	1.12E-12	3.49E-12
RGD1564342	3351	35.5	139	0.378141	1.391873	1.880031	Up	3.77E-13	1.30E-12
Mtrf1l	1264	181.5	713.5	5.109824	18.79449	1.878965	Up	1.41E-13	5.30E-13
Dhx36	5664	1581.5	6191	9.935546	36.49944	1.877203	Up	5.17E-12	1.41E-11
LOC100911809	916	59	233	2.302937	8.45909	1.877027	Up	2.53E-14	1.03E-13
Nrg1	3842	645	2537	6.009724	22.04127	1.874836	Up	5.31E-13	1.77E-12
Slc25a25	3150	590.5	2318	6.700381	24.55474	1.873686	Up	0	0
Semg1	1545	13.5	53	0.310628	1.138318	1.873645	Up	7.07E-07	1.52E-06
Dkk1	2295	13.5	53	0.210615	0.771809	1.873638	Up	7.07E-07	1.52E-06
Tmem202	1282	22.5	88	0.628245	2.300187	1.872353	Up	9.83E-10	2.36E-09
Zfp394	2472	172.5	677	2.495966	9.133362	1.871548	Up	1.17E-13	4.46E-13
Tra2a	1813	1794.5	7020	35.34076	129.2536	1.870799	Up	1.43E-12	4.38E-12
LOC100912569	930	4.5	17.5	0.172836	0.631634	1.869681	Up	0.004389	0.007066
Etv3	1598	267.5	1048	5.998789	21.90418	1.868463	Up	7.08E-13	2.29E-12
RGD1559505	1959	137.5	537	2.50734	9.152893	1.86807	Up	2.87E-13	1.02E-12
Abca4	7163	38	148	0.189431	0.691086	1.867191	Up	1.45E-13	5.43E-13
Adam5	2591	10.5	40.5	0.143794	0.524128	1.865917	Up	1.64E-05	3.23E-05
LOC503131	939	7	27.5	0.267365	0.974427	1.865742	Up	0.000518	0.000914
Dsg3	2928	4.5	17.5	0.054701	0.199084	1.863742	Up	0.004389	0.007066
LOC683508	1990	82	321.5	1.483706	5.396839	1.862909	Up	0	0
Cish	1879	287.5	1120.5	5.470657	19.89823	1.862854	Up	4.35E-13	1.48E-12
Ccnt1	1992	421.5	1645	7.584701	27.58449	1.862693	Up	4.13E-13	1.41E-12

Bc1	152	326.5	1277.5	76.9633	279.8895	1.862615	Up	6.55E-13	2.14E-12
RGD1308782	1657	47	182.5	1.012205	3.678541	1.861632	Up	0	0
Nt5e	2826	975	3795.5	12.3113	44.72933	1.861238	Up	1.67E-12	5.06E-12
LOC100909815	1451	8	31	0.197289	0.716397	1.86045	Up	0.000187	0.000341
Dusp16	1986	387	1504	6.968535	25.29837	1.860117	Up	7.51E-13	2.41E-12
Cfi	2021	17	66	0.300997	1.091652	1.858693	Up	5.63E-08	1.27E-07
Hps4	2372	411.5	1601.5	6.218515	22.53699	1.857652	Up	0	0
Fst	1035	1138.5	4406.5	39.25823	142.1687	1.856537	Up	1.82E-12	5.49E-12
LOC100911964	480	23.5	90.5	1.747706	6.325744	1.855772	Up	5.61E-10	1.36E-09
Zfp143	3040	681	2641.5	8.019876	29.02701	1.855744	Up	1.16E-13	4.45E-13
Mthfr	2104	63	244.5	1.073909	3.886419	1.85557	Up	0	0
Zkscan5	3782	636.5	2467.5	6.020626	21.77229	1.854508	Up	1.16E-12	3.60E-12
3-Mar	1799	261	1007	5.166909	18.68195	1.854271	Up	2.23E-13	8.05E-13
Aipl1	1446	16.5	64	0.40891	1.477701	1.8535	Up	7.66E-08	1.71E-07
Ifit1lb	2187	57	219.5	0.927116	3.349454	1.853104	Up	5.37E-14	2.13E-13
Tex10	3893	1206.5	4663.5	11.06274	39.94789	1.852411	Up	4.33E-12	1.21E-11
Bod1	1474	386.5	1493.5	9.37714	33.85506	1.852151	Up	1.55E-13	5.74E-13
Sat1	1193	2458	9471	73.41459	265.0199	1.851962	Up	0	0
Mios	3744	442	1707	4.216139	15.21963	1.851939	Up	0	0
Ppwd1	2453	713	2754.5	10.38081	37.46491	1.851621	Up	1.46E-12	4.49E-12
LOC691468	3615	170	656.5	1.681166	6.066803	1.851474	Up	3.78E-13	1.30E-12
Gabarapl2	1619	1175.5	4529.5	25.86356	93.31644	1.851211	Up	5.04E-12	1.38E-11
LOC680097	430	28.5	111	2.37768	8.578687	1.851202	Up	1.98E-11	5.08E-11
Ppif	1498	267	1030	6.359545	22.9448	1.851172	Up	7.08E-13	2.29E-12
Cebpd	1139	727.5	2825	22.93822	82.68423	1.849859	Up	1.35E-12	4.15E-12

Kdm4c	4126	599.5	2309.5	5.189438	18.70603	1.849853	Up	7.53E-13	2.42E-12
Runx1	2006	2986	11573	53.45067	192.6163	1.84945	Up	0	0
LOC100911647	754	121	461.5	5.654181	20.36998	1.849054	Up	0	0
LOC100911856	378	80	302.5	7.454843	26.83614	1.847927	Up	0	0
Aste1	2424	66	254.5	0.973823	3.503669	1.847135	Up	0	0
LOC691113	501	43	166	3.074649	11.0605	1.846923	Up	1.18E-13	4.48E-13
Avil	3014	56	215.5	0.664471	2.390281	1.846903	Up	1.58E-14	6.49E-14
Mia	588	69	265	4.183449	15.033	1.845368	Up	0	0
Wdr96	5208	65	250.5	0.446934	1.605616	1.844994	Up	0	0
Rgs2	685	49	189	2.561351	9.198696	1.844525	Up	3.91E-14	1.57E-13
RGD1307887	2071	5	19	0.086114	0.308474	1.840824	Up	0.004105	0.006627
Aldh1l1	3109	4.5	17.5	0.051701	0.185178	1.840655	Up	0.004389	0.007069
Irak3	2972	669.5	2569.5	8.067323	28.88414	1.840116	Up	1.58E-12	4.82E-12
Eif1b	882	1027.5	3939	41.65602	149.0918	1.839604	Up	0	0
RGD1559150	2613	54	207.5	0.741687	2.654196	1.839392	Up	3.66E-14	1.48E-13
Sdc4	2488	4790	18326	68.742	245.9603	1.839162	Up	2.40E-11	6.13E-11
Scn2a1	8553	170	650.5	0.711364	2.544133	1.838514	Up	3.78E-13	1.30E-12
Churc1	535	447.5	1707.5	29.7588	106.4014	1.838128	Up	8.15E-13	2.61E-12
Pp2d1	2461	83.5	317	1.201168	4.293953	1.837869	Up	1.43E-13	5.34E-13
Wdr31	1350	135.5	518	3.584143	12.81198	1.837793	Up	2.15E-13	7.78E-13
Ttc25	2215	31	118.5	0.500545	1.789204	1.837747	Up	3.32E-12	9.51E-12
Thap6	796	108.5	418	4.895859	17.49948	1.837678	Up	0	0
Nhej1	1445	68	261.5	1.687885	6.029827	1.8369	Up	0	0
Wfikkn1	1659	41	157.5	0.886755	3.167735	1.836844	Up	1.47E-13	5.48E-13
Pthlh	1087	27.5	105	0.903961	3.227521	1.836094	Up	3.93E-11	9.88E-11

Faslg	1623	15	57	0.329654	1.176986	1.836074	Up	4.84E-07	1.05E-06
Ebf1	2895	46.5	178.5	0.577233	2.059342	1.834959	Up	7.84E-14	3.07E-13
Boc	4430	21	81	0.170533	0.608224	1.83455	Up	2.75E-09	6.51E-09
Stk17b	2159	1814.5	6921.5	30.00457	106.9798	1.834084	Up	2.11E-12	6.30E-12
Wdr37	4354	508	1936	4.161814	14.83775	1.833988	Up	7.72E-13	2.48E-12
Tert	3378	34.5	132	0.365035	1.300905	1.83341	Up	6.04E-13	1.99E-12
Nebl	3127	9	34	0.101706	0.362441	1.833338	Up	0.000109	0.000203
LOC100910810	1349	15	57	0.398311	1.419384	1.833296	Up	4.84E-07	1.05E-06
Fermt3	2511	17.5	66.5	0.249728	0.889754	1.83305	Up	5.63E-08	1.27E-07
Serpina3n	2061	11	42	0.191261	0.681245	1.832631	Up	1.49E-05	2.96E-05
Atg12	2590	1138	4330.5	15.66385	55.78808	1.832519	Up	1.82E-12	5.48E-12
RGD1566052	1531	369	1407	8.616197	30.64568	1.830561	Up	0	0
Ripk2	2582	1144.5	4365.5	15.86009	56.40584	1.830443	Up	2.98E-12	8.63E-12
Gtf2a2	330	141.5	536	15.2157	54.11373	1.830434	Up	0	0
Bdnf	3751	898	3417.5	8.548721	30.38517	1.829587	Up	2.45E-12	7.21E-12
Pex13	4972	768.5	2926.5	5.523763	19.63243	1.829516	Up	0	0
Odf1	969	5.5	21	0.2034	0.722624	1.828927	Up	0.001541	0.002584
Tfb2m	2092	626	2373.5	10.661	37.87139	1.828765	Up	0	0
Nfkbia	1583	2376	9054	53.71854	190.8224	1.828738	Up	0	0
RGD1565192	922	294	1113	11.3487	40.31098	1.828646	Up	0	0
Nphs1	5820	76	289	0.466878	1.657404	1.827806	Up	0	0
RGD1566036	3167	488.5	1854	5.505602	19.53851	1.827348	Up	1.35E-12	4.17E-12
Tnfrsf22	977	1824.5	6957	66.91413	237.3734	1.826776	Up	1.08E-12	3.37E-12
RGD1564680	4022	5	19	0.044057	0.156153	1.825533	Up	0.004105	0.006626
Fam173b	1378	249.5	944	6.460793	22.88995	1.824931	Up	1.13E-14	4.70E-14

Tubb4a	2086	5	19	0.085495	0.302804	1.824471	Up	0.004105	0.006627
Ndrg1	2906	1069.5	4057.5	13.15328	46.5827	1.824372	Up	2.20E-12	6.53E-12
Actl7a	1507	21	79	0.495975	1.75558	1.823609	Up	4.41E-09	1.04E-08
Polg2	1872	91	344	1.734255	6.135183	1.82279	Up	0	0
Zdhhc17	2904	479	1806.5	5.873827	20.77136	1.822223	Up	0	0
Lrig2	4609	431	1630	3.340588	11.80373	1.821069	Up	0	0
Cdc40	3105	634.5	2398.5	7.295328	25.7572	1.819931	Up	6.54E-13	2.14E-12
Ptprv	5446	155	586.5	1.018962	3.591832	1.817619	Up	3.73E-13	1.29E-12
Sh3bp2	2877	69.5	263	0.866911	3.055173	1.817298	Up	0	0
Mob4	2513	1810.5	6799.5	25.60913	90.24921	1.817256	Up	3.85E-12	1.09E-11
LOC100912071	2598	9	34	0.124181	0.437626	1.817252	Up	0.000109	0.000203
Rufy4	1844	9.5	35.5	0.184505	0.649828	1.816396	Up	6.87E-05	0.00013
Prl2a1	1014	16	60	0.559537	1.969338	1.815404	Up	2.82E-07	6.15E-07
RGD1560859	1023	4.5	17	0.158245	0.556575	1.814415	Up	0.004389	0.007068
Ccdc77	2441	354.5	1334	5.182022	18.22147	1.814052	Up	0	0
Epsti1	1582	12.5	47	0.282194	0.991896	1.813504	Up	3.66E-06	7.55E-06
Ptpn18	1362	6	23	0.157635	0.553889	1.813004	Up	0.001447	0.00243
Tuba3a	1534	8.5	32	0.197717	0.694705	1.812967	Up	0.000117	0.000216
Tspan5	2877	1365	5122.5	16.95831	59.46322	1.810005	Up	1.70E-12	5.14E-12
LOC100912606	1032	10.5	40	0.367685	1.287085	1.807564	Up	1.64E-05	3.23E-05
Fbxo32	1053	31	116	1.051814	3.679649	1.806689	Up	3.67E-12	1.04E-11
Ric8b	1760	374.5	1401	7.600907	26.5874	1.806499	Up	4.81E-13	1.62E-12
Malt1	3729	251.5	940	2.407303	8.418263	1.806105	Up	1.37E-13	5.16E-13
LOC100359662	1209	164	616	4.856816	16.9812	1.805856	Up	1.50E-13	5.56E-13
Fam107a	2875	145.5	540.5	1.797479	6.282199	1.805294	Up	3.55E-15	1.49E-14

Eri2	3451	482	1802	4.982046	17.40977	1.805087	Up	1.31E-14	5.41E-14
Oas1b	1878	70	263	1.338048	4.672221	1.803979	Up	0	0
Doc2a	1447	14.5	54.5	0.360754	1.259662	1.80395	Up	8.32E-07	1.78E-06
Rasal2	3700	491	1837.5	4.750547	16.58657	1.80385	Up	5.39E-13	1.79E-12
LOC100912165	403	43.5	162	3.858901	13.47237	1.803742	Up	1.18E-13	4.49E-13
Slc25a46	2947	978.5	3648	11.83168	41.2891	1.803106	Up	2.73E-12	7.98E-12
Ubap1	2662	1299.5	4855.5	17.439	60.84242	1.80276	Up	4.18E-12	1.17E-11
LOC100911629	774	22	82	1.014872	3.53943	1.802219	Up	2.51E-09	5.94E-09
LOC100911357	1555	81.5	304	1.87269	6.528226	1.801579	Up	5.82E-14	2.30E-13
Rab21	1515	1867.5	6950.5	43.94155	153.1511	1.801299	Up	3.53E-12	1.01E-11
RGD1561589	876	48	178	1.942396	6.769694	1.801253	Up	1.06E-13	4.07E-13
Ccl24	1485	53.5	197.5	1.276224	4.446091	1.800656	Up	4.04E-14	1.62E-13
Slc35g3	1347	20	75.5	0.533857	1.859654	1.800509	Up	1.05E-08	2.44E-08
Trpm1	5030	136	506.5	0.964991	3.360928	1.800273	Up	1.95E-13	7.10E-13
Narg2	3155	507.5	1887.5	5.738582	19.96975	1.79905	Up	9.98E-13	3.15E-12
Fhl4	1626	48	177.5	1.050689	3.655972	1.798919	Up	1.06E-13	4.07E-13
RGD1304978	2026	82.5	307.5	1.456974	5.068457	1.798571	Up	0	0
Mettl17	1609	217	811.5	4.839854	16.83166	1.798142	Up	2.24E-13	8.08E-13
Ces2h	2065	16	59	0.274478	0.954345	1.797819	Up	4.25E-07	9.23E-07
Ecscr-ps1	657	5	19	0.274941	0.955935	1.797788	Up	0.004105	0.006628
Stmn3	1123	7	26	0.223048	0.774583	1.796068	Up	0.000831	0.001423
LOC680700	657	64.5	239.5	3.50642	12.15767	1.793797	Up	0	0
RGD1306820	5203	562	2079.5	3.850674	13.34632	1.79326	Up	3.62E-13	1.25E-12
Gipc2	1376	115	424	2.972268	10.30022	1.79304	Up	0	0
Tfb1m	1660	409.5	1518	8.808423	30.51457	1.792542	Up	4.45E-13	1.51E-12

Tmem8c	1418	17	63	0.426569	1.477623	1.792428	Up	1.64E-07	3.62E-07
Arl13b	3535	512	1895.5	5.165067	17.88793	1.792128	Up	3.68E-13	1.28E-12
Mphosph10	2191	1999.5	7408	32.59273	112.872	1.792065	Up	0	0
Zmym6	4134	146	542.5	1.263475	4.374237	1.791634	Up	3.33E-14	1.35E-13
Terf2	1982	551	2046	9.953887	34.45717	1.791472	Up	7.88E-14	3.09E-13
LOC682206	5649	159.5	590.5	1.006942	3.485601	1.791427	Up	8.53E-14	3.32E-13
Ptges	1254	11	40.5	0.312973	1.082947	1.790851	Up	3.68E-05	7.12E-05
Zufsp	2016	348.5	1289.5	6.164836	21.33142	1.790846	Up	4.24E-14	1.70E-13
Blnk	1765	40.5	149.5	0.820601	2.836362	1.789289	Up	1.16E-13	4.44E-13
Clec12a	1441	11	40	0.268777	0.928642	1.788712	Up	3.68E-05	7.12E-05
Zfp709	2768	237.5	880.5	3.070377	10.60522	1.788286	Up	2.75E-13	9.80E-13
LOC689207	3054	11	41	0.1302	0.449692	1.788212	Up	2.34E-05	4.59E-05
Filip1l	4823	916	3392	6.799636	23.47729	1.787736	Up	0	0
Tnnc2	483	17.5	65	1.303024	4.498639	1.787625	Up	7.74E-08	1.73E-07
Rad51b	1535	39.5	146	0.918752	3.171449	1.787395	Up	1.33E-13	5.00E-13
Tsc22d2	4606	1088	4027	8.456098	29.1561	1.785734	Up	2.48E-12	7.29E-12
Fmo4	1640	34	125.5	0.741148	2.55518	1.785591	Up	7.24E-13	2.33E-12
Rnaseh1	1375	206.5	763.5	5.370446	18.51489	1.785572	Up	2.46E-13	8.83E-13
LOC690358	3674	13	48	0.127395	0.439131	1.785343	Up	5.08E-06	1.04E-05
Msh5	2679	127.5	471.5	1.705257	5.877877	1.785306	Up	9.17E-14	3.56E-13
LOC100910444	2129	75.5	278	1.268026	4.368029	1.784398	Up	0	0
RGD1560234	1104	21	77.5	0.67962	2.340877	1.784248	Up	8.56E-09	1.99E-08
Mettl22	1816	176	653.5	3.478083	11.97904	1.784147	Up	0	0
Itpkc	3309	240.5	890	2.604298	8.96732	1.783782	Up	0	0
Lace1	2045	108	398.5	1.885569	6.491635	1.783582	Up	0	0

LOC100361710	4037	161	590	1.419262	4.883433	1.782755	Up	0	0
Crbn	2257	838.5	3083	13.25994	45.62421	1.782725	Up	0	0
Pcdh9	5595	345.5	1270	2.205417	7.57874	1.780906	Up	9.33E-13	2.96E-12
Cenpq	1470	470	1726	11.41905	39.22499	1.78033	Up	1.65E-12	5.01E-12
Tm2d1	1422	497.5	1824.5	12.47494	42.84556	1.780113	Up	3.54E-13	1.23E-12
Car5a	1200	29.5	109	0.880866	3.025135	1.780005	Up	1.30E-11	3.40E-11
LOC691543	1275	860.5	3142	23.97634	82.32429	1.779706	Up	1.10E-12	3.45E-12
LOC100365187	1572	25	92	0.570166	1.957371	1.779463	Up	3.43E-10	8.35E-10
Swt1	3679	331.5	1215.5	3.214698	11.03099	1.778807	Up	0	0
Scyl3	2966	307.5	1128.5	3.707603	12.70859	1.777245	Up	4.91E-14	1.95E-13
Scn9a	9316	6	22	0.023293	0.07981	1.776703	Up	0.002332	0.003849
Mier3	4758	604	2216	4.536685	15.53507	1.775818	Up	6.80E-13	2.21E-12
Ppef2	3095	7.5	27.5	0.08662	0.296506	1.775299	Up	0.000518	0.000914
Luc7l	1743	787	2875	16.09994	55.08966	1.774726	Up	1.26E-12	3.90E-12
Fam108b1	1623	289.5	1053.5	6.334548	21.67243	1.774547	Up	0	0
Vsx2	3087	12.5	46	0.145173	0.495754	1.771847	Up	5.61E-06	1.14E-05
LOC680693	1099	6	22	0.194315	0.66343	1.771545	Up	0.002332	0.003849
Zbtb17	2795	741	2714	9.500669	32.42923	1.771194	Up	0	0
RGD1311344	2453	40	145.5	0.580229	1.980526	1.77119	Up	1.18E-13	4.48E-13
Mdm4	1923	705.5	2571.5	13.07652	44.63028	1.771044	Up	0	0
Krt7	2061	66.5	243	1.154995	3.939493	1.770123	Up	0	0
RGD1563120	1422	89	325.5	2.24363	7.636327	1.767044	Up	0	0
Lrrc14b	2575	21	76	0.288929	0.9833	1.766915	Up	1.26E-08	2.90E-08
Msrb2	1168	22	79.5	0.669581	2.278255	1.766599	Up	7.31E-09	1.70E-08
Cspp1	4232	708	2583	5.990214	20.37912	1.766412	Up	1.11E-12	3.47E-12

Pole4	1741	871	3160.5	17.78628	60.48964	1.765923	Up	0	0
LOC100912383	1195	2151	7809	64.24139	218.1783	1.763932	Up	0	0
Ttll11	2334	12	44	0.184467	0.626315	1.763527	Up	1.36E-05	2.71E-05
Cnksr1	2512	50	182	0.711789	2.416083	1.763148	Up	3.62E-14	1.46E-13
Lrrc4c	4014	12	44	0.107118	0.363508	1.762782	Up	1.36E-05	2.71E-05
Hoxc5	2734	14	51	0.183655	0.623126	1.762528	Up	2.96E-06	6.13E-06
Trim29	2832	8	29	0.100678	0.341567	1.762425	Up	0.000477	0.000847
Mcart1	2243	1138	4147.5	18.17044	61.61892	1.76178	Up	1.82E-12	5.49E-12
Fmc1	1367	106.5	385.5	2.775419	9.410948	1.761634	Up	0	0
Clec2g	624	73.5	266.5	4.218068	14.30006	1.761367	Up	0	0
LOC100912349	659	16	58	0.867919	2.939811	1.760092	Up	6.52E-07	1.40E-06
Rg9mtd1	2651	616.5	2227	8.277074	28.02894	1.759725	Up	5.19E-13	1.74E-12
Atp13a3	790	94.5	341	4.261164	14.42494	1.759246	Up	0	0
Hic2	4509	90.5	328	0.717505	2.428471	1.758988	Up	0	0
Ralyl	1513	25	90.5	0.590505	1.998517	1.758909	Up	6.61E-10	1.60E-09
Slc30a3	1690	17	61.5	0.35995	1.216828	1.757258	Up	3.80E-07	8.26E-07
LOC100909963	563	9.5	34.5	0.602275	2.035515	1.7569	Up	0.000109	0.000203
LOC100361898	1246	107	386.5	3.067355	10.36609	1.756805	Up	0	0
LOC100911275	1650	18	65	0.389667	1.316874	1.756802	Up	1.45E-07	3.20E-07
Dnajc12	1220	248	892.5	7.243887	24.45572	1.755336	Up	7.11E-14	2.79E-13
Rgs8	3661	11.5	41.5	0.112638	0.379815	1.753605	Up	2.34E-05	4.59E-05
Ns5atp9	333	1218	4384.5	130.4179	439.1619	1.751611	Up	4.65E-12	1.29E-11
Dzip3	5778	395	1421.5	2.437904	8.205937	1.751027	Up	5.15E-14	2.05E-13
Med21	791	561.5	2018.5	25.30724	85.13215	1.750154	Up	0	0
Itgb1bp2	1493	12	43.5	0.288376	0.970044	1.750099	Up	2.14E-05	4.20E-05

Arc	3032	45	162.5	0.533164	1.793373	1.750024	Up	2.49E-14	1.02E-13
RGD1562161	2723	553	1990	7.252922	24.39352	1.749864	Up	6.00E-13	1.97E-12
Ifi27l2b	452	132.5	473	10.41661	35.02757	1.749606	Up	1.14E-13	4.37E-13
Srsf11	2654	2811	10112.5	37.81727	127.1341	1.749234	Up	0	0
LOC500066	2303	11	39.5	0.170167	0.571658	1.748199	Up	5.79E-05	0.00011
Zbtb37	1775	17	61	0.341097	1.144842	1.746894	Up	3.80E-07	8.26E-07
RGD1563273	2355	27	97.5	0.409523	1.37404	1.746407	Up	1.54E-10	3.80E-10
Chrnb2	2197	28	100.5	0.456567	1.531554	1.746099	Up	8.90E-11	2.22E-10
Zfp472	2113	104.5	373	1.758424	5.894501	1.745087	Up	0	0
Glb1l2	2436	515	1849	7.558184	25.33498	1.745019	Up	9.85E-13	3.11E-12
Eif1	1074	7316	26182	242.872	813.8903	1.744638	Up	3.22E-11	8.16E-11
Mnat1	1334	1105.5	3964.5	29.60043	99.17568	1.744368	Up	4.00E-12	1.13E-11
Tph1	3897	10	36	0.091528	0.306576	1.743954	Up	0.0001	0.000187
Clcf1	1891	798	2860	15.0738	50.48418	1.743788	Up	1.74E-12	5.25E-12
Bsn	15951	18.5	66	0.041376	0.138539	1.743435	Up	9.55E-08	2.13E-07
Csnk1g1	1648	160	575	3.478972	11.64236	1.742651	Up	8.90E-14	3.46E-13
LOC100912429	2318	335	1197	5.156096	17.24888	1.742152	Up	1.62E-13	6.00E-13
LOC100911807	1781	86.5	309.5	1.733259	5.798129	1.7421	Up	0	0
Camta1	599	43.5	156	2.587606	8.649986	1.74108	Up	1.18E-13	4.49E-13
Zfp958	3217	197	703	2.177539	7.277441	1.740733	Up	0	0
Enc1	4545	3124	11159	24.5403	81.98087	1.740134	Up	0	0
Ccdc130	1590	319	1144	7.192496	24.02284	1.739842	Up	0	0
Mss51	1589	28.5	101.5	0.636929	2.127257	1.739789	Up	6.36E-11	1.59E-10
Trim6	2452	10	36	0.146403	0.488713	1.739047	Up	0.0001	0.000187
LOC100909555	2078	63623.5	228348.5	1096.934	3661.435	1.738933	Up	0	0

Chdh	3435	6.5	23.5	0.068797	0.22959	1.738636	Up	0.001447	0.00243
Creb1	1125	317.5	1134.5	10.08634	33.65794	1.738545	Up	2.57E-13	9.19E-13
Hist1h4b	1296	399.5	1433	11.05275	36.8814	1.738489	Up	0	0
Olr812	933	24.5	88.5	0.94487	3.150999	1.737622	Up	8.15E-10	1.96E-09
Ly96	492	89	318.5	6.488135	21.60864	1.735732	Up	0	0
P2rx1	1119	10.5	37	0.332437	1.10716	1.735713	Up	6.33E-05	0.00012
Tmem126b	870	243.5	863	9.939723	33.09749	1.735444	Up	0	0
Chchd4	1367	161.5	575.5	4.213866	14.02572	1.734858	Up	0	0
RGD1359158	1372	347.5	1236	9.036215	30.06224	1.734162	Up	1.58E-13	5.85E-13
Nrtn	671	8	29	0.431753	1.43624	1.734018	Up	0.000477	0.000847
Srek1	1485	1212.5	4321.5	29.23375	97.20778	1.733437	Up	5.07E-12	1.39E-11
Bbs5	1393	152.5	543.5	3.912669	13.00149	1.732452	Up	1.08E-13	4.15E-13
RGD1310257	3336	211.5	754	2.273186	7.553344	1.7324	Up	2.52E-13	9.04E-13
RGD1307465	2065	233.5	831	4.042442	13.42554	1.731681	Up	0	0
Fam118b	1595	353.5	1253	7.897386	26.22519	1.731506	Up	0	0
Zfp192	8811	199	707	0.80538	2.674177	1.731353	Up	3.57E-14	1.45E-13
LOC360479	1978	7	25	0.126634	0.420157	1.73026	Up	0.001328	0.002237
Kdm3a	4505	2399.5	8515	19.0257	63.12393	1.730238	Up	0	0
Rars2	1886	247	876	4.680878	15.51498	1.72881	Up	0	0
Sowahc	4467	546.5	1936	4.367445	14.47409	1.728612	Up	1.34E-13	5.05E-13
Cwc22	3099	1039	3684	11.97167	39.66806	1.728353	Up	1.33E-13	5.03E-13
Npff	452	6.5	23	0.513948	1.702869	1.728274	Up	0.001447	0.00243
Gas8	1602	122.5	434	2.736055	9.048799	1.725629	Up	4.93E-14	1.96E-13
Haus8	1464	517	1833	12.64479	41.78779	1.724538	Up	5.77E-14	2.28E-13
Ppp2r2a	2374	4278	15136.5	64.42106	212.8557	1.724271	Up	1.17E-11	3.06E-11

Klhl20	3134	964	3403.5	10.98219	36.25596	1.723052	Up	0	0
RGD1561408	9686	13	45.5	0.047849	0.157903	1.722491	Up	1.94E-05	3.83E-05
Cdk17	3670	828.5	2925	8.0539	26.57382	1.722246	Up	0	0
Cflar	1799	1653	5834.5	32.84063	108.2996	1.721474	Up	6.24E-12	1.68E-11
RGD1562259	360	13	46	1.282615	4.226091	1.720236	Up	1.24E-05	2.48E-05
Cdyl	2208	273	960.5	4.416235	14.53151	1.718296	Up	2.09E-13	7.60E-13
LOC100910795	1937	422	1487	7.781621	25.59607	1.71778	Up	4.13E-13	1.41E-12
Spta1	7957	9	32	0.040546	0.133364	1.717747	Up	0.000274	0.000493
RGD1564719	1821	183.5	644.5	3.589931	11.80634	1.717534	Up	3.15E-13	1.11E-12
Zfp566	1719	80	282.5	1.665972	5.478141	1.717322	Up	0	0
Dnaaf2	3409	799.5	2810.5	8.379408	27.52962	1.716065	Up	0	0
Lamc2	5157	319.5	1127	2.219439	7.289652	1.715655	Up	0	0
Grhl2	4871	17	60	0.124885	0.409959	1.714878	Up	5.88E-07	1.26E-06
Zbtb5	4348	364	1277.5	2.990377	9.813524	1.714444	Up	1.34E-13	5.03E-13
Thoc1	1500	628.5	2204.5	14.94826	49.04978	1.71427	Up	5.00E-13	1.68E-12
Spa17	447	133	467.5	10.64435	34.92502	1.714174	Up	1.20E-13	4.54E-13
Pparg	1830	141.5	496.5	2.761049	9.057487	1.713895	Up	0	0
LOC100361891	2121	1654	5799.5	27.82584	91.21629	1.712867	Up	4.25E-12	1.19E-11
Ccdc42b	948	8	28.5	0.305598	1.001344	1.712233	Up	0.000758	0.001302
Dhx15	3007	5906	20675.5	70.07913	229.5231	1.711583	Up	2.83E-11	7.18E-11
Tada1	1455	318	1115	7.802536	25.5523	1.711438	Up	1.88E-13	6.86E-13
LOC688298	3056	316.5	1108.5	3.703795	12.12877	1.711357	Up	3.85E-13	1.32E-12
Zfyve1	3821	433	1515.5	4.050044	13.25437	1.710459	Up	1.10E-12	3.42E-12
Agtrap	2179	944	3312.5	15.49697	50.69388	1.709825	Up	0	0
Kcnmb4	1028	12.5	44	0.435386	1.423754	1.709332	Up	1.36E-05	2.71E-05

Susd1	2711	72.5	255	0.957265	3.130113	1.709225	Up	0	0
Eif2ak2	3808	1154.5	4025.5	10.81517	35.35154	1.708717	Up	6.86E-12	1.83E-11
Slc28a3	2118	10	35	0.168948	0.552143	1.70846	Up	0.000158	0.000288
Fbxo6	876	395	1383.5	16.14888	52.7717	1.70833	Up	5.15E-14	2.05E-13
Syf2	1292	994	3470.5	27.47878	89.77135	1.707937	Up	6.24E-13	2.04E-12
LOC100909777	771	9.5	33	0.437562	1.429008	1.707454	Up	0.000173	0.000315
Poli	2360	162.5	569	2.463037	8.04199	1.707114	Up	0	0
LOC288330	825	14	49	0.60445	1.973156	1.706809	Up	7.21E-06	1.46E-05
LOC100910276	2383	181	634	2.721995	8.88109	1.706073	Up	1.41E-13	5.30E-13
Rims2	5639	97.5	339	0.615398	2.007755	1.705992	Up	5.35E-14	2.12E-13
Disp2	6549	51	178	0.277872	0.906345	1.705639	Up	8.44E-15	3.52E-14
Wdr70	2111	554	1933.5	9.379631	30.58956	1.705436	Up	0	0
RGD1310324	718	80	278.5	3.970218	12.94704	1.705333	Up	0	0
LOC100912221	677	25	87	1.310378	4.273194	1.705331	Up	2.19E-09	5.21E-09
Cdkl3	1731	125	437.5	2.587313	8.435459	1.705012	Up	3.11E-15	1.31E-14
Cabp1	945	8	28	0.302927	0.987334	1.704569	Up	0.000758	0.001302
Bambi	842	243.5	851.5	10.35403	33.7138	1.703147	Up	0	0
Adm2	1042	14.5	50.5	0.497668	1.620234	1.702947	Up	4.62E-06	9.46E-06
LOC680761	767	9.5	33	0.442087	1.438807	1.702471	Up	0.000173	0.000315
7-Mar	2421	2815	9813	41.59295	135.2734	1.701467	Up	0	0
Rybp	4166	2838	9876.5	24.32578	79.10509	1.701284	Up	0	0
LOC100909604	1707	43	149	0.899714	2.925328	1.70106	Up	1.22E-13	4.63E-13
Vsig1	1562	5.5	19.5	0.126548	0.411327	1.700601	Up	0.004105	0.006629
RGD1564941	1792	10.5	36.5	0.209507	0.680284	1.699136	Up	0.0001	0.000187
Rmi1	2417	869.5	3012.5	12.81146	41.59868	1.699103	Up	1.49E-13	5.54E-13

Crem	4015	380	1321	3.379566	10.9718	1.69889	Up	0	0
Cdc14b	3141	610.5	2119	6.930083	22.48664	1.698124	Up	7.45E-13	2.40E-12
Mtmr7	2669	27	94	0.361774	1.173833	1.698068	Up	4.93E-10	1.19E-09
LOC100359523	1467	12	41.5	0.291142	0.944174	1.69733	Up	5.26E-05	0.0001
Pim2	2026	181.5	632.5	3.210042	10.40686	1.696871	Up	1.41E-13	5.30E-13
LOC100360801	5723	11	38.5	0.068778	0.22295	1.696703	Up	9.11E-05	0.00017
LOC100910827	901	358	1235.5	14.11933	45.76033	1.696426	Up	5.04E-13	1.69E-12
LOC100911700	621	200.5	695.5	11.53981	37.38708	1.69592	Up	0	0
Slc22a14	2077	6.5	23	0.112674	0.364948	1.695531	Up	0.001447	0.00243
Pard6b	3294	176	612	1.914004	6.195364	1.694595	Up	0	0
RGD1565983	1878	283.5	980	5.389416	17.43811	1.694043	Up	0	0
Zfp295	3405	651.5	2259.5	6.844525	22.1458	1.694011	Up	2.50E-13	8.95E-13
LOC100910945	673	14	48.5	0.744376	2.408039	1.693755	Up	1.13E-05	2.25E-05
Erbb4	4060	35	121.5	0.309182	1.000135	1.693665	Up	2.13E-12	6.33E-12
Slc34a1	2440	5.5	19	0.081011	0.261824	1.692397	Up	0.004105	0.006628
Cdh10	3169	17	59	0.192863	0.623011	1.691683	Up	9.12E-07	1.94E-06
Fa2h	2506	9	31.5	0.130113	0.420205	1.69133	Up	0.000434	0.000771
Cradd	793	29.5	101.5	1.331516	4.300015	1.691272	Up	1.12E-10	2.77E-10
Fam204a	2115	365	1257	6.139297	19.82351	1.691067	Up	6.76E-13	2.20E-12
RGD1308049	1538	328.5	1144.5	7.669939	24.76427	1.690973	Up	2.05E-13	7.46E-13
Etaa1	2507	502	1724.5	7.12564	22.96969	1.68864	Up	8.49E-13	2.71E-12
Mastl	2989	1855.5	6391	22.16187	71.43196	1.68849	Up	3.27E-12	9.40E-12
LOC100912084	785	18	61.5	0.813202	2.620814	1.688329	Up	8.26E-07	1.76E-06
Sirt1	3787	1004	3456	9.446786	30.43848	1.688001	Up	0	0
Thyn1	1049	687	2370	23.40198	75.37144	1.687387	Up	0	0

Rnf19a	4187	1335.5	4600.5	11.38773	36.6764	1.687372	Up	6.45E-12	1.73E-11
Capn8	2411	13	45	0.194131	0.625013	1.686858	Up	1.94E-05	3.83E-05
Cd207	999	21	72	0.748182	2.406257	1.685329	Up	6.82E-08	1.53E-07
Map2k1ip1	1240	799.5	2749	23.0195	74.00334	1.684734	Up	0	0
Rragd	1473	81.5	280	1.978887	6.361105	1.684589	Up	5.82E-14	2.30E-13
Mynn	2350	349	1199.5	5.294426	17.0141	1.684185	Up	2.05E-13	7.47E-13
RGD1562153	438	6.5	22	0.52252	1.679018	1.68406	Up	0.002332	0.003849
Slc25a36	4484	1802	6183.5	14.32214	46.00787	1.683634	Up	5.19E-12	1.42E-11
Pnisr	4112	1857	6381	16.13548	51.80242	1.682783	Up	6.78E-12	1.81E-11
RGD1561270	2753	215	737.5	2.784343	8.93695	1.682445	Up	0	0
LOC100912787	1674	17.5	60	0.372879	1.19666	1.682234	Up	5.88E-07	1.26E-06
Plekhh1	6378	32	109.5	0.178814	0.573833	1.682171	Up	3.40E-11	8.60E-11
LOC100363314	1288	18	62.5	0.498295	1.598777	1.681896	Up	5.32E-07	1.15E-06
Smyd4	3249	137	470.5	1.51116	4.842766	1.680175	Up	2.87E-13	1.01E-12
LOC100911374	1087	603.5	2065.5	19.81375	63.48207	1.679847	Up	7.03E-13	2.28E-12
Slc6a14	2138	38	131.5	0.639484	2.04823	1.679397	Up	3.76E-13	1.30E-12
Gcat	1555	770.5	2648.5	17.73699	56.79808	1.679081	Up	0	0
Gtse1	2645	3547.5	12202	48.0712	153.9024	1.678771	Up	0	0
Slc7a9	1746	18	62	0.368899	1.180402	1.677979	Up	5.32E-07	1.15E-06
Casp3	2484	1772	6060	25.46235	81.44403	1.677444	Up	7.60E-13	2.44E-12
LOC100912222	4094	7	24	0.061603	0.19704	1.677418	Up	0.002112	0.003492
Ctxn3	1502	95	324	2.251419	7.198353	1.676833	Up	0	0
Thrb	6243	42	144	0.240549	0.768941	1.676541	Up	1.28E-13	4.85E-13
Mbd4	1892	73.5	251.5	1.389948	4.442565	1.676362	Up	0	0
Tnfrsf9	1410	103.5	356.5	2.637419	8.429608	1.676339	Up	0	0

LOC301861	1173	7.5	26	0.226593	0.723151	1.674193	Up	0.000831	0.001423
Abhd13	5283	804.5	2748.5	5.436783	17.35079	1.674176	Up	1.07E-13	4.12E-13
Paip2	1681	1505.5	5131.5	31.94147	101.9115	1.673814	Up	3.96E-12	1.11E-11
Mmp2	3053	67.5	232.5	0.791428	2.524724	1.673596	Up	0	0
Abcc3	5174	17	58	0.117904	0.376002	1.673129	Up	1.42E-06	2.99E-06
Fam76b	3729	844	2874	8.068212	25.7221	1.672687	Up	8.56E-13	2.73E-12
Ccdc19	1836	36	122	0.696635	2.219494	1.671756	Up	2.76E-12	8.04E-12
Slc25a44	3678	493	1686	4.796705	15.28131	1.671653	Up	4.28E-13	1.46E-12
Nup37	1369	447	1520	11.64651	37.08843	1.671072	Up	8.15E-13	2.61E-12
Henmt1	1693	22	75	0.463637	1.47638	1.670996	Up	3.99E-08	9.04E-08
Spata5	3084	396	1346.5	4.578743	14.57973	1.670941	Up	0	0
Pip5k1a	3518	2213.5	7552.5	22.5161	71.68162	1.670646	Up	0	0
LOC679765	898	7.5	25.5	0.299177	0.951574	1.669317	Up	0.001328	0.002237
Myh7b	6097	55.5	189	0.325682	1.035839	1.669264	Up	0	0
Zfp830	1490	342	1163.5	8.197554	26.06692	1.668955	Up	2.29E-13	8.24E-13
Zfp509	2685	85.5	291.5	1.14	3.622162	1.667817	Up	0	0
Zfand5	2755	2496.5	8440.5	32.19415	102.2534	1.667279	Up	0	0
LOC681647	1998	131	445	2.340121	7.427893	1.66637	Up	8.62E-14	3.36E-13
Rec8	2049	7.5	25.5	0.130838	0.415282	1.666309	Up	0.001328	0.002237
LOC690217	981	502.5	1705	18.29046	58.00317	1.66504	Up	8.49E-13	2.71E-12
Gpr63	2421	19	65	0.281064	0.890435	1.663613	Up	3.10E-07	6.76E-07
Tpd52	924	669	2267	25.9024	82.02533	1.662983	Up	1.58E-12	4.81E-12
Myom2	4513	8	27	0.063177	0.199943	1.662113	Up	0.001198	0.002022
Rell2	1764	76.5	261	1.556538	4.926109	1.662108	Up	0	0
Zfp951	1942	53	180	0.975691	3.082505	1.659608	Up	4.04E-14	1.62E-13

Fbxo8	1646	439.5	1485.5	9.543889	30.13758	1.658914	Up	0	0
Vsig10l	2764	56	189.5	0.724571	2.287862	1.658801	Up	1.58E-14	6.48E-14
MGC94335	1417	549.5	1854	13.83777	43.67879	1.658321	Up	4.66E-14	1.86E-13
Pinx1	996	393	1330	14.13541	44.61442	1.658196	Up	0	0
LOC680767	339	9.5	32	1.000238	3.156867	1.658151	Up	0.000274	0.000493
LOC100911887	3305	114.5	386.5	1.238564	3.906975	1.657384	Up	7.79E-14	3.06E-13
RGD1560978	1669	36	121.5	0.770463	2.430226	1.657293	Up	4.14E-12	1.16E-11
Trpt1	2882	71.5	242	0.887851	2.80035	1.657217	Up	0	0
Fam162a	537	2487.5	8432	166.1801	524.0687	1.657008	Up	0	0
Slc20a2	3655	1733.5	5863.5	16.97644	53.52392	1.65665	Up	2.65E-12	7.76E-12
Slc22a13	2029	6	20.5	0.106381	0.335327	1.656335	Up	0.005941	0.009493
Lsm1	1216	495	1667	14.52628	45.77847	1.656004	Up	4.63E-13	1.56E-12
Tuft1	2388	738	2488	11.03751	34.78002	1.655845	Up	1.08E-12	3.38E-12
Clec2d	702	133.5	450.5	6.791453	21.39545	1.655512	Up	1.20E-13	4.54E-13
Tmod4	1375	9	30.5	0.235469	0.741562	1.655032	Up	0.000682	0.001176
RGD1309077	1472	190.5	638.5	4.602671	14.49085	1.654599	Up	2.69E-13	9.58E-13
Sptbn2	8178	25	85	0.10988	0.345803	1.654028	Up	5.16E-09	1.21E-08
Zfp667	3396	45	152	0.475848	1.49567	1.652219	Up	3.04E-14	1.24E-13
Tp53rk	2312	111.5	375	1.720869	5.402013	1.65036	Up	0	0
Rap1gap2	3803	1263.5	4249.5	11.88334	37.29448	1.650022	Up	4.51E-12	1.25E-11
Fam122a	1331	376	1271	10.13743	31.8128	1.649916	Up	4.39E-13	1.49E-12
Arhgef9	3787	177.5	597.5	1.67651	5.260884	1.649844	Up	0	0
Bin2a	2607	16	54	0.221373	0.694664	1.649836	Up	3.79E-06	7.82E-06
Fam103a1	812	401.5	1346	17.67601	55.4662	1.649816	Up	0	0
LOC690073	7481	937	3148.5	4.478423	14.04302	1.64879	Up	0	0

Fam178b	1721	231.5	778.5	4.820872	15.09923	1.647109	Up	0	0
Catsper2	2234	59	198.5	0.948886	2.970382	1.646341	Up	2.53E-14	1.03E-13
Zwint	1810	2110	7068.5	41.66125	130.3458	1.645566	Up	1.05E-12	3.29E-12
Cwf19l1	2310	993	3321	15.34913	47.97753	1.644202	Up	1.91E-12	5.73E-12
Prelid2	842	29	97.5	1.237206	3.866585	1.643974	Up	6.07E-10	1.47E-09
Rapgef2	6311	3661.5	12249.5	20.73478	64.79052	1.64373	Up	0	0
LOC691020	783	7.5	25.5	0.343117	1.071792	1.643251	Up	0.001328	0.002237
LOC100911550	2346	1426	4776	21.7633	67.92532	1.642052	Up	1.67E-12	5.06E-12
Tmc7	2181	93	310.5	1.525307	4.757889	1.641222	Up	3.69E-14	1.49E-13
Apoo	906	268	891	10.5102	32.7814	1.641087	Up	1.39E-13	5.23E-13
LOC100366035	976	27.5	92.5	1.011468	3.153616	1.640556	Up	1.15E-09	2.75E-09
Defb29	816	96	322	4.211198	13.12617	1.640143	Up	0	0
RGD1308297	4713	2110.5	7035	15.98342	49.81959	1.640137	Up	1.05E-12	3.29E-12
Fam82a1	2012	123	408.5	2.179563	6.786413	1.63861	Up	0	0
LOC310177	675	45	149	2.371956	7.384497	1.638422	Up	4.60E-14	1.84E-13
LOC100151767	2330	1142	3798.5	17.50049	54.4262	1.636906	Up	2.46E-12	7.25E-12
Pde4d	2470	123	408.5	1.779132	5.529135	1.63588	Up	0	0
Med26	3617	383	1277	3.794912	11.79012	1.63544	Up	1.49E-13	5.54E-13
Tsnaxip1	2590	29.5	98.5	0.40923	1.270946	1.634919	Up	3.95E-10	9.60E-10
Lag3	1817	9	30	0.176927	0.549259	1.634336	Up	0.000682	0.001176
LOC100911616	744	10	33	0.477104	1.479657	1.632888	Up	0.00039	0.000694
LOC679894	4945	241	796	1.735702	5.380122	1.63212	Up	2.12E-13	7.71E-13
LOC363391	1046	27	89.5	0.925304	2.867467	1.631778	Up	4.20E-09	9.88E-09
Golga7b	2849	7	23.5	0.088926	0.275549	1.631636	Up	0.003336	0.00544
Nfatc2	3701	168.5	560	1.631549	5.054692	1.631381	Up	1.69E-13	6.23E-13

Cxcl12	1880	790	2616	15.00454	46.43872	1.629929	Up	8.43E-13	2.70E-12
Ptpn2	1494	994.5	3292.5	23.79481	73.57084	1.628487	Up	6.24E-13	2.04E-12
RGD1565647	4141	607	2000	5.215162	16.11283	1.627426	Up	0	0
RGD1311429	4858	1602	5304.5	11.79417	36.42789	1.62697	Up	3.38E-12	9.68E-12
Prpf3	2331	1384.5	4590.5	21.27575	65.7115	1.626936	Up	3.21E-12	9.24E-12
Snip1	2338	343.5	1133	5.243431	16.19203	1.626701	Up	0	0
Uspl1	3115	700.5	2309.5	8.016637	24.74834	1.626263	Up	0	0
Dot1l	6015	1758.5	5834.5	10.48701	32.37408	1.626236	Up	4.98E-12	1.37E-11
Abl2	3764	1761	5829.5	16.74176	51.65108	1.625347	Up	3.10E-12	8.96E-12
LOC689756	762	759.5	2513	35.71061	110.117	1.624613	Up	1.87E-12	5.61E-12
Gpt	1744	66	219.5	1.356812	4.18323	1.624396	Up	0	0
Irs2	6301	2498.5	8271.5	14.20826	43.79474	1.624027	Up	0	0
RGD1306746	2521	206	677.5	2.913969	8.976563	1.623178	Up	2.46E-13	8.84E-13
Slc30a6	2023	371.5	1224	6.557703	20.19464	1.62271	Up	5.35E-13	1.78E-12
Fosl2	984	899	2974	32.7668	100.8246	1.621541	Up	0	0
Col7a1	9170	206.5	680	0.805086	2.476677	1.62119	Up	2.46E-13	8.84E-13
Mterfd1	1450	742	2450.5	18.3412	56.4054	1.620745	Up	4.00E-13	1.37E-12
Taf1a	1534	173.5	572.5	4.041023	12.42001	1.619874	Up	3.79E-13	1.30E-12
MGC125002	1556	382	1252.5	8.741711	26.83562	1.618162	Up	5.33E-13	1.78E-12
Lct	6117	8	26	0.046611	0.143086	1.618143	Up	0.001883	0.003135
Basp1	1024	2220	7340.5	77.82893	238.8586	1.617779	Up	0	0
Ptprn2	5136	94.5	310	0.657446	2.017623	1.617713	Up	0	0
RGD1309906	1289	303.5	996	8.408617	25.80471	1.617694	Up	2.56E-13	9.16E-13
RGD1311783	2144	1290.5	4240.5	21.48822	65.93466	1.617491	Up	7.67E-12	2.04E-11
Ccng1	3198	13360	43784.5	148.9379	456.8522	1.617016	Up	0	0

Sync	2156	82.5	272	1.369921	4.201211	1.616712	Up	0	0
Sub1	782	2290	7482.5	104.301	319.5009	1.615066	Up	0	0
Sclt1	2319	446.5	1461.5	6.874245	21.04773	1.614391	Up	0	0
LOC100909737	795	95.5	315	4.30462	13.17666	1.614027	Up	0	0
Pde4b	3133	1222.5	4018.5	13.98672	42.79111	1.613253	Up	5.44E-12	1.48E-11
LOC498592	759	190.5	623	8.969457	27.43173	1.612753	Up	2.69E-13	9.58E-13
Mrpl47	1960	388.5	1265	7.044035	21.53389	1.612135	Up	6.64E-13	2.16E-12
LOC100361068	969	10	32.5	0.370464	1.132324	1.611883	Up	0.000608	0.001051
LOC100364435	210	94.5	313	16.00279	48.90507	1.611661	Up	0	0
Sarnp	1244	1960	6401.5	56.21555	171.747	1.611244	Up	0	0
Slc15a4	2730	590	1932	7.730852	23.61209	1.610827	Up	0	0
Slco2a1	3392	365.5	1198.5	3.859067	11.7856	1.610701	Up	6.76E-13	2.20E-12
RGD1562794	2514	16	52	0.226369	0.691294	1.610625	Up	9.14E-06	1.84E-05
Rnf25	1466	932	3056	22.76578	69.50982	1.61035	Up	1.55E-12	4.72E-12
RGD1565082	3746	339.5	1110	3.239743	9.890166	1.610115	Up	2.04E-13	7.42E-13
Hccs	2398	709	2313.5	10.53889	32.16961	1.609976	Up	1.51E-12	4.61E-12
Slc25a33	1416	238	777	6.003884	18.30314	1.608123	Up	6.71E-13	2.19E-12
Zfp330	1920	1041	3399	19.3836	59.07696	1.607759	Up	2.83E-12	8.24E-12
Cbx5	1190	1801.5	5879	54.13366	164.9837	1.607725	Up	2.65E-12	7.75E-12
Leng1	1846	227	742.5	4.400524	13.41011	1.607573	Up	3.55E-15	1.49E-14
Cpne5	4308	13	42.5	0.107848	0.328642	1.607519	Up	7.36E-05	0.000139
Ncald	3270	308	1004.5	3.367778	10.26052	1.607235	Up	0	0
Rab9b	3846	39	127.5	0.362707	1.104594	1.606638	Up	3.04E-12	8.81E-12
Il18rap	1884	425.5	1385	8.065941	24.56398	1.606629	Up	1.53E-12	4.67E-12
LOC679983	471	28	92	2.136981	6.502306	1.605378	Up	2.46E-09	5.83E-09

Trmt12	1655	159.5	518.5	3.441839	10.47209	1.605298	Up	8.53E-14	3.32E-13
Pacrgl	1572	185.5	603	4.208833	12.79189	1.603737	Up	0	0
Acot2	2400	271.5	886.5	4.048337	12.30357	1.603675	Up	8.22E-14	3.21E-13
Gtf3c6	1553	519	1686.5	11.9437	36.29073	1.603352	Up	0	0
Rfx3	2441	227	740	3.324126	10.0985	1.603094	Up	3.55E-15	1.49E-14
LOC100911166	2123	58.5	189.5	0.983994	2.987542	1.602238	Up	1.35E-14	5.59E-14
Efha2	1865	187	606.5	3.57531	10.85139	1.60174	Up	3.37E-13	1.18E-12
Ankra2	1658	147	478.5	3.170158	9.618901	1.601318	Up	1.02E-13	3.94E-13
Pclo	15961	52	169.5	0.116041	0.35199	1.600903	Up	5.20E-14	2.06E-13
Ccng2	2506	869.5	2818.5	12.38277	37.55502	1.600671	Up	1.49E-13	5.54E-13
Vgll4	1356	781	2539.5	20.62404	62.544	1.600544	Up	1.94E-13	7.09E-13
RGD1311249	2329	569.5	1853	8.764565	26.55357	1.599151	Up	0	0
Phlda3	1511	6883	22404	163.172	494.2721	1.598912	Up	1.97E-11	5.07E-11
Arid4b	4980	388	1257.5	2.780907	8.41939	1.59816	Up	6.64E-13	2.16E-12
Dnali1	2250	10.5	34	0.166606	0.503712	1.596157	Up	0.000249	0.000448
Tsen15	987	575.5	1862	20.85548	62.99417	1.594791	Up	3.54E-13	1.23E-12
Bloc1s4	1301	395	1279.5	10.87085	32.83403	1.594727	Up	5.15E-14	2.05E-13
Rab12	1984	2129	6871.5	38.26462	115.5524	1.594464	Up	0	0
Cited2	1996	5295.5	17200	95.23586	287.2276	1.592618	Up	1.36E-11	3.54E-11
Ist1	2261	2438	7869.5	38.53173	116.2071	1.592579	Up	0	0
LOC690068	900	221	710	8.727517	26.31148	1.59205	Up	4.91E-14	1.95E-13
RGD1305045	914	1505.5	4839	58.62277	176.6085	1.591022	Up	3.96E-12	1.12E-11
LOC500974	2392	345	1111	5.149296	15.51152	1.590893	Up	9.33E-13	2.96E-12
Vcam1	3007	86	276.5	1.019584	3.069838	1.590182	Up	0	0
Alkbh8	2467	252.5	810	3.643972	10.97151	1.590179	Up	1.17E-13	4.46E-13

RGD1564405	2013	120.5	387	2.138456	6.434723	1.589309	Up	0	0
Scube2-ps1	3543	9	29	0.090088	0.27099	1.588832	Up	0.001068	0.001808
Btg4	1140	16	51.5	0.502221	1.510238	1.588382	Up	1.41E-05	2.81E-05
RGD1563351	1005	8	25.5	0.2837	0.852949	1.588091	Up	0.00294	0.004808
Lsg1	2283	1132.5	3641	17.72029	53.25264	1.587451	Up	4.49E-12	1.25E-11
LOC100912596	521	177	574.5	12.24584	36.77979	1.586622	Up	0	0
Iqcb1	2844	257.5	829.5	3.237549	9.722155	1.586374	Up	5.38E-13	1.79E-12
Krt10	1581	13	42	0.29387	0.882382	1.586225	Up	7.36E-05	0.000139
LOC100909598	483	96	309.5	7.126442	21.39662	1.586129	Up	0	0
RGD1562378	1075	43	139.5	1.438797	4.313683	1.584057	Up	4.65E-13	1.57E-12
Reps2-ps1	2027	11	35	0.194469	0.582703	1.583219	Up	0.000347	0.00062
LOC100360595	395	19.5	62	1.749819	5.24274	1.583115	Up	1.15E-06	2.44E-06
Wdr45	1478	345.5	1109.5	8.363784	25.05448	1.582841	Up	9.33E-13	2.96E-12
LOC100359722	1155	190	609.5	5.879469	17.61101	1.58272	Up	2.69E-13	9.57E-13
Arhgap12	4891	814	2603	5.935874	17.77313	1.582165	Up	0	0
Tfam	1463	1270	4063.5	30.98194	92.74057	1.581773	Up	5.97E-12	1.61E-11
RGD1307461	2597	23.5	75.5	0.325013	0.972874	1.581754	Up	8.58E-08	1.92E-07
Rtp1	825	16.5	52.5	0.711842	2.129525	1.580903	Up	9.14E-06	1.84E-05
Gtf2f2	1481	645.5	2062.5	15.55697	46.52225	1.58036	Up	5.31E-13	1.77E-12
Rsbn1l	3392	741.5	2374	7.820363	23.36577	1.579089	Up	0	0
RGD1309759	1571	1481	4741	33.70516	100.7016	1.579046	Up	5.28E-12	1.44E-11
Gpatch3	1813	66	211	1.304542	3.895542	1.578281	Up	0	0
LOC100363943	1939	138.5	441.5	2.546038	7.600839	1.577905	Up	1.83E-13	6.72E-13
RGD1564927	2908	166	531.5	2.043436	6.098913	1.577556	Up	2.16E-13	7.83E-13
Zfp967	2975	499	1591.5	5.990203	17.86039	1.576087	Up	0	0

Atp6v1b1	2487	99.5	316	1.422509	4.239619	1.575496	Up	1.62E-14	6.66E-14
Gin1	1866	254	809.5	4.856679	14.46853	1.574876	Up	3.19E-13	1.12E-12
Rchy1	1718	995.5	3164.5	20.6404	61.44372	1.573794	Up	0	0
Zfp800	2402	557	1772.5	8.281066	24.6166	1.571743	Up	5.01E-13	1.68E-12
LOC316820	2942	234.5	743.5	2.837093	8.430606	1.571223	Up	4.49E-13	1.52E-12
Epb41l4a	2048	62	197.5	1.084401	3.222093	1.571099	Up	0	0
Sik1	2650	364	1164.5	4.930711	14.64939	1.570973	Up	1.34E-13	5.03E-13
Mucdhl	2712	23	73.5	0.304529	0.904676	1.570823	Up	2.03E-07	4.47E-07
Zkscan1	3168	495.5	1579	5.600126	16.62992	1.57025	Up	4.63E-13	1.56E-12
Suhw3	2214	318	1011.5	5.136489	15.24617	1.569592	Up	1.88E-13	6.86E-13
LOC100910349	1585	65	207	1.468537	4.358593	1.569483	Up	0	0
Utp23	992	267.5	850.5	9.626953	28.56687	1.569192	Up	7.08E-13	2.29E-12
Bcl10	1668	1084	3444.5	23.2139	68.88089	1.569115	Up	3.69E-12	1.05E-11
LOC100909970	967	7	22	0.257252	0.763299	1.569068	Up	0.005232	0.008391
LOC100188936	1469	409.5	1303	9.972825	29.58401	1.568744	Up	4.45E-13	1.51E-12
Zdbf2	7238	14	44.5	0.068896	0.204209	1.567546	Up	6.54E-05	0.000124
LOC100909529	861	306	967	12.64879	37.48983	1.5675	Up	0	0
C1qtnf2	1216	45	143	1.324687	3.925505	1.567227	Up	3.06E-13	1.08E-12
Zwilch	3105	2595	8209.5	29.85611	88.3162	1.564652	Up	0	0
Arf2	2201	1068.5	3366.5	17.27953	51.08685	1.563888	Up	3.46E-12	9.90E-12
Mcm8	3226	360.5	1138.5	3.982095	11.76969	1.563477	Up	4.37E-13	1.48E-12
Tbxa2r	1880	52	166	0.989139	2.922132	1.562776	Up	5.42E-14	2.15E-13
Hemk1	1553	110	348	2.531965	7.474443	1.561709	Up	0	0
Akirin2	1482	1636	5162	39.4335	116.2196	1.55936	Up	2.92E-12	8.48E-12
Trim7	1686	94.5	299	2.007514	5.915039	1.558977	Up	0	0

Uchl5	1824	657	2069.5	12.8579	37.8758	1.558621	Up	0	0
Ccdc59	1539	421	1326.5	9.771889	28.77307	1.55801	Up	4.13E-13	1.41E-12
Ppp6c	1867	1318.5	4153	25.21853	74.19701	1.556877	Up	4.07E-12	1.14E-11
Ash1l	11394	3404.5	10710.5	10.66127	31.36644	1.556842	Up	0	0
Sfrp1	3506	26	82.5	0.264709	0.778055	1.555464	Up	4.02E-08	9.10E-08
Ythdf3	4150	3391	10662	29.15657	85.67554	1.555062	Up	0	0
LOC24906	1607	11	35	0.245295	0.720434	1.554349	Up	0.000347	0.00062
Serpinb1a	1289	55.5	174	1.537369	4.51521	1.55433	Up	0	0
Arg2	1463	18	57	0.44065	1.294018	1.554151	Up	4.72E-06	9.68E-06
Ggct	1875	368.5	1159.5	7.039173	20.66825	1.553938	Up	8.46E-14	3.30E-13
Eny2	2048	1110.5	3474	19.28215	56.60776	1.553734	Up	4.75E-12	1.31E-11
Atl2	2870	1058.5	3322	13.15015	38.60355	1.553654	Up	1.94E-12	5.82E-12
Ociad1	1373	3724	11682	96.85805	283.8617	1.551244	Up	4.10E-12	1.15E-11
Hspa1b	5918	532	1673	3.219552	9.433936	1.551	Up	0	0
Il7	1128	11	34.5	0.349458	1.023932	1.550927	Up	0.000536	0.000945
LOC100363276	5414	8	25	0.052451	0.15367	1.550783	Up	0.00294	0.004807
LOC100360437	1066	35	109.5	1.172182	3.434152	1.550757	Up	3.19E-10	7.78E-10
Ppil4	3463	1189.5	3728.5	12.27332	35.94533	1.550279	Up	3.07E-12	8.87E-12
Ccl28	1418	11	34.5	0.277585	0.81262	1.549651	Up	0.000536	0.000945
Rsf1	4975	1085.5	3403	7.796109	22.81247	1.548996	Up	3.86E-12	1.09E-11
LOC100361658	3579	604.5	1893	6.036086	17.66193	1.548957	Up	6.80E-13	2.21E-12
Cyp21a1	1963	36	112	0.651857	1.906736	1.548478	Up	1.87E-10	4.60E-10
Nfat5	4593	4222	13208.5	32.82542	96.00824	1.548345	Up	1.15E-11	3.01E-11
RGD1309374	2244	238	744	3.783436	11.06381	1.548079	Up	6.71E-13	2.19E-12
Erich1	1108	274	863	8.87894	25.95967	1.547813	Up	1.86E-13	6.82E-13

Mgea5	2751	2417.5	7547.5	31.34721	91.62792	1.54745	Up	0	0
Cryab	706	2665.5	8349	135.0804	394.8085	1.547335	Up	0	0
Chmp4c	1616	22.5	70	0.493784	1.442312	1.546432	Up	3.49E-07	7.60E-07
Bcas2	1083	1143	3568	37.62961	109.9123	1.546413	Up	1.73E-12	5.22E-12
RGD1565025	1107	9	28	0.291957	0.85179	1.544743	Up	0.001661	0.002773
Gpr149	3845	13.5	42	0.124369	0.362821	1.544628	Up	7.36E-05	0.000139
Mbip	1590	663.5	2069.5	14.92155	43.46526	1.542466	Up	2.84E-13	1.01E-12
Rbm4b	1830	289	902	5.641613	16.42869	1.542038	Up	0	0
RGD1563917	1206	8	25	0.238795	0.695083	1.541416	Up	0.00294	0.004806
Trim23	3308	668.5	2079.5	7.202596	20.96107	1.541124	Up	4.14E-13	1.41E-12
Spata2L	3029	95	296.5	1.124748	3.271852	1.540506	Up	0	0
Tmem69	1968	752	2340.5	13.64996	39.68907	1.539845	Up	2.42E-12	7.13E-12
Tbcel	1566	80	248.5	1.823613	5.302282	1.539814	Up	0	0
LOC100912357	787	453.5	1397.5	20.40511	59.32658	1.539748	Up	1.56E-12	4.77E-12
Elac1	2836	107.5	334	1.353854	3.934871	1.539244	Up	0	0
Zcchc3	3057	348.5	1085.5	4.079969	11.85448	1.538802	Up	4.24E-14	1.70E-13
Zfp799	4600	157	487.5	1.218679	3.540135	1.538486	Up	0	0
Ahsa2	3698	1305	4066	12.61914	36.64548	1.538021	Up	7.23E-12	1.93E-11
Chuk	3526	1658	5143	16.75139	48.64137	1.537903	Up	7.21E-12	1.92E-11
Nfkb2	3098	1334	4154	15.41679	44.75704	1.537612	Up	1.66E-12	5.04E-12
Mrps18a	852	850.5	2647	35.74884	103.7222	1.536756	Up	2.20E-12	6.54E-12
Star	1154	14	43	0.432124	1.252628	1.53544	Up	0.000101	0.000188
P4ha1	2145	6424	19945.5	107.1662	310.6443	1.535414	Up	2.55E-11	6.52E-11
Anapc13	616	428	1327.5	24.85772	72.04975	1.535299	Up	2.52E-13	9.04E-13
Afmid	2511	298.5	924	4.253002	12.31761	1.534169	Up	5.55E-13	1.84E-12

Aasdh	3683	255	789.5	2.472076	7.158984	1.534032	Up	0	0
Arl6ip6	1745	1092	3379.5	22.31976	64.62153	1.533693	Up	3.40E-12	9.73E-12
Tlr6	2820	195	602	2.461562	7.126617	1.533644	Up	1.92E-13	7.02E-13
LOC690581	2594	400	1237	5.495077	15.9052	1.533287	Up	0	0
Phkg1	1388	12	37	0.306886	0.888049	1.532935	Up	0.000306	0.000549
Poc5	2329	429.5	1330.5	6.587477	19.06204	1.532905	Up	0	0
Smc5	5932	3439.5	10634	20.68529	59.85132	1.532778	Up	0	0
Adora2a	2485	11	34	0.158627	0.458975	1.532773	Up	0.000536	0.000945
RGD1560314	2199	48	149	0.779776	2.255268	1.532167	Up	3.08E-13	1.08E-12
Ccdc14	2790	376.5	1169	4.832204	13.97379	1.53197	Up	4.39E-13	1.49E-12
RGD1561851	936	39	121	1.483004	4.288144	1.531831	Up	3.76E-11	9.47E-11
LOC686234	1727	242	747.5	4.998273	14.44904	1.531472	Up	3.84E-13	1.32E-12
Sptbn5	10860	23.5	73	0.077352	0.223512	1.530834	Up	2.03E-07	4.47E-07
Ptprr	3578	31	96	0.310188	0.896292	1.530825	Up	4.16E-09	9.78E-09
RGD1309139	2401	11	34	0.164416	0.475032	1.530675	Up	0.000536	0.000945
Lepr	3650	29	90	0.285719	0.825209	1.530162	Up	1.22E-08	2.82E-08
Rsph6a	2352	10.5	32.5	0.160112	0.462297	1.529735	Up	0.000608	0.001051
LOC100911963	1158	12.5	38.5	0.387004	1.11584	1.52771	Up	0.000199	0.00036
Mterfd2	1323	369	1138.5	9.962153	28.71552	1.527301	Up	0	0
Fcn1	1291	6.5	20	0.179941	0.518618	1.527146	Up	0.005941	0.009494
Pold3	1743	627.5	1936.5	12.87695	37.09301	1.526357	Up	6.79E-13	2.20E-12
Pfkfb3	2148	230	710	3.837945	11.0537	1.526123	Up	3.86E-13	1.32E-12
Fpgt	1797	353.5	1092	7.029748	20.24541	1.52605	Up	0	0
LOC100910046	2052	63.5	195	1.102716	3.174916	1.525657	Up	0	0
Rnf32	1959	116.5	358	2.124338	6.113262	1.524929	Up	9.68E-14	3.74E-13

Map2k3	2356	1570	4852	23.87068	68.693	1.524924	Up	6.35E-12	1.70E-11
RGD1566091	307	33.5	103.5	3.91309	11.25435	1.524102	Up	9.32E-10	2.24E-09
Polb	1196	464	1429	13.87718	39.90615	1.523896	Up	0	0
Ppp4r2	4848	4648	14289	34.18898	98.31372	1.523861	Up	2.40E-11	6.13E-11
Taco1	1386	96	295	2.471873	7.107438	1.523725	Up	0	0
Hus1	4469	573	1759.5	4.580691	13.14973	1.521396	Up	7.09E-13	2.29E-12
Fastkd3	2286	354	1085	5.52167	15.84986	1.521294	Up	0	0
LOC680322	389	15	46.5	1.38129	3.959733	1.519387	Up	5.79E-05	0.00011
Tmem33	2263	1725.5	5281	27.15552	77.84001	1.519266	Up	3.79E-12	1.07E-11
Ddx52	2175	1229.5	3770	20.19129	57.83861	1.5183	Up	3.28E-12	9.42E-12
Inhba	1552	1100	3369	25.31749	72.48825	1.517613	Up	3.55E-12	1.01E-11
Rspo1	1705	63.5	195	1.328485	3.803652	1.517603	Up	0	0
Serac1	2141	176	540.5	2.938332	8.411967	1.517446	Up	0	0
Atpaf2	1462	317.5	974.5	7.763334	22.22236	1.517264	Up	2.57E-13	9.19E-13
LOC688133	5384	567	1737	3.759459	10.75797	1.516809	Up	0	0
Xpa	1204	23.5	72	0.694855	1.988331	1.516774	Up	3.11E-07	6.79E-07
Actl7b	1416	13.5	41.5	0.343786	0.982629	1.515136	Up	0.000114	0.000212
Cox7b	473	1634.5	4957	122.5524	350.2106	1.514824	Up	5.32E-12	1.45E-11
MGC125086	1109	58	177.5	1.873509	5.352218	1.514394	Up	1.55E-14	6.40E-14
Tra2b	1978	6504.5	19873	117.4751	335.4333	1.513671	Up	1.08E-11	2.84E-11
Med20	1813	473.5	1448.5	9.345325	26.67669	1.513263	Up	5.88E-13	1.94E-12
Brix1	2456	1654	5041.5	24.02173	68.51737	1.512129	Up	4.25E-12	1.19E-11
Lypla1	2356	2716	8260	41.0246	116.9923	1.511852	Up	0	0
Wrap53	1782	546	1672.5	10.99156	31.32289	1.510821	Up	1.34E-13	5.05E-13
LOC365839	1320	17.5	53.5	0.473313	1.348746	1.510752	Up	1.25E-05	2.51E-05

Olr780	939	9.5	29	0.359276	1.023448	1.510272	Up	0.001068	0.001808
Tatdn3	1232	203	619	5.893774	16.7769	1.509212	Up	1.92E-13	7.01E-13
Mospd1	2220	365	1106	5.852799	16.6369	1.507188	Up	6.76E-13	2.20E-12
LOC500584	1320	327.5	996.5	8.874779	25.22531	1.507089	Up	6.83E-13	2.22E-12
Zfx	3072	578	1749.5	6.685053	18.99573	1.506664	Up	1.55E-13	5.73E-13
Smek2	5014	2718.5	8254.5	19.33898	54.94617	1.506507	Up	0	0
LOC100362170	1503	23	69.5	0.546436	1.552518	1.506484	Up	1.10E-06	2.34E-06
Gpx2	1028	8.5	26	0.297268	0.843538	1.504691	Up	0.001883	0.003135
Pwwp2a	2897	299	908.5	3.688247	10.46343	1.504349	Up	6.08E-13	2.00E-12
Tmem53	1291	31	94	0.859685	2.437925	1.503773	Up	9.61E-09	2.23E-08
Zcrb1	1326	1010	3066	27.22889	77.11543	1.501881	Up	0	0
RGD1305834	5622	406.5	1233	2.585632	7.321174	1.501558	Up	0	0
Commd3	952	2306	6995.5	86.60554	245.1808	1.501315	Up	0	0
Nfkb1	3723	2450	7419	23.52852	66.56341	1.500319	Up	0	0
Pigh	2370	159.5	483.5	2.408317	6.8129	1.500244	Up	8.53E-14	3.33E-13
Ublcp1	2115	914.5	2766.5	15.42492	43.63057	1.500077	Up	0	0
Ccnh	1400	1235	3734.5	31.46639	88.99857	1.49997	Up	6.33E-12	1.70E-11
Metrnl	1380	939	2848.5	24.35356	68.85049	1.499334	Up	0	0
LOC684762	735	54	164	2.639895	7.454472	1.497626	Up	6.53E-14	2.57E-13
Kcnj13	1268	9	27	0.252173	0.711628	1.496709	Up	0.002566	0.004218
LOC100911710	1587	11	33	0.246218	0.694811	1.496683	Up	0.000825	0.001414
Trpm2	4527	15	45	0.117426	0.33128	1.496301	Up	8.85E-05	0.000165
Mpp5	2703	831.5	2509.5	10.98339	30.98293	1.49615	Up	0	0
Cxcr7	1958	304	919	5.545764	15.63538	1.495356	Up	0	0
Ptafr	3370	14	42	0.148654	0.419035	1.495107	Up	0.000154	0.000281

Arhgap4	3195	69	208.5	0.771707	2.175243	1.495052	Up	0	0
Notch4	6483	51.5	156	0.285274	0.80394	1.49474	Up	1.03E-13	3.97E-13
Adhfe1	1884	34	102	0.642117	1.808522	1.493902	Up	2.90E-09	6.86E-09
LOC688765	1899	164	492.5	3.082735	8.681757	1.493776	Up	1.50E-13	5.56E-13
Cabyr	2076	26	78	0.446495	1.257019	1.493291	Up	2.17E-07	4.76E-07
Znrd1	659	248.5	748.5	13.42768	37.79754	1.493082	Up	7.11E-14	2.79E-13
Dclre1a	3540	579.5	1748	5.858302	16.48134	1.492279	Up	9.45E-13	3.00E-12
Csgalnact2	3636	1474	4432	14.44184	40.62883	1.49225	Up	4.34E-12	1.21E-11
Morc3	3847	1366	4103	12.662	35.62025	1.492192	Up	1.59E-12	4.85E-12
Pdcd7	2255	252.5	759.5	4.000031	11.23793	1.490293	Up	1.17E-13	4.46E-13
Tpcn2	2879	262	789.5	3.25661	9.145098	1.489628	Up	1.18E-13	4.49E-13
Akirin1	2200	2112	6352.5	34.30707	96.32935	1.489469	Up	0	0
Fam179b	6552	945	2833.5	5.145552	14.44056	1.488729	Up	2.03E-12	6.06E-12
LOC684189	1327	149.5	448	4.024646	11.29253	1.488436	Up	2.66E-13	9.48E-13
Mfap3	1932	286.5	859	5.293453	14.8476	1.487949	Up	3.49E-14	1.41E-13
LOC688310	791	25	75	1.131674	3.173592	1.487658	Up	3.72E-07	8.09E-07
LOC100910750	828	837.5	2508.5	36.08056	101.125	1.486846	Up	8.45E-13	2.70E-12
Dcdc5	2859	44	131.5	0.546291	1.53075	1.486497	Up	2.07E-11	5.32E-11
Pkp2	2861	1522	4579.5	19.05527	53.39341	1.486472	Up	4.68E-12	1.29E-11
Akr1c12	1237	344	1031	9.924613	27.78828	1.485394	Up	5.51E-13	1.83E-12
Snx2	2049	2229	6679	38.84079	108.7371	1.4852	Up	0	0
Epha4	6338	152.5	457	0.859857	2.40607	1.48451	Up	1.08E-13	4.15E-13
RGD1566380	1418	352.5	1062	8.924253	24.97052	1.484422	Up	6.43E-13	2.10E-12
Aftph	3967	1689	5053	15.20726	42.54443	1.48421	Up	0	0
Nfkbil1	1479	280	844	6.802294	19.02621	1.483895	Up	0	0

LOC501738	681	8.5	25.5	0.442002	1.234968	1.482348	Up	0.00294	0.004807
Shcbp1	2005	1549	4623	27.55634	76.97084	1.481928	Up	2.00E-12	5.98E-12
Rpf2	1226	1006.5	3007	29.32127	81.89637	1.481852	Up	1.84E-12	5.53E-12
Skil	2327	2589.5	7734	39.76276	111.0148	1.481263	Up	0	0
Glb1l3	2331	32.5	97.5	0.498785	1.391662	1.480318	Up	5.61E-09	1.32E-08
Mob3c	942	24	71	0.905587	2.523104	1.478274	Up	9.70E-07	2.06E-06
Nfyb	2569	904	2694.5	12.57785	35.03375	1.477861	Up	0	0
RGD1564776	1721	66	197	1.37228	3.82121	1.477455	Up	0	0
LOC100361713	1252	62.5	185.5	1.777372	4.947048	1.476822	Up	0	0
Ap1s3	2535	547.5	1629	7.692478	21.40691	1.476556	Up	0	0
Zfp40	3424	137.5	406.5	1.431027	3.980926	1.476053	Up	2.87E-13	1.01E-12
RGD1306595	1310	135.5	401.5	3.684828	10.24676	1.475499	Up	2.15E-13	7.78E-13
LOC100911828	532	54	161.5	3.642912	10.12598	1.474897	Up	1.36E-13	5.11E-13
LOC100912347	1191	10	30	0.302373	0.839466	1.473144	Up	0.001452	0.002438
LOC100362820	11276	15	44.5	0.047347	0.13132	1.47175	Up	0.000135	0.000246
Capns2	943	9	26.5	0.338475	0.938707	1.471625	Up	0.003936	0.006371
Cript	1435	880.5	2605	21.8513	60.59369	1.471449	Up	1.52E-12	4.65E-12
Tet2	5810	427.5	1271.5	2.634165	7.300633	1.470676	Up	1.02E-12	3.21E-12
Slc16a3	2118	16	48	0.2714	0.751542	1.469433	Up	5.09E-05	9.72E-05
Htr2a	1566	332.5	984.5	7.58026	20.98676	1.46916	Up	0	0
Scfd1	2095	1828.5	5412.5	31.16304	86.23249	1.468396	Up	8.71E-13	2.78E-12
Upp1	1153	211	626	6.547368	18.11192	1.467952	Up	2.52E-13	9.04E-13
LOC498824	2156	156.5	464	2.598369	7.181113	1.466601	Up	1.64E-13	6.07E-13
Dusp14	1371	271	805.5	7.086929	19.5858	1.466575	Up	8.22E-14	3.21E-13
Asb3	2232	244	720.5	3.900483	10.77601	1.466098	Up	2.85E-13	1.01E-12

LOC100909405	2203	379	1120.5	6.148011	16.9836	1.465951	Up	4.40E-14	1.76E-13
Hspb2	549	7.5	22.5	0.490409	1.354377	1.465573	Up	0.005232	0.00839
Mrs2	1949	275	813	5.044235	13.92641	1.465116	Up	5.96E-13	1.97E-12
Ppil1	1325	974	2886.5	26.37113	72.80248	1.465028	Up	3.08E-13	1.08E-12
RGD1562502	1629	1341.5	3963	29.42513	81.22943	1.464954	Up	4.62E-12	1.28E-11
Chm	3482	851.5	2516	8.725438	24.08383	1.464766	Up	1.69E-12	5.12E-12
Prom2	4294	33	97	0.274599	0.757341	1.463618	Up	1.13E-08	2.62E-08
Espnl	3000	112	333	1.341642	3.700236	1.463618	Up	0	0
Lyrm1	1685	100	295.5	2.126357	5.864423	1.463606	Up	1.75E-14	7.19E-14
Ccdc115	1100	256.5	759	8.337483	22.99353	1.463544	Up	0	0
Bhlhe40	2388	4080	12059.5	61.13646	168.5805	1.463333	Up	1.72E-11	4.45E-11
Zfp131	2455	1145.5	3385	16.67854	45.98927	1.463304	Up	4.30E-12	1.20E-11
Cog4	2773	1267.5	3742.5	16.34	45.04901	1.463088	Up	3.13E-12	9.03E-12
E4f1	2595	366.5	1083	5.050574	13.92328	1.46298	Up	0	0
Tdp2	1966	441.5	1299.5	8.000318	22.0529	1.462839	Up	3.59E-13	1.25E-12
Lmbrd1	2576	912.5	2688	12.63269	34.80836	1.462272	Up	0	0
Ddx28	1931	237.5	702.5	4.403027	12.13008	1.462021	Up	2.75E-13	9.80E-13
Narf	3053	635.5	1876.5	7.444265	20.5078	1.461971	Up	1.34E-12	4.13E-12
Cyld	3267	1047	3082	11.43402	31.48493	1.461328	Up	3.61E-12	1.03E-11
Trpc2	4233	133.5	394	1.12968	3.109923	1.460965	Up	1.20E-13	4.54E-13
Gpn3	1176	388.5	1146	11.8054	32.48689	1.460411	Up	6.64E-13	2.16E-12
Cep63	2795	513.5	1512.5	6.56028	18.04633	1.459876	Up	0	0
Cdk5rap1	1975	327.5	966.5	5.934692	16.3163	1.459069	Up	6.83E-13	2.21E-12
LOC100360950	323	14	41	1.543875	4.243527	1.458708	Up	0.000235	0.000424
Ifngr1	1860	1578.5	4649	30.34129	83.38037	1.458425	Up	2.08E-12	6.19E-12

LOC311026	5717	553	1630	3.462582	9.509306	1.457492	Up	6.00E-13	1.97E-12
RGD1563556	3714	100	293	0.9593	2.634151	1.457284	Up	1.75E-14	7.19E-14
RGD1564519	1099	1645.5	4812.5	53.30245	146.3267	1.45692	Up	5.18E-12	1.42E-11
Hs3st2	2292	9	26.5	0.139259	0.382286	1.456882	Up	0.003936	0.006371
Kitlg	5276	4282.5	12548	28.92609	79.38753	1.456541	Up	2.28E-11	5.83E-11
Ttc22	2553	9	27	0.127268	0.349214	1.456237	Up	0.002566	0.004218
Dhrs7b	1235	693.5	2038.5	20.07718	55.07754	1.455908	Up	1.17E-12	3.63E-12
LOC100910487	879	22	64	0.89038	2.442157	1.455664	Up	4.35E-06	8.92E-06
Mertk	3024	43	126	0.507495	1.391712	1.455396	Up	7.98E-11	1.99E-10
RGD1309058	1892	18	53	0.341039	0.934779	1.454689	Up	2.57E-05	5.04E-05
RGD1309095	1949	555.5	1622	10.14723	27.794	1.453688	Up	0	0
Rabgef1	2826	701	2052.5	8.851614	24.23725	1.453214	Up	1.61E-13	5.97E-13
Pan3	3083	1314.5	3853	15.23208	41.6927	1.452682	Up	4.51E-12	1.25E-11
Ido2	1252	152.5	446.5	4.3611	11.92649	1.451405	Up	1.08E-13	4.15E-13
RGD1310553	3465	1088.5	3178.5	11.1916	30.60613	1.451405	Up	2.48E-12	7.29E-12
LOC100912163	5840	601.5	1758.5	3.674308	10.04773	1.451326	Up	1.08E-13	4.17E-13
Snapc1	1692	365	1065.5	7.698862	21.03871	1.450329	Up	6.76E-13	2.20E-12
Zfp207	1815	4482.5	13150.5	88.46331	241.7209	1.450191	Up	1.90E-11	4.89E-11
Adcy7	4164	10	29	0.085384	0.233169	1.449343	Up	0.002223	0.003672
Trappc6b	1165	759.5	2217.5	23.27382	63.53528	1.44885	Up	1.87E-12	5.61E-12
Mettl20	1632	138	404	3.024031	8.253843	1.448593	Up	1.83E-13	6.73E-13
Fosl1	1663	3136.5	9203.5	67.67083	184.6985	1.448566	Up	0	0
Sdccag8	2550	224	653	3.134766	8.554584	1.44834	Up	7.97E-14	3.12E-13
Dhrs9	1636	3278	9580.5	71.63496	195.4563	1.44811	Up	0	0
Zfp287	5339	267.5	780.5	1.790109	4.884053	1.448031	Up	7.08E-13	2.29E-12

Ero1l	2361	3438	10026	51.98307	141.8238	1.447986	Up	0	0
Jazf1	2861	236.5	690	2.953053	8.056449	1.447937	Up	4.41E-13	1.49E-12
Blzf1	2206	402	1173.5	6.51325	17.76753	1.447794	Up	1.18E-14	4.87E-14
Shroom4	4741	451.5	1319.5	3.405026	9.28708	1.447559	Up	2.05E-12	6.11E-12
Taf13	1727	1378.5	4006	28.40746	77.44764	1.446952	Up	4.03E-12	1.13E-11
Fgf9	1065	57	166	1.918928	5.229307	1.446319	Up	1.56E-13	5.79E-13
Fastkd5-ps1	2975	455.5	1325	5.457252	14.85947	1.445136	Up	3.90E-13	1.34E-12
Azin1	4269	5225.5	15201.5	43.64658	118.8296	1.444953	Up	1.70E-11	4.41E-11
Ppp2r3c	1704	563.5	1638.5	11.78899	32.09263	1.444801	Up	1.35E-13	5.07E-13
Tcf7l2	4017	1122	3270.5	10.00086	27.21631	1.444348	Up	2.95E-12	8.57E-12
RGD1562747	1038	232	675.5	7.988955	21.7328	1.443795	Up	3.80E-13	1.31E-12
Tmem135	3420	481.5	1403.5	5.03631	13.69326	1.443027	Up	3.30E-13	1.15E-12
Zfp111	2817	268	779	3.398598	9.239981	1.44295	Up	1.39E-13	5.23E-13
LOC100910342	814	284	824.5	12.44441	33.83019	1.442813	Up	1.18E-13	4.50E-13
LOC100910558	1574	34.5	100	0.78086	2.121716	1.442096	Up	6.60E-09	1.54E-08
Arhgef3	3617	256.5	746	2.534639	6.885723	1.441828	Up	0	0
Zbtb9	2786	146.5	427	1.879683	5.10609	1.44173	Up	3.33E-14	1.35E-13
Gpr132	2402	39	113.5	0.581232	1.57854	1.441405	Up	1.02E-09	2.44E-09
Pdzrn4	3368	20	58.5	0.212149	0.576033	1.441073	Up	1.30E-05	2.59E-05
RGD1563941	415	26	75	2.228023	6.044605	1.439884	Up	7.44E-07	1.59E-06
Nup54	2277	1059	3069.5	16.60726	45.01031	1.438441	Up	2.72E-12	7.95E-12
Krit1	3045	1538	4458.5	18.04439	48.90179	1.438337	Up	1.46E-12	4.47E-12
LOC688411	1380	7.5	22	0.193851	0.525079	1.437589	Up	0.005232	0.00839
Hist1h2bh	949	307.5	893.5	11.60162	31.42336	1.437511	Up	4.91E-14	1.95E-13
Gtf2b	1227	1220.5	3542	35.58269	96.33935	1.43695	Up	7.20E-12	1.92E-11

Ftsjd1	3553	381	1107.5	3.836348	10.38352	1.43649	Up	3.02E-13	1.06E-12
Vhl	2807	663	1917.5	8.428131	22.80747	1.436222	Up	2.84E-13	1.01E-12
Dnase1l2	1209	12	35	0.35422	0.958344	1.435898	Up	0.000718	0.001235
Ccndbp1	1452	337	974	8.274652	22.37228	1.434942	Up	0	0
Fscn2	1710	20	58	0.418517	1.131366	1.434706	Up	1.30E-05	2.59E-05
Zfp410	3345	983	2844	10.50161	28.37901	1.434213	Up	3.73E-13	1.29E-12
MGC114464	1463	25.5	73.5	0.618408	1.670868	1.433967	Up	8.51E-07	1.82E-06
Efcab7	2157	628	1812.5	10.37372	28.02677	1.433873	Up	5.00E-13	1.68E-12
Golph3l	1920	253	731.5	4.705331	12.70901	1.433484	Up	3.97E-13	1.36E-12
Orc5	2115	607	1752.5	10.25262	27.69108	1.433429	Up	0	0
Wdr53	1459	273	789.5	6.684949	18.05314	1.433262	Up	2.09E-13	7.60E-13
Galr2	1159	40	116	1.234969	3.333796	1.432691	Up	5.95E-10	1.44E-09
Sgip1	4233	470.5	1362	3.974818	10.72928	1.432593	Up	1.65E-12	5.01E-12
Piga	759	105.5	304.5	4.971178	13.41507	1.432194	Up	0	0
Alpk1	3719	32	93	0.308204	0.831265	1.431423	Up	2.91E-08	6.63E-08
Mipol1	1875	273	786.5	5.191383	13.99865	1.431097	Up	2.09E-13	7.61E-13
Gnpda2	1825	189	545	3.699172	9.972774	1.430792	Up	2.87E-13	1.02E-12
LOC290876	1454	26.5	75.5	0.642902	1.732706	1.430355	Up	7.44E-07	1.59E-06
Cgref1	1349	236	681.5	6.259226	16.86899	1.430317	Up	4.41E-13	1.49E-12
LOC362473	1326	14	40.5	0.373045	1.00514	1.429975	Up	0.000355	0.000635
Zfand1	1621	563.5	1618.5	12.36149	33.30117	1.42972	Up	1.35E-13	5.08E-13
Crybb1	1006	18.5	53.5	0.660607	1.779567	1.429663	Up	2.57E-05	5.04E-05
Brd8	3318	1792.5	5167	19.33663	52.0219	1.427783	Up	0	0
Celf3	3234	33.5	97	0.37182	1.000006	1.427333	Up	1.13E-08	2.62E-08
Smad3	4677	1953.5	5638	14.94388	40.18707	1.427177	Up	1.94E-12	5.81E-12

Timm10	1167	264	758.5	8.05512	21.66105	1.427125	Up	6.88E-14	2.71E-13
Rpusd2	3169	136.5	393	1.5376	4.134748	1.427119	Up	1.95E-13	7.11E-13
Rps27l	698	4150	11915.5	211.7178	569.1382	1.426636	Up	3.29E-11	8.33E-11
Cnot7	2614	1569	4502.5	21.3858	57.48132	1.42644	Up	0	0
RGD1564036	1173	102.5	293.5	3.12415	8.396403	1.426308	Up	0	0
Gosr1	2726	1042.5	3003.5	13.67937	36.76388	1.426287	Up	1.99E-12	5.95E-12
Inha	1471	10	28.5	0.239749	0.644102	1.425762	Up	0.003379	0.005507
Prss36	2742	44	127	0.575875	1.54669	1.425356	Up	1.05E-10	2.60E-10
Tmsbl1	412	351	1003.5	30.27924	81.29445	1.424828	Up	9.48E-14	3.68E-13
Iws1	3010	1525.5	4385.5	18.10036	48.58715	1.424556	Up	8.43E-12	2.23E-11
Bet1	1455	864.5	2473.5	21.15114	56.74737	1.423818	Up	0	0
Ypel5	2144	744	2129.5	12.36168	33.15577	1.423385	Up	0	0
Arhgap10	2618	836	2403	11.43074	30.65527	1.423216	Up	9.49E-13	3.01E-12
Uba5	1463	2055.5	5913.5	50.27998	134.7905	1.422663	Up	0	0
Tor1aip2	2289	3751	10760	58.51172	156.8288	1.422393	Up	6.74E-13	2.19E-12
Klhl24	4636	503	1440.5	3.869199	10.36901	1.422172	Up	3.35E-13	1.17E-12
LOC100912083	968	55	157.5	2.018328	5.408545	1.42208	Up	9.73E-13	3.08E-12
LOC687679	424	1079	3083	90.59093	242.7567	1.422073	Up	2.91E-12	8.45E-12
LOC685574	2185	161	463.5	2.637971	7.066836	1.421635	Up	0	0
Llph	1102	658	1882.5	21.314	57.08024	1.42119	Up	1.51E-12	4.63E-12
LOC682147	1818	214	615.5	4.212739	11.28137	1.421112	Up	0	0
LOC100911263	801	52.5	150	2.338542	6.262325	1.421089	Up	2.25E-12	6.66E-12
Eno2	2222	942.5	2700	15.15886	40.59212	1.421039	Up	1.62E-13	5.99E-13
LOC688324	3077	324.5	931.5	3.773415	10.09768	1.420081	Up	0	0
LOC682138	2196	9	26	0.147436	0.39447	1.419829	Up	0.003936	0.00637

Mphosph8	2877	1214	3472	15.07025	40.30977	1.419426	Up	4.15E-12	1.16E-11
Brf2	1869	226.5	651.5	4.342781	11.60747	1.418362	Up	3.18E-14	1.29E-13
Cyr61	1987	15554	44611.5	280.3998	749.1121	1.417696	Up	0	0
RGD1307399	1615	927	2642.5	20.4687	54.64526	1.416677	Up	2.08E-12	6.19E-12
Socs6	2944	966	2760	11.72306	31.28088	1.415931	Up	0	0
Mllt11	1406	472.5	1347.5	11.99859	32.01144	1.415723	Up	1.71E-13	6.32E-13
Med28	2089	1112.5	3181	19.03788	50.78681	1.415581	Up	4.84E-12	1.33E-11
RGD1560492	3091	92.5	261.5	1.057384	2.820317	1.415358	Up	6.66E-15	2.79E-14
Ap4e1	3448	300	855.5	3.105407	8.282067	1.415208	Up	2.14E-13	7.76E-13
LOC100363723	969	20	56.5	0.729092	1.944381	1.415139	Up	2.93E-05	5.70E-05
Ppp1r36	1463	13.5	39	0.331566	0.883859	1.414521	Up	0.000269	0.000483
Plag1	3360	265.5	756	2.822138	7.518729	1.4137	Up	6.59E-13	2.15E-12
Acyp1	552	412	1166	26.50209	70.55131	1.412567	Up	2.14E-13	7.77E-13
H2afj	510	141.5	404	9.92978	26.41759	1.411665	Up	0	0
LOC100359967	3451	177	503.5	1.828657	4.864998	1.411655	Up	0	0
Rngtt	4139	1189	3386.5	10.26841	27.31826	1.411652	Up	3.07E-12	8.87E-12
Pfkfb2	1862	213.5	611.5	4.114206	10.945	1.411586	Up	2.80E-13	9.94E-13
Dnajb6	2474	2314	6597	33.48665	89.05283	1.411075	Up	0	0
Efcab1	1198	16	46	0.475992	1.265436	1.410627	Up	0.000117	0.000216
Clca4l	2899	13	37	0.160265	0.425806	1.409736	Up	0.000621	0.001073
Riok1	2085	757.5	2154.5	12.98237	34.48352	1.409353	Up	8.99E-13	2.86E-12
LOC296884	2452	307.5	875.5	4.490421	11.927	1.409309	Up	4.91E-14	1.95E-13
LOC680430	395	473.5	1363	43.15227	114.6155	1.409294	Up	5.88E-13	1.94E-12
Nkrf	1689	128	363.5	2.705024	7.184047	1.409156	Up	3.69E-14	1.48E-13
Clcnka	2394	11	31.5	0.166334	0.441744	1.409129	Up	0.001915	0.003187

Nup35	1539	1660	4727	38.60219	102.4941	1.408787	Up	3.43E-12	9.82E-12
Krt23	1269	8.5	24	0.238101	0.632138	1.408665	Up	0.004557	0.007328
Smarcad1	4795	1503.5	4262.5	11.18506	29.68858	1.408335	Up	2.81E-12	8.20E-12
Fbxo38	4381	1106.5	3142	9.021269	23.94318	1.408212	Up	2.42E-12	7.13E-12
Jarid1a	9470	2959	8402	11.15269	29.59997	1.408204	Up	0	0
Magohb	630	248	700.5	13.99781	37.14653	1.408027	Up	7.11E-14	2.79E-13
Recql5	3905	255	723.5	2.330216	6.178952	1.406898	Up	0	0
Snrpg	462	714.5	2024	55.15577	146.2446	1.4068	Up	5.15E-13	1.72E-12
Itga2	4379	400	1131	3.257883	8.635005	1.406262	Up	0	0
Ap4b1	2921	459	1301.5	5.613089	14.87713	1.406229	Up	1.20E-12	3.71E-12
Col27a1	5568	524	1489	3.374946	8.944822	1.406188	Up	0	0
Ucn2	911	83	236	3.26394	8.647077	1.405598	Up	1.43E-13	5.34E-13
Ccdc85b	1561	1027	2934	23.62669	62.58603	1.405423	Up	0	0
Mmadhc	1332	1005	2843	26.8983	71.23934	1.405159	Up	8.95E-13	2.85E-12
LOC100912594	3169	206	582.5	2.314499	6.124256	1.403835	Up	2.46E-13	8.83E-13
RGD1565370	483	13	37	0.96311	2.548265	1.403743	Up	0.000621	0.001073
Thumpd2	1876	155	440	2.958338	7.826757	1.403628	Up	3.73E-13	1.29E-12
Pla1a	1743	22.5	64	0.461424	1.220227	1.402984	Up	4.35E-06	8.92E-06
LOC100910555	1625	17	48.5	0.374701	0.990652	1.402641	Up	0.000101	0.000189
Ptprcap	860	9	25.5	0.375142	0.991528	1.402214	Up	0.005991	0.00957
RGD1309870	747	40.5	115	1.948115	5.148305	1.402018	Up	8.90E-10	2.14E-09
Tk2	2389	250	706	3.734982	9.868769	1.401769	Up	2.66E-13	9.47E-13
Pced1a	1137	399	1130	12.57382	33.19947	1.400737	Up	0	0
Gpatc2	1392	248	702	6.380941	16.84249	1.400264	Up	7.11E-14	2.79E-13
Snx14	2955	862	2431	10.40685	27.46583	1.400104	Up	0	0

Snrpa1	1040	1260.5	3554	43.24287	114.1077	1.399862	Up	2.88E-12	8.37E-12
Ctnnal1	3643	158.5	447	1.553004	4.095362	1.39893	Up	1.62E-14	6.66E-14
LOC686779	3415	163	460.5	1.703757	4.492204	1.398704	Up	2.37E-13	8.51E-13
Fbxo24	2095	14	39.5	0.238577	0.628844	1.398249	Up	0.000536	0.000944
Syap1	1491	1243.5	3512.5	29.80009	78.54374	1.39818	Up	7.86E-12	2.08E-11
Hist2h4	396	16.5	47.5	1.506173	3.969503	1.398071	Up	7.74E-05	0.000145
Rab27a	2704	70	196.5	0.9221	2.429719	1.397795	Up	0	0
Pidd	3321	169	477.5	1.820775	4.796222	1.397346	Up	8.88E-15	3.70E-14
Slc22a5	3037	410.5	1154.5	4.83527	12.71657	1.395041	Up	1.10E-12	3.45E-12
Kat8	1558	473	1334.5	10.86432	28.57224	1.395016	Up	5.88E-13	1.94E-12
RGD1306286	9804	825	2322.5	3.00862	7.910987	1.394756	Up	0	0
Tasp1	1990	136.5	384.5	2.452029	6.446572	1.394556	Up	1.95E-13	7.10E-13
Slc33a1	1994	916	2574	16.3857	43.07781	1.394508	Up	0	0
RGD1304595	3795	265	745.5	2.492351	6.551836	1.394392	Up	6.59E-13	2.15E-12
Gpatch4	1903	510.5	1436.5	9.58944	25.2031	1.394083	Up	0	0
LOC100910054	3332	534	1499.5	5.721	15.03601	1.394082	Up	0	0
Il2rg	1611	35.5	100	0.788697	2.072428	1.393778	Up	1.30E-08	3.00E-08
Bud13	2207	565	1592.5	9.174928	24.05883	1.390798	Up	4.35E-13	1.48E-12
LOC100911956	1077	618.5	1733.5	20.5174	53.79795	1.390703	Up	2.89E-15	1.22E-14
Macrod2	1144	53	149.5	1.6618	4.357149	1.390638	Up	6.38E-12	1.71E-11
Kdm5b	6276	1292.5	3621.5	7.347342	19.26157	1.390431	Up	5.07E-12	1.39E-11
LOC100362237	333	26	73	2.776666	7.278489	1.390285	Up	1.67E-06	3.50E-06
LOC100912086	358	428.5	1190	42.36622	111.0388	1.390078	Up	2.52E-13	9.04E-13
Tmco1	1181	1846	5187	55.8928	146.4632	1.389804	Up	4.55E-12	1.26E-11
Dbr1	1942	919	2575	16.89774	44.24848	1.388798	Up	1.36E-12	4.18E-12

Pcmtd2	3547	647	1810.5	6.505311	17.03444	1.388765	Up	0	0
Pet112l	1825	547.5	1536	10.73293	28.09882	1.388466	Up	0	0
Snapc4	4352	267.5	753	2.206106	5.775475	1.388438	Up	7.08E-13	2.29E-12
LOC691280	1377	300.5	839.5	7.768718	20.33593	1.388283	Up	2.14E-13	7.75E-13
Gpr176	3824	940	2634.5	8.781692	22.97968	1.387788	Up	0	0
Braf	3906	659	1845.5	6.025857	15.76751	1.387717	Up	0	0
Tsga10	3030	27	75.5	0.318293	0.832658	1.38737	Up	1.45E-06	3.05E-06
Vldlr	3417	346	967	3.611942	9.44678	1.387048	Up	3.04E-13	1.07E-12
LOC100912122	549	456	1273.5	29.6631	77.56204	1.386681	Up	1.95E-12	5.82E-12
Atrip	2549	208.5	583	2.922567	7.64002	1.386341	Up	3.49E-13	1.21E-12
LOC100911690	765	33.5	93.5	1.556859	4.064727	1.384521	Up	5.69E-08	1.28E-07
Prkab2	1758	121.5	338	2.467365	6.441606	1.384449	Up	0	0
LOC100910318	858	276	765.5	11.42247	29.81731	1.384276	Up	7.04E-14	2.77E-13
Cdh17	3411	103.5	287	1.081484	2.822608	1.384017	Up	0	0
Gmeb2	1964	399	1121	7.288294	19.02195	1.384012	Up	0	0
Plekhd1	3303	46	129	0.499212	1.302573	1.383641	Up	1.78E-10	4.37E-10
Rufy3	1903	159.5	443.5	2.985462	7.785865	1.382903	Up	8.53E-14	3.33E-13
Ehf	2359	13.5	38	0.205387	0.535523	1.382606	Up	0.00041	0.000729
Fbxl12	1926	219.5	612	4.06753	10.59114	1.380632	Up	3.38E-13	1.18E-12
Rab40c	846	110	308	4.654022	12.11777	1.380575	Up	0	0
LOC100910762	1728	131.5	367	2.720599	7.0825	1.380335	Up	8.62E-14	3.36E-13
Dusp5	2436	809.5	2262	11.90079	30.97802	1.380188	Up	1.03E-12	3.24E-12
C1d	3129	683	1895.5	7.766985	20.21443	1.379959	Up	6.82E-13	2.21E-12
Smc4	4241	8386.5	23335	70.61108	183.6791	1.379221	Up	0	0
Ap3m2	1584	263.5	736.5	5.957102	15.49604	1.379217	Up	1.80E-13	6.60E-13

Akip1	1783	403.5	1125.5	8.09033	21.04504	1.37921	Up	0	0
LOC686151	636	89	248	4.992074	12.98338	1.378954	Up	0	0
Armc7	2370	149	417	2.254986	5.862996	1.378519	Up	2.66E-13	9.48E-13
Ikzf5	2778	775	2162	9.990361	25.95957	1.377658	Up	0	0
Ap4s1	892	200.5	557.5	8.023597	20.84809	1.377594	Up	0	0
RGD1308907	3000	392	1093	4.67797	12.15403	1.37748	Up	0	0
Plagl2	3502	405	1124.5	4.128606	10.72611	1.3774	Up	0	0
Nox1	2577	26	72.5	0.360136	0.935271	1.376843	Up	2.48E-06	5.18E-06
RGD1565690	2691	21	58.5	0.279458	0.725633	1.376609	Up	2.53E-05	4.96E-05
Morn2	651	39	109.5	2.156909	5.599855	1.376424	Up	5.03E-09	1.18E-08
Fancl	1653	479.5	1327.5	10.33919	26.81817	1.375088	Up	0	0
LOC100912117	872	97.5	269.5	3.978962	10.31903	1.374843	Up	5.35E-14	2.12E-13
Tcerg1	4366	5202	14440	42.58981	110.4275	1.374519	Up	3.14E-11	7.95E-11
Zfp7	571	10	27.5	0.629689	1.630802	1.372869	Up	0.005099	0.008182
Agpat9	2017	420	1161.5	7.4284	19.23467	1.372586	Up	1.49E-13	5.54E-13
Eef1e1	998	1027.5	2845	36.79183	95.22724	1.371989	Up	0	0
Epha1	3248	16	44.5	0.176272	0.456177	1.37179	Up	0.000264	0.000475
Gatc	1967	954	2644.5	17.34825	44.88016	1.371287	Up	1.35E-12	4.16E-12
RGD1307218	1795	17.5	48.5	0.349341	0.902847	1.369846	Up	0.000101	0.000189
LOC688276	2684	9	25	0.119775	0.309303	1.368697	Up	0.005991	0.009572
RGD1561507	559	20	55	1.278207	3.299419	1.36809	Up	4.37E-05	8.43E-05
LOC100910152	799	161.5	448	7.250365	18.711	1.367761	Up	0	0
Srgn	1320	27	74.5	0.730627	1.884672	1.367108	Up	2.15E-06	4.48E-06
Ina	4593	29.5	80	0.227894	0.587796	1.366952	Up	7.29E-07	1.56E-06
RGD1561694	1070	405	1113	13.48463	34.7689	1.366481	Up	0	0

Unc50	1197	626.5	1722.5	18.64841	48.06703	1.365995	Up	0	0
LOC100909470	563	162.5	447	10.29204	26.51102	1.365063	Up	0	0
Pggt1b	1568	509.5	1401	11.5821	29.82521	1.364635	Up	0	0
LOC679578	960	1233.5	3407.5	46.04493	118.5128	1.363929	Up	4.71E-12	1.30E-11
RGD1311345	2580	1236.5	3408	17.12793	44.08037	1.363786	Up	2.72E-12	7.94E-12
Hells	3164	783	2150.5	8.828328	22.71375	1.363354	Up	0	0
Rpl30	495	1230.5	3371.5	88.44407	227.4422	1.362663	Up	4.82E-12	1.33E-11
Cxadrl1	1381	161	437.5	4.125182	10.60723	1.362518	Up	0	0
LOC691532	384	25	70.5	2.337104	6.008902	1.362379	Up	2.87E-06	5.96E-06
Bola2	327	14.5	39.5	1.568305	4.031587	1.362142	Up	0.000536	0.000944
Ddx20	2752	929	2554.5	12.05819	30.98047	1.361346	Up	1.35E-12	4.16E-12
Perld1	2328	14	38.5	0.216669	0.55659	1.361122	Up	0.000802	0.001376
Kbtbd2	3434	1783.5	4900.5	18.53455	47.6095	1.361032	Up	1.64E-14	6.74E-14
LOC100364990	1673	421	1154	8.965553	23.0236	1.360649	Up	4.13E-13	1.41E-12
Fyttd1	3220	3508	9619	38.83288	99.71632	1.360551	Up	0	0
Tia1	1435	321.5	884.5	7.999158	20.53713	1.360315	Up	0	0
Polq	8527	557	1527	2.330368	5.979062	1.359361	Up	5.01E-13	1.68E-12
Aox3l1	4360	27	74	0.220673	0.566038	1.358989	Up	2.15E-06	4.49E-06
Ctf2p	615	10.5	28.5	0.606737	1.555243	1.357996	Up	0.003379	0.005508
Fhl2	840	962.5	2647	41.05111	105.2253	1.357989	Up	0	0
Wdyhv1	1442	146	399.5	3.615832	9.268088	1.357944	Up	3.33E-14	1.35E-13
RGD1565766	747	110.5	300.5	5.240652	13.4301	1.357652	Up	0	0
Eif3e	1502	3160	8641.5	74.9764	192.0904	1.357277	Up	0	0
Cd47	1952	3107	8501.5	56.77054	145.4427	1.357236	Up	0	0
Kif26b	7439	31	85.5	0.149888	0.383912	1.356894	Up	3.68E-07	8.00E-07

Zbtb34	1763	68	187	1.384085	3.544886	1.356807	Up	0	0
LOC304131	1681	36	99	0.76701	1.96359	1.356177	Up	3.72E-08	8.45E-08
Dbh	2250	14.5	40	0.230476	0.589943	1.355962	Up	0.000355	0.000635
Dennd1b	2753	455	1245	5.893842	15.07576	1.354951	Up	3.90E-13	1.34E-12
Usp15	3247	2303	6299.5	25.31746	64.74003	1.354525	Up	0	0
Arrdc2	2318	204.5	562.5	3.166909	8.09777	1.354449	Up	1.72E-13	6.33E-13
LOC679491	801	98	267	4.355065	11.12894	1.35355	Up	0	0
Adam8	3081	37	102	0.431399	1.102387	1.353536	Up	2.18E-08	4.98E-08
Fancb	2657	399.5	1088	5.356647	13.68222	1.3529	Up	0	0
Sft2d1	995	758	2064.5	27.12225	69.26123	1.352571	Up	0	0
Zfp426	3029	216	588.5	2.544153	6.494846	1.35211	Up	2.34E-13	8.42E-13
Ube2t	989	558	1519.5	20.09983	51.3078	1.351995	Up	1.47E-13	5.48E-13
Slfnl1	1766	51	139	1.032728	2.635485	1.351608	Up	8.77E-11	2.18E-10
Duoxa2	963	10	27.5	0.371581	0.947339	1.350204	Up	0.005099	0.008182
LOC100910503	631	84.5	230	4.777805	12.17808	1.349867	Up	0	0
Nr6a1	1614	27	73.5	0.596117	1.518451	1.348933	Up	3.18E-06	6.58E-06
Figf	1491	24.5	67	0.587411	1.496054	1.348721	Up	4.94E-06	1.01E-05
Ube2v2	3260	2484	6759.5	27.18881	69.24065	1.348606	Up	0	0
Tceb1	2156	1759.5	4762	28.95395	73.73126	1.348516	Up	1.96E-12	5.87E-12
Map4k5	3306	2177	5933.5	23.5263	59.90808	1.348476	Up	0	0
Pyroxd1	1781	249	679.5	5.0025	12.72509	1.346955	Up	1.13E-14	4.69E-14
Ppih	753	669.5	1821.5	31.75241	80.74984	1.346593	Up	1.58E-12	4.81E-12
Abcf3	2536	991.5	2702.5	13.99187	35.58041	1.346494	Up	2.91E-12	8.45E-12
Nol8	4617	477	1297	3.686719	9.37195	1.346012	Up	0	0
Lsr	2097	28	76.5	0.478339	1.215613	1.345578	Up	1.85E-06	3.88E-06

Ccar1	4438	2809.5	7638	22.62376	57.47989	1.345219	Up	0	0
LOC100911652	1412	148	399	3.720598	9.447761	1.344438	Up	0	0
Slc11a1	2012	37.5	102	0.664226	1.685862	1.343739	Up	2.18E-08	4.98E-08
Tmem42	1306	59	161	1.619621	4.10963	1.343352	Up	2.76E-12	8.05E-12
Terf2ip	2165	367	995.5	6.06553	15.38731	1.343035	Up	4.71E-13	1.59E-12
Sstr3	3985	28.5	76.5	0.252534	0.640587	1.34292	Up	1.85E-06	3.88E-06
Akr1c12l1	1292	11	29.5	0.302437	0.766843	1.342297	Up	0.004325	0.006968
Fgfr1op2	2860	1805.5	4904.5	22.58883	57.27401	1.342271	Up	3.55E-12	1.01E-11
Pmp2	1350	20	54.5	0.530972	1.345505	1.341441	Up	6.50E-05	0.000123
RGD1307830	3638	734	1986	7.198475	18.23367	1.340842	Up	9.66E-13	3.06E-12
RGD1563669	1948	272	737.5	4.990245	12.63933	1.340738	Up	7.17E-13	2.31E-12
Phlda1	1811	21199.5	57642	419.8473	1062.665	1.33975	Up	0	0
Sbds	1465	1771.5	4794	43.19271	109.205	1.338179	Up	4.16E-12	1.17E-11
MGC105560	2838	1207.5	3261	15.1765	38.36148	1.337819	Up	7.00E-12	1.87E-11
Klf3	4988	1258.5	3408	9.040781	22.82581	1.336147	Up	2.27E-12	6.73E-12
Hist1h1d	660	107.5	293.5	5.852223	14.76997	1.33561	Up	0	0
LOC100359563	395	156.5	421	14.06199	35.48189	1.335282	Up	1.64E-13	6.07E-13
Fhl3	1067	1250.5	3390.5	42.03039	106.0195	1.334825	Up	4.14E-12	1.16E-11
Mybpc2	3658	14	38	0.137891	0.347813	1.334781	Up	0.000802	0.001377
LOC100909515	468	20.5	55.5	1.563139	3.941064	1.334139	Up	4.37E-05	8.43E-05
Ints12	1566	571	1545.5	13.06131	32.92705	1.333973	Up	0	0
Wdr25l	1895	91	246.5	1.719864	4.335567	1.333926	Up	0	0
Ppig	3964	3381	9125	30.48864	76.83788	1.333546	Up	0	0
Prkcb	2666	19.5	53	0.261623	0.65934	1.333535	Up	5.06E-05	9.67E-05
Snx16	2166	290	779	4.771331	12.02374	1.333422	Up	2.80E-13	9.94E-13

Pdss2	2100	261	705	4.44243	11.19343	1.333231	Up	2.23E-13	8.05E-13
E2f5	1713	489	1315.5	10.19575	25.67858	1.332598	Up	5.92E-13	1.95E-12
Foxn1	2415	168	456	2.50494	6.307821	1.332366	Up	1.69E-13	6.24E-13
Cdc14a	4476	424	1143.5	3.390423	8.530958	1.331243	Up	0	0
LOC690437	1569	31.5	84.5	0.714929	1.798366	1.330814	Up	5.44E-07	1.17E-06
Mcl1	2130	3475.5	9371.5	58.31621	146.6615	1.330522	Up	0	0
LOC100910237	546	27	73	1.76635	4.440727	1.330025	Up	3.18E-06	6.58E-06
Btbd16	1916	12	32	0.221119	0.55573	1.329559	Up	0.002459	0.004051
Gjb4	2659	43	115	0.574571	1.442942	1.328457	Up	6.16E-09	1.44E-08
Tmprss2	3180	20	53.5	0.223609	0.561555	1.328448	Up	9.61E-05	0.000179
Ubxn2b	1494	229.5	615	5.48857	13.78092	1.32817	Up	2.54E-13	9.11E-13
Rnf151	1170	42	113	1.283546	3.220692	1.327236	Up	7.14E-09	1.67E-08
LOC100911506	610	44	119	2.592366	6.499886	1.326145	Up	2.46E-09	5.84E-09
Dd25	3934	1296.5	3470.5	11.748	29.45493	1.326094	Up	4.61E-12	1.28E-11
Arhgap9	2480	20	53.5	0.286262	0.717518	1.325677	Up	9.61E-05	0.000179
Nsun6	2063	354	948.5	6.128263	15.35693	1.325339	Up	0	0
Grpel2	2028	484.5	1296	8.52265	21.34882	1.324782	Up	1.83E-12	5.50E-12
LOC100360499	207	45.5	121.5	7.800296	19.53791	1.324675	Up	2.13E-09	5.05E-09
LOC690155	1302	382	1022	10.46338	26.19994	1.324215	Up	5.33E-13	1.78E-12
Timmdc1	1360	278	745.5	7.311562	18.30766	1.324196	Up	1.88E-13	6.87E-13
Zmat3	7513	1992.5	5335	9.468096	23.70446	1.324013	Up	2.46E-13	8.83E-13
Nufip1	1671	656.5	1760	14.05452	35.17797	1.323638	Up	1.14E-12	3.55E-12
LOC100912875	695	22	58.5	1.131056	2.83033	1.323299	Up	4.77E-05	9.12E-05
Snapc5	1213	70.5	190	2.082335	5.2103	1.323164	Up	0	0
Trim30	1402	37	99	0.942306	2.356915	1.322633	Up	6.99E-08	1.57E-07

RGD1566099	727	209.5	561	10.31128	25.78941	1.322555	Up	1.08E-13	4.16E-13
LOC100911660	3220	817	2183.5	9.049244	22.63238	1.322519	Up	1.28E-12	3.95E-12
LOC100911072	1438	373	996	9.245056	23.11027	1.32178	Up	3.81E-13	1.31E-12
Vwa5b2	4509	78	208.5	0.617097	1.542537	1.321736	Up	0	0
Plk1s1	2584	415	1114	5.756019	14.38483	1.321405	Up	0	0
Klf4	2393	6474.5	17368	96.94846	242.2784	1.321376	Up	1.54E-11	4.00E-11
Ass1	1495	396.5	1060	9.48976	23.70416	1.320697	Up	0	0
LOC100912736	1469	25	67	0.608582	1.519684	1.320247	Up	9.38E-06	1.88E-05
Mlh3	5424	227.5	607.5	1.496371	3.73417	1.31932	Up	3.55E-15	1.49E-14
Alcam	2866	1699.5	4534.5	21.17526	52.78766	1.317821	Up	8.08E-12	2.14E-11
Cenpn	1865	1032.5	2753	19.76647	49.25849	1.317317	Up	1.69E-12	5.13E-12
RGD1563690	778	9.5	25.5	0.432888	1.07868	1.317202	Up	0.005991	0.009571
Ndrg3	2580	795.5	2125.5	11.02794	27.47756	1.317091	Up	0	0
Esf1	3404	1693.5	4505.5	17.75552	44.21623	1.316309	Up	5.62E-12	1.52E-11
LOC100912730	4580	124.5	331	0.966638	2.405619	1.315361	Up	1.27E-13	4.83E-13
Cep290	8251	737.5	1964	3.190389	7.939704	1.315353	Up	0	0
LOC100360772	4131	438	1160.5	3.774854	9.393887	1.315301	Up	7.26E-13	2.34E-12
LOC100912885	568	10.5	27.5	0.656943	1.633077	1.313753	Up	0.005099	0.008183
Hmgb2	1225	2141	5671	62.21218	154.6479	1.313718	Up	0	0
Ttc33	1790	241.5	640.5	4.810599	11.95682	1.313545	Up	2.12E-13	7.71E-13
RGD1565166	1529	23	61.5	0.537145	1.33495	1.313403	Up	2.77E-05	5.40E-05
LOC100911535	868	405.5	1081.5	16.72761	41.56934	1.313289	Up	0	0
Ei24	2115	2227.5	5925.5	37.62247	93.46577	1.312843	Up	0	0
RGD1560263	864	34	91	1.410791	3.503935	1.312471	Up	2.36E-07	5.19E-07
Lims2	2114	21.5	57.5	0.363519	0.902787	1.312354	Up	3.76E-05	7.28E-05

Mrpl50	1795	373	987.5	7.399317	18.37567	1.312333	Up	3.81E-13	1.31E-12
Lrrc48	1856	45	120.5	0.869752	2.159623	1.312104	Up	3.12E-09	7.38E-09
Pbxip1	3000	24.5	65.5	0.293663	0.729095	1.311944	Up	1.09E-05	2.18E-05
LOC683961	525	1331.5	3502.5	89.78481	222.8689	1.311652	Up	5.14E-12	1.41E-11
Rnf43	2349	74.5	198	1.134765	2.815363	1.310927	Up	3.11E-14	1.27E-13
Cox16	770	485	1279	22.36378	55.48251	1.310869	Up	6.91E-13	2.24E-12
LOC367975	1490	18	47	0.426508	1.057369	1.309835	Up	0.000287	0.000515
Nr1d2	1996	617.5	1640.5	11.04508	27.38112	1.309778	Up	8.96E-13	2.86E-12
LOC307738	936	22	58	0.833095	2.064033	1.308914	Up	4.77E-05	9.12E-05
LOC682408	1623	172.5	458.5	3.798086	9.409367	1.308825	Up	1.17E-13	4.46E-13
LOC683456	536	91	238.5	5.99918	14.8584	1.308442	Up	0	0
Tpk1	3331	119	314	1.27302	3.152104	1.308061	Up	0	0
Lrrtm3	3837	22	58.5	0.205467	0.508673	1.30783	Up	4.77E-05	9.12E-05
Lrrn4cl	2372	23	60.5	0.346245	0.857063	1.307606	Up	4.08E-05	7.87E-05
RGD1561852	1130	50	132	1.578254	3.904035	1.306636	Up	6.90E-10	1.67E-09
Col24a1	357	10.5	27.5	1.051645	2.600806	1.306311	Up	0.005099	0.008184
LOC100363455	1590	28	74	0.631588	1.561776	1.306133	Up	4.03E-06	8.28E-06
LOC100912253	4447	455	1201.5	3.651274	9.027301	1.305895	Up	3.90E-13	1.33E-12
Stat6	2635	62	164.5	0.842394	2.081397	1.304985	Up	5.41E-12	1.48E-11
Dtwd1	1587	164	433	3.683373	9.099111	1.304698	Up	1.50E-13	5.56E-13
Tox	3093	148	393	1.712413	4.228489	1.304112	Up	0	0
LOC100359664	1335	251.5	663.5	6.713051	16.57618	1.304071	Up	1.37E-13	5.15E-13
Zfand6	1571	1300.5	3422.5	29.44876	72.70128	1.303774	Up	5.74E-12	1.55E-11
Banp	2090	311	823.5	5.324956	13.14578	1.303759	Up	0	0
Cpeb3	5411	123.5	326.5	0.816023	2.014146	1.303486	Up	0	0

Lca5	3152	260	690	2.958576	7.298382	1.302674	Up		4.56E-13	1.54E-12
LOC100910252	522	18	47	1.225116	3.02161	1.302399	Up		0.000287	0.000515
LOC100912393	1533	31.5	82.5	0.72723	1.79293	1.301836	Up		1.18E-06	2.50E-06
Mterfd3	1643	26	68	0.562769	1.387282	1.301645	Up		1.18E-05	2.36E-05
Fip1l1	1925	4402	11634	81.91024	201.8713	1.30132	Up		1.98E-11	5.10E-11
Taf7	1796	441.5	1164.5	8.766846	21.60217	1.301046	Up		3.59E-13	1.25E-12
LOC100361436	1316	180.5	475.5	4.893361	12.0569	1.300961	Up		3.16E-13	1.11E-12
RGD1562407	2385	4071	10756	61.16489	150.6153	1.300093	Up		2.64E-11	6.73E-11
Rasa2	2556	297	780.5	4.138841	10.18813	1.29959	Up		3.95E-13	1.35E-12
Arhgef5	5585	12	32	0.07709	0.189683	1.298979	Up		0.002459	0.004051
Rnf113a1	1149	168	443	5.235507	12.87707	1.298404	Up		1.69E-13	6.23E-13
LOC100911069	1932	428	1123	7.89033	19.40558	1.298314	Up		2.52E-13	9.04E-13
Tti2	1946	296	780	5.439635	13.3757	1.298033	Up		3.73E-13	1.29E-12
Rbm18	2472	1620	4255	23.38731	57.45125	1.296612	Up		3.30E-12	9.47E-12
Coq5	1606	597.5	1572	13.30877	32.68392	1.296203	Up		2.85E-13	1.01E-12
Sned1	5597	18.5	49	0.118839	0.291809	1.296011	Up		0.000131	0.00024
Fam111a	3191	24633	64730.5	275.7555	677.0188	1.295806	Up		6.25E-11	1.56E-10
Tlk2	3689	1427	3750.5	13.82415	33.94012	1.2958	Up		0	0
Rbbp6	6191	2587	6806	14.94671	36.69611	1.295799	Up		0	0
RGD1565048	573	15.5	40.5	0.958451	2.352733	1.295561	Up		0.000686	0.001181
Kat3	1660	26	68.5	0.56046	1.374185	1.293892	Up		1.18E-05	2.36E-05
Gtf2h3	1878	505	1328.5	9.635352	23.60662	1.292782	Up		0	0
Ints2	1961	1294.5	3385.5	23.54631	57.68241	1.292631	Up		2.60E-12	7.60E-12
LOC679663	339	91.5	238.5	9.579649	23.46107	1.292224	Up		0	0
RGD1305254	7036	36	94.5	0.183575	0.449247	1.291137	Up		2.56E-07	5.61E-07

Plcd4	2696	187.5	491	2.490229	6.089475	1.29004	Up	3.37E-13	1.18E-12
Yes1	1795	829	2167	16.47783	40.26436	1.288977	Up	0	0
LOC497940	1480	203.5	532.5	4.920388	12.02313	1.288968	Up	1.92E-13	7.01E-13
Fam175a	1513	327.5	855.5	7.732848	18.89079	1.288611	Up	6.83E-13	2.22E-12
Usp21	2266	927.5	2432	14.67445	35.84811	1.28859	Up	2.08E-12	6.19E-12
Trpm8	4184	33	86.5	0.282366	0.689486	1.287954	Up	8.68E-07	1.85E-06
LOC100911474	2555	26.5	69.5	0.371923	0.907997	1.287685	Up	8.05E-06	1.63E-05
LOC100910753	2523	2257	5897.5	31.96606	78.01386	1.28719	Up	0	0
Nfatc2ip	1498	186.5	488.5	4.460539	10.88478	1.287023	Up	6.10E-13	2.00E-12
LOC679462	1646	66	173	1.437595	3.507423	1.286754	Up	1.86E-12	5.58E-12
Whamm	2849	351.5	920	4.415116	10.7677	1.286186	Up	9.48E-14	3.68E-13
Rest	3251	1379	3596	15.15338	36.95173	1.286002	Up	6.06E-12	1.63E-11
LOC100911511	1786	61.5	160.5	1.23298	3.006626	1.285996	Up	1.38E-11	3.59E-11
Cpsf7	3158	2099	5483.5	23.76865	57.95565	1.285889	Up	0	0
Nudcd2	1569	1938.5	5046.5	44.01119	107.3121	1.28587	Up	5.60E-12	1.52E-11
Bicd1	4139	945	2463	8.153399	19.87036	1.285145	Up	2.03E-12	6.06E-12
Vegfa	2544	2899.5	7570	40.76208	99.33898	1.285132	Up	0	0
Gad1	3216	80	209.5	0.891913	2.172819	1.284593	Up	0	0
Rnf135	2299	451.5	1171	6.997154	17.04126	1.284192	Up	2.05E-12	6.11E-12
N4bp2l2	2014	1254	3262	22.22119	54.1129	1.284037	Up	5.62E-12	1.52E-11
Map2k4	1194	551	1433.5	16.44868	40.04417	1.283621	Up	7.88E-14	3.09E-13
Muc15	1513	28	72	0.653118	1.589392	1.283059	Up	8.64E-06	1.74E-05
Usp8	4065	1412.5	3683	12.43538	30.23718	1.281872	Up	4.26E-12	1.19E-11
LOC100362724	714	151	393	7.557965	18.35661	1.28023	Up	0	0
LOC100360936	1234	427.5	1108	12.35449	30.00404	1.280122	Up	1.02E-12	3.21E-12

Mrps14	1286	850.5	2198.5	23.48855	57.01287	1.27933	Up	2.20E-12	6.54E-12
Rbm34	2900	1016.5	2637	12.50398	30.3447	1.279057	Up	3.94E-13	1.35E-12
Acot8	1503	231	601.5	5.501526	13.34851	1.278775	Up	0	0
Slc31a2	1763	189.5	491	3.837296	9.30597	1.278066	Up	2.87E-13	1.02E-12
Zc3hc1	1846	941.5	2444.5	18.21807	44.16571	1.277556	Up	0	0
C2	2629	23	60	0.312398	0.757173	1.277239	Up	4.08E-05	7.87E-05
LOC690675	388	16.5	42.5	1.503236	3.641983	1.276653	Up	0.000584	0.001013
Chmp1b	2531	1091	2822.5	15.3756	37.23257	1.275922	Up	4.14E-12	1.16E-11
Zfp174	1221	34.5	89	1.008021	2.440235	1.275494	Up	5.09E-07	1.10E-06
Gdpd2	2500	31	79.5	0.44073	1.066921	1.275485	Up	3.68E-06	7.59E-06
RGD1564300	1138	122.5	317	3.841052	9.294657	1.2749	Up	4.93E-14	1.96E-13
Sec22a	1661	286	740.5	6.144435	14.86025	1.274107	Up	3.49E-14	1.41E-13
LOC100912338	681	32	83	1.684812	4.074457	1.27402	Up	1.48E-06	3.13E-06
Naa30	4511	921.5	2386	7.287687	17.62076	1.273743	Up	0	0
LOC100362172	660	18	47	0.978513	2.365272	1.273344	Up	0.000287	0.000515
Bmp3	2158	16	41.5	0.265041	0.640594	1.273196	Up	0.000862	0.001472
Il10rb	1805	278	721	5.511213	13.31878	1.27302	Up	1.88E-13	6.87E-13
Dem1	2016	188.5	488	3.344523	8.080389	1.272624	Up	1.80E-13	6.62E-13
Pank2	1854	583	1507	11.22593	27.11377	1.27219	Up	5.08E-13	1.70E-12
Bdkrb2	4111	263	682	2.292151	5.533935	1.271604	Up	1.80E-13	6.61E-13
Has3	2465	23	59.5	0.333647	0.805341	1.271275	Up	5.97E-05	0.000114
Rbm5	3018	2462	6360	29.14232	70.3397	1.271224	Up	0	0
RGD1306072	1954	174	450.5	3.191125	7.700875	1.270957	Up	1.43E-13	5.33E-13
Gdf6	1576	15.5	40	0.351383	0.847952	1.270937	Up	0.000686	0.001181
Dpcd	1531	425.5	1097	9.914081	23.91896	1.270604	Up	1.53E-12	4.67E-12

LOC685515	542	13	33.5	0.850863	2.052782	1.270581	Up	0.003088	0.005043
Dynll1	801	9263.5	23988	414.1498	999.1694	1.270577	Up	1.56E-11	4.04E-11
Cpm	5450	768.5	1989	5.046974	12.17062	1.269912	Up	0	0
Fubp1	2380	4543	11765.5	68.41134	164.9518	1.269737	Up	1.79E-11	4.62E-11
Dpm1	2297	819	2113.5	12.72638	30.67909	1.269434	Up	4.71E-14	1.88E-13
Fxr1	2170	1949.5	5022	32.04057	77.22805	1.269225	Up	3.04E-12	8.80E-12
LOC100911177	487	1928	4965	141.2654	340.4863	1.269189	Up	4.36E-12	1.21E-11
Prrt3	3737	17	44	0.161707	0.38973	1.269087	Up	0.000497	0.000879
LOC100360704	825	25.5	65.5	1.101509	2.654529	1.268974	Up	2.03E-05	3.99E-05
Mto1	2405	438	1127	6.491589	15.63931	1.268533	Up	7.26E-13	2.34E-12
Dph5	1572	420	1086	9.572079	23.05864	1.268403	Up	1.49E-13	5.54E-13
Mmachc	1703	212	549.5	4.457886	10.73764	1.268245	Up	0	0
Pus10	1852	225.5	581	4.34908	10.47005	1.267486	Up	0	0
Usp53	6111	1170.5	3012.5	6.83876	16.46253	1.267379	Up	2.96E-12	8.57E-12
Dusp14l1	1305	140	362.5	3.84936	9.265493	1.267249	Up	1.60E-14	6.57E-14
LOC100365212	1456	45	116	1.103969	2.656848	1.267016	Up	1.42E-08	3.27E-08
Il28ra	1593	56	145	1.266196	3.04519	1.266032	Up	1.91E-10	4.70E-10
Tspan31	1480	2068.5	5317	49.88889	119.9194	1.265275	Up	0	0
Zbtb11	4883	685	1764	5.008844	12.03806	1.265053	Up	6.06E-13	1.99E-12
Trappc2	1474	343	880.5	8.290194	19.91963	1.264713	Up	0	0
Slfn3	4860	1877	4815	13.75639	33.05341	1.264698	Up	6.02E-12	1.62E-11
Tnk1	2055	15	39	0.26175	0.628801	1.264415	Up	0.001016	0.001721
Ogt	5308	2668	6850	17.93695	43.08296	1.264183	Up	0	0
LOC100911919	931	30	77	1.153058	2.769049	1.263926	Up	4.31E-06	8.85E-06
Ints6	4391	698.5	1795.5	5.688771	13.65827	1.263586	Up	0	0

Tnfrsf12a	993	6172.5	15914.5	222.7862	534.7777	1.26328	Up	5.65E-12	1.53E-11
LOC100912909	672	19	49.5	1.014288	2.434519	1.263169	Up	0.000244	0.00044
Grpel1	960	954.5	2440	35.36229	84.84971	1.262698	Up	1.35E-12	4.16E-12
Tmem199	1311	941.5	2415.5	25.63205	61.49382	1.262493	Up	0	0
LOC100911285	781	26	66.5	1.185373	2.843371	1.262261	Up	2.53E-05	4.95E-05
Med31	627	273.5	700	15.55346	37.30123	1.261987	Up	2.09E-13	7.60E-13
Tubb3	1796	64	165.5	1.276403	3.057776	1.260398	Up	1.26E-11	3.28E-11
Pik3cb	3213	420	1075.5	4.67487	11.19909	1.260383	Up	1.49E-13	5.54E-13
Col14a1	6437	19	49	0.105888	0.253449	1.259157	Up	0.000244	0.00044
LOC100912470	1437	208	530	5.151546	12.32701	1.258745	Up	3.49E-13	1.21E-12
Tmem140	1396	58.5	149	1.494788	3.575097	1.258041	Up	1.41E-10	3.47E-10
Zfp622	1670	657	1684.5	14.08893	33.69583	1.258008	Up	0	0
Milr1	1659	12	31	0.25883	0.618982	1.257893	Up	0.003658	0.005954
RGD1565247	4725	240	615	1.817561	4.345966	1.257673	Up	0	0
LOC287274	858	157.5	401	6.518141	15.58498	1.257624	Up	0	0
LOC100911519	3192	299	766.5	3.355109	8.020752	1.257378	Up	6.08E-13	2.00E-12
Rap2b	552	351.5	901.5	22.81964	54.54613	1.2572	Up	9.48E-14	3.68E-13
Stk19	1161	213.5	547	6.566216	15.68974	1.256687	Up	2.80E-13	9.94E-13
Trafd1	2466	1255	3207.5	18.18318	43.40671	1.255314	Up	5.54E-12	1.51E-11
LOC100360616	2714	112.5	286.5	1.474931	3.520623	1.255183	Up	0	0
Trpc1	4015	232.5	592	2.062489	4.919874	1.254235	Up	3.80E-13	1.31E-12
Oxnad1	2647	764.5	1958.5	10.35313	24.69608	1.254215	Up	9.61E-14	3.72E-13
Ndufaf2	602	181.5	460	10.70893	25.53394	1.253602	Up	1.41E-13	5.30E-13
Cdkn1b	1755	1620	4133.5	32.95455	78.54241	1.252994	Up	3.30E-12	9.47E-12
Cdc73	2101	868	2208.5	14.73424	35.10729	1.252599	Up	0	0

Trmt11	1568	242	615	5.508039	13.12021	1.25218	Up	3.84E-13	1.32E-12
Rfx7	7998	1605.5	4100	7.176913	17.0942	1.252072	Up	2.78E-12	8.11E-12
Terf1	2056	903	2300	15.67424	37.32761	1.251847	Up	0	0
Uqcrb	1054	2095	5341	71.03807	169.1361	1.25152	Up	0	0
Stx16	2581	738	1883.5	10.23126	24.35254	1.251089	Up	1.08E-12	3.38E-12
Gnpda1	2199	426.5	1086.5	6.930704	16.49419	1.250884	Up	5.03E-13	1.69E-12
Csad	2062	584.5	1493.5	10.15531	24.16166	1.250486	Up	5.51E-13	1.83E-12
Hbs1l	2721	2247	5717	29.49708	70.14623	1.249793	Up	0	0
Slc16a11	1779	44	112	0.886961	2.109007	1.249622	Up	3.49E-08	7.92E-08
Prickle3	2283	52	131	0.80725	1.91891	1.2492	Up	3.28E-09	7.73E-09
Prrg4	1878	2974.5	7561	56.5621	134.439	1.249044	Up	0	0
Tmem93	1267	763	1934	21.43632	50.94522	1.248889	Up	8.47E-13	2.71E-12
Celf5	4383	115	293	0.938871	2.23106	1.24873	Up	0	0
Zbtb40	4238	322	819	2.718783	6.456088	1.247699	Up	3.42E-13	1.19E-12
LOC500251	1486	235.5	598	5.655653	13.42773	1.24745	Up	3.29E-13	1.15E-12
Zgpat	1930	355.5	905.5	6.596063	15.65923	1.247336	Up	5.33E-13	1.78E-12
Mlf1	1112	176.5	448	5.669051	13.45809	1.247294	Up	0	0
Dcun1d5	1230	1591.5	4049.5	46.207	109.6929	1.247286	Up	3.50E-12	9.98E-12
Rnf181	1831	1236	3138	24.11224	57.18597	1.245895	Up	2.72E-12	7.94E-12
Zcchc4	2471	215	547	3.11301	7.382026	1.245706	Up	0	0
Pcnp	616	1595	4058.5	92.6977	219.8125	1.245668	Up	7.92E-12	2.10E-11
Rttn	7078	294	746	1.483903	3.518743	1.245663	Up	0	0
Urb1	7292	850	2157	4.166236	9.869768	1.244272	Up	2.20E-12	6.54E-12
Ocm	659	19	48	1.029946	2.438645	1.243511	Up	0.000358	0.000639
Thpo	1406	30	76	0.764735	1.809173	1.242299	Up	6.28E-06	1.28E-05

Taf1b	1962	258	653	4.696672	11.10458	1.241444	Up	2.94E-13	1.04E-12
Spice1	3056	231.5	587	2.708516	6.400313	1.24064	Up	0	0
RGD1561984	447	26.5	66.5	2.106625	4.978016	1.240638	Up	2.53E-05	4.95E-05
Ninl	4299	562	1426.5	4.681208	11.06098	1.240526	Up	3.62E-13	1.25E-12
Rimbp3	5203	15	38	0.103492	0.244532	1.240503	Up	0.001496	0.002511
Med4	1352	655.5	1655.5	17.32423	40.93382	1.240502	Up	7.02E-13	2.28E-12
Slc25a14	1571	129.5	328	2.946566	6.962046	1.240477	Up	0	0
Wdr12	1490	955.5	2414	22.90245	54.09776	1.240067	Up	1.30E-12	4.01E-12
Ets2	3614	1568.5	3968.5	15.52231	36.65067	1.239497	Up	1.45E-12	4.44E-12
Osgepl1	1835	286	723.5	5.557114	13.11978	1.239336	Up	3.49E-14	1.41E-13
Npas3	2796	44	111	0.559421	1.31997	1.238499	Up	5.04E-08	1.14E-07
Rnf182	1220	53	134.5	1.556398	3.671538	1.238174	Up	1.94E-09	4.61E-09
Zfp507	4167	832	2107	7.148065	16.86139	1.238099	Up	0	0
Cmc1	676	195	490	10.26271	24.2005	1.237626	Up	1.92E-13	7.02E-13
Helq	3728	324	817.5	3.104994	7.321397	1.237529	Up	0	0
LOC100912081	744	11.5	29	0.556569	1.312255	1.237415	Up	0.004325	0.006969
Ppp1r2	2262	4490.5	11302	70.74334	166.7794	1.237275	Up	2.62E-11	6.68E-11
MGC112715	1894	42	106	0.791687	1.865682	1.236701	Up	1.00E-07	2.23E-07
Lpin1	3107	348	880	4.01492	9.455615	1.2358	Up	4.24E-14	1.70E-13
Sh2d1b	399	82	206	7.316581	17.23053	1.235726	Up	9.99E-14	3.86E-13
Acot9	1698	1774.5	4472	37.33509	87.91026	1.235499	Up	4.61E-12	1.28E-11
Zfp307	1608	33.5	84	0.742809	1.748565	1.235107	Up	1.84E-06	3.86E-06
Dmtf1	3359	1407	3538	14.96189	35.1909	1.23391	Up	4.03E-12	1.13E-11
LOC681396	1702	460	1154	9.621617	22.6276	1.233732	Up	1.49E-13	5.55E-13
Cep19	1556	973	2449.5	22.34654	52.54836	1.233594	Up	9.37E-13	2.97E-12

LOC691918	611	17	43	0.995606	2.340806	1.233359	Up	0.00073	0.001254
LOC100362049	333	110.5	278	11.85249	27.84815	1.232391	Up	0	0
LOC682787	978	55	137.5	1.990655	4.676928	1.232318	Up	2.04E-09	4.84E-09
Ranbp10	5211	1537	3866.5	10.55162	24.77219	1.231257	Up	4.01E-12	1.13E-11
Ppapdc3	1982	106.5	268	1.923775	4.516279	1.231195	Up	0	0
Kcnj9	2319	22	55.5	0.340459	0.798843	1.230431	Up	0.000149	0.000273
LOC100360867	945	46	116	1.747897	4.097323	1.229061	Up	2.54E-08	5.80E-08
LOC690183	729	495	1242.5	24.32007	57.00846	1.229029	Up	4.63E-13	1.56E-12
Cenpc1	3148	1274	3188	14.43125	33.80818	1.228176	Up	3.25E-12	9.33E-12
RGD1562281	1502	171.5	429.5	4.08176	9.56051	1.227896	Up	3.66E-13	1.27E-12
Gfm2	3205	588.5	1473	6.560206	15.36293	1.22764	Up	1.12E-12	3.49E-12
LOC688784	482	98	243	7.180253	16.81327	1.227494	Up	0	0
Acot13	743	153	384.5	7.377042	17.27332	1.227431	Up	0	0
Vma21	1541	826.5	2072	19.14528	44.82412	1.227287	Up	0	0
Ccnc	3307	1973.5	4930	21.24969	49.73066	1.226694	Up	3.64E-12	1.03E-11
Nubpl	1157	136	339.5	4.180877	9.782344	1.226375	Up	1.95E-13	7.10E-13
Nfkbie	1669	498	1252.5	10.70285	25.03974	1.226225	Up	6.50E-13	2.13E-12
Mtf1	5300	552	1383.5	3.726425	8.714793	1.225674	Up	0	0
RGD1560849	2317	17	42.5	0.262297	0.613375	1.225569	Up	0.001066	0.001805
Ska3	1603	1176	2948	26.23822	61.35628	1.225541	Up	5.76E-12	1.56E-11
Phldb2	3861	1085.5	2718	10.05203	23.49818	1.225062	Up	3.86E-12	1.09E-11
Rps6kb1	2287	1618	4051	25.28798	59.09216	1.224515	Up	3.72E-12	1.05E-11
RGD1563300	471	14	35	1.061186	2.479062	1.224118	Up	0.002602	0.004274
Fem1b	2199	1082.5	2708.5	17.59947	41.1042	1.223754	Up	2.04E-12	6.10E-12
Utp3	1678	1513.5	3777.5	32.21462	75.23119	1.223615	Up	2.80E-13	9.96E-13

Hint3	900	247	617.5	9.779095	22.83156	1.223257	Up	0	0
Ubox5	3686	315.5	790	3.07038	7.165866	1.222724	Up	4.99E-13	1.67E-12
Dnal1	619	138	344.5	7.959921	18.56738	1.221944	Up	1.83E-13	6.73E-13
LOC100912278	1933	755.5	1884.5	13.95908	32.54609	1.22128	Up	1.01E-12	3.19E-12
Zfp423	4667	15	38	0.116361	0.271073	1.220076	Up	0.001496	0.00251
Tmbim4	894	703.5	1747.5	27.99632	65.21331	1.219929	Up	4.27E-13	1.45E-12
Dtx2	2665	670	1677.5	9.02155	21.01434	1.219927	Up	2.10E-13	7.62E-13
Rn28s	4786	233538	587627	1750.991	4076.151	1.219036	Up	8.21E-10	1.98E-09
Zfp512	3424	588.5	1463.5	6.136092	14.28413	1.219021	Up	1.12E-12	3.49E-12
Klhl12	1921	205	509	3.807572	8.861368	1.218658	Up	4.23E-13	1.44E-12
Car11	1346	32	80	0.853272	1.985026	1.21808	Up	4.55E-06	9.32E-06
Gtsf1	852	22	54.5	0.916577	2.131961	1.217854	Up	0.000217	0.000393
LOC681367	1017	106.5	265	3.738472	8.693417	1.217475	Up	0	0
Lias	1636	242	601.5	5.284006	12.27709	1.216265	Up	3.84E-13	1.32E-12
Car12	1065	204	509.5	6.861253	15.94153	1.216246	Up	1.72E-13	6.33E-13
LOC100912062	1312	26	65	0.708245	1.645154	1.215903	Up	3.67E-05	7.11E-05
Timm21	1415	686	1707	17.32765	40.24345	1.215678	Up	0	0
Cntd1	2501	168.5	416	2.390763	5.551545	1.215418	Up	1.69E-13	6.23E-13
LOC100362431	3455	70.5	175.5	0.731077	1.697087	1.214965	Up	1.03E-11	2.71E-11
Fam175b	2940	1089.5	2709.5	13.24732	30.7449	1.214647	Up	3.07E-12	8.87E-12
Isg15	663	41	102.5	2.216299	5.143285	1.214537	Up	2.45E-07	5.37E-07
Ube2b	2109	2868.5	7114	48.49954	112.55	1.214523	Up	0	0
Sh2b1	2791	1316.5	3275.5	16.90537	39.18207	1.212712	Up	3.15E-12	9.10E-12
Sgol1	1727	695	1725.5	14.40032	33.3513	1.211642	Up	7.07E-13	2.29E-12
Myocd	4952	12	30	0.086712	0.200809	1.211514	Up	0.005402	0.008656

Grin3b	3178	158.5	394.5	1.787094	4.136106	1.210658	Up	1.62E-14	6.66E-14
Mnd1	893	179.5	445.5	7.199453	16.66156	1.210564	Up	0	0
LOC100361188	1198	134.5	331	3.99227	9.238844	1.210503	Up	0	0
RGD1559704	1002	13	32.5	0.463681	1.072576	1.209874	Up	0.004534	0.007293
Zfp324	794	39.5	98.5	1.79062	4.141248	1.209606	Up	3.37E-07	7.35E-07
Zfp68	4654	1160.5	2866.5	8.885593	20.5421	1.209044	Up	3.48E-12	9.94E-12
LOC100910104	3986	20	49.5	0.179257	0.414274	1.208558	Up	0.00044	0.000782
Dpf2	2404	2084.5	5159	31.00426	71.64907	1.208482	Up	0	0
LOC100910127	2325	18.5	45.5	0.28411	0.655893	1.207009	Up	0.000617	0.001067
Abcd4	2358	248.5	615	3.76582	8.690962	1.206552	Up	7.11E-14	2.79E-13
LOC100359945	3117	219	541.5	2.510998	5.794196	1.206348	Up	3.38E-13	1.18E-12
Yipf4	1847	1234.5	3040.5	23.7748	54.83679	1.205711	Up	5.53E-12	1.50E-11
Rpp30	1444	732	1806	18.11718	41.78344	1.205572	Up	0	0
Cyp19a1	2863	300	744	3.758766	8.667966	1.205434	Up	2.14E-13	7.75E-13
Ier2	1564	2452.5	6077.5	56.24771	129.7054	1.205373	Up	0	0
Sdr42e1	2635	36.5	90	0.493602	1.137956	1.205025	Up	1.14E-06	2.42E-06
Patl1	4007	2002.5	4949	17.87288	41.20422	1.20502	Up	0	0
LOC100360884	355	28	69	2.812648	6.481662	1.204435	Up	2.64E-05	5.17E-05
Zc3h7a	3542	1489	3664.5	15.01194	34.55153	1.202639	Up	5.61E-12	1.52E-11
Trim11	2373	350	866.5	5.291295	12.1781	1.202596	Up	3.03E-13	1.07E-12
Zfp691	1483	62.5	153.5	1.504773	3.462121	1.202111	Up	3.23E-10	7.87E-10
Lcn2	876	91	224.5	3.717863	8.550672	1.201564	Up	0	0
Scn3a	6822	13.5	33.5	0.071189	0.163619	1.200605	Up	0.003088	0.005043
LOC100360290	476	54	131.5	4.013664	9.224403	1.200536	Up	1.01E-08	2.34E-08
Rhpn2	3185	90.5	223	1.01613	2.335058	1.200373	Up	0	0

LOC100911625	2213	1990.5	4901.5	32.21362	74.01709	1.200188	Up	0	0
Topors	3887	1950.5	4800	17.93976	41.21986	1.200179	Up	4.74E-12	1.31E-11
Nxt2	2447	268	657	3.897487	8.954162	1.200014	Up	1.39E-13	5.23E-13
Cnppd1	2235	1550.5	3811	24.80038	56.92736	1.19876	Up	3.74E-12	1.06E-11
Sgms1	2283	821.5	2018.5	12.87613	29.55166	1.19854	Up	0	0
RGD1311517	1779	201.5	496	4.05447	9.304474	1.198411	Up	0	0
Heatr8	4238	17	41.5	0.142456	0.326829	1.198024	Up	0.001547	0.002593
Dock2	6183	30.5	75	0.176468	0.404401	1.196379	Up	9.10E-06	1.84E-05
Sdad1	2274	780	1912	12.26258	28.09925	1.196268	Up	1.66E-12	5.03E-12
Zfp167	4837	101.5	250.5	0.751412	1.721138	1.195687	Up	0	0
Arv1	1142	318.5	782.5	9.985599	22.86053	1.194938	Up	1.88E-13	6.87E-13
Lsm3	709	1398	3424.5	70.46001	161.287	1.194754	Up	7.63E-12	2.03E-11
Setd5	7114	4503	11051.5	22.64683	51.83881	1.194724	Up	1.22E-11	3.19E-11
LOC688452	1112	72	176	2.315869	5.300431	1.194556	Up	2.21E-11	5.67E-11
Pogz	6063	1776.5	4356.5	10.48166	23.98771	1.194429	Up	3.75E-12	1.06E-11
Rsl24d1	1543	1585.5	3876	36.64898	83.85566	1.194135	Up	4.62E-12	1.28E-11
Cyp1b1	2321	584	1428.5	8.99794	20.58243	1.193746	Up	5.51E-13	1.83E-12
Sar1b	1190	1508.5	3681.5	45.20221	103.3904	1.193637	Up	2.75E-12	8.01E-12
Polk	3821	433	1060.5	4.050944	9.26411	1.193394	Up	1.10E-12	3.43E-12
Rab11fip2	1564	127.5	311.5	2.908495	6.65105	1.193309	Up	9.17E-14	3.56E-13
Crebl2	3505	338.5	827.5	3.446896	7.880153	1.192926	Up	1.46E-13	5.43E-13
Serpine1	3053	44546	109094.5	522.9182	1193.478	1.190514	Up	2.00E-10	4.91E-10
Lin7c	594	868	2121	52.29413	119.3403	1.19036	Up	0	0
Hist1h2ac	606	36	89	2.138984	4.881066	1.19027	Up	1.64E-06	3.46E-06
Wibg	1164	959.5	2351.5	29.55692	67.44187	1.190148	Up	0	0

Parp11	1375	189	461.5	4.90564	11.19045	1.189754	Up	2.87E-13	1.02E-12
Kcnk3	2077	42	102	0.719172	1.640469	1.189699	Up	4.29E-07	9.31E-07
Eif4ebp3	634	135.5	330.5	7.648131	17.43627	1.188912	Up	2.15E-13	7.77E-13
Gng7	2897	14.5	35	0.177617	0.404915	1.18885	Up	0.002602	0.004275
Gcfc1	3793	563.5	1370.5	5.287874	12.05264	1.18859	Up	1.35E-13	5.08E-13
Pum1	5009	3600	8812	25.74351	58.67284	1.188484	Up	0	0
Atp5s	2015	72	175.5	1.277469	2.910787	1.188122	Up	3.16E-11	8.00E-11
Kansl2	2082	1914.5	4671.5	32.88059	74.8883	1.187504	Up	4.86E-12	1.34E-11
LOC100366237	821	1496.5	3643	65.01357	148.0674	1.187441	Up	1.49E-12	4.56E-12
Epcam	1469	300.5	732	7.316143	16.64455	1.185895	Up	2.14E-13	7.76E-13
Pex3	1952	522.5	1273	9.560768	21.74514	1.185494	Up	0	0
RGD1560925	3966	14	34	0.125447	0.285313	1.185462	Up	0.003802	0.006181
Rbm44	3057	374	909	4.363542	9.923753	1.185386	Up	4.81E-13	1.62E-12
Rpl35a	348	290	703.5	29.70401	67.55136	1.185327	Up	2.80E-13	9.94E-13
Anapc10	1113	411	1000	13.20345	30.02544	1.18527	Up	0	0
Ercc5	3750	316	769.5	3.011052	6.845574	1.184904	Up	3.85E-13	1.32E-12
Nif3l1	1534	325.5	792	7.585194	17.23165	1.183803	Up	9.53E-14	3.69E-13
Flrt2	2900	517.5	1261	6.386544	14.50664	1.183607	Up	5.77E-14	2.29E-13
Rbbp5	3852	1361.5	3313.5	12.64061	28.69991	1.18298	Up	2.75E-12	8.01E-12
Pabpc1l	2182	22	54	0.362887	0.82378	1.18274	Up	0.000217	0.000393
Btrc	2334	963	2343.5	14.77214	33.531	1.182616	Up	1.71E-12	5.17E-12
Dusp11	1155	495	1202	15.30241	34.73372	1.182578	Up	4.63E-13	1.56E-12
Surf6	2049	1089.5	2653	19.03502	43.20347	1.182491	Up	3.07E-12	8.87E-12
Smek1	4388	3051	7420.5	24.85793	56.40788	1.182191	Up	0	0
St6galnac2	2035	13.5	33	0.239214	0.542735	1.181944	Up	0.003088	0.005042

Taf9	1703	4350.5	10541.5	91.07159	206.5882	1.181685	Up	2.71E-11	6.89E-11
Hoxa6	876	14.5	35.5	0.595903	1.351463	1.181374	Up	0.002602	0.004274
Psmd9	1448	2067.5	5039	51.13064	115.9316	1.181014	Up	0	0
Impact	3386	2042.5	4948.5	21.51968	48.78588	1.180807	Up	0	0
Rgs7bp	1364	75	182	1.967133	4.457478	1.180133	Up	1.36E-11	3.53E-11
LOC688292	3396	100	244	1.060104	2.401997	1.180028	Up	2.09E-14	8.54E-14
Endou	1236	12.5	30.5	0.363509	0.823501	1.179779	Up	0.005402	0.008655
LOC100125367	1993	254.5	616.5	4.557181	10.32379	1.17976	Up	3.19E-13	1.12E-12
Eif5	3504	8237	19916.5	83.74844	189.701	1.179593	Up	5.27E-12	1.44E-11
Aurkc	621	41.5	100	2.369611	5.364704	1.178848	Up	5.06E-07	1.09E-06
Cebpb	1841	784.5	1909.5	15.25241	34.5286	1.178755	Up	1.17E-13	4.46E-13
Ccdc150	3309	64	155.5	0.691744	1.56582	1.178609	Up	4.78E-10	1.16E-09
Frmd5	2578	181	440.5	2.521441	5.706339	1.178317	Up	1.41E-13	5.29E-13
Nmd3	1848	1728.5	4186	33.42821	75.62862	1.177866	Up	5.07E-12	1.39E-11
Ireb2	3323	1606	3883	17.23185	38.95717	1.176811	Up	4.80E-12	1.32E-11
Tbccd1	2167	492	1193	8.132764	18.38296	1.176551	Up	0	0
LOC501110	2039	32	77.5	0.562143	1.270539	1.17643	Up	1.36E-05	2.70E-05
Rarb	3092	188	456	2.175509	4.915357	1.175943	Up	1.80E-13	6.63E-13
LOC100912350	2265	244	591.5	3.854288	8.707571	1.175806	Up	2.85E-13	1.01E-12
Prpf38a	1656	1217	2942	26.2619	59.32705	1.175719	Up	2.01E-12	6.01E-12
Qtrt1	1431	192	464.5	4.802708	10.84893	1.175633	Up	0	0
Scn3b	3910	23	55.5	0.209756	0.47379	1.175533	Up	0.000264	0.000474
LOC100912536	1208	494	1190.5	14.562	32.88638	1.175281	Up	0	0
Arhgap20	3884	131	316.5	1.206163	2.723063	1.174806	Up	8.62E-14	3.36E-13
Mbnl2	4595	3972	9603.5	30.90995	69.75525	1.174231	Up	1.04E-11	2.73E-11

Atad5	6120	881	2125.5	5.136625	11.59074	1.17408	Up	5.46E-13	1.82E-12
Fastkd2	2240	375	904	5.973601	13.47517	1.173631	Up	0	0
Zscan21	2218	413	997	6.657027	15.01643	1.173592	Up	4.70E-13	1.58E-12
Lrr1	1496	77	185.5	1.832968	4.13416	1.173413	Up	1.41E-11	3.66E-11
Acvr2a	2277	477.5	1150	7.487203	16.8806	1.172868	Up	0	0
Rrp15	1190	987.5	2384	29.69431	66.93955	1.172672	Up	1.20E-12	3.72E-12
Repin1	1716	336.5	818	7.051854	15.89523	1.172519	Up	2.61E-13	9.32E-13
Junb	1822	13518	32890	266.6417	601.0154	1.1725	Up	0	0
Glyctk	2002	139.5	338	2.498686	5.631789	1.172424	Up	1.77E-13	6.53E-13
Tub	1582	68	164.5	1.539903	3.470215	1.172185	Up	1.75E-10	4.31E-10
Gins4	1079	821.5	1982.5	27.23756	61.37786	1.172121	Up	0	0
Thoc2	7636	2754.5	6620	12.86065	28.95069	1.170634	Up	0	0
LOC100363376	606	562	1345.5	33.00437	74.16651	1.168111	Up	3.62E-13	1.25E-12
Dram2	1435	562	1351	13.96731	31.37601	1.167608	Up	3.62E-13	1.25E-12
Fam107b	3085	82	196.5	0.946665	2.126145	1.167314	Up	4.36E-12	1.21E-11
Dkk4	1491	205	492	4.901818	11.00611	1.166916	Up	4.23E-13	1.44E-12
Ube2d3	1531	8619.5	20693.5	200.9609	451.2151	1.1669	Up	3.42E-12	9.79E-12
Ttc17	4540	983	2368.5	7.759652	17.41489	1.166258	Up	3.73E-13	1.29E-12
Eps8l2	3140	39	94	0.445355	0.998905	1.165392	Up	1.44E-06	3.04E-06
Ccdc134	1583	363.5	873	8.21083	18.41174	1.165026	Up	0	0
Lacc1	1482	180	432	4.340725	9.732795	1.164918	Up	3.16E-13	1.11E-12
Parg	3974	1696	4073.5	15.2575	34.20632	1.164744	Up	6.20E-12	1.67E-11
Mak16	1741	1301	3123	26.70851	59.87623	1.164684	Up	4.03E-13	1.38E-12
Magoh	669	1597	3842.5	85.46434	191.5529	1.164348	Up	8.39E-12	2.22E-11
Ddx47	1747	1931	4635.5	39.49329	88.49886	1.164051	Up	4.86E-12	1.34E-11

LOC100361180	456	181.5	431	14.08108	31.55245	1.163994	Up	1.41E-13	5.30E-13
Ndufs4	1512	1269.5	3039.5	29.94388	67.0757	1.163529	Up	6.03E-12	1.62E-11
Tmem178b	10116	89	214	0.314762	0.70495	1.163259	Up	3.27E-13	1.14E-12
LOC100363472	866	45	108	1.856095	4.156438	1.163077	Up	2.62E-07	5.72E-07
U2surp	4113	3524	8442.5	30.60663	68.52765	1.162842	Up	4.43E-13	1.50E-12
Mki67ip	1490	1292	3093	30.9354	69.24463	1.162444	Up	5.07E-12	1.39E-11
Shq1	2322	218	522.5	3.353073	7.504806	1.162331	Up	0	0
Jmjd4	1281	83.5	199.5	2.327774	5.209773	1.162269	Up	2.77E-12	8.08E-12
Dph3	3242	1257.5	3010.5	13.84388	30.9832	1.162238	Up	5.54E-12	1.50E-11
Znrf2	5774	452.5	1083	2.799265	6.26204	1.161585	Up	6.60E-13	2.15E-12
Tnrc6a	7938	3133	7509.5	14.11707	31.57464	1.161326	Up	0	0
Zfc3h1	7029	1741	4162.5	8.843911	19.77817	1.161152	Up	1.89E-12	5.68E-12
Calcrl	2095	68	163	1.161461	2.597208	1.161021	Up	2.49E-10	6.09E-10
Oraov1	2664	841.5	2012	11.27441	25.20847	1.160856	Up	9.66E-13	3.06E-12
LOC100912478	2628	250	598.5	3.39509	7.589291	1.160515	Up	2.66E-13	9.47E-13
Uqcc	1388	643	1537	16.54459	36.97473	1.16018	Up	0	0
RGD1308139	2880	969	2318	12.03499	26.88175	1.159392	Up	1.75E-12	5.29E-12
Supv3l1	2415	867	2076.5	12.84854	28.66856	1.157865	Up	0	0
Lingo1	2510	24	58	0.341694	0.762165	1.157401	Up	0.000155	0.000282
LOC687756	1788	22	52.5	0.43772	0.976039	1.15693	Up	0.00045	0.000799
Ercc8	2521	525.5	1253	7.444309	16.59836	1.156831	Up	4.15E-14	1.66E-13
Ndor1	2338	251	600.5	3.847947	8.578841	1.156694	Up	1.37E-13	5.15E-13
Gng5	1392	2421.5	5769	61.989	138.1943	1.156613	Up	0	0
Acer3	1840	1788.5	4247	34.58582	77.05793	1.155763	Up	1.02E-12	3.21E-12
Tdrkh	1970	585	1395.5	10.61114	23.63327	1.15524	Up	8.75E-13	2.79E-12

Zfp839	2751	28	66.5	0.364206	0.81115	1.155214	Up	7.80E-05	0.000146
LOC100911233	2419	1117	2666	16.52947	36.80258	1.154767	Up	4.68E-12	1.29E-11
RGD1305225	1793	410	975	8.169014	18.18625	1.154614	Up	1.10E-12	3.45E-12
Krr1	1691	786	1871.5	16.59795	36.94179	1.154249	Up	0	0
Cib2	1433	126	301	3.142326	6.990879	1.153641	Up	1.12E-13	4.31E-13
Plrg1	1830	1646	3918.5	32.10639	71.4231	1.15353	Up	5.04E-12	1.38E-11
Procr	1400	222.5	532.5	5.703567	12.68638	1.153344	Up	0	0
Casp8ap2	6781	1692.5	4021	8.904278	19.79587	1.152629	Up	8.42E-12	2.23E-11
LOC100359464	2771	110.5	264	1.425594	3.168501	1.152238	Up	0	0
Msl2	4957	1258	2992	9.07017	20.13799	1.150719	Up	2.27E-12	6.73E-12
Pot1	2730	618.5	1466.5	8.077212	17.92762	1.150255	Up	2.89E-15	1.22E-14
Tlr4	3373	661	1563.5	6.976836	15.48486	1.150213	Up	0	0
Cnksr3	3350	1822	4337.5	19.47533	43.21568	1.149907	Up	1.27E-12	3.92E-12
Ptp4a2	1095	3857	9151.5	125.7995	279.137	1.149848	Up	1.16E-11	3.04E-11
RGD1560891	3368	21.5	51	0.227149	0.50369	1.148895	Up	0.000371	0.000662
Ctdspl2	1621	892	2115	19.66241	43.58477	1.148384	Up	4.11E-13	1.40E-12
Nek11	2646	59	140	0.795504	1.763096	1.148171	Up	6.31E-09	1.47E-08
Hspa1a	4045	403	961	3.576537	7.925939	1.148019	Up	0	0
Zfp647	2350	34	81	0.518203	1.148099	1.147657	Up	9.69E-06	1.94E-05
Tmem80	2195	208	494.5	3.388759	7.505343	1.147161	Up	3.49E-13	1.21E-12
Lsm16	2298	645	1534	10.05333	22.26187	1.146901	Up	5.31E-13	1.77E-12
Pcgf1	828	113.5	268.5	4.9068	10.86185	1.146415	Up	2.09E-13	7.59E-13
Baiap2	1798	1673	3983	33.39535	73.91322	1.146185	Up	4.20E-12	1.18E-11
Rrn3	3445	2379	5640	24.67806	54.60688	1.145854	Up	0	0
Bcl6b	3338	32	76	0.343382	0.759616	1.145454	Up	1.94E-05	3.82E-05

Ptpn12	3167	4698.5	11098.5	52.90761	117.0078	1.145058	Up		7.09E-12	1.89E-11
C1H6orf35	988	455.5	1077.5	16.48185	36.43445	1.144425	Up		3.90E-13	1.34E-12
LOC685596	336	51.5	121.5	5.468419	12.08764	1.144337	Up		6.79E-08	1.53E-07
Ak8	1581	26.5	63	0.599601	1.325281	1.144224	Up		7.66E-05	0.000144
Mtfp1	1382	36	85	0.930465	2.055412	1.143404	Up		6.91E-06	1.40E-05
Ufsp2	1539	931	2202.5	21.65	47.80319	1.14274	Up		6.36E-13	2.08E-12
Txnl1	1301	3812.5	9005.5	104.6589	231.0673	1.142618	Up		4.19E-12	1.17E-11
LOC100363539	464	1341	3160.5	103.1138	227.5678	1.142059	Up		4.62E-12	1.28E-11
Ccdc111	1783	267.5	632.5	5.36479	11.8373	1.141747	Up		7.08E-13	2.29E-12
Rpgrip1l	7081	710	1677	3.58339	7.906395	1.141695	Up		3.82E-13	1.31E-12
Egfr	4161	268	633	2.303885	5.083142	1.141652	Up		1.39E-13	5.23E-13
Casp8	2003	375	886.5	6.693009	14.76598	1.14155	Up		0	0
Gpr3	969	15	35.5	0.555695	1.225472	1.140971	Up		0.004601	0.007398
Csrp2	842	568	1337.5	24.05981	53.05191	1.14078	Up		1.29E-13	4.89E-13
Crtap	839	579	1367.5	24.69974	54.45365	1.140533	Up		9.45E-13	3.00E-12
Tmem222	1490	780.5	1847	18.74359	41.32157	1.140498	Up		1.66E-12	5.04E-12
Gprc5a	2094	274.5	650	4.685716	10.32924	1.140393	Up		1.86E-13	6.82E-13
LOC100912698	992	42	99	1.515016	3.338301	1.139781	Up		1.24E-06	2.63E-06
Bdkrb1	1312	82.5	195	2.248122	4.953299	1.139669	Up		6.20E-12	1.67E-11
Zfp346	3298	287.5	680.5	3.123629	6.880071	1.1392	Up		4.35E-13	1.48E-12
Slc38a9	1807	131.5	309.5	2.597849	5.721178	1.138995	Up		8.62E-14	3.36E-13
Apool	1445	464	1091.5	11.45494	25.21948	1.138569	Up		0	0
RGD1311756	3102	1069.5	2522.5	12.32589	27.13471	1.138448	Up		2.20E-12	6.53E-12
RGD1564943	2954	728	1711.5	8.791051	19.34668	1.137978	Up		1.26E-12	3.91E-12
Mrps18b	1064	1507.5	3566.5	50.79149	111.7692	1.137864	Up		7.77E-12	2.06E-11

Spin4	750	40.5	95.5	1.925796	4.237454	1.137743	Up	1.74E-06	3.65E-06
LOC100910678	304	217	510	25.40682	55.87754	1.137053	Up	2.24E-13	8.07E-13
Ndufa7	523	1043.5	2457.5	71.2019	156.533	1.136479	Up	7.12E-13	2.30E-12
Vps37a	1743	747	1758.5	15.3054	33.63801	1.136051	Up	0	0
Pfdn2	868	1204	2825	49.49607	108.7595	1.135755	Up	9.08E-13	2.89E-12
LOC100911762	653	555	1302.5	30.32695	66.6119	1.13518	Up	0	0
Tgfbr3	3931	81	190.5	0.738934	1.622879	1.135038	Up	2.08E-11	5.33E-11
LOC100362027	480	6354.5	14922.5	472.4784	1037.298	1.13451	Up	3.12E-11	7.91E-11
Rab5a	649	453.5	1068	25.03464	54.92221	1.133464	Up	1.56E-12	4.77E-12
Commd6	909	436	1024	17.14317	37.60671	1.133356	Up	4.26E-13	1.45E-12
Npap60	3027	2715.5	6385	32.12466	70.45953	1.133114	Up	0	0
Sdcbp	2077	9797.5	22895.5	167.7646	367.8224	1.132571	Up	0	0
Tbx19	2542	14.5	34	0.204678	0.448683	1.132343	Up	0.003802	0.00618
Srp19	784	979	2291	44.55247	97.59373	1.131283	Up	0	0
Kctd21	881	21	49	0.848393	1.85795	1.130908	Up	0.000767	0.001316
LOC679110	693	29.5	69.5	1.520344	3.326887	1.129775	Up	4.61E-05	8.82E-05
Mapk1ip1	1445	238	560	5.895296	12.8971	1.129411	Up	6.71E-13	2.18E-12
LOC100911561	953	27.5	64	1.02926	2.251581	1.12933	Up	9.26E-05	0.000173
LOC100365010	348	405	947	41.56848	90.91383	1.12901	Up	0	0
Snrnp27	873	755.5	1763	30.85571	67.45882	1.12847	Up	1.01E-12	3.19E-12
RGD1562543	776	21	49.5	0.9691	2.118682	1.12845	Up	0.000767	0.001316
RGD1309085	1074	244	570	8.115642	17.74105	1.128314	Up	2.85E-13	1.01E-12
LOC100363310	1014	1103	2582	38.81596	84.84501	1.12818	Up	2.51E-12	7.39E-12
LOC100359498	471	3814	8896.5	288.5833	630.5869	1.127707	Up	5.62E-12	1.52E-11
Amacr	1542	562.5	1318.5	13.043	28.49952	1.127662	Up	3.62E-13	1.25E-12

Ces5a	2147	114	264.5	1.889845	4.128394	1.127313	Up	8.33E-14	3.25E-13
Nanp	1335	255.5	597.5	6.831435	14.92222	1.127202	Up	0	0
Dcp1b	1784	239.5	561	4.806591	10.49787	1.127011	Up	5.84E-13	1.93E-12
LOC100365259	2702	1739.5	4058.5	22.96732	50.15767	1.126888	Up	6.82E-12	1.82E-11
LOC100910723	1262	373	872.5	10.56937	23.08062	1.126793	Up	3.81E-13	1.31E-12
Atxn7l3b	3525	2510.5	5856	25.39912	55.45732	1.1266	Up	0	0
Ovca2	2935	330	772	4.020983	8.777212	1.126214	Up	2.96E-13	1.04E-12
LOC100360654	294	78	182	9.4467	20.61904	1.126095	Up	6.76E-11	1.69E-10
Atp7b	5506	62.5	145.5	0.406237	0.886602	1.125967	Up	5.42E-09	1.27E-08
Arhgef6	2319	864	2024.5	13.34947	29.13271	1.125857	Up	0	0
Usp32	6396	2051.5	4789.5	11.45564	24.99625	1.125654	Up	0	0
Nuf2	1813	1742.5	4072	34.36315	74.97738	1.125593	Up	3.16E-13	1.11E-12
Chordc1	2564	2976.5	6939	41.37783	90.24898	1.125052	Up	0	0
LOC690728	2760	2295	5355.5	29.71091	64.79791	1.124955	Up	0	0
Nrbf2	1759	283	662	5.760638	12.56155	1.124714	Up	0	0
Dguok	997	76	177.5	2.721957	5.935415	1.124705	Up	1.33E-10	3.29E-10
Ccdc51	1530	158	370	3.701259	8.070405	1.124625	Up	1.62E-14	6.66E-14
RGD1560854	1299	239	557	6.579701	14.34369	1.124322	Up	5.84E-13	1.93E-12
Zc3h15	2036	3580.5	8351.5	62.80219	136.9072	1.124312	Up	0	0
LOC100360053	537	16.5	39	1.106426	2.411332	1.123924	Up	0.001842	0.003067
Mplkip	1529	359	838.5	8.394187	18.27717	1.12258	Up	0	0
Tada2a	2124	812	1892.5	13.6766	29.77438	1.122362	Up	1.63E-12	4.97E-12
Prss46	750	65	151	3.087451	6.720678	1.122191	Up	3.28E-09	7.74E-09
Mll5	7209	4109	9596	20.42555	44.44976	1.121801	Up	2.80E-11	7.12E-11
LOC684112	6223	2574.5	6019.5	14.83129	32.27557	1.121799	Up	0	0

Tmem88	1567	79	183.5	1.795954	3.906528	1.121137	Up	8.05E-11	2.01E-10
RGD1304868	1594	148.5	345	3.326258	7.234599	1.121013	Up	0	0
Ccdc40	5157	14.5	34	0.10089	0.21942	1.120913	Up	0.003802	0.00618
LOC687118	2256	191	447.5	3.038417	6.608044	1.120904	Up	8.66E-14	3.37E-13
RGD1561636	213	75	174	12.52166	27.2188	1.120178	Up	2.21E-10	5.43E-10
Tbkbp1	3340	70.5	164	0.754189	1.639083	1.119891	Up	5.08E-10	1.23E-09
LOC367289	4758	1640.5	3817	12.33202	26.79896	1.119769	Up	2.90E-12	8.43E-12
RGD1563296	2115	657	1527.5	11.09585	24.11206	1.119735	Up	0	0
Pnpo	2076	162	378	2.788742	6.059592	1.119606	Up	0	0
Hbp1	2671	1104	2571	14.77734	32.09741	1.11907	Up	4.36E-12	1.21E-11
ST7	2098	826.5	1922.5	14.0878	30.58818	1.118528	Up	0	0
Fbxo33	3320	620	1436.5	6.662721	14.45627	1.117512	Up	8.34E-13	2.67E-12
Dbf4	2263	1303.5	3025	20.56534	44.60301	1.116926	Up	4.43E-12	1.23E-11
Ttc23	1948	126.5	294	2.323264	5.037486	1.116551	Up	1.12E-13	4.31E-13
Rps6kb2	1769	573	1335	11.62011	25.18259	1.115803	Up	7.09E-13	2.29E-12
Rabl3	1961	347.5	805	6.324172	13.70407	1.115656	Up	1.58E-13	5.86E-13
Cttnbp2nl	4791	3447	8000.5	25.73876	55.74923	1.115009	Up	0	0
Exoc3l1	2511	55	128.5	0.785838	1.701216	1.114263	Up	4.92E-08	1.11E-07
LOC100364116	398	567.5	1307	50.68039	109.7097	1.114192	Up	0	0
Tipin	1208	989.5	2284.5	29.1777	63.16119	1.114172	Up	2.57E-12	7.53E-12
Cyp2u1	2154	308	717	5.125688	11.09087	1.113555	Up	0	0
LOC100364165	6860	2748	6362.5	14.30281	30.94684	1.113493	Up	0	0
Bbs10	2667	143	331	1.913691	4.138895	1.112888	Up	0	0
Slc25a16	3204	595.5	1376	6.623608	14.32436	1.112782	Up	1.07E-12	3.35E-12
Cbr4	1187	139.5	322.5	4.199803	9.070595	1.110876	Up	1.77E-13	6.53E-13

Med10	860	852	1964	35.33768	76.30791	1.110625	Up	9.49E-13	3.01E-12
Tctex1d2	883	147	340	5.961015	12.87091	1.110485	Up	1.02E-13	3.94E-13
Serpinb10	1500	19	43.5	0.448285	0.967918	1.110469	Up	0.002228	0.00368
Dr1	3035	1414	3268.5	16.63921	35.91972	1.110189	Up	0	0
Pcca	2495	420	971	6.009381	12.97106	1.110008	Up	1.49E-13	5.55E-13
Cdc7	2917	611	1408.5	7.478313	16.13825	1.109699	Up	4.93E-13	1.66E-12
RGD1311564	2166	46	106	0.757557	1.634719	1.109617	Up	8.93E-07	1.90E-06
LOC687575	935	3033	7007.5	115.9927	250.0324	1.108082	Up	0	0
Hmha1	3891	54	124.5	0.49749	1.072323	1.107999	Up	1.16E-07	2.57E-07
Cited1	873	68.5	158.5	2.805435	6.046689	1.107921	Up	1.41E-09	3.37E-09
LOC100912905	1032	47.5	109.5	1.647277	3.549909	1.107699	Up	5.34E-07	1.15E-06
Cacna1c	8257	70.5	164	0.308546	0.664762	1.107353	Up	5.08E-10	1.23E-09
Ttc27	2821	1589.5	3659	20.11856	43.30941	1.106154	Up	3.80E-12	1.07E-11
LOC100361445	171	124.5	283.5	25.78611	55.4913	1.105668	Up	1.29E-13	4.87E-13
Exnef	2541	217	500.5	3.053386	6.570346	1.105559	Up	2.24E-13	8.07E-13
Snap47	1822	242	555.5	4.730107	10.17394	1.104934	Up	3.84E-13	1.32E-12
Gtf2h1	2632	2199.5	5065.5	29.87825	64.25651	1.104747	Up	0	0
Tomm5	628	861.5	1977	48.85286	105.0316	1.104308	Up	1.12E-12	3.49E-12
Fcho2	3051	695.5	1599	8.137999	17.4924	1.103982	Up	7.07E-13	2.29E-12
Srp9	1328	1191	2737.5	31.96522	68.70568	1.103926	Up	3.02E-12	8.74E-12
Rnf168	3311	691.5	1589.5	7.450875	16.01416	1.103866	Up	1.59E-12	4.83E-12
Golph3	2530	2487	5718.5	35.07284	75.38143	1.103855	Up	0	0
Crnkl1	2311	422	972.5	6.53221	14.03774	1.103668	Up	4.13E-13	1.41E-12
Ccdc53	1039	433	997.5	14.90648	32.02078	1.103069	Up	1.10E-12	3.43E-12
LOC683722	1483	608.5	1396.5	14.6351	31.43124	1.102767	Up	1.66E-12	5.04E-12

Armc8	4447	686.5	1574.5	5.510258	11.82808	1.102024	Up	0	0
Stard6	1297	130.5	300	3.591773	7.709285	1.101901	Up	0	0
Cdh6	3594	35.5	83.5	0.352893	0.757275	1.101586	Up	8.18E-06	1.65E-05
Fam134c	3106	833.5	1911.5	9.575255	20.54248	1.101227	Up	9.78E-13	3.09E-12
Slc25a42	2830	93	213.5	1.171863	2.51351	1.1009	Up	3.98E-12	1.12E-11
Gbe1	2937	1014.5	2328	12.34435	26.47709	1.100893	Up	2.17E-12	6.44E-12
LOC681224	1191	177.5	409.5	5.347619	11.4699	1.100884	Up	0	0
Synj1	5970	1433	3290.5	8.582355	18.39051	1.099516	Up	4.69E-12	1.30E-11
Ndufa4	890	3277.5	7509.5	131.5187	281.8086	1.099448	Up	0	0
Tmem87a	2880	2037	4653.5	25.19469	53.95871	1.098736	Up	0	0
Id2	725	1654.5	3807	81.76302	175.103	1.098683	Up	4.25E-12	1.19E-11
Tmem60	1370	438.5	1006.5	11.45053	24.5129	1.098127	Up	7.26E-13	2.34E-12
Stx3	1054	16.5	38	0.559358	1.196868	1.09742	Up	0.002667	0.004372
Rab28	1483	1866.5	4268.5	44.91701	96.10867	1.097405	Up	5.21E-12	1.42E-11
LOC362695	1020	282	641.5	9.821051	21.0096	1.097099	Up	6.96E-13	2.25E-12
Mtmr14	2656	446	1021	6.009213	12.8516	1.0967	Up	0	0
Vdr	2043	56	129	0.985333	2.105922	1.095768	Up	5.86E-08	1.32E-07
Akap3	2889	14.5	33.5	0.180292	0.385119	1.094967	Up	0.005515	0.00883
Vapa	1618	5927.5	13538.5	130.697	279.1601	1.094867	Up	1.85E-11	4.78E-11
LOC679140	1033	600.5	1370	20.74338	44.25957	1.093339	Up	0	0
Pspc1	2034	1089.5	2493	19.14974	40.85861	1.093316	Up	3.07E-12	8.87E-12
Glipr1	1072	80	183.5	2.670925	5.69443	1.092212	Up	1.34E-10	3.32E-10
LOC64038	853	66	150.5	2.759279	5.882726	1.092193	Up	7.68E-09	1.79E-08
RGD1563956	603	99.5	225.5	5.847947	12.46679	1.092088	Up	1.46E-12	4.47E-12
Insig2	720	259	588.5	12.79636	27.27846	1.092028	Up	1.39E-13	5.21E-13

Adnp2	4263	1292.5	2957.5	10.85915	23.14629	1.09187	Up	5.07E-12	1.39E-11
LOC685233	7054	1903	4351.5	9.659311	20.58743	1.091772	Up	4.21E-12	1.18E-11
Pkdcc	1855	47.5	108.5	0.916746	1.952571	1.090781	Up	7.53E-07	1.61E-06
LOC100912007	339	62	141.5	6.520744	13.88668	1.090593	Up	2.12E-08	4.86E-08
Jmjd6	1730	1202	2745	24.86708	52.9563	1.090565	Up	5.72E-12	1.54E-11
Tmem41a	1444	261	595	6.450272	13.73397	1.090317	Up	2.23E-13	8.05E-13
Cox7a2l2	449	1119.5	2542.5	88.89907	189.2811	1.09029	Up	5.35E-12	1.46E-11
Tmem171	1103	42	96	1.359953	2.894409	1.089712	Up	3.51E-06	7.25E-06
Zscan30	1673	142.5	323.5	3.040858	6.471718	1.08967	Up	2.91E-13	1.03E-12
Cox17	440	529.5	1204	42.92828	91.30323	1.088737	Up	0	0
LOC100909597	998	112.5	256.5	4.042013	8.596661	1.088703	Up	0	0
LOC500684	2686	74	168.5	0.98541	2.095617	1.088579	Up	1.01E-09	2.43E-09
Nsmaf	2787	742	1690.5	9.523496	20.24921	1.088303	Up	4.00E-13	1.37E-12
Slfn2	2078	1111	2530	19.11269	40.62952	1.087997	Up	5.50E-12	1.50E-11
Rup2	569	14.5	33	0.910363	1.934736	1.087622	Up	0.005515	0.008831
Coq4	1257	128.5	292	3.648679	7.752147	1.087222	Up	3.73E-14	1.50E-13
Itgax	3623	40	91	0.395384	0.839829	1.086841	Up	6.97E-06	1.42E-05
Rps29	318	3750	8479.5	418.7515	889.2959	1.086569	Up	5.56E-12	1.51E-11
Gphn	3220	450	1023	4.995776	10.60454	1.085901	Up	1.46E-12	4.47E-12
Csnk1g3	4344	1812	4109	14.87533	31.57031	1.085646	Up	3.37E-12	9.65E-12
Golga7	2012	3007	6835.5	53.40711	113.3384	1.085533	Up	0	0
Chmp5	1355	2709	6140.5	71.23155	151.1344	1.085244	Up	0	0
LOC100909715	810	107	243	4.728337	10.03049	1.084987	Up	1.22E-13	4.64E-13
LOC680017	3850	2453	5551	22.68375	48.1134	1.08478	Up	0	0
Arl6	1005	337	762.5	11.94589	25.33034	1.084352	Up	0	0

RGD1562420	877	867	1942.5	34.87893	73.94508	1.084098	Up	0	0
LOC684441	525	633.5	1439	43.12	91.41223	1.08403	Up	0	0
RGD1311745	808	435.5	989.5	19.25718	40.80012	1.083177	Up	1.31E-12	4.03E-12
Tbpl1	1270	602.5	1366	16.91156	35.8255	1.082977	Up	7.49E-13	2.41E-12
Arglu1	1742	1592.5	3606.5	32.59987	69.05814	1.082945	Up	4.26E-12	1.19E-11
Atxn3	3071	423	958	4.918145	10.41122	1.081953	Up	1.33E-13	5.02E-13
LOC689153	1854	28.5	65	0.553004	1.170519	1.081786	Up	0.000111	0.000207
Dync2li1	1689	432.5	980	9.144801	19.35428	1.081629	Up	9.58E-13	3.03E-12
Ccnk	2552	1645.5	3742	23.13205	48.94735	1.081338	Up	5.18E-12	1.42E-11
Ppfibp2	3389	1836.5	4160	19.39678	40.97092	1.078783	Up	8.68E-12	2.29E-11
Det1	1873	277.5	628.5	5.300502	11.19432	1.078566	Up	6.93E-14	2.73E-13
Cep152	5396	465	1050	3.075329	6.49369	1.078298	Up	0	0
Akap8l	2194	537	1215	8.756941	18.48618	1.077948	Up	6.73E-14	2.65E-13
LOC100911576	1707	7465	16841	156.12	329.5115	1.077674	Up	0	0
Chrm3	3578	82	185.5	0.818792	1.727782	1.077352	Up	1.87E-10	4.60E-10
RGD1560402	1218	49	111.5	1.431552	3.019768	1.076858	Up	7.52E-07	1.61E-06
Eml4	4837	593	1337.5	4.377776	9.233887	1.07674	Up	1.48E-12	4.54E-12
LOC100909856	2558	299.5	677.5	4.196918	8.849185	1.076215	Up	6.08E-13	2.00E-12
Pdgfc	1116	2015.5	4553	64.66807	136.3421	1.076106	Up	0	0
Mtmr10	4644	2818	6351.5	21.67107	45.68637	1.075993	Up	0	0
RGD1309492	3986	697	1575.5	6.253676	13.1805	1.075629	Up	0	0
RGD1311558	3581	94	209.5	0.927768	1.955372	1.075607	Up	2.43E-11	6.20E-11
Zfp426l2	2627	171.5	387.5	2.337913	4.926662	1.075389	Up	3.66E-13	1.27E-12
RGD1563702	1626	71	160	1.560727	3.288616	1.075262	Up	3.28E-09	7.75E-09
Evi2a	1410	302	682	7.65323	16.12263	1.074946	Up	5.71E-13	1.89E-12

Rfesd	771	196	442	9.095158	19.15989	1.074918	Up	1.28E-13	4.85E-13
Slc27a5	2359	26.5	59	0.397233	0.83655	1.074465	Up	0.000317	0.000568
LOC100363502	1197	3069.5	6899.5	91.42483	192.4493	1.07382	Up	0	0
Mrps31	1361	652	1471.5	17.14567	36.08328	1.073486	Up	1.68E-12	5.10E-12
Cyp4v3	3216	909.5	2045.5	10.10478	21.25111	1.072501	Up	2.43E-12	7.16E-12
Klf11	3825	297.5	669.5	2.779917	5.846019	1.072413	Up	3.95E-13	1.35E-12
Clec2d2	702	52.5	118	2.669153	5.612028	1.07214	Up	3.21E-07	6.99E-07
Gemin2	1350	270.5	606	7.120414	14.96897	1.071942	Up	4.53E-13	1.53E-12
LOC100910202	3255	677.5	1523	7.436606	15.62707	1.071331	Up	5.28E-13	1.76E-12
Shfm1	753	3042.5	6835	144.0405	302.6621	1.071234	Up	0	0
LOC689656	1205	135	303.5	4.005579	8.413898	1.070764	Up	2.15E-13	7.78E-13
E2f3	2020	491.5	1105.5	8.694314	18.25513	1.070158	Up	5.39E-13	1.79E-12
Glmn	354	557.5	1248.5	56.06925	117.7079	1.069929	Up	5.01E-13	1.68E-12
LOC100360754	436	23	52	1.886332	3.957142	1.068875	Up	0.000765	0.001312
Nip7	1175	700.5	1571	21.26726	44.59675	1.068305	Up	0	0
Snrpe	296	2573	5778.5	310.9881	652.042	1.068106	Up	0	0
LOC100362083	3570	104	234	1.042425	2.185606	1.06809	Up	7.64E-13	2.45E-12
Myh11	6555	164.5	369	0.896987	1.880127	1.067672	Up	1.50E-13	5.56E-13
RGD1563446	1002	40	90	1.427328	2.99163	1.067615	Up	9.80E-06	1.96E-05
Gpatch1	3073	398.5	895	4.637213	9.716465	1.067173	Up	5.90E-13	1.95E-12
Zfp386	1907	428	958	7.989259	16.73915	1.067093	Up	2.52E-13	9.05E-13
RGD1311463	1347	552	1238	14.64692	30.68714	1.067036	Up	0	0
Gpr182	1908	16	36	0.30067	0.62994	1.067033	Up	0.005486	0.008786
Dclre1c	2162	343.5	770.5	5.675318	11.89037	1.067021	Up	0	0
LOC100912076	1208	21	47	0.619686	1.298245	1.066953	Up	0.00155	0.002598

LOC100910638	1729	318.5	713	6.5749	13.76483	1.065946	Up	1.88E-13	6.87E-13
LOC691807	1163	2330.5	5201	71.40834	149.3301	1.06434	Up	0	0
LOC687029	671	428	959.5	22.80738	47.68867	1.064145	Up	2.52E-13	9.04E-13
Txn1	330	4185	9291.5	449.8971	940.3997	1.063679	Up	2.70E-11	6.86E-11
Npas2	4273	797.5	1792	6.692634	13.9882	1.063564	Up	4.88E-13	1.64E-12
Bcap29	1767	1853.5	4129.5	37.31734	77.99099	1.063462	Up	5.00E-12	1.37E-11
Nmnat3	1854	46	103.5	0.887826	1.855331	1.063329	Up	2.47E-06	5.15E-06
Pawr	2122	347.5	778.5	5.862181	12.24844	1.063088	Up	1.58E-13	5.86E-13
Hdgfrp3	2750	1080.5	2405.5	13.98981	29.22514	1.062834	Up	5.71E-12	1.54E-11
Gls	4219	1790	3974	15.07306	31.44802	1.060997	Up	4.89E-12	1.34E-11
Mocs3	1961	127.5	284.5	2.32728	4.854971	1.060818	Up	9.61E-14	3.72E-13
Bax	865	2014.5	4507.5	83.37707	173.9203	1.060704	Up	0	0
Rpe	2119	1232	2748.5	20.75399	43.28499	1.060478	Up	3.75E-12	1.06E-11
Rg9mtd3	1301	299.5	668	8.22016	17.13489	1.059698	Up	6.08E-13	2.00E-12
Lin52	2353	402.5	896.5	6.108222	12.73252	1.059694	Up	1.18E-14	4.87E-14
Coq10a	1287	130	291	3.618924	7.542705	1.059521	Up	0	0
Sec61g	443	2338	5188	187.4302	390.6354	1.059469	Up	0	0
Olig2	2650	31	69.5	0.420544	0.876465	1.059441	Up	0.000131	0.00024
LOC687970	1191	19	42.5	0.570368	1.187984	1.058551	Up	0.003156	0.005151
Proca1	1444	47	104.5	1.163498	2.420826	1.05703	Up	2.90E-06	6.02E-06
Larp6	2336	147.5	329.5	2.262989	4.708349	1.056992	Up	1.02E-13	3.94E-13
Glrx3	1245	4547	10140	130.6367	271.7986	1.056978	Up	1.32E-11	3.43E-11
LOC689229	3079	301	670	3.489008	7.258936	1.056941	Up	0	0
RGD1566320	646	244	538.5	13.36474	27.80379	1.056849	Up	2.85E-13	1.01E-12
LOC100910121	1904	862.5	1920	16.18112	33.64137	1.055925	Up	0	0

Rbm22	1725	1892	4223	39.31063	81.71652	1.055709	Up	5.43E-12	1.48E-11
St3gal3	1207	356.5	796.5	10.59532	22.0234	1.05561	Up	6.28E-13	2.06E-12
Hoxc8	1228	22.5	50.5	0.656338	1.36383	1.055154	Up	0.000914	0.001559
Wbp5	1043	3010.5	6676	102.8397	213.6546	1.054883	Up	0	0
RGD1308165	1625	306	682	6.740375	14.00222	1.054755	Up	0	0
Atp5j	573	2256	4996.5	140.2083	291.245	1.054662	Up	0	0
Atf2	1852	1260.5	2813	24.39472	50.67327	1.054656	Up	2.88E-12	8.37E-12
Tma7	528	475	1055.5	32.12728	66.72752	1.054483	Up	1.22E-12	3.79E-12
Top1	2304	2140	4761.5	33.20729	68.95011	1.054053	Up	0	0
Ctgf	2349	47404	105482.5	721.6349	1498.173	1.053863	Up	1.44E-10	3.55E-10
Zcchc6	5688	1493	3318	9.379868	19.46805	1.053469	Up	5.68E-12	1.54E-11
LOC100362458	5870	2146.5	4771	13.07817	27.12836	1.052641	Up	0	0
Ppp2r2d	1734	1413	3138.5	29.10807	60.37823	1.052609	Up	4.61E-12	1.28E-11
Rad52	1864	100	221.5	1.915394	3.971475	1.052033	Up	8.81E-12	2.33E-11
RGD1563842	1948	18	40	0.330646	0.685561	1.051998	Up	0.003794	0.006168
Slu7	2256	1551.5	3453	24.64131	51.07981	1.051674	Up	2.30E-12	6.80E-12
Sox9	2652	1032	2298	13.96275	28.93801	1.051382	Up	1.69E-12	5.13E-12
LOC100912749	574	126.5	277.5	7.8106	16.18239	1.050919	Up	1.41E-13	5.30E-13
LOC691069	7134	252	561	1.263598	2.617381	1.050587	Up	1.17E-13	4.46E-13
Stap2	1439	747	1658.5	18.58143	38.48546	1.050452	Up	0	0
RGD1563666	4050	65	143.5	0.572175	1.185076	1.050451	Up	4.78E-08	1.08E-07
Rad1	1321	302.5	667	8.154831	16.87949	1.049544	Up	5.71E-13	1.89E-12
Rhob	2183	8069.5	17913.5	132.2073	273.6255	1.049401	Up	0	0
LOC100909561	1303	34.5	77.5	0.945025	1.955738	1.049289	Up	3.91E-05	7.56E-05
Thumpd3	2002	1325.5	2939.5	23.68463	48.99854	1.048788	Up	2.55E-12	7.48E-12

Psmd10	696	292.5	646	14.9999	31.01549	1.048036	Up	0	0
Bcar3	3337	1987	4408	21.31898	44.05393	1.047133	Up	0	0
Prim2	2141	1100	2436	18.3868	37.98957	1.046933	Up	3.55E-12	1.01E-11
Cldn1	3275	24.5	54	0.265853	0.549126	1.046509	Up	0.000639	0.001104
Ppp1r12a	3300	3024	6687.5	32.76213	67.64129	1.045875	Up	0	0
RGD1306487	2816	2571	5673	32.592	67.2837	1.045739	Up	0	0
Rc3h2	3930	1346.5	2975	12.24556	25.27121	1.045236	Up	3.49E-12	9.95E-12
Cks1bp6	735	681	1504.5	33.05674	68.21892	1.045228	Up	1.16E-13	4.45E-13
Pard6g	1149	130	287.5	4.050578	8.348092	1.043319	Up	0	0
Srsf10	1218	1732.5	3821.5	50.81076	104.7108	1.043204	Up	7.47E-12	1.99E-11
LOC100910207	2608	1025.5	2259	14.02284	28.89693	1.043138	Up	2.27E-13	8.17E-13
Rnf4	1040	1323	2906	45.28496	93.27834	1.04251	Up	6.80E-12	1.82E-11
Mmd	2671	2970.5	6544.5	39.70556	81.76767	1.04219	Up	0	0
Odc1	2506	7928.5	17448.5	113.0015	232.5674	1.041307	Up	1.92E-11	4.96E-11
Cdca5	1508	389	861.5	9.259075	19.05404	1.041157	Up	0	0
LOC100365214	294	116.5	254.5	14.09847	29.00066	1.040548	Up	5.13E-13	1.72E-12
Wfdc6a	752	60	132	2.849707	5.861648	1.040493	Up	1.57E-07	3.48E-07
Kcnip4	1289	102	225	2.831122	5.820118	1.039674	Up	6.07E-12	1.63E-11
Coq3	1058	444.5	977.5	15.01499	30.8631	1.039479	Up	1.07E-12	3.36E-12
Elmo3	2469	65	143.5	0.941116	1.93445	1.039478	Up	4.78E-08	1.08E-07
RGD1307325	2069	430	946.5	7.421581	15.25489	1.039473	Up	7.47E-13	2.40E-12
Mysm1	2560	687.5	1509.5	9.583211	19.69756	1.039436	Up	0	0
Ager	1443	59	130	1.462673	3.005326	1.038914	Up	1.87E-07	4.13E-07
Hdac2	2195	3277.5	7181	53.17652	109.1947	1.038041	Up	0	0
Mpv17l	1542	456.5	1003	10.58802	21.74168	1.03803	Up	1.95E-12	5.83E-12

RGD1309148	872	186.5	409	7.642329	15.68338	1.037152	Up	6.10E-13	2.00E-12
Rnf19b	1331	915	2014	24.62816	50.54083	1.037141	Up	0	0
Mmaa	3295	62.5	137	0.678828	1.391986	1.036026	Up	8.03E-08	1.79E-07
Ttpal	4643	1743	3822	13.39661	27.47	1.035989	Up	2.81E-12	8.17E-12
Anxa1	1401	14852.5	32530	377.9843	774.8388	1.03557	Up	0	0
Nrip1	7910	363	793.5	1.635904	3.353273	1.035482	Up	0	0
Kcnj6	16380	41.5	91.5	0.091062	0.186584	1.0349	Up	1.15E-05	2.30E-05
Cnih4	1665	1305.5	2862	28.02417	57.39355	1.034217	Up	7.23E-12	1.93E-11
Gabarapl1	1719	890.5	1955	18.55343	37.98355	1.033688	Up	5.11E-15	2.14E-14
Rheb	1088	2171.5	4759.5	71.28697	145.9333	1.033598	Up	0	0
Heatr7b1	5064	128.5	281.5	0.907384	1.856722	1.032973	Up	5.75E-14	2.28E-13
Tbp	1923	656.5	1440	12.22109	25.00713	1.032967	Up	1.14E-12	3.56E-12
LOC100363434	363	3169	6906.5	310.3634	634.8029	1.03235	Up	0	0
Med17	4181	656.5	1438	5.61038	11.47219	1.031971	Up	1.14E-12	3.56E-12
LOC100909827	3011	28	61	0.330852	0.676086	1.03102	Up	0.000438	0.000778
LOC100361876	171	33.5	72.5	6.938067	14.17365	1.030606	Up	0.000128	0.000235
Cdc5l	2847	3144	6872	39.46096	80.61365	1.030598	Up	0	0
LOC100911867	1934	34	74.5	0.627888	1.28261	1.030502	Up	0.000107	0.0002
LOC690734	944	53	115.5	1.995652	4.073969	1.029574	Up	1.41E-06	2.99E-06
Wdr44	4020	541.5	1180	4.806575	9.804587	1.028448	Up	0	0
Rc3h1	3195	970	2119	10.85569	22.14067	1.028248	Up	0	0
mrpl11	812	486	1059.5	21.35562	43.55157	1.028109	Up	1.59E-13	5.89E-13
Smg8	3215	656.5	1432	7.300393	14.88769	1.028074	Up	1.14E-12	3.56E-12
Upf3b	2350	876.5	1913.5	13.3304	27.17984	1.027816	Up	1.50E-12	4.58E-12
Cnot2	1810	1423.5	3119	28.18737	57.46567	1.027651	Up	4.31E-12	1.20E-11

Slirp	2827	1079.5	2360.5	13.67826	27.87348	1.027009	Up	2.91E-12	8.45E-12
Hiat1	2727	1513	3294.5	19.77595	40.28573	1.026522	Up	2.80E-13	9.96E-13
LOC100361008	441	70.5	153	5.665186	11.53828	1.026233	Up	2.02E-08	4.63E-08
LOC689665	594	18	39	1.078548	2.196609	1.026188	Up	0.005355	0.008583
Cycs	1228	2274	4943	65.98337	134.3512	1.025835	Up	0	0
Rbm28	3631	1581	3451.5	15.58143	31.7238	1.025738	Up	5.17E-12	1.41E-11
MGC95208	1371	351.5	766.5	9.159754	18.64525	1.025428	Up	9.48E-14	3.68E-13
Dll1	3171	200	437.5	2.262692	4.605071	1.025183	Up	0	0
Rimbp2	6862	2506.5	5470.5	13.07886	26.60858	1.024655	Up	0	0
Nhs	5813	886	1935.5	5.45634	11.09938	1.024474	Up	0	0
Arhgap30	3672	66	143.5	0.640976	1.303883	1.024471	Up	7.70E-08	1.72E-07
RGD1559962	630	404.5	882.5	23.0056	46.79303	1.024309	Up	2.91E-13	1.03E-12
Nr1h4	2070	178	392.5	3.10887	6.322982	1.024215	Up	1.14E-13	4.36E-13
Ryr3	15122	38	83	0.089692	0.182357	1.023716	Up	3.79E-05	7.34E-05
LOC684988	528	7116	15431	479.5822	975.0536	1.023703	Up	1.68E-11	4.34E-11
RGD1561431	2555	64.5	141	0.904568	1.838607	1.023311	Up	5.69E-08	1.28E-07
Gpx8	1062	5954.5	12938.5	200.2177	406.6475	1.022209	Up	3.14E-11	7.95E-11
Dctn3	956	869	1884.5	32.39065	65.78546	1.022191	Up	1.49E-13	5.54E-13
LOC100365062	1496	1859.5	4051	44.46275	90.30065	1.022139	Up	5.01E-12	1.37E-11
RGD1304879	1627	86	187	1.888611	3.834563	1.021736	Up	6.72E-10	1.62E-09
C1galt1c1	1356	814	1761.5	21.3752	43.38114	1.02113	Up	0	0
LOC100910706	1269	145	313.5	4.070631	8.261163	1.021092	Up	5.55E-15	2.32E-14
Klhl11	2441	859	1862.5	12.5681	25.50368	1.020939	Up	0	0
LOC100912665	1267	334.5	723.5	9.400722	19.07033	1.020486	Up	3.40E-13	1.18E-12
LOC100909481	1341	812.5	1770	21.66898	43.95351	1.020347	Up	1.63E-12	4.97E-12

Spag1	3681	68	147.5	0.661655	1.341376	1.019562	Up	5.45E-08	1.23E-07
Plscr1	1569	457	990	10.38707	21.05764	1.019555	Up	1.73E-12	5.23E-12
Rbx1	1549	2323	5031	53.46598	108.3767	1.019361	Up	0	0
Derl3	1357	26.5	57	0.695196	1.408363	1.018527	Up	0.000628	0.001084
RGD1305158	660	132.5	288	7.166813	14.51015	1.017659	Up	1.29E-13	4.86E-13
Tgif2	714	23	50	1.156697	2.341794	1.017603	Up	0.001516	0.002543
LOC691979	2039	1266.5	2740.5	22.14125	44.8183	1.017351	Up	4.30E-12	1.20E-11
LOC100359951	395	1393.5	3002	125.353	253.6304	1.016731	Up	4.69E-12	1.30E-11
LOC100365822	506	744	1616.5	52.63675	106.4987	1.016693	Up	0	0
Hivep2	9731	2268.5	4922.5	8.344856	16.88014	1.016368	Up	0	0
Ttc1	1471	1518	3280.5	36.87236	74.54241	1.015522	Up	2.68E-12	7.84E-12
LOC361985	1583	1760.5	3806.5	39.72439	80.30761	1.015512	Up	6.29E-12	1.69E-11
Rnf7	1256	1102	2383.5	31.3264	63.3145	1.015157	Up	2.55E-12	7.49E-12
Tead3	2576	420.5	912	5.851074	11.81878	1.014307	Up	1.49E-13	5.55E-13
Kcnt1	3714	467	1012.5	4.505112	9.09903	1.01415	Up	6.78E-13	2.20E-12
Zdhhc4	1279	503.5	1092.5	14.1129	28.4998	1.013937	Up	3.35E-13	1.17E-12
Tceal1	1215	131	282	3.82932	7.732	1.013753	Up	1.47E-13	5.46E-13
Surf1	981	80	173	2.913426	5.882282	1.013659	Up	3.59E-09	8.47E-09
Grk4	1997	201	432.5	3.595587	7.25711	1.013168	Up	0	0
Dcp1a	1806	461	1001	9.149595	18.46672	1.013148	Up	7.39E-13	2.38E-12
Bloc1s2	936	1370.5	2951.5	52.18983	105.3266	1.01303	Up	2.05E-12	6.13E-12
Snw1	2275	1904.5	4111.5	29.89279	60.32397	1.012934	Up	4.81E-12	1.32E-11
Faf2	1994	1492	3228	26.76599	53.98662	1.012201	Up	1.99E-12	5.94E-12
Rnf20	4234	1992	4296.5	16.81325	33.9059	1.011938	Up	2.46E-13	8.82E-13
LOC100911902	624	23	49.5	1.318014	2.657848	1.011893	Up	0.002118	0.003502

RGD1311080	3047	20	43	0.23337	0.470572	1.011799	Up	0.00369	0.006004
Ankrd39	1095	304.5	657	9.942563	20.04688	1.011688	Up	0	0
Mmp3	1771	3492	7530.5	70.38493	141.9095	1.011633	Up	0	0
RGD1562500	1071	25.5	55	0.851714	1.716223	1.010796	Up	0.000752	0.001292
Ino80c	2547	805.5	1735	11.29803	22.76444	1.01071	Up	1.90E-12	5.71E-12
Ints7	3415	2260	4885.5	23.67682	47.70428	1.010643	Up	0	0
Dnm3	3252	88	190.5	0.969538	1.953148	1.010431	Up	6.57E-10	1.59E-09
Sp140	3003	559	1207	6.663431	13.4228	1.010349	Up	3.48E-13	1.21E-12
Cdk6	1503	903.5	1950	21.50374	43.31323	1.01022	Up	0	0
Mrpl32	768	876.5	1889.5	40.71796	82.01162	1.010163	Up	1.50E-12	4.58E-12
Ilkap	1318	970	2093	26.30905	52.98452	1.010012	Up	0	0
Btf3l4	1794	1026.5	2211.5	20.40543	41.09113	1.009874	Up	2.32E-12	6.87E-12
Txnl4b	1743	259.5	557.5	5.322682	10.71727	1.009712	Up	1.39E-13	5.21E-13
Katna1	1852	522.5	1127	10.08939	20.30574	1.009048	Up	0	0
Luc7l2	2647	3886.5	8382	52.51818	105.6953	1.009022	Up	2.56E-12	7.50E-12
Ptcd1	2981	470	1009.5	5.623881	11.31789	1.008967	Up	1.65E-12	5.01E-12
Mmp10	1717	1049	2256	21.80998	43.88908	1.008874	Up	1.06E-12	3.33E-12
Wbp4	1574	652.5	1405	14.81878	29.81923	1.008816	Up	1.68E-12	5.10E-12
Acyp2	1156	402.5	864	12.39241	24.93556	1.008748	Up	1.18E-14	4.87E-14
Hspb11	735	219	473	10.66662	21.4624	1.008708	Up	3.38E-13	1.18E-12
Arpp19	339	366.5	787.5	38.62088	77.63884	1.007398	Up	0	0
Chd1	6568	2866	6164.5	15.58296	31.31729	1.00699	Up	0	0
Prkg1	2016	146.5	317	2.608141	5.241329	1.00691	Up	3.40E-14	1.38E-13
Ticam2	3315	97	209	1.047916	2.104387	1.005877	Up	1.01E-10	2.52E-10
Scai	2040	155.5	334.5	2.726892	5.473352	1.005167	Up	3.73E-13	1.29E-12

Yae1d1	3433	1231	2635.5	12.78316	25.63315	1.003766	Up	3.14E-12	9.07E-12
Ttc30a1	2466	36	77.5	0.523546	1.049809	1.003739	Up	0.000104	0.000194
Ppcs	1371	154	330	4.00143	8.023083	1.003641	Up	2.09E-13	7.60E-13
LOC100911627	774	142	305.5	6.567846	13.16799	1.003542	Up	2.97E-13	1.05E-12
LOC100360828	339	1121	2402.5	118.0077	236.5745	1.003413	Up	4.79E-12	1.32E-11
Fbxl7	3473	18	39	0.186119	0.373103	1.003346	Up	0.005355	0.008582
Taf2	5042	1726.5	3697.5	12.20475	24.46487	1.003269	Up	1.07E-12	3.35E-12
RGD1565641	412	762	1636.5	66.12315	132.5378	1.003176	Up	1.46E-12	4.47E-12
LOC100910516	507	112.5	241.5	7.92706	15.8881	1.003089	Up	4.01E-12	1.13E-11
Elp6	1303	421	906	11.5629	23.1751	1.003072	Up	4.13E-13	1.41E-12
Bnip3	1641	6153	13171	133.6616	267.8876	1.003043	Up	1.71E-11	4.42E-11
Intu	2636	180.5	388	2.449712	4.909515	1.002969	Up	3.16E-13	1.11E-12
Klhl2	3009	2558	5485	30.37379	60.86622	1.002815	Up	0	0
Zfp74	2809	207	442.5	2.628353	5.266613	1.002716	Up	1.16E-13	4.45E-13
Lin9	3163	954.5	2045.5	10.77738	21.59468	1.002669	Up	1.35E-12	4.16E-12
Lrrc32	3953	41.5	89.5	0.375883	0.753066	1.002493	Up	2.23E-05	4.39E-05
Ccdc99	2310	955.5	2049	14.78946	29.62428	1.002211	Up	1.30E-12	4.01E-12
LOC100911033	1818	433.5	929	8.507395	17.03941	1.002086	Up	1.10E-12	3.43E-12
RGD1565495	1371	84	179.5	2.188226	4.382045	1.001843	Up	3.40E-09	8.01E-09
Igbp1	1464	649.5	1389	15.83147	31.69241	1.001342	Up	2.14E-12	6.35E-12
Calm2	1112	11829	25257	379.0116	758.5391	1.000982	Up	0	0
Kif21a	6087	1574	3374.5	9.242604	18.49702	1.000921	Up	5.47E-12	1.49E-11
RGD1560568	555	24	51.5	1.539117	3.079406	1.000549	Up	0.001762	0.002938
Naa20	1173	1220	2610	37.13341	74.28702	1.000393	Up	7.20E-12	1.92E-11
Gdnf	700	126	271	6.447536	12.89846	1.00038	Up	3.09E-13	1.09E-12

RGD1564400	984	345.5	741	12.5586	25.1236	1.000368	Up	9.33E-13	2.96E-12
Rpl24	541	9923.5	21147	652.2227	1304.502	1.000063	Up	0	0
C1ql4	873	11.5	0	0.473013	0.001	-8.88574	Down	0.000488	0.000865
Hhipl1	2578	9	0	0.12381	0.001	-6.95198	Down	0.001953	0.003239
Ankrd22	714	25	1	1.257733	0.04676	-4.7494	Down	4.17E-07	9.05E-07
Serpinb8	3029	202.5	9.5	2.389498	0.103376	-4.53074	Down	6.38E-49	3.62E-48
Trim54	1450	16	1	0.39564	0.023025	-4.10289	Down	0.000145	0.000265
Plk1	2198	6371.5	447	104.0135	6.774615	-3.94049	Down	0	0
Ucp1	1222	13.5	1	0.393672	0.028058	-3.81051	Down	0.000976	0.001662
Pcdh8	2919	39	3	0.47593	0.034005	-3.80693	Down	3.02E-09	7.15E-09
Rs1	675	12.5	1	0.666475	0.049462	-3.75216	Down	0.001831	0.00305
Adam11	2528	12	1	0.169403	0.012851	-3.72054	Down	0.001831	0.00305
Ppp1r1b	1764	486	40	9.866773	0.759111	-3.70019	Down	9.92E-99	7.01E-98
Fam181b	1522	1718.5	143	40.45941	3.130364	-3.69207	Down	0	0
Vom1r95	945	28.5	2.5	1.088582	0.086896	-3.64701	Down	4.63E-07	1.00E-06
Fbn2	8756	556.5	51	2.274352	0.193642	-3.55399	Down	########	########
Olfml2b	3122	197	19	2.261984	0.200592	-3.49525	Down	9.46E-39	5.05E-38
RGD1565157	1341	26.5	2.5	0.696222	0.061907	-3.49138	Down	1.62E-06	3.41E-06
Lypd6	709	197	19	9.964428	0.906135	-3.45899	Down	9.46E-39	5.04E-38
Klrb1c	654	79	8	4.334715	0.407025	-3.41275	Down	4.60E-16	1.96E-15
Cdc20	1777	4389	445	88.46724	8.339015	-3.40719	Down	0	0
Svs4	597	28.5	3	1.726015	0.16325	-3.40229	Down	2.56E-06	5.32E-06
Gpr37	3104	628.5	64	7.240702	0.687808	-3.39605	Down	########	########
LOC100365744	995	836.5	86	30.13467	2.892931	-3.38082	Down	########	########
Sox12	1020	647	67.5	22.81136	2.195744	-3.37697	Down	########	########

Sdk2	6162	3768.5	400	21.92358	2.16435	-3.34048	Down	0	0
Chtf18	3100	1290	139.5	14.90154	1.504294	-3.3083	Down	########	########
Gli1	3501	120	13.5	1.232072	0.129641	-3.2485	Down	4.06E-23	1.88E-22
Tcf7	2964	1151	133	13.91357	1.49843	-3.21497	Down	########	########
Tubb2b	1700	4155	489	87.55592	9.610501	-3.18752	Down	0	0
LOC100911871	648	33	4	1.832029	0.206091	-3.15209	Down	6.04E-07	1.30E-06
Lrrc15	1737	420.5	51.5	8.70002	0.985993	-3.14137	Down	1.71E-73	1.11E-72
Fam64a	1487	3201	390	76.98543	8.731039	-3.14036	Down	0	0
Negr1	1809	24	3	0.474102	0.053875	-3.1375	Down	2.74E-05	5.37E-05
Ntng1	5085	45	5.5	0.316779	0.036023	-3.13648	Down	2.33E-09	5.53E-09
Igfbp5	1630	6404.5	788.5	141.0781	16.1658	-3.12548	Down	0	0
RGD1307119	2041	122	15	2.143986	0.248458	-3.10922	Down	2.84E-22	1.31E-21
Wnt4	1211	390.5	48.5	11.53751	1.338985	-3.10712	Down	6.18E-68	3.90E-67
Scg3	2144	54.5	7	0.921236	0.109425	-3.07363	Down	2.43E-10	5.96E-10
Fasn	9136	21488	2745	84.20284	10.03179	-3.06929	Down	0	0
Scd1	4475	2954.5	377.5	23.63332	2.815927	-3.06914	Down	0	0
Mpz	1029	47	6	1.628284	0.194675	-3.06421	Down	3.26E-09	7.68E-09
Olr428	936	15.5	2	0.594708	0.072301	-3.0401	Down	0.001312	0.002211
Olr1568	939	71	9.5	2.730693	0.335383	-3.02539	Down	2.46E-13	8.83E-13
Crmp1	2846	1517	203	19.10634	2.381421	-3.00416	Down	########	########
Ace	4142	2926	399.5	25.30381	3.219213	-2.97457	Down	0	0
Jup	3463	3390.5	464.5	35.14363	4.482537	-2.97088	Down	0	0
Cd93	4779	583	80	4.36131	0.557197	-2.9685	Down	1.76E-95	1.23E-94
Mafa	1086	21	3	0.696165	0.0914	-2.92916	Down	0.000157	0.000286
Tm4sf1	1950	6557.5	924	120.1754	15.84006	-2.92349	Down	0	0

Taar4	1044	21.5	3	0.739387	0.097663	-2.92044	Down	0.000157	0.000286
Rtn4rl2	1304	654	94.5	18.03973	2.408821	-2.90478	Down	########	########
Pcdha1	5244	49.5	7	0.337388	0.045082	-2.9038	Down	4.24E-09	9.96E-09
Fdps	1271	17361.5	2541.5	489.9513	66.81041	-2.87449	Down	0	0
Ranbp1	1052	8507	1246	290.0186	39.58647	-2.87307	Down	0	0
Erbb3	4224	2905.5	432.5	24.628	3.415636	-2.85007	Down	0	0
Tmprss3	2691	19.5	3	0.260258	0.036217	-2.8452	Down	0.000488	0.000866
Rab3d	2226	890.5	133.5	14.32353	2.008172	-2.83443	Down	########	########
Taar8a	1125	16.5	2.5	0.525076	0.073793	-2.83097	Down	0.000729	0.001252
LOC100912950	1047	26	4	0.886409	0.125833	-2.81646	Down	3.40E-05	6.59E-05
Fgfrl1	2351	1774.5	272.5	27.07268	3.86808	-2.80715	Down	########	########
LOC313641	14269	10936	1693.5	27.44601	3.967175	-2.79041	Down	0	0
Fads1	3380	8925.5	1385	94.60516	13.67909	-2.78995	Down	0	0
Cenpf	10069	9506	1473.5	33.71313	4.883108	-2.78744	Down	0	0
Ccrl2	1979	26	4	0.470698	0.068392	-2.78291	Down	3.40E-05	6.59E-05
Bace2	1545	634	100	14.69574	2.149308	-2.77345	Down	5.13E-96	3.60E-95
Klf13	602	249	39.5	14.90358	2.18543	-2.76967	Down	7.50E-39	4.00E-38
Iqgap3	5795	3132	495.5	19.35941	2.852479	-2.76275	Down	0	0
Cspg4	8074	11462.5	1824	50.84113	7.543763	-2.75264	Down	0	0
Cdc25b	2804	2126.5	339.5	27.15562	4.050561	-2.74506	Down	0	0
LOC100910695	5042	2869	460	20.34705	3.043144	-2.74119	Down	0	0
Cmklr1	2530	31	5	0.440491	0.066338	-2.73121	Down	7.43E-06	1.50E-05
Sv2b	5213	467.5	76	3.202153	0.485363	-2.72191	Down	7.23E-70	4.60E-69
Ppp1r9a	3846	57.5	9.5	0.53595	0.081416	-2.71872	Down	6.81E-10	1.64E-09
Paqr4	2033	1430.5	233.5	25.26835	3.839287	-2.71842	Down	########	########

Plxnd1	6104	1372.5	225.5	8.072585	1.23186	-2.71219	Down	########	########
Slc25a23	3184	152	25	1.714008	0.261863	-2.71049	Down	1.25E-23	5.82E-23
Rxra	1404	631.5	104.5	16.14811	2.475051	-2.70584	Down	4.40E-93	3.06E-92
F5	6315	79	13	0.44828	0.068872	-2.7024	Down	6.22E-13	2.04E-12
Prex2	4852	415	68.5	3.051384	0.470888	-2.69601	Down	5.11E-62	3.13E-61
Selenbp1	1685	429	71	9.116175	1.413747	-2.6889	Down	1.14E-63	7.07E-63
Nkain1	2429	2091.5	347	30.8702	4.789554	-2.68825	Down	########	########
Arhgef17	7405	1880	314.5	9.098134	1.418285	-2.68142	Down	########	########
Tgfbi	2696	4231	709	56.07347	8.780137	-2.675	Down	0	0
LOC363060	1312	109.5	18.5	2.987137	0.469059	-2.67092	Down	2.65E-17	1.15E-16
Col15a1	5193	482	82	3.319755	0.527194	-2.65467	Down	4.03E-70	2.57E-69
Fkbp5	3508	2834.5	482	28.92246	4.597618	-2.65323	Down	0	0
LOC681309	3174	689.5	118	7.783715	1.239806	-2.65034	Down	4.46E-99	3.16E-98
Abca7	6513	1278	218	7.031176	1.120547	-2.64956	Down	########	########
Metrn	924	2854.5	493.5	110.6765	17.77656	-2.6383	Down	0	0
Fam149a	3966	1029.5	177.5	9.295676	1.493673	-2.63769	Down	########	########
XAF1	1011	172	30	6.089452	0.981804	-2.63281	Down	1.25E-25	5.94E-25
Nthl1	1084	813	141	26.86969	4.346901	-2.62792	Down	########	########
Limk1	3290	2899	509	31.60727	5.157929	-2.61539	Down	0	0
Six3	1598	203	36	4.565057	0.751017	-2.60372	Down	1.13E-29	5.61E-29
Kif21b	5387	1467	260.5	9.771183	1.614575	-2.59738	Down	########	########
Tubb5	2395	87175.5	15539.5	1306.537	216.635	-2.59241	Down	0	0
Avp	585	16.5	3	1.013682	0.168137	-2.59189	Down	0.002577	0.004234
LOC100912243	844	57	10	2.403731	0.399844	-2.58777	Down	2.36E-09	5.60E-09
Pif1	1915	106	19	1.9756	0.329843	-2.58244	Down	4.52E-16	1.93E-15

LOC100911260	4927	2855	515.5	20.79164	3.489436	-2.57494	Down	0	0
Dip2a	5445	1719	310	11.31455	1.899488	-2.5745	Down	########	########
RGD1564114	1739	17	3	0.352116	0.059149	-2.57362	Down	0.00149	0.002499
Elovl6	2490	857.5	154.5	12.28483	2.064181	-2.57324	Down	########	########
Capn5	2150	2172	391.5	36.19393	6.082227	-2.57308	Down	########	########
Odf4	1464	19.5	3.5	0.478775	0.08074	-2.56799	Down	0.000488	0.000866
LOC100911959	2411	1984.5	359	29.4365	4.968339	-2.56677	Down	########	########
Usp5	3207	3981	720.5	44.4246	7.499017	-2.56659	Down	0	0
Hist1h2bc	381	81	15	7.645086	1.293181	-2.56361	Down	2.38E-12	7.02E-12
Map1a	10120	6943	1273.5	24.57299	4.207842	-2.54592	Down	0	0
Tgfb1	1482	14321	2639	347.2647	59.47321	-2.54572	Down	0	0
Col9a3	2845	263	48	3.307301	0.567089	-2.54401	Down	2.72E-37	1.44E-36
Tcf3	2562	4610.5	850.5	64.50648	11.07542	-2.54208	Down	0	0
Olr1581	933	13.5	2.5	0.518071	0.088979	-2.54162	Down	0.004181	0.006747
Tril	4859	231	42.5	1.701394	0.292301	-2.54119	Down	5.05E-33	2.58E-32
LOC100909468	2758	914.5	169.5	11.9115	2.049751	-2.53883	Down	########	########
Acaca	7038	3578.5	660	18.18122	3.129624	-2.53839	Down	0	0
Ssbp4	1525	2352.5	436.5	55.46382	9.558947	-2.53662	Down	0	0
Nfix	1445	4676	868.5	116.1044	20.0191	-2.53597	Down	0	0
Col23a1	2733	16	3	0.210328	0.036319	-2.53383	Down	0.002577	0.004234
Mmp14	2448	3477	646.5	50.94581	8.799764	-2.53343	Down	0	0
Rnf144a	1657	145	27	3.130957	0.543479	-2.52631	Down	5.89E-21	2.68E-20
Kcna1	1746	217	40.5	4.451556	0.775725	-2.52069	Down	7.76E-31	3.88E-30
Efhd1	1832	113.5	21	2.219893	0.387622	-2.51777	Down	1.16E-16	5.00E-16
Pcdhac2	5418	727.5	138	4.810337	0.845068	-2.509	Down	1.67E-97	1.18E-96

Tnfaip8l1	804	125	23.5	5.579718	0.982015	-2.50637	Down	2.12E-18	9.34E-18
Prph	1753	591.5	111.5	12.10462	2.130508	-2.50629	Down	3.52E-80	2.35E-79
Itga6	5979	28819.5	5432.5	172.4278	30.35118	-2.50617	Down	0	0
Hapln1	2191	422	79.5	6.879507	1.214104	-2.50241	Down	7.26E-58	4.35E-57
Htr1b	1161	123	23.5	3.804324	0.671524	-2.50213	Down	6.08E-18	2.66E-17
Lmnb1	2154	5091.5	966	84.75444	14.97668	-2.50057	Down	0	0
Nfic	1565	4245.5	810	97.40637	17.28238	-2.49471	Down	0	0
Hspa12a	6331	58	11	0.327458	0.058151	-2.49343	Down	4.47E-09	1.05E-08
Rps6ka4	3119	4170	794.5	47.91399	8.512527	-2.49279	Down	0	0
Abhd8	1982	1864	357	33.76106	5.998684	-2.49264	Down	########	########
Dsp	9607	623	118.5	2.318163	0.412521	-2.49044	Down	6.20E-84	4.20E-83
RGD1563510	5590	1964	376.5	12.59676	2.244094	-2.48885	Down	########	########
Dhcr24	1551	12407.5	2375.5	286.6834	51.11207	-2.48772	Down	0	0
Zbtb42	1727	170	32.5	3.535661	0.634813	-2.47758	Down	3.80E-24	1.78E-23
Gas2l1	2725	1779.5	345	23.45929	4.222	-2.47416	Down	########	########
Gpr125	4043	1307.5	252.5	11.56891	2.082122	-2.47413	Down	########	########
Prlhr	1452	170	32.5	4.181204	0.753183	-2.47285	Down	3.80E-24	1.78E-23
Slc6a17	6411	535	103	2.988627	0.539907	-2.4687	Down	1.69E-71	1.09E-70
Fbxo23	1632	377.5	73	8.269159	1.494508	-2.46807	Down	1.14E-50	6.51E-50
Racgap1	2474	3600.5	700.5	52.03408	9.447296	-2.46148	Down	0	0
Grap2	1288	31	6	0.862578	0.156926	-2.45857	Down	2.43E-05	4.78E-05
S100a16	1036	59.5	11.5	2.043218	0.37191	-2.45782	Down	2.63E-09	6.24E-09
Hcfc1	9128	6508	1273	25.54703	4.653792	-2.45668	Down	0	0
LOC100909752	5193	856	168	5.904705	1.077332	-2.4544	Down	########	########
Tp53i11	2664	1168.5	229.5	15.73582	2.872681	-2.45358	Down	########	########

Shf	1444	477.5	94	11.85598	2.168393	-2.45092	Down	7.07E-63	4.35E-62
Sdc3	4841	4098	806.5	30.36061	5.566539	-2.44735	Down	0	0
Htra3	2440	152	30	2.23382	0.410494	-2.44408	Down	4.60E-21	2.09E-20
Plcb3	3726	2843	561.5	27.34999	5.032165	-2.44229	Down	0	0
Scx	1134	313	62	9.895387	1.825381	-2.43856	Down	1.14E-41	6.21E-41
Gper	1278	836	166.5	23.44519	4.335253	-2.4351	Down	########	########
Itga9	4495	1330	264.5	10.59076	1.963284	-2.43147	Down	########	########
Ttyh3	4473	5269.5	1050.5	42.22144	7.82904	-2.43107	Down	0	0
LOC100909901	2115	2753.5	549	46.60635	8.654023	-2.42908	Down	0	0
Pik3cd	4788	87.5	17.5	0.656269	0.121934	-2.42819	Down	9.95E-13	3.14E-12
Cyb5r3	1893	6680.5	1339	126.7419	23.61307	-2.42424	Down	0	0
Ncan	5201	145.5	29	1.003641	0.187025	-2.42394	Down	5.62E-20	2.52E-19
Crat	2757	3273	660	42.60522	7.991511	-2.41449	Down	0	0
Kctd17	1634	1023.5	206	22.43924	4.21021	-2.41406	Down	########	########
Ttll12	2412	4082	826	60.73184	11.42525	-2.41023	Down	0	0
Nkx2-2	1393	634.5	129	16.38068	3.08277	-2.4097	Down	4.51E-81	3.03E-80
Idh2	1695	3663.5	738.5	77.3943	14.56791	-2.40943	Down	0	0
Angptl2	2138	3928.5	794.5	65.91042	12.42389	-2.40739	Down	0	0
LOC366669	20881	2951	595.5	5.044256	0.951051	-2.40705	Down	0	0
Col13a1	3146	69	14	0.784638	0.148002	-2.40641	Down	4.07E-10	9.90E-10
Mc4r	1888	71	14.5	1.353255	0.255698	-2.40392	Down	1.45E-10	3.57E-10
Hnrnpa0	681	941	192	49.74642	9.405092	-2.40308	Down	########	########
Rcc2	3497	12542.5	2542	128.2978	24.26402	-2.40261	Down	0	0
Tcf7l1	2736	326.5	66.5	4.292506	0.815268	-2.39647	Down	1.18E-42	6.47E-42
LOC686288	1685	19.5	4	0.419043	0.079791	-2.39281	Down	0.001544	0.002588

Trpm4	4203	713	145.5	6.073432	1.157823	-2.3911	Down	5.44E-91	3.77E-90
Trim40	744	17	3.5	0.826106	0.157666	-2.38945	Down	0.00149	0.0025
Cnp	2326	3281	673	50.53661	9.660845	-2.38711	Down	0	0
Slc43a3	2030	575	118.5	10.14694	1.950486	-2.37914	Down	3.22E-73	2.09E-72
Pitpnm1	4191	1144.5	236.5	9.795747	1.885214	-2.37743	Down	########	########
Sh3glb2	1849	2066.5	428	40.07201	7.717553	-2.37638	Down	########	########
Cyfip2	6253	3511	728.5	20.10313	3.88862	-2.37009	Down	0	0
Lmf2	2760	3003.5	625	39.03338	7.560093	-2.36823	Down	0	0
Dctn1	4432	6895.5	1434.5	55.75509	10.80515	-2.36738	Down	0	0
Osbp2	3952	681	142	6.171747	1.200083	-2.36255	Down	2.38E-85	1.62E-84
Syt7	1440	300	63	7.464796	1.453798	-2.36028	Down	2.53E-38	1.34E-37
Azi1	3600	606	127	6.034661	1.177063	-2.35808	Down	7.56E-76	4.95E-75
Trrap	12520	5452	1141	15.57688	3.04153	-2.35654	Down	0	0
Mepe	1655	6594.5	1380.5	142.7758	27.88541	-2.35617	Down	0	0
Gpd1	2848	2124	446	26.71867	5.226516	-2.35393	Down	########	########
Qprt	1225	164	34.5	4.795253	0.938445	-2.35326	Down	7.69E-22	3.52E-21
Alg9	2597	1133.5	238	15.58888	3.051044	-2.35314	Down	########	########
Mpa2l	1692	33	7	0.697561	0.136529	-2.35311	Down	2.53E-05	4.96E-05
Fxyd4	1362	52.5	11	1.37531	0.269644	-2.35063	Down	1.01E-07	2.24E-07
Plcd1	2791	1737	365.5	22.31496	4.384963	-2.34737	Down	########	########
Ibsp	1948	267	56	4.89575	0.963943	-2.34451	Down	2.59E-34	1.34E-33
Ehd1	3250	2427	515	26.75517	5.269479	-2.34409	Down	########	########
Pde7b	1754	56	12	1.14376	0.225337	-2.34363	Down	3.74E-08	8.50E-08
Ptov1	1748	2865	608.5	58.71457	11.60511	-2.33896	Down	0	0
Cbs	2539	32.5	7	0.458375	0.090984	-2.33285	Down	4.23E-05	8.16E-05

Sorcs2	5714	1472.5	314	9.237059	1.834067	-2.33239	Down	########	########
Erf	3711	4096.5	873.5	39.59444	7.868211	-2.33119	Down	0	0
Pde1c	3187	218.5	47	2.452481	0.488698	-2.32723	Down	1.02E-27	4.98E-27
Bckdk	1816	3396.5	727.5	67.01623	13.37817	-2.32463	Down	0	0
LOC100125365	1785	2714.5	582.5	54.59664	10.91757	-2.32216	Down	0	0
Carm1	3156	3884	836.5	44.15443	8.834511	-2.32134	Down	0	0
Nova2	1851	18.5	4	0.360582	0.072149	-2.32128	Down	0.002599	0.00427
Tuba1c	1559	4185.5	911.5	96.55821	19.41432	-2.31428	Down	0	0
Akt1	1613	8131	1757	180.6103	36.35513	-2.31265	Down	0	0
Pcdha10	5337	37	8	0.248613	0.050046	-2.31258	Down	9.25E-06	1.86E-05
Map2k6	1690	366	79	7.757648	1.566543	-2.30803	Down	1.99E-45	1.10E-44
LOC100363275	5461	9965	2168.5	65.36996	13.24458	-2.30323	Down	0	0
Alkbh2	1235	155.5	34	4.504804	0.916236	-2.29767	Down	7.12E-20	3.20E-19
LOC686539	2081	234	51	4.025884	0.819523	-2.29645	Down	2.16E-29	1.07E-28
Pcdha3	5226	47.5	10.5	0.325624	0.066477	-2.29227	Down	4.52E-07	9.79E-07
Sh2d3c	3078	32	7	0.373133	0.076221	-2.29143	Down	4.23E-05	8.16E-05
Pou6f1	2231	23	5	0.368128	0.075228	-2.29086	Down	0.000546	0.000962
RGD1311575	6819	2330.5	511.5	12.23129	2.500748	-2.29014	Down	########	########
RGD1564899	4909	236	52	1.724016	0.352926	-2.28833	Down	2.22E-29	1.10E-28
LOC498426	2062	893.5	197	15.55457	3.195394	-2.28327	Down	########	########
Arhgef1	3243	6413	1414.5	70.90527	14.57024	-2.28287	Down	0	0
LOC100912782	1562	17186.5	3825.5	394.6726	81.57502	-2.27446	Down	0	0
RGD1311021	1857	680.5	150.5	13.10833	2.712367	-2.27286	Down	1.62E-81	1.09E-80
RGD1563946	3025	1006	223.5	11.91834	2.467207	-2.27223	Down	########	########
Ppp2r5b	2213	530.5	118	8.580412	1.776567	-2.27195	Down	1.94E-63	1.20E-62

Pcdha7	5211	54	12	0.371141	0.076884	-2.27121	Down	1.03E-07	2.29E-07
Svil	8155	4318.5	957.5	18.94224	3.925165	-2.27078	Down	0	0
Agrn	7286	5296.5	1179	26.02292	5.403301	-2.26787	Down	0	0
Lrp8	3176	2217	495	24.97245	5.193347	-2.2656	Down	########	########
Cxcl17	795	18	4	0.807301	0.167984	-2.26478	Down	0.002599	0.00427
Pllp	1475	263.5	59	6.382549	1.32815	-2.26471	Down	4.21E-32	2.13E-31
Kazald1	1645	1487	331.5	32.347	6.734394	-2.26401	Down	########	########
Maz	1434	3558	804	89.2574	18.62984	-2.26036	Down	0	0
Myh14	6443	274.5	61.5	1.52608	0.318895	-2.25868	Down	1.39E-33	7.12E-33
Mvd	1704	2584	580	54.33149	11.35455	-2.25852	Down	########	########
Acan	6939	2111	473.5	10.88565	2.276092	-2.2578	Down	########	########
Slc25a1	1669	3290.5	739.5	70.65578	14.77981	-2.25718	Down	0	0
Arhgdia	1963	28151	6323	513.9222	107.5722	-2.25624	Down	0	0
Tuba1b	780	61529.5	13849.5	2826.859	591.9252	-2.25571	Down	0	0
RGD1561381	459	27	6	2.101148	0.440351	-2.25445	Down	0.000195	0.000354
LOC100909441	1552	8309	1887.5	192.4264	40.40087	-2.25185	Down	0	0
LOC100911365	1091	187.5	42.5	6.153673	1.293574	-2.25009	Down	3.05E-23	1.41E-22
Cdh3	3169	613	139	6.93499	1.463006	-2.24496	Down	5.27E-72	3.40E-71
Srebf2	5060	11260.5	2549.5	79.77514	16.83105	-2.24481	Down	0	0
LOC652956	2157	3843	878.5	64.04797	13.55121	-2.24073	Down	0	0
Nkd2	4024	4174.5	947.5	37.1913	7.87151	-2.24025	Down	0	0
Mageh1	1280	139.5	31.5	3.888389	0.82409	-2.2383	Down	9.57E-18	4.19E-17
Tuba1a	1617	41343.5	9421	915.3899	194.2529	-2.23645	Down	0	0
LOC683470	3158	73.5	17	0.837094	0.177731	-2.23569	Down	1.19E-09	2.86E-09
Tmem132a	3066	6189	1412	72.36955	15.37491	-2.2348	Down	0	0

Sema3f	3485	1743.5	398	17.95215	3.815481	-2.23422	Down	########	########
Pddc1	1098	236	54	7.705215	1.638696	-2.23329	Down	1.66E-28	8.11E-28
Fhod1	3713	3797.5	867	36.66268	7.800557	-2.23266	Down	0	0
6-Sep	2088	285	65	4.882283	1.039771	-2.23129	Down	3.40E-34	1.75E-33
St3gal2	3221	4304	983.5	47.82535	10.1886	-2.23082	Down	0	0
Mcm3	2918	4518.5	1032.5	55.44042	11.81338	-2.23052	Down	0	0
Chst12	1634	928	214.5	20.43964	4.356071	-2.23027	Down	########	########
Peg12	2604	242	55	3.321954	0.708977	-2.22822	Down	2.36E-29	1.17E-28
Pycr1	930	397	91	15.29926	3.267849	-2.22705	Down	8.33E-47	4.67E-46
LOC685707	9398	11439.5	2627.5	43.61615	9.338764	-2.22356	Down	0	0
Aqp1	2623	3911.5	898.5	53.31309	11.43122	-2.22151	Down	0	0
Siat7F	2243	841.5	194	13.4417	2.885261	-2.21994	Down	1.04E-96	7.28E-96
Igfbp4	2194	16782	3861.5	273.6999	58.78816	-2.219	Down	0	0
Cables1	2600	153	35	2.105706	0.452553	-2.21814	Down	5.24E-19	2.32E-18
Cnpy3	1814	2563	592.5	50.64979	10.89335	-2.21711	Down	########	########
Mmp15	3818	3162.5	731	29.73502	6.397475	-2.21659	Down	0	0
Dpysl2	4370	11890	2745	97.28481	20.98211	-2.21306	Down	0	0
F2rl1	3011	538	124	6.380733	1.37674	-2.21247	Down	1.52E-62	9.37E-62
LOC100912604	1251	1936.5	448.5	55.54165	11.98436	-2.21242	Down	########	########
Cckbr	2152	506.5	117	8.409372	1.814757	-2.21222	Down	9.15E-59	5.52E-58
RGD1564836	276	348.5	81.5	45.58069	9.853895	-2.20966	Down	1.05E-40	5.67E-40
LOC100911751	774	29.5	7	1.367165	0.296134	-2.20687	Down	0.000191	0.000347
Dapk1	5878	1549.5	360	9.442235	2.047848	-2.20502	Down	########	########
LOC100911830	1820	423.5	99.5	8.287256	1.807218	-2.19712	Down	6.77E-49	3.84E-48
Pik3r2	3137	2913.5	684	33.32914	7.274587	-2.19585	Down	0	0

Pcdh20	5179	453	105.5	3.120056	0.681765	-2.19423	Down	1.12E-52	6.49E-52
Hspa8	2130	19738	4641.5	332.1669	72.74945	-2.1909	Down	0	0
Duox1	5212	304.5	72	2.090614	0.458452	-2.18909	Down	3.07E-35	1.60E-34
Nup210	6941	1422.5	335	7.339465	1.611768	-2.18703	Down	########	########
Ptgfrn	5825	2035	479.5	12.50129	2.749635	-2.18476	Down	########	########
Dpysl3	1792	10113	2389.5	202.2478	44.56883	-2.18202	Down	0	0
Ptrf	1393	7931.5	1880.5	203.9133	45.01317	-2.17954	Down	0	0
RGD1359378	1567	166.5	39.5	3.817664	0.843031	-2.17903	Down	4.61E-20	2.07E-19
Inpp5j	3322	113.5	27	1.226287	0.270814	-2.17892	Down	6.40E-14	2.52E-13
Flna	8412	56285	13326	239.2271	52.86145	-2.17809	Down	0	0
RGD1562342	2646	909.5	216	12.30278	2.719331	-2.17766	Down	########	########
Rnf213	15614	2229.5	528	5.096673	1.127273	-2.17672	Down	########	########
Git1	3236	4424	1050.5	49.00751	10.84237	-2.17632	Down	0	0
Mrf	5531	992	237	6.433372	1.425886	-2.17372	Down	########	########
LOC498045	1941	4600	1095.5	84.97655	18.83679	-2.17351	Down	0	0
Igsf3	6569	3578	849.5	19.47102	4.320102	-2.17219	Down	0	0
Sgsm2	4712	968	230.5	7.353245	1.634446	-2.16958	Down	########	########
Ipo4	3717	5724	1367.5	55.15705	12.28932	-2.16614	Down	0	0
Mex3a	1362	108	26	2.855962	0.638662	-2.16085	Down	2.77E-13	9.84E-13
LOC680121	2109	30423	7313	517.022	115.7562	-2.15914	Down	0	0
Anxa6	2625	18402	4419	251.1139	56.28729	-2.15746	Down	0	0
LOC688272	2279	357.5	86	5.618368	1.26067	-2.15596	Down	1.67E-40	9.00E-40
Heatr5a	7753	5985.5	1439.5	27.54211	6.192953	-2.15294	Down	0	0
Actr1b	3193	11318	2726.5	126.8451	28.52236	-2.1529	Down	0	0
Pcdha12	5250	55.5	13.5	0.377788	0.08508	-2.15068	Down	1.68E-07	3.72E-07

Lrrk1	6445	2071	501	11.50603	2.593079	-2.14965	Down	########	########
Mvk	1723	1630	394.5	33.86379	7.637752	-2.14852	Down	########	########
Mapk10	2759	33	8	0.428622	0.096808	-2.1465	Down	6.88E-05	0.00013
Rnase4	1360	207.5	50	5.451352	1.231427	-2.14628	Down	4.39E-24	2.06E-23
Akap12	6255	7377.5	1787.5	42.19876	9.539772	-2.14517	Down	0	0
Gpt2	1769	967.5	235.5	19.63553	4.442365	-2.14407	Down	########	########
Hapln4	3687	2535	617.5	24.67278	5.585654	-2.14312	Down	########	########
Mpnd	1566	1600.5	388.5	36.5538	8.277284	-2.14279	Down	########	########
Dbc1	2420	643	156	9.531389	2.159646	-2.14189	Down	3.72E-71	2.38E-70
Mboat1	2853	1827	443.5	22.90497	5.197722	-2.13971	Down	########	########
Gpc1	3509	16455.5	4008.5	167.8653	38.1218	-2.13862	Down	0	0
Maged1	2695	9218.5	2253	122.6319	27.8607	-2.13803	Down	0	0
Slc29a1	2142	1105	269.5	18.42989	4.191594	-2.13648	Down	########	########
RGD1304563	5071	2244.5	546.5	15.83418	3.602434	-2.136	Down	########	########
Acsf2	3095	633	154	7.331108	1.668229	-2.13571	Down	6.66E-70	4.25E-69
Kif20a	2949	7563	1845	91.83884	20.9124	-2.13475	Down	0	0
Hmgcs1	3275	13603.5	3321.5	148.588	33.87501	-2.13302	Down	0	0
RGD1565297	813	33	8	1.434823	0.32853	-2.12678	Down	6.88E-05	0.00013
Hip1	7376	5408	1330	26.26191	6.014758	-2.12639	Down	0	0
Clip2	4847	4535	1115	33.4975	7.672848	-2.12622	Down	0	0
Tk1	2263	4686	1152.5	74.12963	16.98115	-2.12612	Down	0	0
RGD1311422	3215	2134.5	525	23.7965	5.452248	-2.12583	Down	########	########
Lat2	615	28	7	1.639412	0.375622	-2.12582	Down	0.000313	0.00056
LOC680835	6031	2286.5	561.5	13.56968	3.10921	-2.12577	Down	########	########
Mki67	8791	21552.5	5290	87.67299	20.09457	-2.12533	Down	0	0

Slc6a6	2489	3377	828	48.43994	11.11273	-2.12398	Down	0	0
LOC499229	7308	159	39	0.776887	0.178542	-2.12144	Down	1.34E-18	5.93E-18
LOC689955	2113	24482.5	6049	415.1502	95.59485	-2.11863	Down	0	0
Ide	4276	4312	1066	36.05875	8.318648	-2.11593	Down	0	0
Foxa1	2189	24	6	0.392324	0.09069	-2.11303	Down	0.000878	0.001499
Palm	2571	1930	478.5	26.96349	6.236695	-2.11215	Down	########	########
Fam173a	861	416	103.5	17.26102	3.99824	-2.11008	Down	8.56E-46	4.77E-45
Tbc1d2b	5706	1831	455	11.47737	2.661181	-2.10865	Down	########	########
Flot1	1718	3335	832.5	69.67708	16.15615	-2.1086	Down	0	0
Mapk3	1875	9628.5	2404.5	184.3062	42.82989	-2.10542	Down	0	0
Sbf1	6155	7580.5	1901	44.16507	10.30567	-2.09947	Down	0	0
Apoa5	1850	34	8.5	0.662597	0.154615	-2.09945	Down	4.19E-05	8.09E-05
Odz4	8565	304	76	1.270198	0.296462	-2.09913	Down	1.32E-33	6.75E-33
Msto1	1972	741	186	13.48344	3.148604	-2.0984	Down	3.16E-79	2.10E-78
Esyt1	3820	3442	863	32.24643	7.536026	-2.09726	Down	0	0
Mov10	3781	1554.5	390	14.73865	3.444474	-2.09725	Down	########	########
Evc	4315	898	226	7.453006	1.74406	-2.09537	Down	2.13E-95	1.49E-94
Acaa1a	1619	516	129.5	11.40679	2.67637	-2.09154	Down	6.65E-56	3.94E-55
Pnmal2	2575	24	6	0.334404	0.078494	-2.09095	Down	0.000878	0.001499
Lhx1	1938	210	53	3.884246	0.913055	-2.08886	Down	1.71E-23	7.96E-23
Tmem119	2167	7620.5	1925.5	126.0095	29.68078	-2.08593	Down	0	0
Fry	10792	1042	264	3.457848	0.815393	-2.08431	Down	########	########
Btbd6	2147	853	216	14.22277	3.358058	-2.0825	Down	3.48E-90	2.40E-89
Ankrd52	3387	2685.5	684	28.4154	6.71319	-2.0816	Down	########	########
Nut	3688	44	11	0.426604	0.100801	-2.08138	Down	5.38E-06	1.09E-05

Fam131b	4046	498.5	126	4.409422	1.042397	-2.08068	Down	2.03E-53	1.19E-52
Efhd2	2135	4846.5	1234	81.50959	19.29416	-2.07881	Down	0	0
Adprhl1	1545	50	12.5	1.15692	0.273906	-2.07853	Down	7.46E-07	1.60E-06
Emilin1	3836	5885	1493.5	54.93647	13.00661	-2.07852	Down	0	0
LOC100912447	7288	371	94	1.819521	0.430867	-2.07824	Down	3.64E-40	1.96E-39
Il17rc	2322	631	160.5	9.735153	2.306	-2.07781	Down	4.34E-67	2.72E-66
Olr1735	927	58.5	15	2.266516	0.537327	-2.0766	Down	2.55E-07	5.60E-07
Tle2	2431	118	30	1.740681	0.412754	-2.0763	Down	9.58E-14	3.71E-13
Odz3	10972	2478.5	631	8.082953	1.920158	-2.07366	Down	########	########
Pcdha6	5289	51	13	0.344286	0.081892	-2.07181	Down	1.17E-06	2.48E-06
Mgat4b	2395	2279.5	584	34.04668	8.105008	-2.07063	Down	########	########
Kremen1	2045	619.5	158	10.84122	2.58304	-2.06939	Down	1.94E-65	1.21E-64
Aspm	9727	4837.5	1233	17.76847	4.234074	-2.0692	Down	0	0
Timp3	1778	5927	1517.5	119.4912	28.49243	-2.06825	Down	0	0
Scd	5055	84875.5	21710	600.0514	143.42	-2.06484	Down	0	0
Afap1l2	3205	868.5	222	9.704067	2.32046	-2.06418	Down	7.08E-91	4.90E-90
Pde6d	1081	757.5	194.5	25.10631	6.006739	-2.0634	Down	2.48E-79	1.65E-78
LOC100909828	552	91	23.5	5.938523	1.422174	-2.06201	Down	5.79E-11	1.45E-10
Sod3	1729	13465	3466	279.63	66.98745	-2.06156	Down	0	0
Sfxn5	1083	93	24	3.080741	0.738212	-2.06117	Down	5.56E-11	1.39E-10
Pou5f1	1388	46.5	12	1.204367	0.288647	-2.0609	Down	5.13E-06	1.05E-05
Pcolce	1547	8574.5	2214	198.805	47.73057	-2.05837	Down	0	0
Med16	2942	1931.5	499	23.5666	5.658844	-2.05816	Down	########	########
Myo5a	5620	5517.5	1420	35.10882	8.435013	-2.05737	Down	0	0
Cacna1e	6973	89.5	23.5	0.465741	0.111937	-2.05684	Down	1.49E-10	3.67E-10

Fzd1	4399	6773	1749	55.20445	13.2708	-2.05653	Down	0	0
Aldh2	1889	3454.5	892.5	65.48259	15.756	-2.05521	Down	0	0
LOC684969	1887	33154.5	8564	628.9089	151.3961	-2.05452	Down	0	0
Khdrbs1	2655	6954	1794	93.59267	22.54137	-2.05382	Down	0	0
Kcnab2	1590	50.5	13	1.13561	0.27354	-2.05364	Down	1.88E-06	3.94E-06
Leprel4	1407	4346.5	1125	110.7825	26.70737	-2.05242	Down	0	0
LOC688655	2027	2576.5	665.5	45.41879	10.96392	-2.05053	Down	########	########
Alk	6375	125	32.5	0.701542	0.169572	-2.04863	Down	2.16E-14	8.85E-14
LOC100911721	2309	385.5	100	5.986249	1.447112	-2.04848	Down	1.16E-40	6.23E-40
LOC688932	2083	10016	2594.5	172.093	41.63775	-2.04722	Down	0	0
LOC100911378	1426	483	125	12.12978	2.934819	-2.04721	Down	9.07E-51	5.20E-50
Ptprz1	7871	7234	1874	32.84781	7.950868	-2.04661	Down	0	0
Zmiz2	4385	2225.5	579	18.22728	4.415614	-2.04541	Down	########	########
Afap1l1	3436	1047.5	273.5	10.92499	2.648761	-2.04424	Down	########	########
Fkbp4	2845	8646.5	2252	108.9191	26.41926	-2.04359	Down	0	0
Glt25d1	3039	4415	1152	52.0399	12.63202	-2.04253	Down	0	0
Vat1	2628	1640	426.5	22.35645	5.431555	-2.04125	Down	########	########
Capn1	3020	2675	697.5	31.74345	7.719819	-2.03982	Down	########	########
Atoh8	2375	1754.5	458.5	26.54413	6.456212	-2.03963	Down	########	########
Clec1a	3299	92	24	0.997374	0.242887	-2.03785	Down	8.89E-11	2.21E-10
Fam123b	3663	385.5	101	3.759545	0.918364	-2.03342	Down	2.80E-40	1.50E-39
LOC100911911	3422	3184	835.5	33.37545	8.161436	-2.03189	Down	0	0
C1ql3	768	90	24	4.208281	1.029275	-2.0316	Down	2.26E-10	5.54E-10
Gusb	2483	1654.5	433.5	23.83805	5.834166	-2.03067	Down	########	########
Hmx3	1362	79.5	21	2.097721	0.513453	-2.03052	Down	2.73E-09	6.46E-09

Itpr3	8806	8041.5	2117	32.71422	8.019075	-2.02841	Down	0	0
RGD1305592	1594	705.5	186.5	15.86796	3.894159	-2.02673	Down	5.90E-72	3.80E-71
Lphn1	5579	4898	1291.5	31.48063	7.73164	-2.02562	Down	0	0
Susd4	2121	197	52	3.331954	0.819384	-2.02376	Down	3.02E-21	1.38E-20
Ccnjl	2990	34	9	0.407283	0.100194	-2.02323	Down	0.000106	0.000197
Megf10	4054	6512.5	1719	57.48263	14.14976	-2.02235	Down	0	0
5-Sep	3666	2265.5	600.5	22.17536	5.461847	-2.0215	Down	########	########
Coro7	3499	991	262.5	10.15482	2.502283	-2.02085	Down	########	2.32E-99
Cad	7126	4616	1218.5	23.17427	5.712024	-2.02045	Down	0	0
Foxp4	3401	3298	877	34.87252	8.601892	-2.01936	Down	0	0
Adamtsl4	4180	815	216	6.982773	1.723098	-2.0188	Down	1.56E-82	1.05E-81
Col6a3	10811	2081	551.5	6.881147	1.700035	-2.01708	Down	########	########
Olr1273	936	26	7	0.994591	0.245841	-2.01638	Down	0.000821	0.001408
Pop5	1439	646.5	172	16.11042	3.98251	-2.01624	Down	1.73E-65	1.08E-64
Slc22a25	2595	20.5	5.5	0.283896	0.070242	-2.01496	Down	0.002494	0.004103
Pkd1	13813	3362	892	8.709338	2.157123	-2.01345	Down	0	0
Calm3	599	2899	772.5	173.5996	43.02498	-2.01252	Down	########	########
Pip4k2b	1987	1811	482	32.63073	8.090711	-2.01189	Down	########	########
Vom1r85	909	69.5	18.5	2.73811	0.678995	-2.01171	Down	2.08E-08	4.77E-08
Cxxc5	2081	4578.5	1224	78.9585	19.58508	-2.01134	Down	0	0
Cdkn2d	1234	953.5	253.5	27.65424	6.85973	-2.01128	Down	4.83E-96	3.38E-95
Exd2	3197	1521.5	404.5	16.99489	4.216802	-2.01088	Down	########	########
Dync1h1	14279	20878	5548	52.23208	12.97453	-2.00925	Down	0	0
Vash1	3022	845	225.5	10.01525	2.495326	-2.0049	Down	4.13E-85	2.81E-84
Atp5d	811	4838	1296	213.9632	53.32418	-2.0045	Down	0	0

LOC100360260	1293	976.5	260	26.95926	6.721865	-2.00385	Down	5.58E-98	3.94E-97
Lrp4	7784	2192.5	585.5	10.0799	2.514482	-2.00315	Down	########	########
Bub3	1364	2526.5	673.5	66.13926	16.51141	-2.00204	Down	########	########
Pdgfra	6532	4541.5	1213.5	24.86367	6.208244	-2.00178	Down	0	0
Flot2	2562	1537	411	21.47923	5.366503	-2.00089	Down	########	########
L3mbtl3	2532	318	85	4.490026	1.121872	-2.00082	Down	5.94E-33	3.03E-32
Pcdha8	5247	37.5	10	0.254484	0.06363	-1.99979	Down	6.17E-05	0.000117
Camk1	1439	679	183	16.91281	4.228968	-1.99974	Down	6.48E-68	4.09E-67
Sox10	3029	4913.5	1319.5	58.17545	14.55281	-1.99911	Down	0	0
Hspa2	2501	370.5	100	5.309094	1.329181	-1.99793	Down	1.14E-37	6.05E-37
LOC685179	4496	5019.5	1351	40.00271	10.02337	-1.99673	Down	0	0
Csrp1	1720	11449.5	3075	238.1349	59.7148	-1.99562	Down	0	0
Chrna4	2149	533.5	145.5	8.949142	2.246035	-1.99437	Down	3.51E-53	2.04E-52
Daam2	3299	355.5	96	3.862517	0.96991	-1.99362	Down	3.47E-36	1.81E-35
Txndc5	2631	9745	2624.5	132.467	33.29458	-1.99227	Down	0	0
Myo1c	3810	11775.5	3174.5	110.7414	27.84454	-1.99173	Down	0	0
Zfp161	1350	203	55	5.390932	1.355537	-1.99167	Down	2.57E-21	1.17E-20
LOC100911345	3988	81.5	22	0.733362	0.184631	-1.98988	Down	2.57E-09	6.09E-09
Kcnab1	3522	665.5	179.5	6.735782	1.696588	-1.98921	Down	1.23E-66	7.72E-66
Klhl34	2329	67	18	1.029648	0.25958	-1.9879	Down	5.29E-08	1.19E-07
RGD1561333	840	1276	346.5	54.5742	13.77688	-1.98597	Down	########	########
LOC690422	1647	894.5	241.5	19.39432	4.90344	-1.98377	Down	7.77E-89	5.34E-88
Fstl1	2283	27609.5	7477	432.5712	109.3925	-1.98342	Down	0	0
RGD1307929	7456	10848.5	2938	52.04428	13.16448	-1.98309	Down	0	0
LOC100909788	1333	7619	2062	204.2618	51.676	-1.98285	Down	0	0

Slc44a2	3453	2144	581	22.2027	5.617126	-1.98283	Down	########	########
Ung	1855	1181.5	321.5	22.82918	5.777957	-1.98225	Down	########	########
Fam108c1	2208	1345	365	21.8038	5.523182	-1.98101	Down	########	########
Sh3pxd2a	3373	4125.5	1122	43.78543	11.09197	-1.98094	Down	0	0
Gucy1a3	2666	53.5	14.5	0.723781	0.183443	-1.98022	Down	1.11E-06	2.35E-06
LOC100361457	1919	45824.5	12471	854.7132	216.6636	-1.97998	Down	0	0
Rgl2	2894	1494.5	407.5	18.47507	4.688865	-1.97827	Down	########	########
LOC684035	3163	1775	484.5	20.15521	5.116522	-1.97792	Down	########	########
Cdk5	1178	1208.5	329	36.69535	9.32068	-1.97709	Down	########	########
Nov	1475	413	112.5	10.01231	2.545536	-1.97573	Down	1.03E-41	5.58E-41
G6pd	2408	5638.5	1541.5	83.91336	21.36143	-1.97389	Down	0	0
Chd1l	2999	189	52	2.257964	0.575897	-1.97114	Down	1.18E-19	5.26E-19
Srm	1265	1330	363.5	37.70349	9.619018	-1.97074	Down	########	########
Slc48a1	1572	1975	542	45.02634	11.49634	-1.96959	Down	########	########
Crocc	6308	417	114.5	2.374505	0.606522	-1.969	Down	9.14E-42	4.98E-41
Clmp	3925	1454	397.5	13.2556	3.385913	-1.96899	Down	########	########
Slc29a2	1678	148	41	3.171126	0.810405	-1.96828	Down	1.33E-15	5.63E-15
Timm22	1145	800	219.5	24.99838	6.388956	-1.96818	Down	1.89E-78	1.25E-77
Fgd1	3684	1034	283.5	10.05367	2.57256	-1.96645	Down	########	########
LOC688318	890	1122.5	309.5	45.17137	11.56434	-1.96573	Down	########	########
Preb	2024	1098.5	302	19.42726	4.978961	-1.96417	Down	########	########
Pcdhgc3	4614	7541	2076	58.56781	15.01409	-1.96379	Down	0	0
Ccdc74a	1269	58	16	1.630964	0.418116	-1.96375	Down	6.11E-07	1.31E-06
Epn1	2047	2314.5	638.5	40.58499	10.41423	-1.96239	Down	########	########
Sort1	6690	501.5	138	2.67902	0.68762	-1.96202	Down	1.83E-49	1.04E-48

Adamtsl3	7268	5852	1610	28.77454	7.390859	-1.96098	Down	0	0
Tmem104	1976	683	189	12.398	3.18472	-1.96087	Down	1.71E-66	1.07E-65
Wdr34	1882	493.5	136	9.393629	2.414084	-1.96021	Down	1.26E-48	7.12E-48
Cerk	4319	2985	824	24.7563	6.368241	-1.95883	Down	########	########
Ehd2	2057	4484	1239	78.11945	20.10693	-1.95799	Down	0	0
Recql4	3860	799.5	220.5	7.412693	1.909182	-1.95704	Down	6.90E-78	4.56E-77
Tln2	8647	4908	1355.5	20.30252	5.231673	-1.95631	Down	0	0
Gpsm3	1259	122	34	3.487518	0.9002	-1.95388	Down	4.91E-13	1.65E-12
Mmp16	3519	871.5	241	8.847007	2.284718	-1.95317	Down	2.57E-84	1.74E-83
Hsp90ab1	2338	71767	19863	1098.344	283.6613	-1.95309	Down	0	0
Kifc1	2316	3331	923	51.56571	13.32786	-1.95197	Down	0	0
Immt	1938	3787.5	1048.5	69.83136	18.06647	-1.95056	Down	0	0
Mypop	1866	395.5	110	7.616702	1.973928	-1.9481	Down	6.50E-39	3.47E-38
LOC686298	1826	262.5	73	5.149894	1.335234	-1.94745	Down	2.49E-26	1.20E-25
Antxr1	3384	469	130.5	4.964403	1.287922	-1.94657	Down	4.09E-46	2.28E-45
Spon2	1773	1950.5	541	39.33147	10.20466	-1.94646	Down	########	########
Spag11b	336	22	6	2.33783	0.606908	-1.94562	Down	0.002316	0.003824
Usp1	3563	4088	1136	40.96778	10.63975	-1.94503	Down	0	0
RGD1309823	2079	811.5	228	13.9923	3.634628	-1.94475	Down	2.48E-77	1.63E-76
Tmem50b	2289	500	138	7.777516	2.020306	-1.94474	Down	2.87E-49	1.63E-48
Ahcy	2029	4696.5	1309	82.90202	21.53491	-1.94473	Down	0	0
LOC100909925	624	46	13	2.647056	0.688347	-1.94318	Down	1.21E-05	2.43E-05
Actg1	1909	51482.5	14360.5	964.8233	250.9874	-1.94265	Down	0	0
Map2k2	1376	2687.5	751	70.0402	18.22463	-1.94229	Down	########	########
Mknk2	3458	4547	1272	47.15215	12.27329	-1.9418	Down	0	0

Nt5dc2	1822	1104	308.5	21.70848	5.652295	-1.94135	Down	########	########
B4galt2	2019	1263	354	22.43132	5.845384	-1.94014	Down	########	########
Cyp51	3046	27848	7779.5	327.1598	85.25931	-1.94007	Down	0	0
Clip3	2123	1469.5	411.5	24.8364	6.47368	-1.9398	Down	########	########
A3galt2	1936	682.5	191	12.62758	3.291519	-1.93975	Down	1.41E-65	8.80E-65
Kif3c	3419	4935.5	1380.5	51.73533	13.49188	-1.93906	Down	0	0
Ramp3	1234	64	18	1.872586	0.488462	-1.93871	Down	2.11E-07	4.63E-07
Actb	1293	184825	51866.5	5130.646	1338.845	-1.93815	Down	0	0
Gnai2	2218	27003.5	7571	436.5213	113.9142	-1.9381	Down	0	0
Adi1	1333	444	124.5	11.93084	3.116585	-1.93666	Down	2.08E-43	1.14E-42
Nacc1	2044	3677.5	1033	64.59257	16.87616	-1.93638	Down	0	0
Maged2	2112	1350.5	378	22.85779	5.976333	-1.93535	Down	########	########
Abi3	1708	35.5	10	0.743235	0.194419	-1.93465	Down	0.000156	0.000285
Prrx2	1230	53.5	15	1.558989	0.407888	-1.93437	Down	2.61E-06	5.44E-06
Tbx2	1234	132.5	37.5	3.862417	1.01058	-1.93432	Down	6.51E-14	2.57E-13
LOC100360453	1046	1828	511.5	62.31146	16.31128	-1.93363	Down	########	########
Tep1	8215	1980	556	8.617305	2.257464	-1.93253	Down	########	########
Uhrf1	2536	3899	1094.5	55.07885	14.43569	-1.93186	Down	0	0
Taldo1	1225	5702	1604.5	166.5979	43.6715	-1.93161	Down	0	0
Dlg5	7246	3189.5	896.5	15.76465	4.135016	-1.93073	Down	########	########
Col6a2	3626	39404.5	11116	389.7279	102.3737	-1.92862	Down	0	0
Sepn1	3203	7995	2257.5	89.49317	23.50811	-1.92862	Down	0	0
Lama5	11307	8267.5	2333	26.20473	6.886631	-1.92796	Down	0	0
Rab3a	1404	400	113	10.21262	2.685192	-1.92726	Down	8.29E-39	4.42E-38
Nefl	2032	409	115.5	7.206101	1.895732	-1.92646	Down	7.73E-40	4.15E-39

Pkn1	3087	1579.5	445	18.32201	4.820095	-1.92644	Down	########	########
LOC100911010	336	25	7	2.65391	0.698237	-1.92633	Down	0.001319	0.002222
Pcdhga3	4712	609.5	172	4.631321	1.218513	-1.9263	Down	3.17E-58	1.91E-57
Tpm4	895	14849	4190	593.9695	156.3055	-1.92602	Down	0	0
Llgl1	3111	2496	706	28.77244	7.57351	-1.92565	Down	########	########
Ptprb	6539	70	20	0.383761	0.101015	-1.92564	Down	7.25E-08	1.63E-07
Nnt	4149	4809	1357	41.45415	10.9169	-1.92495	Down	0	0
Pycard	582	178.5	50	10.94963	2.889933	-1.92177	Down	3.18E-18	1.40E-17
Hdgf	1847	10200.5	2887	197.6288	52.16125	-1.92174	Down	0	0
Prrc2a	6664	27423	7795	147.7504	38.99845	-1.92167	Down	0	0
Pls1	1961	320.5	90.5	5.823303	1.537816	-1.92096	Down	1.64E-31	8.25E-31
Eya2	1596	382.5	109	8.611978	2.274538	-1.92077	Down	9.48E-37	4.98E-36
Fam176b	1102	825.5	234	26.81926	7.086129	-1.9202	Down	6.70E-78	4.43E-77
RGD1560015	1124	325	92.5	10.37464	2.741575	-1.91998	Down	9.12E-32	4.59E-31
Pcdhgb8	4714	687	195	5.219896	1.379556	-1.91982	Down	4.08E-65	2.53E-64
Pcbp4	2022	6146.5	1748.5	109.2086	28.8813	-1.91888	Down	0	0
LOC690976	854	6622.5	1880	277.9916	73.51898	-1.91885	Down	0	0
Sv2a	3554	1275	362	12.83887	3.400429	-1.91673	Down	########	########
LOC100911642	3167	591	168.5	6.682829	1.77279	-1.91444	Down	3.60E-56	2.14E-55
Thop1	2408	2503	714	37.26639	9.888365	-1.91407	Down	########	########
Sv2c	2622	94	27	1.287221	0.341741	-1.91329	Down	4.56E-10	1.11E-09
Spr	1219	1022.5	291.5	30.03787	7.983434	-1.9117	Down	1.13E-95	7.94E-95
Loxl3	2840	12666	3609	159.7638	42.47117	-1.91139	Down	0	0
Lypd2	545	23	6.5	1.524849	0.405623	-1.91046	Down	0.001431	0.002404
Mxd3	989	476	137	17.29068	4.603936	-1.90906	Down	5.66E-45	3.14E-44

Add1	3826	12905.5	3692.5	120.8599	32.20268	-1.90808	Down	0	0
Tubg1	1685	3810.5	1094	81.22889	21.65957	-1.90699	Down	0	0
Mras	3626	845	242	8.354551	2.227995	-1.90682	Down	6.36E-79	4.23E-78
Coro1c	3417	14141	4048	148.2818	39.54665	-1.90671	Down	0	0
Egflam	3714	395	112.5	3.797828	1.013373	-1.90601	Down	3.39E-38	1.80E-37
Tacr1	3408	150.5	43	1.577945	0.421254	-1.90528	Down	2.82E-15	1.19E-14
LOC100911769	4209	380.5	109.5	3.246643	0.867832	-1.90346	Down	2.30E-36	1.21E-35
Tubgcp2	2852	2400.5	689	30.13538	8.068588	-1.90107	Down	########	########
Cbx6	3095	3782.5	1086.5	43.80439	11.73309	-1.90049	Down	0	0
Chrdl1	4141	1875	538	16.17581	4.334147	-1.90002	Down	########	########
Lonrf3	2711	295.5	84.5	3.903241	1.046122	-1.89962	Down	8.35E-29	4.10E-28
LOC100360791	818	10906	3143	477.4536	127.974	-1.89951	Down	0	0
Pabpc1	2190	48222.5	13909.5	790.1692	211.8442	-1.89916	Down	0	0
Znf469-ps1	11157	2003	577.5	6.438606	1.726247	-1.89911	Down	########	########
Ptms	1154	14267	4104.5	442.8905	118.7485	-1.89904	Down	0	0
Dgkz	3560	3291	947	33.15332	8.894919	-1.8981	Down	########	########
Tmtc4	2586	2499.5	716.5	34.52233	9.266977	-1.89736	Down	########	########
Bola1	746	263	76	12.68676	3.406163	-1.8971	Down	1.86E-25	8.84E-25
Klhdc8a	3199	739	212.5	8.271831	2.221022	-1.89698	Down	4.04E-69	2.56E-68
Dnmt1	5917	6981	2010	42.21616	11.34467	-1.89578	Down	0	0
Pofut1	1188	656	189	19.7813	5.317599	-1.89529	Down	2.87E-61	1.75E-60
Foxm1	3393	2324.5	671.5	24.55054	6.607897	-1.89349	Down	########	########
Serpinb13	1608	21	6	0.466962	0.125697	-1.89335	Down	0.003719	0.006049
Xylt2	3375	1374	397.5	14.58797	3.928891	-1.89259	Down	########	########
Slc25a10	1946	2445	707.5	45.08884	12.15243	-1.89153	Down	########	########

Slc25a39	1473	3655	1059	88.92045	23.9671	-1.89146	Down	0	0
Cpox	2810	567	164	7.212971	1.946312	-1.88985	Down	5.76E-53	3.35E-52
Pacsin3	1778	1485	431	29.92612	8.075993	-1.88969	Down	########	########
Bcr	4692	1708	494.5	13.04844	3.524559	-1.88836	Down	########	########
LOC684352	1582	854	248	19.35467	5.230423	-1.88768	Down	1.51E-78	9.99E-78
Pcdhgb7	4748	1228.5	356.5	9.268778	2.505023	-1.88756	Down	########	########
Fads2	1706	4999	1451.5	105.0799	28.42386	-1.88631	Down	0	0
Sap30l	1575	1013	295	23.06147	6.24426	-1.88488	Down	1.50E-92	1.04E-91
Rab6b	4577	2938	854	22.98648	6.230661	-1.88333	Down	########	########
Katnb1	1968	734	213	13.35676	3.62403	-1.8819	Down	8.18E-68	5.16E-67
Clybl	1250	113.5	33	3.245677	0.880692	-1.88181	Down	1.22E-11	3.19E-11
Lmna	3091	33359.5	9712.5	386.3062	104.8711	-1.88113	Down	0	0
Clic5	5847	618	180	3.780842	1.026582	-1.88086	Down	3.84E-57	2.29E-56
Prkar2a	1541	2803	816.5	65.17048	17.70375	-1.88016	Down	########	########
Fam151a	1912	27.5	8	0.514215	0.139694	-1.8801	Down	0.001193	0.002014
Dhcr7	2737	2548.5	745.5	33.40292	9.081848	-1.87892	Down	########	########
Zdhhc8	3384	1432.5	420.5	15.21038	4.136585	-1.87854	Down	########	########
Fndc5	2178	138	40.5	2.276735	0.619796	-1.8771	Down	5.17E-14	2.05E-13
Lmo4	1650	9149.5	2672.5	198.3979	54.01842	-1.87687	Down	0	0
Lbp	1881	318.5	93	6.079568	1.655488	-1.87671	Down	4.53E-30	2.25E-29
Lhx6	3407	373	109.5	3.92514	1.069212	-1.8762	Down	5.04E-35	2.61E-34
LOC100912402	1601	7183.5	2102	160.747	43.81879	-1.87517	Down	0	0
LOC690617	642	105	31	5.846146	1.593911	-1.87491	Down	8.54E-11	2.13E-10
Wif1	2182	2629	767.5	43.02305	11.73012	-1.87489	Down	########	########
Des	2169	68.5	20	1.132067	0.308684	-1.87475	Down	1.78E-07	3.92E-07

Cndp1	2675	295	86	3.938257	1.074716	-1.8736	Down	4.19E-28	2.05E-27
Nras	1326	2417.5	704	65.00844	17.75356	-1.87252	Down	########	########
Mgat5	2623	523.5	154	7.151369	1.955383	-1.87077	Down	4.80E-48	2.71E-47
Slc4a2	4053	7150	2102	63.17039	17.29647	-1.86877	Down	0	0
Lfng	1860	1986	584.5	38.28002	10.48422	-1.86837	Down	########	########
Cdh24	2728	993	293.5	13.08385	3.586576	-1.86711	Down	1.92E-89	1.32E-88
Cadm4	1167	2784.5	819	85.52762	23.4455	-1.86708	Down	########	########
Aco1	3564	2095	614.5	21.02229	5.764211	-1.86672	Down	########	########
LOC100910384	2195	40.5	12	0.659584	0.180885	-1.86649	Down	8.17E-05	0.000153
Sec24d	3883	6383	1875.5	58.88291	16.15061	-1.86626	Down	0	0
Myh10	7648	6392.5	1878.5	29.90593	8.209111	-1.86513	Down	0	0
LOC100909580	2346	1696.5	500.5	25.95948	7.127984	-1.8647	Down	########	########
Furin	4259	10338.5	3052	87.09466	23.91527	-1.86465	Down	0	0
Fkbp10	2589	11848.5	3489	163.9673	45.03356	-1.86434	Down	0	0
Polr3h	1269	1086.5	320.5	30.63462	8.418379	-1.86355	Down	8.46E-98	5.97E-97
Vmac	516	57.5	17	3.994696	1.098209	-1.86293	Down	2.18E-06	4.56E-06
Tlr5	4255	115	34	0.968732	0.266358	-1.86273	Down	1.12E-11	2.93E-11
Ppl	6281	6881.5	2028.5	39.2376	10.78978	-1.86257	Down	0	0
LOC100909973	939	457	135.5	17.47878	4.811568	-1.86103	Down	4.81E-42	2.62E-41
Ppox	1793	392	115.5	7.815861	2.152943	-1.86009	Down	1.43E-36	7.50E-36
Pcdhga9	4732	627	185.5	4.7502	1.308517	-1.86006	Down	3.94E-57	2.35E-56
Atp9a	3286	4764	1408.5	51.89046	14.29999	-1.85945	Down	0	0
RGD1309310	4854	948.5	281.5	6.999991	1.929262	-1.8593	Down	4.90E-85	3.33E-84
Slc26a1	3414	51	15	0.535051	0.147482	-1.85914	Down	6.46E-06	1.31E-05
LOC100361389	3105	196	58	2.263582	0.62481	-1.85712	Down	6.93E-19	3.07E-18

LOC100359816	6955	729	216.5	3.760244	1.039223	-1.85532	Down	7.82E-66	4.87E-65
Ecel1	7798	22	6.5	0.101688	0.028118	-1.85459	Down	0.002316	0.003824
Epb41l1	5813	2092.5	622.5	12.89116	3.565945	-1.85403	Down	########	########
Tpcn1	4735	13843.5	4107.5	104.698	28.96787	-1.85371	Down	0	0
Aspscr1	1708	1541	458	32.33757	8.969005	-1.85019	Down	########	########
RGD1560175	8065	2214	660	9.841683	2.731878	-1.84901	Down	########	########
Astn1	7201	53.5	16	0.26629	0.073933	-1.84872	Down	5.85E-06	1.19E-05
Dapk3	1514	2165	649	51.27712	14.24699	-1.84766	Down	########	########
Cybb	1713	52.5	15.5	1.097186	0.304989	-1.84698	Down	4.12E-06	8.46E-06
Rhobtb2	2903	406.5	122	5.021602	1.396586	-1.84624	Down	7.78E-37	4.09E-36
Tubb4b	1578	25368.5	7582	576.3495	160.3046	-1.84613	Down	0	0
Fzd2	1912	2044	614.5	38.43867	10.69375	-1.84579	Down	########	########
Tsc2	5654	3169	946.5	20.08874	5.590585	-1.84532	Down	########	########
Ppp2r4	2479	8657	2581.5	124.7672	34.75474	-1.84396	Down	0	0
Vwa5a	5941	994	298	5.98947	1.66968	-1.84286	Down	6.17E-88	4.23E-87
Parp1	3571	4094.5	1221.5	41.0093	11.43507	-1.84249	Down	0	0
Map4	5535	18612	5565.5	120.3842	33.57054	-1.84238	Down	0	0
RGD1306271	8101	1019.5	304.5	4.501184	1.255664	-1.84185	Down	1.32E-90	9.12E-90
Cdr2l	2015	883.5	264.5	15.74265	4.394374	-1.84095	Down	1.46E-78	9.70E-78
Sema4c	4189	2050.5	614	17.51145	4.893865	-1.83925	Down	########	########
Stk25	2021	2684.5	804.5	47.56601	13.2972	-1.83881	Down	########	########
Htra1	2012	28885	8674	514.2996	143.8196	-1.83835	Down	0	0
Itga4	3736	2219.5	665	21.25328	5.943745	-1.83824	Down	########	########
Anxa7	1885	3671.5	1100	69.67548	19.49543	-1.83751	Down	0	0
Ifi35	1101	275	82	8.925704	2.498019	-1.83718	Down	1.12E-25	5.36E-25

Bmp7	2445	840	253	12.33186	3.452541	-1.83666	Down	3.37E-74	2.19E-73
Pdzrn3	4109	1454.5	436.5	12.68598	3.552055	-1.83651	Down	########	########
Hyou1	4498	8151.5	2449	64.83841	18.15751	-1.83628	Down	0	0
Kank2	4792	2886.5	868	21.56467	6.042081	-1.83555	Down	########	########
Lrp5	5101	3921	1180	27.55855	7.722572	-1.83535	Down	0	0
Mpdz	7516	2432.5	730	11.57927	3.245366	-1.83509	Down	########	########
Prr12	7017	2647	798	13.53378	3.795721	-1.83412	Down	########	########
Ppargc1b	3163	37	11	0.41804	0.117248	-1.83408	Down	0.000142	0.00026
Gsr	1850	2058.5	617.5	39.80603	11.16611	-1.83386	Down	########	########
Col8a1	2432	2956	888.5	43.52706	12.21021	-1.83382	Down	########	########
Clcn2	3217	212.5	64	2.360019	0.662249	-1.83335	Down	7.07E-20	3.17E-19
Mast3	5371	1083	326.5	7.229048	2.028811	-1.83317	Down	3.34E-95	2.33E-94
Galnt2	3842	4384	1321.5	40.86648	11.47359	-1.8326	Down	0	0
Pck2	2203	696.5	208.5	11.29174	3.170268	-1.83259	Down	2.49E-62	1.53E-61
Igfbp6	869	20	6	0.827509	0.23259	-1.83098	Down	0.005925	0.009469
Plxnb2	6769	15228	4595	80.6316	22.66729	-1.83073	Down	0	0
LOC100910915	628	176.5	53.5	10.06741	2.832077	-1.82976	Down	8.52E-17	3.68E-16
Pcdhga12	4738	593	178.5	4.480747	1.261143	-1.82901	Down	4.25E-53	2.48E-52
Haghl	1047	52	15.5	1.778843	0.500712	-1.82889	Down	4.12E-06	8.46E-06
Creb3l1	2672	3251	985.5	43.7713	12.32281	-1.82865	Down	########	########
Mug1	4620	26	8	0.202495	0.057033	-1.828	Down	0.001878	0.003127
Dnajb14	1729	374.5	112.5	7.741167	2.180432	-1.82794	Down	3.45E-34	1.78E-33
Rps4x	915	6719	2032.5	262.8614	74.14529	-1.82588	Down	0	0
LOC689803	1448	688.5	208	16.92028	4.773519	-1.82563	Down	7.87E-61	4.79E-60
Gna11	1080	1352	410.5	44.83513	12.65383	-1.82506	Down	########	########

Mesdc1	2046	1703.5	517	29.88155	8.433813	-1.825	Down	########	########
Unc5b	2838	10429	3165.5	131.8468	37.2407	-1.82391	Down	0	0
RGD1561609	4993	172	52	1.233128	0.348431	-1.82338	Down	2.23E-16	9.55E-16
Tmem44	1343	23	7	0.613245	0.173349	-1.82278	Down	0.003327	0.005425
LOC100910298	1025	89	27	3.113745	0.880336	-1.82253	Down	4.17E-09	9.80E-09
Eml1	2618	1318	400.5	18.03522	5.099758	-1.82232	Down	########	########
RGD1307222	5370	4723.5	1434	31.50969	8.910053	-1.82229	Down	0	0
Stub1	1283	3037.5	925	84.96904	24.06165	-1.8202	Down	########	########
Arrb1	1353	1741.5	528.5	46.10231	13.05963	-1.81972	Down	########	########
Tacc3	2042	3091.5	938	54.07304	15.32137	-1.81936	Down	########	########
LOC100910790	1513	767	232.5	18.15208	5.143876	-1.81921	Down	1.42E-67	8.92E-67
Mapk8ip1	3003	1790.5	546	21.37167	6.058041	-1.81878	Down	########	########
Slc22a17	2365	1021	312	15.45631	4.383968	-1.81789	Down	2.62E-88	1.80E-87
Ube2c	763	2600	793	122.1552	34.66532	-1.81715	Down	########	########
LOC100911560	1613	70	21.5	1.549344	0.439719	-1.81701	Down	1.62E-07	3.57E-07
Cttn	2951	13454.5	4101	163.2934	46.36984	-1.81621	Down	0	0
LOC100362372	4238	21	6.5	0.177583	0.050463	-1.81518	Down	0.003719	0.006049
Trim28	3001	10738	3278	128.1211	36.41242	-1.81501	Down	0	0
Itgb4	5896	215	66	1.308254	0.371901	-1.81465	Down	8.98E-20	4.02E-19
Papss1	2375	6653	2027.5	100.2404	28.49971	-1.81445	Down	0	0
Kif22	2098	3719	1134.5	63.39473	18.03836	-1.81329	Down	0	0
Cenpb	2681	5268.5	1612	70.35927	20.02978	-1.81259	Down	0	0
Bcas1	2675	377	115	5.046096	1.437676	-1.81143	Down	9.52E-34	4.89E-33
Pfkm	2757	1725.5	527.5	22.42187	6.391039	-1.81078	Down	########	########
Ccny	1456	2199.5	671.5	54.04732	15.40803	-1.81054	Down	########	########

Ncor2	8576	22964.5	7044	96.16002	27.4194	-1.81024	Down	0	0
LOC690955	2490	41	12.5	0.589432	0.168147	-1.80961	Down	5.21E-05	9.94E-05
Pdk2	2207	744.5	229.5	12.10669	3.454881	-1.8091	Down	2.71E-64	1.68E-63
Ogdh	3266	4322	1324	47.43559	13.54098	-1.80864	Down	0	0
LOC100909685	768	72	22	3.358411	0.958737	-1.80857	Down	1.46E-07	3.23E-07
MGC93975	1673	4755	1458	101.7562	29.06125	-1.80795	Down	0	0
LOC311578	7233	2601.5	800	12.87911	3.682268	-1.80637	Down	########	########
Pcdha2	5244	35.5	11	0.24306	0.069518	-1.80584	Down	0.000346	0.000619
Mn1	3915	261	80	2.396241	0.685452	-1.80565	Down	1.02E-23	4.74E-23
Ano4	3975	428	131	3.843942	1.100069	-1.80499	Down	6.02E-38	3.19E-37
Col6a1	4044	39276.5	12062	347.8652	99.60322	-1.80426	Down	0	0
Dclk2	3982	277	84.5	2.49135	0.714022	-1.80289	Down	2.22E-25	1.06E-24
Apba3	1939	1413	436	26.15337	7.496157	-1.80277	Down	########	########
Prkra	1589	695	214	15.62676	4.483938	-1.80118	Down	3.80E-60	2.30E-59
Kcng1	2745	479	148	6.25772	1.795828	-1.80099	Down	8.15E-42	4.44E-41
Flnc	9049	2851.5	878	11.27831	3.237045	-1.8008	Down	########	########
Arntl2	2749	594.5	183	7.7361	2.220912	-1.80045	Down	1.26E-51	7.30E-51
Prkcdbp	1049	10681.5	3307.5	366.0766	105.1276	-1.8	Down	0	0
Spn	3866	202	62	1.865831	0.535898	-1.79979	Down	1.14E-18	5.03E-18
Mybbp1a	3834	7059	2178	65.92686	18.95448	-1.79833	Down	0	0
LOC100910454	1348	526	161.5	13.9195	4.002982	-1.79796	Down	2.97E-46	1.66E-45
Mcm5	2528	5498	1698.5	77.96736	22.42625	-1.79768	Down	0	0
Lztr1	2779	2005	618.5	25.82276	7.434039	-1.79642	Down	########	########
Lpcat3	1935	3712	1146.5	68.70598	19.78306	-1.79617	Down	0	0
Zcchc24	4423	2392.5	738.5	19.36548	5.576466	-1.79606	Down	########	########

Tacc1	7653	4479	1383	20.94694	6.034857	-1.79535	Down	0	0
LOC304558	10561	985.5	304.5	3.345276	0.964287	-1.79459	Down	2.85E-84	1.93E-83
RT1-T24-1	1059	648.5	201	21.90906	6.324975	-1.7924	Down	1.02E-55	6.06E-55
Olfm2	2024	559	174	9.896419	2.857314	-1.79225	Down	4.22E-48	2.38E-47
Ldlr	3037	7341	2275	86.61338	25.0244	-1.79125	Down	0	0
LOC100909949	1411	867	268.5	22.02915	6.367548	-1.7906	Down	2.95E-74	1.93E-73
Dnpep	1674	3167.5	981.5	67.70761	19.57886	-1.79002	Down	########	########
Tbcb	1107	731	226.5	23.64269	6.838088	-1.78973	Down	7.14E-63	4.39E-62
Zfp385a	2293	3100	965	48.59663	14.06876	-1.78836	Down	########	########
LOC687022	3134	359.5	111.5	4.102943	1.1894	-1.78643	Down	1.01E-31	5.08E-31
Wasf1	2579	1117.5	347.5	15.53327	4.504369	-1.78596	Down	1.39E-94	9.68E-94
LOC688708	2814	204.5	63.5	2.595867	0.752918	-1.78565	Down	1.03E-18	4.56E-18
Ppp1r9b	4505	4644.5	1446	36.95945	10.72098	-1.78551	Down	0	0
Igsf8	2206	1486	464	24.14632	7.004478	-1.78545	Down	########	########
Hsd17b8	970	188	58.5	6.945948	2.014926	-1.78544	Down	2.22E-17	9.64E-17
LOC100360117	862	7270	2264	301.8459	87.58021	-1.78514	Down	0	0
Tpm3	1770	16496.5	5135	333.9062	96.95508	-1.78405	Down	0	0
LOC691317	1029	27	8.5	0.941705	0.273603	-1.78319	Down	0.001193	0.002014
Pcdhga7	4761	625.5	195	4.705837	1.367828	-1.78256	Down	1.97E-53	1.15E-52
Ubqln4	3258	3590	1122	39.49953	11.48956	-1.78151	Down	########	########
Nlrp1a	3690	622	194	6.018544	1.753345	-1.77931	Down	3.33E-53	1.94E-52
Chl1	3511	2601	810.5	26.4617	7.71067	-1.77898	Down	########	########
Rpl8	845	9910.5	3096.5	419.9004	122.373	-1.77876	Down	0	0
RGD1564125	1173	79.5	24.5	2.417629	0.704627	-1.77866	Down	3.22E-08	7.33E-08
Adam23	2876	137	42.5	1.705553	0.497284	-1.7781	Down	3.84E-13	1.32E-12

Arap3-ps1	5350	1253.5	392	8.386175	2.450492	-1.77494	Down	########	########
LOC100362640	955	14733	4616.5	551.9567	161.2963	-1.77484	Down	0	0
Twsg1	4181	2862	895	24.44899	7.148624	-1.77404	Down	########	########
Col3a1	4792	16093	5042	120.0498	35.1226	-1.77316	Down	0	0
Ppm1j	1711	98	31	2.055569	0.601748	-1.77231	Down	1.78E-09	4.24E-09
Actn4	3885	52146	16399	481.2702	140.9228	-1.77194	Down	0	0
Yipf2	1977	935	294	16.95548	4.964958	-1.7719	Down	2.50E-78	1.66E-77
LOC100361376	4792	2780	873	20.76839	6.083491	-1.77142	Down	########	########
Guca2a	567	469.5	149	29.8945	8.757733	-1.77125	Down	1.17E-39	6.26E-39
Il17d	1193	55.5	17.5	1.6755	0.49088	-1.77115	Down	5.27E-06	1.07E-05
Abcd1	3411	824	259	8.652275	2.535352	-1.77089	Down	2.84E-69	1.80E-68
Leprel2	2049	1012.5	318.5	17.70598	5.192118	-1.76984	Down	1.07E-84	7.26E-84
Foxk2	4359	3761.5	1183.5	30.88879	9.058257	-1.76978	Down	0	0
Anapc2	2681	3167	995.5	42.30451	12.409	-1.76942	Down	########	########
Eif2c1	4452	939	295.5	7.550364	2.215937	-1.76863	Down	9.73E-79	6.46E-78
LOC100911445	1014	94	30	3.339804	0.980675	-1.76792	Down	4.66E-09	1.09E-08
Coro2b	3310	2194.5	691.5	23.77966	6.983756	-1.76765	Down	########	########
LOC681300	948	150	47.5	5.675212	1.670489	-1.7644	Down	6.37E-14	2.51E-13
Pcdha4	5241	41.5	13	0.282961	0.083329	-1.76371	Down	0.000114	0.000211
Elfn2-ps1	2574	164.5	52	2.299656	0.677278	-1.7636	Down	6.84E-15	2.86E-14
Ptprs	6040	9393.5	2969.5	55.73356	16.41538	-1.7635	Down	0	0
Phf19	3789	660	208.5	6.230604	1.837081	-1.76196	Down	1.17E-55	6.91E-55
Sult2a2	993	39.5	12.5	1.42369	0.419824	-1.76178	Down	0.000128	0.000234
Ptpn5	2815	25.5	8	0.326082	0.096162	-1.7617	Down	0.002935	0.004799
RGD1561149	5170	389	123.5	2.700824	0.796755	-1.76119	Down	2.33E-33	1.19E-32

Nxn	1347	970	307.5	25.81714	7.619372	-1.76058	Down	1.48E-80	9.93E-80
Ehd3	2108	2792	881.5	47.39493	13.99527	-1.75979	Down	########	########
Bub1	4583	3891	1228.5	30.30778	8.949825	-1.75976	Down	0	0
Psmb8	1086	1905.5	601	62.7693	18.53699	-1.75965	Down	########	########
Keap1	2208	845.5	267.5	13.7305	4.055219	-1.75953	Down	1.52E-70	9.71E-70
Aldh18a1	3413	4158.5	1320	43.64228	12.89173	-1.75928	Down	0	0
Ncln	2923	6415.5	2039	78.74925	23.26658	-1.75901	Down	0	0
Lrp1	14714	30747.5	9725.5	74.6823	22.07723	-1.75821	Down	0	0
LOC100910245	772	160	51	7.406558	2.190449	-1.75758	Down	1.77E-14	7.27E-14
Ankrd13b	3085	855	271.5	9.951249	2.944528	-1.75684	Down	4.31E-71	2.77E-70
Spats2l	2506	580	186	8.302199	2.457201	-1.75648	Down	4.23E-48	2.39E-47
Syndig1	2055	294	93.5	5.128841	1.519278	-1.75525	Down	1.38E-25	6.58E-25
Prr7	970	1025	328.5	38.01909	11.26641	-1.7547	Down	6.98E-84	4.73E-83
Adrbk1	2683	2100	668.5	28.02527	8.305118	-1.75466	Down	########	########
Fam189b	3053	1076.5	342	12.65572	3.750636	-1.75458	Down	1.07E-88	7.34E-88
Fam120a	5018	15386	4897.5	109.7812	32.54409	-1.75416	Down	0	0
LOC100909464	980	290	92	10.59477	3.141619	-1.75377	Down	3.56E-25	1.69E-24
Evl	1854	841	267.5	16.24816	4.821263	-1.75279	Down	8.11E-70	5.16E-69
Aox1	4303	770	244.5	6.392567	1.896962	-1.7527	Down	3.22E-64	1.99E-63
Epb4.1	5108	5531.5	1759	38.75072	11.50084	-1.75248	Down	0	0
RGD1310852	1328	230.5	73	6.194677	1.838658	-1.75238	Down	2.85E-20	1.29E-19
Gnb4	1516	3062.5	974	72.17044	21.45749	-1.74993	Down	########	########
Twist1	1513	1875.5	598.5	44.39501	13.2042	-1.7494	Down	########	########
LOC100911256	5640	309	98.5	1.960159	0.583005	-1.74939	Down	9.90E-27	4.78E-26
Pacs1	4198	1742	557	14.87619	4.430051	-1.74761	Down	########	########

Cyp2a2	2259	175.5	55.5	2.769184	0.824842	-1.74727	Down	5.97E-16	2.54E-15
Gnptab	4370	2176	695.5	17.83104	5.311459	-1.74721	Down	########	########
Iars2	3053	2436.5	779.5	28.57162	8.512469	-1.74693	Down	########	########
Akr7a2	1272	814.5	261	22.91376	6.831491	-1.74594	Down	8.05E-67	5.04E-66
Fam134a	2541	1160.5	370.5	16.34019	4.873933	-1.74527	Down	4.56E-95	3.19E-94
Id3	983	6901	2208	250.7825	74.88217	-1.74374	Down	0	0
Padi3	3099	335.5	107.5	3.879378	1.158578	-1.74347	Down	1.31E-28	6.43E-28
Abca2	8040	2184	701.5	9.733559	2.90694	-1.74347	Down	########	########
Pcdhga8	4689	571.5	183	4.359523	1.304156	-1.74105	Down	2.04E-47	1.15E-46
Setd6	1683	302.5	97	6.420894	1.921046	-1.74088	Down	8.96E-26	4.29E-25
Tmem109	1924	2641.5	846.5	49.12204	14.69921	-1.74063	Down	########	########
Sgsm3	2854	795.5	256.5	9.98748	2.989406	-1.74026	Down	5.84E-65	3.62E-64
Fkbp8	1679	4389.5	1413.5	93.74453	28.09637	-1.73835	Down	0	0
Ergic3	1338	2619.5	842.5	70.03723	20.99951	-1.73777	Down	########	########
LOC500956	1875	130	42	2.487699	0.745945	-1.73767	Down	7.55E-12	2.01E-11
RGD1307722	1831	255.5	82.5	4.996525	1.498668	-1.73724	Down	5.67E-22	2.60E-21
Ppdpf	685	714	231.5	37.49571	11.24715	-1.73717	Down	3.51E-58	2.11E-57
Tyro3	3726	344.5	111.5	3.312539	0.994144	-1.73641	Down	5.57E-29	2.74E-28
Gprasp2	3799	490	157.5	4.599821	1.381435	-1.73541	Down	6.11E-41	3.30E-40
Ptp4a3	1353	46	15	1.220815	0.366816	-1.73472	Down	5.78E-05	0.00011
Sun1	2497	3688	1190	52.93903	15.91194	-1.73422	Down	########	########
LOC680768	1194	1586	509	47.40821	14.2546	-1.73371	Down	########	########
Arhgef2	4217	6695	2159	56.81907	17.09109	-1.73313	Down	0	0
Prelp	1446	708	229	17.59577	5.293002	-1.73307	Down	1.00E-57	6.00E-57
Tubb2a	1545	8261.5	2669	191.4987	57.60899	-1.73297	Down	0	0

Etv5	3897	6235.5	2014	57.30868	17.25087	-1.73208	Down	0	0
LOC683420	2269	1740	561	27.42877	8.257912	-1.73184	Down	########	########
Tmem9	1120	1655	532.5	52.81877	15.91744	-1.73044	Down	########	########
Cbln2	2385	165	53	2.465231	0.743061	-1.73017	Down	9.33E-15	3.88E-14
Plekhh3	3001	519	168	6.205061	1.870538	-1.72999	Down	9.92E-43	5.43E-42
Olfml2a	5515	122	39.5	0.793658	0.239371	-1.72927	Down	2.46E-11	6.28E-11
Dazl	2836	46.5	15	0.586411	0.176905	-1.72894	Down	5.78E-05	0.00011
LOC100911851	1907	416.5	135	7.799782	2.353602	-1.72856	Down	1.80E-34	9.32E-34
Mrc2	4443	4871.5	1582	39.26819	11.87291	-1.72569	Down	0	0
Coro1b	1800	5975.5	1940.5	118.9995	35.98309	-1.72557	Down	0	0
LOC100910835	1213	1934	630	57.21236	17.30758	-1.72492	Down	########	########
Gpd1l	1261	425.5	138	12.07731	3.655179	-1.72429	Down	3.71E-35	1.93E-34
LOC295112	1941	112.5	37	2.068524	0.626229	-1.72384	Down	3.83E-10	9.32E-10
Hoxa13	1412	43	14	1.093371	0.331031	-1.72375	Down	0.0001	0.000187
Eef2	2944	86448.5	28084.5	1051.743	318.474	-1.72353	Down	0	0
Mpst	1304	742.5	242	20.42011	6.186356	-1.72283	Down	8.58E-60	5.20E-59
Ankrd41	1821	341	110	6.685959	2.026661	-1.72203	Down	9.44E-29	4.64E-28
Setd8	969	2532	823.5	93.55792	28.36851	-1.72157	Down	########	########
LOC100911774	1172	728.5	237.5	22.2808	6.756841	-1.72138	Down	7.85E-59	4.74E-58
Sptan1	7866	18249	5925.5	82.9691	25.16116	-1.72138	Down	0	0
Slc8a1	6134	836	272	4.870149	1.47695	-1.72134	Down	2.39E-67	1.50E-66
Nptxr	4870	65268	21277	480.6653	145.8206	-1.72084	Down	0	0
Pip5k1c	4343	3092.5	1007	25.55401	7.754783	-1.72039	Down	########	########
Ankrd34a	2887	228	74.5	2.837491	0.86109	-1.72038	Down	1.36E-19	6.08E-19
LOC691221	2357	342.5	111.5	5.192794	1.576147	-1.72011	Down	1.28E-28	6.27E-28

Cotl1	1618	4796	1567	106.3782	32.29606	-1.71977	Down	0	0
Slc36a2	2436	37	12	0.546566	0.165945	-1.71969	Down	0.000306	0.000549
Larp1	6337	12733	4143.5	71.87836	21.83557	-1.71888	Down	0	0
Gda	1460	1147	373	28.03059	8.519165	-1.71822	Down	8.68E-92	6.02E-91
Pnp	1052	3360	1094	114.2707	34.73258	-1.71809	Down	########	########
LOC100911673	1014	84	27.5	2.976167	0.905017	-1.71744	Down	3.58E-08	8.14E-08
LOC100910370	848	4436.5	1446.5	187.2434	56.9574	-1.71696	Down	0	0
Grk6	2828	3900	1273.5	49.37524	15.02116	-1.71679	Down	########	########
Fam210b	1772	303.5	99	6.123448	1.863259	-1.71652	Down	2.49E-25	1.18E-24
RGD1306058	840	1317.5	430.5	56.11986	17.11342	-1.71338	Down	########	########
Ltbp4	5157	1417	464	9.847915	3.00432	-1.71278	Down	########	########
Pcdhga10	4689	564	184	4.302105	1.313579	-1.71154	Down	7.62E-46	4.25E-45
Atg2a	6340	1307.5	428.5	7.394374	2.257997	-1.71138	Down	########	########
Bend4	1383	134	44	3.477847	1.062198	-1.71114	Down	5.98E-12	1.61E-11
Zc3h7b	5466	5116.5	1676	33.532	10.24308	-1.71089	Down	0	0
fut10	1311	273.5	90	7.473168	2.283076	-1.71074	Down	9.94E-23	4.59E-22
Tmem150a	1482	301.5	99	7.290041	2.228471	-1.70987	Down	5.67E-25	2.69E-24
Arhgap31	4566	2737.5	899	21.49612	6.58043	-1.70782	Down	########	########
Vcan	4175	26228	8583.5	224.2786	68.66018	-1.70775	Down	0	0
Phc1	3917	1792.5	590	16.39996	5.024998	-1.7065	Down	########	########
Rnf187	1905	4423.5	1453.5	83.16004	25.48167	-1.70643	Down	0	0
Igtp	2125	401	132	6.741982	2.073063	-1.70141	Down	1.04E-32	5.29E-32
Shisa2	959	106	35	3.971928	1.221312	-1.70141	Down	1.12E-09	2.68E-09
Nxpe3	3137	497	163	5.663529	1.741967	-1.70098	Down	2.48E-40	1.33E-39
LOC681410	2684	7386.5	2432.5	98.05473	30.17636	-1.70017	Down	0	0

Wdr6	4164	4377	1444	37.61295	11.57967	-1.69964	Down	0	0
Tbcc	1184	1347	446.5	40.78745	12.56052	-1.69923	Down	########	########
Palmd	2262	1100	363.5	17.40147	5.360237	-1.69884	Down	1.77E-86	1.21E-85
Pcdhga1	4548	637	210.5	5.021816	1.54696	-1.69877	Down	6.91E-51	3.97E-50
Calr	1865	44052.5	14568	846.4899	260.9904	-1.6975	Down	0	0
LOC100911326	1008	701.5	232.5	24.99402	7.706631	-1.69741	Down	1.49E-55	8.82E-55
Ssx2ip	2547	1621	534	22.70143	6.999826	-1.69739	Down	########	########
LOC497848	2103	829.5	274.5	14.09854	4.347422	-1.69731	Down	1.74E-65	1.08E-64
Tmem214	2877	2878	953	35.8736	11.0665	-1.69672	Down	########	########
Gnaz	1529	36	12	0.845509	0.260851	-1.69659	Down	0.00047	0.000834
Fgf22	489	57	19	4.162843	1.284354	-1.69653	Down	9.78E-06	1.96E-05
Srf	4106	2766	919	24.175	7.460321	-1.69621	Down	########	########
Plec	14886	32714.5	10834.5	78.69504	24.29541	-1.69559	Down	0	0
RGD1311849	5746	1123.5	372	6.996251	2.162112	-1.69414	Down	8.06E-88	5.51E-87
Fam125b	1376	822.5	273.5	21.45413	6.630554	-1.69405	Down	1.50E-64	9.30E-64
Rpl18a	645	15901	5280	884.0377	273.239	-1.69394	Down	0	0
Lrrc59	2825	12369	4098.5	156.6171	48.41501	-1.69372	Down	0	0
Ppap2c	1603	889	294	19.79226	6.119977	-1.69334	Down	4.79E-70	3.06E-69
LOC100911928	1168	177.5	59	5.431321	1.679556	-1.69322	Down	4.63E-15	1.94E-14
Steap3	2707	2309	765.5	30.53741	9.450778	-1.69207	Down	########	########
Pcnxl3	7280	3640.5	1210.5	17.92751	5.548446	-1.69202	Down	########	########
Tcof1	4627	3679	1223	28.47833	8.815597	-1.69173	Down	########	########
Amot	5853	918	304	5.598523	1.735314	-1.68985	Down	3.79E-72	2.44E-71
Sh3d21	1876	68.5	23	1.304903	0.405009	-1.68792	Down	1.67E-06	3.51E-06
LOC100359678	7422	1312.5	436.5	6.323588	1.962745	-1.68787	Down	########	########

Gli3	5027	1690	564.5	12.05281	3.743665	-1.68685	Down	########	########
Ptch1	4305	769.5	256	6.39976	1.988298	-1.68648	Down	2.56E-60	1.55E-59
Cic	5429	8074.5	2698	53.42735	16.60122	-1.68629	Down	0	0
Mapkapk2	2909	3586.5	1195.5	44.115	13.71079	-1.68596	Down	########	########
Trim62	1546	385.5	129.5	8.962907	2.787026	-1.68524	Down	8.82E-31	4.41E-30
Anxa8	1856	1947.5	650	37.56911	11.6887	-1.68443	Down	########	########
RGD1308059	1224	416.5	139	12.1479	3.781182	-1.6838	Down	3.02E-33	1.54E-32
LOC100910951	649	27	9	1.482486	0.461604	-1.68329	Down	0.002563	0.004214
Kif1c	4117	9668.5	3227.5	84.07528	26.18554	-1.68291	Down	0	0
Angpt1	1891	1043.5	348.5	19.74045	6.150372	-1.68241	Down	6.14E-81	4.12E-80
Ckap4	2548	11345	3791.5	159.386	49.67227	-1.68201	Down	0	0
Rai1	7600	1636	548	7.720015	2.407839	-1.68086	Down	########	########
Glb1	2833	3159	1061.5	39.92802	12.47781	-1.67804	Down	########	########
LOC691806	1151	154.5	51.5	4.767103	1.490331	-1.67748	Down	2.17E-13	7.83E-13
Shox2	1588	844.5	284.5	19.05502	5.958499	-1.67715	Down	4.05E-65	2.51E-64
Sardh	3071	1718	577.5	20.04574	6.269141	-1.67696	Down	########	########
RGD1560795	771	217	73	10.08839	3.155301	-1.67685	Down	6.22E-18	2.72E-17
LOC687090	1685	2715	910.5	57.67198	18.04477	-1.67629	Down	########	########
Pcdhgc5	3157	655	219.5	7.445576	2.3303	-1.67587	Down	2.39E-51	1.38E-50
LOC679881	1902	240.5	80.5	4.509114	1.412349	-1.67475	Down	6.74E-20	3.03E-19
Acads	1748	865.5	291	17.7368	5.556042	-1.67462	Down	1.03E-66	6.43E-66
Ubtf	3204	4868	1635	54.39753	17.04233	-1.67442	Down	0	0
Neto2	1578	930.5	313	21.12375	6.61893	-1.6742	Down	1.49E-71	9.54E-71
Lphn2	5928	4548.5	1528	27.44343	8.599856	-1.67408	Down	0	0
Vapb	732	1987.5	668.5	97.3763	30.52805	-1.67344	Down	########	########

Galk1	1486	2668.5	894.5	64.1518	20.11208	-1.67343	Down	########	########
Extl3	3198	13183.5	4435.5	147.7082	46.32997	-1.67273	Down	0	0
Inppl1	4640	6200.5	2093.5	47.88483	15.05016	-1.66979	Down	0	0
Efna4	1199	117.5	39.5	3.512197	1.104028	-1.6696	Down	1.98E-10	4.88E-10
LOC690801	2055	1346.5	452	23.36467	7.34654	-1.66919	Down	########	########
Dag1	5169	17446.5	5887.5	120.9623	38.03974	-1.66898	Down	0	0
Ilvbl	2632	1314	444	17.88817	5.626308	-1.66875	Down	########	5.70E-99
Iqgap1	6158	22308	7518	129.5828	40.76339	-1.66853	Down	0	0
Bcat2	1548	532	179	12.27833	3.86469	-1.66769	Down	1.10E-41	5.97E-41
Numa1	7793	6887.5	2329.5	31.69479	9.978266	-1.66738	Down	0	0
Mum1	2463	925.5	311.5	13.43257	4.22925	-1.66726	Down	2.83E-71	1.82E-70
Sox2	2323	2209.5	744.5	33.98268	10.69958	-1.66725	Down	########	########
LOC310891	2079	78.5	26.5	1.35126	0.425782	-1.66612	Down	2.17E-07	4.76E-07
LOC100361061	613	3528.5	1194.5	205.8775	64.89281	-1.66566	Down	########	########
Mboat7	2303	921	312.5	14.33882	4.521158	-1.66516	Down	2.82E-70	1.80E-69
Cdc42bpb	6397	5763	1950.5	32.27149	10.17634	-1.66504	Down	0	0
Pcdhga5	4719	558	188	4.234119	1.335436	-1.66475	Down	1.45E-43	7.95E-43
Nudc	1294	2539	861	70.32446	22.18636	-1.66435	Down	########	########
Prpf19	2156	7108	2410	118.0939	37.26086	-1.6642	Down	0	0
Cmtm3	1519	770.5	261.5	18.15395	5.728374	-1.66409	Down	5.34E-59	3.23E-58
Mxd4	1185	252	85	7.597985	2.398632	-1.6634	Down	1.62E-20	7.34E-20
LOC100911286	1025	464.5	158	16.30682	5.149089	-1.66309	Down	5.20E-36	2.71E-35
Man2a1	6959	2185	738.5	11.21749	3.54222	-1.66302	Down	########	########
Dpy19l1	4663	3586.5	1212.5	27.45511	8.677087	-1.66179	Down	########	########
Abcb10	3460	631	214	6.528106	2.063664	-1.66146	Down	1.26E-48	7.13E-48

Hcn4	3971	35.5	12	0.321989	0.101799	-1.6613	Down	0.000717	0.001235
LOC362306	879	35.5	12	1.444844	0.456817	-1.66123	Down	0.000717	0.001235
Eif4ebp2	1759	2375.5	808	48.44055	15.32095	-1.66071	Down	########	########
Fpgs	2586	915.5	311	12.68811	4.014849	-1.66006	Down	1.59E-69	1.01E-68
Nrp1	3862	1217	413	11.2636	3.567103	-1.65884	Down	4.61E-92	3.20E-91
Taar3	1029	173	59	6.038325	1.912558	-1.65865	Down	2.36E-14	9.66E-14
Abcb9	3303	508	173	5.507613	1.744601	-1.65853	Down	2.90E-39	1.55E-38
Rapgef5	5460	1730	587.5	11.32384	3.588901	-1.65775	Down	########	########
Nt5c3l	1683	1288	437	27.39138	8.681365	-1.65773	Down	2.36E-97	1.66E-96
Daglb	3019	559	190	6.623937	2.101191	-1.65648	Down	3.80E-43	2.08E-42
Klc2	3008	2126.5	726	25.3801	8.053632	-1.65599	Down	########	########
Trove2	3737	1191	405	11.36593	3.611335	-1.65411	Down	6.82E-90	4.70E-89
Pcdhga11	4688	590.5	201	4.507558	1.432818	-1.65349	Down	2.61E-45	1.45E-44
Lgr5	3130	38	13	0.435162	0.13838	-1.65292	Down	0.00041	0.000729
RGD1561416	945	87	29.5	3.29615	1.048425	-1.65256	Down	4.28E-08	9.68E-08
Csk	2108	3164.5	1082	53.80338	17.12833	-1.65131	Down	########	########
RT1-T24-3	1458	248.5	85	6.132091	1.952592	-1.65099	Down	8.17E-20	3.66E-19
Adcy2	4008	27.5	9.5	0.246592	0.078574	-1.65	Down	0.002563	0.004214
Slc2a12	4118	86.5	29	0.750873	0.239271	-1.64992	Down	6.47E-08	1.45E-07
Reck	3863	1970.5	672	18.24226	5.813497	-1.64981	Down	########	########
Tpx2	3170	5726.5	1954	64.5809	20.58458	-1.64954	Down	0	0
Fam83d	2253	1399	478	22.24428	7.091388	-1.64929	Down	########	########
Smad6	1377	431.5	149	11.28495	3.598936	-1.64876	Down	6.54E-33	3.33E-32
Chrd	3267	134	46	1.470501	0.468991	-1.64868	Down	2.40E-11	6.14E-11
Tpt1	794	23613.5	8064.5	1063.282	339.2137	-1.64826	Down	0	0

Tmem121	1587	172	59	3.884712	1.239522	-1.64802	Down	3.54E-14	1.43E-13
Dlat	2628	2773	947.5	37.75277	12.0474	-1.64786	Down	########	########
Arid1b	8301	3273	1121.5	14.13197	4.510319	-1.64766	Down	########	########
Ppp2r5d	2924	4006	1374	49.11	15.69538	-1.64568	Down	########	########
Flii	4035	4928.5	1689	43.74926	13.98533	-1.64534	Down	0	0
Itpk1	1635	1622	558	35.58815	11.38009	-1.64489	Down	########	########
Fam63a	2054	756.5	259.5	13.1902	4.220461	-1.64399	Down	3.78E-57	2.25E-56
Vim	1796	20726.5	7134	413.6006	132.3878	-1.64347	Down	0	0
Gna12	3345	9412	3230.5	100.7102	32.24196	-1.6432	Down	0	0
Cnnm3	4107	926.5	318	8.06726	2.584443	-1.64223	Down	2.21E-69	1.41E-68
Tkt	2098	7813	2686.5	133.4858	42.77022	-1.64201	Down	0	0
Adh5	1413	2208.5	756	55.75887	17.87065	-1.64161	Down	########	########
Mkrn2	2234	704.5	242	11.29092	3.619476	-1.64131	Down	4.24E-53	2.47E-52
Chst2	1998	6771.5	2333.5	121.3305	38.90364	-1.64096	Down	0	0
LOC100360180	2148	9146.5	3149	152.6083	48.93635	-1.64085	Down	0	0
Fbxl18	2322	157.5	54.5	2.439135	0.782658	-1.63992	Down	5.06E-13	1.70E-12
Scap	4301	2839	977.5	23.64536	7.588016	-1.63976	Down	########	########
Pigq	3024	2690	926.5	31.85829	10.24207	-1.63716	Down	########	########
LOC683522	1869	475	165	9.123334	2.933506	-1.63694	Down	7.10E-36	3.70E-35
Telo2	3211	573.5	197.5	6.385074	2.053113	-1.63689	Down	1.58E-43	8.69E-43
E2f1	2643	1476.5	509	19.97875	6.427729	-1.63609	Down	########	########
Bgn	2446	42414.5	14620.5	620.3845	199.6279	-1.63585	Down	0	0
LOC100910032	899	78	27	3.121885	1.004721	-1.63562	Down	4.33E-07	9.39E-07
Acsl1	3657	2192	754.5	21.42866	6.899207	-1.63504	Down	########	########
Lsp1	1257	326	112.5	9.288965	2.991302	-1.63474	Down	1.74E-25	8.30E-25

Bhlhb9	2701	235	80.5	3.100788	0.998552	-1.63473	Down	5.15E-19	2.28E-18
Arpc1b	1536	21732.5	7493	505.9814	162.9526	-1.63463	Down	0	0
Exoc7	2328	264	91.5	4.060109	1.307793	-1.63438	Down	7.64E-21	3.47E-20
Tmem63a	3269	3422	1182	37.4745	12.07415	-1.63399	Down	########	########
Dis3l	3234	762	263	8.441229	2.719862	-1.63392	Down	5.07E-57	3.02E-56
Rfc2	1556	1381.5	476	31.67795	10.21692	-1.63252	Down	########	########
Lrp10	2941	7744	2683	94.39091	30.44662	-1.63237	Down	0	0
Mrps30	1435	1245.5	432	31.07883	10.03213	-1.6313	Down	2.30E-91	1.59E-90
Nol3	1532	263.5	91	6.151067	1.986097	-1.6309	Down	1.14E-20	5.16E-20
Il16	4985	99	34	0.709605	0.229158	-1.63067	Down	9.92E-09	2.30E-08
Col18a1	5007	368	127.5	2.633972	0.851342	-1.62943	Down	2.13E-28	1.04E-27
LOC100909797	1005	84	29	2.982279	0.964296	-1.62887	Down	1.47E-07	3.24E-07
Ehmt2	4023	4496.5	1562	40.027	12.961	-1.6268	Down	0	0
Ddb1	4249	20877.5	7241.5	175.5042	56.8937	-1.62516	Down	0	0
Diaph1	5588	7424.5	2577	47.5408	15.41171	-1.62514	Down	0	0
Ajap1	2603	1155.5	402	15.90693	5.159277	-1.62441	Down	1.54E-84	1.04E-83
RGD1304694	3211	3519	1224	39.26307	12.7396	-1.62385	Down	########	########
Scn1a	8399	998	346.5	4.243429	1.376888	-1.62382	Down	1.10E-73	7.13E-73
Ptpn11	5526	8426.5	2929.5	54.50311	17.68495	-1.62382	Down	0	0
Itpkb	3951	287.5	100	2.609114	0.846616	-1.62378	Down	3.24E-22	1.49E-21
Gnb2	1670	15010	5235.5	322.2273	104.5757	-1.62353	Down	0	0
Tecr	1178	4826.5	1684.5	147.1462	47.78059	-1.62275	Down	0	0
RGD1308908	7104	3457.5	1207	17.45343	5.671802	-1.62163	Down	########	########
Nkx6-1	3211	168.5	59	1.883739	0.61262	-1.62054	Down	1.76E-13	6.47E-13
Galntl4	2546	259.5	90.5	3.648655	1.187296	-1.61968	Down	2.88E-20	1.30E-19

Kcnk12	1900	73.5	26	1.397375	0.454977	-1.61885	Down	1.67E-06	3.50E-06
Sorcs1	4416	57.5	20	0.465473	0.151616	-1.61828	Down	1.95E-05	3.85E-05
Nin	6718	351	122.5	1.870701	0.609398	-1.61812	Down	6.57E-27	3.18E-26
Tln1	8647	20217	7066.5	83.68763	27.26256	-1.61809	Down	0	0
Gnl1	2083	2857.5	998	49.09929	16.00352	-1.61731	Down	########	########
Osr2	1739	386.5	135.5	7.978858	2.600669	-1.6173	Down	3.41E-29	1.68E-28
Snd1	3475	13205	4616.5	136.0472	44.34845	-1.61715	Down	0	0
Fgf2	1016	243.5	84.5	8.567253	2.793146	-1.61694	Down	3.09E-19	1.37E-18
Zfpm1	3088	1225	431.5	14.26566	4.652026	-1.61662	Down	3.16E-88	2.17E-87
Mapre2	2463	3250	1137	47.29548	15.42339	-1.61658	Down	########	########
Ncdn	3662	4015.5	1407	39.3163	12.82161	-1.61655	Down	########	########
Ppm1f	1935	1268	443	23.44924	7.649642	-1.61608	Down	3.76E-92	2.61E-91
Plxnb1	8395	655.5	229.5	2.793179	0.911486	-1.61562	Down	2.06E-48	1.17E-47
LOC100910912	789	83	29	3.756274	1.226004	-1.61534	Down	2.20E-07	4.84E-07
LOC100909460	3300	2501	874	27.1164	8.850901	-1.61527	Down	########	########
LOC363527	1427	225	79	5.648897	1.843909	-1.6152	Down	1.44E-17	6.28E-17
Plxna3	7376	1138	397.5	5.522186	1.803092	-1.61477	Down	4.65E-83	3.14E-82
Penk	1446	6632.5	2321.5	164.203	53.61717	-1.61471	Down	0	0
St3gal5	2723	6129.5	2146.5	80.53209	26.30196	-1.61439	Down	0	0
Myo1d	5303	3552.5	1248	24.0342	7.856014	-1.61322	Down	########	########
Psmd3	2147	6936	2437	115.8916	37.88177	-1.6132	Down	0	0
Nedd4	5373	64672.5	22666	430.2854	140.7821	-1.61183	Down	0	0
Rplp0	1093	61433	21580	2012.933	658.6465	-1.61172	Down	0	0
Sipa1	3123	2089	736.5	24.00932	7.856207	-1.61169	Down	########	########
Mocos	3037	549.5	192.5	6.484546	2.121999	-1.61158	Down	7.81E-41	4.21E-40

Hdac6	4145	1495.5	524	12.91564	4.226753	-1.6115	Down	########	########
Wbscr17	1797	79	28	1.577895	0.516711	-1.61057	Down	5.65E-07	1.22E-06
Ccdc6	1633	829.5	292	18.20931	5.964451	-1.61022	Down	3.40E-60	2.07E-59
Kpnb1	2991	18912	6636	226.0074	74.04586	-1.60988	Down	0	0
Mtss1l	3447	1342	472.5	13.9697	4.578218	-1.60944	Down	1.59E-96	1.12E-95
Adra1a	1428	28.5	10	0.717173	0.235062	-1.60928	Down	0.003378	0.005506
Vamp5	309	500.5	176	58.05886	19.031	-1.60916	Down	5.17E-37	2.72E-36
Hr	5233	4788	1683.5	32.78284	10.74761	-1.60892	Down	0	0
LOC100911724	1619	328	116	7.209806	2.364897	-1.60818	Down	1.12E-24	5.28E-24
Hspa12b	3350	193	68	2.063424	0.677702	-1.60632	Down	3.27E-15	1.38E-14
LOC100362543	1161	161	57	4.973548	1.633719	-1.60612	Down	7.55E-13	2.43E-12
LOC100362423	605	241	85.5	14.29016	4.695243	-1.60575	Down	1.33E-18	5.87E-18
RGD1310444	4321	4366	1538	36.17633	11.88711	-1.60565	Down	0	0
Wdr1	2844	15980.5	5626.5	200.9137	66.05909	-1.60475	Down	0	0
Pcnxl2	6378	117	41	0.658217	0.216457	-1.60449	Down	7.79E-10	1.88E-09
Adam15	2815	3066.5	1081.5	39.03327	12.83861	-1.60422	Down	########	########
Serpina5	2187	139.5	49	2.274735	0.748447	-1.60373	Down	2.41E-11	6.16E-11
Aacs	3190	2913	1028.5	32.67298	10.75605	-1.60295	Down	########	########
Fam101b	1384	163.5	58	4.23619	1.394606	-1.60291	Down	6.59E-13	2.15E-12
MGC108823	1628	88	31	1.929298	0.635191	-1.60281	Down	1.12E-07	2.49E-07
Lpcat4	2488	1718.5	608	24.75694	8.151238	-1.60274	Down	########	########
Prex1	5438	1139.5	402.5	7.513081	2.473895	-1.60262	Down	8.34E-82	5.61E-81
Dock7	6759	4298	1515	22.72622	7.487102	-1.60188	Down	########	########
Zfp362	1890	456	162	8.671037	2.856965	-1.60172	Down	1.77E-33	9.05E-33
LOC100910536	5009	777.5	275	5.559618	1.832077	-1.60151	Down	3.72E-56	2.20E-55

Nbeal2-ps1	8696	826	292.5	3.402342	1.12171	-1.60083	Down	1.10E-59	6.68E-59
Top2a	4987	13507	4766	96.68868	31.88146	-1.60063	Down	0	0
Rrbp1	5141	12018	4245.5	83.56913	27.57692	-1.59951	Down	0	0
Chpf2	3763	1975	700	18.80846	6.208044	-1.59917	Down	########	########
Cpt1a	4356	2579.5	913.5	21.19344	6.998577	-1.59848	Down	########	########
Sc5dl	2185	5081	1803.5	83.37227	27.54905	-1.59757	Down	0	0
Sptbn1	7332	27032	9572.5	131.8619	43.60671	-1.59641	Down	0	0
Stat5b	2671	610.5	217	8.196331	2.712444	-1.59538	Down	3.82E-44	2.11E-43
LOC100192314	1494	178	63	4.277916	1.415709	-1.59538	Down	4.64E-14	1.85E-13
Cdon	4573	340	121	2.663975	0.882026	-1.59469	Down	2.61E-25	1.24E-24
Tmem51	2034	625.5	222	11.00935	3.645758	-1.59444	Down	2.78E-45	1.54E-44
Duoxa1	1665	103	36.5	2.207418	0.731092	-1.59423	Down	7.60E-09	1.77E-08
Ap2a2	3258	6203	2205	68.20617	22.59743	-1.59374	Down	0	0
Agpat2	1362	1005	357.5	26.4355	8.762434	-1.59307	Down	9.53E-72	6.13E-71
H1fx	1058	387.5	139	13.19719	4.374448	-1.59306	Down	3.11E-28	1.52E-27
Rcc1	1584	1321.5	469.5	29.8661	9.900163	-1.59298	Down	8.38E-94	5.84E-93
Hcfc1r1	629	2844.5	1012.5	162.1615	53.76057	-1.59281	Down	########	########
Hpdl	1631	38	13.5	0.83405	0.276623	-1.59221	Down	0.00041	0.000729
Gsn	2612	12095	4306	165.958	55.05931	-1.59176	Down	0	0
Bak1	1923	1925.5	687.5	35.93069	11.92105	-1.59171	Down	########	########
Lss	2851	2306.5	817.5	28.91204	9.595325	-1.59127	Down	########	########
Chml	1866	151	53.5	2.900866	0.962779	-1.59121	Down	2.87E-12	8.34E-12
Nr2f6	1775	1324.5	474	26.74965	8.881209	-1.59069	Down	6.53E-93	4.54E-92
Fam102b	5732	4272	1514.5	26.58904	8.82934	-1.59045	Down	########	########
Elmo2	4577	3057.5	1089	23.94085	7.949976	-1.59045	Down	########	########

Slc26a6	2543	249.5	88.5	3.503453	1.163501	-1.5903	Down	4.04E-19	1.79E-18
Xdh	4198	2466	876.5	20.99884	6.974795	-1.59009	Down	########	########
Apol3	1455	71.5	25.5	1.75507	0.582965	-1.59005	Down	1.92E-06	4.01E-06
Lasp1	3425	11306.5	4028.5	118.0977	39.25171	-1.58915	Down	0	0
Fam125a	1102	1515.5	542.5	49.43044	16.43139	-1.58894	Down	########	########
Ndst1	4051	1557.5	557.5	13.76314	4.580374	-1.58727	Down	########	########
2-Sep	3225	20782	7399.5	229.9999	76.58393	-1.58652	Down	0	0
LOC100910833	1251	432	154	12.34947	4.114284	-1.58574	Down	1.18E-31	5.93E-31
Pa2g4	1602	7940	2843.5	177.6787	59.23836	-1.58467	Down	0	0
Calml4	896	306.5	109.5	12.26074	4.089739	-1.58397	Down	6.61E-23	3.06E-22
Mtor	8554	4723.5	1690	19.7616	6.592286	-1.58385	Down	0	0
RGD1311078	872	252.5	90.5	10.37634	3.463482	-1.583	Down	4.57E-19	2.03E-18
Cobl	4777	223.5	80	1.674959	0.559126	-1.58288	Down	6.06E-17	2.62E-16
Mbnl1	1164	2323.5	833	71.62543	23.9129	-1.58268	Down	########	########
Sema6c	3272	56	20	0.612777	0.204626	-1.58237	Down	2.93E-05	5.70E-05
Fdxr	1786	212.5	76.5	4.258968	1.423763	-1.5808	Down	3.39E-16	1.45E-15
LOC100359865	737	388.5	138.5	18.83738	6.301661	-1.57979	Down	1.11E-28	5.42E-28
Xxylt1	2820	2073.5	744.5	26.31235	8.805578	-1.57925	Down	########	########
Bcl9l	5711	5682.5	2048	35.71978	11.9557	-1.57902	Down	0	0
Mcm2	3261	5786	2078.5	63.5527	21.27419	-1.57885	Down	0	0
Creld2	1368	1705.5	611	44.55663	14.92233	-1.57817	Down	########	########
Pcdhga2	4718	614	219.5	4.655732	1.56044	-1.57706	Down	2.92E-44	1.61E-43
Zfp277	1593	863.5	310.5	19.36261	6.489802	-1.57703	Down	6.70E-61	4.08E-60
Gpam	2646	837	302	11.34237	3.802085	-1.57686	Down	9.52E-59	5.73E-58
Adam12	2646	6222	2240	84.28247	28.27043	-1.57594	Down	0	0

Man2b1	3150	2685.5	967	30.55969	10.25094	-1.57587	Down	########	########
Aph1a	2069	2784	999.5	48.14413	16.15189	-1.57566	Down	########	########
Nmral1	1016	405	145.5	14.27132	4.787925	-1.57565	Down	1.33E-29	6.60E-29
Mbd3	1744	3876	1394.5	79.52511	26.6896	-1.57513	Down	########	########
Whsc1	5698	4793	1725	30.06021	10.10066	-1.57341	Down	0	0
Eef2k	2175	1020.5	368.5	16.80844	5.655536	-1.57145	Down	3.27E-71	2.10E-70
Fam211a	1901	570.5	206	10.72833	3.611301	-1.57083	Down	1.84E-40	9.92E-40
Fam20b	5051	3513.5	1266	24.87481	8.37656	-1.57026	Down	########	########
Nrp2	3338	14231	5145	152.8545	51.48288	-1.56999	Down	0	0
Strn	2985	1595.5	575.5	19.1422	6.44729	-1.56999	Down	########	########
Ncapd2	4579	4664	1688	36.461	12.28607	-1.56933	Down	0	0
Cdipt	1890	2520	910.5	47.72888	16.0923	-1.56849	Down	########	########
Vangl2	2813	68.5	25	0.874933	0.295119	-1.56788	Down	6.34E-06	1.29E-05
LOC100911431	7029	7754	2806	39.52897	13.33632	-1.56755	Down	0	0
LOC100912309	377	36	13	3.416967	1.153657	-1.5665	Down	0.000936	0.001595
Akr1a1	1124	10738.5	3871	340.8196	115.0722	-1.56647	Down	0	0
LOC683720	1935	306.5	111	5.676436	1.916608	-1.56643	Down	2.38E-22	1.10E-21
Zcchc14	5409	4219.5	1530.5	27.94967	9.438374	-1.56622	Down	########	########
Smo	2382	112	41	1.689244	0.570512	-1.56605	Down	5.61E-09	1.31E-08
Dagla	5568	1699	616.5	10.94544	3.696735	-1.56601	Down	########	########
Itgb8	2791	744	269	9.495014	3.208832	-1.56512	Down	2.92E-52	1.69E-51
Eif4g1	5388	28790	10444.5	191.4084	64.69651	-1.56489	Down	0	0
Ywhaq	2099	13829	5003.5	235.2922	79.54972	-1.56452	Down	0	0
Efr3b	2600	135	49	1.853344	0.62679	-1.56408	Down	1.17E-10	2.89E-10
Tbc1d16	2301	239	87	3.72595	1.260388	-1.56374	Down	1.05E-17	4.61E-17

Parp14	7087	1689	610.5	8.5127	2.880316	-1.56339	Down	########	########
LOC100360417	3722	366	133	3.519179	1.193269	-1.56032	Down	2.32E-26	1.12E-25
Man2a2	4200	1604.5	584	13.6921	4.643214	-1.56015	Down	########	########
Nadk	3443	2313.5	841	24.02439	8.149436	-1.55973	Down	########	########
Barx2-ps1	873	48	17.5	1.97009	0.668751	-1.55872	Down	0.000101	0.000189
Phospho1	1999	329	120.5	5.912235	2.006934	-1.55871	Down	9.87E-24	4.61E-23
Nacc2	3165	1499.5	545	16.97143	5.763006	-1.55822	Down	########	########
Stxbp1	3546	4235.5	1543.5	42.76995	14.52434	-1.55813	Down	########	########
Dpy19l3	7610	2543	924.5	11.95195	4.059482	-1.55788	Down	########	########
Got2	2372	9900	3602	149.3175	50.72268	-1.55768	Down	0	0
Glipr2	680	898	326.5	47.34684	16.08683	-1.55739	Down	2.36E-62	1.45E-61
Celsr1	9228	457	167	1.773281	0.602644	-1.55704	Down	2.91E-32	1.47E-31
Pdia4	2398	11929.5	4343	178.1015	60.53529	-1.55685	Down	0	0
LOC100910198	756	30.5	11	1.439464	0.489359	-1.55657	Down	0.002887	0.004723
Mef2c	1899	1832	667.5	34.50896	11.7381	-1.55577	Down	########	########
Aatk	5297	477	174.5	3.2313	1.099613	-1.55512	Down	1.10E-33	5.62E-33
Yif1a	1109	787.5	288	25.44351	8.666277	-1.55381	Down	3.09E-54	1.81E-53
Ttc13	3306	2386.5	871	25.87253	8.812981	-1.55372	Down	########	########
Dbn1	2697	4138.5	1514.5	54.99476	18.74185	-1.55303	Down	########	########
LOC690911	11965	2669.5	979.5	7.99739	2.727792	-1.5518	Down	########	########
Rnf26	2222	2515.5	919.5	40.46036	13.81095	-1.5507	Down	########	########
Fkbp9	3028	4547.5	1662.5	53.71305	18.34548	-1.54985	Down	########	########
Gp1bb	947	62	23	2.35423	0.80422	-1.54959	Down	1.88E-05	3.70E-05
Clcn4	4415	328.5	119.5	2.652401	0.906427	-1.54904	Down	7.71E-24	3.61E-23
Gatsl2	4257	467.5	171.5	3.937563	1.345781	-1.54886	Down	7.73E-33	3.94E-32

Elac2	2957	897	329.5	10.85981	3.714983	-1.54757	Down	2.28E-61	1.39E-60
Gpr64	4524	84	31	0.663396	0.226987	-1.54726	Down	5.44E-07	1.17E-06
Clstn1	4408	7906	2898	64.14389	21.9617	-1.54632	Down	0	0
Acsl3	2779	4488	1643	57.71413	19.76388	-1.54606	Down	########	########
Col4a2	6432	9262.5	3404.5	51.65505	17.68956	-1.54601	Down	0	0
Trak2	6041	3184	1166.5	18.82677	6.447867	-1.54589	Down	########	########
LOC683923	3034	248	91	2.927766	1.002868	-1.54567	Down	4.08E-18	1.79E-17
LOC100362733	979	630	231.5	22.97604	7.875992	-1.5446	Down	1.23E-43	6.77E-43
Vax2	879	344	127	14.03391	4.81459	-1.54343	Down	2.50E-24	1.18E-23
Fam114a1l1	1701	41	15	0.856432	0.293887	-1.54308	Down	0.00046	0.000817
LOC100910501	1023	27.5	10	0.961074	0.329881	-1.5427	Down	0.005098	0.008182
Acpl2	2859	1462	538.5	18.31523	6.288334	-1.54229	Down	2.11E-98	1.49E-97
Oasl2	1750	613	225.5	12.52256	4.301872	-1.54149	Down	1.93E-42	1.05E-41
Itsn1	4025	1540.5	567	13.70219	4.707658	-1.54132	Down	########	########
LOC100911062	1285	79.5	29.5	2.220743	0.763316	-1.54069	Down	1.08E-06	2.29E-06
Fam129b	3678	15196	5604	148.0508	50.89883	-1.54039	Down	0	0
Sft2d2	5083	1081.5	397	7.595422	2.612053	-1.53995	Down	1.88E-73	1.22E-72
Ykt6	597	1622	598.5	97.26621	33.47145	-1.53901	Down	########	########
Efna3	1696	73	27	1.546622	0.533104	-1.53663	Down	3.18E-06	6.58E-06
8-Sep	4008	5928	2190.5	52.92476	18.2464	-1.53633	Down	0	0
Nlgn2	3993	998	370.5	8.965253	3.091451	-1.53606	Down	5.15E-67	3.23E-66
Apeh	2381	519	193	7.813842	2.694563	-1.53598	Down	1.63E-35	8.47E-35
Scarb2	2070	2819.5	1043.5	48.76349	16.81724	-1.53586	Down	########	########
Cpd	4377	4088.5	1511.5	33.39813	11.52662	-1.5348	Down	########	########
LOC686590	4465	6128	2270.5	49.22676	16.99418	-1.5344	Down	0	0

Cobra1	2545	2540	938.5	35.66899	12.31622	-1.53411	Down	########	########
Klhl5	3421	670.5	248	7.017956	2.424534	-1.53334	Down	1.14E-45	6.35E-45
Megf8	11587	4774	1770.5	14.7456	5.094261	-1.53334	Down	0	0
Col16a1	5241	3873.5	1436	26.46641	9.143648	-1.53332	Down	########	########
Lepre1	2524	2555	946.5	36.25022	12.52487	-1.53319	Down	########	########
Zbtb2	3068	496.5	184	5.800491	2.004685	-1.5328	Down	3.92E-34	2.02E-33
Serpinf1	1434	1148	426.5	28.66454	9.90699	-1.53275	Down	1.03E-76	6.79E-76
Chaf1b	1958	768.5	285	14.0609	4.861053	-1.53235	Down	6.65E-52	3.84E-51
A2ld1	1179	107.5	40	3.258058	1.126609	-1.53203	Down	2.07E-08	4.75E-08
Stard8	4777	105	39	0.787127	0.272197	-1.53195	Down	2.40E-08	5.49E-08
Copg1	2625	7341	2722.5	100.0472	34.60306	-1.53171	Down	0	0
Arf1	900	11927.5	4435	475.5273	164.4958	-1.53148	Down	0	0
Cdk5rap2	5654	2765	1026	17.50694	6.057724	-1.53108	Down	########	########
Cbx3	1745	3915	1443.5	79.78208	27.61645	-1.53054	Down	########	########
Atp1a1	3636	48639	18042.5	478.4544	165.6481	-1.53026	Down	0	0
Adamts4	3339	1036	384.5	11.11355	3.848002	-1.53014	Down	1.60E-69	1.02E-68
Ergic1	2828	4220.5	1568.5	53.41649	18.50642	-1.52926	Down	########	########
Ctdspl	4123	1038	384	8.99218	3.115847	-1.52905	Down	7.51E-70	4.78E-69
Ap1s1	1302	5659	2100.5	155.6851	53.94789	-1.52899	Down	0	0
Gcn1l1	8538	8165.5	3033	34.2049	11.86071	-1.52801	Down	0	0
Vars	3980	8469.5	3152	76.25006	26.44056	-1.52799	Down	0	0
Pald	4213	688	256	5.841233	2.027017	-1.52691	Down	1.71E-46	9.60E-46
Dnm1	3221	1913	711	21.257	7.382063	-1.52584	Down	########	########
LOC686310	3904	1391.5	519	12.76543	4.434082	-1.52553	Down	8.29E-92	5.75E-91
Pcdha5	5385	37.5	14	0.248814	0.086465	-1.52488	Down	0.001195	0.002017

Abcc1	4981	1993	742	14.28813	4.972779	-1.52269	Down	########	########
RGD1565043	5460	3118.5	1164.5	20.45931	7.130493	-1.52068	Down	########	########
Pou3f2	1814	116	43.5	2.288865	0.798388	-1.51947	Down	4.19E-09	9.86E-09
LOC365077	862	460.5	174	19.22631	6.70801	-1.51913	Down	6.93E-31	3.47E-30
Stat2	3464	1284.5	481	13.26918	4.633388	-1.51794	Down	2.22E-84	1.51E-83
Phyh	1198	2307.5	864	69.04822	24.11395	-1.51774	Down	########	########
Ssc5d	4495	573.5	215.5	4.580054	1.599935	-1.51735	Down	1.58E-38	8.41E-38
Smarcc1	3670	5316	1991	51.84597	18.11478	-1.51706	Down	0	0
Ankrd27	4280	1754.5	658	14.67471	5.127996	-1.51687	Down	########	########
Ep400	10610	4948	1857	16.69295	5.84297	-1.51447	Down	0	0
Ptk2b	3907	400.5	150.5	3.673877	1.286656	-1.51368	Down	2.07E-27	1.01E-26
Ccdc102a	2299	1792.5	675.5	27.98415	9.802584	-1.51338	Down	########	########
Ralbp1	3622	5017.5	1887	49.57922	17.37482	-1.51274	Down	0	0
Nit2	1054	541	203	18.35223	6.432849	-1.51243	Down	1.82E-36	9.52E-36
Itpripl2	1926	1088	409.5	20.24824	7.098831	-1.51214	Down	2.11E-71	1.35E-70
LOC100911603	1058	143	53	4.814806	1.688659	-1.5116	Down	6.57E-11	1.64E-10
Nomo1	4201	7859.5	2953.5	66.88394	23.46018	-1.51145	Down	0	0
Acot11	2052	53.5	20	0.932526	0.327162	-1.51114	Down	9.61E-05	0.000179
Mlec	1788	5684	2142	113.818	39.93504	-1.511	Down	0	0
Rnase12	438	29	11	2.388848	0.838482	-1.51046	Down	0.004324	0.006968
B3galtl-ps1	3394	1284.5	484	13.55265	4.7574	-1.51033	Down	1.37E-83	9.27E-83
Zmym3	5473	1227.5	463	8.029809	2.820646	-1.50934	Down	7.81E-80	5.22E-79
Dnaaf3	2160	142.5	53.5	2.358177	0.828817	-1.50855	Down	9.64E-11	2.40E-10
Rpl13a	690	12110.5	4552	626.2496	220.1362	-1.50834	Down	0	0
Flnb	8861	9390.5	3536	37.89403	13.32084	-1.50829	Down	0	0

Mpzl1	2291	2864.5	1082.5	44.79419	15.76568	-1.50652	Down	########	########
LOC100911372	1082	17690.5	6669.5	584.527	205.7484	-1.50639	Down	0	0
Hdac5	3754	2650.5	1005	25.31833	8.914875	-1.5059	Down	########	########
Arsa	1524	654	247.5	15.3874	5.418823	-1.5057	Down	2.58E-43	1.42E-42
Selp	3185	67	25	0.75292	0.265171	-1.50557	Down	9.38E-06	1.88E-05
Papd7	3819	2753.5	1040	25.79139	9.085384	-1.50527	Down	########	########
Lama4	6057	5310	2004	31.35594	11.04878	-1.50485	Down	0	0
Tcf19	1616	2201.5	832.5	48.79219	17.20821	-1.50355	Down	########	########
LOC100911261	4389	3224.5	1217.5	26.27157	9.267691	-1.50322	Down	########	########
LOC100911205	3951	496	188	4.496939	1.586819	-1.50281	Down	4.47E-33	2.28E-32
Adamts7	5664	3329	1261	21.05468	7.433522	-1.50202	Down	########	########
Wdr46	2276	1128.5	429	17.76884	6.280378	-1.50043	Down	1.15E-72	7.46E-72
Marveld1	2772	1625	616.5	21.01696	7.434567	-1.49923	Down	########	########
Dock1	6785	5843.5	2214.5	30.79612	10.89811	-1.49867	Down	0	0
Xbp1	1851	2026.5	769	39.1374	13.86038	-1.49758	Down	########	########
RGD1561672	6050	131	50	0.775664	0.274734	-1.4974	Down	1.01E-09	2.42E-09
Dzip1	3778	3043.5	1155.5	28.83349	10.21313	-1.49732	Down	########	########
Dnajc10	4159	7923	2993	67.86256	24.04891	-1.49664	Down	0	0
Cdh13	3403	2066.5	785.5	21.77676	7.72095	-1.49594	Down	########	########
Cyb561d2	1500	225.5	85	5.366988	1.90392	-1.49514	Down	6.40E-16	2.73E-15
LOC100911825	1400	80	30.5	2.043118	0.725105	-1.49451	Down	1.37E-06	2.90E-06
Atf6b	2507	4072	1553.5	58.27928	20.6942	-1.49376	Down	########	########
Phf2	4931	2131	814.5	15.49853	5.511439	-1.49163	Down	########	########
Rsu1	1582	4151.5	1585	94.12727	33.48426	-1.49113	Down	########	########
Nelf	2848	1626.5	622	20.44848	7.279001	-1.49018	Down	########	########

LOC684558	3945	4544.5	1741	41.33825	14.71687	-1.49001	Down	########	########
Sdc1	3021	13263	5066	157.3818	56.03793	-1.48979	Down	0	0
Plekhg2	5011	808	309.5	5.788644	2.061299	-1.48967	Down	4.11E-52	2.38E-51
Rpusd1	1747	483.5	185.5	9.946209	3.543276	-1.48906	Down	9.44E-32	4.75E-31
Ruvbl2	1504	3405	1304.5	81.12362	28.92137	-1.48799	Down	########	########
Tnfrsf11b	2832	160	61	2.017399	0.719455	-1.48752	Down	1.37E-11	3.58E-11
Pcsk6	4153	3584	1369.5	30.88612	11.0163	-1.48732	Down	########	########
Kpna2	1977	6562	2505.5	118.6622	42.32853	-1.48716	Down	0	0
Radil	3687	99.5	38	0.967927	0.345565	-1.48594	Down	1.29E-07	2.86E-07
Smarca4	5568	9491.5	3642	61.10933	21.82246	-1.48558	Down	0	0
Pdxk	939	266	102	10.13973	3.62189	-1.4852	Down	3.72E-18	1.63E-17
Uqcrc1	1595	6273.5	2406	140.8862	50.34418	-1.48463	Down	0	0
Xkr5	2196	546.5	209	8.883528	3.176295	-1.48379	Down	1.03E-35	5.38E-35
Mlxip	2754	660	254	8.608401	3.077941	-1.48378	Down	1.80E-42	9.85E-42
Fbln1	2787	2326.5	892.5	29.90045	10.6912	-1.48374	Down	########	########
Pcif1	3977	1277	490.5	11.49275	4.109701	-1.48362	Down	6.63E-81	4.45E-80
Bicc1	3226	2617	1006	29.08927	10.40302	-1.48349	Down	########	########
Adamts9	7965	897	344	4.025866	1.44002	-1.48321	Down	2.06E-57	1.23E-56
LOC681468	252	60	23	8.544848	3.057924	-1.4825	Down	4.08E-05	7.87E-05
LOC100909635	588	340.5	130.5	20.6078	7.376919	-1.4821	Down	7.02E-23	3.25E-22
Fsd1	841	83	32	3.548566	1.270366	-1.48199	Down	1.48E-06	3.13E-06
Lgi4	2630	360	139	4.899611	1.754626	-1.4815	Down	8.72E-24	4.08E-23
Fbxo31	1775	980	376.5	19.77447	7.084555	-1.48089	Down	1.62E-62	9.95E-62
Mical3	6367	1466.5	565	8.253463	2.958465	-1.48015	Down	5.69E-92	3.95E-91
Galns	1575	408	158	9.290678	3.334422	-1.47835	Down	1.25E-26	6.05E-26

Slc27a1	3210	866	334	9.662602	3.468004	-1.47831	Down	5.26E-55	3.10E-54
Car3	802	218.5	84	9.748566	3.499118	-1.4782	Down	4.84E-15	2.03E-14
LOC498276	1427	84.5	32.5	2.123932	0.762594	-1.47775	Down	1.01E-06	2.15E-06
siat7D	3237	4074	1571	45.11021	16.21099	-1.47648	Down	########	########
Gas2l3	2562	1235.5	475	17.23676	6.199819	-1.47519	Down	5.11E-78	3.38E-77
Trappc9	4122	1063.5	410.5	9.240699	3.325676	-1.47435	Down	4.14E-67	2.60E-66
Dnajc22	1371	232	89.5	6.043093	2.175251	-1.47411	Down	5.14E-16	2.19E-15
Blvrb	884	1237	480	50.18576	18.06953	-1.47372	Down	4.58E-77	3.02E-76
Acy1	1356	1701	659	44.98295	16.21232	-1.47229	Down	########	########
Amdhd2	1497	288	111	6.90177	2.4876	-1.47221	Down	2.18E-19	9.70E-19
Atxn7l3	3698	2881.5	1115	27.87216	10.05784	-1.4705	Down	########	########
Gipc1	1607	3704	1437	82.60295	29.83027	-1.46942	Down	########	########
Irf2bp1	2702	1032	401	13.69122	4.945563	-1.46904	Down	1.87E-64	1.16E-63
Map6	3581	2717.5	1055.5	27.22074	9.843153	-1.46751	Down	########	########
Dnajc9	1613	2745.5	1063.5	60.77947	21.97863	-1.46748	Down	########	########
Pam	3770	5960.5	2310	56.56745	20.46672	-1.46669	Down	0	0
Hdac7	4092	1827.5	710.5	16.00942	5.798211	-1.46524	Down	########	########
Gfra1	3616	507.5	197.5	5.021883	1.818928	-1.46514	Down	1.57E-32	7.97E-32
Tsc22d4	1665	1156	449.5	24.92606	9.034248	-1.46418	Down	4.98E-72	3.21E-71
Rps6ka2	5396	1381.5	537.5	9.159789	3.320267	-1.46402	Down	1.54E-85	1.05E-84
Herc3	5230	1074	417	7.328704	2.657017	-1.46375	Down	4.37E-67	2.75E-66
LOC100911422	1126	1623.5	630.5	51.56251	18.69603	-1.46359	Down	########	1.25E-99
Htatsf1	2911	2264	880	27.76996	10.07372	-1.46293	Down	########	########
Unc45a	3335	2634	1024.5	28.24476	10.24916	-1.46248	Down	########	########
Mlst8	3601	570	222.5	5.663309	2.055544	-1.46212	Down	3.09E-36	1.61E-35

Psmb9	913	801	311.5	31.40932	11.41023	-1.46086	Down	1.76E-50	1.01E-49
Troap	2256	1126	441	17.91683	6.516438	-1.45916	Down	2.74E-69	1.74E-68
LOC682402	5307	6542.5	2556	44.15024	16.05933	-1.45901	Down	0	0
LOC100912455	3153	216	84.5	2.454646	0.893192	-1.45847	Down	1.01E-14	4.22E-14
Zbtb45	2197	459.5	180	7.51208	2.73374	-1.45834	Down	3.45E-29	1.70E-28
Atp5b	1759	31015	12108	630.7942	229.6473	-1.45775	Down	0	0
Rrp1b	4414	1584.5	619	12.8473	4.679573	-1.45702	Down	4.54E-97	3.20E-96
Gpr77	1312	358	139.5	9.756155	3.553666	-1.457	Down	1.82E-23	8.48E-23
Ablim1	6488	798.5	311	4.397467	1.602188	-1.45663	Down	5.27E-50	3.01E-49
Acadsb	1321	67	26.5	1.824011	0.664648	-1.45645	Down	1.73E-05	3.42E-05
Rab38	1402	232	91	5.903337	2.152924	-1.45523	Down	1.66E-15	7.04E-15
Lgals3bp	2151	7888.5	3089	131.6067	47.99998	-1.45513	Down	0	0
Nans	2039	5617	2196.5	98.60368	35.98935	-1.45407	Down	0	0
LOC100125385	1483	316	124	7.641762	2.789187	-1.45406	Down	1.45E-20	6.57E-20
Srgap2	3559	2531	993.5	25.4798	9.310493	-1.45242	Down	########	########
Fanca	4611	1184	464	9.189689	3.359679	-1.45169	Down	1.08E-72	6.99E-72
Dhx37	4177	1329	523	11.41127	4.173235	-1.45122	Down	7.88E-81	5.28E-80
Anapc5	2384	14565	5710	218.4589	79.90247	-1.45105	Down	0	0
Ednra	4595	595	233.5	4.6304	1.694139	-1.45058	Down	1.95E-37	1.03E-36
Pold1	3321	1445	567.5	15.58518	5.703181	-1.45034	Down	3.91E-88	2.68E-87
LOC100909992	822	239.5	94	10.39973	3.805912	-1.45023	Down	7.27E-16	3.09E-15
Parvb	1997	53	21	0.952265	0.348835	-1.44882	Down	0.000176	0.000321
Slk	3796	5736	2249.5	54.03119	19.8045	-1.44796	Down	0	0
Dysf	6319	117	46	0.662275	0.242759	-1.44791	Down	1.76E-08	4.04E-08
Ptgs1	2794	10322.5	4053.5	132.1713	48.4486	-1.44788	Down	0	0

R3hdm4	1803	1968.5	773	39.08771	14.3304	-1.44764	Down	########	########
Hnrnpul1	3746	12676	4992	121.2784	44.47588	-1.44723	Down	0	0
Socs1	639	217	86	12.1993	4.476472	-1.44636	Down	2.24E-14	9.16E-14
RGD1565590	1299	343.5	134.5	9.428539	3.460721	-1.44596	Down	2.44E-22	1.12E-21
LOC688400	3307	378.5	148	4.079155	1.497444	-1.44577	Down	2.23E-24	1.05E-23
Akt2	1984	2733.5	1080	49.4695	18.16066	-1.44572	Down	########	########
Mfsd3	1531	239.5	94.5	5.60276	2.056959	-1.44563	Down	7.27E-16	3.09E-15
Rmnd5a	5671	3107.5	1217.5	19.54718	7.177058	-1.4455	Down	########	########
Hmx2	1497	234.5	93	5.614325	2.06391	-1.44373	Down	2.53E-15	1.07E-14
LOC679082	2551	301	118.5	4.22082	1.552484	-1.44294	Down	1.09E-19	4.88E-19
Tnfaip1	3885	4332	1712	39.91337	14.69517	-1.44153	Down	########	########
Tmem169	2942	133	52.5	1.615911	0.595023	-1.44133	Down	1.57E-09	3.74E-09
Clip1	4597	2492.5	983	19.38918	7.14358	-1.44053	Down	########	########
Glg1	5292	8648	3409	58.41307	21.52276	-1.44043	Down	0	0
Fbln2	4520	411.5	163	3.256875	1.20021	-1.4402	Down	7.53E-26	3.61E-25
Sec23a	2510	3405	1343.5	48.48091	17.86611	-1.44019	Down	########	########
Anxa11	2363	2473	980.5	37.48999	13.82091	-1.43965	Down	########	########
RGD1564019	3141	84	33	0.955128	0.352202	-1.43929	Down	1.84E-06	3.86E-06
Ppm1g	2182	5974	2362	97.94776	36.12078	-1.43918	Down	0	0
Lhpp	1687	480.5	190.5	10.20845	3.765049	-1.43902	Down	5.20E-30	2.58E-29
LOC686349	1188	273	107.5	8.213744	3.031341	-1.43808	Down	5.28E-18	2.32E-17
Ermp1	5095	3224.5	1274.5	22.58797	8.340577	-1.43734	Down	########	########
Polg	3651	2579.5	1023	25.29338	9.341333	-1.43706	Down	########	########
Dennd1a	4114	1765	701.5	15.39768	5.686946	-1.43699	Down	########	########
Zdhhc12	1145	525.5	209	16.44157	6.078465	-1.43557	Down	2.23E-32	1.13E-31

LOC304396	2564	1015	402.5	14.18146	5.247247	-1.43437	Down	1.60E-61	9.77E-61
Lima1	4204	5076.5	2018.5	43.27074	16.03381	-1.43227	Down	########	########
RGD1306730	2504	1945.5	773	27.79848	10.30168	-1.43213	Down	########	########
Tex261	1111	3201.5	1272.5	103.0945	38.22022	-1.43156	Down	########	########
Qser1	5635	3340.5	1325	21.17658	7.851451	-1.43144	Down	########	########
Cntnap1	5379	91.5	36	0.607148	0.225121	-1.43135	Down	7.88E-07	1.69E-06
Galnt12	2277	103	41	1.618905	0.600773	-1.43013	Down	1.70E-07	3.74E-07
Cd99	686	2099.5	834.5	109.412	40.61479	-1.42969	Down	########	########
Plxna2	10201	1469.5	586.5	5.15274	1.913863	-1.42885	Down	3.34E-87	2.29E-86
Spryd3	2414	1863.5	742.5	27.6512	10.27045	-1.42884	Down	########	########
Actr1a	2767	7648.5	3048.5	98.83884	36.74649	-1.42747	Down	0	0
LOC684111	1329	117	46.5	3.154532	1.173241	-1.42693	Down	1.76E-08	4.04E-08
Mxra7	647	128.5	51.5	7.1082	2.644313	-1.42659	Down	5.53E-09	1.30E-08
Prtfdc1	1099	1092	436.5	35.53288	13.22001	-1.42643	Down	3.40E-65	2.11E-64
Isoc1	3315	1600	638	17.23046	6.411458	-1.42624	Down	7.10E-95	4.96E-94
LOC100909582	1561	77	31	1.773909	0.660148	-1.42607	Down	7.73E-06	1.56E-05
Rnd2	1387	168	67	4.338369	1.61472	-1.42587	Down	2.39E-11	6.12E-11
Actn2	2943	151.5	60.5	1.842734	0.685882	-1.42581	Down	2.15E-10	5.28E-10
Afg3l1	2504	1969	784	28.1335	10.47459	-1.4254	Down	########	########
Fam114a1	3224	3854.5	1537.5	42.74977	15.91842	-1.42522	Down	########	########
LOC688280	2828	1343	534.5	17.00034	6.331048	-1.42505	Down	2.63E-80	1.76E-79
Fto	1571	1368.5	548	31.24656	11.64148	-1.42442	Down	8.93E-81	5.99E-80
Plekhg3	4379	1306.5	522	10.70305	3.987901	-1.42432	Down	1.76E-77	1.16E-76
Tnfrsf19	1385	147	58	3.80083	1.416344	-1.42414	Down	2.94E-10	7.18E-10
Cant1	2701	1279.5	511	16.97764	6.32842	-1.42372	Down	5.74E-76	3.76E-75

Uba7	3063	94.5	38	1.104643	0.411851	-1.42339	Down	8.25E-07	1.76E-06
Paqr8	1630	424.5	169.5	9.340995	3.483138	-1.42319	Down	2.08E-26	1.00E-25
Mta1	2741	5745	2302	75.139	28.02085	-1.42306	Down	0	0
Wee1	3305	1214.5	484	13.10402	4.890141	-1.42206	Down	1.89E-72	1.22E-71
LOC100362124	4903	944.5	378	6.899672	2.57508	-1.42191	Down	2.47E-56	1.47E-55
Mex3d	2734	2142.5	860	28.13456	10.50536	-1.42122	Down	########	########
Rbpms2	1903	68	27	1.277439	0.477007	-1.42117	Down	2.15E-05	4.23E-05
Stac2	2880	733.5	294.5	9.137137	3.412315	-1.42099	Down	5.21E-44	2.87E-43
Crabp1	755	301	121	14.27957	5.332853	-1.42097	Down	6.02E-19	2.67E-18
Pdcd4	1410	473	188.5	11.97012	4.471393	-1.42064	Down	2.09E-29	1.03E-28
LOC100911896	927	162.5	65	6.25567	2.338126	-1.41981	Down	6.78E-11	1.70E-10
Fkrp	2860	1006.5	404	12.6143	4.71555	-1.41956	Down	1.24E-59	7.52E-59
Phf15	2712	2209.5	887	29.19438	10.91766	-1.41903	Down	########	########
Scarb1	2497	3073.5	1234.5	44.12181	16.50784	-1.41834	Down	########	########
Fbxl19	2421	1223.5	492.5	18.12936	6.784202	-1.41808	Down	6.68E-72	4.30E-71
Akap2	3467	3167.5	1273	32.74954	12.26533	-1.41689	Down	########	########
Iqce	2487	577.5	231.5	8.321239	3.117369	-1.41647	Down	4.18E-35	2.17E-34
RGD1560813	694	500.5	201	25.80166	9.668371	-1.41612	Down	1.94E-30	9.69E-30
Fam38a	8221	11130	4479.5	48.52144	18.18666	-1.41574	Down	0	0
Sec31a	4422	10424.5	4199	84.51792	31.6956	-1.41497	Down	0	0
Nrbp	2152	1993	801.5	33.17004	12.44288	-1.41456	Down	########	########
Lmbr1l	1055	104	42	3.52636	1.323171	-1.41418	Down	2.08E-07	4.59E-07
Ube3c	5056	6484	2602.5	45.79707	17.18847	-1.41381	Down	0	0
Trabd	2397	1299	523	19.42182	7.293285	-1.41304	Down	3.74E-76	2.45E-75
Pomgnt1	2745	3326	1340.5	43.39316	16.29518	-1.41302	Down	########	########

LOC100911693	1761	1756	707	35.70866	13.41373	-1.41256	Down	########	########
Ndrg2	2042	877.5	353	15.40801	5.788318	-1.41246	Down	5.14E-52	2.97E-51
C1qtnf5	1364	918.5	370	24.13696	9.068417	-1.41232	Down	3.02E-54	1.77E-53
Fhod3	5330	448.5	181	3.012481	1.132254	-1.41175	Down	3.20E-27	1.55E-26
Kcnmb3	1637	43	17.5	0.945893	0.35554	-1.41167	Down	0.00073	0.001254
Lpcat1	3621	1535.5	617.5	15.15336	5.697654	-1.4112	Down	6.70E-90	4.62E-89
Utrn	10705	5693	2291	19.00826	7.147874	-1.41104	Down	0	0
Tagln2	1325	19976.5	8071.5	540.3887	203.3168	-1.41027	Down	0	0
LOC100910195	3927	611.5	247	5.569642	2.095605	-1.41022	Down	1.70E-36	8.89E-36
Tgfb1i1	1972	1260.5	511.5	22.96056	8.640975	-1.40989	Down	4.94E-73	3.20E-72
Shmt2	2075	3682.5	1488	63.56595	23.93112	-1.40937	Down	########	########
LOC690137	1807	161.5	65	3.193195	1.202459	-1.40901	Down	9.74E-11	2.42E-10
Alms1	9965	1289.5	519.5	4.622485	1.741485	-1.40835	Down	1.42E-75	9.27E-75
Chd3	7343	7242.5	2925.5	35.31024	13.30527	-1.40809	Down	0	0
LOC100911770	574	57	23	3.554386	1.339367	-1.40805	Down	0.000127	0.000232
Atp8a1	5335	712	287.5	4.773849	1.799449	-1.4076	Down	1.88E-42	1.03E-41
RGD1565149	2697	419	170	5.562819	2.097463	-1.40717	Down	2.17E-25	1.03E-24
RGD1562618	1548	2702.5	1093	62.49615	23.56943	-1.40685	Down	########	########
Tmem184b	3324	3175.5	1288	34.2663	12.92443	-1.40669	Down	########	########
Msh2	3002	2211.5	894	26.32023	9.935146	-1.40556	Down	########	########
Tmem161a	2159	564	229	9.367116	3.53584	-1.40555	Down	1.41E-33	7.24E-33
Kat2a	3059	2152	871	25.2113	9.516938	-1.4055	Down	########	########
Phgdh	1803	3325	1347.5	66.08756	24.94841	-1.40543	Down	########	########
Slc29a3	1831	684	277	13.40218	5.061686	-1.40478	Down	1.54E-40	8.28E-40
RGD1309821	2332	270	110	4.151851	1.568674	-1.40421	Down	8.50E-17	3.67E-16

C1r	2797	5752	2333.5	73.70694	27.85015	-1.40412	Down	0	0
Gcs1	2702	759.5	307.5	10.05134	3.798407	-1.40392	Down	6.89E-45	3.81E-44
Trpv2	2811	59.5	24	0.755888	0.285693	-1.40371	Down	0.000107	0.000199
9-Sep	2866	12588.5	5110	157.5174	59.53877	-1.40361	Down	0	0
Unc93b1	2270	543	220	8.563896	3.237312	-1.40347	Down	1.68E-32	8.52E-32
Ctbp1	2430	6484.5	2631	95.5327	36.14288	-1.40228	Down	0	0
Il33	2471	36279	14688	524.5777	198.5583	-1.40159	Down	0	0
Ghrhr	1629	71	29	1.565597	0.592706	-1.40133	Down	2.24E-05	4.40E-05
RGD1306926	3691	430	174.5	4.173704	1.580506	-1.40094	Down	3.99E-26	1.92E-25
Fn1	8331	206812	84012.5	889.1291	336.7281	-1.40081	Down	0	0
Slc36a4	2973	322	130.5	3.870214	1.46597	-1.40056	Down	5.01E-20	2.25E-19
Mfge8	2020	64414	26194	1142.544	432.9208	-1.40007	Down	0	0
Myo18a	6951	5673	2308.5	29.23116	11.08209	-1.39928	Down	0	0
Ccni	2671	8999.5	3648.5	120.1865	45.56572	-1.39925	Down	0	0
Mmgt2	2093	317	128.5	5.402917	2.049573	-1.39842	Down	9.80E-20	4.39E-19
Pccb	1833	1847.5	754	36.1309	13.71198	-1.3978	Down	########	########
Lrrc20	3034	463.5	189	5.464382	2.074755	-1.39712	Down	1.30E-27	6.31E-27
LOC100909794	702	154	62	7.809857	2.96536	-1.39709	Down	2.25E-10	5.53E-10
Arhgef25	2118	648	263	10.95218	4.158514	-1.39708	Down	2.35E-38	1.25E-37
Ier5l	1612	2352.5	964	52.41777	19.91221	-1.3964	Down	########	########
Pigy	1135	155	63.5	4.896296	1.860363	-1.39611	Down	2.75E-10	6.72E-10
LOC686774	5587	3309	1346	21.16626	8.042948	-1.39597	Down	########	########
Ccnd3	1843	4124.5	1684.5	80.16724	30.4718	-1.39554	Down	########	########
Cdca3	1524	3742	1527.5	87.99277	33.45138	-1.39532	Down	########	########
Med12	6792	2438	994.5	12.85518	4.888374	-1.39492	Down	########	########

Esd	1072	2381	969.5	79.18276	30.11349	-1.39478	Down	########	########
Ccnf	3313	2250	917.5	24.32131	9.254407	-1.39401	Down	########	########
Pabpc4	2611	11853	4836	162.4902	61.82923	-1.39399	Down	0	0
S1pr2	2754	1046	426	13.5788	5.167023	-1.39395	Down	1.66E-60	1.01E-59
Tapbpl	1886	1353.5	552	25.71062	9.78368	-1.39392	Down	1.66E-77	1.10E-76
LOC100909406	831	345.5	142.5	14.95916	5.693225	-1.39371	Down	1.07E-20	4.83E-20
Plp2	1098	5744.5	2345	187.3457	71.32721	-1.39318	Down	0	0
Anpep	3320	33	13.5	0.355504	0.135353	-1.39314	Down	0.003088	0.005043
Acly	4331	11812	4822	97.66906	37.18617	-1.39314	Down	0	0
Adam9	3095	3125	1277.5	36.15065	13.78969	-1.39043	Down	########	########
Ppp1r14b	745	4878.5	1995	234.192	89.382	-1.38964	Down	########	########
Ado	1169	463.5	190.5	14.24592	5.437243	-1.3896	Down	2.24E-27	1.09E-26
Dusp15	3164	44	18	0.497618	0.189938	-1.38951	Down	0.000898	0.001532
Adarb1	6382	1254	514.5	7.042284	2.688102	-1.38946	Down	2.11E-71	1.36E-70
Naa10	938	578.5	237	22.04167	8.415537	-1.38911	Down	8.10E-34	4.16E-33
Mcm6	2929	6852	2801.5	83.69869	31.9619	-1.38885	Down	0	0
Sqle	2085	7685	3137.5	131.5226	50.23366	-1.38858	Down	0	0
Acap3	4440	1443.5	592	11.65754	4.452588	-1.38855	Down	9.20E-82	6.19E-81
Bicd2	4586	6068.5	2483	47.33856	18.08959	-1.38786	Down	0	0
Ltbp3	4389	9654	3963.5	78.91605	30.15693	-1.38783	Down	0	0
Rabif	1804	268	109.5	5.315913	2.032265	-1.38723	Down	9.97E-17	4.30E-16
Acsbg1	2689	213	87.5	2.832528	1.082892	-1.3872	Down	1.66E-13	6.12E-13
B4galt7	1591	605.5	248	13.62283	5.209321	-1.38686	Down	2.45E-35	1.27E-34
Josd1	2724	1244.5	509.5	16.34674	6.25115	-1.38681	Down	4.64E-71	2.97E-70
Xpo5	4927	3512.5	1437.5	25.49312	9.750516	-1.38656	Down	########	########

Pygb	3873	3658	1500	33.80955	12.93153	-1.38654	Down	########	########
Kif26a	5779	134	55.5	0.833392	0.318847	-1.38613	Down	6.00E-09	1.40E-08
Cep250	7614	1902	781	8.947043	3.424626	-1.38546	Down	########	########
Arl4c-ps1	3440	12679	5199	131.8451	50.46684	-1.38544	Down	0	0
Plekhb1	1903	433.5	178.5	8.168985	3.130476	-1.38377	Down	1.24E-25	5.93E-25
Tbc1d1	5313	7709.5	3168.5	51.89584	19.89514	-1.3832	Down	0	0
LOC685152	5490	6978	2870	45.50745	17.45227	-1.38269	Down	0	0
Cabin1	7260	2683.5	1106	13.26002	5.08682	-1.38225	Down	########	########
Tgfb3	2619	5090.5	2093.5	69.64413	26.71785	-1.3822	Down	########	########
Lrrk2	7581	1338	548.5	6.307654	2.420646	-1.38171	Down	3.65E-76	2.39E-75
Msn	2099	21470	8819.5	365.6582	140.4385	-1.38056	Down	0	0
Hibch	1738	215	88.5	4.420311	1.697746	-1.38053	Down	1.41E-13	5.30E-13
Sfxn1	2178	2104.5	865	34.51841	13.26588	-1.37965	Down	########	########
PCOLCE2	1871	8234.5	3393.5	157.5659	60.59022	-1.3788	Down	0	0
Padi2	4507	103.5	42	0.819255	0.31512	-1.37841	Down	2.99E-07	6.53E-07
Tbc1d25	2378	331	137	4.996435	1.922327	-1.37805	Down	9.66E-20	4.32E-19
Tap2	2446	1044	430	15.27251	5.879244	-1.37723	Down	2.95E-59	1.78E-58
Podnl1	2119	63	26	1.064142	0.409654	-1.37721	Down	7.66E-05	0.000144
RGD1308023	6002	1281	528	7.634404	2.93901	-1.37719	Down	3.28E-72	2.12E-71
LOC691153	1521	308	128	7.275453	2.801388	-1.3769	Down	2.36E-18	1.04E-17
RGD1563349	5912	170	70.5	1.028562	0.396079	-1.37677	Down	6.24E-11	1.56E-10
LOC100909750	478	2513.5	1040	188.4499	72.59934	-1.37615	Down	########	########
Agap3	4337	2000.5	828	16.54338	6.374678	-1.37583	Down	########	########
LOC100910979	2575	51	21	0.707158	0.27263	-1.37509	Down	0.000371	0.000662
LOC100359616	318	1074	449	121.7256	46.93672	-1.37484	Down	2.30E-59	1.39E-58

Ccdc85c	763	243.5	101	11.49146	4.434821	-1.37361	Down	8.61E-15	3.59E-14
Klhl23	3697	561	232	5.419522	2.092221	-1.37313	Down	2.08E-32	1.05E-31
Suox	2184	306	126.5	5.012272	1.936072	-1.37233	Down	1.61E-18	7.11E-18
Zfp64	2419	814	338	12.07221	4.665416	-1.37161	Down	6.00E-46	3.35E-45
Syt11	1812	1634	679	32.31747	12.49445	-1.37103	Down	2.55E-90	1.76E-89
LOC100364403	698	272.5	113	13.95872	5.397292	-1.37086	Down	2.16E-16	9.24E-16
Gnb1	1544	11198	4638	259.2322	100.2669	-1.3704	Down	0	0
Otx1	1229	71	29.5	2.079815	0.804688	-1.36995	Down	2.24E-05	4.40E-05
Parp9	3179	700	290	7.869549	3.045101	-1.36979	Down	6.62E-40	3.55E-39
Shisa5	1479	3440.5	1431.5	83.43035	32.28686	-1.36963	Down	########	########
Stim1	3839	2197.5	914	20.49203	7.937808	-1.36825	Down	########	########
Atm	9756	1187.5	492.5	4.338048	1.682334	-1.36658	Down	2.10E-66	1.31E-65
Emp2	3154	535.5	222.5	6.072617	2.355427	-1.36633	Down	8.32E-31	4.16E-30
Dnase2a	1433	1039.5	432	25.98381	10.08193	-1.36584	Down	4.92E-58	2.96E-57
Prps2	2697	1228.5	510	16.23601	6.301065	-1.36553	Down	2.11E-68	1.34E-67
LOC100912571	2076	2221.5	923	38.26478	14.85175	-1.36538	Down	########	########
Ckb	1463	7381	3083	181.1086	70.29558	-1.36535	Down	0	0
Npepl1	1882	964	403	18.37524	7.133945	-1.36499	Down	1.81E-53	1.06E-52
Sidt2	4741	2103	878	15.89222	6.171993	-1.36451	Down	########	########
Ube2e2	914	939.5	391.5	36.8283	14.30819	-1.36397	Down	1.75E-52	1.01E-51
Exosc5	1051	641.5	267.5	21.89207	8.50573	-1.3639	Down	2.39E-36	1.25E-35
Kcnd1	3712	446.5	186	4.30691	1.673423	-1.36385	Down	9.85E-26	4.71E-25
Fbn1	8942	16901.5	7025	67.47245	26.22658	-1.36327	Down	0	0
Aldh1a3	1539	1858	774	43.1947	16.7922	-1.36306	Down	########	########
Nono	2438	13598	5662.5	199.4691	77.56398	-1.36271	Down	0	0

Ndufa11	538	1834.5	766	122.1482	47.50576	-1.36246	Down	########	2.14E-99
Ndp	1925	178.5	74.5	3.318231	1.290944	-1.36199	Down	3.26E-11	8.25E-11
Hdgfrp2	2157	3262	1358.5	54.12563	21.06469	-1.36149	Down	########	########
Tm7sf2	1510	446.5	186.5	10.58644	4.122709	-1.36055	Down	9.85E-26	4.71E-25
Comtd1	2308	251.5	105	3.90584	1.521237	-1.36039	Down	4.50E-15	1.89E-14
RGD1304827	1743	490	206	10.09573	3.933495	-1.35986	Down	9.83E-28	4.79E-27
Dact3	2803	258	108.5	3.299778	1.286093	-1.35937	Down	1.95E-15	8.26E-15
Sntb1	2502	331	138.5	4.738268	1.846892	-1.35926	Down	1.65E-19	7.37E-19
LOC100911730	2148	5583.5	2331.5	92.92518	36.23291	-1.35877	Down	########	########
LOC500046	4838	1232	513	9.098594	3.548375	-1.35849	Down	2.62E-68	1.66E-67
Mfhas1	3177	1015	425.5	11.46249	4.471117	-1.35821	Down	4.78E-56	2.83E-55
Foxk1	2487	505	212	7.301274	2.848896	-1.35775	Down	1.33E-28	6.50E-28
Slc9a3r2	2110	861.5	361.5	14.64418	5.718996	-1.35649	Down	1.07E-47	6.02E-47
Snn	2931	2110	881.5	25.76374	10.06152	-1.35649	Down	########	########
Psat1	2079	3391	1417.5	58.31265	22.77391	-1.35643	Down	########	########
Frmd4a	3538	3807	1600	38.59595	15.07419	-1.35637	Down	########	########
LOC498972	1647	1489.5	625	32.44716	12.67447	-1.35617	Down	4.96E-81	3.33E-80
Bend6	1768	594	248.5	12.01928	4.69546	-1.35601	Down	1.14E-33	5.83E-33
Abr	4924	3182.5	1335.5	23.1437	9.041823	-1.35593	Down	########	########
Rbbp8	3135	2185	914.5	24.89721	9.728672	-1.35567	Down	########	########
Arrb2	1758	414.5	174.5	8.444611	3.302473	-1.35448	Down	1.09E-23	5.08E-23
Raph1	7305	4346.5	1823	21.28122	8.325812	-1.35392	Down	########	########
Dock11	6660	71	30	0.381387	0.14931	-1.35294	Down	3.89E-05	7.51E-05
H2afy2	1983	71.5	30	1.292097	0.506457	-1.3512	Down	3.89E-05	7.52E-05
Tnnt2	1096	253.5	106.5	8.298741	3.25287	-1.35118	Down	3.83E-15	1.61E-14

LOC100362940	2483	455.5	191	6.571155	2.576191	-1.35091	Down	6.22E-26	2.98E-25
Alad	1278	410	172	11.48334	4.503233	-1.35051	Down	1.50E-23	7.01E-23
Gorasp2	2177	5569	2347.5	91.73807	35.99687	-1.34965	Down	########	########
Itgb5	3696	22918.5	9642	221.8428	87.08524	-1.34904	Down	0	0
Cdkn2c	1462	1084.5	457.5	26.48939	10.40425	-1.34824	Down	5.01E-59	3.02E-58
Gpr180	1835	933	393.5	18.22512	7.162213	-1.34745	Down	3.98E-51	2.29E-50
Get4	2091	3491.5	1474	59.79891	23.50122	-1.34738	Down	########	########
Kctd15	2418	1369	577.5	20.29285	7.979295	-1.34664	Down	4.16E-74	2.71E-73
Myrip	2571	190	80	2.651115	1.043075	-1.34576	Down	1.22E-11	3.19E-11
LOC100912770	833	363	153.5	15.56863	6.12584	-1.34566	Down	7.29E-21	3.31E-20
Lppr5	2808	547.5	231.5	6.99463	2.752349	-1.34558	Down	1.55E-30	7.75E-30
RGD1306151	4168	990.5	418	8.493282	3.343111	-1.34513	Down	6.43E-54	3.76E-53
Uso1	3842	6789.5	2861	63.1929	24.88375	-1.34456	Down	0	0
Dennd4b	5111	1208.5	511	8.469893	3.335915	-1.34426	Down	3.49E-65	2.17E-64
Bcs1l	1685	433.5	183	9.179919	3.617974	-1.3433	Down	1.79E-24	8.44E-24
LOC100911483	564	1813.5	768	115.1331	45.37832	-1.34323	Down	1.15E-96	8.07E-96
Aagab	1087	739.5	312.5	24.37201	9.612814	-1.34219	Down	1.09E-40	5.87E-40
Exosc4	1106	456.5	192	14.73137	5.814619	-1.34114	Down	7.47E-26	3.58E-25
LOC100912484	1296	271.5	115	7.484088	2.954925	-1.3407	Down	8.81E-16	3.74E-15
Rpl3	1317	24820	10461	671.6668	265.2103	-1.34061	Down	0	0
Mfsd6	4840	1121	474	8.283664	3.271002	-1.34054	Down	1.16E-60	7.04E-60
LOC100912427	1122	4014.5	1710.5	128.4155	50.73528	-1.33976	Down	########	########
LOC100911918	781	403.5	171	18.51257	7.314654	-1.33964	Down	9.88E-23	4.57E-22
Sec61a1	3061	11472	4850	133.934	52.92736	-1.33944	Down	0	0
Gtf2i	3988	8737	3701.5	78.37228	30.98546	-1.33875	Down	0	0

Nr2f2	1571	840.5	358.5	19.22011	7.599058	-1.33872	Down	2.93E-45	1.63E-44
Exoc8	2498	548.5	232.5	7.859767	3.10836	-1.33833	Down	1.86E-30	9.28E-30
Nde1	2153	2121	897.5	35.21863	13.92955	-1.33819	Down	########	########
Slc41a3	2263	944.5	400	14.91964	5.904518	-1.33732	Down	3.58E-51	2.06E-50
Seli	2636	1527	644	20.65892	8.176521	-1.33721	Down	2.35E-82	1.58E-81
Setd3	2794	2944.5	1247.5	37.64062	14.89842	-1.33713	Down	########	########
RGD1309926	3242	1786.5	759	19.74386	7.815234	-1.33704	Down	1.05E-94	7.33E-94
Tmem158	999	548	236	19.78784	7.84393	-1.33497	Down	1.47E-29	7.27E-29
Rab6a	3380	7561	3214	79.90123	31.69545	-1.33394	Down	0	0
Syt6	1850	39.5	17	0.766965	0.304365	-1.33336	Down	0.003202	0.005225
Polr2a	6616	10309	4390.5	55.82485	22.15658	-1.33317	Down	0	0
Morc4	4076	947	403.5	8.316674	3.301454	-1.33291	Down	6.12E-51	3.52E-50
Bcl7c	986	1035.5	442.5	37.63868	14.94372	-1.33268	Down	3.66E-55	2.16E-54
Olfml3	1758	2376	1013.5	48.38874	19.21984	-1.33208	Down	########	########
Lrrc16a	5007	2618	1115	18.71563	7.438805	-1.3311	Down	########	########
Gclm	1524	844.5	358	19.76078	7.855236	-1.33091	Down	7.52E-46	4.19E-45
Tmem63b-ps1	2582	3716.5	1590	51.63799	20.52828	-1.33082	Down	########	########
Fam213a	1210	621	265	18.379	7.312732	-1.32958	Down	8.01E-34	4.12E-33
LOC689914	1562	1570.5	672.5	36.08167	14.35844	-1.32937	Down	2.10E-82	1.42E-81
Cnih2	1365	165	70.5	4.34183	1.728	-1.3292	Down	3.60E-10	8.77E-10
LOC691631	3470	1799.5	770.5	18.61021	7.408354	-1.32887	Down	3.72E-94	2.59E-93
Gadd45gip1	1212	1338	573.5	39.61446	15.78365	-1.3276	Down	1.97E-70	1.26E-69
Pde8a	2560	1336.5	573	18.71933	7.462009	-1.32689	Down	3.88E-70	2.47E-69
LOC100363332	3542	72	31	0.72706	0.289918	-1.32643	Down	4.66E-05	8.91E-05
Prrx1	1375	5824	2493.5	151.8546	60.57656	-1.32586	Down	########	########

Def8	3196	519	222	5.806551	2.316856	-1.32551	Down	2.04E-28	9.96E-28
Pgp	2535	1083	464	15.30311	6.108197	-1.32501	Down	2.65E-57	1.58E-56
Ugt1a2	2237	592	253	9.463528	3.778392	-1.32461	Down	3.09E-32	1.56E-31
Hyal2	1940	862	369	15.9105	6.352698	-1.32454	Down	4.90E-46	2.74E-45
Bag6	3659	11571	4970.5	113.5016	45.327	-1.32427	Down	0	0
Igf2r	8810	14803.5	6324	60.01097	23.9781	-1.32351	Down	0	0
Gstm7	1208	56	24	1.663572	0.664804	-1.32328	Down	0.000317	0.000569
Dgat1	1751	464	198.5	9.476355	3.787683	-1.32302	Down	1.11E-25	5.29E-25
Rbck1	2350	2599.5	1119	39.69154	15.86985	-1.32254	Down	########	########
Kdelc2	3768	3831.5	1641.5	36.35758	14.53981	-1.32225	Down	########	########
Tysnd1	2313	254.5	109	3.939842	1.575686	-1.32216	Down	1.31E-14	5.41E-14
Nfia	2200	1584.5	680	25.83501	10.33592	-1.32166	Down	1.12E-82	7.58E-82
Ywhag	1634	7372.5	3168.5	161.5886	64.71387	-1.32018	Down	0	0
Gsk3a	2155	2840	1223.5	47.26339	18.95305	-1.31829	Down	########	########
Rassf3	3290	930	399	10.10927	4.053963	-1.31827	Down	2.47E-49	1.41E-48
Klf16	2622	1299.5	558	17.73916	7.114471	-1.31811	Down	4.72E-68	2.98E-67
Agpat1	2091	5116.5	2203.5	87.75202	35.19923	-1.31789	Down	########	########
LOC364707	1200	1815	784.5	54.39787	21.82325	-1.31768	Down	2.15E-93	1.49E-92
Timm17b	850	440.5	190	18.589	7.458699	-1.31745	Down	6.17E-24	2.89E-23
Ralgds	3684	682	294.5	6.642422	2.665653	-1.31722	Down	2.54E-36	1.33E-35
Ntn3	2428	56	24	0.825786	0.331501	-1.31676	Down	0.000317	0.000569
Elovl1	1507	2583.5	1110	61.29434	24.62194	-1.31581	Down	########	########
Gpr137	1687	508	219	10.78343	4.33255	-1.31553	Down	1.81E-27	8.77E-27
Cdh2	4350	21484.5	9248	176.7418	71.01911	-1.31536	Down	0	0
Myh9	6060	15689	6760.5	92.65971	37.25289	-1.31459	Down	0	0

Ralgps2	1570	306.5	132	6.988082	2.810484	-1.31408	Down	3.82E-17	1.66E-16
Abhd14b	1603	923.5	397	20.63019	8.297234	-1.31405	Down	9.42E-49	5.33E-48
Fam65a	4162	2266	976.5	19.50463	7.846606	-1.31368	Down	########	########
Dvl3	2007	1839.5	795.5	32.90498	13.24024	-1.31338	Down	1.82E-94	1.27E-93
Camkk2	2132	1347	580	22.59121	9.090315	-1.31336	Down	3.40E-70	2.17E-69
Med25	2327	2233.5	966.5	34.47962	13.87409	-1.31335	Down	########	########
Scrib	5547	4052	1750.5	26.19091	10.54021	-1.31316	Down	########	########
Etv1	6803	2235	965.5	11.74336	4.727563	-1.31268	Down	########	########
Sh2d4a	2461	75	32.5	1.089111	0.43853	-1.3124	Down	2.76E-05	5.41E-05
Wbp1	1175	571	247.5	17.41743	7.015307	-1.31195	Down	1.72E-30	8.59E-30
LOC100912459	2397	311	134.5	4.657307	1.87621	-1.31167	Down	1.96E-17	8.52E-17
Slc27a2	2963	42.5	18.5	0.516259	0.208001	-1.31151	Down	0.001868	0.003111
Porcn	1874	265.5	115	5.072211	2.044495	-1.31087	Down	6.79E-15	2.84E-14
Slc39a7	2053	5827	2523	101.7491	41.01394	-1.31083	Down	########	########
Eef1d	2042	5432	2344.5	95.16022	38.37039	-1.31037	Down	########	########
Actr2	1326	8891	3829	239.0905	96.45498	-1.30963	Down	0	0
Arhgap24	2728	539.5	234	7.080101	2.858216	-1.30866	Down	1.09E-28	5.37E-28
Prr5	1802	182	79	3.619477	1.461186	-1.30864	Down	1.13E-10	2.81E-10
Abhd11	1151	446.5	193.5	13.91728	5.626498	-1.30657	Down	3.77E-24	1.77E-23
Plekho1	1477	1160.5	504	28.18353	11.39936	-1.3059	Down	1.05E-59	6.34E-59
LOC100911597	7404	43731.5	18971	211.4809	85.58216	-1.30515	Down	0	0
Atp13a2	3905	806.5	350.5	7.400226	2.994732	-1.30514	Down	4.65E-42	2.54E-41
Pemt	893	129	56	5.170419	2.092678	-1.30493	Down	5.86E-08	1.32E-07
Cyb561	2709	64	28	0.844107	0.341761	-1.30444	Down	0.000158	0.000287
Slc19a1	2303	327	143	5.085795	2.059406	-1.30424	Down	8.63E-18	3.77E-17

Psmg1	1146	1188	514.5	37.0166	14.99028	-1.30414	Down	1.40E-61	8.59E-61
Cd151	1668	6351.5	2754	135.9732	55.11671	-1.30276	Down	0	0
Gan	2730	358	155.5	4.693714	1.903187	-1.30231	Down	1.13E-19	5.04E-19
Chpf	2946	3917	1713.5	47.76014	19.38002	-1.30124	Down	########	########
Tp53i13	1392	552	240	14.18743	5.760226	-1.30042	Down	2.91E-29	1.44E-28
Emd	1274	1066.5	466	29.99765	12.19094	-1.29904	Down	2.15E-54	1.26E-53
Ptdss1	2547	3865.5	1681	54.20361	22.03468	-1.29861	Down	########	########
Arhgef40	6118	6951	3032.5	40.684	16.54048	-1.29846	Down	0	0
Igsf11	3675	481	210.5	4.699537	1.910768	-1.29837	Down	1.63E-25	7.75E-25
Wdtc1	3942	1799	784	16.34252	6.645576	-1.29816	Down	4.46E-91	3.09E-90
RGD1306565	5058	375	163	2.653308	1.078954	-1.29816	Down	2.15E-20	9.69E-20
Syngr2	1108	947.5	415.5	30.72016	12.49284	-1.29808	Down	2.69E-48	1.52E-47
Khsrp	2184	4878.5	2139	80.2503	32.64532	-1.29763	Down	########	########
Ptprn	2952	256	112	3.107815	1.264269	-1.2976	Down	3.07E-14	1.25E-13
Ppfibp1	3264	6421.5	2797.5	70.36105	28.62349	-1.29758	Down	0	0
Mcrs1	1912	1467.5	640.5	27.46003	11.17317	-1.29729	Down	1.84E-74	1.20E-73
Nrep	440	183.5	80	14.88479	6.058056	-1.29691	Down	1.34E-10	3.32E-10
LOC100359528	1752	99	43.5	2.032144	0.827155	-1.29677	Down	2.10E-06	4.39E-06
Mrpl12	1563	1990.5	869	45.5471	18.54347	-1.29645	Down	########	2.58E-99
Amfr	3528	8388.5	3663.5	85.00636	34.67433	-1.2937	Down	0	0
Pcyt2	1846	2069	903.5	40.07702	16.35219	-1.29329	Down	########	########
Abca3	6360	2595.5	1136.5	14.61725	5.964994	-1.29308	Down	########	########
RGD1563962	1800	284.5	124	5.646978	2.304981	-1.29272	Down	1.09E-15	4.61E-15
Zfp428	1238	246	108	7.116422	2.905311	-1.29246	Down	1.17E-13	4.46E-13
LOC100910357	1482	184.5	81	4.44725	1.818105	-1.29048	Down	1.59E-10	3.91E-10

Cpa2	1306	48.5	21.5	1.333467	0.54515	-1.29046	Down	0.001093	0.00185
Zbtb8a	1982	111	49	2.01066	0.822227	-1.29006	Down	7.52E-07	1.61E-06
Ctnna1	3643	15188	6664.5	149.2741	61.10188	-1.28867	Down	0	0
Rabep2	2222	421.5	185.5	6.800122	2.78542	-1.28767	Down	2.86E-22	1.32E-21
Pcdh7	3755	2182.5	959	20.82018	8.529149	-1.28751	Down	########	########
D2hgdh	2279	119	52	1.868705	0.765737	-1.28712	Down	2.28E-07	5.02E-07
Lsmd1	711	1026	452.5	51.75351	21.21094	-1.28685	Down	1.14E-51	6.60E-51
Myo19	3841	722.5	318.5	6.728436	2.758747	-1.28626	Down	7.00E-37	3.68E-36
Pitpna	1638	7192	3163	157.1065	64.43357	-1.28586	Down	0	0
Ece2	3010	160.5	70.5	1.907567	0.782432	-1.2857	Down	1.99E-09	4.73E-09
Ubxn6	1632	908.5	400.5	19.90976	8.166524	-1.28568	Down	6.28E-46	3.50E-45
Qsox1	2439	315	139	4.632266	1.90015	-1.2856	Down	6.36E-17	2.75E-16
Sntb2	1698	210.5	93	4.440584	1.821716	-1.28545	Down	1.07E-11	2.82E-11
Cdc42ep1	2263	4317.5	1903.5	68.47954	28.10387	-1.2849	Down	########	########
Nr1h2	1959	2148	949.5	39.36724	16.16168	-1.28442	Down	########	########
Art3	1510	36.5	16	0.858693	0.352575	-1.28421	Down	0.005486	0.008786
Ssrp1	2856	11708	5155	146.7221	60.2651	-1.28369	Down	0	0
Prkcsh	2011	6055.5	2672	108.0094	44.37422	-1.28336	Down	########	########
Triobp	6725	5007	2210.5	26.70822	10.97387	-1.28321	Down	########	########
Pgm3	2083	1247.5	549	21.43167	8.806846	-1.28305	Down	1.71E-62	1.05E-61
Ryk	1785	4687.5	2067.5	94.11073	38.69568	-1.28219	Down	########	########
Dcbld1	2369	337.5	149	5.104279	2.102548	-1.27957	Down	6.16E-18	2.70E-17
Mybl2	3710	4262.5	1883.5	41.18022	16.96577	-1.27932	Down	########	########
Mir322	95	59	26	22.10858	9.109021	-1.27924	Down	0.000317	0.000568
Aak1	7976	1260.5	558	5.667463	2.335173	-1.27917	Down	1.99E-62	1.22E-61

Ccr4	1083	47.5	21	1.576588	0.649883	-1.27855	Down	0.00155	0.002598
Ntn1	1815	335	149	6.635897	2.735887	-1.27828	Down	1.19E-17	5.21E-17
Sephs2	2342	1202.5	531	18.35368	7.567078	-1.27826	Down	6.47E-60	3.92E-59
Apbb1	2554	1254	554.5	17.58958	7.253382	-1.278	Down	2.01E-62	1.23E-61
Retsat	1855	1579	697	30.43782	12.56081	-1.27694	Down	2.95E-78	1.96E-77
Rgs12	5331	1143	507	7.674517	3.167963	-1.27652	Down	1.27E-56	7.55E-56
LOC100912917	933	219.5	97	8.435973	3.482659	-1.27637	Down	3.93E-12	1.11E-11
Lig3	3874	1634.5	723.5	15.09046	6.233972	-1.27541	Down	1.50E-80	1.00E-79
Tcirg1	2505	989	439.5	14.16101	5.852117	-1.27489	Down	3.65E-49	2.07E-48
Map1b	11884	24102.5	10661	72.49667	29.97228	-1.27429	Down	0	0
Itga3	4867	41625.5	18494.5	306.361	126.8036	-1.27264	Down	0	0
Iglon5	2539	72.5	32	1.021661	0.423268	-1.27127	Down	7.81E-05	0.000147
Npm3	904	849	375.5	33.45392	13.86559	-1.27067	Down	7.63E-43	4.18E-42
LOC100359600	1969	3643	1618.5	66.06351	27.4096	-1.26917	Down	########	########
LOC100912372	1716	59.5	26.5	1.235556	0.512704	-1.26896	Down	0.000317	0.000568
Mtch1	1807	5697.5	2533	112.7891	46.81311	-1.26864	Down	########	########
Cul4a	2859	2799	1247	35.04912	14.54836	-1.26852	Down	########	########
Tnfsf12	1576	548.5	244.5	12.47321	5.177618	-1.26847	Down	7.93E-28	3.87E-27
Twist2	1200	163.5	73	4.88574	2.028781	-1.26796	Down	3.26E-09	7.68E-09
LOC100911824	4011	1113	494	9.920229	4.119368	-1.26795	Down	4.06E-55	2.39E-54
Rab11fip3	5105	1527	681	10.71152	4.448113	-1.2679	Down	2.89E-74	1.88E-73
Jak3	3793	476.5	213	4.502945	1.870364	-1.26755	Down	3.71E-24	1.74E-23
Incenp	3154	4182	1861	47.43298	19.70912	-1.26703	Down	########	########
Asb13	1907	315.5	140	5.932268	2.46521	-1.26687	Down	1.04E-16	4.49E-16
Irak1	2749	4430.5	1975.5	57.7314	23.99211	-1.2668	Down	########	########

LOC682571	1518	1411	626	33.11477	13.76643	-1.26632	Down	1.88E-69	1.19E-68
Gnpnat1	1664	784.5	349	16.82691	6.995371	-1.2663	Down	4.06E-39	2.17E-38
Ndufaf3	1659	482.5	215	10.42527	4.334397	-1.26618	Down	1.38E-24	6.50E-24
Cacna2d2	3502	47	21	0.483354	0.200977	-1.26605	Down	0.00155	0.002598
Fut8	1728	1191.5	529.5	24.61554	10.23546	-1.26599	Down	8.92E-59	5.38E-58
Pltp	2025	97.5	43.5	1.718507	0.714754	-1.26564	Down	4.15E-06	8.53E-06
Gpx3	1488	80	36	1.933079	0.804116	-1.26542	Down	3.87E-05	7.48E-05
Pus7	2464	1139.5	508	16.5647	6.890625	-1.26541	Down	7.62E-56	4.51E-55
Ppp1r12c	2910	2411.5	1078	29.68045	12.35999	-1.26384	Down	########	########
Psmd2	2920	15843	7050.5	193.4447	80.59502	-1.26316	Down	0	0
Orai1	1184	699.5	314.5	21.25748	8.856589	-1.26315	Down	1.84E-34	9.49E-34
Timp4	1701	42	19	0.878143	0.366049	-1.26242	Down	0.003156	0.005151
LOC100912851	5317	877.5	391	5.900423	2.459591	-1.2624	Down	1.98E-43	1.09E-42
Gsdmd	1712	1271.5	569	26.61484	11.09643	-1.26213	Down	1.15E-61	7.07E-61
Mbtps1	3895	5187.5	2322.5	47.67498	19.88504	-1.26155	Down	########	########
LOC100910713	2399	2019.5	907	30.20834	12.60168	-1.26133	Down	3.21E-96	2.25E-95
Col4a1	6579	4012.5	1795.5	21.84481	9.11439	-1.26107	Down	########	########
Hadh	1660	816	364	17.56392	7.331265	-1.26048	Down	1.74E-40	9.36E-40
Inpp5f	4794	1814.5	813	13.53578	5.650704	-1.26028	Down	4.88E-87	3.34E-86
Specc1	2829	3941	1762.5	49.85064	20.8158	-1.25993	Down	########	########
Shc1	3541	14968.5	6699	151.267	63.20033	-1.25909	Down	0	0
Deptor	1551	147	66	3.389229	1.416073	-1.25906	Down	2.09E-08	4.79E-08
RGD735029	2368	1442.5	646	21.80718	9.112239	-1.25893	Down	1.27E-69	8.05E-69
Robo2	8972	3402.5	1522.5	13.57605	5.673035	-1.25887	Down	########	########
Tnpo2	4807	5276.5	2363	39.25439	16.40706	-1.25854	Down	########	########

LOC686432	1195	383.5	172.5	11.50781	4.813786	-1.25737	Down	1.30E-19	5.81E-19
Vps52	3196	2864	1286.5	32.12134	13.43836	-1.25718	Down	########	########
LOC687101	549	904.5	408.5	59.36327	24.85312	-1.25614	Down	1.12E-43	6.15E-43
Mlf2	1537	9201.5	4141.5	214.804	89.95812	-1.2557	Down	0	0
Setd7	1977	1182.5	529.5	21.386	8.956332	-1.25569	Down	1.67E-57	9.99E-57
Acat1	2186	1855.5	833.5	30.39562	12.73068	-1.25555	Down	1.27E-88	8.69E-88
Gmppb	1923	588	263.5	10.95345	4.587715	-1.25554	Down	1.73E-29	8.56E-29
Sspn	4842	723.5	323.5	5.343062	2.237956	-1.25548	Down	5.82E-36	3.04E-35
RGD1563307	822	172.5	78	7.514487	3.150573	-1.25406	Down	1.92E-09	4.57E-09
RGD1311952	1424	86	39	2.170328	0.909962	-1.25404	Down	2.28E-05	4.48E-05
Vac14	3093	1814	817.5	21.03715	8.82246	-1.25369	Down	3.32E-86	2.26E-85
Caskin2	4879	741.5	334	5.441844	2.282598	-1.25342	Down	3.38E-36	1.77E-35
Tle1	2892	497	224	6.151254	2.580686	-1.25313	Down	8.10E-25	3.83E-24
Cep97	2817	494.5	221.5	6.268918	2.631107	-1.25255	Down	5.06E-25	2.40E-24
Galt	1375	131.5	59.5	3.429894	1.439829	-1.25227	Down	1.35E-07	2.99E-07
Wdr83	1168	278	125.5	8.520336	3.579276	-1.25124	Down	1.29E-14	5.35E-14
Olr1328	924	58.5	26.5	2.275737	0.956061	-1.25116	Down	0.000447	0.000794
Aars2	3323	471.5	213	5.08582	2.136801	-1.25103	Down	1.88E-23	8.73E-23
LOC100909510	1351	1583	715	41.90645	17.62292	-1.24972	Down	4.84E-75	3.16E-74
Bst2	809	177.5	79.5	7.854979	3.303711	-1.24952	Down	6.04E-10	1.46E-09
Tom1l2	1953	693.5	313	12.73653	5.359075	-1.24892	Down	7.91E-34	4.07E-33
Lphn3	5413	654	296	4.325458	1.82021	-1.24875	Down	6.86E-32	3.46E-31
Sirpa	3880	1972.5	890	18.22556	7.669913	-1.24868	Down	3.59E-93	2.50E-92
LOC100912251	2251	113	51	1.802174	0.758431	-1.24865	Down	1.04E-06	2.21E-06
Cdc37	1604	8884.5	4015	198.5476	83.59972	-1.24791	Down	0	0

Hn1	1364	3357.5	1511.5	87.90401	37.02259	-1.24752	Down	########	########
Nefm	3067	1414	638	16.4987	6.952783	-1.24669	Down	2.31E-67	1.45E-66
Slc6a9	3211	610.5	276	6.817938	2.873392	-1.24658	Down	7.14E-30	3.55E-29
Lrsam1	4894	2086.5	941.5	15.24727	6.426304	-1.24649	Down	1.53E-98	1.08E-97
Nosip	1442	704.5	319.5	17.48674	7.370898	-1.24635	Down	3.99E-34	2.05E-33
Qpctl	2166	631.5	285	10.42642	4.395079	-1.24628	Down	5.99E-31	3.00E-30
Iqsec2	2969	1208.5	548.5	14.61801	6.162653	-1.24612	Down	3.34E-57	2.00E-56
Ubr4	15828	8008	3615.5	18.08438	7.627468	-1.24547	Down	0	0
Hsd3b7	1795	412	186	8.219269	3.469609	-1.24424	Down	8.90E-21	4.04E-20
Capg	1500	7868	3566.5	188.0822	79.43281	-1.24356	Down	0	0
Grb14	1950	794	359.5	14.55608	6.148927	-1.24322	Down	1.95E-38	1.04E-37
Stat5a	3616	459.5	209	4.555765	1.925233	-1.24266	Down	1.33E-22	6.16E-22
Wasf2	1705	2036	925.5	42.88909	18.12472	-1.24265	Down	7.09E-95	4.95E-94
Trim46	2780	215.5	98	2.779929	1.175326	-1.24199	Down	2.37E-11	6.06E-11
Ppp1ca	1392	10058	4561.5	258.7184	109.4185	-1.24153	Down	0	0
Ddr1	2834	4636	2108.5	58.68453	24.82443	-1.24122	Down	########	########
Brd3	3133	2085.5	946	23.8573	10.09369	-1.24098	Down	2.26E-97	1.59E-96
Rab3b	1159	46	21	1.424173	0.602608	-1.24083	Down	0.002186	0.003613
Cbr3	1175	464	211	14.12374	5.977801	-1.24044	Down	6.91E-23	3.20E-22
Gpsm1	3425	710.5	322.5	7.44106	3.149897	-1.2402	Down	2.41E-34	1.24E-33
LOC100910156	2635	13343.5	6062.5	181.1373	76.68641	-1.24004	Down	0	0
Traf4af1	1490	1374	623.5	32.93508	13.94525	-1.23985	Down	7.02E-65	4.35E-64
Zfp710	2290	571.5	261	8.96261	3.795789	-1.23952	Down	1.60E-27	7.75E-27
Plod1	2912	7219	3284	88.82922	37.63767	-1.23886	Down	0	0
LOC679522	999	254.5	115.5	9.086387	3.851478	-1.23829	Down	2.59E-13	9.24E-13

LOC100910396	2908	800.5	361.5	9.812274	4.15921	-1.23828	Down	7.33E-39	3.92E-38
RGD1560917	668	41	18.5	2.185117	0.926656	-1.2376	Down	0.00267	0.004377
Chd4	6578	17531	7968.5	95.32608	40.42935	-1.23747	Down	0	0
LOC294909	1989	121.5	55.5	2.18398	0.926404	-1.23725	Down	5.19E-07	1.12E-06
Rbm15b	3073	1796	818	20.94487	8.885458	-1.23708	Down	1.70E-83	1.15E-82
Tfeb	2291	469.5	214.5	7.35279	3.119433	-1.23701	Down	5.73E-23	2.66E-22
Tufm	1884	2300	1046	43.69598	18.5431	-1.23662	Down	########	########
Lrrn3	3080	629	285.5	7.29353	3.095807	-1.2363	Down	1.14E-30	5.70E-30
Sel1l	3939	3525.5	1601.5	31.98326	13.58031	-1.2358	Down	########	########
Hyal1	1350	97.5	44.5	2.582858	1.097528	-1.23471	Down	6.82E-06	1.38E-05
Clasp1	7590	3920.5	1787.5	18.48816	7.857559	-1.23445	Down	########	########
Arl2bp	2038	2654.5	1209.5	46.5232	19.7866	-1.23343	Down	########	########
Foxq1	2634	85	39	1.160614	0.493654	-1.23332	Down	3.18E-05	6.17E-05
Dab2ip	6508	4842	2212	26.65568	11.34506	-1.23238	Down	########	########
Ttl	1393	747.5	340.5	19.23401	8.189706	-1.23178	Down	8.50E-36	4.43E-35
Slc9a3r1	1492	3270.5	1496	78.65313	33.492	-1.23169	Down	########	########
Spp1	1503	246035.5	112363.5	5861.423	2496.032	-1.23161	Down	0	0
Mis18bp1	4059	783	356.5	6.876899	2.929797	-1.23096	Down	1.61E-37	8.49E-37
Fam100b	1277	3755	1718	105.4585	44.93073	-1.2309	Down	########	########
Fam115c	2760	107	48.5	1.383301	0.58946	-1.23065	Down	1.75E-06	3.67E-06
Ppfia1	5191	3682	1684	25.3748	10.81499	-1.23036	Down	########	########
Hnrnpul2	5666	8844.5	4036	55.78513	23.77746	-1.23029	Down	0	0
Ckap5	6651	7935.5	3615	42.57386	18.14678	-1.23025	Down	0	0
Fdft1	3173	5399.5	2464.5	60.80574	25.91867	-1.23022	Down	########	########
LOC690085	846	94.5	43	4.003503	1.70654	-1.23019	Down	1.13E-05	2.26E-05

Dpagt1	1840	1843	843	35.84656	15.28988	-1.22926	Down	6.67E-85	4.53E-84
Tmcc2	3716	254.5	116.5	2.453718	1.046828	-1.22894	Down	4.17E-13	1.42E-12
Masp1	5112	503.5	231	3.532108	1.50762	-1.22826	Down	3.24E-24	1.52E-23
Rogdi	1553	882	404.5	20.36494	8.694021	-1.22799	Down	1.93E-41	1.05E-40
Gmpr	1452	366.5	168	9.044898	3.862317	-1.22764	Down	4.64E-18	2.04E-17
Fbxl14	3819	1912	875.5	17.91024	7.648102	-1.22761	Down	6.37E-88	4.37E-87
Ctdsp1	2323	3793.5	1737.5	58.50858	24.99024	-1.22728	Down	########	########
Tshz1	5076	5150	2362.5	36.3781	15.54182	-1.22691	Down	########	########
Pard3b	3612	366.5	168	3.629162	1.55138	-1.22608	Down	4.64E-18	2.04E-17
Copb2	3025	9971.5	4567	117.8616	50.38855	-1.22593	Down	0	0
Arhgef19	3648	579	266.5	5.690257	2.43274	-1.22591	Down	1.30E-27	6.31E-27
Gga1	2765	2004	922	26.01543	11.12255	-1.22588	Down	4.49E-91	3.10E-90
Vash2	3761	502.5	229.5	4.769723	2.039331	-1.22581	Down	1.74E-24	8.21E-24
Insc	2341	258.5	118	3.935979	1.682889	-1.22578	Down	2.96E-13	1.05E-12
Adarb2	2720	45	20.5	0.590105	0.252456	-1.22494	Down	0.001858	0.003095
Usp28	4171	997	458	8.551529	3.659161	-1.22467	Down	2.36E-46	1.32E-45
Ppp6r1	4345	4356	2003.5	35.89916	15.38083	-1.22282	Down	########	########
Gramd4	2482	880.5	404	12.70269	5.44459	-1.22224	Down	3.64E-41	1.97E-40
Ccna2	1730	4855.5	2232	100.5196	43.08466	-1.22223	Down	########	########
Adamts8	3631	3211.5	1479	31.64822	13.56733	-1.22199	Down	########	########
LOC100912585	3068	1038.5	478.5	12.14578	5.210248	-1.22103	Down	5.93E-48	3.34E-47
Nploc4	3727	3375.5	1552	32.36483	13.88509	-1.22089	Down	########	########
LOC100362342	847	198	91	8.347309	3.5817	-1.22067	Down	2.07E-10	5.08E-10
LOC100362583	884	4096.5	1885	165.7165	71.1212	-1.22037	Down	########	########
LOC100911548	1250	200	92.5	5.73541	2.461624	-1.22029	Down	1.74E-10	4.28E-10

Rgs14	2854	602.5	278	7.559222	3.247068	-1.2191	Down	2.34E-28	1.14E-27
Slc44a1	2851	5089.5	2340	63.78568	27.4005	-1.21903	Down	########	########
RGD1309594	1887	747	345	14.18513	6.094568	-1.21878	Down	8.75E-35	4.53E-34
Tmem164	5337	946.5	436	6.338146	2.723281	-1.21872	Down	8.79E-44	4.84E-43
Slc45a1	2482	104.5	48	1.509012	0.648577	-1.21826	Down	4.70E-06	9.62E-06
Ltbr	2103	4025	1860	68.68193	29.52172	-1.21815	Down	########	########
Klk1	873	53	24.5	2.171093	0.933364	-1.21791	Down	0.000901	0.001537
Men1	2996	953.5	440	11.41327	4.907476	-1.21766	Down	6.13E-44	3.38E-43
Rtn4rl1	3309	66	30.5	0.714585	0.307328	-1.21733	Down	0.000219	0.000397
LOC100911734	2954	1490.5	688	18.07718	7.774721	-1.21731	Down	1.09E-67	6.84E-67
Abhd2	2023	7182.5	3308.5	126.9439	54.60688	-1.21704	Down	0	0
Acmsd	1011	40	18.5	1.410084	0.60693	-1.21618	Down	0.003794	0.006168
Tomm40b	927	168	77.5	6.467667	2.785898	-1.2151	Down	4.37E-09	1.03E-08
Vcl	5270	16807	7753	114.0461	49.12938	-1.21496	Down	0	0
Cask	3819	860	398	8.048861	3.469062	-1.21424	Down	1.25E-39	6.70E-39
Grn	1956	8699.5	4023.5	159.2214	68.64483	-1.21381	Down	0	0
Sapcd2	1581	1119	518.5	25.36873	10.93891	-1.21358	Down	5.10E-51	2.93E-50
LOC684934	1993	1268.5	587	22.77911	9.829834	-1.21247	Down	1.43E-57	8.54E-57
Map7d2	3783	167	77	1.574947	0.680038	-1.21162	Down	6.03E-09	1.41E-08
Reep5	2917	9531	4406	116.7382	50.41018	-1.21149	Down	0	0
LOC100912004	807	221.5	102.5	9.841796	4.25006	-1.21144	Down	2.27E-11	5.81E-11
LOC100363720	896	74	34.5	2.962992	1.280017	-1.21089	Down	0.000107	0.0002
Rcbtb2	1971	296.5	137.5	5.376952	2.324777	-1.2097	Down	1.16E-14	4.80E-14
Rap1gds1	3397	3792.5	1757	39.92816	17.26386	-1.20965	Down	########	########
Galnt10	1812	6258.5	2910.5	123.8796	53.58762	-1.20897	Down	########	########

Hdlbp	4414	26844	12431.5	217.48	94.08285	-1.20888	Down	0	0
Pcsk1n	945	344	161	13.09867	5.66717	-1.20872	Down	1.92E-16	8.24E-16
Bop1	2587	2360.5	1097	32.66349	14.13832	-1.20807	Down	########	########
Prc1	2928	8635.5	4002.5	105.4181	45.65197	-1.20737	Down	0	0
Mkl1	4378	2518.5	1173	20.62621	8.936507	-1.2067	Down	########	########
Abl1	6158	2636.5	1227	15.34001	6.64643	-1.20665	Down	########	########
Psmb10	1021	1073.5	498	37.58755	16.29347	-1.20596	Down	9.79E-49	5.54E-48
Plbd2	2217	4511.5	2097	72.87748	31.59104	-1.20596	Down	########	########
Sdpr	2148	75	35	1.246478	0.54066	-1.20506	Down	0.000125	0.000229
LOC100911978	2569	48.5	23	0.676777	0.293654	-1.20457	Down	0.002943	0.00481
Sdsl	1378	74	34.5	1.929919	0.837515	-1.20435	Down	0.000107	0.0002
Pcyox1	2292	2706.5	1258.5	42.22786	18.33746	-1.2034	Down	########	########
Gatad2a	4510	6264.5	2923	49.81116	21.6413	-1.20268	Down	########	########
LOC685487	2451	116.5	54	1.706567	0.741448	-1.20268	Down	1.64E-06	3.45E-06
LOC100912611	600	221.5	103	13.27353	5.770404	-1.20181	Down	3.61E-11	9.10E-11
Eif3i	1098	4106.5	1916	133.8326	58.24657	-1.20018	Down	########	########
Runx3	1230	306	143.5	8.945061	3.893299	-1.2001	Down	7.81E-15	3.26E-14
Acadm	1954	1121.5	522.5	20.50334	8.925568	-1.19984	Down	1.70E-50	9.71E-50
Agap1	4330	2493	1166	20.62619	8.980351	-1.19963	Down	########	########
Heatr2	3417	1209.5	564	12.66611	5.514944	-1.19956	Down	4.03E-54	2.36E-53
LOC685909	1896	1395.5	651	26.28058	11.4431	-1.19952	Down	3.97E-62	2.43E-61
Amph	2052	848	396.5	14.80717	6.447915	-1.19939	Down	2.14E-38	1.14E-37
Rps6	801	7120.5	3307.5	316.866	138.0158	-1.19904	Down	0	0
Nradd	871	109	51	4.466189	1.945611	-1.19882	Down	3.83E-06	7.88E-06
Mtm1	2536	747.5	348.5	10.53611	4.59036	-1.19866	Down	3.46E-34	1.79E-33

Itm2c	2083	8071	3783	139.0198	60.59679	-1.19798	Down	0	0
Hoxb5	1534	57.5	27	1.344466	0.586469	-1.19691	Down	0.001016	0.001722
LOC100910210	761	1510.5	706	70.92362	30.93954	-1.19681	Down	7.72E-67	4.84E-66
Icmt	1767	1131	527.5	22.85956	9.974303	-1.19651	Down	7.29E-51	4.18E-50
LOC686105	977	353.5	166	13.01319	5.681895	-1.19553	Down	1.11E-16	4.81E-16
LOC683007	4590	7908.5	3700.5	61.57869	26.88881	-1.19543	Down	0	0
Csnk1e	1251	1215	569.5	34.84288	15.21578	-1.1953	Down	6.00E-54	3.51E-53
Nudt16l1	1288	673	314	18.65411	8.147717	-1.19503	Down	7.18E-31	3.59E-30
Rfx2	3147	286.5	134.5	3.265235	1.427066	-1.19413	Down	6.83E-14	2.69E-13
Setx	8588	2344	1096	9.736592	4.255897	-1.19395	Down	########	########
Rnf5	1142	736	346	23.11593	10.1052	-1.19379	Down	4.29E-33	2.19E-32
Sympk	4031	3929.5	1844	34.94374	15.27608	-1.19376	Down	########	########
Ecm1	1913	1690	793.5	31.6755	13.85028	-1.19345	Down	4.94E-74	3.21E-73
Bckdhb	1491	754.5	353	18.07665	7.906267	-1.19306	Down	3.78E-34	1.95E-33
Uggt1	5053	6247	2926.5	44.18281	19.32449	-1.19305	Down	########	########
Adrb1	1401	101	47.5	2.588257	1.132279	-1.19275	Down	7.74E-06	1.57E-05
Spred2	1233	1312	616.5	38.16521	16.70545	-1.19194	Down	8.32E-58	4.99E-57
Wipf1	3968	4429.5	2078.5	39.94104	17.48501	-1.19175	Down	########	########
Anxa4	1988	4785.5	2240.5	86.02798	37.66514	-1.19158	Down	########	########
Mapk7	2738	498	235	6.540462	2.864244	-1.19124	Down	9.67E-23	4.47E-22
RGD1303003	1301	846.5	397	23.29901	10.20389	-1.19115	Down	6.27E-38	3.32E-37
Slc35b1	1287	2893.5	1358.5	80.42632	35.23919	-1.19049	Down	########	########
Csda	1845	13352	6272	259.0706	113.5297	-1.19028	Down	0	0
Prmt2	2014	1197	562.5	21.2896	9.337504	-1.18904	Down	6.77E-53	3.94E-52
Mfsd10	1371	1174.5	553	30.67359	13.46149	-1.18816	Down	1.46E-51	8.40E-51

Lrfn4	2368	1836.5	866	27.81304	12.20683	-1.18807	Down	2.14E-79	1.42E-78
Smarcd3	1700	714	336	15.03672	6.599864	-1.18798	Down	4.36E-32	2.20E-31
Wfdc1	1071	126.5	59.5	4.230513	1.856924	-1.18792	Down	6.83E-07	1.47E-06
LOC100909978	1406	186.5	87.5	4.7467	2.083852	-1.18767	Down	1.47E-09	3.52E-09
Cry1	2938	525.5	247.5	6.389665	2.806258	-1.18709	Down	4.94E-24	2.31E-23
Hdac9	3503	188	88.5	1.926318	0.846184	-1.1868	Down	1.24E-09	2.97E-09
Hap1	3126	459.5	216.5	5.261443	2.311579	-1.18658	Down	3.37E-21	1.53E-20
Csnk1g2	2405	6052.5	2858	90.22574	39.64778	-1.1863	Down	########	########
Socs7	2633	545	256.5	7.411064	3.257413	-1.18595	Down	5.74E-25	2.71E-24
Pgls	2376	1795	851	27.1604	11.94018	-1.18568	Down	7.91E-77	5.21E-76
Ifitm2	630	559.5	263	31.72021	13.95196	-1.18493	Down	1.74E-25	8.30E-25
Stard10	1334	414.5	195	11.1149	4.889827	-1.18464	Down	3.08E-19	1.37E-18
LOC100911832	2107	120.5	57	2.047951	0.901067	-1.18448	Down	1.83E-06	3.84E-06
LOC100911084	1284	287.5	136	8.032085	3.535597	-1.18382	Down	1.23E-13	4.68E-13
Mid1ip1	1925	2519.5	1189	46.89364	20.64188	-1.18382	Down	########	########
Cyp26b1	4552	2750.5	1300	21.64969	9.533316	-1.1833	Down	########	########
Vps8	4880	1129	532	8.262675	3.640079	-1.18264	Down	1.29E-49	7.36E-49
Hsd17b7	1704	1204.5	570	25.30994	11.15228	-1.18237	Down	2.79E-52	1.62E-51
Macf1	23521	11432.5	5384	17.35572	7.648425	-1.18218	Down	0	0
LOC100912795	2256	1161	550.5	18.47249	8.140731	-1.18215	Down	2.09E-50	1.19E-49
Anks1a	5616	931.5	441.5	5.94393	2.619645	-1.18205	Down	9.01E-41	4.86E-40
Tns1	6126	3940	1866	23.07014	10.16885	-1.18187	Down	########	########
Mtmr4	5683	2581.5	1218	16.24115	7.159693	-1.18168	Down	########	########
Reln	11453	7754	3658.5	24.20181	10.67096	-1.18143	Down	0	0
Sytl2	4044	3237.5	1525.5	28.59809	12.61049	-1.18129	Down	########	########

Fam89b	1215	1532.5	725.5	45.19842	19.9311	-1.18125	Down	4.31E-66	2.69E-65
RGD1560603	4048	511.5	243	4.537459	2.001308	-1.18094	Down	6.22E-23	2.88E-22
Adamts1	4395	5393.5	2546.5	43.87988	19.35658	-1.18074	Down	########	########
Psmc4	1376	3840	1815.5	99.89144	44.06803	-1.18063	Down	########	########
Tgm2	3526	1292	610.5	13.11237	5.786415	-1.18019	Down	2.71E-56	1.61E-55
Wdfy3	14272	4824	2282	12.08296	5.332343	-1.18013	Down	########	########
Ankrd13a	3672	6038	2861.5	58.91804	26.00907	-1.17969	Down	########	########
Dpep2	1658	42	20	0.906105	0.400022	-1.1796	Down	0.005152	0.008265
Rcor2l1	2344	665.5	315.5	10.17889	4.494407	-1.17938	Down	1.34E-29	6.65E-29
Xylt1	2718	146	69	1.924663	0.849886	-1.17926	Down	1.19E-07	2.64E-07
Cds2	1523	450.5	213.5	10.56747	4.667586	-1.17888	Down	1.42E-20	6.44E-20
Ncoa3	7462	1328	628.5	6.376583	2.816586	-1.17884	Down	1.28E-57	7.67E-57
Cmpk2	3054	81.5	38.5	0.953139	0.421035	-1.17874	Down	7.29E-05	0.000138
Npdc1	1386	1870	889	48.40805	21.38945	-1.17835	Down	1.71E-79	1.14E-78
Svs1	2867	48.5	23	0.607432	0.268468	-1.17797	Down	0.002943	0.00481
Slc10a7	1023	378	179.5	13.23765	5.852476	-1.17753	Down	1.64E-17	7.16E-17
Nisch	5575	14640.5	6955	94.14871	41.62679	-1.17743	Down	0	0
Rp2	4228	571	270	4.814289	2.129314	-1.17693	Down	9.81E-26	4.69E-25
LOC100911130	837	420.5	200	18.01446	7.96913	-1.17666	Down	4.50E-19	2.00E-18
Dazap1	1574	4734.5	2254.5	108.0275	47.79633	-1.17643	Down	########	########
Tmem8a	3455	1204.5	571.5	12.49444	5.529758	-1.176	Down	4.35E-52	2.51E-51
Cap1	2584	16296.5	7734	225.8398	99.96513	-1.1758	Down	0	0
Mmp11	2253	737.5	351	11.76054	5.206601	-1.17554	Down	2.92E-32	1.48E-31
Pfas	4488	1937.5	916.5	15.42774	6.830696	-1.17542	Down	3.31E-83	2.24E-82
Sugp2	4293	882.5	418	7.35459	3.258767	-1.17432	Down	1.13E-38	6.01E-38

Ppap2a	1290	2051	976.5	56.89079	25.21618	-1.17385	Down	7.97E-87	5.44E-86
Pkn3	2880	2427.5	1155.5	30.19758	13.39327	-1.17292	Down	########	########
Srd5a1	2638	326	154.5	4.418342	1.960316	-1.17242	Down	2.21E-15	9.35E-15
Dazap2	1778	3960	1883	79.62495	35.33772	-1.17201	Down	########	########
LOC100911729	936	124	58.5	4.711107	2.091003	-1.17187	Down	8.14E-07	1.74E-06
Ric8a	2567	1752	833	24.41813	10.83904	-1.17172	Down	1.42E-74	9.26E-74
LOC100909913	1952	171	81	3.130293	1.389566	-1.17166	Down	1.07E-08	2.49E-08
Gtpbp1	2647	1486	708	20.10157	8.928018	-1.1709	Down	3.01E-63	1.86E-62
LOC100363005	8687	5527.5	2625.5	22.73977	10.09977	-1.17089	Down	########	########
Capn2	3556	9818	4667	98.72506	43.84948	-1.17086	Down	0	0
Pafah1b3	865	346.5	165.5	14.3469	6.374875	-1.17027	Down	6.21E-16	2.65E-15
Msx1	1806	1441	688.5	28.62877	12.73127	-1.16909	Down	4.11E-61	2.51E-60
Zhx3	3621	796.5	380.5	7.878489	3.503985	-1.16892	Down	1.54E-34	7.97E-34
Dbnl	1750	3289.5	1569	67.23751	29.91103	-1.16859	Down	########	########
Mif4gd	1322	304	145	8.202494	3.649007	-1.16856	Down	3.57E-14	1.45E-13
Tns4	2498	81.5	39	1.169189	0.52017	-1.16845	Down	0.000115	0.000213
Herc2	15167	3841.5	1826.5	9.036698	4.021201	-1.16817	Down	########	########
LOC100363675	1993	1056.5	503.5	18.90573	8.4132	-1.1681	Down	1.76E-45	9.80E-45
Plekha8	6657	1261.5	599.5	6.767778	3.011739	-1.16809	Down	2.76E-54	1.62E-53
RGD1308350	3771	7834.5	3740.5	74.39075	33.11379	-1.16769	Down	0	0
Uchl1	1058	5027.5	2401	170.1938	75.76383	-1.1676	Down	########	########
Diras2	1201	304	145	9.0647	4.036127	-1.16729	Down	3.57E-14	1.45E-13
Comt	1669	4817	2304	103.4555	46.0646	-1.16728	Down	########	########
Map3k4	4908	2422.5	1156.5	17.64975	7.861898	-1.1667	Down	########	########
Ptbp3	2860	2032.5	968.5	25.3648	11.29985	-1.16652	Down	7.71E-86	5.26E-85

Yap1	1471	2165.5	1038	52.80828	23.52856	-1.16635	Down	4.48E-90	3.09E-89
Rnf126	1450	1916	920.5	47.5039	21.17345	-1.16579	Down	1.14E-79	7.60E-79
Hdac4	4015	498.5	239	4.450466	1.984047	-1.16551	Down	5.67E-22	2.60E-21
Otud5	2632	2475	1185	33.71128	15.03373	-1.16503	Down	########	########
Itfg3	2652	1727.5	827	23.294	10.392	-1.16448	Down	2.04E-72	1.32E-71
RGD1308234	3979	157	75	1.411616	0.629759	-1.16448	Down	5.70E-08	1.28E-07
Tram1	2778	7622.5	3634.5	98.00832	43.72567	-1.16442	Down	0	0
Spin1	1128	381	181.5	12.06501	5.38365	-1.16417	Down	1.59E-17	6.92E-17
B9d2	1037	409	196	14.12753	6.305125	-1.16391	Down	2.24E-18	9.86E-18
Rfng	1005	366.5	176.5	13.0844	5.840618	-1.16365	Down	1.73E-16	7.43E-16
Myg1	1207	1022.5	489.5	30.28805	13.52626	-1.16299	Down	1.18E-43	6.50E-43
Mark4	3217	1276	613.5	14.22774	6.355722	-1.16258	Down	1.33E-53	7.77E-53
Eme2	1614	353	169.5	7.836855	3.503726	-1.16138	Down	4.25E-16	1.81E-15
Stard4	5271	2098.5	1003.5	14.20795	6.352555	-1.16129	Down	6.86E-88	4.70E-87
Nedd8	795	3524	1686	158.2977	70.77807	-1.16127	Down	########	########
Oaf	1743	5080	2439	104.3931	46.68499	-1.161	Down	########	########
Sumf2	1466	1094.5	525.5	26.71362	11.9509	-1.16046	Down	2.59E-46	1.45E-45
Hmgcr	4319	8034	3845	66.39897	29.73332	-1.15908	Down	0	0
Pdzd8	7183	3902.5	1870.5	19.40726	8.694206	-1.15847	Down	########	########
Hdhd3	1204	132.5	63.5	3.922936	1.757485	-1.15842	Down	6.37E-07	1.37E-06
Arsb	1862	1387.5	667.5	26.70096	11.96361	-1.15824	Down	5.51E-58	3.31E-57
Aldh16a1	2591	931	447.5	12.85943	5.763044	-1.15792	Down	1.26E-39	6.74E-39
LOC100359512	731	831	399.5	40.70972	18.24443	-1.15792	Down	1.50E-35	7.83E-35
LOC100361417	3869	1055.5	506.5	9.743702	4.366909	-1.15786	Down	8.88E-45	4.91E-44
LOC100910875	1342	769	371	20.5846	9.227203	-1.1576	Down	1.09E-32	5.55E-32

Wdr55	1650	497	239.5	10.7919	4.837691	-1.15756	Down	7.69E-22	3.52E-21
Snx17	1944	1650.5	794	30.3296	13.59842	-1.15729	Down	1.54E-68	9.73E-68
Dctd	2021	1568.5	755.5	27.82914	12.48238	-1.1567	Down	3.88E-65	2.41E-64
LOC100912332	1546	333	159.5	7.691194	3.450612	-1.15636	Down	2.37E-15	9.99E-15
Acaa2	1619	437	209.5	9.618222	4.315557	-1.15622	Down	1.31E-19	5.85E-19
Ctif	1430	372	179	9.314259	4.180447	-1.15578	Down	1.03E-16	4.46E-16
Ndufv1	1506	1956	940	46.45944	20.85697	-1.15544	Down	3.81E-81	2.56E-80
Tmem2	6474	2262	1086.5	12.50346	5.614477	-1.1551	Down	9.58E-94	6.67E-93
Fbxw5	2345	905.5	437.5	13.85148	6.220631	-1.15491	Down	4.22E-38	2.24E-37
RGD1563348	678	1992	958.5	105.0755	47.21078	-1.15424	Down	1.79E-82	1.21E-81
LOC100361644	315	1450.5	704.5	165.6462	74.45129	-1.15374	Down	2.75E-59	1.66E-58
Myl9	1008	381	184	13.56276	6.100668	-1.15261	Down	5.92E-17	2.56E-16
Psap	2585	20072	9656.5	277.3482	124.7621	-1.15252	Down	0	0
LOC100910289	1301	1559	750.5	42.74361	19.23025	-1.15233	Down	6.72E-65	4.16E-64
Reep4	1233	1336.5	645.5	38.86809	17.48851	-1.15218	Down	1.91E-55	1.13E-54
Ebp	1071	1040.5	501	34.72645	15.62716	-1.15198	Down	9.41E-44	5.18E-43
Srpx	1827	320.5	154	6.266102	2.820618	-1.15156	Down	1.40E-14	5.77E-14
Zfp598	3275	2594	1252.5	28.37577	12.7849	-1.15022	Down	########	########
Col5a1	5551	20496	9914	132.3277	59.6246	-1.15014	Down	0	0
Pcp4l1	1600	104	50.5	2.332361	1.05124	-1.1497	Down	1.18E-05	2.36E-05
Mtmr6	3779	3420	1651	32.36809	14.59106	-1.14949	Down	########	########
Atp2a2	4471	20168	9736.5	161.2514	72.73114	-1.14867	Down	0	0
Myo9a	8575	1786.5	861.5	7.439266	3.355567	-1.1486	Down	9.54E-74	6.20E-73
Sfxn3	2699	3016.5	1458	39.99083	18.04056	-1.14843	Down	########	########
Zfp358	2031	891.5	433	15.78216	7.11969	-1.14841	Down	5.05E-37	2.66E-36

LOC100911280	4644	1111.5	538	8.563966	3.863545	-1.14835	Down	4.24E-46	2.37E-45
Slc46a1	1900	137.5	67	2.592141	1.169745	-1.14795	Down	7.95E-07	1.70E-06
Sufu	1757	447	217	9.118985	4.115787	-1.1477	Down	2.04E-19	9.11E-19
Spsb2	1257	177.5	86.5	5.062731	2.286403	-1.14684	Down	1.54E-08	3.55E-08
Pmepa1	693	12542	6101.5	651.1071	294.0656	-1.14676	Down	0	0
Cdk16	1791	5213	2528.5	104.3963	47.1502	-1.14674	Down	########	########
Elk4	10129	3048.5	1472.5	10.74963	4.855418	-1.14662	Down	########	########
LOC100912203	1038	1130	547	38.94704	17.59228	-1.14657	Down	6.78E-47	3.81E-46
Arhgap18	2292	943.5	456.5	14.71851	6.649489	-1.14632	Down	1.65E-39	8.86E-39
Mbrl	2100	2952	1433.5	50.45774	22.8074	-1.14557	Down	########	########
Srebf1	5011	407	197.5	2.90865	1.315255	-1.14501	Down	6.34E-18	2.78E-17
Gtf3a	1349	1671.5	811.5	44.34735	20.05437	-1.14493	Down	4.20E-68	2.65E-67
Hmga1	324	3933	1916	436.2554	197.3217	-1.14462	Down	########	########
Lrba	9352	1348	652.5	5.1529	2.331314	-1.14424	Down	1.06E-55	6.26E-55
Pitrm1	3178	5824.5	2829.5	65.68561	29.72065	-1.14411	Down	########	########
Ildr2	2162	1157.5	563	19.18867	8.683337	-1.14393	Down	1.98E-47	1.11E-46
Mpv17l2	1005	318	154.5	11.29507	5.114241	-1.1431	Down	2.57E-14	1.05E-13
Insr	5399	985.5	478	6.522144	2.956565	-1.14142	Down	7.07E-41	3.82E-40
Cdh18	3593	120.5	59	1.199998	0.54398	-1.14141	Down	4.43E-06	9.09E-06
Gpd2	2400	1088	527.5	16.20572	7.349956	-1.1407	Down	3.73E-45	2.07E-44
RGD1559938	1174	522	254	15.90262	7.212649	-1.14066	Down	2.65E-22	1.22E-21
Crtc1	1893	549	267.5	10.41618	4.726689	-1.13992	Down	2.12E-23	9.85E-23
Myo1b	3607	3645	1772.5	36.14749	16.40435	-1.13982	Down	########	########
Akap13	12224	4538	2201	13.25178	6.015386	-1.13946	Down	########	########
Gpc4	2669	3956	1922	52.99161	24.05667	-1.13933	Down	########	########

Prkacb	1122	1226.5	596	39.06615	17.73729	-1.13913	Down	2.92E-50	1.67E-49
Arhgef11	4584	2193.5	1069	17.1558	7.790991	-1.13882	Down	6.09E-88	4.17E-87
Galnt1	1838	6777	3292	131.5741	59.75233	-1.13881	Down	########	########
Rerg	2261	92.5	45	1.462283	0.66409	-1.13877	Down	5.37E-05	0.000102
Cdk18	1522	179.5	87.5	4.217339	1.915569	-1.13856	Down	1.30E-08	2.99E-08
Mib2	3799	816	398	7.692945	3.494907	-1.13828	Down	8.83E-34	4.54E-33
Fuca1	1515	2379.5	1160.5	56.19814	25.5463	-1.13741	Down	3.52E-95	2.46E-94
Hebp2	2549	680.5	331	9.552846	4.342501	-1.1374	Down	1.51E-28	7.39E-28
Ndst4	2619	85	41.5	1.16332	0.528867	-1.13727	Down	8.06E-05	0.000151
P2ry2	2011	308.5	150.5	5.48923	2.495706	-1.13716	Down	9.37E-14	3.64E-13
Kirrel3	3640	240.5	117	2.361965	1.074384	-1.13648	Down	5.17E-11	1.30E-10
Ccdc88b	4919	51	25	0.371815	0.169134	-1.13642	Down	0.002799	0.004582
Plod3	2775	4057.5	1979.5	52.38567	23.83166	-1.13629	Down	########	########
Foxred1	2205	469.5	229	7.610439	3.463301	-1.13583	Down	4.74E-20	2.13E-19
Card10	3124	67	33	0.77166	0.351237	-1.13552	Down	0.000641	0.001107
Map3k11	3536	1761	861	17.86732	8.13361	-1.13536	Down	1.65E-70	1.05E-69
Lgals3	948	4456.5	2183.5	168.5845	76.75786	-1.13509	Down	########	########
Bloc1s3	1750	108	53	2.210303	1.006515	-1.13488	Down	1.29E-05	2.57E-05
Mfsd5	1761	1703.5	832.5	34.63487	15.77596	-1.1345	Down	2.30E-68	1.45E-67
Gaa	3408	2608	1277	27.44483	12.50366	-1.13418	Down	########	########
LOC100909679	1297	174	85.5	4.830591	2.201251	-1.13388	Down	2.49E-08	5.68E-08
RT1-Cl	1107	931	455.5	30.15835	13.74548	-1.1336	Down	3.88E-38	2.06E-37
Rgs10	858	467	229	19.45771	8.870022	-1.13333	Down	8.61E-20	3.86E-19
Brf1	2380	933	455.5	14.02956	6.396405	-1.13314	Down	2.14E-38	1.14E-37
Kntc1	6479	4339	2115	23.91286	10.90266	-1.13311	Down	########	########

Arhgap21	6640	5819	2846.5	31.38223	14.30913	-1.13301	Down	########	########
Limd1	3937	3081.5	1506.5	28.02096	12.77861	-1.13278	Down	########	########
LOC100912343	644	51.5	25	2.847746	1.298867	-1.13257	Down	0.002799	0.004582
Ybx1	1536	10897	5339	253.8576	115.8627	-1.1316	Down	0	0
Otud4	3832	1935	945.5	18.04749	8.238147	-1.13141	Down	1.72E-77	1.14E-76
Hnrnpab	1592	5874	2875	131.9321	60.22612	-1.13133	Down	########	########
RT1-CE2	1098	119.5	58.5	3.912981	1.786593	-1.13106	Down	3.90E-06	8.02E-06
RGD1308428	6155	1809.5	886	10.52364	4.808012	-1.13012	Down	4.22E-72	2.72E-71
Ftsj1	1185	399	196	12.07515	5.51689	-1.13011	Down	4.54E-17	1.97E-16
Dym	2516	952	466	13.54986	6.191596	-1.12989	Down	7.98E-39	4.26E-38
RGD1307155	1246	132	64.5	3.79038	1.732263	-1.12968	Down	9.93E-07	2.11E-06
Bub1b	3617	2963	1448.5	29.2618	13.37527	-1.12945	Down	########	########
Pdia6	1744	16644	8131.5	340.8882	155.8504	-1.12914	Down	0	0
Smarcd1	3068	2523	1239.5	29.47809	13.47846	-1.12899	Down	3.20E-99	2.27E-98
Nr2c2	2508	949	465.5	13.5473	6.198051	-1.12812	Down	1.27E-38	6.78E-38
LOC100909609	883	271.5	132.5	10.96704	5.018576	-1.12782	Down	2.84E-12	8.27E-12
Fblim1	1475	46	23	1.12295	0.513896	-1.12775	Down	0.005583	0.008934
Gnao1	2068	707.5	348	12.26524	5.614367	-1.12738	Down	6.57E-29	3.23E-28
Sec14l1	2946	3347.5	1645	40.6675	18.61945	-1.12707	Down	########	########
RGD1566084	3412	230.5	113	2.423159	1.109675	-1.12675	Down	1.89E-10	4.65E-10
LOC100911504	912	203	100.5	7.993203	3.664829	-1.12503	Down	2.45E-09	5.81E-09
Fam60a	2617	888.5	437	12.12327	5.558936	-1.1249	Down	6.66E-36	3.47E-35
Pycr2	1582	1077.5	530	24.37286	11.17953	-1.12442	Down	3.48E-43	1.91E-42
LOC100910255	5963	9660.5	4744.5	57.93816	26.58036	-1.12415	Down	0	0
Mospd3	1037	1556	770.5	53.91716	24.73682	-1.12408	Down	7.73E-61	4.71E-60

Impdh2	1677	5774	2841.5	123.3133	56.58251	-1.1239	Down	########	########
LOC100361060	321	1456.5	722	163.1634	74.89806	-1.12332	Down	8.91E-57	5.30E-56
Hspa5	2580	30469.5	14982.5	422.1647	193.8322	-1.123	Down	0	0
LOC100361083	4534	2070.5	1018	16.30001	7.484489	-1.1229	Down	1.77E-81	1.19E-80
LOC679346	4503	530	260.5	4.200965	1.929339	-1.12261	Down	3.12E-22	1.44E-21
Pxdn	6563	9045.5	4455.5	49.33924	22.6707	-1.12191	Down	0	0
C1s	2908	1624	798.5	19.93616	9.163418	-1.12143	Down	1.94E-64	1.20E-63
Cers5	2655	3402	1676	45.80613	21.06667	-1.12058	Down	########	########
Plekhg4	3561	1350.5	665.5	13.58447	6.247985	-1.1205	Down	1.43E-53	8.36E-53
B3galt6	2137	207.5	102.5	3.469006	1.595694	-1.12034	Down	1.73E-09	4.12E-09
Crip2	1154	5432	2687	168.749	77.63177	-1.12016	Down	########	########
Mex3b	3401	556.5	275.5	5.856753	2.697241	-1.11862	Down	7.76E-23	3.59E-22
Clasp2	5570	2998.5	1476	19.20035	8.845428	-1.11813	Down	########	########
LOC100364016	1275	1262.5	625	35.50055	16.35479	-1.11813	Down	1.37E-49	7.80E-49
LOC680083	902	568	281	22.53821	10.38802	-1.11745	Down	2.77E-23	1.29E-22
Scfd2	2360	1371	677.5	20.74705	9.56454	-1.11714	Down	4.45E-54	2.61E-53
Wsb2	1575	5942	2937	135.0474	62.26646	-1.11694	Down	########	########
Pak4	2857	1980	982	24.86044	11.46333	-1.11682	Down	1.57E-76	1.03E-75
Ints3	3774	3911	1932	37.09846	17.107	-1.11677	Down	########	########
Sh2b3	3680	2995	1480	29.13075	13.43342	-1.11672	Down	########	########
Set	1519	9962	4920	234.5603	108.2237	-1.11594	Down	0	0
Parp12	3444	1403.5	694	14.58431	6.72934	-1.11588	Down	4.40E-55	2.59E-54
Dak	2403	518.5	255.5	7.714694	3.560542	-1.11551	Down	1.33E-21	6.09E-21
Fzd4	6867	294.5	146.5	1.536321	0.709191	-1.11523	Down	1.12E-12	3.49E-12
Tmem147	881	891	438.5	36.11194	16.67023	-1.1152	Down	4.18E-36	2.18E-35

Ftsjd2	3748	3114.5	1542	29.75071	13.73646	-1.11491	Down	########	########
Extl2	2016	551	271	9.740787	4.498724	-1.11452	Down	6.36E-23	2.95E-22
Fam89a	1407	238.5	118.5	6.072734	2.805179	-1.11425	Down	1.44E-10	3.55E-10
Oplah	4003	445	221	3.982056	1.839866	-1.11391	Down	2.00E-18	8.82E-18
Cln5	2549	1277.5	632.5	17.92237	8.281133	-1.11386	Down	3.09E-50	1.77E-49
Cdk4	1395	6787	3363	174.1164	80.45315	-1.11383	Down	########	########
Trap1	2264	2571.5	1274.5	40.66173	18.78867	-1.11381	Down	5.18E-99	3.67E-98
Ap2a1	2934	4624.5	2297	56.51882	26.12072	-1.11354	Down	########	########
Trerf1	3861	1299	645.5	12.07	5.578383	-1.11351	Down	1.08E-50	6.20E-50
Aldh3b1	1913	204	101.5	3.818578	1.765557	-1.11291	Down	2.77E-09	6.57E-09
Gemin5	5012	1668.5	826.5	11.89986	5.502304	-1.11284	Down	5.53E-65	3.42E-64
Ate1	4531	3764.5	1863.5	29.65272	13.71566	-1.11234	Down	########	########
Tspan3	1644	4274.5	2115.5	92.7789	42.9159	-1.11228	Down	########	########
RGD1311946	2091	1051	521.5	18.0255	8.339442	-1.11202	Down	1.69E-41	9.16E-41
Flt1	4734	1337	663	10.1062	4.675656	-1.112	Down	2.80E-52	1.62E-51
Nid2	4782	14544	7227.5	109.0115	50.46383	-1.11116	Down	0	0
Aldh5a1	3504	242	120	2.470349	1.144155	-1.11043	Down	1.02E-10	2.52E-10
Gcsh	1273	1752	868.5	49.09176	22.74022	-1.11024	Down	4.30E-68	2.72E-67
Ankrd11	8653	5800.5	2879.5	24.00499	11.12272	-1.10983	Down	########	########
M6pr	2282	3237	1605.5	50.71051	23.49894	-1.10969	Down	########	########
Psme2	1366	2052.5	1021	53.80327	24.93612	-1.10946	Down	1.17E-78	7.78E-78
Hpcal1	1721	2936.5	1460.5	61.1785	28.36955	-1.10868	Down	########	########
Eif2c2	2626	1511	752	20.62024	9.564312	-1.10833	Down	2.29E-58	1.38E-57
Mdh2	1324	8905	4426	240.8422	111.7128	-1.10829	Down	0	0
Map1s	3189	1800	898	20.24425	9.393589	-1.10776	Down	8.44E-69	5.35E-68

Lsm4	911	2126.5	1060	83.74568	38.86127	-1.10768	Down	4.86E-81	3.26E-80
LOC100910838	2579	103.5	51.5	1.438604	0.667573	-1.10767	Down	2.49E-05	4.89E-05
Tle3	4776	4390	2188	32.95304	15.29304	-1.10754	Down	########	########
2-Mar	1329	711.5	354	19.14867	8.888361	-1.10726	Down	2.45E-28	1.20E-27
Ndufb9	669	2537	1263.5	135.7487	63.02404	-1.10696	Down	1.10E-96	7.74E-96
LOC688018	9194	14009	6956.5	54.42994	25.27019	-1.10696	Down	0	0
LOC100909835	846	73.5	36.5	3.105777	1.442043	-1.10684	Down	0.000371	0.000662
Fam69b	1560	254	126	5.821833	2.705858	-1.10539	Down	3.58E-11	9.03E-11
LOC288777	481	404	203.5	30.25642	14.06625	-1.105	Down	1.91E-16	8.19E-16
Nipsnap3b	1140	805.5	400	25.20395	11.71941	-1.10475	Down	5.21E-32	2.63E-31
Pogk	6927	2557	1274.5	13.2	6.137843	-1.10473	Down	3.02E-97	2.13E-96
B4galt1	2298	1233.5	614.5	19.16411	8.911986	-1.10459	Down	6.86E-48	3.87E-47
Lbr	1863	1783.5	888.5	34.21054	15.90952	-1.10455	Down	1.95E-68	1.23E-67
Impa2	1341	184.5	92.5	4.926831	2.291223	-1.10454	Down	2.42E-08	5.52E-08
Rrm1	3038	8331	4142	97.89965	45.53814	-1.10423	Down	0	0
Suds3	2255	2747	1371.5	43.63448	20.29741	-1.10417	Down	########	########
Dtwd2	1804	160	80	3.165415	1.472586	-1.10404	Down	1.99E-07	4.37E-07
Rtkn	2588	1474	737	20.43447	9.511237	-1.1033	Down	2.24E-56	1.33E-55
Cdca7l	2599	1009	502.5	13.87721	6.460148	-1.10308	Down	1.39E-39	7.45E-39
Ptn	1370	3369.5	1678	87.77386	40.86976	-1.10276	Down	########	########
Phka1	3729	372	186	3.564459	1.660967	-1.10166	Down	2.00E-15	8.45E-15
Ppp5c	1973	2491.5	1244	45.18626	21.06127	-1.10129	Down	2.83E-94	1.97E-93
LOC681996	1391	4819.5	2413.5	124.2028	57.90009	-1.10106	Down	########	########
Dock9	9363	1338	668	5.107955	2.381778	-1.10071	Down	1.63E-51	9.39E-51
Actr5	2622	403	201.5	5.498333	2.564223	-1.10047	Down	1.12E-16	4.85E-16

Pds5a	4030	4647.5	2318.5	41.18454	19.21034	-1.10022	Down	########	########
LOC100362311	4356	755.5	377.5	6.221147	2.902978	-1.09965	Down	8.54E-30	4.24E-29
Trim2	6918	3327	1659.5	17.18158	8.017659	-1.09961	Down	########	########
Bok	1383	1458	730.5	37.71671	17.61375	-1.0985	Down	1.32E-55	7.78E-55
Adamtsl5	1051	256.5	129.5	8.760023	4.091095	-1.09845	Down	6.87E-11	1.72E-10
Ldlrad3	2258	103.5	52	1.647943	0.76967	-1.09835	Down	3.81E-05	7.37E-05
Hn1l	2747	3897.5	1942	50.55028	23.61044	-1.09829	Down	########	########
Rnaseh2a	1720	1059.5	530.5	22.00622	10.28939	-1.09675	Down	6.61E-41	3.57E-40
Fbxl6	1726	529	266	10.98478	5.137017	-1.0965	Down	5.00E-21	2.28E-20
Ubash3b	2585	917	459.5	12.70834	5.944291	-1.0962	Down	1.23E-35	6.41E-35
Arvcf	3887	771.5	387	7.106832	3.325005	-1.09585	Down	4.90E-30	2.44E-29
Glce	4869	2604.5	1303	19.09051	8.931919	-1.09581	Down	5.18E-98	3.66E-97
Sgpl1	2033	1163.5	582	20.43908	9.564498	-1.09557	Down	9.19E-45	5.08E-44
Hlcs	2172	401.5	201	6.622679	3.099194	-1.09552	Down	2.01E-16	8.64E-16
Cd2bp2	3004	2343.5	1174.5	27.86477	13.0399	-1.09551	Down	4.27E-88	2.93E-87
Dip2b	5317	874	438.5	5.885011	2.754724	-1.09514	Down	5.96E-34	3.07E-33
Slc27a4	3067	1346	675.5	15.713	7.35616	-1.09493	Down	2.78E-51	1.60E-50
Rab3il1	1134	128.5	64.5	4.070225	1.905732	-1.09476	Down	3.34E-06	6.90E-06
Acbd4	1816	373	187.5	7.359539	3.448391	-1.09369	Down	2.25E-15	9.51E-15
Sema3b	2542	186	94	2.623248	1.229646	-1.09311	Down	3.07E-08	6.98E-08
Atp6v0a2	2571	1203.5	607	16.78123	7.866353	-1.09308	Down	2.28E-45	1.26E-44
LOC498205	2114	381	192	6.465111	3.03059	-1.09308	Down	1.70E-15	7.18E-15
Pnkd	2902	950.5	478.5	11.7434	5.508594	-1.09209	Down	2.02E-36	1.06E-35
LOC361914	1784	154	78	3.089876	1.449647	-1.09185	Down	5.09E-07	1.10E-06
Hk1	3653	8431	4242.5	82.69056	38.80846	-1.09135	Down	0	0

Edem1	5686	8009	4023	50.29104	23.60815	-1.09102	Down	########	########
Rasl10a	974	116.5	58.5	4.278556	2.0085	-1.09101	Down	9.69E-06	1.94E-05
Aco2	3287	8699	4385.5	94.82631	44.54795	-1.08993	Down	0	0
Add3	4194	4233	2130.5	36.08084	16.9514	-1.08983	Down	########	########
Tsta3	1354	653.5	329.5	17.26358	8.112484	-1.08952	Down	1.80E-25	8.57E-25
Usp42	5749	792.5	398.5	4.935364	2.320594	-1.08866	Down	9.95E-31	4.97E-30
Slc25a38	1686	327	165	6.939824	3.264723	-1.08794	Down	1.80E-13	6.61E-13
Ube2m	1963	5421.5	2748.5	99.22485	46.69139	-1.08754	Down	########	########
Pus7l	2795	177	88.5	2.258467	1.062785	-1.08749	Down	3.62E-08	8.23E-08
Capn12	2932	944	473	11.45404	5.390369	-1.0874	Down	1.50E-36	7.87E-36
Glrx5	2149	714	360	11.86568	5.587088	-1.08663	Down	1.18E-27	5.76E-27
LOC100363271	954	17645	8913	662.0399	311.7771	-1.0864	Down	0	0
Ptger1	2216	705	357	11.40921	5.37459	-1.08597	Down	4.67E-27	2.26E-26
Fam126a	1798	1011.5	510.5	20.11668	9.478152	-1.08571	Down	2.01E-38	1.07E-37
Nucks1	1783	7391.5	3732.5	148.2706	69.87091	-1.08547	Down	########	########
LOC687713	2819	1458.5	738.5	18.55215	8.743707	-1.08527	Down	3.29E-54	1.93E-53
Myh1	6031	782	395	4.638064	2.18607	-1.08518	Down	5.24E-30	2.61E-29
LOC685772	1180	889.5	450.5	27.0096	12.73077	-1.08515	Down	1.01E-33	5.20E-33
Prkar2b	3182	2133.5	1078.5	23.97077	11.30233	-1.08466	Down	9.33E-79	6.20E-78
Samd4b	4298	4795.5	2433	40.0416	18.88297	-1.08441	Down	########	########
Prmt1	1201	3550	1791	105.4851	49.7591	-1.08401	Down	########	########
B4galt4	2106	60.5	30.5	1.028097	0.485018	-1.08387	Down	0.001517	0.002543
Spns2	3871	431	217	3.963244	1.869733	-1.08385	Down	2.28E-17	9.93E-17
Arrdc1	1597	679.5	344.5	15.24589	7.195621	-1.08323	Down	4.33E-26	2.08E-25
Fam213b	807	104	52.5	4.592284	2.168101	-1.08278	Down	2.82E-05	5.50E-05

Cenpa	1300	1563.5	791	43.00865	20.30559	-1.08275	Down	5.20E-58	3.12E-57
Skiv2l	3912	2097.5	1064	19.21473	9.073304	-1.08251	Down	9.83E-77	6.46E-76
Pcdhgb2	2740	67.5	34.5	0.883509	0.417261	-1.0823	Down	0.000987	0.001673
Vcp	3214	13868	6995.5	153.7813	72.63531	-1.08214	Down	0	0
Fam100a	1343	689	350.5	18.44115	8.711041	-1.08201	Down	2.74E-26	1.32E-25
Elovl5	900	2389.5	1211	95.09719	44.92581	-1.08186	Down	2.33E-87	1.59E-86
LOC691920	12900	713.5	361	1.976217	0.933615	-1.08184	Down	2.35E-27	1.14E-26
Arhgap39	4612	553.5	281	4.30496	2.034192	-1.08154	Down	2.13E-21	9.71E-21
RGD1561963	7284	1827.5	926.5	8.98329	4.246133	-1.08109	Down	2.91E-67	1.83E-66
Nfatc3	5953	3093.5	1566	18.5725	8.78321	-1.08035	Down	########	########
Sp110	1783	771	391	15.48583	7.324534	-1.08014	Down	2.45E-29	1.21E-28
Klc1	1917	5528.5	2810	103.4108	48.9235	-1.07979	Down	########	########
RGD1304952	2456	57	29	0.833976	0.394592	-1.07965	Down	0.002479	0.00408
Casc4	1628	1602	810	35.09059	16.60529	-1.07944	Down	1.53E-59	9.27E-59
Abhd16a	2022	1511	767	26.76533	12.66631	-1.07937	Down	9.69E-56	5.74E-55
Ahcyl2	5020	1500	760.5	10.67518	5.052972	-1.07906	Down	1.36E-55	8.06E-55
Tsku	2600	5385	2732	74.20014	35.12234	-1.07903	Down	########	########
Sms	1675	2037.5	1031	43.42346	20.55893	-1.07871	Down	4.94E-75	3.23E-74
Ctnnbip1	2565	1066	541	14.86081	7.036205	-1.07864	Down	7.42E-40	3.98E-39
Ephb2	2970	148	76	1.787385	0.846464	-1.07833	Down	1.29E-06	2.74E-06
Efnb1	3275	5573	2837	61.00015	28.91477	-1.07701	Down	########	########
Plcb1	5217	1233.5	627.5	8.450699	4.006876	-1.07659	Down	1.29E-45	7.18E-45
Fut11	1534	398	204	9.292644	4.411804	-1.07472	Down	1.59E-15	6.72E-15
Ifi44	2922	229.5	116.5	2.800383	1.329744	-1.07448	Down	8.81E-10	2.12E-09
Gstm2	657	66	33.5	3.607755	1.714016	-1.07372	Down	0.000874	0.001492

Prep	2743	3675.5	1870	47.91546	22.77016	-1.07335	Down	########	########
Rbm9	1971	1679.5	857.5	30.58084	14.53412	-1.07318	Down	6.27E-61	3.82E-60
LOC688430	547	51	26	3.306926	1.572133	-1.07277	Down	0.004335	0.006983
Lypla2	1536	2007.5	1026	46.84345	22.26971	-1.07276	Down	3.31E-72	2.13E-71
Cd97	3046	510.5	260	5.998193	2.851895	-1.07261	Down	1.04E-19	4.66E-19
C2cd2l	3367	899.5	459.5	9.581609	4.558358	-1.07175	Down	2.11E-33	1.08E-32
Nfe2l1	1358	2136.5	1093.5	56.46368	26.87439	-1.07109	Down	1.50E-76	9.84E-76
Zbtb7c	4065	486.5	249	4.295593	2.044875	-1.07084	Down	1.21E-18	5.37E-18
Ctps2	3272	1312	666.5	14.30763	6.814169	-1.07018	Down	1.25E-48	7.09E-48
LOC680257	842	1475	756	62.86216	29.94255	-1.06999	Down	3.28E-53	1.91E-52
Npr2	3469	1001	511	10.32248	4.917256	-1.06986	Down	5.13E-37	2.71E-36
Dalrd3	1761	430.5	220.5	8.756645	4.171517	-1.06981	Down	1.01E-16	4.36E-16
Cacnb3	2520	1598.5	818	22.72609	10.83211	-1.06904	Down	1.12E-57	6.71E-57
Nrl	717	139.5	71.5	6.972007	3.32372	-1.06878	Down	2.34E-06	4.88E-06
Rangap1	2783	5814	2967.5	74.68535	35.61524	-1.06833	Down	########	########
Gatad1	2477	3546.5	1811	51.16573	24.40089	-1.06824	Down	########	########
Lnpep	3137	988.5	506	11.25929	5.370107	-1.06809	Down	2.81E-36	1.47E-35
LOC100910506	3005	572.5	292.5	6.804582	3.245455	-1.06809	Down	7.56E-22	3.46E-21
Dennd2a	4424	1508	771.5	12.19166	5.818756	-1.06711	Down	1.10E-54	6.44E-54
Chrna7	1509	409	208.5	9.704403	4.631876	-1.06704	Down	3.39E-16	1.45E-15
RGD1563634	2595	965.5	494	13.33578	6.366815	-1.06666	Down	1.65E-35	8.56E-35
Arhgef18	6131	1716	881	10.03516	4.791238	-1.06659	Down	2.31E-61	1.41E-60
Dnm2	3463	3846.5	1973	39.84472	19.02587	-1.06643	Down	########	########
Pqlc3	1929	3026	1546.5	56.01978	26.75887	-1.06592	Down	########	########
LOC100912079	3118	587.5	300.5	6.726994	3.214075	-1.06556	Down	2.54E-22	1.17E-21

Pde3b	4381	135.5	69	1.102355	0.526864	-1.06509	Down	3.33E-06	6.89E-06
LOC683626	2634	3661	1874	49.70991	23.75903	-1.06506	Down	########	########
E2f7	3369	677	348	7.193869	3.438798	-1.06486	Down	3.70E-25	1.75E-24
Ascc1	1508	1102.5	565	26.14233	12.50003	-1.06446	Down	3.65E-40	1.96E-39
Polr3d	1921	1885.5	969	35.19495	16.83083	-1.06426	Down	5.09E-67	3.19E-66
Canx	4986	29303.5	14973	209.6921	100.2833	-1.06419	Down	0	0
Med14	4901	5479	2810	40.02217	19.14076	-1.06415	Down	########	########
LOC681173	675	181	92.5	9.570571	4.57723	-1.06413	Down	5.82E-08	1.31E-07
Cers4	3342	1847.5	945	19.76041	9.454889	-1.06348	Down	1.84E-66	1.15E-65
Slc25a4	1289	14611.5	7502	405.9665	194.3208	-1.06292	Down	0	0
Als2cl	4090	87	45	0.763262	0.365356	-1.06288	Down	0.000242	0.000436
RGD1306717	3478	1244	639	12.8107	6.132223	-1.06287	Down	6.48E-45	3.59E-44
Prrc1	1730	2917.5	1501.5	60.48547	28.95494	-1.06278	Down	########	########
Pter	2111	1519	780	25.71764	12.31232	-1.06266	Down	1.68E-54	9.89E-54
Foxo6	2540	58	30	0.822064	0.393624	-1.06243	Down	0.002785	0.00456
Dock8	7495	1428.5	734	6.823158	3.267834	-1.0621	Down	3.22E-51	1.86E-50
9-Mar	1659	319	164	6.88782	3.299359	-1.06186	Down	1.19E-12	3.69E-12
Adcy9	1983	81.5	42	1.46792	0.703503	-1.06115	Down	0.000416	0.00074
Kat7	3441	3047	1567	31.70233	15.20039	-1.06048	Down	########	########
Pkd2	4851	9120.5	4675.5	67.13089	32.19406	-1.06018	Down	0	0
Scaf1	3851	5871	3027.5	54.71614	26.2407	-1.06016	Down	########	########
Ank2	14261	546	280.5	1.371131	0.657727	-1.0598	Down	1.04E-20	4.72E-20
Osbp	4408	3020.5	1551.5	24.48383	11.74485	-1.0598	Down	########	########
Tradd	1333	430	221.5	11.54384	5.537981	-1.05969	Down	1.50E-16	6.44E-16
Pecr	1188	489.5	252.5	14.76405	7.082845	-1.05969	Down	1.69E-18	7.45E-18

Kif2c	2755	1980	1017.5	25.69157	12.32629	-1.05956	Down	1.90E-70	1.22E-69
Pfkl	2770	9983.5	5142	129.1927	62.02787	-1.05854	Down	0	0
Anp32a	1017	3872.5	1989.5	136.2173	65.407	-1.05839	Down	########	########
Kif1b	6032	3526.5	1811.5	20.91484	10.04409	-1.05818	Down	########	########
Isoc2b	1065	251.5	129.5	8.45318	4.060979	-1.05767	Down	2.89E-10	7.07E-10
Asmtl	894	327.5	170	13.14796	6.31852	-1.05718	Down	1.30E-12	4.01E-12
Ybx2	1354	80	41.5	2.126929	1.022306	-1.05694	Down	0.000371	0.000662
LOC684173	1966	222.5	114.5	4.043167	1.943764	-1.05663	Down	2.96E-09	7.00E-09
Gja6	861	56	29	2.335357	1.124527	-1.05433	Down	0.003353	0.005466
Anapc1	8895	7501	3869	30.13967	14.51931	-1.05369	Down	########	########
Sipa1l2	6690	2291.5	1180.5	12.26625	5.909579	-1.05357	Down	1.13E-80	7.57E-80
LOC688293	1474	439	228	10.70942	5.161868	-1.05291	Down	1.82E-16	7.83E-16
Stk38l	2406	1743.5	898	25.90196	12.48465	-1.05291	Down	9.01E-62	5.52E-61
Kdelr3	1308	2750	1422	75.2411	36.2719	-1.05267	Down	1.59E-95	1.12E-94
Arf5	1058	4421.5	2294	149.8362	72.23501	-1.05262	Down	########	########
Ring1	1741	913.5	473.5	18.84079	9.08461	-1.05236	Down	9.70E-33	4.94E-32
Smarcd2	2593	2354.5	1219	32.52833	15.68512	-1.0523	Down	9.58E-82	6.44E-81
Ddx54	3128	3384	1750.5	38.73947	18.6833	-1.05205	Down	########	########
Wdr18	1387	1223.5	633.5	31.62773	15.25659	-1.05176	Down	2.21E-43	1.21E-42
Pwwp2b	2283	408.5	211	6.404622	3.090021	-1.0515	Down	1.47E-15	6.21E-15
Zfyve9	6457	948.5	490.5	5.249667	2.533201	-1.05126	Down	4.04E-34	2.08E-33
Mcm7	2357	5813.5	3006	88.31043	42.61462	-1.05124	Down	########	########
LOC100362709	756	1996	1032.5	94.48214	45.59356	-1.05121	Down	7.30E-70	4.65E-69
Nudt14	816	208.5	108	9.153332	4.418843	-1.05063	Down	1.48E-08	3.42E-08
Gstk1	897	277	143.5	11.04948	5.334623	-1.05052	Down	4.50E-11	1.13E-10

Adrm1	1444	4012	2083	99.67802	48.13312	-1.05025	Down	########	########
Mst4	1882	804.5	416	15.24434	7.364087	-1.04969	Down	4.07E-29	2.01E-28
Cbl	3797	923	479	8.705977	4.206369	-1.04943	Down	6.07E-33	3.09E-32
Sgta	1260	2953	1533.5	84.1078	40.64637	-1.04911	Down	########	########
Psmd7	1588	4170	2162	93.99223	45.42367	-1.0491	Down	########	########
Alg6	3007	675	348.5	7.982522	3.858182	-1.04892	Down	6.48E-25	3.06E-24
Gpr162	2873	86.5	45	1.082448	0.523253	-1.04872	Down	0.000323	0.000579
Taf10	869	718.5	374.5	29.62792	14.32556	-1.04837	Down	9.26E-26	4.43E-25
Bcl9	4458	1326	689.5	10.6661	5.158245	-1.04808	Down	1.92E-46	1.08E-45
Kiaa0895l	2003	85	44	1.521086	0.735656	-1.048	Down	0.00029	0.00052
Ttc9	2109	634.5	329	10.75613	5.202731	-1.04782	Down	3.86E-23	1.79E-22
Gramd1a	2550	914	474.5	12.8463	6.213801	-1.0478	Down	1.08E-32	5.49E-32
Rcbtb1	3632	1279.5	663.5	12.58499	6.087637	-1.04775	Down	4.04E-45	2.24E-44
LOC100911448	965	106	55	3.938913	1.905673	-1.0475	Down	5.36E-05	0.000102
Xk	2856	156.5	81	1.961313	0.949101	-1.04719	Down	9.51E-07	2.02E-06
Man1a2	3482	2193.5	1135.5	22.51701	10.89655	-1.04714	Down	2.19E-76	1.44E-75
Pi4ka	6579	1913	993.5	10.40526	5.035818	-1.04701	Down	2.21E-66	1.38E-65
LOC100910831	351	75.5	39.5	7.733722	3.743102	-1.04693	Down	0.000717	0.001235
Kif3a	2279	1534.5	795.5	24.08142	11.65803	-1.0466	Down	8.49E-54	4.97E-53
Spag5	3881	3101	1612	28.56185	13.84471	-1.04475	Down	########	########
Trim9	3689	64	33.5	0.623597	0.302333	-1.04447	Down	0.001601	0.002674
Nes	5946	7511.5	3899.5	45.17076	21.91001	-1.0438	Down	########	########
Stoml2	1213	2008	1045.5	59.33578	28.79173	-1.04325	Down	3.82E-69	2.42E-68
Mmp19	2006	3148	1645.5	56.28813	27.33367	-1.04215	Down	########	########
Abcf2	2573	3054.5	1592	42.51242	20.64458	-1.04212	Down	########	########

Wrb	2419	749.5	389	11.04851	5.365593	-1.04204	Down	5.42E-27	2.62E-26
Ulk4	3895	282	147	2.592786	1.25981	-1.0413	Down	5.22E-11	1.31E-10
Tyw1	2557	1347.5	702.5	18.84746	9.160416	-1.04089	Down	8.34E-47	4.68E-46
S100b	1488	958	501	23.07409	11.21874	-1.04036	Down	1.70E-33	8.72E-33
Ankrd9	1481	279.5	145.5	6.76612	3.290096	-1.0402	Down	5.58E-11	1.40E-10
Zcchc2	4206	604.5	315	5.147586	2.503221	-1.04011	Down	7.37E-22	3.38E-21
Ephb6	4066	89	46.5	0.782548	0.380604	-1.03989	Down	0.000202	0.000366
Agpat3	6003	1595	833	9.512972	4.631537	-1.03841	Down	8.24E-55	4.84E-54
Fbxl17	2136	1241.5	646.5	20.76958	10.11293	-1.03827	Down	2.20E-43	1.21E-42
Rpl36	364	9409	4939	927.8166	451.7713	-1.03825	Down	0	0
Man1c1	3767	614.5	321	5.844752	2.846686	-1.03786	Down	4.58E-22	2.11E-21
Erbb2	4727	544	284.5	4.124491	2.009139	-1.03764	Down	8.55E-20	3.83E-19
Ddit4l	1107	66	34.5	2.120985	1.0336	-1.03706	Down	0.001332	0.002243
Pdia5	1765	408.5	213	8.266734	4.028607	-1.03704	Down	3.17E-15	1.33E-14
Mapt	5159	89	46.5	0.617867	0.30119	-1.03662	Down	0.000202	0.000366
Mnt	4568	1485	779	11.65942	5.687287	-1.03568	Down	1.37E-50	7.86E-50
Hipk1	7996	4645	2429.5	20.7752	10.14046	-1.03474	Down	########	########
Rab1b	1873	2666.5	1398	51.04589	24.92123	-1.03442	Down	1.84E-89	1.27E-88
Slc45a4	4620	727.5	381	5.632029	2.750016	-1.03421	Down	1.11E-25	5.30E-25
Sox13	3563	1244.5	652.5	12.52966	6.118368	-1.03413	Down	9.36E-43	5.12E-42
LOC100361545	885	4063.5	2125	164.2663	80.21489	-1.03409	Down	########	########
Mettl13	3135	990.5	518.5	11.30394	5.521726	-1.03363	Down	1.63E-34	8.41E-34
Mcm4	3339	10889.5	5703	116.7172	57.02213	-1.03342	Down	0	0
Slitrk6	4248	905	474	7.596988	3.71244	-1.03306	Down	1.29E-31	6.47E-31
Tbcd	3869	1015.5	531	9.381757	4.584724	-1.03302	Down	2.34E-35	1.21E-34

Il17ra	3845	1290	677	12.0141	5.871254	-1.03299	Down	3.98E-44	2.19E-43
Trim47	2198	2719	1427.5	44.37777	21.69405	-1.03254	Down	5.24E-91	3.63E-90
Cby1	472	173	91	13.12397	6.415889	-1.03248	Down	3.83E-07	8.32E-07
Purb	2473	2944.5	1543.5	42.55358	20.80368	-1.03244	Down	1.02E-98	7.17E-98
LOC100909548	2264	3201.5	1675	50.53398	24.70809	-1.03227	Down	########	########
Sparc	2025	25701	13479.5	454.2954	222.153	-1.03208	Down	0	0
RGD1309437	1181	497	261.5	15.06205	7.369348	-1.03131	Down	5.78E-18	2.53E-17
Rab5b	3289	4236	2223.5	46.12063	22.56608	-1.03126	Down	########	########
Glud1	2998	12048	6309.5	143.6544	70.29054	-1.0312	Down	0	0
Enpep	3822	199	104.5	1.862375	0.911322	-1.03111	Down	3.95E-08	8.96E-08
Acat3	2050	9224.5	4829.5	160.5855	78.59803	-1.03078	Down	########	########
Wrnip1	2640	1322	694.5	17.90569	8.76545	-1.03052	Down	3.81E-45	2.11E-44
Aplp1	2296	728	382	11.36264	5.563398	-1.03026	Down	1.23E-25	5.87E-25
Polm	2896	608	319	7.517452	3.681041	-1.03013	Down	1.12E-21	5.11E-21
Raly	1509	8012.5	4209.5	190.1246	93.11155	-1.02991	Down	########	########
Klhdc5	3228	737	386.5	8.167353	4.000171	-1.02981	Down	4.72E-26	2.27E-25
Eef1a1	1737	73396	38492	1510.13	740.2108	-1.02867	Down	0	0
Pmpca	1760	2604.5	1369.5	52.97084	25.96556	-1.0286	Down	7.44E-87	5.08E-86
Edem2	1898	877	460	16.52301	8.101599	-1.0282	Down	1.40E-30	6.97E-30
LOC680262	1631	563	295	12.34769	6.054697	-1.02811	Down	2.97E-20	1.34E-19
Pkm	1971	48248	25381	876.872	430.0732	-1.02778	Down	0	0
Anks1b	5579	435.5	229.5	2.795264	1.371074	-1.02768	Down	8.05E-16	3.42E-15
Rpa1	2049	3016.5	1585.5	52.60651	25.807	-1.02748	Down	########	1.52E-99
Eif1ax	3059	8658.5	4532	100.865	49.49004	-1.02722	Down	########	########
Fam20c	2960	692	366	8.383169	4.113335	-1.02719	Down	5.59E-24	2.62E-23

Klhl17	2057	128.5	67.5	2.232716	1.0958	-1.02682	Down	1.12E-05	2.25E-05
Commd5	1100	111	58.5	3.607204	1.771071	-1.02626	Down	4.19E-05	8.08E-05
Igsf9b	4242	268.5	142	2.273506	1.116343	-1.02614	Down	3.75E-10	9.12E-10
Eef1g	1433	35182	18524	878.4005	431.4214	-1.02578	Down	0	0
Fert2	3387	787	414	8.318112	4.085456	-1.02576	Down	2.05E-27	9.96E-27
Tmub1	1288	781	413.5	21.75519	10.69231	-1.02479	Down	7.22E-27	3.49E-26
Akr1b8	993	17895.5	9431	645.3849	317.2728	-1.02444	Down	0	0
Trpv4	3211	56.5	30	0.630973	0.310247	-1.02416	Down	0.005014	0.008049
Plcg1	5106	3877.5	2045	27.16511	13.3615	-1.02367	Down	########	########
Kctd10	2913	5991.5	3160.5	73.6659	36.23415	-1.02365	Down	########	########
Parp3	2123	1939.5	1023	32.71046	16.09091	-1.02351	Down	1.46E-64	9.03E-64
Fam116b	2294	360.5	191	5.637426	2.773924	-1.02311	Down	4.16E-13	1.42E-12
Ttc3	7364	7204	3790	34.90453	17.1877	-1.02204	Down	########	########
RGD1562451	2294	1210	636	18.80859	9.269666	-1.0208	Down	2.45E-41	1.33E-40
Amelx	444	638.5	339	51.56897	25.42439	-1.02029	Down	5.57E-22	2.56E-21
Cnnm4	4599	1351	715	10.5189	5.186295	-1.02021	Down	3.65E-45	2.03E-44
Tspan9	4202	440	232.5	3.748573	1.848497	-1.01999	Down	6.32E-16	2.69E-15
Gpm6b	2099	4990.5	2629.5	84.7584	41.81233	-1.01943	Down	########	########
Dpp3	2632	1689.5	893	22.9864	11.33964	-1.01941	Down	3.65E-56	2.16E-55
Rbms2	1309	2550	1351.5	69.86749	34.49933	-1.01805	Down	2.02E-83	1.37E-82
LOC679168	1161	326	172	10.03679	4.957047	-1.01775	Down	3.67E-12	1.04E-11
Hm13	1392	4096.5	2179	105.6432	52.17748	-1.0177	Down	########	########
RGD1560248	5130	2259.5	1193	15.71154	7.760539	-1.0176	Down	9.99E-75	6.52E-74
Dpp9	3514	2747.5	1456.5	27.96316	13.81541	-1.01725	Down	1.28E-89	8.81E-89
RGD1310427	6364	2833.5	1498	15.91087	7.861069	-1.01721	Down	6.12E-93	4.25E-92

Mt3	405	129.5	68.5	11.43118	5.652461	-1.01602	Down	1.25E-05	2.50E-05
Pdxdc1	2863	1633.5	863.5	20.4038	10.08933	-1.01601	Down	2.00E-54	1.18E-53
Cdk10	1629	901	479.5	19.83043	9.808425	-1.01562	Down	2.45E-30	1.22E-29
LOC685025	2374	1598.5	848.5	24.12567	11.94409	-1.01427	Down	9.78E-53	5.68E-52
Ahdc1	6103	1556	829	9.155211	4.532733	-1.01421	Down	7.13E-51	4.09E-50
Fbf1	3696	542.5	289	5.269418	2.608898	-1.0142	Down	9.55E-19	4.23E-18
Atp2c1	4946	3858	2044	27.85405	13.79736	-1.01349	Down	########	########
Nudt3	2082	2475.5	1318	42.59904	21.10207	-1.01344	Down	5.97E-80	3.99E-79
Fkbp2	651	1348	717.5	74.24291	36.78139	-1.01328	Down	1.71E-44	9.44E-44
Slc47a1	1749	805	426.5	16.43396	8.141753	-1.01327	Down	1.36E-27	6.59E-27
Gpx4	1025	4889	2599.5	170.7019	84.58045	-1.01308	Down	########	########
Ptdss2	2190	1284	683	21.00882	10.41118	-1.01286	Down	1.86E-42	1.02E-41
RGD1310769	1683	1484.5	789	31.57082	15.64551	-1.01284	Down	7.33E-49	4.16E-48
Rps7	666	12298.5	6536	661.2868	327.7384	-1.01273	Down	0	0
Pak1	2539	2080.5	1107.5	29.3538	14.54952	-1.01258	Down	1.37E-67	8.66E-67
Lmtk2	8223	2370.5	1261	10.32297	5.119442	-1.0118	Down	8.26E-77	5.43E-76
Tfap4	2136	301.5	161	5.065763	2.512723	-1.01153	Down	5.51E-11	1.38E-10
Tbrg1	1519	2047	1091.5	48.30708	23.96304	-1.01142	Down	2.69E-66	1.68E-65
Upk1b	1801	53	28.5	1.057172	0.524583	-1.01097	Down	0.005436	0.008708
LOC498330	1514	108	57.5	2.557493	1.269479	-1.01049	Down	6.65E-05	0.000126
Pank3	3030	2725	1445	32.0473	15.90991	-1.01028	Down	8.22E-89	5.65E-88
Xpr1	2410	1223	650.5	18.14246	9.009988	-1.00977	Down	1.31E-40	7.06E-40
Rcan3	790	223	119	10.12552	5.029153	-1.00961	Down	1.53E-08	3.51E-08
Kif7	4490	477	255	3.806962	1.891122	-1.0094	Down	1.41E-16	6.08E-16
Farp1	4564	5422	2893.5	42.58851	21.15751	-1.00929	Down	########	########

Srprb	914	1551	826	60.72752	30.18119	-1.0087	Down	9.07E-51	5.20E-50
LOC100362836	2449	688	366.5	10.0388	4.989635	-1.00858	Down	1.65E-23	7.67E-23
Mapk14	3452	2703.5	1440	28.00796	13.9239	-1.00827	Down	4.74E-87	3.24E-86
Leprel1	2387	1307.5	697.5	19.61319	9.757206	-1.00728	Down	6.61E-43	3.62E-42
Dcxr	879	194	103.5	7.906684	3.933771	-1.00716	Down	1.08E-07	2.41E-07
Arhgef7	4379	3272.5	1748	26.78888	13.3283	-1.00714	Down	########	########
Herpud2	2085	867.5	462.5	14.88479	7.406163	-1.00704	Down	4.39E-29	2.16E-28
Zfp771	2837	749.5	402.5	9.489968	4.726133	-1.00574	Down	6.85E-25	3.24E-24
Arl2	893	583	312	23.39018	11.6487	-1.00573	Down	7.24E-20	3.25E-19
LOC688146	1679	165.5	88	3.524954	1.756307	-1.00506	Down	1.13E-06	2.39E-06
Slc38a10	4699	2830.5	1512.5	21.54855	10.74041	-1.00454	Down	2.29E-90	1.58E-89
Nr2c2ap	1544	2063.5	1101.5	47.7593	23.82858	-1.00309	Down	1.44E-66	9.00E-66
Slc35c1	3027	921	492.5	10.9083	5.444452	-1.00257	Down	1.34E-30	6.70E-30
Rpn1	2214	11632.5	6221	187.9464	93.80612	-1.00257	Down	0	0
RGD1309534	2502	598	320.5	8.542881	4.266156	-1.00179	Down	2.42E-20	1.09E-19
Cdk2ap1	1252	6004	3217.5	171.785	85.78707	-1.00177	Down	########	########
Rt1.aa	1617	2641.5	1418	58.53087	29.23179	-1.00166	Down	2.31E-83	1.56E-82
LOC681658	1601	615.5	329.5	13.77091	6.878891	-1.00138	Down	6.84E-21	3.11E-20
Postn	3177	16456.5	8788.5	184.8438	92.35374	-1.00106	Down	0	0
LOC100909645	3075	178	95.5	2.077695	1.038794	-1.00007	Down	4.36E-07	9.47E-07
